# Supplementary material for: Stereoselective chemical N-glycoconjugation of amines via CO2 incorporation
Source: Nat Commun. 2024 Nov 29;15:10373. doi: 10.1038/s41467-024-54523-4 (PMC11606974; doi:10.1038/s41467-024-54523-4)
Supplement: Supplementary file 1 — Supplementary Information [file 41467_2024_54523_MOESM1_ESM.pdf]

Stereoselective Chemical *N*-Glycoconjugation of Amines via CO_2 Incorporation

Zihan Peng,[1,#] Qian Xiao,[1,#] Yan Xia,[1] Mingyu Xia,[1] Jia Yu,[1,2] Pengfei Fang,[1] Yu Tang,[1]* and Biao Yu[1]*

[1]State Key Laboratory of Chemical Biology, Shanghai Institute of Organic Chemistry, University of Chinese Academy of Sciences, Chinese Academy of Sciences, Shanghai 200032, China

[2]Key Laboratory of Structure-Based Drugs Design and Discovery of Ministry of Education, Shenyang Pharmaceutical University, Shenyang 110016, China

[#]These authors contributed equally: Zihan Peng, Qian Xiao.

Corresponding authors:

E-mails: tangyu@sioc.ac.cn; byu@sioc.ac.cn

Supplementary information

Table of Contents

1. General information ………………………………………………………….. 3

2. General procedures for the preparation of substrates ……………………… 4

3. Reaction optimization for the stereoselective *N*-glycoconjugation ……….. 23

4. A summary schematic diagram of the scope of glycosyl halides……..……..27

5. Additional examples ……………………………………..…………..….. 28

6. Unsuccessful examples ……………………………………..………… 29

7. Identification of a common side reaction …………………………………… 30

8. General procedure for the stereoselective *N*-glycoconjugation ……………. 31

9. Competition experiments ……………………..…………..……..…… 108

10. Reactivity test of equatorial anomer of glycosyl chloride….………………113

11. NMR experiments………………………………………………………..114

12. Secondary α-deuterium kinetic isotope effects determination……………..115

13. Kinetic studies…………………………………………………………117

14. The scale-up reactions ……………………………………………………… 128

15. Deprotection ………………………………………………………………. 130

16. Preparation of crizotinib glucoside GA95 …………………………………. 133

17. Bioactivity study on crizotinib glucosides ………………………………… 134

18. Pharmacokinetic analysis of crizotinib glucosides…………………………138

19. NMR spectra ………………………..……………………………….….. 140

20. References ……………………..…………………………..……… 244

1. General information

All chemicals were purchased from commercial suppliers and used without further purification unless otherwise noted. All amines were commercially available (from Bidepharm, Adamas, Macklin and others) unless otherwise noted. Dimethyl sulfoxide (anhydrous), *N,N*-dimethylformamide (anhydrous), acetonitrile (anhydrous) were used as received from J&K Scientific. All purification procedures of products were carried out using reagent grade solvents. Thin layer chromatography (TLC) was performed on TLC Silica Gel 60 F254 (Merck). The TLC plates were visualized with UV light and/or by staining with EtOH/H_2SO_4 (8%, v/v). Flash column chromatography was performed on Silica Gel 60 (40-64 μm, Fluka, Canada). NMR spectra were measured on Bruker AM 400, Agilent 500 or 600 MHz NMR spectrometer at 25 °C. ^{1}H and ^{13}C NMR signals were calibrated to the residual proton and carbon resonance of the solvent ($CDCl_3$: δH = 7.26 ppm; δC = 77.16 ppm; CD_3OD: δH = 3.31 ppm; δC = 49.00 ppm). Multiplicities are recorded as: s = singlet, d = doublet, t = triplet, dd = doublet of doublets, td = triplet of doublets, br s = broad singlet, m = multiplet. ^{19}F NMR spectra were recorded on Bruker 400 MHz spectrometer with complete proton decoupling. High-resolution mass spectra were recorded with IonSpec 4.7 Tesla FTMS or APEXIII 7.0 Tesla FTMS. Optical rotations were measured on an Anton Paar MCP5500 S2 polarimeter.

2. General procedures for the preparation of substrates

2.1 Amines used in this study

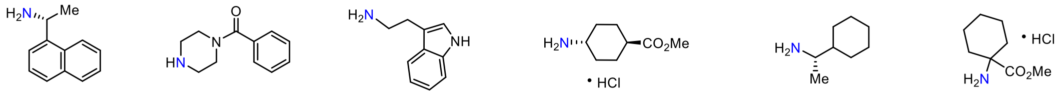

A1 A2 A3 A4 A5 A6

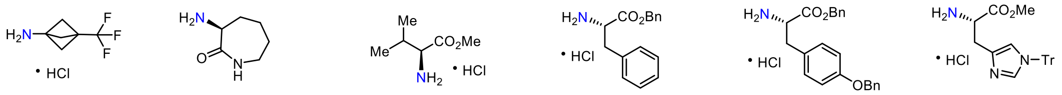

A7 A8 A9 A10 A11 A12

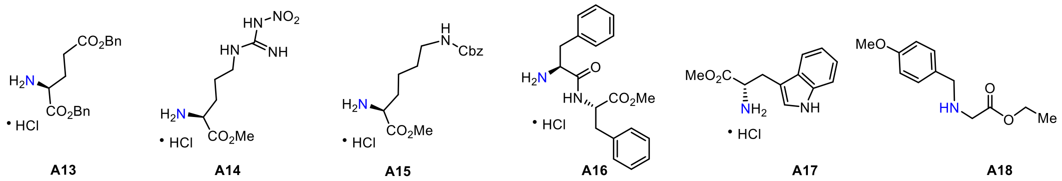

A13 A14 A15 A16 A17 A18

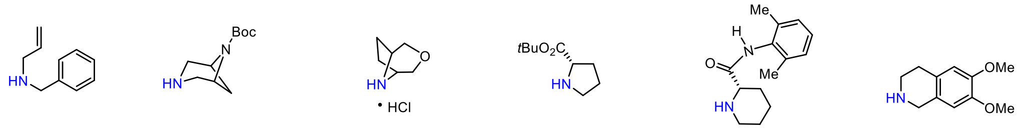

A19 A20 A21 A22 A23 A24

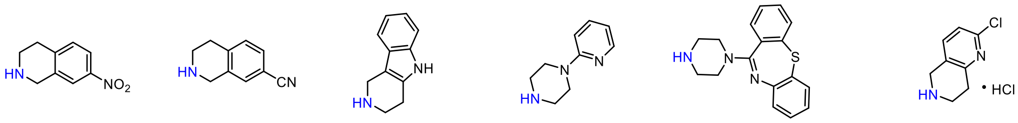

A25 A26 A27 A28 A29 A30

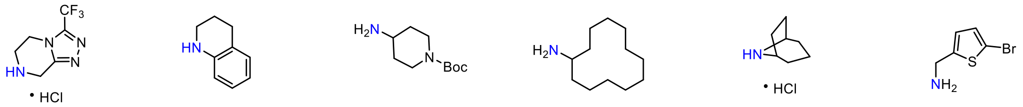

A31 A32 A33 A34 A35 A36

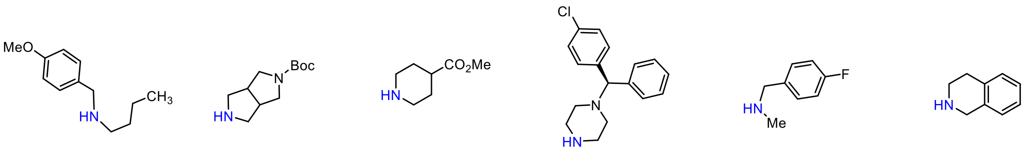

A37 A38 A39 A40 A41 A42

Figure S1

2.2 Amine-containing drugs used in this study

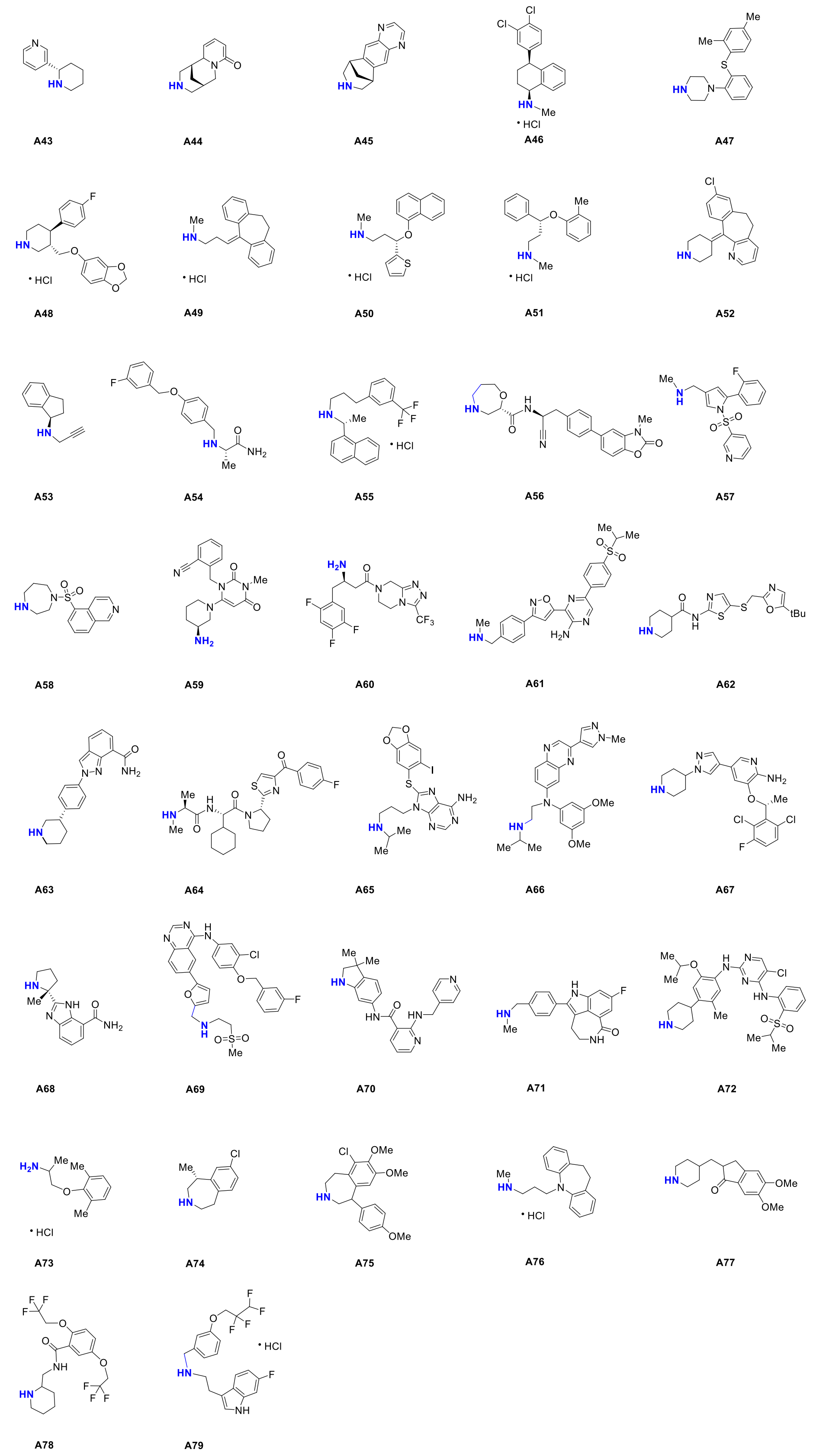

Figure S2

2.3 Glycosyl halides and epoxide used in this study

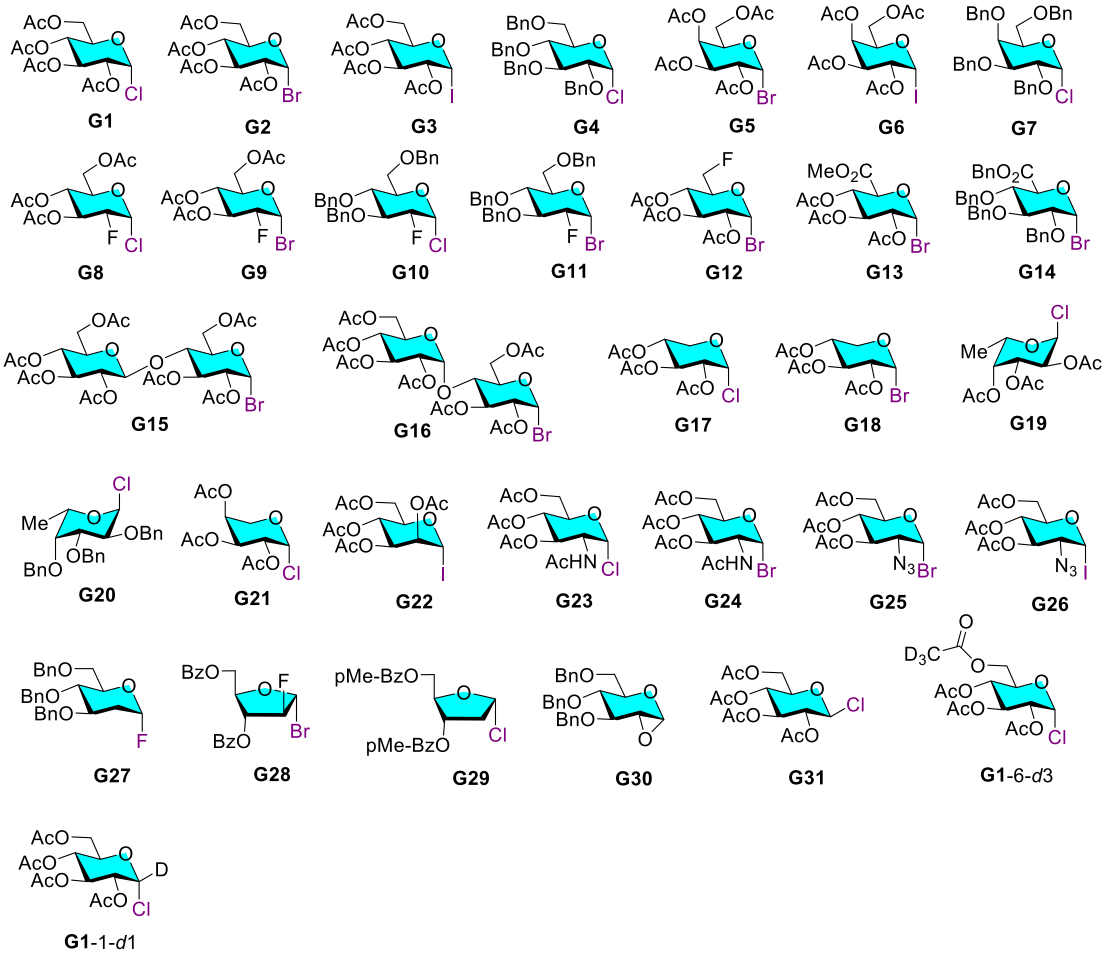

Figure S3

Commercially available glycosyl halides **G2**, **G5**, **G13**, **G15**, **G16**, **G23**, **G28**, **G29** and **G31** were purchased from local suppliers.

Glycosyl halides **G1**[1], **G3**[2], **G4**[3], **G6**[2], **G7**[3], **G8**[4], **G9**[5], **G11**[6], **G12**[7], **G14**[8], **G17**[1], **G18**[9], **G19**[10], **G20**[11], **G21**[1], **G22**[2], **G24**[12], **G25**[13], **G26**[14], **G27**[15] and glycosyl epoxide **G30**[16] are known compounds, their synthesis and characterization data were provided below.

Glycosyl halides **G10, G1**-6-*d*3 and **G1**-1-*d*1 are new compounds, and were synthesized according to the method below.

2.3.1 Synthesis of G1

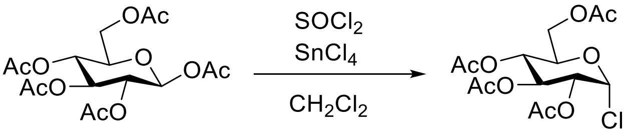

S1 **G1**

Figure S4

To a solution of commercially available **S1** (4.68 g, 12.0 mmol) in dry CH_2Cl_2 (40 mL) was added $SOCl_2$ (2.0 mL, 24 mmol) and $SnCl_4$ (1M in CH_2Cl_2, 12.0 mL, 12.0 mmol), and the mixture was stirred at RT for 20 h. After that, the resulting mixture was washed with saturated $NaHCO_3$ (100 mL), then dried over anhydrous Na_2SO_4 and filtered. The solvent was removed in vacuo and the residue was purified by silica gel column chromatography (petroleum ether/EtOAc = 3:1) to give **G1** as a white solid (4.00 g, 81%). The characterization data are consistent with the literature[1].

^{1}H NMR (500 MHz, $CDCl_3$): δ 6.29 (d, J = 4.2 Hz, 1H), 5.56 (t, J = 9.8 Hz, 1H), 5.19 – 5.10 (m, 1H), 5.01 (dd, J = 10.4, 4.1 Hz, 1H), 4.35 – 4.27 (m, 2H), 4.17 – 4.08 (m, 1H), 2.10 (s, 6H), 2.05 (s, 3H), 2.04 (s, 3H);

^{13}C NMR (126 MHz, $CDCl_3$): δ 170.5, 169.9, 169.5, 90.1, 70.7, 70.4, 69.4, 67.4, 61.1, 20.7, 20.6, 20.5.

2.3.2 Synthesis of G3

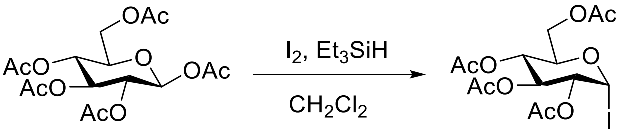

S1 **G3**

Figure S5

To a solution of commercially available **S1** (4.68 g, 12.0 mmol) in dry CH_2Cl_2 (60 mL) was added I_2 (9.16 g, 36 mmol) and Et_3SiH (2.88 mL, 18 mmol), and the mixture was stirred at 60 °C for 2 h. After that, the reaction was diluted with ethyl acetate (100 ml), and extracted with water (300 mL), 10% $Na_2S_2O_3$ (200 mL), and brine (300 mL). The organic layer was dried over Na_2SO_4. The solvent was removed in vacuo and the residue was purified by silica gel column chromatography (petroleum ether/EtOAc =

2:1) to give **G3** as a white solid (4.34 g, 79%). The spectroscopic data are consistent with the literature[2].

^{1}H NMR (500 MHz, $CDCl_3$): δ 6.99 (d, *J* = 4.2 Hz, 1H), 5.47 (t, *J* = 9.8 Hz, 1H), 5.18 (t, *J* = 9.8 Hz, 1H), 4.34 (dd, *J* = 4.2, 12.6 Hz, 1H), 4.21 (dd, *J* = 4.2, 9.8 Hz, 1H), 4.11 (dd, *J* = 2.1, 12.6 Hz, 1H) 4.06 (dq, *J* = 2.1, 9.8 Hz, 1H), 2.10 (d, *J* = 4.9 Hz, 6H), 2.05 (s, 3H), 2.03 (s, 3H).

^{13}C NMR (126 MHz, $CDCl_3$): δ 170.65, 169.99, 169.75, 169.62, 75.01, 73.05, 71.84, 70.41, 67.01, 60.97, 20.97, 20.81, 20.76, 20.70.

2.3.3 Synthesis of G4

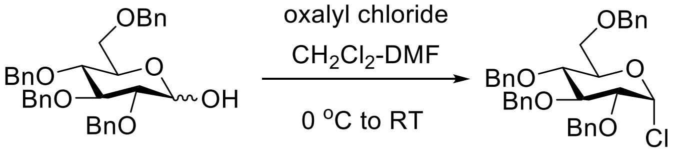

S2 **G4**

Figure S6

A solution of oxalyl chloride (14.9 g, 117 mmol) in dichloromethane (48 mL) was added dropwise to a stirring solution of commercially available **S2** (21.2 g, 39 mmol) in dichloromethane (144 mL) and DMF (48 mL), and the resulting mixture was stirred under argon for 30 min at 0 °C. The external cooling was removed, and the reaction mixture was allowed to slowly warm to rt and stirred for additional 3 h at rt. After that, the resulting mixture was concentrated in vacuo. The residue was dissolved in ethyl acetate (300 mL) and washed with saturated $NaHCO_3$ (500 mL), then dried over anhydrous Na_2SO_4 and filtered. The solvent was removed in vacuo and the residue was purified by silica gel column chromatography (petroleum ether) to give **G4** as a colorless syrup (14.7 g, 67%). The spectroscopic data are consistent with the literature[3].

^{1}H NMR (400 MHz, $CDCl_3$): δ 7.33 – 7.24 (m, 18H), 7.16 – 7.12 (m, 2H), 6.07 (d, *J* = 3.7 Hz, 1H), 4.97 (d, *J* = 10.8 Hz, 1H), 4.83 (d, *J* = 10.8, Hz, 1H), 4.82 (d, *J* = 10.8, Hz, 1H), 4.71 (q, *J* = 11.8 Hz, 2H), 4.57 (d, *J* = 12.1 Hz, 1H), 4.50 (d, *J* = 10.9, Hz, 1H), 4.46 (d, *J* = 11.9 Hz, 1H), 4.09 – 4.06 (m, 1H), 4.03 (t, *J* = 9.2, Hz, 1H), 3.79 –

3.69 (m, 3H), 3.64 (dd, J = 10.8, 1.9 Hz, 1H).

2.3.4 Synthesis of G6

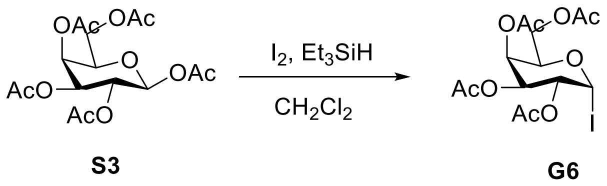

Figure S7

To a solution of commercially available **S3** (5.85 g, 15.0 mmol) in dry CH_2Cl_2 (60 mL) was added I_2 (11.4 g, 45 mmol) and Et_3SiH (3.6 mL, 22.5 mmol), and the mixture was stirred at 60 °C for 2 h. After that, the reaction was diluted with CH_2Cl_2 (100 ml), and extracted with water (300 mL), 10% $Na_2S_2O_3$ (200 mL), and brine (300 mL). The organic layer was dried over Na_2SO_4. The solvent was removed in vacuo and the residue was purified by silica gel column chromatography (petroleum ether/EtOAc = 2:1) to give **G6** as a white solid (4.27 g, 62%). The spectroscopic data are consistent with the literature[2].

^{1}H NMR (500 MHz, $CDCl_3$): δ 7.10 (d, J = 4.0 Hz, 1H), 5.49 (d, J = 2.5 Hz, 1H), 5.28 (dd, J = 3.0, 10.5 Hz, 1H), 4.37 (dd, J = 4.0, 11.0 Hz, 1H), 4.26-4.18 (m, 2H), 4.11 (dd, J = 6.5, 11.0 Hz, 1H), 2.15 (s, 3H), 2.11 (s, 3H), 2.06 (s, 3H), 2.00 (s, 3H).

^{13}C NMR (126 MHz, $CDCl_3$): δ 170.44, 170.02, 169.99, 169.88, 75.31, 73.75, 69.87, 67.64, 66.70, 60.77, 21.05, 20.76, 20.70.

2.3.5 Synthesis of G7

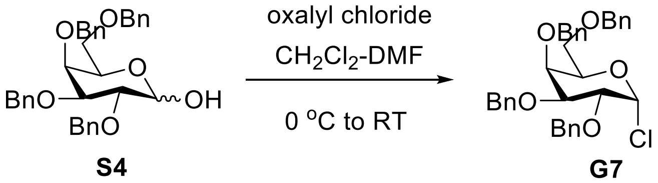

Figure S8

A solution of oxalyl chloride (2 mL, 23 mmol) in dichloromethane (10 mL) was added dropwise to a stirring solution of commercially available **S4** (4.4 g, 8.1 mmol) in dichloromethane (30 mL) and DMF (0.2 mL), and the resulting mixture was stirred

under argon for 30 min at 0 °C. The external cooling was removed, and the reaction mixture was allowed to slowly warm to rt and stirred for additional 3 h at rt. After that, the resulting mixture was concentrated in vacuo. The residue was dissolved in ethyl acetate (300 mL) and washed with saturated $NaHCO_3$ (500 mL), then dried over anhydrous Na_2SO_4 and filtered. The solvent was removed in vacuo and the product is used directly without further purification (> 95% NMR yield). The spectroscopic data are consistent with the literature[3].

^{1}H NMR (500 MHz, $CDCl_3$): δ 7.42 – 7.20 (m, 20H), 6.14 (*d*, J = 3.8 Hz, 1H), 4.94 (d, *J* = 11.3 Hz, 1H), 4.85 (d, *J* = 11.7 Hz, 1H), 4.78 – 4.72 (m, 2H), 4.71(d, *J* = 11.6 Hz, 1H), 4.56 (d, *J* = 11.3 Hz, 1H), 4.48 (d, *J* = 11.8 Hz, 1H), 4.40 (d, *J* = 11.8 Hz, 1H), 4.25 – 4.21 (m, 1H), 4.21 (dd, *J* = 9.8, 3.8 Hz, 1H), 4.01 – 3.99 (m, 1H), 3.97 (dd, *J* = 9.7, 2.8 Hz, 1H), 3.55 (dd, *J* = 9.4, 6.9 Hz, 1H), 3.53 (dd, *J* = 9.4, 6.1 Hz, 1H).

2.3.6 Synthesis of G8

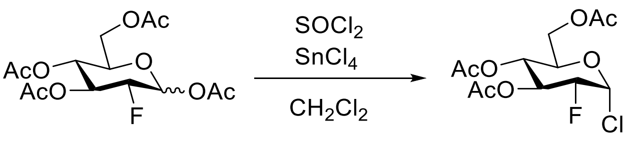

S5 **G8**

Figure S9

To a solution of **S5**[17] (200 mg, 0.57 mmol) in dry CH_2Cl_2 (2 mL) was added $SOCl_2$ (60 μL, 1.14 mmol) and $SnCl_4$ (1M in CH_2Cl_2, 0.6 mL, 0.6 mmol), and the mixture was stirred at RT for 24 h. After that, the resulting mixture was washed with saturated $NaHCO_3$ (10 mL), then dried over anhydrous Na_2SO_4 and filtered. The solvent was removed in vacuo and the residue was purified by silica gel column chromatography (petroleum ether/EtOAc = 3:1) to give crude **G8** as a colorless syrup (> 95% yield), which was immediately used in the glycosylation reactions without further purification and characterization.

2.3.7 Synthesis of G9

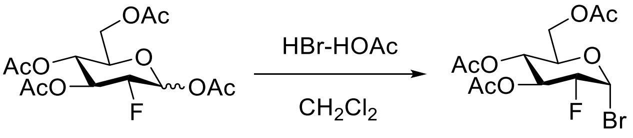

S5 **G9**

Figure S10

To a solution of **S5**[17] (200 mg, 0.57 mmol) in dry CH_2Cl_2 (4 mL) was added 33% HBr/HOAc (4 mL), and the mixture was stirred at 0 °C for 5 h. After that, the resulting mixture was washed with saturated $NaHCO_3$ (50 mL), then dried over anhydrous Na_2SO_4 and filtered. The solvent was removed in vacuo and the residue was purified by silica gel column chromatography (petroleum ether/EtOAc = 1:1) to give crude **G9** as a white solid (230 mg, 99% yield). The characterization data are consistent with the literature[18].

^{1}H NMR ($CDCl_3$, 500 MHz) δ: 6.51 (dd, J = 4.6 Hz, 1.1 Hz, 1H), 5.59 (ddd, J = 11.3 Hz, 9.8 Hz, 9.4 Hz, 1H), 5.09 (t, J = 9.8 Hz, 1H), 4.52 (ddd, J = 49.3 Hz, 9.4 Hz, 4.6 Hz, 1H), 4.37 – 4.21 (m, 2H), 4.09 (dd, J = 10.6 Hz, 3.0 Hz, 1H), 2.07, 2.06, 2.03 (s, 9H).

2.3.8 Synthesis of G10

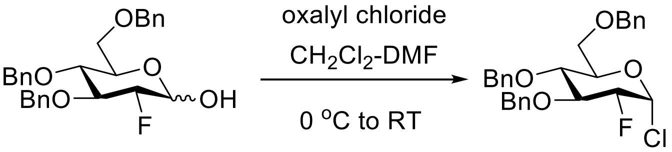

S6 **G10**

Figure S11

A solution of oxalyl choloride (190 mg, 1.46 mmol) in dichloromethane (3.0 mL) was added dropwise to a stirring solution of **S6**[19] (220 mg, 0.49 mmol) in dichloromethane (9.0 mL) and DMF (3.0 mL), and the resulting mixture was stirred under argon for 30 min at 0 °C. The external cooling was removed, and the reaction mixture was allowed to slowly warm to rt and stirred for additional 3 h at rt. After that, the resulting mixture was concentrated in vacuo. The residue was dissolved in ethyl acetate (50 mL) and washed with saturated $NaHCO_3$ (50 mL), then dried over anhydrous Na_2SO_4 and

filtered. The solvent was removed in vacuo and the residue was purified by silica gel column chromatography (petroleum ether/EtOAc = 4:1) to give **G10** as a colorless syrup (214 mg, 94%).

$[\alpha]_D^{25}$ = 107.0 (*c* 1.0, $CHCl_3$)

^{1}H NMR (500 MHz, $CDCl_3$) δ 7.49–7.31 (m, 13H), 7.25–7.19 (m, 2H), 6.29 (d, *J* = 4.1 Hz, 1H), 4.97 (d, *J* = 11.0 Hz, 1H), 4.92 (d, *J* = 10.7 Hz, 1H), 4.83 (d, *J* = 11.1 Hz, 1H), 4.81–4.67 (m, 1H), 4.65 (d, *J* = 11.9 Hz, 1H), 4.58 (d, *J* = 11.0 Hz, 1H), 4.54 (d, *J* = 12.1 Hz, 1H), 4.28–4.15 (m, 2H), 3.89–3.81 (m, 2H), 3.77–3.70 (m, 1H).

^{13}C NMR (126 MHz, $CDCl_3$) δ 138.02, 137.82, 137.62, 128.53, 128.51, 128.11, 128.03, 128.00, 127.97, 127.95, 91.19 (d, *J* = 2.1 Hz), 90.51 (d, *J* = 222.2 Hz), 80.15 (d, *J* = 15.8 Hz), 75.61 (d, *J* = 8.6 Hz), 75.43, 75.29 (d, *J* = 2.9 Hz), 73.55, 73.39, 67.50.

^{19}F NMR (376 MHz, $CDCl_3$) δ -190.12.

HRMS (ESI) *m/z* calcd for $C_{27}H_{28}O_4ClFNa$ $[M+Na]^+$ 493.1552; **found**: 493.1555.

2.3.9 Synthesis of G11

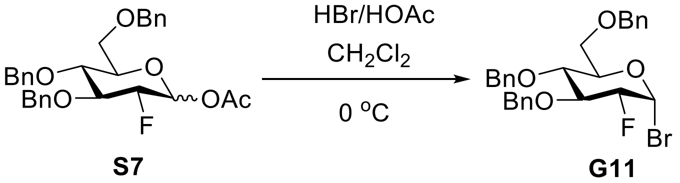

Figure S12

To a solution of **S7**[19] (1.50 g, 3.0 mmol) in dry CH_2Cl_2 (30 mL) was added 33% HBr/HOAc (7 mL), and the mixture was stirred at 0 °C for 5 h. After that, the resulting mixture was washed with saturated $NaHCO_3$ (50 mL), then dried over anhydrous Na_2SO_4 and filtered. The solvent was removed in vacuo and the residue was purified by silica gel column chromatography (petroleum ether/EtOAc = 8:1) to give **G11** as a colorless syrup (0.72 g, 46% yield). The characterization data are consistent with the literature[19].

^{1}H NMR (500 MHz, $CDCl_3$) δ 7.39–7.17 (m, 15H), 6.57 (d, *J* = 4.1 Hz, 1H), 4.92 (d, *J* = 11.0 Hz, 1H), 4.86 4.92 (d, *J* = 11.0 Hz, 1H), 4.79 4.92 (d, *J* = 11.0 Hz, 1H), 4.60

4.92 (d, J = 11.0 Hz, 1H), 4.54 4.92 (d, J = 11.0 Hz, 1H), 4.51 (ddd, J = 50.0 Hz, 9.0 Hz, 1H), 4.49 (d, J = 12.0 Hz, 1H), 4.19 (td, J = 11.5 Hz, 9.0 Hz, 1H), 4.09 (m, 1H), 3.82 (t, J = 9.0 Hz, 1H), 3.81 (dd, J = 3.5 Hz, 1H), 3.69 (dd, J = 11.0 Hz, 2.0 Hz, 1H). **^{13}C NMR** (126 MHz, $CDCl_3$) δ 138.27, 138.06, 137.86, 128.84-128.27, 90.46 (d, J = 188.1 Hz), 88.26 (d, J = 18.9 Hz), 81.30 (d, J = 15.5 Hz), 75.78, 75.62 (d, J = 8.8 Hz), 75.60, 75.46 (d, J = 1.2 Hz), 73.89, 67.68.

2.3.10 Synthesis of G12

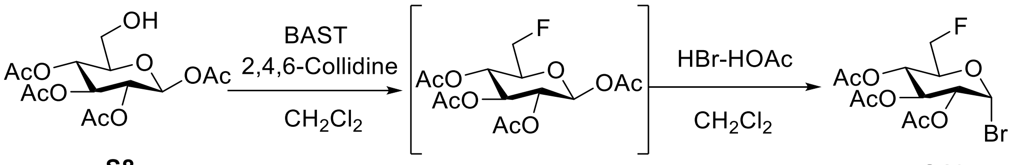

Figure S13

To a solution of commercially available **S8** (3.0 g, 9.0 mmol) in dry CH_2Cl_2 (60 mL) was added 2,4,6-collidine (2.1 mL, 18 mmol), and the mixture was stirred at -20 °C under Ar for 5 mins, bis(2-methoxyethyl)aminosulfur trifluoride [(BAST), 3.3 mL, 18 mmol] was added, and the mixture was stirred at RT for 24 h. After that, the resulting mixture was quenched by MeOH (15 mL) at 0 °C. The solvent was removed in vacuo and the residue was purified by silica gel column chromatography (petroleum ether/EtOAc = 4:1) to give crude **S9** as a white solid, and then directly used in the next step without further characterization.

To a solution of **S9** in dry CH_2Cl_2 (60 mL) was added 33% HBr/HOAc (15 mL), and the mixture was stirred at 0 °C for 5 h. After that, the resulting mixture was washed with saturated $NaHCO_3$ (100 mL), then dried over anhydrous Na_2SO_4 and filtered. The solvent was removed in vacuo and the residue was purified by silica gel column chromatography (petroleum ether/EtOAc = 6:1) to give **G12** as a colorless syrup (1.04 g, 33% yield for 2 steps). The characterization data are consistent with the literature[7].

^{1}H NMR (500 MHz, $CDCl_3$) δ 6.62 (d, J = 4.0 Hz, 1H), 5.56 (t, J = 9.7 Hz, 1H), 5.19 (t, J = 10.2 Hz, 1H), 4.82 (dd, J = 9.7 Hz, 4.0 Hz, 1H), 4.53 (ddd, J = 46.8 Hz, 10.8 Hz, 2.4 Hz, 1H), 4.47 (ddd, J = 46.8 Hz, 10.8 Hz, 3.3 Hz, 1H), 4.26 (ddt, J = 24.9 Hz,

10.4 Hz, 2.7 Hz, 1H), 2.09 (s, 3H), 2.06 (s, 3H), 2.03 (s, 3H).

2.3.11 Synthesis of G14

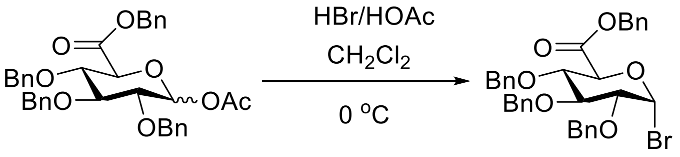

Figure S14

To a solution of **S10**[20] (100 mg, 0.17 mmol) in dry CH_2Cl_2 (0.5 mL) was added 33% HBr/HOAc (0.1 mL) at 0 °C, and the mixture was stirred at RT for 3 h. After that, CH_2Cl_2 (5 mL) was added, and the resulting mixture was washed with saturated $NaHCO_3$ (20 mL), then dried over anhydrous Na_2SO_4 and filtered. The solvent was removed in vacuo to give crude **G14** as a colorless syrup (> 95% yield), which was immediately used in the glycosylation reactions without further purification and characterization.

2.3.12 Synthesis of G17

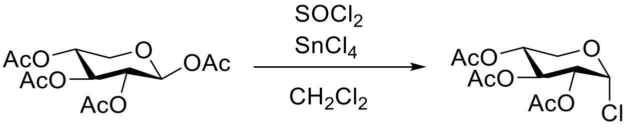

Figure S15

To a solution of commercially available **S11** (950 mg, 3.0 mmol) in dry CH_2Cl_2 (15 mL) was added $SOCl_2$ (0.45 mL, 6 mmol) and $SnCl_4$ (1M in CH_2Cl_2, 3.0 mL, 3.0 mmol), and the mixture was stirred at RT for 20 h. After that, the resulting mixture was washed with saturated $NaHCO_3$ (50 mL), then dried over anhydrous Na_2SO_4 and filtered. The solvent was removed in vacuo and the residue was purified by silica gel column chromatography (petroleum ether/EtOAc = 4:1) to give **G17** as a white solid (643 mg, 73%).

$[\alpha]_D^{25}$ = 152.0 (*c* 1.0, $CHCl_3$)

^{1}H NMR (500 MHz, $CDCl_3$) δ 6.20 (d, *J* = 3.9 Hz, 1H), 5.50 (t, *J* = 9.8 Hz, 1H), 4.97

(td, J = 10.3, 6.0 Hz, 1H), 4.90 (dd, J = 10.0, 4.0 Hz, 1H), 3.96 (dd, J = 11.4, 6.0 Hz, 1H), 3.86 (t, J = 11.1 Hz, 1H), 2.05 (s, 3H), 2.00 (s, 6H).

^{13}C NMR (126 MHz, $CDCl_3$) δ 169.93, 169.86, 169.81, 90.77 (d, J = 7.3 Hz), 70.97, 68.79, 68.29, 60.88, 20.68, 20.64, 20.60.

HRMS (ESI) *m/z* calcd for $C_{11}H_{15}O_7ClNa$ $[M+Na]^+$ 317.0399; **found**: 317.0398.

2.3.13 Synthesis of G18

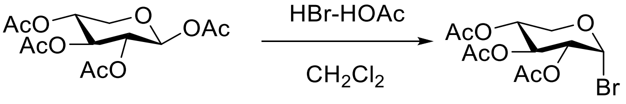

S11 G18

Figure S16

To a solution of commercially available **S11** (1.0 g, 3.14 mmol) in dry CH_2Cl_2 (20 mL) was added 33% HBr-HOAc (3.4 mL), and the mixture was stirred at 0 °C for 5 mins. After that, the resulting mixture was washed with saturated $NaHCO_3$ (100 mL), then dried over anhydrous Na_2SO_4 and filtered. The solvent was removed in vacuo to give crude **G18** as a colorless syrup (> 95% yield), which was immediately used in the glycosylation reactions without further purification. The characterization data are consistent with the literature[9].

^{1}H NMR (500 MHz, $CDCl_3$) δ 6.58 (d, J = 3.9 Hz, 1H), 5.56 (t, J = 9.9 Hz, 1H), 5.49 (td, J = 9.9, 6.0 Hz, 1H), 4.77 (dd, J = 9.9, 3.9 Hz, 1H), 4.05 (dd, J = 11.1, 6.0 Hz, 1H), 3.88 (t, J = 11.1 Hz, 1H), 2.10 (s, 3H), 2.06 (s, 3H), 2.05 (s, 3H).

2.3.14 Synthesis of G19

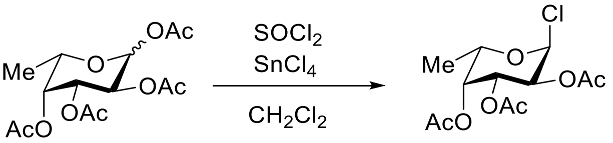

S12 G19

Figure S17

To a solution of commercially available **S12** (208 mg, 0.63 mmol) in dry CH_2Cl_2 (3 mL) was added $SOCl_2$ (0.10 mL, 1.2 mmol) and $SnCl_4$ (1M in CH_2Cl_2, 0.63 mL, 0.63

mmol), and the mixture was stirred at RT for 20 h. After that, the resulting mixture was washed with saturated $NaHCO_3$ (10 mL), then dried over anhydrous Na_2SO_4 and filtered. The solvent was removed in vacuo and the residue was purified by silica gel column chromatography (petroleum ether/EtOAc = 3:1) to give **G19** as a brown solid (142 mg, 74%).

$[\alpha]_D^{25}$ = -215.5 (*c* 1.0, $CHCl_3$)

^{1}H NMR (500 MHz, $CDCl_3$) δ 6.23 (d, *J* = 4.0 Hz, 1H), 5.28 (dd, *J* = 10.7, 3.3 Hz, 1H), 5.23 (dd, *J* = 3.4, 1.3 Hz, 1H), 5.10 (dd, *J* = 10.7, 3.9 Hz, 1H), 4.39–4.27 (m, 1H), 2.05 (s, 3H), 1.98 (s, 3H), 1.88 (s, 3H), 1.08 (d, *J* = 6.6 Hz, 3H).

^{13}C NMR (126 MHz, $CDCl_3$) δ 170.07, 169.92, 169.55, 91.80 (d, *J* = 3.4 Hz), 70.06, 67.80, 67.75, 67.35, 20.48, 20.40, 20.34, 15.43.

HRMS (ESI) *m/z* calcd for $C_{12}H_{17}O_7ClNa$ $[M+Na]^+$ 331.0555; **found**: 331.0555.

2.3.15 Synthesis of G20

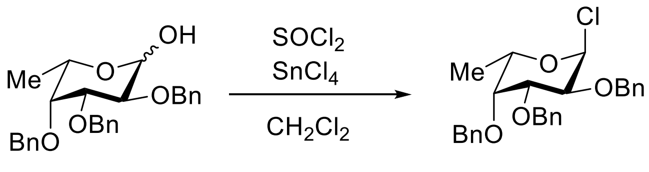

Figure S18

A solution of oxalyl choloride (0.55 mL, 6.44 mmol) in dichloromethane (1.0 mL) was added dropwise to a stirring solution of **S13**[21] (1.0 g, 2.3 mmol) in dichloromethane (8.0 mL) and DMF (53 μL), and the resulting mixture was stirred under argon for 30 min at 0 °C. The external cooling was removed, and the reaction mixture was allowed to slowly warm to rt and stirred for additional 2 h at rt. After that, the resulting mixture was concentrated in vacuo. The residue was dissolved in ethyl acetate (50 mL) and washed with saturated $NaHCO_3$ (50 mL), then dried over anhydrous Na_2SO_4 and filtered. The solvent was removed in vacuo and the residue was purified by silica gel column chromatography (petroleum ether/EtOAc = 8:1) to give **G20** as a colorless syrup (0.72 g, 69%). The characterization data are consistent

with the literature[22].

^{1}H NMR (500 MHz, $CDCl_3$) δ 7.72 – 7.27 (m, 15H), 6.14 (d, *J* = 3.7 Hz, 1H), 4.99 (d, *J* = 11.4 Hz, 1H), 4.89 (d, *J* = 11.8 Hz, 1H), 4.80 – 4.70 (m, 3H), 4.65 (d, *J* = 11.4 Hz, 1H), 4.21 (dd, *J* = 9.9, 3.9 Hz, 1H), 4.16 (t, *J* = 6.5 Hz, 1H), 3.98 (dd, *J* = 9.9, 2.8 Hz, 1H), 3.69 (d, *J* = 2.7 Hz, 1H), 1.16 (d, *J* = 6.5 Hz, 3H).

2.3.16 Synthesis of G21

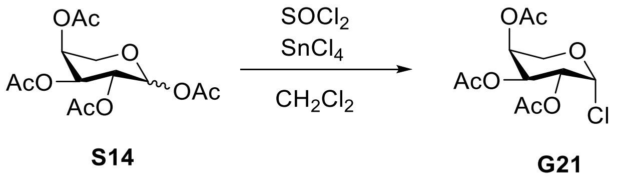

Figure S19

To a solution of **S14**[23] (800 mg, 2.5 mmol) in dry CH_2Cl_2 (10 mL) was added $SOCl_2$ (0.40 mL, 5 mmol) and $SnCl_4$ (1M in CH_2Cl_2, 2.5 mL, 2.5 mmol), and the mixture was stirred at RT for 20 h. After that, the resulting mixture was washed with saturated $NaHCO_3$ (50 mL), then dried over anhydrous Na_2SO_4 and filtered. The solvent was removed in vacuo and the residue was purified by silica gel column chromatography (petroleum ether/EtOAc = 3:1) to give **G21** as a white solid (601 mg, 81%).

$[\alpha]_D^{25}$ = 217.5 (*c* 0.25, $CHCl_3$)

^{1}H NMR (500 MHz, $CDCl_3$) δ 6.27 (d, *J* = 3.9 Hz, 1H), 5.37–5.23 (m, 2H), 5.18 (ddd, *J* = 12.1, 3.9, 1.8 Hz, 1H), 4.16 (d, *J* = 13.4 Hz, 1H), 3.80 (dd, *J* = 13.4, 1.8 Hz, 1H), 2.05 (s, 3H), 2.01 (s, 3H), 1.92 (s, 3H).

^{13}C NMR (126 MHz, $CDCl_3$) δ 169.91, 169.87, 169.60, 92.24 (d, *J* = 4.2 Hz), 67.99, 67.98, 67.92, 66.55, 62.98, 20.66, 20.50, 20.46.

HRMS (ESI) *m/z* calcd for $C_{11}H_{15}O_7ClNa$ $[M+Na]^+$ 317.0399; **found**: 317.0402.

2.3.17 Synthesis of G22

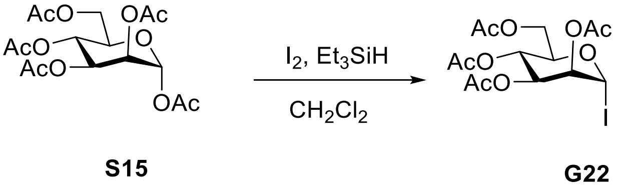

Figure S20

To a solution of commercially available **S15** (3.51 g, 9.0 mmol) in dry CH_2Cl_2 (45 mL) was added I_2 (6.87 g, 27 mmol) and Et_3SiH (2.16 mL, 13.5 mmol), and the mixture was stirred at 60 °C for 2 h. After that, the reaction was diluted with ethyl acetate (100 ml), and extracted with water (200 mL), 10% $Na_2S_2O_3$ (200 mL), and brine (200 mL). The organic layer was dried over Na_2SO_4. The solvent was removed in vacuo and the residue was purified by silica gel column chromatography (petroleum ether/EtOAc = 2:1) to give **G3** as a white solid (2.72 g, 66%). The spectroscopic data are consistent with the literature[2].

^{1}H NMR (500 MHz, $CDCl_3$) δ 6.70 (s, 1H), 5.79 (dd, *J* = 10.2, 3.4 Hz, 1H), 5.47 (dd, *J* = 3.4, 1.4 Hz, 1H), 5.38 (t, *J* = 10.2 Hz, 1H), 4.35 (dd, *J* = 12.6, 5.0 Hz, 1H), 4.14 (dd, *J* = 12.4, 2.1 Hz, 1H), 3.96 (ddd, *J* = 10.3, 5.0, 2.1 Hz, 1H), 2.17 (s, 3H), 2.10 (s, 3H), 2.08 (s, 3H), 2.01 (s, 3H).

2.3.18 Synthesis of G24

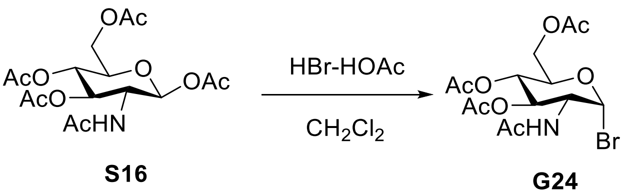

Figure S21

To a solution of commercially available **S16** (1.0 g, 2.6 mmol) in dry CH_2Cl_2 (10 mL) was added 33% HBr-HOAc (10 mL), and the mixture was stirred at 0 °C for 5 h. After that, the solvent was removed in vacuo to give crude **G18** as a colorless syrup (> 95% yield), which was immediately used in the glycosylation reactions without further purification and characterization.

2.3.19 Synthesis of G25

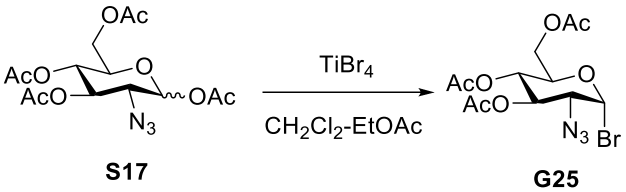

Figure S22

To a solution of **S17**[24] (1.2 g, 3.2 mmol) in a mixture of dry CH_2Cl_2 (2 mL) and EtOAc (2.5 mL) was added $TiBr_4$ (2.5g, 2.1mmol), and the mixture was stirred at RT for 3 days. At the end of the reaction, toluene (50 mL) and acetonitrile (16 mL) were added, and anhydrous sodium acetate (20 g) was added to the system with stirring to decolorize it, the mixture was filtered through celite, washed with methyl *tert*-butyl ether (50 mL), and the solvent was removed in vacuo, then dissolved in toluene (50 mL), evaporated to dryness, and repeated three times, to obtain the product **G25** as a colorless syrup (1.2 g, 96%), which was immediately used in the glycosylation reactions without further purification. The spectroscopic data are consistent with the literature[25].

^{1}H NMR (500 MHz, $CDCl_3$) δ 6.42 (d, *J* = 4.0 Hz, 1H), 5.50 (t, *J* = 9.6 Hz, 1H), 5.14 (t, *J* = 9.6 Hz, 1H), 4.35 (dd, *J* = 14.4Hz, 4.1 Hz, 1H), 4.30 - 4.33 (m, 1H), 4.12 (dd, *J* = 12.7 Hz, 2.1 Hz, 1H), 3.80 (dd, *J* = 10.0 Hz, 3.8Hz, 1H), 2.11 (s, 3H), 2.09 (s, 3H), 2.06 (s, 3H).

2.3.20 Synthesis of G26

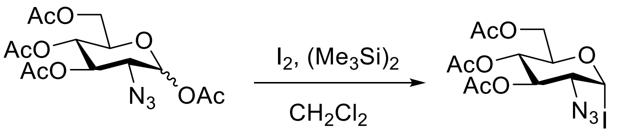

S17 G26

Figure S23

To a solution of **S17**[24] (0.70 g, 1.9 mmol) in dry CH_2Cl_2 (5 mL) was added I_2 (0.50 g, 2.1 mmol) and $(Me_3Si)_2$ (0.40 mL, 2.1 mmol), and the mixture was stirred at RT for 3 h. After that, the reaction mixture was washed with 10% $Na_2S_2O_3$ (20 mL), and brine (20 mL). The organic layer was dried over Na_2SO_4. The solvent was removed in vacuo and the residue was purified by silica gel column chromatography (petroleum ether/EtOAc = 8:1) to give **G26** as a white solid (0.50 g, 32%). The spectroscopic data are consistent with the literature[14].

^{1}H NMR (500 MHz, $CDCl_3$) δ 6.78 (d, *J* = 4.2 Hz, 1H),5.37 (t, *J* = 9.8 Hz, 1H), 5.16

(t, J = 9.8 Hz, 1H), 4.36 (dd, J = 12.8 Hz, 4.2 Hz, 1H), 4.13 – 4.06 (m, 2H), 3.31 (dd, J = 9.8 Hz, 4.2 Hz, 1H), 2.10 (s, 3H), 2.09 (s, 3H), 2.06 (s, 3H).

2.3.21 Synthesis of G27

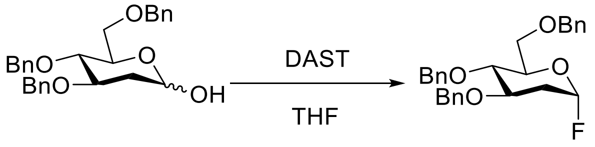

Figure S24

A solution of **S18**[26] (750 mg, 1.7 mmol) in dry THF (10 mL) was stirred at -30 °C under Ar for 5 mins, diethylaminosulfur trifluoride [(DAST), 0.3 mL, 2 mmol] was added, and the mixture was stirred at RT for 40 mins. After that, the resulting mixture was quenched by MeOH (5 mL) at 0 °C. The solvent was removed in vacuo and the residue was purified by silica gel column chromatography (petroleum ether/EtOAc = 5:1) to give **G27** (0.53 g, 71%). The characterization data are consistent with the literature[15].

^{1}H NMR (500 MHz, CD_2Cl_2) δ 7.44 – 7.22 (m, 15H), 5.75 (dt, J = 52.3, 2.2 Hz, 1H), 4.91 (d, J = 10.9 Hz, 1H), 4.70 – 4.63 (m, 2H), 4.61 (d, J = 10.9 Hz, 1H), 4.59 – 4.49 (m, 2H), 3.98 – 3.93 (m, 1H), 3.91 (ddd, J = 10.0, 4.0, 2.0 Hz, 1H), 3.79 (dd, J = 10.8, 3.9 Hz, 1H), 3.73 – 3.63 (m, 2H), 2.47 (dtd, J = 13.8, 4.9, 1.6 Hz, 1H), 1.74 (dddd, J = 40.0, 14.0, 11.4, 2.8 Hz, 1H)

2.3.22 Synthesis of G30

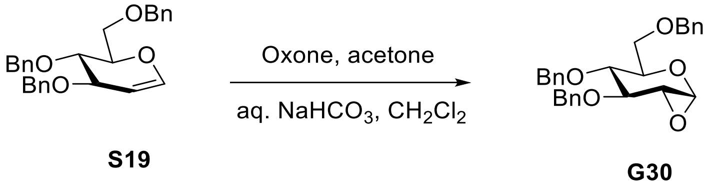

Figure S25

A solution of commercially available **S19** (4.0 g, 9.6 mmol) in CH_2Cl_2 (40 mL) was added acetone (4 mL) and saturated $NaHCO_3$ aqueous solution (67 mL), the mixture was stirred at 0 °C for 5 mins, a solution of Oxone (11.8 g, 19.2 mmol) in water (50

mL) was slowly added, and the mixture was stirred at 0 °C for 30 mins, then at RT for 2 h. After that, the resulting mixture was extracted by CH_2Cl_2 (100 mL × 3). The organic phases were combined, dried over anhydrous $NaSO_4$ and filtered. The solvent was removed in vacuo to give **G30** (>99% yield). The characterization data are consistent with the literature[16].

^{1}H NMR (500 MHz, $CDCl_3$): δ 7.38 - 7.21 (m, 13H), 7.21 - 7.14 (m, 2H), 4.99 (br, 1H), 4.80 (d, *J* = 11.0 Hz, 1H), 4.80 (d, *J* = 11.4 Hz, 1H), 4.69 (d, *J* = 11.4 Hz, 1H), 4.62 (d, *J* = 11.9 Hz, 1H), 4.58 (d, *J* = 11.0 Hz, 1H), 4.62 (d, *J* = 11.9 Hz, 1H), 3.98 (d, *J* = 7.8 Hz, 1H), 3.81 - 3.61 (m, 4H), 3.07 (d, *J* = 1.8 Hz, 1H);

2.3.23 Synthesis of G1-6-*d*3

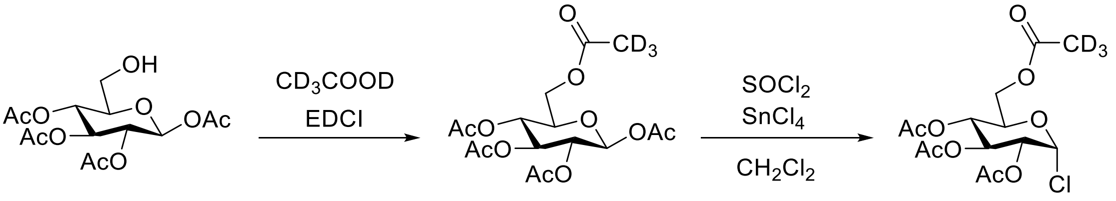

Figure S26

To a mixture of commercially available **S8** (5.0 g, 14 mmol), EDCI (8.1 g, 42 mmol) and DMAP (5.1 g, 42 mmol) were added CH_2Cl_2 (50 mL) and stirred at RT for 5 mins, CD_3COOD (1.8 g, 28 mmol) was added, and the solution was stirred at RT for 3 h. After that, the resulting mixture was washed with saturated $NaHCO_3$ (50 mL), then dried over anhydrous Na_2SO_4 and filtered. The solvent was removed in vacuo and the residue was purified by silica gel column chromatography (petroleum ether/EtOAc = 2.5:1) to give **S20** as a colorless syrup (2.5 g, 45%).

^{1}H NMR (500 MHz, $CDCl_3$) δ 5.61 (dd, *J* = 8.3, 1.8 Hz, 1H), 5.14 (td, *J* = 9.5, 2.1 Hz, 1H), 5.00 (ddd, *J* = 10.4, 6.9, 3.9 Hz, 2H), 4.17 (ddd, *J* = 12.2, 4.6, 2.6 Hz, 1H), 3.98 (dt, *J* = 12.6, 2.5 Hz, 1H), 3.76 (ddd, *J* = 10.1, 4.7, 2.2 Hz, 1H), 2.00-1.96 (m, 3H), 1.92-1.89 (m, 6H), 1.88-1.86 (m, 3H).

^{13}C NMR (126 MHz, $CDCl_3$) δ 170.39, 169.87, 169.86, 169.22, 169.04, 168.75, 91.50, 72.57, 72.49, 70.09, 67.61, 61.28, 20.61, 20.38.

To a solution of **S20** (2.0 g, 5.0 mmol) in dry CH_2Cl_2 (10 mL) was added $SOCl_2$ (0.80 mL, 10 mmol) and $SnCl_4$ (1M in CH_2Cl_2, 5.0 mL, 5.0 mmol), and the mixture was stirred at RT for 21 h. After that, the resulting mixture was washed with saturated $NaHCO_3$ (50 mL), then dried over anhydrous Na_2SO_4 and filtered. The solvent was removed in vacuo and the residue was purified by silica gel column chromatography (petroleum ether/EtOAc = 2:1) to give **G1**-6-*d*3 as a white solid (1.6 g, 89%).

^{1}H NMR (500 MHz, $CDCl_3$) δ 6.28 (d, *J* = 4.0 Hz, 1H), 5.55 (t, *J* = 9.8 Hz, 1H), 5.13 (dd, *J* = 11.0, 8.5 Hz, 1H), 5.00 (dd, *J* = 10.0, 4.0 Hz, 1H), 4.45 – 4.27 (m, 2H), 4.17 – 4.07 (m, 1H), 2.09 (s, 4H), 2.04 (s, 3H), 2.03 (s, 3H).

^{13}C NMR (126 MHz, $CDCl_3$) δ 170.72, 170.04, 170.02, 169.62, 90.19, 70.84, 70.49, 69.52, 67.51, 61.19, 20.81, 20.75, 20.72, 20.68.

2.3.24 Synthesis of G1-1-*d*

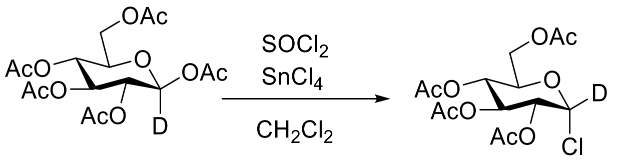

Figure S27

To a solution of **S21**[27] (131 mg, 0.34 mmol) in dry CH_2Cl_2 (5 mL) was added $SOCl_2$ (50 μL, 0.68 mmol) and $SnCl_4$ (40 μL, 0.34 mmol), and the mixture was stirred at RT overnight. After that, the resulting mixture was washed with saturated $NaHCO_3$ (50 mL), then dried over anhydrous Na_2SO_4 and filtered. The solvent was removed in vacuo and the residue was purified by silica gel column chromatography (petroleum ether/EtOAc = 4:1) to give **G1**-1-*d* as a white solid (95 mg, 76%).

^{1}H NMR (500 MHz, $CDCl_3$) δ 5.54 (dd, *J* = 10.1, 9.4 Hz, 1H), 5.12 (td, *J* = 9.7, 1.8 Hz, 1H), 4.99 (d, *J* = 10.1 Hz, 1H), 4.34 – 4.27 (m, 2H), 4.15 – 4.09 (m, 1H), 2.09 (s, 3H), 2.09 (s, 3H), 2.04 (s, 3H), 2.02 (s, 3H).

^{13}C NMR (126 MHz, $CDCl_3$) δ 170.65, 170.00, 169.60, 89.90, 70.79, 70.49, 69.54, 67.55, 61.24, 20.78, 20.73, 20.69, 20.66.

3. Reaction optimization for the stereoselective *N*-glycoconjugation.

3.1 Glycosyl electrophile screening

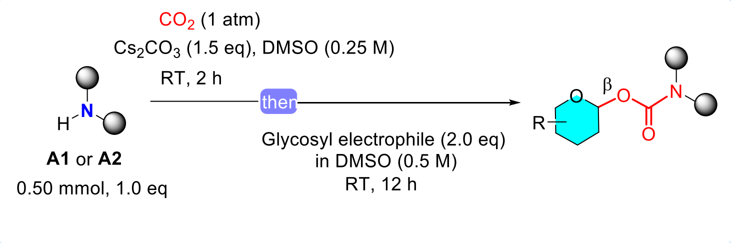

Amine

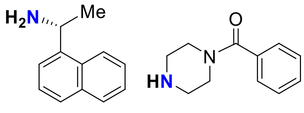

A1 **A2**

Glycosyl electrophiles

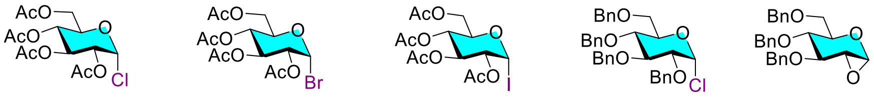

G1 **G2** **G3** **G4** **G30**

Entry	Amine	Glycosyl Electrophile	Product	Yield (%)
**1**	**A1**	**G1**	**GA1**	0
**2**	**A1**	**G2**	**GA1**	22
**3**	**A1**	**G3**	**GA1**	40
**4**	**A1**	**G4**	**GA2**	43
**5**	**A2**	**G1**	**GA12**	96
**6**	**A2**	**G2**	**GA12**	99
**7**	**A2**	**G3**	**GA12**	66
**8**[a]	**A2**	**G4**	**GA13**	92
**9**	**A2**	**G30**	**GA34**	0

[a]3.0 eq **G4** was used

Table S1

3.2 Solvent screening

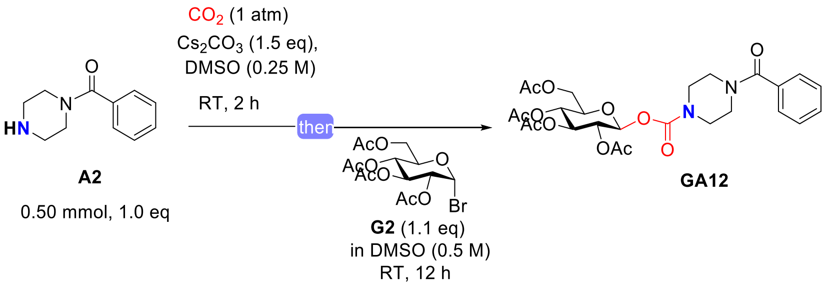

Entry	Solvent	Yield (%)
1	DMSO	86
2	MeCN	63
3	Acetone	30
4	THF	0
5	H_2O	0

Table S2

3.3 Base evaluation

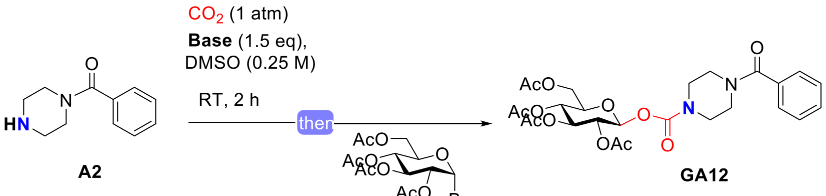

Base	Yield
K_2CO_3	67%
Cs_2CO_3	86%

Fig. S28

3.4 Stoichiometry optimization of Cs_2CO_3

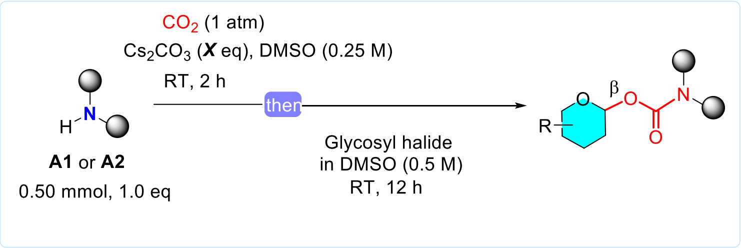

Amine

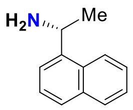

A1

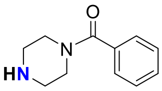

A2

Glycosyl halides

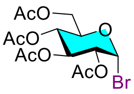

G2

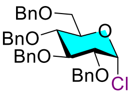

G4

Entry	Amine	Glycosyl Electrophile (equiv.)	Equiv. Cs_2CO_3	Product	Yield (%)
**1**	**A1**	**G4** (3.0)	2.0	**GA2**	75
**2**	**A1**	**G4** (3.0)	2.5	**GA2**	65
**3**	**A2**	**G2** (1.1)	1.0	**GA12**	72
**4**	**A2**	**G2** (1.1)	2.0	**GA12**	86

Table S3

3.5 Stoichiometry optimization of glycosyl halides

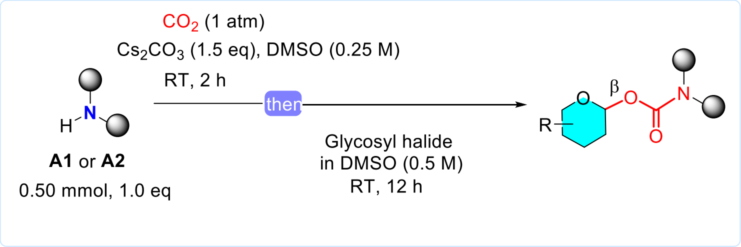

Amine **Glycosyl halides**

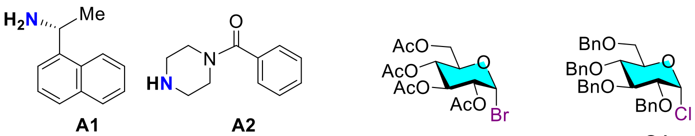

A1 **A2** **G2** **G4**

Entry	Amine	Glycosyl Electrophile (equiv.)	Product	Yield (%)
**1**	**A1**	**G4** (2.0)	**GA2**	43
**2**	**A1**	**G4** (3.0)	**GA2**	75
**3**	**A2**	**G2** (1.1)	**GA12**	86
**4**	**A2**	**G2** (2.0)	**GA12**	99

Table S4

4. A summary schematic diagram of the scope of glycosyl halides

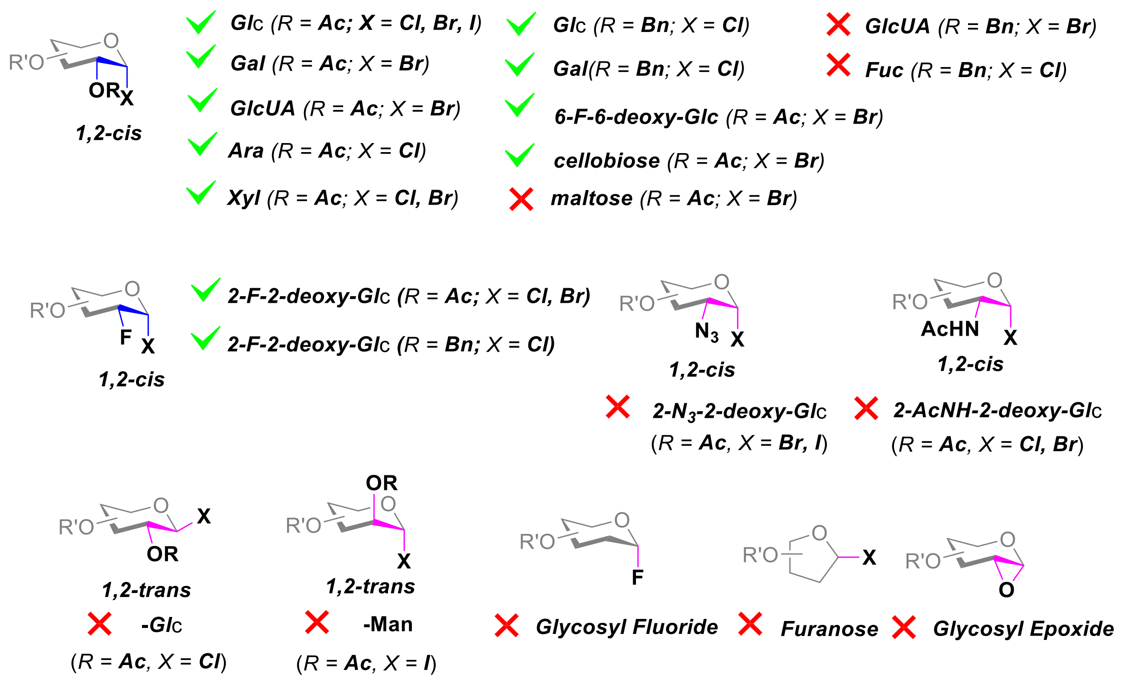

Fig. S29

5. Additional examples

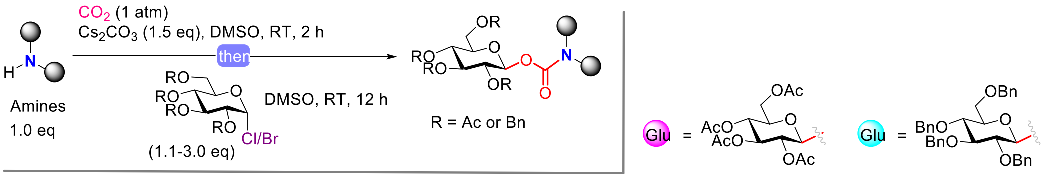

Primary amine glycoconjugates (all from glycosyl chlorides)

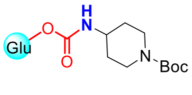

GA96, 68%

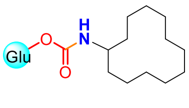

GA97, 39%

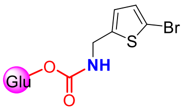

GA98, 29%

Secondary amine glycoconjugates (all from glycosyl bromides)

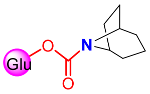

GA99, 56%

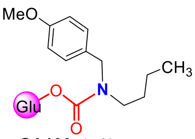

GA100, 95%

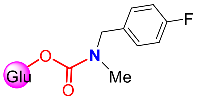

GA101, 74%

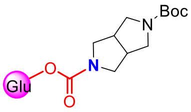

GA102, 76%

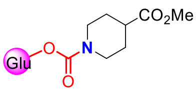

GA103, 60%

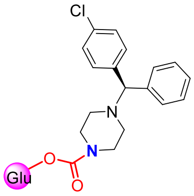

GA104, 78%

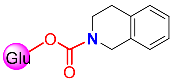

GA105, 62%

Drug glycocojugates (all from glycosyl bromides except **GA106**)

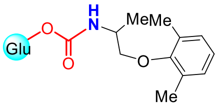

GA106, 66%
[from mexiletine, antiarrhythmic]

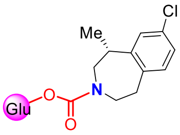

GA107, 97%
[from iorcaserin, anti-obesity]

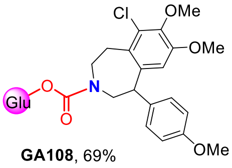

GA108, 69%
[from fenoldopam derivative, antihypertensive]

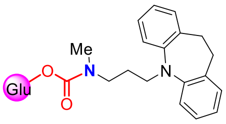

GA109, 70%
[from desipramine, antidepressant]

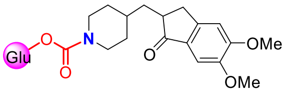

GA110, 68%
[from donepezil fragment, AChE inhibitor]

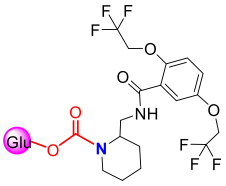

GA111, 72%
[from flecainide, antiarrhythmic]

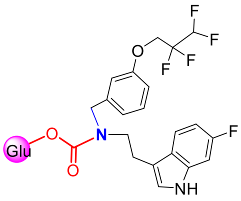

GA112, 58%
[from idalopirdine, 5HT6 antagonist]

Figure S30

6. Unsuccessful examples

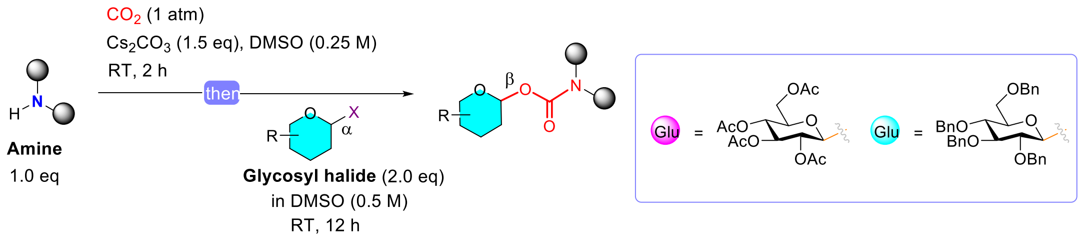

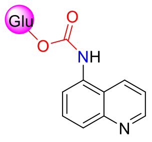

GA113, 0 % (X = Br)

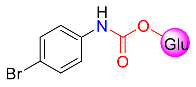

GA114, 0 % (X = Br)

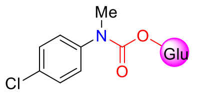

GA115, 0 % (X = Br)

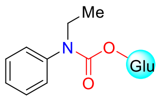

GA116, 0 % (X = Cl)

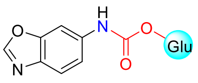

GA117, 0 % (X = Cl)

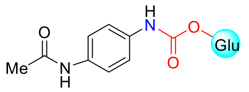

GA118, 0 % (X = Cl)

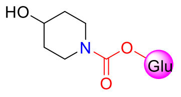

GA119, Complex mixture (X = Br)

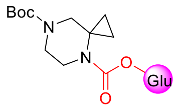

GA120, 0 % (X = Br)

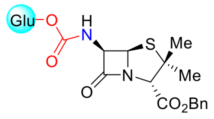

GA121, 0 % (X = Cl)

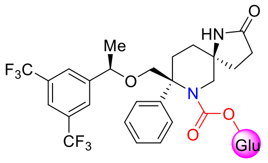

GA122, 0 % (X = Br)

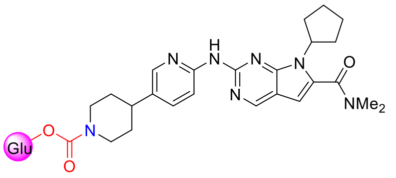

GA123, 0 % (X = Br)

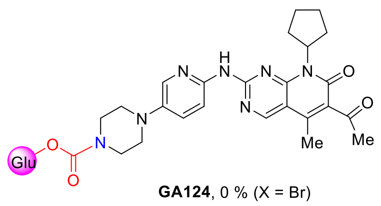

GA124, 0 % (X = Br)

Figure S31

7. Identification of a common side reaction

During the substrate scope study, a common side product was identified, in that when using the combination of primary amine and glycosyl halides bearing acetyl protecting groups, acetyl transfer from the sugar moiety to the amino group was frequently observed. This side reaction could explain the relatively lower yields of *N*-glycoconjugation reaction using this substrate combination. A typical example was provided below.

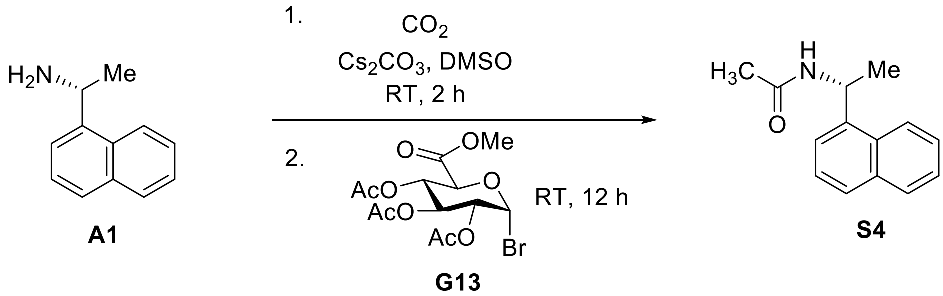

Figure S32

An oven-dried Schlenk tube (25 ml) was charged with **A1** (171 mg, 1.00 mmol), Cs_2CO_3 (490 mg, 1.5 mmol), and a Teflon-coated magnetic stirring bar. After the mixture was evacuated and backfilled with CO_2 gas three times, DMSO (2.0 mL) was added via a syringe. The reaction mixture was allowed to stir for 2 h under a CO_2 atmosphere at RT, and then a solution of **G13** (794 mg, 2.0 mmol) in DMSO (2 mL) was added, and the reaction was further stirred at RT for 12 h. H_2O (10 mL) was added, and the mixture was extracted with CH_2Cl_2 (20 mL×3), washed with saturated NH_4Cl (50 mL), then dried over anhydrous Na_2SO_4 and filtered. The solvent was removed in vacuo, and the residue was purified by silica gel column chromatography (petroleum ether/EtOAc = 1:1) to give **S4**[28] as a white solid (177.2 mg, 83%).

^{1}H NMR (500 MHz, $CDCl_3$) δ 8.08 (d, *J* = 8.4 Hz, 1H), 7.89–7.84 (m, 1H), 7.79 (d, *J* = 8.1 Hz, 1H), 7.61–7.47 (m, 3H), 7.44 (t, *J* = 7.6 Hz, 1H), 5.90 (q, *J* = 7.0 Hz, 1H), 5.85 (s, 1H), 1.94 (s, 3H), 1.65 (d, *J* = 6.6 Hz, 3H).

^{13}C NMR (126 MHz, $CDCl_3$) δ 169.16, 138.29, 134.03, 131.22, 128.89, 128.51, 126.72, 126.01, 125.29, 123.53, 122.67, 44.75, 23.43, 20.74.

8. General procedure for the stereoselective *N*-glycoconjugation

An oven-dried Schlenk tube (25 mL) were charged with amine (0.50 mmol, 1.00 eq), Cs_2CO_3 (0.75 mmol, 1.5 eq), and a Teflon-coated magnetic stirring bar. After the mixture was evacuated and backfilled with CO_2 gas three times, DMSO (2.0 mL, 0.25 M) was added via a syringe. The reaction mixture was allowed to stir for 2 h under a CO_2 atmosphere at RT, and then a solution of glycosyl halide (1.00 mmol, 2.00 eq) in DMSO (2 mL, 0.50 M) was added, and the reaction was further stirred at RT for 12 h. H_2O (10 mL) was added, and the mixture was extracted with CH_2Cl_2 (20 mL×3), washed with saturated NH_4Cl (50 mL), then dried over anhydrous Na_2SO_4 and filtered. The solvent was removed in vacuo, and the residue was purified by silica gel column chromatography to afford the desired product.

Note: Occasionally, gelation occurred when using Cs_2CO_3 as the base, leading to diminished yields. To circumvent this problem, K_2CO_3 was used instead.

Reaction Setup

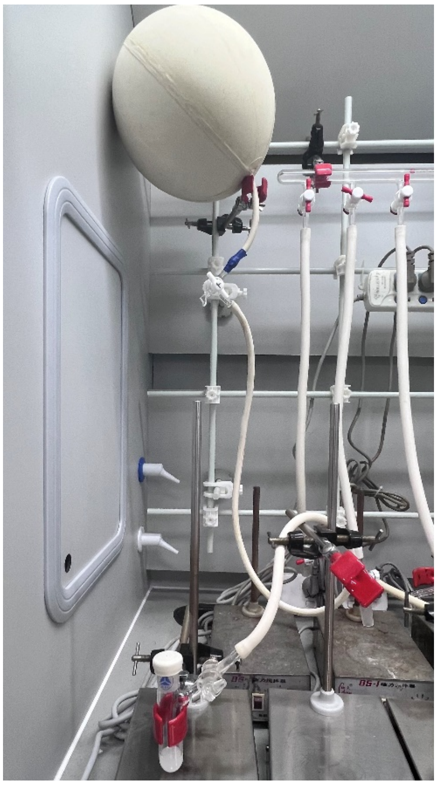

Figure S33

Compound GA1

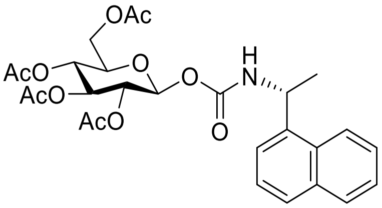

Compound **GA1** was synthesized according to the *General procedure* using amine **A1** (171 mg, 1.0 mmol, 1.0 eq) and glycosyl halide **G3** (0.916 g, 2.0 mmol, 2.0 eq) in the presence of Cs_2CO_3 (490 mg, 1.5 mmol, 1.5 eq). After the reaction, the mixture was purified by silica gel flash chromatography (petroleum ether/EtOAc = 4:1) to give **GA1** as a yellow liquid (218 mg, 40%).

$[\alpha]_D^{25}$ = -8.3 (*c* 1.0, $CHCl_3$).

^{1}H NMR (500 MHz, $CDCl_3$) δ 8.07 (d, *J* = 8.3 Hz, 1H), 7.91–7.84 (m, 1H), 7.79 (q, *J* = 7.0 Hz, 1H), 7.62–7.41 (m, 5H), 5.69 (d, *J* = 8.3 Hz, 1H), 5.67–5.60 (m, 1H), 5.35 (d, *J* = 7.8 Hz, 1H), 5.25 (t, *J* = 9.5 Hz, 1H), 5.15–5.04 (m, 2H), 4.29 (dd, *J* = 12.4, 4.3 Hz, 1H), 4.05 (dd, *J* = 12.4, 2.2 Hz, 1H), 3.80 (ddd, *J* = 10.1, 4.4, 2.2 Hz, 1H), 2.07 (s, 3H), 2.03–1.98 (m, 9H), 1.65 (d, *J* = 6.8 Hz, 3H).

^{13}C NMR (126 MHz, $CDCl_3$) δ 170.95, 170.22, 169.67, 169.56, 152.97, 137.99, 134.05, 130.76, 129.05, 128.58, 126.67, 125.97, 125.42, 123.04, 122.48, 92.96, 77.41, 77.16, 76.91, 72.93, 72.50, 70.41, 67.94, 61.53, 47.06, 21.76, 20.81, 20.70.

HRMS (ESI) m/z calcd for $C_{27}H_{31}NO_{11}Na$ $[M+Na]^+$ 568.1789; **found**: 568.1797.

Compound GA2

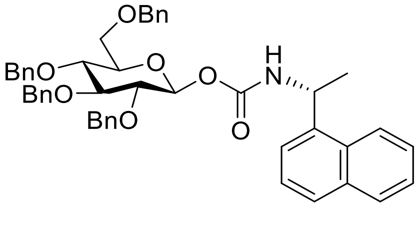

Compound **GA2** was synthesized according to the *General procedure* using amine **A1** (171 mg, 1.00 mmol, 1.0 eq) and glycosyl halide **G4** (1680 mg, 1.50 mmol, 3.0 eq) in the presence of Cs_2CO_3 (490 mg, 1.50 mmol, 1.5 eq). After the reaction, the mixture

was purified by silica gel flash chromatography (petroleum ether/EtOAc = 6:1) to give **GA2** as a white solid (554 mg, 75%).

$[\alpha]_D^{25}$ = -68.9 (*c* 1.0, $CHCl_3$).

^{1}H NMR (500 MHz, $CDCl_3$) δ 8.16–8.08 (m, 1H), 7.91–7.84 (m, 1H), 7.79 (d, *J* = 8.0 Hz, 1H), 7.56–7.42 (m, 4H), 7.39–7.22 (m, 18H), 7.17–7.11 (m, 2H), 5.68 (dt, *J* = 7.1 Hz, 1H), 5.59 (d, *J* = 8.2 Hz, 1H), 5.02 (d, *J* = 8.0 Hz, 1H), 4.91 (d, *J* = 10.9 Hz, 1H), 4.86–4.74 (m, 4H), 4.60 (d, *J* = 12.1 Hz, 1H), 4.53 (d, *J* = 10.8 Hz, 1H), 4.45 (d, *J* = 12.1 Hz, 1H), 3.77–3.68 (m, 4H), 3.58–3.51 (m, 2H), 1.66 (d, *J* = 6.8 Hz, 3H).

^{13}C NMR (126 MHz, $CDCl_3$) δ 153.58, 138.54, 138.45, 138.25, 138.18, 138.10, 134.06, 130.89, 128.97, 128.56, 128.53, 128.45, 128.21, 128.07, 128.05, 128.02, 127.89, 127.85, 127.73, 126.68, 125.94, 125.35, 123.31, 122.49, 95.31, 84.96, 81.37, 75.91, 75.42, 75.17, 75.13, 73.62, 68.13, 46.89, 21.76.

HRMS (ESI) m/z calcd for $C_{47}H_{47}NO_7Na$ $[M+Na]^+$ 760.3245; **found**: 760.3248.

Compound GA4

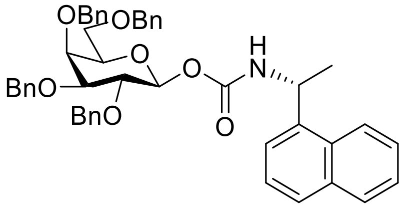

Compound **GA4** was synthesized according to the *General procedure* using amine **A1** (171 mg, 1.00 mmol, 1.0 eq) and glycosyl halide **G7** (1680 mg, 1.50 mmol, 3.0 eq) in the presence of Cs_2CO_3 (490 mg, 1.50 mmol, 1.5 eq). After the reaction, the mixture was purified by silica gel flash chromatography (petroleum ether/EtOAc = 4:1) to give **GA4** as a white solid (317 mg, 43%).

$[\alpha]_D^{25}$ = -13.6 (*c* 0.1, CH_2Cl_2).

^{1}H NMR (600 MHz, $CDCl_3$) δ 8.09–8.05 (m, 1H), 7.87–7.83 (m, 1H), 7.76 (d, *J* = 8.0 Hz, 1H), 7.50–7.39 (m, 4H), 7.38–7.20 (m, 20H), 5.67–5.61 (m, 1H), 5.55 (d, *J* = 8.1 Hz, 1H), 5.01 (d, *J* = 8.0 Hz, 1H), 4.91 (d, *J* = 11.6 Hz, 1H), 4.83 (d, *J* = 11.3 Hz, 1H), 4.75 (d, *J* = 11.3 Hz, 1H), 4.72 (s, 2H), 4.60 (d, *J* = 11.6 Hz, 1H), 4.41 (d, *J* = 11.7 Hz,

1H), 4.37 (d, *J* = 11.7 Hz, 1H), 3.96 (d, *J* = 2.9 Hz, 1H), 3.89 (t, *J* = 8.9 Hz, 1H), 3.70–3.66 (m, 1H), 3.64–3.52 (m, 3H), 1.64 (d, *J* = 6.8 Hz, 3H).

^{13}C NMR (151 MHz, $CDCl_3$) δ 153.69, 138.75, 138.66, 138.40, 138.30, 137.93, 134.05, 130.90, 128.92, 128.55, 128.46, 128.44, 128.37, 128.32, 128.18, 128.08, 127.92, 127.81, 127.75, 127.71, 126.62, 125.90, 125.32, 123.34, 122.44, 95.60, 82.54, 78.52, 75.40, 74.79, 73.94, 73.61, 73.26, 73.04, 68.04, 46.87, 29.85, 21.77.

HRMS (ESI) m/z calcd for $C_{47}H_{47}NO_7Na$ $[M+Na]^+$ 760.3245; **found**: 760.3252.

Compound GA5

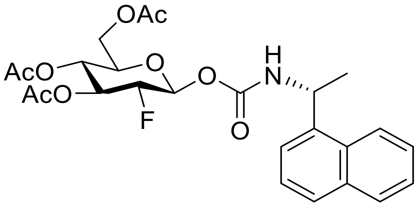

Compound **GA5** was synthesized according to the *General procedure* using amine **A1** (68 mg, 0.40 mmol, 1.0 eq) and glycosyl halide **G8** (260 mg, 0.80 mmol, 2.0 eq) in the presence of Cs_2CO_3 (196 mg, 0.60 mmol, 1.5 eq). After the reaction, the mixture was purified by silica gel flash chromatography (petroleum ether/EtOAc = 2.5:1) to give a mixture of **GA5** and *N*-acetyl-(1-naphthyl)ethylamine. To remove the side product, the mixture was dissolved with methanol (2 mL), and K_2CO_3 (10.0 mg) was added. After 30 min, the mixture was purified by silica gel flash chromatography (petroleum ether/EtOAc = 1:1 to methanol/EtOAc = 1:4). The product was dissolved with pyridine (1 mL), and Ac_2O (0.1 mL) was added. Overnight, the mixture was purified by silica gel flash chromatography (petroleum ether/EtOAc = 1:1) to give **GA5** as a yellow solid (91 mg, 45%).

$[\alpha]_D^{25}$ = 35.5 (*c* 1.0, $CHCl_3$).

^{1}H NMR (500 MHz, $CDCl_3$) δ 8.10 (d, *J* = 8.5 Hz, 1H), 7.89–7.84 (m, 1H), 7.79 (d, *J* = 7.8 Hz, 1H), 7.55 (ddd, *J* = 8.4, 6.8, 1.5 Hz, 1H), 7.53–7.46 (m, 2H), 7.45 (t, *J* = 7.6 Hz, 1H), 5.77 (dd, *J* = 8.1, 2.9 Hz, 1H), 5.67 (dt, *J* = 7.0 Hz, 1H), 5.44–5.32 (m, 2H), 5.04 (t, *J* = 9.8 Hz, 1H), 4.48–4.32 (m, 1H), 4.29 (dd, *J* = 12.5, 4.4 Hz, 1H), 4.11 (q, *J* = 7.1 Hz, 1H), 4.05 (dd, *J* = 12.5, 2.2 Hz, 1H), 3.82 (ddd, *J* = 10.1, 4.3, 2.2 Hz, 1H),

2.08 (s, 3H), 2.02 (s, 3H), 2.01 (s, 3H), 1.68 (d, *J* = 6.8 Hz, 3H).

^{13}C NMR (126 MHz, $CDCl_3$) δ 170.68, 170.01, 169.67, 152.80, 138.06, 137.81, 130.79, 129.04, 128.58, 126.69, 125.98, 125.40, 123.10, 122.52, 92.48 (d, *J* = 24.3 Hz), 88.36 (d, *J* = 192.1 Hz), 77.41, 77.16, 76.91, 72.91 (d, *J* = 19.2 Hz), 72.50, 67.71, 61.37, 47.18, 21.70, 20.77, 20.66, 14.31.

HRMS (ESI) m/z calcd for $C_{25}H_{28}NO_9FNa$ $[M+Na]^+$ 528.1640; **found**: 528.1647.

Compound GA6

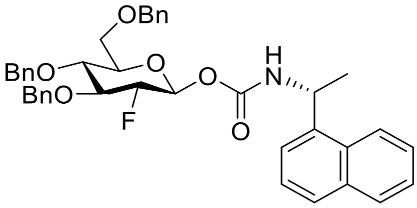

Compound **GA6** was synthesized according to the *General procedure* using amine **A1** (34 mg, 0.20 mmol, 1.0 eq) and glycosyl halide **G10** (282 mg, 0.60 mmol, 3.0 eq) in the presence of Cs_2CO_3 (98 mg, 0.30 mmol, 1.50 eq). After the reaction, the mixture was purified by silica gel flash chromatography (petroleum ether/EtOAc = 7:1) to give **GA6** as a yellow solid (64 mg, 43%).

$[\alpha]_D^{25}$ = 22.0 (*c* 1.0, $CHCl_3$).

^{1}H NMR (500 MHz, $CDCl_3$) δ 8.13 (d, *J* = 8.3 Hz, 1H), 7.88 (d, *J* = 7.9 Hz, 1H), 7.80 (d, *J* = 8.1 Hz, 1H), 7.58–7.43 (m, 4H), 7.41–7.24 (m, 13H), 7.19–7.12 (m, 2H), 5.75–5.65 (m, 2H), 5.29 (d, *J* = 7.9 Hz, 1H), 4.82 (d, *J* = 10.8 Hz, 1H), 4.78 (d, *J* = 11.1 Hz, 1H), 4.59 (d, *J* = 12.1 Hz, 1H), 4.52 (d, *J* = 11.0 Hz, 1H), 4.50–4.35 (m, 2H), 3.84 (dt, *J* = 14.6, 8.8 Hz, 1H), 3.75 (t, *J* = 9.3 Hz, 1H), 3.71 (d, *J* = 2.6 Hz, 2H), 3.57 (dd, *J* = 9.8, 2.6 Hz, 1H), 1.69 (d, *J* = 6.8 Hz, 1H).

^{13}C NMR (126 MHz, $CDCl_3$) δ 153.38, 138.31, 138.05, 138.01, 137.95, 134.07, 130.83, 129.01, 128.56, 128.52, 128.50, 128.47, 128.18, 128.03, 127.95, 127.81, 126.65, 125.93, 125.42, 123.21, 122.50, 92.82 (d, J = 22.0 Hz), 92.22 (d, J = 187.5 Hz), 83.25 (d, J = 14.9 Hz), 76.57 (d, J = 7.0 Hz), 75.56, 75.20 (d, J = 17.1 Hz), 73.64, 67.87, 47.10, 21.82.

HRMS (ESI) m/z calcd for $C_{40}H_{40}NO_6FNa$ $[M+Na]^+$ 672.2732; **found**: 672.2739.

Compound GA8

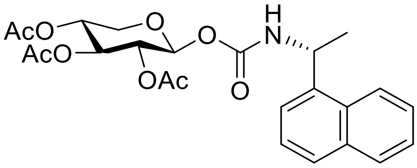

Compound **GA8** was synthesized according to the *General procedure* using amine **A1** (34 mg, 0.20 mmol, 1.0 eq) and glycosyl halide **G17** (118 mg, 0.40 mmol, 2.0 eq) in the presence of Cs_2CO_3 (98.0 mg, 0.30 mmol, 1.5 eq). After the reaction, the mixture was purified by silica gel flash chromatography (petroleum ether/EtOAc = 3:1) to give **GA8** as a white solid (34 mg, 36%).

$[\alpha]_D^{25}$ = -14.7 (*c* 0.5, $CHCl_3$).

^{1}H NMR (500 MHz, $CDCl_3$) δ 8.07 (d, *J* = 8.6 Hz, 1H), 7.85 (d, *J* = 8.0 Hz, 1H), 7.77 (d, *J* = 8.1 Hz, 1H), 7.60–7.37 (m, 4H), 5.65 (d, *J* = 7.5 Hz, 1H), 5.44 (d, *J* = 7.9 Hz, 1H), 5.23 (t, *J* = 8.9 Hz, 1H), 5.03 (dd, *J* = 9.1, 7.6 Hz, 1H), 4.95 (td, *J* = 9.1, 5.4 Hz, 1H), 4.06 (dd, *J* = 11.9, 5.3 Hz, 1H), 3.43 (dd, *J* = 12.0, 9.4 Hz, 1H), 2.08 (s, 3H), 2.05–1.99 (m, 9H), 1.64 (d, *J* = 6.8 Hz, 3H).

^{13}C NMR (126 MHz, $CDCl_3$) δ 170.03, 169.95, 169.72, 153.17, 138.08, 134.02, 130.78, 128.99, 128.50, 126.65, 125.92, 125.40, 123.02, 122.41, 93.45, 71.82, 70.10, 68.71, 62.96, 46.99, 21.65, 20.81, 20.79, 20.76.

HRMS (ESI) m/z calcd for $C_{24}H_{27}NO_9Na$ $[M+Na]^+$ 496.1578; **found**: 496.1584.

Compound GA10

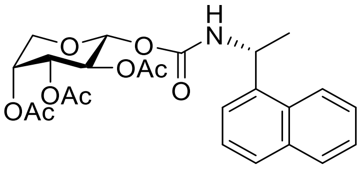

Compound **GA10** was synthesized according to the *General procedure* using amine **A1** (34 mg, 0.20 mmol, 1.0 eq) and glycosyl halide **G21** (118 mg, 0.40 mmol, 2.0 eq) in the presence of Cs_2CO_3 (139 mg, 0.30 mmol, 1.5 eq). After the reaction, the mixture was purified by silica gel flash chromatography (petroleum ether/EtOAc = 2.4:1) to give **GA10** as a white solid (48 mg, 51%).

$[\alpha]_D^{25}$ = 26.6 (*c* 1.0, $CHCl_3$).

^{1}H NMR (500 MHz, $CDCl_3$) δ 8.07 (d, *J* = 8.5 Hz, 1H), 7.85 (d, *J* = 8.0 Hz, 1H), 7.77 (d, *J* = 8.0 Hz, 1H), 7.53 (ddd, *J* = 8.5, 6.8, 1.5 Hz, 1H), 7.51–7.46 (m, 2H), 7.43 (t, *J* = 7.6 Hz, 1H), 5.65 (p, *J* = 7.1 Hz, 1H), 5.60 (d, *J* = 7.7 Hz, 1H), 5.41 (d, *J* = 7.7 Hz, 2H), 5.31–5.25 (m, 2H), 5.09 (dd, *J* = 9.8, 3.5 Hz, 1H), 3.97 (dd, *J* = 13.4, 2.8 Hz, 1H), 3.73 (dd, *J* = 13.3, 1.6 Hz, 1H), 2.10 (s, 3H), 2.09 (s, 3H), 2.01 (s, 3H), 1.64 (d, *J* = 6.8 Hz, 3H).

^{13}C NMR (126 MHz, $CDCl_3$) δ 170.30, 170.04, 169.81, 153.20, 138.04, 134.00, 130.77, 128.96, 128.48, 126.64, 125.90, 125.35, 123.04, 122.40, 93.59, 70.42, 68.29, 67.64, 64.40, 46.94, 21.61, 20.94, 20.88, 20.71.

HRMS (ESI) m/z calcd for $C_{24}H_{27}NO_9Na$ $[M+Na]^+$ 496.1578; **found**: 496.1584.

Compound GA12

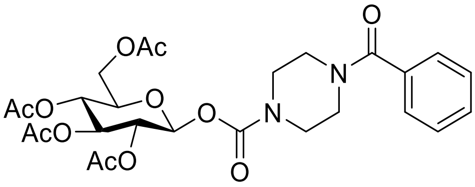

Compound **GA8** was synthesized according to the *General procedure* using amine **A2** (95 mg, 0.50 mmol, 1.0 eq) and glycosyl halide **G1** (366 mg, 1.00 mmol, 2.0 eq) in the presence of Cs_2CO_3 (245 mg, 0.30 mmol, 1.5 eq). After the reaction, the mixture was purified by silica gel flash chromatography (petroleum ether/EtOAc = 1:1 to 1:3) to give **GA12** as a white solid (271 mg, 96%).

$[\alpha]_D^{25}$ = -17.3 (*c* 1.0, $CHCl_3$).

^{1}H NMR (500 MHz, $CDCl_3$) δ 7.43–7.31 (m, 1H), 5.58 (d, *J* = 8.3 Hz, 1H), 5.24 (t, *J* = 9.5 Hz, 1H), 5.15–5.02 (m, 1H), 4.27 (dd, *J* = 12.6, 4.2 Hz, 1H), 4.06 (dd, *J* = 12.5, 2.3 Hz, 1H), 3.82 (ddd, *J* = 10.2, 4.2, 2.2 Hz, 1H), 3.78–3.22 (m, 8H), 2.03 (s, 3H), 2.00–1.94 (m, 9H).

^{13}C NMR (126 MHz, $CDCl_3$) δ 170.64, 170.60, 170.02, 169.45, 169.35, 152.52, 135.00, 130.14, 128.65, 127.04, 93.41, 72.42, 72.34, 70.04, 67.76, 61.33, 46.93, 44.03, 41.70, 20.74, 20.64, 20.58, 20.57.

HRMS (ESI) m/z calcd for $C_{26}H_{32}N_2O_{12}Na$ $[M+Na]^+$ 587.1847; **found**: 587.1851.

Compound GA13

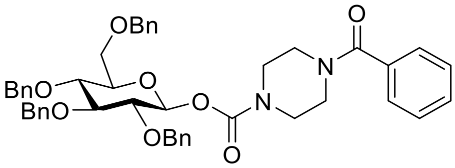

Compound **GA13** was synthesized according to the *General procedure* using amine **A2** (95 mg, 0.50 mmol, 1.0 eq) and glycosyl halide **G4** (839 mg, 1.50 mmol, 3.0 eq) in the presence of Cs_2CO_3 (245 mg, 0.30 mmol, 1.5 eq). After the reaction, the mixture was purified by silica gel flash chromatography (petroleum ether/EtOAc = 4:1 to 2:1 to 1:1) to give **GA13** (350 mg, 92%) as a white liquid.

$[\alpha]_D^{25}$ = -7.7 (*c* 0.1, CH_2Cl_2).

^{1}H NMR (600 MHz, $CDCl_3$) δ 7.47–7.40 (m, 3H), 7.40–7.35 (m, 2H), 7.35–7.25 (m, 18H), 7.16–7.12 (m, 2H), 5.62 (d, *J* = 8.1 Hz, 1H), 4.98–4.70 (m, 5H), 4.63 (d, *J* = 12.1 Hz, 1H), 4.54 (d, *J* = 10.8 Hz, 1H), 4.48 (d, *J* = 12.1 Hz, 1H), 3.82–3.71 (m, 4H), 3.69–3.11 (m, 10H).

^{13}C NMR (151 MHz, $CDCl_3$) δ 170.71, 153.36, 138.46, 138.26, 138.13, 137.94, 135.31, 130.20, 128.77, 128.57, 128.55, 128.52, 128.13, 127.99, 127.90, 127.89, 127.85, 127.60, 127.18, 95.84, 84.97, 75.78, 75.37, 75.08, 75.01, 73.65, 68.12, 47.36, 44.04, 41.94.

HRMS (ESI) m/z calcd for $C_{46}H_{48}N_2O_8Na$ $[M+Na]^+$ 779.3307; **found**: 779.3303.

Compound GA14

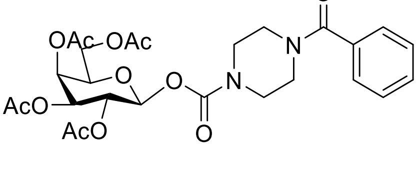

Compound **GA14** was synthesized according to the *General procedure* using amine **A2** (95 mg, 0.50 mmol, 1.0 eq) and glycosyl halide **G5** (226 mg, 0.55 mmol, 1.1 eq) in the presence of Cs_2CO_3 (245 mg, 0.30 mmol, 1.5 eq). After the reaction, the mixture was purified by silica gel flash chromatography (petroleum ether/EtOAc = 1:1 to 2:3) to give **GA14** as a yellow liquid (211 mg, 75%).

$[\alpha]_D^{25}$ = -0.5 (*c* 1.0, $CHCl_3$).

^{1}H NMR (500 MHz, $CDCl_3$) δ 7.47–7.35 (m, 5H), 5.59 (d, *J* = 8.3 Hz, 1H), 5.42 (d, *J* = 3.4 Hz, 1H), 5.40–5.24 (m, 1H), 5.09 (dd, *J* = 10.5, 3.4 Hz, 1H), 4.20–4.03 (m, 3H), 3.90–3.27 (m, 8H), 2.15 (s, 3H), 2.03 (s, 6H), 1.99 (s, 3H).

^{13}C NMR (126 MHz, $CDCl_3$) δ 170.79, 170.46, 170.23, 170.06, 169.68, 152.72, 135.13, 130.26, 128.78, 127.16, 94.00, 71.57, 70.61, 67.89, 66.82, 60.98, 47.31, 44.47, 41.89, 20.87, 20.80, 20.76, 20.67.

HRMS (ESI) m/z calcd for $C_{26}H_{32}N_2O_{12}Na$ $[M+Na]^+$ 587.1847; **found**: 587.1853.

Compound GA15

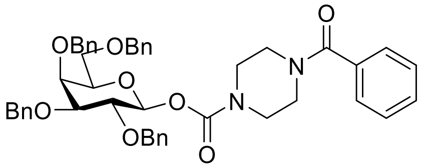

Compound **GA15** was synthesized according to the *General procedure* using amine **A2** (95 mg, 0.50 mmol, 1.0 eq) and glycosyl halide **G7** (839 mg, 1.50 mmol, 3.0 eq) in the presence of Cs_2CO_3 (245 mg, 0.30 mmol, 1.5 eq). After the reaction, the mixture was purified by silica gel flash chromatography (petroleum ether/EtOAc = 3:1 to 1:1) to give **GA15** as a white liquid (305 mg, 81%).

$[\alpha]_D^{25}$ = -18.2 (*c* 1.0, $CHCl_3$).

^{1}H NMR (500 MHz, $CDCl_3$) δ 7.49–7.26 (m, 25H), 5.58 (d, *J* = 8.1 Hz, 1H), 4.95 (d, *J* = 11.4 Hz, 1H), 4.88 (d, *J* = 11.5 Hz, 1H), 4.78–4.69 (m, 3H), 4.65 (d, *J* = 11.4 Hz, 1H), 4.49–4.40 (m, 2H), 4.03 (d, *J* = 2.8 Hz, 1H), 3.98 (bs, 1H), 3.83–3.06 (m, 12H).

^{13}C NMR (126 MHz, $CDCl_3$) δ 170.62, 153.36, 138.53, 138.51, 138.22, 137.79, 135.30, 130.11, 128.70, 128.52, 128.51, 128.42, 128.33, 128.06, 127.93, 127.79, 127.77, 127.71, 127.65, 127.12, 96.09, 82.54, 75.26, 74.89, 73.92, 73.56, 73.17, 72.95, 67.80.

HRMS (ESI) m/z calcd for $C_{46}H_{48}N_2O_8Na$ $[M+Na]^+$ 779.3307; **found**: 779.3310.

Compound GA16

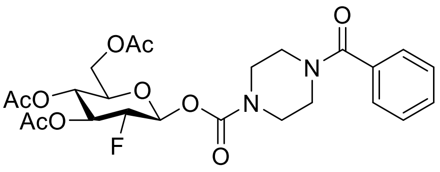

Compound **GA16** was synthesized according to the *General procedure* using amine **A2** (54 mg, 0.28 mmol, 1.0 eq) and glycosyl halide **G9** (210 mg, 0.59 mmol, 3.0 eq) in the presence of Cs_2CO_3 (139 mg, 0.43 mmol, 1.5 eq). After the reaction, the mixture was purified by silica gel flash chromatography (petroleum ether/EtOAc = 1:1) to give **GA16** as a white liquid (136 mg, 91%).

$[\alpha]_D^{25}$ = 27.1 (*c* 1.0, $CHCl_3$).

^{1}H NMR (500 MHz, $CDCl_3$) δ 7.45–7.34 (m, 5H), 5.75 (dd, *J* = 8.2, 2.9 Hz, 1H), 5.36 (dt, *J* = 14.3, 9.3 Hz, 1H), 5.05 (t, *J* = 9.7 Hz, 1H), 4.59–4.32 (m, 1H), 4.28 (dd, *J* = 12.6, 4.3 Hz, 1H), 4.12–4.04 (m, 1H), 3.85 (ddd, *J* = 10.2, 4.3, 2.2 Hz, 1H), 3.81–3.28 (m, 8H), 2.05 (s, 3H), 2.04 (s, 3H), 2.00 (s, 3H).

^{13}C NMR (126 MHz, $CDCl_3$) δ 170.70, 170.57, 169.90, 169.59, 152.55, 135.14, 130.18, 128.71, 127.11, 92.99 (d, *J* = 24.3 Hz), 88.27 (d, *J* = 192.3 Hz), 72.78 (d, *J* = 19.7 Hz), 72.57, 67.62 (d, *J* = 7.1 Hz), 61.34, 44.11, 20.75, 20.67, 20.58.

HRMS (ESI) m/z calculated for $C_{24}H_{29}N_2O_{10}FNa$ $[M+Na]^+$ 547.1698; **found**: 547.1703.

Compound GA18

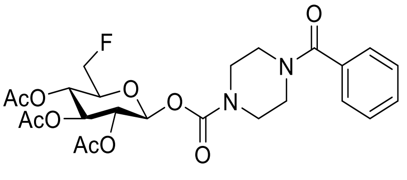

Compound **GA18** was synthesized according to the *General procedure* using amine **A2** (190 mg, 1.00 mmol, 1.0 eq) and glycosyl halide **G12** (740 mg, 2.00 mmol, 2.0 eq) in the presence of Cs_2CO_3 (490 mg, 1.50 mmol, 1.5 eq). After the reaction, the mixture was purified by silica gel flash chromatography (petroleum ether/EtOAc = 2:1) to give **GA18** as a white solid (444 mg, 85%).

$[\alpha]_D^{25}$ = -12.8 (*c* 1.0, $CHCl_3$).

^{1}H NMR (500 MHz, $CDCl_3$) δ 7.45–7.32 (m, 5H), 5.63 (d, *J* = 8.3 Hz, 1H), 5.28 (t, *J* = 9.5 Hz, 1H), 5.11 (t, *J* = 9.7 Hz, 2H), 4.50 (ddd, *J* = 45.2, 10.7, 2.4 Hz, 1H), 4.41 (ddd, *J* = 46.5, 10.6, 3.9 Hz, 1H), 3.83 (dddd, *J* = 23.3, 10.2, 3.8, 2.3 Hz, 1H), 3.76–3.18 (m, 8H), 2.01 (s, 3H), 2.01–1.98 (m, 6H).

^{13}C NMR (126 MHz, $CDCl_3$) δ 170.67, 170.09, 169.37, 169.30, 152.57, 135.10, 130.16, 128.69, 127.08, 93.37, 80.59 (d, *J* = 176.4 Hz), 77.42, 77.16, 76.91, 73.02 (d, *J* = 19.5 Hz), 72.41, 70.06, 67.61 (d, *J* = 6.7 Hz), 47.10, 44.01, 41.76, 20.65, 20.61, 20.59.

HRMS (ESI) m/z calcd for $C_{24}H_{29}N_2O_{10}FNa$ $[M+Na]^+$ 547.1698; **found**: 547.1698.

Compound GA19

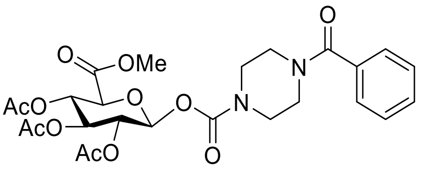

Compound **GA19** was synthesized according to the *General procedure* using amine **A2** (95 mg, 0.50 mmol, 1.0 eq) and glycosyl halide **G13** (218 mg, 0.55 mmol, 1.1 eq) in the presence of Cs_2CO_3 (245 mg, 0.75 mmol, 1.5 eq). After the reaction, the mixture was purified by silica gel flash chromatography (petroleum ether/EtOAc = 1:1) to give **GA19** as a yellow liquid (149 mg, 54%).

$[\alpha]_D^{25}$ = -1.9 (*c* 1.0, $CHCl_3$).

^{1}H NMR (500 MHz, $CDCl_3$) δ 7.48–7.36 (m, 5H), 5.68 (d, *J* = 7.9 Hz, 1H), 5.34 (t, *J* = 9.3 Hz, 1H), 5.25–5.11 (m, 2H), 4.19 (d, *J* = 9.8 Hz, 1H), 3.73 (s, 3H), 3.68–3.25 (m, 8H), 2.03 (s, 9H).

^{13}C NMR (126 MHz, $CDCl_3$) δ 170.82, 169.93, 169.60, 169.39, 166.96, 135.17, 130.29, 128.81, 127.19, 93.26, 72.94, 71.61, 70.06, 69.30, 53.17, 20.74, 20.71, 20.59.

HRMS (ESI) m/z calcd for $C_{25}H_{30}N_2O_{12}Na$ $[M+Na]^+$ 573.1691; **found**: 573.1698.

Compound GA20

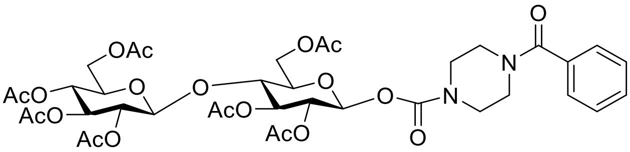

Compound **GA20** was synthesized according to the *General procedure* using amine **A2** (190 mg, 1.00 mmol, 1.0 eq) and glycosyl halide **G15** (1048 mg, 1.50 mmol, 1.5 eq) in the presence of Cs_2CO_3 (490 mg, 1.50 mmol, 1.5 eq). After the reaction, the mixture was purified by silica gel flash chromatography (petroleum ether/EtOAc = 1:1) to give **GA20** as a white solid (587 mg, 69%).

$[\alpha]_D^{25}$ = -21.1 (*c* 1.0, $CHCl_3$).

^{1}H NMR (500 MHz, $CDCl_3$) δ 7.46–7.27 (m, 5H), 5.57–5.50 (m, 1H), 5.24–5.16 (m, 1H), 5.11–5.04 (m, 1H), 5.04–4.94 (m, 2H), 4.88–4.80 (m, 1H), 4.50–4.38 (m, 2H), 4.34–4.26 (m, 1H), 4.11–3.93 (m, 3H), 3.82–3.12 (m, 23H), 2.09–1.87 (m, 21H).

^{13}C NMR (126 MHz, $CDCl_3$) δ 170.60, 170.43, 170.20, 170.16, 169.56, 169.53, 169.27, 168.97, 152.50, 135.09, 130.09, 128.63, 127.02, 100.63, 93.32, 75.90, 73.29, 72.84, 71.93, 71.86, 71.47, 70.32, 67.78, 61.57, 43.82, 20.85, 20.63, 20.50, 20.47.

HRMS (ESI) m/z calcd for $C_{38}H_{48}N_2O_{20}Na$ $[M+Na]^+$ 875.2693; **found**: 875.2702.

Compound GA22

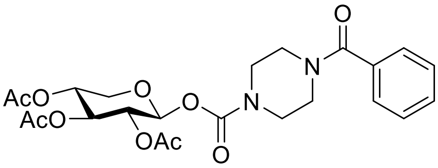

Compound **GA22** was synthesized according to the *General procedure* using amine **A2** (48 mg, 0.25 mmol, 1.0 eq) and glycosyl halide **G17** (221 mg, 0.75 mmol, 5.0 eq) in the presence of Cs_2CO_3 (123 mg, 0.375 mmol, 1.5 eq). After the reaction, the mixture was purified by silica gel flash chromatography (petroleum ether/EtOAc = 1:1 to 1:2) to give **GA22** as a yellow liquid (108.6 mg, 88%).

$[\alpha]_D^{25}$ = -23.6 (*c* 1.0, $CHCl_3$).

^{1}H NMR (500 MHz, $CDCl_3$) (mixture of α and β anomers) δ 7.46–7.20 (m, 5.42H), 6.12 (d, *J* = 3.6 Hz, 0.08H, H1-α), 5.56 (d, *J* = 7.1 Hz, 1H, H1-β), 5.33–5.28 (m,

0.08H), 5.16 (t, J = 8.5 Hz, 1H), 4.96 (t, J = 7.7 Hz, 1.12H), 4.87 (td, J = 8.5, 5.0 Hz, 1H), 4.08–4.00 (m, 1H), 3.84–3.04 (m, 10.14H), 1.99–1.91 (m, 9.64H).

^{13}C NMR (126 MHz, $CDCl_3$) (mixture of α and β anomers) δ 170.43, 169.90, 169.63, 169.49, 169.41, 169.19, 152.52, 134.96, 129.94, 128.49, 126.91, 93.50 (C1-β), 90.79 (C1-α), 70.70, 69.36, 69.22, 69.19, 68.37, 68.22, 62.48, 60.51, 47.05, 43.81, 41.66, 20.54, 20.49, 20.42.

HRMS (ESI) m/z calcd for $C_{23}H_{28}N_2O_{10}Na$ $[M+Na]^+$ 515.1636; **found**: 515.1636.

Compound GA23

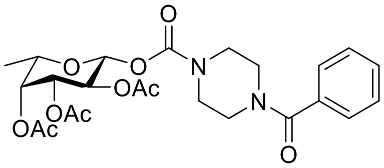

Compound **GA23** was synthesized according to the *General procedure* using amine **A2** (95 mg, 0.50 mmol, 1.0 eq) and glycosyl halide **G19** (308 mg, 1.00 mmol, 2.0 eq) in the presence of Cs_2CO_3 (245 mg, 0.75 mmol, 1.5 eq). After the reaction, the mixture was purified by silica gel flash chromatography (petroleum ether/EtOAc = 3:2 to 1:3) to give **GA23** as a white solid (245 mg, 97%).

$[\alpha]_D^{25}$ = 7.6 (*c* 1.0, $CHCl_3$).

^{1}H NMR (500 MHz, $CDCl_3$) δ 7.38–7.28 (m, 5H), 5.51 (d, J = 8.3 Hz, 1H), 5.28–5.15 (m, 2H), 5.03 (dd, J = 10.5, 3.5 Hz, 1H), 3.94–3.88 (m, 1H), 3.82–3.07 (m, 8H), 2.09 (s, 3H), 1.95 (s, 3H), 1.90 (s, 3H), 1.14 (d, J = 6.3 Hz, 3H).

^{13}C NMR (126 MHz, $CDCl_3$) δ 170.44, 170.32, 169.78, 169.48, 152.69, 135.02, 129.94, 128.50, 126.94, 93.77, 77.42, 77.16, 76.90, 70.77, 69.88, 67.80, 47.12, 43.87, 41.68, 20.63, 20.51, 20.44, 15.78.

HRMS (ESI) m/z calcd for $C_{24}H_{30}N_2O_{10}Na$ $[M+Na]^+$ 529.1793; **found**: 529.1797.

Compound GA24

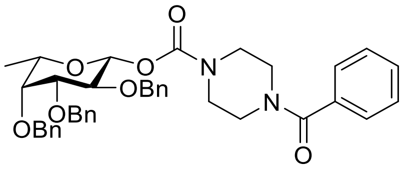

Compound **GA24** was synthesized according to the *General procedure* using amine **A2** (57 mg, 0.30 mmol, 1.0 eq) and glycosyl halide **G20** (408 mg, 0.90 mmol, 3.0 eq) in the presence of Cs_2CO_3 (147 mg, 0.45 mmol, 1.5 eq). After the reaction, the mixture was purified by silica gel flash chromatography (petroleum ether/EtOAc = 4:1 to 1:1) to give **GA24** as a yellow liquid (32 mg, 16%).

$[\alpha]_D^{25}$ = 22.8 (*c* 1.0, $CHCl_3$).

^{1}H NMR (500 MHz, $CDCl_3$) δ 7.54–7.14 (m, 20H), 5.54 (d, *J* = 8.1 Hz, 1H), 4.99 (d, *J* = 11.6 Hz, 1H), 4.87 (d, *J* = 11.5 Hz, 1H), 4.81–4.61 (m, 4H), 3.96 (s, 1H), 3.70–3.56 (m, 4H), 3.60–3.14 (m, 6H), 2.98 (s, 1H), 1.19 (d, *J* = 6.3 Hz, 3H).

^{13}C NMR (126 MHz, $CDCl_3$) δ 170.69, 153.55, 138.61, 138.36, 138.33, 135.34, 130.15, 128.75, 128.72, 128.59, 128.47, 128.37, 127.91, 127.89, 127.75, 127.63, 127.17, 96.09, 82.93, 76.05, 75.28, 74.88, 73.29, 71.49, 42.79, 16.86.

HRMS (ESI) m/z calcd for $C_{39}H_{42}N_2O_7Na$ $[M+Na]^+$ 673.2884; **found**: 673.2891.

Compound GA25

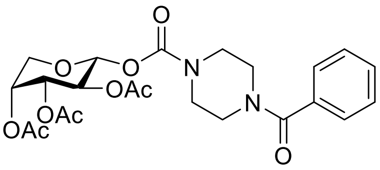

Compound **GA25** was synthesized according to the *General procedure* using amine **A2** (95 mg, 0.50 mmol, 1.0 eq) and glycosyl halide **G21** (294 mg, 1.0 mmol, 2.00 eq) in the presence of Cs_2CO_3 (245 mg, 0.75 mmol, 1.5 eq). After the reaction, the mixture was purified by silica gel flash chromatography (petroleum ether/EtOAc = 3:2 to 1:2) to give **GA25** as a yellow liquid (248 mg, 97%).

$[\alpha]_D^{25}$ = -3.6 (*c* 1.0, $CHCl_3$).

^{1}H NMR (500 MHz, $CDCl_3$) δ 7.40–7.28 (m, 5H), 5.52 (d, *J* = 7.1 Hz, 1H), 5.26–5.18

(m, 2H), 5.08 (dd, J = 9.5, 3.5 Hz, 1H), 3.94 (dd, J = 13.1, 3.4 Hz, 1H), 3.83–3.13 (m, 0H), 2.05 (s, 3H), 1.98 (s, 3H), 1.95 (s, 3H).

^{13}C NMR (126 MHz, $CDCl_3$) δ 170.47, 169.95, 169.64, 169.36, 152.62, 135.01, 129.98, 128.53, 126.95, 93.64, 69.56, 68.00, 67.11, 43.84, 20.74, 20.63, 20.52.

HRMS (ESI) m/z calcd for $C_{23}H_{28}N_2O_{10}Na$ $[M+Na]^+$ 515.1636; **found**: 515.1641.

Compound GA35

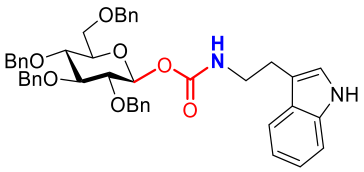

Compound **GA35** was synthesized according to the *General procedure* using amine **A3** (80 mg, 0.50 mmol, 1.0 eq) and glycosyl halide **G4** (836.7 mg, 1.50 mmol, 3.0 eq) in the presence of Cs_2CO_3 (245 mg, 0.75 mmol, 1.5 eq). After the reaction, the mixture was purified by silica gel flash chromatography (petroleum ether/EtOAc = 8:1 to 5:1) to give **GA35** as a white solid (228 mg, 63%).

$[\alpha]_D^{25}$ = -4.7 (c 0.1, CH_2Cl_2).

^{1}H NMR (600 MHz, $CDCl_3$) δ 7.89 (s, 1H), 7.58 (d, J = 7.9 Hz, 1H), 7.34–7.23 (m, 18H), 7.21–7.16 (m, 2H), 7.15–7.08 (m, 3H), 6.93 (d, J = 2.3 Hz, 1H), 5.58 (d, J = 8.2 Hz, 1H), 4.88 (d, J = 10.9 Hz, 1H), 4.86–4.78 (m, 2H), 4.74 (q, J = 8.2, 7.0 Hz, 1H), 4.69 (s, 2H), 4.62 (d, J = 12.1 Hz, 1H), 4.52 (d, J = 10.8 Hz, 1H), 4.47 (d, J = 12.1 Hz, 1H), 3.77–3.68 (m, 4H), 3.62–3.44 (m, 4H), 2.98–2.93 (m, 2H).

^{13}C NMR (151 MHz, $CDCl_3$) δ 154.41, 138.57, 138.48, 138.22, 138.10, 136.48, 128.53, 128.50, 128.43, 128.10, 128.05, 128.02, 127.99, 127.89, 127.81, 127.75, 127.30, 122.39, 122.33, 119.64, 118.79, 112.62, 111.37, 95.25, 84.93, 81.27, 75.87, 75.28, 75.12, 74.92, 73.62, 68.27, 41.24, 25.58.

HRMS (ESI) m/z calcd for $C_{45}H_{46}N_2O_7Na$ $[M+Na]^+$ 749.3201; **found**: 749.3197.

Compound GA36

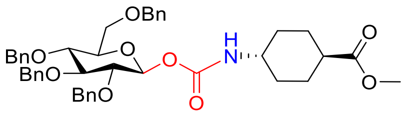

Compound **GA36** was synthesized according to the *General procedure* using amine **A4** (95 mg, 0.49 mmol, 1.0 eq) and glycosyl halide **G4** (822 mg, 1.47 mmol, 3.0 eq) in the presence of Cs_2CO_3 (240 mg, 0.735 mmol, 1.5 eq). After the reaction, the mixture was purified by silica gel flash chromatography (petroleum ether/EtOAc = 8:1 to 4:1) to give **GA36** (348 mg, 98%) as a white solid.

$[\alpha]_D^{25}$ = -0.9 (*c* 0.1, CH_2Cl_2).

^{1}H NMR (600 MHz, $CDCl_3$) δ 7.34–7.24 (m, 18H), 7.15–7.10 (m, 2H), 5.54 (d, *J* = 8.1 Hz, 1H), 4.89 (d, *J* = 10.9 Hz, 1H), 4.83–4.78 (m, 2H), 4.76–4.71 (m, 2H), 4.63 (d, *J* = 12.1 Hz, 1H), 4.52 (d, *J* = 10.0 Hz, 2H), 4.46 (d, *J* = 12.1 Hz, 1H), 3.77–3.69 (m, 4H), 3.67 (s, 3H), 3.58–3.44 (m, 3H), 2.23 (tt, *J* = 12.1, 3.6 Hz, 1H), 2.13–1.98 (m, 4H), 1.59–1.47 (m, 2H), 1.17–1.07 (m, 2H).

^{13}C NMR (151 MHz, $CDCl_3$) δ 175.79, 153.49, 138.54, 138.41, 138.20, 138.07, 128.54, 128.51, 128.47, 128.17, 128.08, 128.04, 127.98, 127.87, 127.82, 127.79, 95.11, 84.96, 81.34, 75.87, 75.29, 75.09, 73.60, 68.19, 51.81, 49.64, 42.35, 32.40, 32.26, 27.79.

HRMS (ESI) m/z calcd for $C_{43}H_{49}NO_9Na$ $[M+Na]^+$ 746.3303; **found**: 746.3300.

Compound GA37

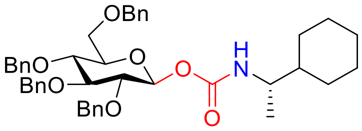

Compound **GA37** was synthesized according to the *General procedure* using amine **A5** (127 mg, 1.0 mmol, 1.0 eq) and glycosyl halide **G4** (1680 mg, 3.0 mmol, 3.0 eq) in the presence of Cs_2CO_3 (490 mg, 1.5 mmol, 1.5 eq). After the reaction, the mixture was purified by silica gel flash chromatography (petroleum ether/EtOAc = 8:1) to give **GA37** as a white solid (374 mg, 54%).

$[\alpha]_D^{25}$ = -7.2 (*c* 1.0, $CHCl_3$).

^{1}H NMR (500 MHz, $CDCl_3$) (mixture of rotamers) δ 7.41–7.28 (m, 18H), 7.23–7.17 (m, 2H), 5.67–5.61 (m, 1H), 4.97 (d, *J* = 10.9 Hz, 1H), 4.93–4.83 (m, 3H), 4.82–4.78 (m, 1H), 4.72–4.65 (m, 2H), 4.59 (d, *J* = 10.7 Hz, 1H), 4.53 (d, *J* = 12.1 Hz, 1H),

3.86–3.75 (m, 4H), 3.71–3.58 (m, 3H), 1.85–1.66 (m, 5H), 1.42–1.20 (m, 4H), 1.16 (d, *J* = 6.9 Hz, 3H), 1.12–0.93 (m, 2H).

^{13}C NMR (126 MHz, $CDCl_3$) (mixture of rotamers) δ 153.86, 153.84, 138.50, 138.48, 138.32, 138.18, 138.13, 138.03, 138.00, 128.45, 128.42, 128.38, 128.15, 128.11, 128.00, 127.97, 127.94, 127.91, 127.78, 127.75, 127.72, 127.68, 95.08, 94.99, 84.91, 84.85, 81.28, 81.21, 77.36, 75.80, 75.78, 75.27, 75.20, 75.02, 73.49, 73.48, 68.12, 68.09, 51.60, 43.30, 43.16, 29.11, 28.95, 28.88, 26.40, 26.20, 26.17, 18.13, 18.11.

HRMS (ESI) m/z calcd for $C_{43}H_{51}NO_7Na$ $[M+Na]^+$ 716.3558; **found**: 716.3564.

Compound A38

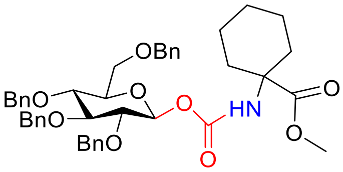

Compound **GA38** was synthesized according to the *General procedure* using amine **A6** (78 mg, 0.4 mmol, 1.0 eq) and glycosyl halide **G4** (671 mg, 1.2 mmol, 3.0 eq) in the presence of Cs_2CO_3 (196 mg, 0.6 mmol, 1.5 eq). After the reaction, the mixture was purified by silica gel flash chromatography (petroleum ether/EtOAc = 8:1 to 4:1) to give **GA38** (124mg, 43%) as a white solid.

$[\alpha]_D^{25}$ = 23.2 (*c* 0.1, CH_2Cl_2).

^{1}H NMR (600 MHz, $CDCl_3$) δ 7.38–7.23 (m, 18H), 7.16–7.12 (m, 2H), 5.53 (d, *J* = 8.1 Hz, 1H), 4.96–4.87 (m, 2H), 4.86–4.78 (m, 3H), 4.75 (d, *J* = 11.1 Hz, 1H), 4.63 (d, *J* = 12.1 Hz, 1H), 4.53 (d, *J* = 10.9 Hz, 1H), 4.49 (d, *J* = 12.1 Hz, 1H), 3.78–3.69 (m, 3H), 3.67 (s, 3H), 3.61–3.51 (m, 2H), 2.09 (d, *J* = 14.0 Hz, 1H), 1.96 (d, *J* = 13.9 Hz, 1H), 1.91–1.79 (m, 2H), 1.67–1.61 (m, 2H), 1.49–1.38 (m, 2H), 1.35–1.22 (m, 2H).

^{13}C NMR (151 MHz, $CDCl_3$) δ 174.49, 153.16, 138.54, 138.35, 138.18, 138.12, 128.57, 128.55, 128.48, 128.41, 128.09, 128.05, 127.92, 127.88, 127.85, 127.79, 95.11, 84.99, 81.07, 75.94, 75.40, 75.17, 75.06, 73.61, 68.20, 59.40, 52.56, 33.05, 32.08, 25.19, 21.39, 21.31.

HRMS (ESI) m/z calcd for $C_{43}H_{49}NO_9Na$ $[M+Na]^+$ 746.3306; **found**: 746.3300.

Compound GA39

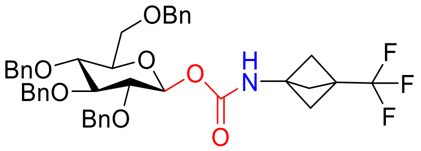

Compound **GA39** was synthesized according to the *General procedure* using amine **A7** (94 mg, 0.5 mmol, 1.0 eq) and glycosyl halide **G4** (839 mg, 1.0 mmol, 2.0 eq) in the presence of Cs_2CO_3 (245 mg, 0.75 mmol, 1.5 eq). After the reaction, the mixture was purified by silica gel flash chromatography (petroleum ether/EtOAc = 8:1) to give **GA39** (255 mg, 71%) as a yellow liquid.

$[\alpha]_D^{25}$ = 12.9 (*c* 0.1, CH_2Cl_2).

^{1}H NMR (600 MHz, $CDCl_3$) δ 7.36–7.21 (m, 18H), 7.18–7.10 (m, 2H), 5.50 (d, *J* = 8.1 Hz, 1H), 5.05 (s, 1H), 4.89 (d, *J* = 10.9 Hz, 1H), 4.85–4.79 (m, 2H), 4.77 (d, *J* = 11.5 Hz, 1H), 4.69 (d, *J* = 11.5 Hz, 1H), 4.63 (d, *J* = 12.1 Hz, 1H), 4.52 (d, *J* = 10.7 Hz, 1H), 4.47 (d, *J* = 12.1 Hz, 1H), 3.79–3.68 (m, 4H), 3.60–3.47 (m, 2H), 2.25 (s, 6H).

^{13}C NMR (151 MHz, $CDCl_3$) δ 153.12, 138.49, 138.12, 138.01, 128.57, 128.55, 128.51, 128.14, 128.10, 128.03, 127.94, 127.86, 123.38 (q, *J* = 274.1 Hz), 95.10, 84.92, 81.37, 77.37, 77.16, 76.95, 75.89, 75.41, 75.19, 75.16, 73.63, 68.13, 51.46, 45.49, 45.48, 35.09 (q, *J* = 40.3, 39.7 Hz).

HRMS (ESI) m/z calcd for $C_{41}H_{42}F_3NO_7Na$ $[M+Na]^+$ 740.2817; **found**: 740.2806.

Compound GA40

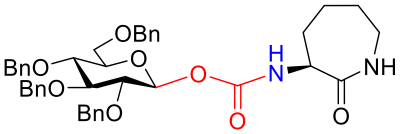

Compound **GA40** was synthesized according to the *General procedure* using amine **A8** (128 mg, 1.0 mmol, 1.0 eq) and glycosyl halide **G4** (1680 mg, 3.0 mmol, 3.0 eq) in presence of Cs_2CO_3 (490 mg, 1.5 mmol, 1.5 eq). After the reaction, the mixture was purified by silica gel flash chromatography (petroleum ether/EtOAc = 3:1 to 1:2) to give **GA40** as a white solid (662 mg, 95%).

$[\alpha]_D^{25}$ = 13.9 (*c* 1.0, $CHCl_3$).

^{1}**H NMR** (500 MHz, $CDCl_3$) δ 7.38–7.24 (m, 18H), 7.19–7.14 (m, 2H), 6.59 (t, *J* = 6.4 Hz, 1H), 6.31 (d, *J* = 6.0 Hz, 1H), 5.59 (d, *J* = 8.1 Hz, 1H), 4.93 (d, *J* = 10.9 Hz, 1H), 4.88–4.79 (m, 3H), 4.75 (d, *J* = 11.2 Hz, 1H), 4.66 (d, *J* = 12.1 Hz, 1H), 4.55 (d, *J* = 10.8 Hz, 1H), 4.51 (d, *J* = 12.1 Hz, 1H), 4.33 (ddd, *J* = 11.5, 6.0, 1.8 Hz, 1H), 3.81–3.72 (m, 4H), 3.64–3.57 (m, 2H), 3.30–3.17 (m, 2H), 2.21–2.14 (m, 1H), 2.05–1.98 (m, 1H), 1.86–1.71 (m, 2H), 1.61–1.49 (m, 1H), 1.45–1.32 (m, 1H).

^{13}**C NMR** (126 MHz, $CDCl_3$) δ 175.06, 153.42, 138.53, 138.16, 138.07, 128.46, 128.45, 128.39, 128.16, 128.03, 127.96, 127.93, 127.81, 127.75, 127.72, 127.70, 95.42, 84.88, 80.99, 77.36, 75.76, 75.35, 75.05, 74.98, 73.51, 68.19, 53.77, 42.13, 31.74, 28.90, 28.04.

HRMS (ESI) m/z calcd for $C_{41}H_{46}N_2O_8Na$ $[M+Na]^+$ 717.3146; **found**: 717.3151.

Compound GA41

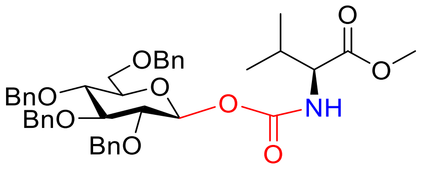

Compound **GA41** was synthesized according to the *General procedure* using amine **A9** (168 mg, 1.0 mmol, 1.0 eq) and glycosyl halide **G4** (1680 mg, 3.0 mmol, 3.0 eq) in presence of Cs_2CO_3 (490 mg, 1.5 mmol, 1.5 eq). After the reaction, the mixture was purified by silica gel flash chromatography (petroleum ether/EtOAc = 10:1) to give **GA41** as a white solid (495 mg, 71%).

$[\alpha]_D^{25}$ = -3.2 (*c* 1.0, $CHCl_3$).

^{1}**H NMR** (500 MHz, $CDCl_3$) δ 7.43–7.24 (m, 18H), 7.20–7.14 (m, 2H), 5.60 (d, *J* = 8.1 Hz, 1H), 5.32 (d, *J* = 9.1 Hz, 1H), 4.94 (d, *J* = 10.9 Hz, 1H), 4.88–4.81 (m, 3H), 4.77 (d, *J* = 11.1 Hz, 1H), 4.66 (d, *J* = 12.1 Hz, 1H), 4.56 (d, *J* = 10.8 Hz, 1H), 4.52 (d, *J* = 12.1 Hz, 1H), 4.35 (dd, *J* = 9.1, 4.7 Hz, 1H), 3.84–3.71 (m, 7H), 3.64–3.56 (m, 2H), 2.21 (pd, *J* = 6.9, 4.8 Hz, 1H), 1.01 (d, *J* = 6.9 Hz, 3H), 0.94 (d, *J* = 6.9 Hz, 3H).

^{13}**C NMR** (126 MHz, $CDCl_3$) δ 172.21, 154.27, 138.49, 138.14, 138.03, 128.51, 128.47, 128.42, 128.41, 128.38, 128.02, 128.00, 127.96, 127.84, 127.82, 127.79, 127.74, 95.47, 84.92, 80.86, 77.33, 75.85, 75.39, 75.08, 75.04, 73.54, 68.09, 59.15,

52.28, 31.36, 19.01, 17.66.

HRMS (ESI) m/z calcd for $C_{41}H_{47}NO_9Na$ $[M+Na]^+$ 720.3143; **found**: 720.3150.

Compound GA42

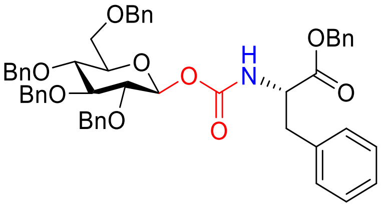

Compound **GA42** was synthesized according to the *General procedure* using amine **A10** (292 mg, 1.0 mmol, 1.0 eq) and glycosyl halide **G4** (1680 mg, 3.0 mmol, 3.0 eq) in presence of Cs_2CO_3 (490 mg, 1.5 mmol, 1.5 eq). After the reaction, the mixture was purified by silica gel flash chromatography (petroleum ether/EtOAc = 8:1) to give **GA42** as a white solid (469 mg, 57%).

$[\alpha]_D^{25}$ = -9.4 (*c* 1.0, $CHCl_3$).

^{1}H NMR (600 MHz, $CDCl_3$) δ 7.32–7.13 (m, 26H), 7.12–7.08 (m, 2H), 7.03–6.97 (m, 2H), 5.53 (d, *J* = 8.2 Hz, 1H), 5.20 (d, *J* = 8.2 Hz, 1H), 5.11–5.04 (m, 2H), 4.86 (d, *J* = 10.9 Hz, 1H), 4.80–4.75 (m, 2H), 4.72–4.63 (m, 3H), 4.60 (d, *J* = 12.1 Hz, 1H), 4.49 (d, *J* = 10.7 Hz, 1H), 4.45 (d, *J* = 12.1 Hz, 1H), 3.78–3.64 (m, 4H), 3.56–3.47 (m, 2H), 3.12–3.05 (m, 2H).

^{13}C NMR (151 MHz, $CDCl_3$) δ 170.99, 153.73, 138.48, 138.13, 138.06, 138.01, 135.33, 135.06, 129.45, 128.73, 128.69, 128.68, 128.62, 128.47, 128.45, 128.43, 128.40, 128.31, 128.03, 127.94, 127.82, 127.78, 127.75, 127.24, 95.47, 84.92, 80.60, 77.27, 75.80, 75.34, 75.04, 74.89, 73.60, 68.15, 67.36, 54.87, 38.03.

HRMS (ESI) m/z calcd for $C_{51}H_{51}NO_9Na$ $[M+Na]^+$ 844.3456; **found**: 844.3457.

Compound GA43

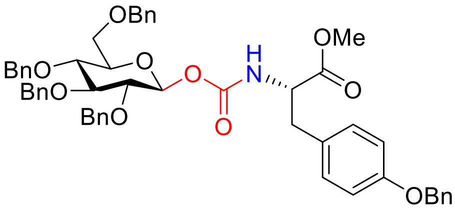

Compound **GA43** was synthesized according to the *General procedure* using amine **A11** (322 mg, 1.0 mmol, 1.0 eq) and glycosyl halide **G4** (1680 mg, 3.0 mmol, 3.00 eq) in presence of Cs_2CO_3 (490 mg, 1.5 mmol, 1.5 eq). After the reaction, the mixture was purified by silica gel flash chromatography (petroleum ether/EtOAc = 2:1) to give **GA43** as a white solid (549 mg, 64%).

$[\alpha]_D^{25}$ = 21.4 (*c* 1.0, $CHCl_3$).

^{1}H NMR (500 MHz, $CDCl_3$) δ 7.55–7.32 (m, 23H), 7.26 (dd, *J* = 7.1, 2.2 Hz, 2H), 7.15 (d, *J* = 8.1 Hz, 2H), 7.01 (d, *J* = 8.1 Hz, 2H), 5.72 (d, *J* = 8.1 Hz, 1H), 5.42 (d, *J* = 8.1 Hz, 1H), 5.08 (s, 2H), 5.02 (d, *J* = 10.9 Hz, 1H), 4.98–4.91 (m, 2H), 4.88 (d, *J* = 11.3 Hz, 1H), 4.85–4.80 (d, *J* = 11.3 Hz, 1H), 4.78–4.71 (m, 2H), 4.66 (d, *J* = 10.8 Hz, 1H), 4.59 (d, *J* = 12.2 Hz, 1H), 3.92–3.81 (m, 4H), 3.78 (s, 3H), 3.71–3.65 (m, 2H), 3.18 (d, *J* = 5.5 Hz, 2H).

^{13}C NMR (126 MHz, $CDCl_3$) δ 171.62, 157.99, 153.70, 138.37, 138.03, 138.00, 137.89, 136.89, 130.33, 128.52, 128.36, 128.33, 128.25, 128.22, 127.92, 127.85, 127.82, 127.70, 127.68, 127.65, 127.61, 127.45, 115.01, 95.36, 84.78, 80.57, 77.18, 75.68, 75.23, 74.91, 74.80, 73.46, 69.88, 68.05, 54.89, 52.26, 37.00.

HRMS (ESI) m/z calcd for $C_{52}H_{53}NO_{10}Na$ $[M+Na]^+$ 874.3562; **found**: 874.3566.

Compound GA44

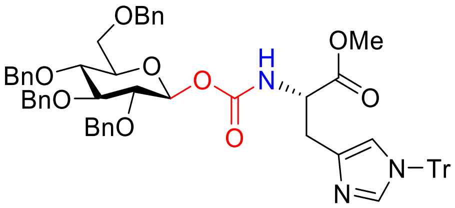

Compound **GA44** was synthesized according to the *General procedure* using amine **A12** (224 mg, 0.5 mmol, 1.0 eq) and glycosyl halide **G4** (839 mg, 1.5 mmol, 3.0 eq) in presence of Cs_2CO_3 (245 mg, 0.75 mmol, 1.5 eq). After the reaction, the mixture was purified by silica gel flash chromatography (petroleum ether/EtOAc = 3:1 to 1:2) to give **GA44** as a white solid (254 mg, 52%).

$[\alpha]_D^{25}$ = 8.0 (*c* 1.0, $CHCl_3$).

^{1}H NMR (500 MHz, $CDCl_3$) δ 7.44–7.20 (m, 27H), 7.19–7.15 (m, 2H), 7.15–7.09 (m,

6H), 6.83 (d, J = 8.0 Hz, 1H), 6.60 (d, J = 1.4 Hz, 1H), 5.64 (d, J = 8.1 Hz, 1H), 4.95 (d, J = 10.9 Hz, 1H), 4.91 (d, J = 11.0 Hz, 1H), 4.87–4.82 (m, 2H), 4.74 (d, J = 11.0 Hz, 1H), 4.68–4.62 (m, 2H), 4.57 (d, J = 10.8 Hz, 1H), 4.49 (d, J = 12.1 Hz, 1H), 3.82–3.71 (m, 4H), 3.66–3.56 (m, 5H), 3.18–3.01 (m, 2H).

^{13}C NMR (126 MHz, $CDCl_3$) δ 171.69, 154.26, 142.28, 138.75, 138.59, 138.18, 138.09, 136.29, 129.77, 128.53, 128.41, 128.40, 128.35, 128.33, 128.11, 127.96, 127.92, 127.74, 127.68, 127.65, 127.62, 119.62, 95.57, 84.76, 80.93, 77.31, 75.73, 75.34, 75.32, 75.01, 74.91, 73.51, 68.23, 54.36, 52.14, 29.83.

HRMS (ESI) m/z calcd for $C_{61}H_{59}N_3O_9Na$ $[M+Na]^+$ 1000.4144; **found**: 1000.4153.

Compound GA45

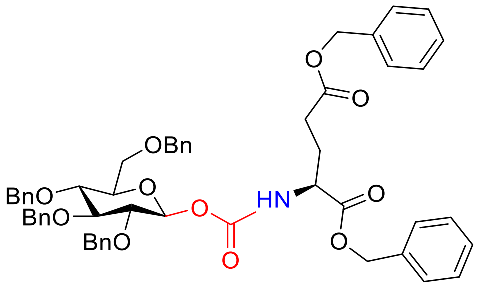

Compound **GA45** was synthesized according to the *General procedure* using amine **A13** (110 mg, 0.3 mmol, 1.0 eq) and glycosyl halide **G4** (503 mg, 0.9 mmol, 3.0 eq) in presence of Cs_2CO_3 (147 mg, 0.45 mmol, 1.5 eq). After the reaction, the mixture was purified by silica gel flash chromatography (petroleum ether/EtOAc = 8:1), then the mixture was purified by silica gel flash chromatography (dichloromethane/EtOAc = 10:1) to give **GA45** (141 mg, 53%) as a white solid.

$[\alpha]_D^{25}$ = -0.6 (c 1.0, CH_2Cl_2).

^{1}H NMR (600 MHz, $CDCl_3$) δ 7.38–7.21 (m, 28H), 7.17–7.09 (m, 2H), 5.53 (d, J = 8.1 Hz, 1H), 5.45 (d, J = 8.1 Hz, 1H), 5.19–5.11 (m, 2H), 5.08 (s, 2H), 4.89 (d, J = 10.9 Hz, 1H), 4.83–4.77 (m, 2H), 4.75 (d, J = 11.2 Hz, 1H), 4.71 (d, J = 11.2 Hz, 1H), 4.61 (d, J = 12.1 Hz, 1H), 4.51 (d, J = 10.7 Hz, 1H), 4.48–4.41 (m, 2H), 3.77–3.67 (m, 4H), 3.57–3.50 (m, 2H), 2.50–2.35 (m, 2H), 2.29–2.20 (m, 1H), 2.07–1.98 (m, 1H).

^{13}C NMR (151 MHz, $CDCl_3$) δ 172.53, 171.41, 154.01, 138.56, 138.20, 138.18, 138.08, 135.84, 135.19, 128.81, 128.70, 128.55, 128.52, 128.47, 128.42, 128.40,

128.08, 128.02, 127.89, 127.87, 127.82, 127.79, 95.61, 84.92, 80.92, 75.87, 75.43, 75.13, 75.08, 73.61, 68.15, 67.60, 66.71, 53.52, 30.17, 27.63.

HRMS (ESI) m/z calcd for $C_{54}H_{55}NO_{11}Na$ $[M+Na]^+$ 916.3670; **found**: 916.3667.

Compound GA46

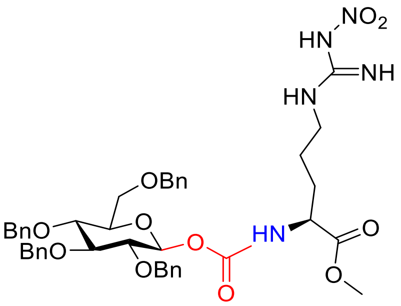

Compound **GA46** was synthesized according to the *General procedure* using amine **A14** (270 mg, 1.0 mmol, 1.0 eq) and glycosyl halide **G4** (1680 mg, 3.0 mmol, 3.0 eq) in presence of Cs_2CO_3 (490 mg, 1.5 mmol, 1.5 eq). After the reaction, the mixture was purified by silica gel flash chromatography (petroleum ether/EtOAc = 1:1 to 1:3) to give **GA46** as a white solid (450 mg, 56%).

$[\alpha]_D^{25}$ = 5.9 (*c* 1.0, $CHCl_3$).

^{1}H NMR (600 MHz, $CDCl_3$) δ 7.40–7.26 (m, 18H), 7.19–7.15 (m, 2H), 5.89 (bs, 1H), 5.58 (d, *J* = 8.1 Hz, 1H), 4.95 (d, *J* = 11.0 Hz, 1H), 4.90–4.75 (m, 4H), 4.59 (d, *J* = 12.0 Hz, 1H), 4.56–4.48 (m, 2H), 4.45–4.36 (m, 1H), 3.80–3.64 (m, 9H), 3.63–3.57 (m, 1H), 3.35 (bs, 1H), 3.26–3.15 (m, 1H), 2.49 (bs, 1H), 2.00–1.89 (m, 1H), 1.81–1.63 (m, 3H).

^{13}C NMR (151 MHz, $CDCl_3$) δ 172.11, 159.28, 154.92, 138.29, 137.98, 137.84, 137.49, 128.47, 128.42, 128.22, 128.15, 127.95, 127.91, 127.87, 127.79, 95.64, 84.63, 80.64, 77.30, 75.77, 75.13, 75.03, 75.00, 73.38, 68.36, 52.81, 40.34, 30.04, 24.35.

HRMS (ESI) m/z calcd for $C_{42}H_{50}N_5O_{11}Na$ $[M+Na]^+$ 800.3501; **found**: 800.3508.

Compound GA47

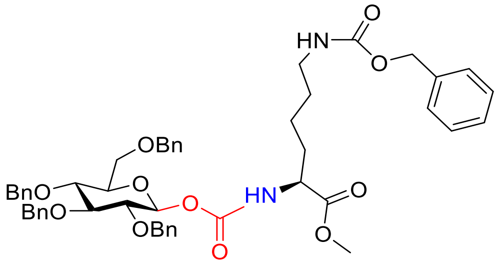

Compound **GA47** was synthesized according to the *General procedure* using amine **A15** (331 mg, 1.0 mmol, 1.0 eq) and glycosyl halide **G4** (1680 mg, 3.0 mmol, 3.0 eq) in presence of Cs_2CO_3 (490 mg, 1.5 mmol, 1.5 eq). After the reaction, the mixture was purified by silica gel flash chromatography (petroleum ether/EtOAc = 2:1) to give **GA47** as a white solid (695 mg, 81%).

$[\alpha]_D^{25}$ = 7.3 (*c* 1.0, $CHCl_3$).

^{1}H NMR (500 MHz, $CDCl_3$) δ 7.39–7.28 (m, 23H), 7.21–7.15 (m, 2H), 5.64–5.56 (m, 2H), 5.20–5.02 (m, 3H), 4.95 (d, *J* = 10.9 Hz, 1H), 4.89–4.83 (m, 3H), 4.78 (d, *J* = 11.3 Hz, 1H), 4.63 (d, *J* = 12.0 Hz, 1H), 4.55 (d, *J* = 10.8 Hz, 1H), 4.49 (d, *J* = 11.9 Hz, 1H), 4.39 (td, *J* = 8.1, 4.9 Hz, 1H), 3.75 (s, 6H), 3.63–3.58 (m, 2H), 3.22–3.11 (m, 2H), 1.93–1.82 (m, 1H), 1.77–1.66 (m, 1H), 1.56–1.46 (m, 2H), 1.44–1.34 (m, 2H).

^{13}C NMR (126 MHz, $CDCl_3$) δ 172.53, 156.59, 154.02, 138.41, 138.07, 138.04, 137.87, 136.63, 128.52, 128.41, 128.39, 128.36, 128.35, 128.27, 128.14, 128.08, 128.00, 127.89, 127.86, 127.77, 127.75, 127.70, 95.42, 84.74, 80.86, 77.28, 75.73, 75.17, 74.96, 74.93, 73.40, 68.17, 66.61, 53.75, 52.44, 40.44, 31.90, 29.34, 22.31.

HRMS (ESI) m/z calcd for $C_{50}H_{56}N_2O_{11}Na$ $[M+Na]^+$ 883.3776; **found**: 883.3781.

Compound GA48

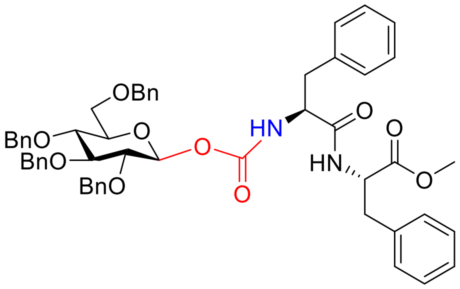

Compound **GA48** was synthesized according to the *General procedure* using amine **A16** (182 mg, 0.5 mmol, 1.0 eq) nd glycosyl halide **G4** (839 mg, 1.5 mmol, 3.0 eq) in presence of Cs_2CO_3 (245 mg, 0.75 mmol, 1.5 eq). After the reaction, the mixture was

purified by silica gel flash chromatography (toluene/EtOAc = 20:1 to 10:1) to give **GA48** as a yellow solid (268 mg, 60%).

$[\alpha]_D^{25}$ = 13.3 (*c* 1.0, $CHCl_3$).

^{1}H NMR (500 MHz, $CDCl_3$) δ 7.37–7.12 (m, 28H), 7.01–6.92 (m, 2H), 6.05 (d, *J* = 7.5 Hz, 1H), 5.54 (d, *J* = 8.1 Hz, 1H), 5.41 (d, *J* = 7.8 Hz, 1H), 4.91 (d, *J* = 10.9 Hz, 1H), 4.86–4.80 (m, 2H), 4.78–4.69 (m, 3H), 4.65 (d, *J* = 12.1 Hz, 1H), 4.54 (d, *J* = 10.7 Hz, 1H), 4.50 (d, *J* = 12.1 Hz, 1H), 4.38 (td, *J* = 7.7, 5.6 Hz, 1H), 3.81–3.70 (m, 4H), 3.67 (s, 3H), 3.61–3.54 (m, 2H), 3.14 (dd, *J* = 13.8, 5.8 Hz, 1H), 3.09–2.96 (m, 3H).

^{13}C NMR (126 MHz, $CDCl_3$) δ 171.25, 169.93, 153.95, 138.50, 138.14, 138.00, 136.11, 135.53, 129.53, 129.26, 128.88, 128.71, 128.51, 128.47, 128.41, 128.09, 127.99, 127.98, 127.87, 127.85, 127.81, 127.79, 127.29, 127.27, 95.60, 84.94, 80.70, 77.30, 75.84, 75.37, 75.10, 75.02, 73.64, 68.15, 56.24, 53.57, 52.42, 38.58, 37.93.

HRMS (ESI) m/z calcd for $C_{54}H_{56}N_2O_{10}Na$ $[M+Na]^+$ 915.3827; **found**: 915.3828.

Compound GA49

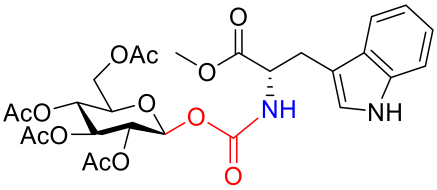

Compound **GA49** was synthesized according to the *General procedure* using amine **A17** (127 mg, 0.5 mmol, 1.0 eq) and glycosyl halide **G2** (226 mg, 0.55 mmol, 1.1 eq) in presence of Cs_2CO_3 (245 mg, 0.75 mmol, 1.5 eq). After the reaction, the mixture was purified by silica gel flash chromatography (petroleum ether/EtOAc = 2:1 to 2:3) to give **GA49** as a brown liquid (69 mg, 38%).

$[\alpha]_D^{25}$ = 4.2 (*c* 1.0, $CHCl_3$).

^{1}H NMR (500 MHz, $CDCl_3$) δ 8.29 (s, 1H), 7.50 (d, *J* = 7.9 Hz, 1H), 7.36 (d, *J* = 8.1 Hz, 1H), 7.22–7.15 (m, 1H), 7.10 (t, *J* = 7.5 Hz, 1H), 6.99 (d, *J* = 2.4 Hz, 1H), 5.67 (d, *J* = 8.4 Hz, 1H), 5.47 (d, *J* = 7.9 Hz, 1H), 5.24 (t, *J* = 9.5 Hz, 1H), 5.17–5.06 (m, 2H), 4.65 (dt, *J* = 8.0, 5.5 Hz, 1H), 4.28 (dd, *J* = 12.5, 4.2 Hz, 1H), 4.23 (dd, *J* = 12.5, 2.3 Hz, 1H), 3.83 (ddd, *J* = 10.2, 4.2, 2.3 Hz, 1H), 3.69 (s, 3H), 3.38 (dd, *J* = 14.9, 5.4 Hz, 1H), 3.28 (dd, *J* = 14.8, 5.6 Hz, 1H), 2.09 (s, 3H), 2.04 (s, 3H), 2.02 (s, 3H), 2.00 (s, 3H).

^{13}C NMR (126 MHz, $CDCl_3$) δ 171.84, 170.94, 170.26, 169.63, 169.59, 153.31, 136.21, 127.42, 123.31, 122.38, 119.82, 118.59, 111.43, 109.27, 92.98, 72.94, 72.59, 70.06, 67.93, 61.48, 54.51, 52.66, 27.61, 20.95, 20.74, 20.72.

HRMS (ESI) m/z calcd for $C_{27}H_{32}N_2O_{13}Na$ $[M+Na]^+$ 615.1797; **found**: 615.1800.

Compound GA50

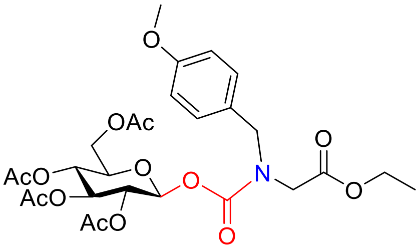

Compound **GA50** was synthesized according to the *General procedure* using amine **A18** (1.12 g, 5.0 mmol, 1.0 eq) and glycosyl halide **G2** (4.11 g, 10.0 mmol, 2.0 eq) in presence of Cs_2CO_3 (2.45 g, 7.5 mmol, 1.5 eq). After the reaction, the mixture was

purified by silica gel flash chromatography (petroleum ether/EtOAc = 2:1) to give **GA50** as a white solid (3.4 g, >99%).

$[\alpha]_D^{25}$ = 16.7 (*c* 1.0, $CHCl_3$).

^{1}H NMR (500 MHz, $CDCl_3$) (mixture of rotamers) δ 7.14–7.09 (m, 1.23H), 7.08–7.03 (m, 0.69H), 6.81–6.76 (m, 2H), 5.71–5.67 (m, 1H), 5.28–5.02 (m, 3H), 4.76 (d, *J* = 14.9 Hz, 0.64H), 4.45 (d, *J* = 15.4 Hz, 0.36H), 4.36 (d, *J* = 15.5 Hz, 0.36H), 4.31–4.24 (m, 1H), 4.15–4.01 (m, 4.68H), 3.91 (d, *J* = 17.6 Hz, 0.36H), 3.84–3.79 (m, 1H), 3.76 (d, *J* = 17.6 Hz, 0.36H), 3.73–3.71 (m, 3H), 3.48 (d, *J* = 18.2 Hz, 0.62H), 2.04–2.01 (m, 3H), 1.99–1.91 (m, 9H), 1.21–1.15 (m, 3H).

^{13}C NMR (126 MHz, $CDCl_3$) (mixture of rotamers) δ 170.54, 170.51, 169.98, 169.92, 169.52, 169.37, 169.35, 168.82, 168.79, 159.35, 159.28, 154.08, 153.89, 129.77, 129.48, 129.30, 127.86, 114.06, 113.80, 93.59, 93.26, 72.68, 72.49, 72.43, 70.14, 69.77, 67.92, 67.82, 61.43, 61.38, 61.17, 61.12, 55.21, 51.11, 50.54, 47.48, 46.77, 20.60, 20.51, 20.49, 14.08, 14.01.

HRMS (ESI) m/z calcd for $C_{27}H_{35}NO_{14}Na$ $[M+Na]^+$ 620.1950; **found**: 620.1955.

Compound GA51

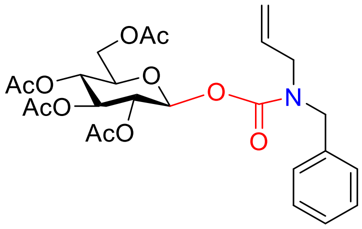

Compound **GA51** was synthesized according to the *General procedure* using amine **A19** (74 mg, 0.5 mmol, 1.0 eq) and glycosyl halide **G2** (411 mg, 1.0 mmol, 2.0 eq) in presence of Cs_2CO_3 (245 mg, 0.75 mmol, 1.5 eq). After the reaction, the mixture was purified by silica gel flash chromatography (petroleum ether/EtOAc = 2:1) to give **GA51** as a white solid (232 mg, 89%).

$[\alpha]_D^{25}$ = 10.5 (*c* 1.0, $CHCl_3$).

^{1}H NMR (500 MHz, $CDCl_3$) (mixture of rotamers) δ 7.28–7.15 (m, 4H), 7.12–7.06 (m, 1H), 5.77–5.54 (m, 2H), 5.25–5.17 (m, 1H), 5.15–4.97 (m, 4H), 4.56–4.45 (m, 1H), 4.35–4.18 (m, 2H), 4.10–4.01 (m, 1H), 3.97–3.79 (m, 2H), 3.71–3.53 (m, 1H),

2.03–1.99 (m, 3H), 1.98–1.90 (m, 9H).

^{13}C NMR (126 MHz, $CDCl_3$) (mixture of rotamers) δ 170.48, 169.94, 169.33, 169.31, 169.08, 169.06, 154.04, 153.60, 136.73, 136.70, 132.48, 132.35, 132.33, 128.55, 128.05, 127.60, 127.42, 127.31, 118.04, 116.95, 93.26, 72.75, 72.68, 72.33, 70.06, 69.99, 67.84, 61.38, 61.34, 49.92, 49.09, 48.31, 20.62, 20.50, 20.47, 20.32.

HRMS (ESI) m/z calcd for $C_{25}H_{31}NO_{11}Na$ $[M+Na]^+$ 544.1789; **found**: 544.1789.

Compound GA52

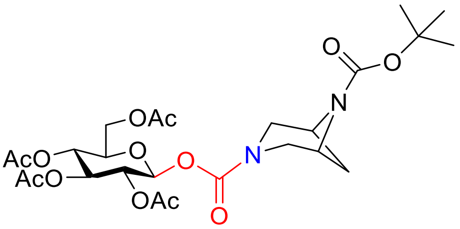

Compound **GA52** was synthesized according to the *General procedure* using amine **A20** (200 mg, 1.01 mmol, 1.0 eq) and glycosyl halide **G2** (831 mg, 2.02 mmol, 2.0 eq) in presence of Cs_2CO_3 (494 mg, 1.51 mmol, 1.5 eq). After the reaction, the mixture was purified by silica gel flash chromatography (petroleum ether/EtOAc = 4:1 to 1:1) to give **GA52** as a white solid (428 mg, 74%).

$[\alpha]_D^{25}$ = -6.5 (*c* 0.1, CH_2Cl_2).

^{1}H NMR (600 MHz, $CDCl_3$) (mixture of rotamers) δ 5.70–5.59 (m, 1H), 5.27 (td, *J* = 9.4, 4.3 Hz, 1H), 5.19 (dd, *J* = 9.7, 8.4 Hz, 1H), 5.16–5.10 (m, 1H), 4.33–4.23 (m, 1H), 4.19–3.96 (m, 4H), 3.84 (dddd, *J* = 14.3, 10.0, 4.3, 2.2 Hz, 1H), 3.51–3.26 (m, 2H), 2.61–2.55 (m, 1H), 2.09–2.07 (m, 3H), 2.06–2.04 (m, 2H), 2.03–2.00 (m, 9H), 1.42 (s, 3H), 1.41 (s, 6H).

^{13}C NMR (151 MHz, $CDCl_3$) (mixture of rotamers) δ 170.76, 170.22, 170.20, 169.58, 169.48, 156.12, 154.27, 93.26, 93.21, 80.80, 80.51, 72.81, 72.78, 72.66, 72.47, 70.38, 70.03, 68.08, 68.01, 61.64, 61.58, 58.10, 57.58, 45.84, 28.58, 28.40, 28.36, 20.88, 20.83, 20.81, 20.73, 20.71, 20.64.

HRMS (ESI) m/z calcd for $C_{25}H_{36}N_2O_{13}Na$ $[M+Na]^+$ 595.2112; **found**: 595.2110.

Compound GA53

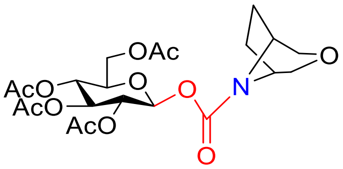

Compound **GA53** was synthesized according to the *General procedure* using amine **A21** (75 mg, 0.5 mmol, 1.0 eq) and glycosyl halide **G2** (411 mg, 1.0 mmol, 2.0 eq) in presence of Cs_2CO_3 (244 mg, 0.75 mmol, 1.5 eq). After the reaction, the mixture was purified by silica gel flash chromatography (dichloromethane/MeOH = 100:1) to give **GA53** (170 mg, 70%) as a white solid.

$[\alpha]_D^{25}$ = -11.4 (*c* 0.1, CH_2Cl_2).

^{1}H NMR (600 MHz, $CDCl_3$) (mixture of rotamers) δ 5.69–5.62 (m, 1H), 5.31–5.24 (m, 1H), 5.19–5.10 (m, 2H), 4.35–4.29 (m, 1H), 4.24–4.07 (m, 3H), 3.88–3.82 (m, 1H), 3.74 (d, *J* = 10.9 Hz, 0.42H), 3.71–3.66 (m, 1H), 3.62–3.54 (m, 2H), 3.42 (d, *J* = 11.0 Hz, 0.55H), 2.10–2.07 (m, 3H), 2.06–1.96 (m, 12H), 1.93–1.77 (m, 1H).

^{13}C NMR (151 MHz, $CDCl_3$) (mixture of rotamers) δ 170.77, 170.19, 169.60, 169.49, 169.39, 151.18, 150.59, 93.28, 93.19, 72.71, 72.64, 72.24, 72.15, 71.83, 70.34, 70.30, 68.13, 68.05, 61.63, 61.58, 56.02, 55.99, 55.41, 27.48, 27.34, 26.72, 26.68, 20.88, 20.76, 20.74, 20.72.

HRMS (ESI) m/z calcd for $C_{21}H_{29}NO_{12}Na$ $[M+Na]^+$ 510.1580; **found**: 510.1582.

Compound GA54

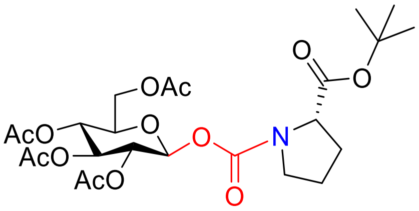

Compound **GA54** was synthesized according to the *General procedure* using amine **A22** (200 mg, 1.16 mmol, 1.0 eq) and glycosyl halide **G2** (954 mg, 2.32 mmol, 2.0 eq) in presence of Cs_2CO_3 (567 mg, 1.74 mmol, 1.5 eq). After the reaction, the mixture was purified by silica gel flash chromatography (petroleum ether/EtOAc = 4:1 to 2:1) to give **GA54** as a white solid (542 mg, 86%).

$[\alpha]_D^{25}$ = -25.4 (*c* 0.1, CH_2Cl_2).

^{1}**H NMR** (600 MHz, $CDCl_3$) (mixture of rotamers) δ 5.66–5.62 (m, 1H), 5.27–5.21 (m, 1H), 5.20–5.15 (m, 0.65H), 5.13–5.09 (m, 0.67H), 5.08–5.03 (m, 0.69H), 4.31–4.19 (m, 1.73H), 4.13–4.04 (m, 1.38H), 3.84–3.78 (m, 1H), 3.62–3.56 (m, 0.33H), 3.52–3.37 (m, 1.67H), 2.21–2.10 (m, 1H), 2.09–2.04 (m, 4H), 2.04–1.98 (m, 9H), 1.98–1.82 (m, 2H), 1.46–1.41 (m, 9H).

^{13}**C NMR** (151 MHz, $CDCl_3$) (mixture of rotamers) δ 171.22, 171.15, 170.77, 170.69, 170.20, 170.17, 169.73, 169.59, 169.34, 152.24, 151.98, 93.23, 92.97, 81.76, 81.53, 72.94, 72.75, 72.63, 72.32, 70.45, 70.23, 68.08, 68.03, 61.64, 60.04, 59.79, 47.12, 46.75, 30.89, 29.96, 28.09, 27.92, 24.12, 23.37, 20.86, 20.78, 20.75, 20.72, 20.70.

HRMS (ESI) m/z calcd for $C_{24}H_{35}NO_{13}Na$ $[M+Na]^+$ 568.1997; **found**: 568.2001.

Compound GA55

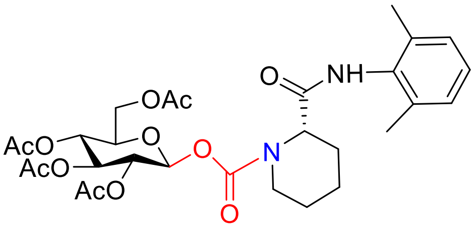

Compound **GA55** was synthesized according to the *General procedure* using amine **A23** (200 mg, 0.86 mmol, 1.0 eq) and glycosyl halide **G2** (707 mg, 1.72 mmol, 2.0 eq) in presence of Cs_2CO_3 (420 mg, 1.29 mmol, 1.5 eq). After the reaction, the mixture was purified by silica gel flash chromatography (petroleum ether/EtOAc = 3:1 to 1:1) to give **GA55** as a white solid (294 mg, 56%).

$[\alpha]_D^{25}$ = -47.8 (*c* 0.1, CH_2Cl_2).

^{1}**H NMR** (600 MHz, $CDCl_3$) (mixture of rotamers) δ 7.34 (bs, 0.53H), 7.13–7.01 (m, 3H), 5.69 (d, *J* = 8.4 Hz, 0.41H), 5.66 (d, *J* = 8.2 Hz, 0.58H), 5.33–5.24 (m, 1H), 5.22–5.13 (m, 1.62H), 5.08 (t, *J* = 9.8 Hz, 0.44H), 4.92 (d, *J* = 4.3 Hz, 0.56H), 4.80 (d, *J* = 5.8 Hz, 0.41H), 4.33 (dd, *J* = 12.5, 4.3 Hz, 0.55H), 4.29–3.97 (m, 2.47H), 3.91–3.78 (m, 1H), 3.21–3.02 (m, 1H), 2.49–2.28 (m, 1H), 2.23 (s, 2.85H), 2.20 (s, 3.50H), 2.10 (s, 1.68H), 2.08 (s, 1.42H), 2.05–2.00 (m, 6.51H), 1.95 (s, 1.31H), 1.93 (s, 1.86H), 1.82–1.66 (m, 3.65H), 1.59–1.45 (m, 1.54H).

^{13}**C NMR** (151 MHz, $CDCl_3$) (mixture of rotamers) δ 170.75, 170.60, 170.15, 169.75,

169.71, 169.57, 169.55, 169.09, 168.53, 154.76, 153.00, 135.46, 135.19, 133.61, 133.57, 128.40, 128.36, 127.61, 127.49, 94.01, 93.76, 72.76, 72.62, 72.60, 72.46, 70.52, 70.31, 67.97, 67.95, 61.58, 61.39, 55.33, 54.73, 42.66, 42.61, 26.22, 26.01, 24.79, 20.89, 20.79, 20.71, 20.69, 20.62, 20.29, 20.16, 18.68.

HRMS (ESI) m/z calcd for $C_{29}H_{38}N_2O_{12}Na$ $[M+Na]^+$ 629.2325; **found**: 629.2317.

Compound GA56

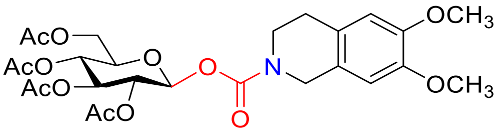

Compound **GA56** was synthesized according to the *General procedure* using amine **A24** (200 mg, 1.03 mmol, 1.0 eq) and glycosyl halide **G2** (468 mg, 1.14 mmol, 1.1 eq) in presence of Cs_2CO_3 (509 mg, 1.55 mmol, 1.5 eq). After the reaction, the mixture was purified by silica gel flash chromatography (petroleum ether/EtOAc = 1:1) to give **GA56** as a white solid (370 mg, 63%).

$[\alpha]_D^{25}$ = -24.8 (*c* 0.1, $CHCl_3$).

^{1}H NMR (500 MHz, $CDCl_3$) (mixture of rotamers) δ 6.63-6.56 (m, 2H), 5.71-5.64 (m, 1H), 5.31-5.25 (m, 1H), 5.24-5.18 (m, 1H), 5.14 (t, *J* = 9.6 Hz, 1H), 4.62-4.40 (m, 2H), 4.34-4.28 (m, 1H), 4.14-4.07 (m, 1H), 3.90-3.80 (m, 7H), 3.74-3.55 (m, 2H), 2.82-2.62 (m, 2H), 2.08 (s, 3H), 2.03 (s, 3H), 2.02-2.00 (m, 6H).

^{13}C NMR (126 MHz, $CDCl_3$) (mixture of rotamers) δ 170.77, 170.19, 169.60, 169.56, 169.54, 153.04, 152.88, 147.93, 147.89, 126.08, 126.02, 124.62, 124.40, 111.50, 109.16, 109.13, 93.37, 72.82, 72.77, 72.60, 72.57, 70.26, 70.24, 68.06, 68.01, 61.60, 61.56, 56.13, 56.11, 56.08, 56.07, 45.71, 45.52, 42.05, 41.98, 28.45, 28.01, 20.87, 20.78, 20.73, 20.72, 20.69.

HRMS (ESI) m/z calcd for $C_{26}H_{33}NO_{13}Na$ $[M+Na]^+$ 590.1848; **found**: 590.1844.

Compound A57

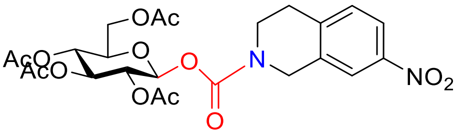

Compound **GA57** was synthesized according to the *General procedure* using amine **A25** (200 mg, 1.12 mmol, 1.0 eq) and glycosyl halide **G2** (921 mg, 2.24 mmol, 2.0 eq) in presence of Cs_2CO_3 (537 mg, 1.68 mmol, 1.5 eq). After the reaction, the mixture was purified by silica gel flash chromatography (petroleum ether/EtOAc = 4:1 to 2:1 to 1:1) to give **GA57** (528 mg, 85%) as a yellow solid.

$[\alpha]_D^{25}$ = -30.3 (*c* 0.1, CH_2Cl_2).

^{1}H NMR (600 MHz, $CDCl_3$) (mixture of rotamers) δ 8.09–8.00 (m, 2H), 7.31 (d, *J* = 8.3 Hz, 1H), 5.69–5.65 (m, 1H), 5.29 (t, *J* = 9.5 Hz, 1H), 5.24–5.19 (m, 1H), 5.17–5.11 (m, 1H), 4.77–4.67 (m, 1.58H), 4.62 (d, *J* = 17.0 Hz, 0.45H), 4.33–4.28 (m, 1H), 4.14–4.09 (m, 1H), 3.89–3.83 (m, 1H), 3.81–3.66 (m, 2H), 3.02–2.94 (m, 1.51H), 2.90–2.83 (m, 0.49H), 2.08 (s, 3.18H), 2.04–2.02 (m, 7.43H), 1.96 (s, 1.35H).

^{13}C NMR (126 MHz, $CDCl_3$) (mixture of rotamers) δ 170.76, 170.74, 170.21, 170.16, 169.62, 169.57, 169.45, 152.88, 152.80, 146.81, 141.92, 141.90, 134.45, 134.28, 129.95, 129.92, 121.94, 121.78, 121.76, 93.63, 93.59, 72.70, 72.68, 72.65, 70.31, 70.28, 68.03, 67.99, 61.58, 61.53, 45.80, 45.65, 41.36, 41.20, 29.19, 28.82, 20.88, 20.80, 20.74, 20.73.

HRMS (ESI) m/z calcd for $C_{24}H_{28}N_2O_{13}Na$ $[M+Na]^+$ 575.1487; **found**: 575.1484.

Compound GA58

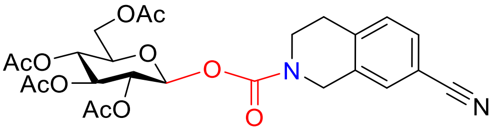

Compound **GA58** was synthesized according to the *General procedure* using amine **A26** (158 mg, 1.0 mmol, 1.0 eq) and glycosyl halide **G2** (822 mg, 2.0 mmol, 2.0 eq) in presence of Cs_2CO_3 (490 mg, 1.5 mmol, 1.5 eq). After the reaction, the mixture was purified by silica gel flash chromatography (petroleum ether/EtOAc = 1.8:1) to give **GA58** (2812 mg, 53%) as a yellow solid.

$[\alpha]_D^{25}$ = -21.0 (*c* 1.0, $CHCl_3$).

^{1}H NMR (500 MHz, $CDCl_3$) (mixture of rotamers) δ 7.42–7.33 (m, 2H), 7.22–7.17 (m, 1H), 5.65–5.59 (m, 1H), 5.27–5.20 (m, 1H), 5.16–5.10 (m, 1H), 5.09–5.03 (m,

1H), 4.65–4.45 (m, 2H), 4.27–4.20 (m, 1H), 4.07–4.00 (m, 1H), 3.86–3.79 (m, 1H), 3.68–3.58 (m, 2H), 2.92–2.71 (m, 2H), 2.02–1.97 (m, 3H), 1.97–1.92 (m, 7.74H), 1.87 (s, 1.41H).

^{13}C NMR (126 MHz, $CDCl_3$) (mixture of rotamers) δ 170.45, 169.91, 169.88, 169.36, 169.33, 169.30, 169.22, 152.61, 152.57, 139.78, 139.77, 134.18, 134.00, 130.18, 130.14, 130.03, 130.01, 129.66, 129.59, 118.46, 118.42, 110.33, 93.29, 93.26, 72.36, 72.31, 70.04, 70.03, 67.80, 67.77, 61.35, 61.31, 45.22, 45.10, 41.13, 41.00, 28.93, 28.54, 20.62, 20.55, 20.50, 20.49, 20.46.

HRMS (ESI) m/z calcd for $C_{25}H_{28}N_2O_{11}Na$ $[M+Na]^+$ 555.1585; **found**: 555.1590.

Compound GA59

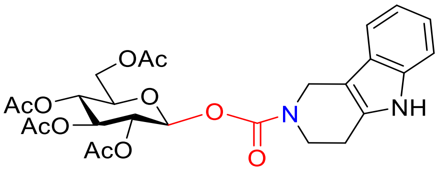

Compound **GA59** was synthesized according to the *General procedure* using amine **A27** (86 mg, 0.5 mmol, 1.0 eq) and glycosyl halide **G2** (226 mg, 0.55 mmol, 1.1 eq) in presence of Cs_2CO_3 (245 mg, 0.75 mmol, 1.5 eq). After the reaction, the mixture was purified by silica gel flash chromatography (petroleum ether/EtOAc = 3:2 to 1:1) to give **GA59** as a brown liquid (206 mg, 75%).

$[\alpha]_D^{25}$ = -33.7 (*c* 1.0, $CHCl_3$).

^{1}H NMR (500 MHz, $CDCl_3$) (mixture of rotamers) δ 7.97 (s, 1H), 7.49–7.41 (m, 1H), 7.35–7.28 (m, 1H), 7.19–7.13 (m, 1H), 7.13–7.07 (m, 1H), 5.73–5.66 (m, 1H), 5.35–5.20 (m, 2H), 5.19–5.11 (m, 1H), 4.78–4.57 (m, 2H), 4.35–4.28 (m, 1H), 4.19–4.05 (m, 1H), 3.99–3.71 (m, 3H), 2.95–2.68 (m, 2H), 2.10–1.98 (m, 12H).

^{13}C NMR (126 MHz, $CDCl_3$) (mixture of rotamers) δ 170.86, 170.82, 170.28, 170.19, 169.66, 169.58, 169.45, 153.57, 152.96, 135.90, 135.88, 131.42, 131.28, 125.49, 125.37, 121.98, 119.89, 119.88, 117.90, 117.82, 110.87, 110.82, 106.97, 106.88, 93.44, 93.41, 72.79, 72.67, 72.52, 72.51, 70.26, 70.12, 68.00, 67.90, 61.56, 61.47, 60.55, 41.89, 41.80, 41.75, 41.62, 23.69, 23.18, 21.21, 20.88, 20.82, 20.75, 20.73, 20.53, 14.31.

HRMS (ESI) m/z calcd for $C_{26}H_{30}N_2O_{11}Na$ $[M+Na]^+$ 569.1742; **found**: 569.1750.

Compound GA60

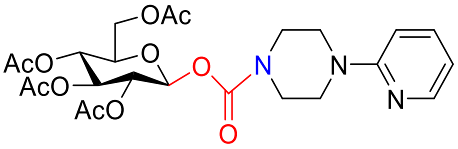

Compound **GA60** was synthesized according to the *General procedure* using amine **A28** (215 mg, 1.30 mmol, 1.0 eq) and glycosyl halide **G2** (802 mg, 1.95 mmol, 1.5 eq in presence of Cs_2CO_3 (635 mg, 1.95 mmol, 1.5 eq). After the reaction, the mixture was purified by silica gel flash chromatography (petroleum ether/EtOAc = 4:1 to 3:1 to 1:1) to give **GA60** as a white solid (266 mg, 38%).

$[\alpha]_D^{25}$ = -37.1 (*c* 0.1, CH_2Cl_2).

^{1}**H NMR** (600 MHz, $CDCl_3$) (mixture of rotamers) δ 8.19 (ddd, *J* = 4.9, 2.0, 0.9 Hz, 1H), 7.50 (ddd, *J* = 8.9, 7.2, 2.0 Hz, 1H), 6.75–6.58 (m, 2H), 5.65 (d, *J* = 8.3 Hz, 1H), 5.28 (t, *J* = 9.5 Hz, 1H), 5.19 (dd, *J* = 9.7, 8.3 Hz, 1H), 5.14 (dd, *J* = 10.1, 9.3 Hz, 1H), 4.32 (dd, *J* = 12.5, 4.3 Hz, 1H), 4.12 (dd, *J* = 12.6, 2.2 Hz, 1H), 3.86 (ddd, *J* = 10.1, 4.3, 2.2 Hz, 1H), 3.72–3.50 (m, 7H), 3.42 (dd, *J* = 12.5, 6.7 Hz, 1H), 2.08 (s, 3H), 2.04–2.03 (m, 6H), 2.02 (s, 3H).

^{13}**C NMR** (151 MHz, $CDCl_3$) δ 170.77, 170.19, 169.59, 169.48, 159.06, 152.80, 148.07, 137.90, 114.11, 107.46, 93.51, 72.70, 72.64, 70.26, 68.05, 61.58, 45.13, 44.99, 43.89, 43.65, 20.88, 20.76, 20.73, 20.72.

HRMS (ESI) m/z calcd for $C_{24}H_{31}N_3O_{11}Na$ $[M+Na]^+$ 560.1858; **found**: 560.1851.

Compound GA61

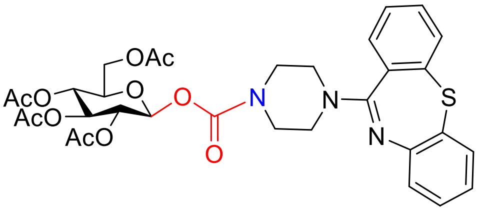

Compound **GA61** was synthesized according to the *General procedure* using amine **A29** (100 mg, 0.34 mmol, 1.0 eq) and glycosyl halide **G2** (279 mg, 0.68 mmol, 2.0 eq)

in presence of Cs_2CO_3 (266 mg, 0.51 mmol, 1.5 eq). After the reaction, the mixture was purified by silica gel flash chromatography (petroleum ether/EtOAc = 4:1 to 3:1 to 1:1), then added methyl alcohol overnight, filter to give **GA61** as a white solid (152 mg, 67%).

$[\alpha]_D^{25}$ = -7.6 (*c* 0.1, CH_2Cl_2).

^{1}H NMR (600 MHz, $CDCl_3$) (mixture of rotamers) δ 7.54–7.49 (m, 1H), 7.41–7.37 (m, 1H), 7.37–7.33 (m, 1H), 7.33–7.29 (m, 2H), 7.21–7.15 (m, 1H), 7.09–7.05 (m 1H), 6.93–6.88 (m, 1H), 5.69–5.58 (m, 1H), 5.28 (t, *J* = 9.5 Hz, 0.63H), 5.20–5.04 (m 2H), 4.34–4.28 (m, 1H), 4.15–4.07 (m, 1H), 3.89–3.80 (m, 1H), 3.76–3.35 (m, 8H), 2.11–2.06 (m, 4H), 2.05–2.00 (m, 8H).

^{13}C NMR (126 MHz, $CDCl_3$) (mixture of rotamers) δ 172.07, 171.39, 171.35, 171.30, 170.80, 170.19, 169.76, 169.61, 169.54, 169.50, 160.82, 160.79, 160.76, 160.73, 153.37, 152.84, 152.82, 148.61, 140.21, 140.18, 134.00, 133.96, 132.46, 132.40, 132.38, 131.27, 131.25, 129.34, 128.95, 128.66, 128.63, 128.09, 128.07, 128.02, 125.39, 123.46, 123.43, 95.71, 93.53, 75.67, 75.63, 75.01, 74.93, 72.67, 72.63, 71.82, 71.80, 70.23, 70.18, 68.04, 67.76, 62.61, 62.06, 61.77, 61.57, 44.02, 43.71, 21.02, 20.96, 20.93, 20.90, 20.83, 20.79, 20.77, 20.73.

HRMS (ESI) m/z calcd for $C_{32}H_{36}N_3O_{11}S$ $[M+H]^+$ 670.2069; **found**: 670.2065.

Compound GA62

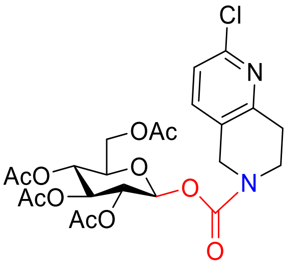

Compound **GA62** was synthesized according to the *General procedure* using amine **A30** (200 mg, 0.98 mmol, 1.0 eq) and glycosyl halide **G2** (806 mg, 1.96 mmol, 2.0 eq) in presence of Cs_2CO_3 (479 mg, 1.47 mmol, 1.5 eq). After the reaction, the mixture was purified by silica gel flash chromatography (petroleum ether/EtOAc = 4:1 to 3:1 to 2:1 to 1:1) to give **GA62** (454 mg, 85%) as a yellow solid.

$[\alpha]_D^{25}$ = -25.2 (*c* 0.1, CH_2Cl_2).

^{1}H NMR (600 MHz, $CDCl_3$) (mixture of rotamers) δ 7.42–7.36 (m, 1H), 7.21–7.16 (m, 1H), 5.67 (d, *J* = 8.2 Hz, 0.41H), 5.65 (d, *J* = 8.2 Hz, 0.54H), 5.31–5.25 (m, 1H), 5.24–5.17 (m, 1H), 5.17–5.10 (m, 1H), 4.70–4.49 (m, 2H), 4.36–4.26 (m, 1H), 4.16–4.06 (m, 1H), 3.89–3.83 (m, 1H), 3.83–3.68 (m, 2H), 3.15–2.84 (m, 2H), 2.09–2.06 (m, 3.21H), 2.04–2.01 (m, 7.63H), 1.94 (s, 1.29H).

^{13}C NMR (151 MHz, $CDCl_3$) (mixture of rotamers) δ 170.77, 170.73, 170.21, 170.14, 169.62, 169.58, 169.48, 155.10, 155.03, 152.86, 152.78, 149.52, 149.47, 137.07, 137.02, 127.08, 126.85, 122.41, 122.38, 93.64, 93.59, 72.71, 72.66, 70.31, 70.20, 68.01, 61.54, 44.80, 44.64, 41.56, 41.40, 31.87, 31.48, 20.87, 20.76, 20.74, 20.72.

HRMS (ESI) m/z calcd for $C_{23}H_{27}ClN_2O_{11}Na$ $[M+Na]^+$ 565.1179; **found**: 565.1196.

Compound GA63

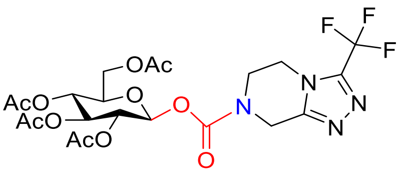

Compound **GA63** was synthesized according to the *General procedure* using amine **A31** (200 mg, 0.87 mmol, 1.0 eq) and glycosyl halide **G2** (716 mg, 1.74 mmol, 2.0 eq) in presence of Cs_2CO_3 (425 mg, 1.31 mmol, 1.5 eq). After the reaction, the mixture was purified by silica gel flash chromatography (petroleum ether/EtOAc = 4:1 to 2:1 to 1:1) to give **GA63** as a white solid (479 mg, 97%).

$[\alpha]_D^{25}$ = -13.7 (*c* 0.1, CH_2Cl_2).

^{1}H NMR (600 MHz, $CDCl_3$) (mixture of rotamers) δ 5.64 (d, *J* = 8.3 Hz, 1H), 5.33–5.24(m, 1H), 5.21–5.08 (m, 2H), 5.04–4.79 (m, 2H), 4.33–4.24 (m, 1H), 4.24–3.80 (m, 6H), 2.09–2.05 (m, 3H), 2.04–2.00 (m, 9H).

^{13}C NMR (151 MHz, $CDCl_3$) (mixture of rotamers) δ 170.72, 170.64, 170.19, 170.03, 169.74, 169.60, 169.47, 169.27, 152.57, 152.13, 149.73, 149.56, 143.71 (q, J = 40.1 Hz), 118.24 (q, J = 270.5 Hz), 94.08, 72.79, 72.76, 72.48, 72.28, 70.33, 70.15, 67.88, 67.79, 61.44, 61.36, 43.51, 43.17, 41.47, 41.36, 40.70, 40.36, 20.82, 20.80, 20.71, 20.67.

HRMS (ESI) m/z calcd for $C_{21}H_{25}F_3N_4O_{11}Na$ $[M+Na]^+$ 589.1364; **found**: 589.1364.

Compound GA64

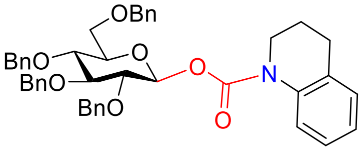

Compound **GA64** was synthesized according to the *General procedure* using amine **A32** (133 mg, 1.0 mmol, 1.0 eq) and glycosyl halide **G4** (1680 mg, 3.0 mmol, 3.0 eq) in presence of Cs_2CO_3 (490 mg, 1.5 mmol, 1.5 eq). After the reaction, the mixture was purified by silica gel flash chromatography (petroleum ether/EtOAc = 7:1) to give **GA64** as a white solid (384 mg, 55%).

$[\alpha]_D^{25}$ = -37.1 (*c* 1.0, $CHCl_3$).

^{1}H NMR (500 MHz, $CDCl_3$) (mixture of rotamers) δ 7.44–7.00 (m, 24H), 5.72 (d, *J* = 8.1 Hz, 1H), 4.94 (d, *J* = 11.0 Hz, 1H), 4.91–4.83 (m, 5H), 4.82–4.49 (m, 1H), 4.03–3.92 (m, 2H), 3.89–3.75 (m, 4H), 3.73–3.53 (m, 3H), 2.82–2.70 (m, 2H), 2.02–1.85 (m, 2H).

^{13}C NMR (126 MHz, $CDCl_3$) (mixture of rotamers) δ 153.04, 138.54, 138.15, 138.13, 138.04, 137.79, 128.64, 128.49, 128.46, 128.42, 128.08, 127.99, 127.88, 127.86, 127.78, 127.76, 127.74, 127.72, 126.07, 124.25, 95.90, 84.96, 81.10, 77.38, 75.70, 75.44, 75.06, 74.88, 73.60, 68.17, 44.92, 27.20, 23.49.

HRMS (ESI) m/z calcd for $C_{44}H_{45}NO_7Na$ $[M+Na]^+$ 722.3088; **found**: 722.3094.

Compound GA65

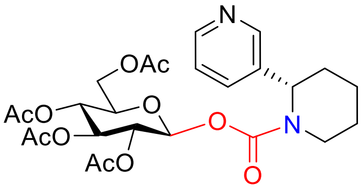

Compound **GA65** was synthesized according to the *General procedure* using amine **A43** (81 mg, 0.5 mmol, 1.0 eq) and glycosyl halide **G2** (411 mg, 1.0 mmol, 2.0 eq) in presence of Cs_2CO_3 (245 mg, 0.75 mmol, 1.5 eq). After the reaction, the mixture was purified by silica gel flash chromatography (petroleum ether/EtOAc = 1:2) to give **GA65** as a white solid (267 mg, 50%).

$[\alpha]_D^{25}$ = -59.9 (*c* 1.0, CH_2Cl_2).

^{1}H NMR (500 MHz, $CDCl_3$) δ 8.53–8.36 (m, 2H), 7.64–7.40 (m, 1H), 7.30–7.20 (m, 1H), 5.69 (d, *J* = 8.3 Hz, 1H), 5.55–5.33 (m, 1H), 5.25 (t, *J* = 9.5 Hz, 1H), 5.20–4.99 (m, 2H), 4.34–4.18 (m, 1H), 4.14–3.95 (m, 2H), 3.82 (d, *J* = 10.1 Hz, 1H), 2.72 (t, *J* = 13.2 Hz, 1H), 2.37–2.23 (m, 1H), 2.08–1.94 (m, 13H), 1.71–1.36 (m, 4H).

^{13}C NMR (126 MHz, $CDCl_3$) δ 170.65, 170.07, 169.47, 169.43, 153.58, 148.34, 148.27, 134.56, 123.54, 93.58, 72.62, 72.53, 70.32, 67.99, 61.49, 52.24, 40.71, 27.52, 25.15, 20.78, 20.67, 20.63, 20.61, 19.08.

HRMS (ESI) m/z calcd for $C_{25}H_{33}N_2O_{11}Na$ $[M+Na]^+$ 537.2079; **found**: 537.2081.

Compound GA66

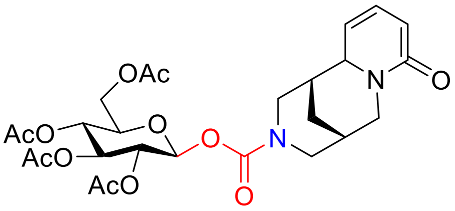

Compound **GA66** was synthesized according to the *General procedure* using amine **A44** (95 mg, 0.5 mmol, 1.0 eq) and glycosyl halide **G2** (226 mg, 0.55 mmol, 1.1 eq) in presence of Cs_2CO_3 (245 mg, 0.75 mmol, 1.5 eq). After the reaction, the mixture was purified by silica gel flash chromatography (petroleum ether/EtOAc = 1:1 to 1:4) to give **GA66** as a yellow solid (143 mg, 51%).

$[\alpha]_D^{25}$ = -56.2 (*c* 1.0, $CHCl_3$).

^{1}H NMR (500 MHz, $CDCl_3$) (mixture of rotamers) δ 7.32–7.17 (m, 1H), 6.43–6.37 (m, 1H), 6.08–6.02 (m, 1H), 5.45–5.39 (m, 1H), 5.22 (t, *J* = 9.5 Hz, 0.77H), 5.19–5.10 (m, 1H), 5.07 (t, *J* = 9.7 Hz, 0.76H), 5.01–4.95 (m, 0.42H), 4.31 (d, *J* = 13.3 Hz, 0.21H), 4.26 (dd, *J* = 12.5, 4.2 Hz, 0.79H), 4.22–4.13 (m, 1.88H), 4.13–4.03 (m, 1.49H), 3.95 (dd, *J* = 12.3, 2.2 Hz, 0.22H), 3.91–3.79 (m, 1.79H), 3.75 (ddd, *J* = 10.1, 4.3, 2.2 Hz, 0.78H), 3.72–3.66 (m, 0.21H), 3.18–2.99 (m, 3H), 2.53–2.44 (m, 1H), 2.16 (s, 2.36H), 2.05 (s, 2.45H), 2.02–1.96 (m, 8H), 1.95–1.90 (m, 1H).

^{13}C NMR (126 MHz, $CDCl_3$) (mixture of rotamers) δ 170.73, 170.37, 170.05, 169.60, 163.29, 152.79, 148.44, 139.07, 117.78, 117.60, 105.83, 105.64, 93.43, 93.07, 72.73,

72.46, 72.44, 72.31, 70.10, 69.68, 67.97, 61.77, 61.46, 51.33, 51.11, 50.58, 50.35, 48.84, 41.10, 34.42, 34.36, 27.16, 25.95, 25.81, 20.85, 20.80, 20.74, 20.69.

HRMS (ESI) m/z calcd for $C_{26}H_{32}N_2O_{12}Na$ $[M+Na]^+$ 587.1847; **found**: 587.1842.

Compound GA67

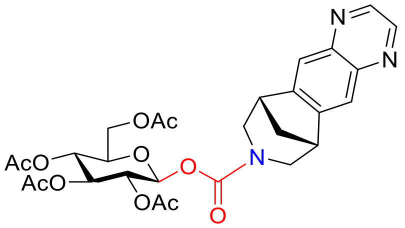

Compound **GA67** was synthesized according to the *General procedure* using amine **A45** (21 mg, 0.1 mmol, 1.0 eq) and glycosyl halide **G2** (123 mg, 0.3 mmol, 3.0 eq) in presence of K_2CO_3 (21 mg, 0.15 mmol, 1.5 eq). After the reaction, the mixture was purified by silica gel flash chromatography (petroleum ether/EtOAc = 1:1 to 1:4) to give **GA67** as a yellow liquid (51 mg, 87%).

$[\alpha]_D^{25}$ = -15.4 (*c* 1.0, $CHCl_3$).

^{1}H NMR (500 MHz, $CDCl_3$) (mixture of rotamers) δ 8.80–8.62 (m, 2H), 7.93–7.70 (m, 2H), 5.35 (d, *J* = 8.2 Hz, 0.31H), 5.27 (d, *J* = 8.1 Hz, 0.68H), 5.16–4.91 (m, 2.72H), 4.23 (dd, *J* = 12.5, 4.2 Hz, 0.73H), 4.13–3.89 (m, 3.33H), 3.75–3.64 (m, 1H), 3.58 (d, *J* = 10.3 Hz, 0.30H), 3.52–3.37 (m, 2.44H), 3.36–3.22 (m, 1H), 2.46–2.36 (m, 1H), 2.15–1.84 (m, 12H).

^{13}C NMR (126 MHz, $CDCl_3$) (mixture of rotamers) δ 170.64, 170.59, 170.07, 169.98, 169.48, 169.43, 169.35, 169.27, 154.13, 153.65, 148.43, 148.21, 147.97, 147.84, 144.15, 144.03, 143.46, 143.44, 143.41, 143.31, 122.75, 122.67, 122.58, 122.08, 93.19, 92.99, 72.63, 72.40, 72.20, 70.14, 69.74, 67.94, 67.91, 61.46, 61.32, 50.05, 49.94, 49.68, 41.09, 40.86, 39.76, 39.71, 39.69, 39.62, 20.78, 20.72, 20.63, 20.59, 20.37.

HRMS (ESI) m/z calcd for $C_{28}H_{32}N_3O_{11}$ $[M+H]^+$ 586.2031; **found**: 586.2035.

Compound GA68

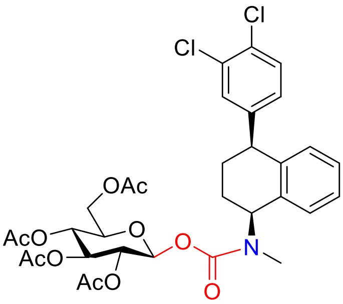

Compound **GA68** was synthesized according to the *General procedure* using amine **A46** (171 mg, 0.5 mmol, 1.0 eq) and glycosyl halide **G2** (411 mg, 1.0 mmol, 2.0 eq) in presence of Cs_2CO_3 (245 mg, 0.75 mmol, 1.5 eq). After the reaction, the mixture was purified by silica gel flash chromatography (petroleum ether/EtOAc = 2.4:4) to give **GA68** as a white solid (355 mg, 99%).

$[\alpha]_D^{25}$ = 17.2 (*c* 1.0, $CHCl_3$).

^{1}H NMR (500 MHz, $CDCl_3$) (mixture of rotamers) δ 7.32 (d, *J* = 8.3 Hz, 1H), 7.31–7.16 (m, 2.83H), 7.10–7.02 (m, 1.57H), 6.98–6.93 (m, 1H), 6.81–6.76 (m, 1H), 5.79 (d, *J* = 8.2 Hz, 0.56H), 5.71 (d, *J* = 8.3 Hz, 0.43H), 5.45 (dt, *J* = 10.3, 5.8 Hz, 0.58H), 5.39–5.20 (m, 2H), 5.20–5.09 (m, 1.45H), 4.36–4.29 (m, 1H), 4.24–4.07 (m, 2H), 3.91–3.85 (m, 1H), 2.70 (s, 1.26H), 2.66 (s, 1.68H), 2.35–2.23 (m, 1H), 2.11 (s, 1.75H), 2.08 (s, 3.13H), 2.08–1.95 (m, 9.52H), 1.82–1.66 (m, 2.41H), 1.55 (dq, *J* = 11.3, 3.2 Hz, 0.50H).

^{13}C NMR (126 MHz, $CDCl_3$) (mixture of rotamers) δ 170.80, 170.77, 170.21, 170.19, 169.58, 169.44, 169.33, 154.85, 154.50, 146.95, 146.90, 138.46, 138.05, 135.65, 135.37, 132.50, 131.11, 130.94, 130.72, 130.35, 130.27, 128.09, 127.81, 127.68, 127.51, 126.86, 93.65, 93.56, 72.73, 72.55, 70.57, 70.27, 68.10, 68.01, 61.58, 55.56, 43.09, 30.05, 29.97, 22.60, 21.74, 20.87, 20.74, 20.71.

HRMS (ESI) m/z calcd for $C_{32}H_{35}NO_{11}Cl_2Na$ $[M+Na]^+$ 702.1479; **found**: 702.1482.

Compound GA69

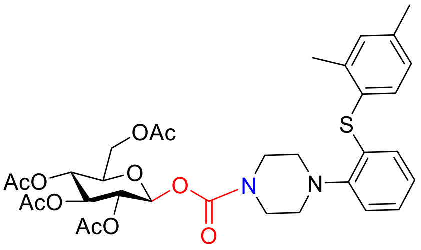

Compound **GA69** was synthesized according to the *General procedure* using amine **A47** (60 mg, 0.2 mmol, 1.0 eq) and glycosyl halide **G2** (165 mg, 0.4 mmol, 2.0 eq) in presence of Cs_2CO_3 (98 mg, 0.3 mmol, 1.5 eq). After the reaction, the mixture was purified by silica gel flash chromatography (petroleum ether/EtOAc = 4:1) to give **GA69** (132 mg, 98%) as a white solid.

$[\alpha]_D^{25}$ = 0.6 (*c* 0.1, CH_2Cl_2).

^{1}H NMR (600 MHz, $CDCl_3$) (mixture of rotamers) δ 7.35 (d, *J* = 7.8 Hz, 1H), 7.17–7.14 (m, 1H), 7.07 (td, *J* = 7.5, 1.5 Hz, 1H), 7.05–6.98 (m, 2H), 6.89 (td, *J* = 7.6, 1.3 Hz, 1H), 6.53 (dd, *J* = 7.9, 1.4 Hz, 1H), 5.66 (d, *J* = 8.3 Hz, 1H), 5.29 (t, *J* = 9.5 Hz, 1H), 5.20 (dd, *J* = 9.7, 8.3 Hz, 1H), 5.15 (t, *J* = 9.7 Hz, 1H), 4.34 (dd, *J* = 12.6, 4.2 Hz, 1H), 4.14 (dd, *J* = 12.6, 2.2 Hz, 1H), 3.87 (ddd, *J* = 10.1, 4.2, 2.2 Hz, 1H), 3.74–3.62 (m, 3H), 3.62–3.54 (m, 1H), 3.10–3.00 (m, 3H), 2.99–2.92 (m, 1H), 2.36 (s, 3H), 2.31 (s, 3H), 2.10 (s, 3H), 2.06 (s, 3H), 2.04 (s, 3H), 2.03 (s, 3H).

^{13}C NMR (151 MHz, $CDCl_3$) (mixture of rotamers) δ 170.82, 170.23, 169.62, 169.52, 152.90, 148.64, 142.47, 139.49, 136.24, 134.81, 131.88, 128.00, 127.73, 126.51, 125.70, 125.01, 120.01, 93.50, 72.76, 72.64, 70.25, 68.09, 61.61, 51.56, 51.34, 44.73, 44.56, 21.33, 20.91, 20.80, 20.76, 20.73.

HRMS (ESI) m/z calcd for $C_{33}H_{41}N_2O_{11}S$ $[M+H]^+$ 673.2432; **found**: 673.2426.

Compound GA70

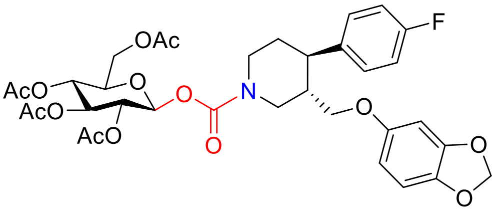

Compound **GA70** was synthesized according to the *General procedure* using amine **A48** (150 mg, 0.4 mmol, 1.0 eq) and glycosyl halide **G2** (329 mg, 0.8 mmol, 2.0 eq)

in presence of Cs_2CO_3 (196 mg, 0.6 mmol, 1.5 eq). After the reaction, the mixture was purified by silica gel flash chromatography (petroleum ether/EtOAc = 4:1) to give **GA70** (190 mg, 67%) as a white solid.

$[\alpha]_D^{25}$ = 3.7 (*c* 0.1, CH_2Cl_2).

^{1}H NMR (600 MHz, $CDCl_3$) (mixture of rotamers) δ 7.17–7.06 (m, 2H), 7.01–6.94 (m, 2H), 6.66–6.60 (m, 1H), 6.42 (d, *J* = 2.5 Hz, 0.44H), 6.34 (d, *J* = 2.5 Hz, 0.53H), 6.19–6.10 (m, 1H), 5.91–5.86 (m, 2H), 5.70–5.63 (m, 1H), 5.34–5.25 (m, 1H), 5.24–5.10 (m, 2H), 4.53–4.44 (m, 0.55H), 4.38–4.28 (m, 2H), 4.23–4.09 (m, 1.69H), 3.90–3.85 (m, 1H), 3.66–3.58 (m, 1H), 3.48–3.38 (m, 1H), 2.98–2.84 (m, 2H), 2.76–2.68 (m, 1H), 2.18–1.99 (m, 12H), 1.89–1.74 (m, 1H), 1.56–1.48 (m, 2H).

^{13}C NMR (151 MHz, $CDCl_3$) (mixture of rotamers) δ 170.81, 170.26, 170.23, 169.62, 169.40, 169.37, 161.82 (d, *J* = 245.1 Hz), 154.24, 152.91, 152.72, 148.33, 141.95, 141.92, 138.69, 128.92 (d, *J* = 7.9 Hz), 128.79 (d, *J* = 7.9 Hz), 115.79 (d, *J* = 21.3 Hz), 115.74 (d, *J* = 21.3 Hz), 108.00, 105.86, 105.68, 101.28, 98.35, 98.12, 93.64, 93.53, 72.88, 72.74, 72.65, 70.39, 70.21, 68.61, 68.56, 68.14, 68.10, 61.65, 47.60, 47.30, 45.01, 44.86, 43.88, 43.80, 41.84, 34.16, 33.49, 20.93, 20.90, 20.79, 20.76, 20.74.

HRMS (ESI) m/z calcd for $C_{34}H_{38}FNO_{14}Na$ $[M+Na]^+$ 726.2173; **found**: 726.2169.

Compound GA71

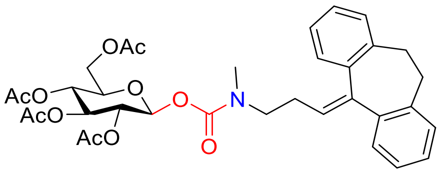

Compound **GA71** was synthesized according to the *General procedure* using amine **A49** (150 mg, 0.5 mmol, 1.0 eq) and glycosyl halide **G2** (411 mg, 1.0 mmol, 2.0 eq) in presence of Cs_2CO_3 (245 mg, 0.75 mmol, 1.5 eq). After the reaction, the mixture was purified by silica gel flash chromatography (petroleum ether/EtOAc = 2.4:1) to give **GA71** as a white solid (196 mg, 62%).

$[\alpha]_D^{25}$ = 2.9 (*c* 1.0, $CHCl_3$).

^{1}H NMR (500 MHz, $CDCl_3$) (mixture of rotamers) δ 7.30–6.96 (m, 8H), 5.83–5.69

(m, 1H), 5.63 (d, J = 8.2 Hz, 0.51H), 5.59 (d, J = 8.3 Hz, 0.49H), 5.29–5.20 (m, 1H), 5.20–5.02 (m, 2H), 4.37–4.19 (m, 1H), 4.09–3.94 (m, 1H), 3.88–3.75 (m, 1H), 3.53–3.11 (m, 4H), 2.99–2.85 (m, 1H), 2.85–2.66 (m, 4H), 2.45–2.20 (m, 2H), 2.05 (s, 3H), 2.02–1.97 (m, 6H).

^{13}C NMR (126 MHz, $CDCl_3$) (mixture of rotamers) δ 170.60, 170.04, 169.46, 169.35, 153.67, 153.58, 145.18, 144.89, 140.86, 139.75, 139.36, 137.04, 136.97, 130.02, 128.52, 128.18, 128.08, 127.99, 127.93, 127.62, 127.60, 127.25, 127.21, 126.05, 125.82, 93.14, 77.42, 77.16, 76.90, 72.74, 72.35, 72.27, 70.19, 68.02, 67.92, 61.58, 61.47, 48.94, 48.39, 33.70, 31.92, 28.01, 27.54, 20.73, 20.70, 20.58.

HRMS (ESI) m/z calcd for $C_{34}H_{39}NO_{11}Na$ $[M+Na]^+$ 660.2415; **found**: 660.2421.

Compound GA72

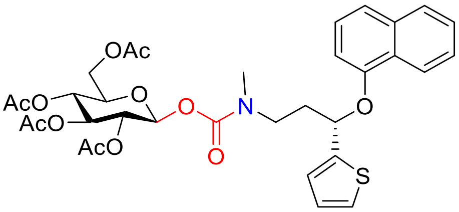

Compound **GA72** was synthesized according to the *General procedure* using amine **A50** (67 mg, 0.2 mmol, 1.0 eq) and glycosyl halide **G2** (90 mg, 0.22 mmol, 1.1 eq) in presence of Cs_2CO_3 (98 mg, 0.3 mmol, 1.5 eq). After the reaction, the mixture was purified by silica gel flash chromatography (petroleum ether/EtOAc = 2:1) to give **GA72** as a yellow solid (69 mg, 52%).

$[\alpha]_D^{25}$ = 36.8 (c 1.0, $CHCl_3$).

^{1}H NMR (500 MHz, $CDCl_3$) (mixture of rotamers) δ 8.35–8.28 (m, 1H), 7.80–7.74 (m, 1H), 7.53–7.46 (m, 2H), 7.43–7.37 (m, 1H), 7.33–7.24 (m, 1H), 7.24–7.18 (m, 1H), 7.14 (d, J = 3.5 Hz, 0.47H), 7.07 (d, J = 3.6 Hz, 0.44H), 6.96–6.90 (m, 1H), 6.86 (d, J = 7.7 Hz, 0.49H), 6.81 (d, J = 7.7 Hz, 0.45H), 5.79–5.59 (m, 2H), 5.30–5.23 (m, 1H), 5.22–5.09 (m, 2H), 4.35–4.23 (m, 1H), 4.16–4.08 (m, 1H), 3.87–3.82 (m, 1H), 3.63–3.41 (m, 2H), 2.94 (s, 1.49H), 2.88 (s, 1.35H), 2.56–2.43 (m, 1H), 2.40–2.29 (m, 0.50H), 2.22–2.14 (m, 0.65H), 2.12–2.05 (m, 2H), 2.06 – 2.01 (m, 8.50H), 1.99 (s, 1.30H), 1.93 (s, 1.46H).

^{13}C NMR (126 MHz, $CDCl_3$) (mixture of rotamers) δ 170.76, 170.18, 169.58, 169.47, 153.83, 153.74, 153.12, 153.04, 144.53, 144.48, 134.69, 127.69, 127.64, 126.78, 126.70, 126.50, 126.15, 126.12, 125.79, 125.73, 125.49, 125.46, 125.29, 125.15, 125.10, 125.02, 122.06, 121.98, 120.99, 107.09, 107.05, 93.33, 74.33, 73.92, 72.85, 72.79, 72.53, 72.44, 70.29, 70.24, 68.02, 61.58, 46.70, 46.36, 37.70, 36.65, 35.68, 34.73, 20.85, 20.82, 20.70, 20.64.

HRMS (ESI) m/z calcd for $C_{33}H_{37}NO_{12}SNa$ $[M+Na]^+$ 694.1929; **found**: 694.1937.

Compound GA73

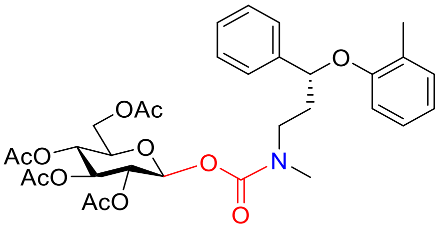

Compound **GA73** was synthesized according to the *General procedure* using amine **A51** (146 mg, 0.5 mmol, 1.0 eq) and glycosyl halide **G2** (411 mg, 1.0 mmol, 2.0 eq) in presence of Cs_2CO_3 (245 mg, 0.75 mmol, 1.5 eq). After the reaction, the mixture was purified by silica gel flash chromatography (petroleum ether/EtOAc = 2:1) to give **GA73** as a yellow solid (69 mg, 52%).

$[\alpha]_D^{25}$ = -11.3 (*c* 1.0, $CHCl_3$).

^{1}H NMR (500 MHz, $CDCl_3$) (mixture of rotamers) δ 7.36–7.27 (m, 4H), 7.25–7.20 (m, 1H), 7.13–7.07 (m, 1H), 6.98–6.89 (m, 1H), 6.82–6.72 (m, 1H), 6.59 (d, *J* = 8.7 Hz, 0.53H), 6.55 (d, *J* = 8.2 Hz, 0.46H), 5.61 (d, *J* = 8.4 Hz, 1H), 5.28–5.05 (m, 3H), 4.99 (dd, *J* = 9.6, 8.4 Hz, 0.52H), 4.34–4.23 (m, 1H), 4.13–4.06 (m, 1H), 3.85–3.77 (m, 1H), 3.58–3.35 (m, 2H), 2.90 (s, 1.59H), 2.84 (s, 1.44H), 2.34 (s, 1.57H), 2.32 (s, 1.46H), 2.26–2.08 (m, 2H), 2.04–1.97 (m, 12H).

^{13}C NMR (126 MHz, $CDCl_3$) (mixture of rotamers) δ 170.68, 170.14, 170.11, 169.52, 169.37, 169.17, 155.72, 155.64, 153.80, 153.66, 141.54, 141.47, 130.73, 130.71, 128.76, 127.74, 126.99, 126.97, 126.64, 125.73, 125.67, 120.48, 120.45, 112.71, 112.67, 93.25, 93.23, 77.33, 77.29, 76.72, 72.88, 72.75, 72.46, 72.32, 70.23, 70.14,

69.88, 67.98, 61.63, 61.53, 46.81, 46.18, 37.59, 36.56, 35.52, 34.64, 20.79, 20.76, 20.66, 20.64, 20.54, 16.60, 16.58.

HRMS (ESI) m/z calcd for $C_{32}H_{39}NO_{12}Na$ $[M+Na]^+$ 652.2364; **found**: 652.2365.

Compound GA74

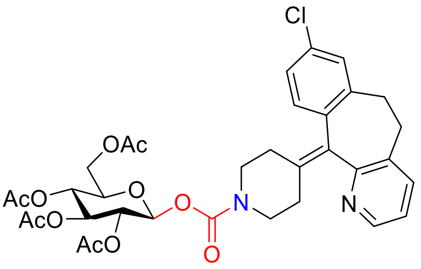

Compound **GA74** was synthesized according to the *General procedure* using amine **A52** (156 mg, 0.5 mmol, 1.0 eq) and glycosyl halide **G2** (226 mg, 0.55 mmol, 1.1 eq) in presence of Cs_2CO_3 (245 mg, 0.75 mmol, 1.5 eq). After the reaction, the mixture was purified by silica gel flash chromatography (petroleum ether/EtOAc = 1:1 to 1:4) to give **GA74** as a white solid (148mg, 43%).

$[\alpha]_D^{25}$ = -33.7 (*c* 1.0, $CHCl_3$).

^{1}H NMR (600 MHz, $CDCl_3$) (mixture of rotamers) δ 8.40 (d, *J* = 4.8 Hz, 1H), 7.49–7.41 (m, 1H), 7.20–7.05 (m, 4H), 5.73–5.58 (m, 1H), 5.38–5.23 (m, 1H), 5.22–5.06 (m, 2H), 4.36–4.27 (m, 1H), 4.15–4.06 (m, 1H), 3.89–3.65 (m, 3H), 3.41–3.29 (m, 2H), 3.25–3.10 (m, 2H), 2.94–2.71 (m, 2H), 2.61–2.20 (m, 4H), 2.10 (s, 1.31H), 2.07–2.04 (m, 3.48H), 2.03–1.99 (m, 6.41H), 1.97 (s, 1.29H).

^{13}C NMR (151 MHz, $CDCl_3$) (mixture of rotamers) δ 170.80, 170.75, 170.18, 169.58, 169.55, 169.49, 169.41, 169.35, 156.76, 152.76, 152.72, 152.66, 152.61, 146.73, 139.58, 137.90, 137.56, 136.82, 134.91, 133.55, 133.27, 130.62, 130.53, 129.17, 129.14, 129.12, 126.44, 122.57, 93.47, 72.75, 72.73, 72.70, 72.60, 70.30, 70.27, 70.25, 70.09, 68.06, 68.04, 61.58, 61.55, 45.18, 45.12, 45.07, 45.04, 31.73, 31.62, 30.73, 30.65, 30.54, 30.46, 30.38, 30.24, 20.90, 20.87, 20.78, 20.73, 20.71.

HRMS (ESI) m/z calcd for $C_{34}H_{38}N_2O_{11}$ $[M+H]^+$ 685.2159; **found**: 685.2162.

Compound GA75

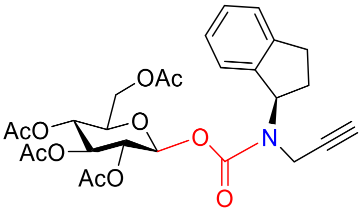

Compound **GA75** was synthesized according to the *General procedure* using amine **A53** (100 mg, 0.584 mmol, 1.0 eq) and glycosyl halide **G2** (480 mg, 1.168 mmol, 2.0 eq) in presence of Cs_2CO_3 (285 mg, 0.876 mmol, 1.5 eq). After the reaction, the mixture was purified by silica gel flash chromatography (petroleum ether/EtOAc = 4:1 to 2:1) to give a residue, which was then treated with methanol overnight, and then filted to provide **GA75** as a white solid (294 mg, 92%).

$[\alpha]_D^{25}$ = 18.1 (*c* 0.1, CH_2Cl_2).

^{1}H NMR (600 MHz, $CDCl_3$) (mixture of rotamers) δ 7.31–7.13 (m, 3.63H), 7.02 (d, *J* = 7.5 Hz, 0.38H), 5.86–5.73 (m, 1.62H), 5.64 (t, *J* = 8.0 Hz, 0.38H), 5.31–5.09 (m, 3H), 4.39–4.32 (m, 1H), 4.19–4.08 (m, 1.69H), 4.06–4.00 (m, 0.39H), 3.91–3.84 (m, 1H), 3.56–3.49 (m, 0.36H), 3.39–3.32 (m, 0.57H), 3.11–3.00 (m, 1H), 2.90–2.82 (m, 1H), 2.53–2.42 (m, 1H), 2.36–2.18 (m, 1H), 2.11 (s, 3H), 2.05–1.97 (m, 9H).

^{13}C NMR (151 MHz, $CDCl_3$) (mixture of rotamers) δ 170.81, 170.31, 170.22, 169.59, 169.57, 169.31, 169.26, 153.80, 153.69, 144.26, 143.79, 140.26, 140.05, 128.50, 128.47, 126.98, 126.78, 125.37, 125.26, 124.53, 123.92, 93.67, 93.54, 80.56, 80.05, 73.13, 72.88, 72.72, 72.70, 71.26, 70.81, 70.24, 70.17, 68.02, 67.98, 62.52, 62.22, 61.61, 61.57, 33.46, 32.72, 30.46, 30.29, 30.18, 29.79, 20.93, 20.90, 20.74, 20.72, 20.65.

HRMS (ESI) m/z calcd for $C_{27}H_{31}NO_{11}Na$ $[M+Na]^+$ 568.1788; **found**: 568.1789.

Compound GA76

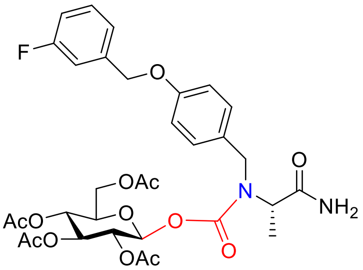

Compound **GA76** was synthesized according to the *General procedure* using amine **A54** (151 mg, 0.5 mmol, 1.0 eq) and glycosyl halide **G2** (411 mg, 1.0 mmol, 2.0 eq) in presence of Cs_2CO_3 (245 mg, 0.75 mmol, 1.5 eq). After the reaction, the mixture was purified by silica gel flash chromatography (petroleum ether/EtOAc = 1:1 to 1:5) to give **GA76** as a white solid (329 mg, 98%).

$[\alpha]_D^{25}$ = 18.1 (*c* 1.0, $CHCl_3$).

^{1}H NMR (500 MHz, $CDCl_3$) (mixture of rotamers) δ 7.30 (q, *J* = 7.3 Hz, 1H), 7.21 (d, *J* = 8.2 Hz, 1H), 7.17–7.07 (m, 3H), 6.97 (td, *J* = 8.4, 2.5 Hz, 1H), 6.87 (d, *J* = 8.3 Hz, 2H), 6.11 (bs, 0.55H), 5.88 (bs, 1H), 5.78 (bs, 0.41H), 5.72 (d, *J* = 8.3 Hz, 1H), 5.25 (t, *J* = 9.4 Hz, 1H), 5.15–5.06 (m, 2H), 5.00 (s, 2H), 4.57–4.43 (m, 1H), 4.41–4.32 (m, 1.65H), 4.25 (dd, *J* = 12.6, 4.4 Hz, 1.50H), 4.15–4.08 (m, 1H), 3.86–3.81 (m, 1H), 2.12–1.90 (m, 12H).

^{13}C NMR (126 MHz, $CDCl_3$) (mixture of rotamers) δ 173.42, 172.90, 170.64, 170.09, 169.71, 169.47, 169.42, 162.95 (d, *J* = 246.1 Hz), 157.97, 154.07, 139.51 (d, *J* = 7.2 Hz), 130.16 (d, *J* = 8.3 Hz), 129.88, 129.41, 128.87, 122.74 (d, *J* = 3.0 Hz), 114.96, 114.83 (d, *J* = 21.3 Hz), 114.21 (d, *J* = 22.0 Hz), 93.59, 72.67, 72.53, 72.46, 70.32, 70.20, 69.16, 69.14, 67.90, 61.41, 56.07, 55.49, 49.76, 48.99, 20.72, 20.66, 20.59, 20.56, 15.72, 14.70.

HRMS (ESI) m/z calcd for $C_{32}H_{37}N_2O_{13}FNa$ $[M+Na]^+$ 699.2172; **found**: 699.2179.

Compound GA77

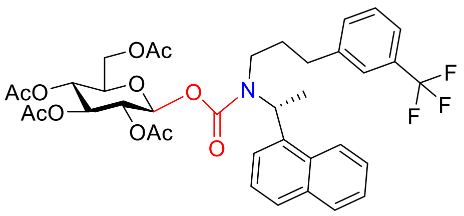

Compound **GA77** was synthesized according to the *General procedure* using amine **A55** (118 mg, 0.3 mmol, 1.0 eq) and glycosyl halide **G2** (247 mg, 0.6 mmol, 2.0 eq) in presence of Cs_2CO_3 (147 mg, 0.45 mmol, 1.5 eq). After the reaction, the mixture was purified by silica gel flash chromatography (petroleum ether/EtOAc = 4:1) to give **GA77** (145 mg, 66%) as a white solid.

$[\alpha]_D^{25}$ = -2.0 (*c* 0.1, CH_2Cl_2).

^{1}H NMR (600 MHz, $CDCl_3$) (mixture of rotamers) δ 8.17 (d, *J* = 8.4 Hz, 0.30H), 8.05 (d, *J* = 8.3 Hz, 1H), 7.90–7.85 (m, 2H), 7.84–7.78 (m, 2H), 7.76 (d, *J* = 8.2 Hz, 0.33H), 7.57–7.47 (m, 4.24H), 7.45–7.18 (m, 8.64H), 6.97 (s, 1H), 6.91 (d, *J* = 7.7 Hz, 1H), 6.88–6.82 (m, 1H), 6.64 (d, *J* = 0.6 Hz, 0.58H), 6.18 (q, *J* = 6.9 Hz, 1H), 5.98 (q, *J* = 6.8 Hz, 0.47H), 5.84 (d, *J* = 8.0 Hz, 0.51H), 5.81 (d, *J* = 8.3 Hz, 1H), 5.57 (dt, *J* = 4.3, 0.9 Hz, 0.58H), 5.38–5.27 (m, 2.07H), 5.27–5.19 (m, 1.16H), 5.17–5.08 (m, 2.14H), 4.45–4.40 (m, 0.60H), 4.40–4.28 (m, 2.34H), 4.23 (dd, *J* = 12.1, 3.5 Hz, 0.63H), 4.20–4.08 (m, 1.66H), 3.92–3.87 (m, 1.48H), 3.10 (td, *J* = 9.6, 9.1, 4.8 Hz, 0.40H), 2.95–2.80 (m, 2.43H), 2.77–2.54 (m, 1.08H), 2.23–2.15 (m, 3.12H), 2.11–2.09 (m, 7.74H), 2.05–2.01 (m, 14.81H), 1.98 (s, 2.86H), 1.67 (d, *J* = 6.9 Hz, 1.53H), 1.63 (d, *J* = 6.9 Hz, 3.47H).

^{13}C NMR (151 MHz, $CDCl_3$) (mixture of rotamers) δ 170.75, 170.63, 170.26, 170.23, 169.72, 169.62, 169.60, 169.46, 153.88, 153.63, 142.36, 142.30, 139.41, 135.18, 134.97, 134.11, 133.81, 132.23, 132.11, 131.89, 131.72, 131.50, 131.39, 130.8–130.2 (m), 129.21, 129.17, 129.15, 128.92, 128.81, 128.68, 128.64, 127.54, 127.03, 126.77, 126.22, 126.11, 125.99, 125.85, 125.51, 125.21, 125.18, 125.1–124.5 (m), 123.77, 123.42, 123.10, 122.8–122.6 (m) , 93.53, 93.42, 77.37, 77.16, 76.95, 74.28, 73.11, 72.94, 72.60, 72.44, 70.50, 70.44, 68.23, 67.62, 66.48, 61.72, 61.68, 61.09, 51.43, 50.90, 43.37, 42.20, 33.50, 32.95, 30.99, 30.02, 20.90, 20.86, 20.83, 20.73, 20.70, 20.64, 18.04, 17.25.

HRMS (ESI) m/z calcd for $C_{37}H_{40}F_3NO_{11}Na$ $[M+Na]^+$ 754.2451; **found**: 754.2446.

Compound GA78

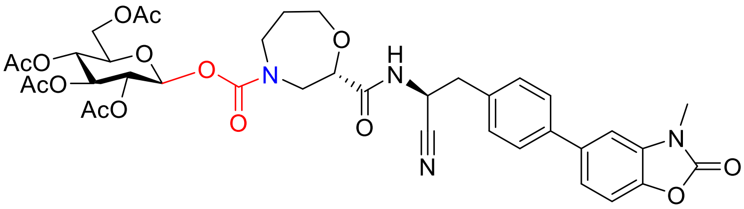

Compound **GA78** was synthesized according to the *General procedure* using amine **A56** (21 mg, 0.05 mmol, 1.0 eq) and glycosyl halide **G2** (41 mg, 0.1 mmol, 2.0 eq) in

presence of Cs_2CO_3 (25 mg, 0.075 mmol, 1.5 eq). After the reaction, the mixture was purified by silica gel flash chromatography (petroleum ether/EtOAc = 1:1 to 1:4) to give **GA78** (39 mg, 98%) as a white solid.

$[\alpha]_D^{25}$ = -19.4 (*c* 1.0, $CHCl_3$).

^{1}H NMR (600 MHz, $CDCl_3$) (mixture of rotamers) δ 7.52–7.47 (m, 2H), 7.34–7.31 (d, *J* = 7.9 Hz, 0.74H), 7.31–7.28 (d, *J* = 7.8 Hz, 1.38H), 7.26–7.23 (m, 1H), 7.21–7.17 (m, 1.33H), 7.14 (d, *J* = 8.9 Hz, 0.33H), 7.09–7.07 (m, 1H), 7.05 (d, *J* = 9.1 Hz, 0.65H), 5.63 (d, *J* = 8.1 Hz, 0.35H), 5.59 (d, *J* = 8.3 Hz, 0.65H), 5.25–5.17 (m, 1H), 5.16–5.03 (m, 3H), 4.21–4.13 (m, 1H), 4.12–3.97 (m, 4H), 3.78–3.72 (m, 1H), 3.67 (dt, *J* = 14.2, 5.9 Hz, 0.32H), 3.55–3.41 (m, 2.35H), 3.41–3.28 (m, 4.23H), 3.19–2.99 (m, 2.34H), 2.05–1.93 (m, 12H), 1.91–1.86 (m, 1.33H), 1.79–1.70 (m, 0.70H).

^{13}C NMR (151 MHz, $CDCl_3$) (mixture of rotamers) δ 170.74, 170.56, 170.16, 170.05, 169.53, 169.46, 169.43, 169.12, 168.84, 154.96, 153.37, 142.38, 140.38, 140.35, 137.16, 137.13, 133.33, 133.19, 132.42, 130.26, 130.15, 127.79, 121.60, 117.90, 110.19, 106.95, 93.50, 93.47, 79.56, 78.70, 72.62, 72.58, 72.52, 70.47, 70.29, 69.85, 69.10, 67.97, 67.87, 61.42, 61.34, 51.32, 50.86, 46.44, 45.82, 41.41, 41.09, 38.86, 38.69, 30.08, 29.19, 28.30, 20.81, 20.77, 20.71, 20.68, 20.65.

HRMS (ESI) m/z calcd for $C_{38}H_{42}N_4O_{15}Na$ $[M+Na]^+$ 817.2539; **found**: 817.2546.

Compound GA79

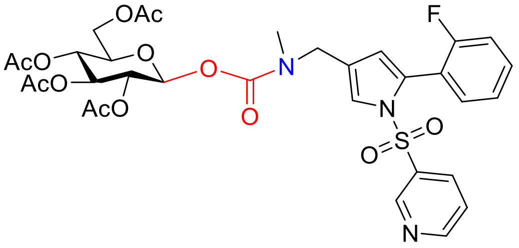

Compound **GA79** was synthesized according to the *General procedure* using amine **A57** (86 mg, 0.25 mmol, 1.0 eq) and glycosyl halide **G2** (206 mg, 0.5 mmol, 2.0 eq) in presence of Cs_2CO_3 (122 mg, 0.375 mmol, 1.5 eq). After the reaction, the mixture was purified by silica gel flash chromatography (petroleum ether/EtOAc = 1:1 to 1:4) to give **GA79** as a yellow solid (187 mg, 99%).

$[\alpha]_D^{25}$ = -6.2 (*c* 1.0, $CHCl_3$).

^{1}H NMR (500 MHz, $CDCl_3$) (mixture of rotamers) δ 8.77–8.72 (m, 1H), 8.59–8.53 (m, 1H), 7.69–7.63 (m, 1H), 7.44–7.29 (m, 3H), 7.16–7.08 (m, 2H), 7.03–6.96 (m, 1H), 6.20 (d, J = 1.9 Hz, 0.45H), 6.15 (d, J = 1.9 Hz, 0.49H), 5.68 (d, J = 8.3 Hz, 0.52H), 5.65 (d, J = 8.3 Hz, 0.48H), 5.28–5.21 (m, 1H), 5.20–5.14 (m, 1H), 5.14–5.05 (m, 1H), 4.33–4.16 (m, 3H), 4.13–4.02 (m, 1H), 3.86–3.78 (m, 1H), 2.87 (s, 1.52H), 2.82 (s, 1.44H), 2.08–1.94 (m, 12H).

^{13}C NMR (126 MHz, $CDCl_3$) (mixture of rotamers) δ 170.69, 170.12, 169.54, 169.53, 169.44, 160.69 (d, J = 249.4 Hz), 154.37, 154.03, 153.51, 147.97, 135.01, 134.74, 133.26, 131.46, 131.40, 129.35, 129.32, 123.86, 123.76, 123.58, 123.50 (d, J = 3.7 Hz), 122.01, 121.88, 118.78, 118.62, 117.78, 117.58, 115.45 (d, J = 21.8 Hz), 93.45, 93.42, 72.82, 72.68, 72.57, 72.48, 70.29, 70.12, 67.96, 61.52, 45.43, 45.04, 34.36, 33.80, 20.77, 20.69, 20.66, 20.65, 20.60, 20.53.

HRMS (ESI) m/z calcd for $C_{32}H_{34}N_3O_{13}SFNa$ $[M+Na]^+$ 742.1689; **found**: 742.1699.

Compound GA80

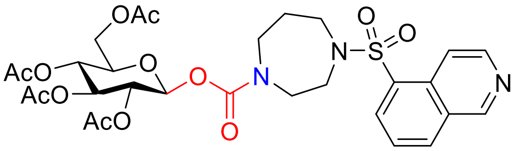

Compound **GA80** was synthesized according to the *General procedure* using amine **A58** (291 mg, 1.0 mmol, 1.0 eq) and glycosyl halide **G2** (452 mg, 1.1 mmol, 1.1 eq) in presence of K_2CO_3 (208 mg, 1.5 mmol, 1.5 eq). After the reaction, the mixture was purified by silica gel flash chromatography (petroleum ether/EtOAc = 1:1 to 1:4) to give **GA80** as a white solid (422 mg, 64%).

$[\alpha]_D^{25}$ = 20.4 (c 1.0, $CHCl_3$).

^{1}H NMR (600 MHz, $CDCl_3$) (mixture of rotamers) δ 9.32 (s, 1H), 8.66 (t, J = 6.5 Hz, 1H), 8.38–8.32 (m, 1H), 8.29 (ddd, J = 7.4, 4.9, 1.2 Hz, 1H), 8.18 (dq, J = 8.2, 1.3 Hz, 1H), 7.66 (ddd, J = 8.2, 7.3, 5.1 Hz, 1H), 5.63–5.58 (m, 1H), 5.26–5.20 (m, 1H), 5.16–5.03 (m, 2H), 4.27–4.20 (m, 1H), 4.11–4.05 (m, 1H), 3.89–3.77 (m, 1H), 3.74–3.57 (m, 2H), 3.56–3.20 (m, 6H), 2.05–1.81 (m, 14H).

^{13}C NMR (151 MHz, $CDCl_3$) (mixture of rotamers) δ 170.57, 170.04, 170.02, 169.55,

169.49, 169.40, 153.41, 153.36, 153.00, 145.27, 134.20, 134.18, 133.73, 133.71, 133.13, 133.10, 131.55, 129.25, 125.96, 125.94, 117.40, 117.37, 93.34, 93.31, 72.66, 72.61, 72.50, 72.47, 72.41, 70.15, 70.12, 67.97, 61.46, 49.61, 49.54, 49.15, 49.13, 47.49, 47.35, 46.41, 46.10, 28.55, 28.02, 20.75, 20.69, 20.66, 20.62.

HRMS (ESI) m/z calcd for $C_{29}H_{35}N_3O_{13}Na$ $[M+Na]^+$ 688.1783; **found**: 688.1788.

Compound GA81

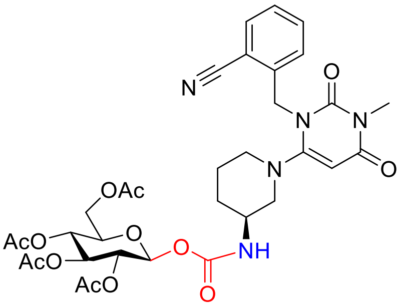

Compound **GA81** was synthesized according to the *General procedure* using amine **A59** (170 mg, 0.5 mmol, 1.0 eq) and glycosyl halide **G1** (366 mg, 1.0 mmol, 2.0 eq) in presence of Cs_2CO_3 (245 mg, 0.75 mmol, 1.5 eq). After the reaction, the mixture was purified by silica gel flash chromatography (petroleum ether/EtOAc = 1:1 to 1:3) to give **GA81** as a white solid (187 mg, 96%).

$[\alpha]_D^{25}$ = 40.9 (*c* 1.0, $CHCl_3$).

^{1}H NMR (500 MHz, $CDCl_3$) δ 7.67 (d, *J* = 7.8 Hz, 1H), 7.56–7.47 (m, 1H), 7.35 (t, *J* = 7.6 Hz, 1H), 7.11 (d, *J* = 7.9 Hz, 1H), 5.56 (d, *J* = 8.4 Hz, 1H), 5.35–5.14 (m, 5H), 5.06 (t, *J* = 9.7 Hz, 1H), 5.00 (t, *J* = 9.0 Hz, 1H), 4.27 (dd, *J* = 12.5, 4.2 Hz, 1H), 4.12–4.00 (m, 1H), 3.78 (ddd, *J* = 10.1, 4.2, 2.3 Hz, 1H), 3.74–3.65 (m, 1H), 3.24 (s, 3H), 3.21–3.15 (m, 1H), 2.91–2.42 (m, 3H), 2.03 (s, 3H), 2.00–1.97 (m, 6H), 1.95 (s, 3H), 1.91–1.80 (m, 1H), 1.80–1.72 (m, 1H), 1.66–1.56 (m, 1H), 1.48–1.37 (m, 1H).

^{13}C NMR (126 MHz, $CDCl_3$) δ 170.54, 169.99, 169.50, 169.49, 162.96, 159.32, 152.85, 152.54, 140.46, 133.41, 133.31, 128.11, 126.95, 117.17, 110.74, 92.71, 90.86, 72.79, 72.38, 70.13, 67.74, 61.43, 55.42, 46.06, 28.00, 20.72, 20.62, 20.55.

HRMS (ESI) m/z calcd for $C_{33}H_{39}N_5O_{13}Na$ $[M+Na]^+$ 736.2437; **found**: 736.2438.

Compound GA82

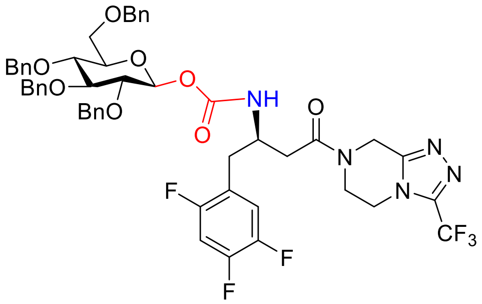

Compound **GA82** was synthesized according to the *General procedure* using amine **A60** (407 mg, 1.0 mmol, 1.0 eq) and glycosyl halide **G4** (1680 mg, 3.0 mmol, 3.0 eq) in presence of Cs_2CO_3 (490 mg, 1.5 mmol, 1.5 eq). After the reaction, the mixture was purified by silica gel flash chromatography (petroleum ether/EtOAc = 1:1 to 1:3) to give **GA82** as a white solid (187 mg, 96%).

$[\alpha]_D^{25}$ = 40.9 (*c* 1.0, $CHCl_3$).

^{1}H NMR (600 MHz, $CDCl_3$) (mixture of rotamers) δ 7.29-7.20 (m, 18H), 7.14-7.09 (m, 2H), 7.07-7.00 (m, 1H), 6.74-6.65 (m, 1H), 5.97-5.92 (m, 1H), 5.47 (d, *J* = 8.2 Hz, 1H), 4.92-4.84 (m, 2H), 4.84-4.76 (m, 3H), 4.73-4.62 (m, 2H), 4.57-4.51 (m, 1H), 4.48 (d, *J* = 10.8 Hz, 1H), 4.41 (d, *J* = 12.0 Hz, 1H), 4.28-3.82 (m, 5H), 3.73-3.59 (m, 4H), 3.58-3.53 (m, 1H), 3.52-3.45 (m, 1H), 3.00-2.88 (m, 2H), 2.76-2.64 (m, 2H).

^{13}C NMR (151 MHz, $CDCl_3$) (mixture of rotamers) δ 170.29, 169.59, 169.35, 161.98, 156.14 (dd, *J* = 243.3, 9.1 Hz), 153.88, 153.80, 150.33, 149.61, 148.93 (dt, *J* = 251.5, 13.5 Hz), 146.74 (dd, *J* = 243.8, 12.6 Hz), 144.20-142.93 (m), 138.37, 138.33, 138.10, 137.99, 137.97, 137.93, 137.81, 137.75, 131.30, 129.62, 128.86, 128.83, 128.81, 128.67, 128.53, 128.45, 128.41, 128.30, 128.15, 128.04, 127.98, 127.93, 127.91, 127.86, 127.83, 127.79, 127.76, 127.74, 127.67, 121.43-120.94 (m), 119.53-118.87 (m), 117.42, 117.35, 115.63, 115.56, 105.45 (dd, *J* = 28.6, 20.7 Hz), 95.39, 95.36, 84.65, 80.97, 80.84, 77.31, 77.27, 75.78, 75.12, 75.03, 74.77, 74.74, 73.43, 69.13, 68.45, 68.39, 49.16, 48.93, 43.60, 43.19, 42.47, 41.56, 39.07, 37.91, 37.78, 36.47, 36.13, 35.33, 32.88, 32.54.

HRMS (ESI) m/z calcd for $C_{51}H_{49}N_5O_8F_6Na$ $[M+Na]^+$ 996.3378; **found**: 996.3377.

Compound GA83

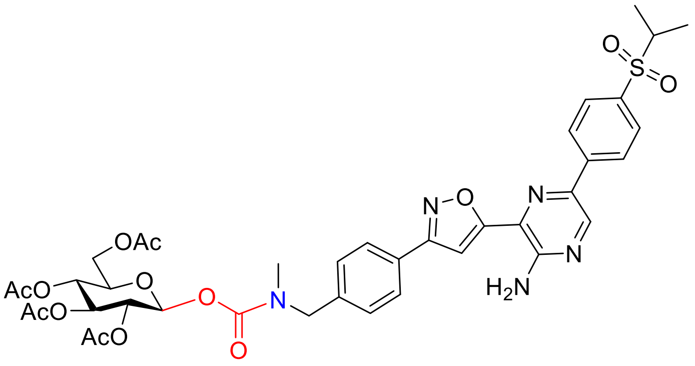

Compound **GA83** was synthesized according to the *General procedure* using amine **A61** (23 mg, 0.05 mmol, 1.0 eq) and glycosyl halide **G2** (41 mg, 0.1 mmol, 2.0 eq) in presence of Cs_2CO_3 (25 mg, 0.075 mmol, 1.5 eq). After the reaction, the mixture was purified by silica gel flash chromatography (petroleum ether/EtOAc = 1:1 to 1:3) to give **GA83** as a white solid (41 mg, 97%).

$[\alpha]_D^{25}$ = -5.2 (*c* 0.4, $CHCl_3$).

^{1}H NMR (600 MHz, $CDCl_3$) (mixture of rotamers) δ 8.64 (s, 1H), 8.18–8.12 (m, 2H), 7.99–7.94 (m, 2H), 7.90–7.82 (m, 2H), 7.39–7.34 (m, 2H), 7.33–7.27 (m, 1H), 6.14 (bs, 2H), 5.72 (d, *J* = 8.3 Hz, 1H), 5.32–5.23 (m, 1H), 5.24–5.10 (m, 2H), 4.66–4.59 (m, 0.48H), 4.56–4.48 (m, 1H), 4.42–4.26 (m, 1.52H), 4.20–4.06 (m, 1.53H), 3.90–3.84 (m, 1H), 3.28–3.19 (m, 1H), 2.94 (s, 1.52H), 2.86 (s, 1.52H), 2.09–2.05 (m, 3H), 2.04–1.99 (m, 6H), 1.99–1.96 (m, 1.67H), 1.92–1.87 (m, 1.96H), 1.35–1.27 (m, 6H).

^{13}C NMR (151 MHz, $CDCl_3$) (mixture of rotamers) δ 170.74, 170.17, 169.57, 169.47, 169.44, 169.36, 162.51, 162.47, 154.44, 153.86, 150.89, 150.86, 141.28, 140.04, 139.99, 139.39, 139.31, 136.63, 136.59, 129.85, 128.60, 128.03, 127.85, 127.74, 127.44, 126.11, 125.93, 102.20, 102.17, 102.14, 93.58, 93.49, 72.86, 72.73, 72.64, 72.54, 70.36, 70.13, 68.03, 61.58, 55.81, 52.72, 52.25, 34.88, 34.09, 20.85, 20.72, 20.68, 20.66, 20.60, 15.84.

HRMS (ESI) m/z calcd for $C_{39}H_{43}N_5O_{14}SNa$ $[M+Na]^+$ 860.2419; **found**: 860.2423.

Compound GA84

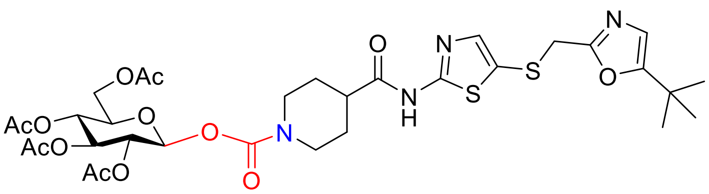

Compound **GA84** was synthesized according to the *General procedure* using amine **A62** (19 mg, 0.05 mmol, 1.0 eq) and glycosyl halide **G2** (41 mg, 0.1 mmol, 2.0 eq) in presence of Cs_2CO_3 (25 mg, 0.075 mmol, 1.5 eq). After the reaction, the mixture was purified by silica gel flash chromatography (petroleum ether/EtOAc = 1:1 to 1:4) to give **GA84** as a white solid (28 mg, 73%).

$[\alpha]_D^{25}$ = -6.2 (*c* 0.4, $CHCl_3$).

^{1}H NMR (600 MHz, $CDCl_3$) (mixture of rotamers) δ 11.61 (bs, 1H), 7.27 (s, 1H), 6.57 (s, 1H), 5.65–5.59 (m, 1H), 5.30–5.23 (m, 1H), 5.18–5.08 (m, 2H), 4.34–4.27 (m, 1H), 4.21–4.15 (m, 1H), 4.13–4.04 (m, 1H), 3.95 (s, 2H), 3.87–3.81 (m, 1H), 3.03–2.88 (m, 2H), 2.62–2.53 (m, 1H), 2.11–1.99 (m, 12H), 1.94–1.75 (m, 4H), 1.23 (s, 9H).

^{13}C NMR (151 MHz, $CDCl_3$) (mixture of rotamers) δ 172.30, 172.24, 170.85, 170.76, 170.18, 170.16, 169.59, 169.57, 169.50, 162.63, 162.00, 158.89, 152.70, 152.55, 143.55, 121.64, 120.22, 93.48, 72.75, 72.61, 72.57, 70.23, 70.19, 68.05, 68.01, 61.59, 61.55, 43.43, 43.37, 43.33, 43.26, 42.30, 42.16, 34.94, 31.55, 28.66, 28.10, 27.89, 20.88, 20.86, 20.78, 20.76, 20.70, 20.69.

HRMS (ESI) m/z calcd for $C_{32}H_{42}N_4O_{13}S_2Na$ $[M+Na]^+$ 777.2082; **found**: 777.2077.

Compound GA85

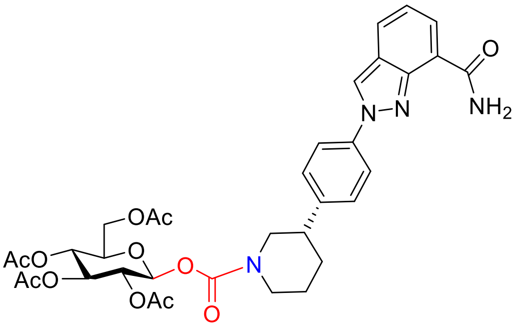

Compound **GA85** was synthesized according to the *General procedure* using amine **A63** (30 mg, 0.084 mmol, 1.0 eq) and glycosyl halide **G2** (69 mg, 0.168 mmol, 2.0 eq) in presence of K_2CO_3 (17 mg, 0.126 mmol, 1.5 eq). After the reaction, the mixture was purified by silica gel flash chromatography (petroleum ether/EtOAc = 1:1 to 1:4), then the mixture was purified by silica gel flash chromatography (dichloromethane/ MeOH = 10:1) to give **GA85** (27 mg, 46%) as a yellow solid.

$[\alpha]_D^{25}$ = -12.9 (*c* 0.1, CH_2Cl_2).

^{1}H NMR (600 MHz, $CDCl_3$) (mixture of rotamers) δ 9.13–8.99 (m, 1H), 8.53–8.48 (m, 1H), 8.31 (d, *J* = 7.0 Hz, 1H), 7.96–7.77 (m, 3H), 7.50–7.37 (m, 2H), 7.30–7.24 (m, 1H), 6.07 (d, *J* = 3.6 Hz, 1H), 5.72 (d, *J* = 8.2 Hz, 0.49H), 5.65 (d, *J* = 8.3 Hz, 0.48H), 5.34–5.25 (m, 1H), 5.23–5.07 (m, 2H), 4.41–4.06 (m, 4H), 3.90–3.82 (m, 1H), 2.90–2.77 (m, 2.44H), 2.68–2.60 (m, 0.63H), 2.11 (s, 3H), 2.08–2.00 (m, 9H), 1.93–1.67 (m, 4H).

^{13}C NMR (151 MHz, $CDCl_3$) (mixture of rotamers) δ 170.79, 170.76, 170.22, 170.17, 169.61, 169.53, 167.24, 152.98, 152.39, 147.05, 143.60, 143.16, 138.78, 138.75, 131.09, 128.54, 128.52, 125.19, 123.79, 122.67, 121.81, 121.77, 121.43, 121.19, 93.55, 93.44, 72.79, 72.63, 72.60, 70.56, 70.26, 68.08, 61.61, 51.23, 50.64, 44.66, 44.54, 42.44, 41.90, 31.49, 30.78, 29.83, 29.79, 25.38, 25.32, 20.91, 20.85, 20.79, 20.74, 20.72.

HRMS (ESI) m/z calcd for $C_{34}H_{38}N_4O_{12}Na$ $[M+Na]^+$ 717.2381; **found**: 717.2378.

Compound GA86

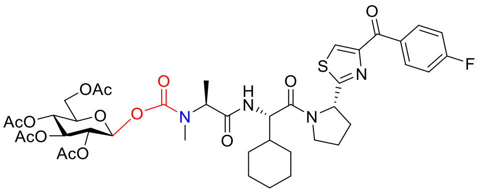

Compound **GA86** was synthesized according to the *General procedure* using amine **A64** (20 mg, 0.04 mmol, 1.0 eq) and glycosyl halide **G2** (33 mg, 0.08 mmol, 2.0 eq) in presence of Cs_2CO_3 (20 mg, 0.06 mmol, 1.5 eq). After the reaction, the mixture was purified by silica gel flash chromatography (petroleum ether/EtOAc = 1:1 to 1:4) to give **GA86** as a white liquid (32 mg, 90%).

$[\alpha]_D^{25}$ = -36.6 (*c* 0.1, $CHCl_3$).

^{1}H NMR (600 MHz, $CDCl_3$) (mixture of rotamers) δ 8.34–8.27 (m, 2H), 8.16 (s, 0.81H), 7.34–7.28 (m, 1H), 7.15 (t, *J* = 8.6 Hz, 2H), 6.69 (d, *J* = 8.8 Hz, 0.31H), 6.61 (d, *J* = 8.6 Hz, 0.63H), 5.63 (d, *J* = 8.3 Hz, 0.81H), 5.60–5.48 (m, 1.54H), 5.34–5.24 (m, 1.12H), 5.22–5.04 (m, 2.08H), 4.97–4.94 (m, 0.25H), 4.74 (q, *J* = 7.1 Hz, 0.62H), 4.68–4.58 (m, 0.94H), 4.55–4.47 (m, 0.45H), 4.42 (q, *J* = 12.0 Hz, 0.58H), 4.34–4.25 (m, 1.07H), 4.15–4.06 (m, 1.31H), 3.98–3.70 (m, 3.39H), 3.07–2.68 (m, 3.61H), 2.46–2.39 (m, 1H), 2.37–1.97 (m, 18.76H), 1.84–1.57 (m, 10.19H), 1.48–0.91 (m, 14.46H).

^{13}C NMR (151 MHz, $CDCl_3$) (mixture of rotamers) δ 222.31, 185.22, 172.10, 172.07, 171.46, 171.20, 171.09, 170.74, 170.64, 170.55, 170.15, 169.81, 169.73, 169.57, 169.55, 166.64, 164.95, 154.58, 153.98, 153.55, 153.36, 139.21, 133.51, 133.45, 128.48, 128.42, 128.35, 127.37, 127.24, 115.49, 115.35, 113.37, 93.81, 74.23, 72.71, 72.66, 72.60, 72.41, 70.34, 69.93, 68.18, 67.97, 61.68, 61.54, 58.82, 55.32, 55.23, 54.91, 54.70, 50.93, 47.73, 47.67, 42.32, 41.49, 41.04, 40.93, 38.99, 35.72, 33.35, 32.07, 31.67, 30.53, 30.46, 30.16, 30.07, 28.35, 28.13, 27.43, 26.17, 26.02, 26.00, 25.88, 25.59, 24.69, 22.25, 21.43, 21.29, 20.85, 20.79, 20.71, 20.69, 20.63, 20.20, 18.39, 14.65, 14.31, 13.95.

HRMS (ESI) m/z calcd for $C_{41}H_{51}N_4O_{14}SFNa$ $[M+Na]^+$ 897.2999; **found**: 897.3001.

Compound GA87

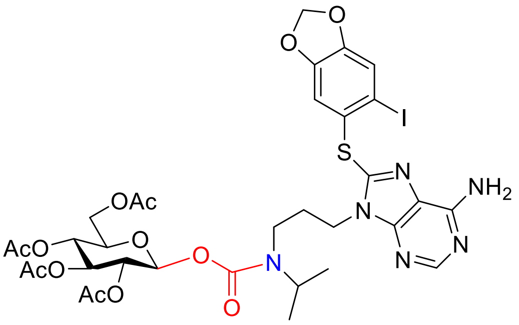

Compound **GA87** was synthesized according to the *General procedure* using amine **A65** (9 mg, 0.0176 mmol, 1.0 eq) and glycosyl halide **G2** (15 mg, 0.0352 mmol, 2.0 eq) in presence of Cs_2CO_3 (9 mg, 0.0264 mmol, 1.5 eq). After the reaction, the mixture was purified by silica gel flash chromatography (petroleum ether/EtOAc = 1:1), and the crude product was further purified by silica gel flash chromatography (dichloromethane/MeOH = 3:1) to give **GA87** (12 mg, 77%) as a yellow solid.

$[\alpha]_D^{25}$ = -4.4 (*c* 1.0, $CHCl_3$).

^{1}H NMR (600 MHz, $CDCl_3$) (mixture of rotamers) δ 8.32 (s, 0.46H), 8.28 (s, 0.48H), 7.36–7.30 (m, 1H), 7.04–7.00 (m, 1H), 6.55 (bs, 1H), 6.45 (bs, 1H), 6.01 (s, 2H), 5.67–5.62 (m, 1H), 5.27–5.22 (m, 1H), 5.21–5.05 (m, 2H), 4.34–4.18 (m, 3H), 4.19–4.07 (m, 2H), 3.85–3.79 (m, 1H), 3.31–3.16 (m, 2H), 3.10–3.01 (m, 0.78H), 2.17–2.08 (m, 1H), 2.08–1.99 (m, 12H), 1.97 (s, 2H), 1.19–1.02 (m, 6H).

^{13}C NMR (151 MHz, $CDCl_3$) (mixture of rotamers) δ 170.75, 170.22, 169.68, 169.59, 169.42, 169.38, 153.70, 153.14, 152.96, 151.48, 149.91, 149.66, 149.48, 148.39, 126.64, 126.50, 119.93, 119.58, 113.53, 113.45, 102.60, 93.27, 93.20, 93.14, 93.03, 72.90, 72.84, 72.57, 70.48, 70.24, 68.20, 68.06, 61.77, 61.59, 48.72, 48.21, 42.14, 42.02, 40.79, 40.39, 30.58, 29.94, 29.83, 29.79, 21.15, 20.93, 20.90, 20.78, 20.73, 20.48, 20.45.

HRMS (ESI) m/z calcd for $C_{33}H_{39}N_6O_{13}SINa$ $[M+Na]^+$ 909.1233; **found**: 909.1226.

Compound GA88

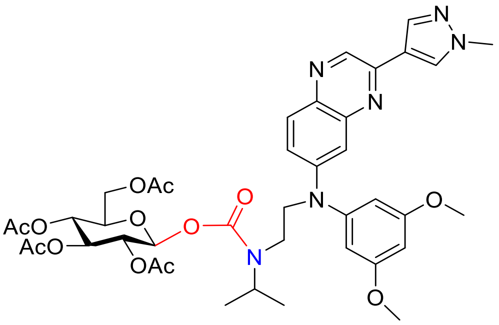

Compound **GA88** was synthesized according to the *General procedure* using amine **A66** (45 mg, 0.1 mmol, 1.0 eq) and glycosyl halide **G2** (83 mg, 0.2 mmol, 2.0 eq) in presence of Cs_2CO_3 (49 mg, 0.15 mmol, 1.5 eq). After the reaction, the mixture was purified by silica gel flash chromatography (petroleum ether/EtOAc = 1:1 to 1:6) to give **GA88** as an orange solid (72 mg, 88%).

$[\alpha]_D^{25}$= 13.5 (*c* 1.0, $CHCl_3$).

^{1}H NMR (600 MHz, $CDCl_3$) (mixture of rotamers) δ 8.79 (d, *J* = 13.6 Hz, 1H), 8.36–8.15 (m, 1H), 8.13 (d, *J* = 8.5 Hz, 1H), 7.84–7.73 (m, 1H), 7.53–7.30 (m, 2H), 6.43–6.35 (m, 2H), 6.35–6.23 (m, 1H), 5.74 (d, *J* = 8.4 Hz, 0.47H), 5.72 (d, *J* = 8.3 Hz, 0.52H), 5.29–5.21 (m, 1H), 5.22–5.07 (m, 2H), 4.37–4.04 (m, 4H), 4.01–3.91 (m, 4H), 3.91–3.79 (m, 1H), 3.77 (s, 3H), 3.74 (s, 3H), 3.59–3.46 (m, 1H), 3.44–3.30 (m, 1H), 2.06 (s, 1.47H), 2.03–1.95 (m, 9H), 1.91 (s, 1.47H), 1.19 (d, *J* = 6.8 Hz, 1.86H), 1.13 (d, *J* = 6.8 Hz, 1.44H), 1.10 (d, *J* = 6.8 Hz, 1.75H), 1.02 (d, *J* = 6.7 Hz, 1.58H).

^{13}C NMR (151 MHz, $CDCl_3$) (mixture of rotamers) δ 170.68, 170.12, 170.09, 169.52, 169.31, 169.27, 161.90, 161.84, 153.92, 153.44, 149.30, 148.16, 147.83, 139.58, 138.42, 138.32, 137.07, 136.81, 130.30, 129.57, 129.53, 123.14, 122.55, 103.48, 103.30, 97.25, 96.53, 93.28, 93.14, 72.96, 72.81, 72.49, 72.43, 70.37, 70.16, 67.99, 61.59, 61.51, 55.50, 55.44, 51.91, 50.91, 48.89, 48.24, 40.22, 39.87, 39.43, 20.99, 20.78, 20.69, 20.67, 20.64, 20.61, 20.58, 20.49.

HRMS (ESI) m/z calcd for $C_{40}H_{48}N_6O_{13}Na$ $[M+Na]^+$ 843.3172; **found**: 843.3171.

Compound GA89

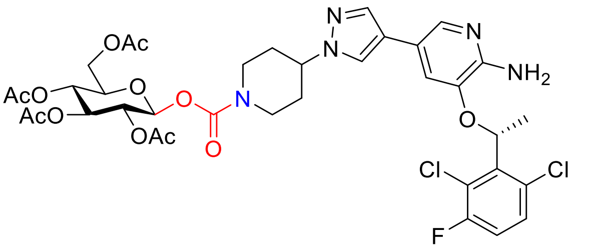

Compound **GA89** was synthesized according to the *General procedure* using amine **A67** (225 mg, 0.5 mmol, 1.0 eq) and glycosyl halide **G2** (411 mg, 1.0 mmol, 2.0 eq) in presence of Cs_2CO_3 (245 mg, 0.75 mmol, 1.5 eq). After the reaction, the mixture was purified by silica gel flash chromatography (petroleum ether/EtOAc = 1:4 to 1:10) to give **GA89** as a yellow solid (72 mg, 88%).

$[\alpha]_D^{25}$ = -36.6 (*c* 1.0, $CHCl_3$).

^{1}H NMR (600 MHz, $CDCl_3$) (mixture of rotamers) δ 7.72–7.67 (m, 1H), 7.55–7.50 (m, 1H), 7.48–7.42 (m, 1H), 7.30–7.25 (m, 1H), 7.05–6.99 (m, 1H), 6.85–6.82 (m, 1H), 6.09–6.00 (m, 1H), 5.65–5.59 (m, 1H), 5.29–5.23 (m, 1H), 5.18–5.13 (m, 1H), 5.13–5.07 (m, 1H), 4.96 (bs, 2H), 4.36–4.04 (m, 5H), 3.88–3.80 (m, 1H), 3.11–2.87 (m, 3H), 2.19–2.08 (m, 2H), 2.09–1.91 (m, 14H), 1.82 (d, *J* = 6.7 Hz, 3H).

^{13}C NMR (151 MHz, $CDCl_3$) (mixture of rotamers) δ 170.69, 170.10, 169.53, 169.46, 158.43, 156.77, 152.64, 152.53, 148.96, 139.96, 136.84, 136.10, 136.01, 134.77, 130.03, 128.99, 128.96, 123.06, 122.80, 122.14, 122.02, 120.06, 119.96, 118.94, 118.91, 116.93, 116.77, 115.15, 115.11, 93.47, 72.66, 72.58, 72.52, 70.18, 67.97, 61.51, 61.47, 58.86, 58.68, 43.17, 43.11, 42.99, 32.27, 32.10, 31.95, 31.85, 20.80, 20.71, 20.64, 20.63, 18.93.

HRMS (ESI) m/z calcd for $C_{36}H_{41}N_5O_{12}Cl_2FNa$ $[M+Na]^+$ 824.2107; **found**: 824.2115.

Compound GA90

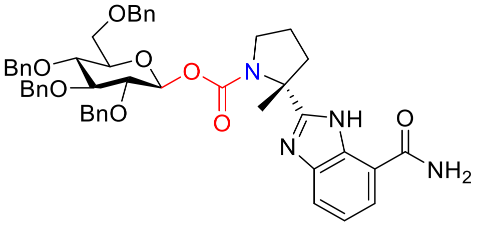

Compound **GA90** was synthesized according to the *General procedure* using amine

A68 (24 mg, 0.1 mmol, 1.0 eq) and glycosyl halide **G4** (168 mg, 0.3 mmol, 3.0 eq) in presence of Cs_2CO_3 (49 mg, 0.15 mmol, 1.5 eq). After the reaction, the mixture was purified by silica gel flash chromatography (petroleum ether/EtOAc = 1:1 to 1:3) to give **GA90** as a white solid (50 mg, 56%).

$[\alpha]_D^{25}$ = 53.3 (*c* 0.5, $CHCl_3$).

^{1}H NMR (600 MHz, $CDCl_3$) δ 8.07 (bs, 1H), 7.48 (bs, 1H), 7.42–7.20 (m, 19H), 7.17–7.12 (m, 2H), 6.32 (bs, 1H), 5.67 (d, *J* = 8.1 Hz, 1H), 4.92 (d, *J* = 11.0 Hz, 1H), 4.89–4.74 (m, 4H), 4.55–4.50 (m, 2H), 4.43 (d, *J* = 12.0 Hz, 1H), 3.83–3.56 (m, 7H), 3.41–3.33 (m, 1H), 3.23–3.12 (m, 1H), 2.09–1.86 (m, 7H).

^{13}C NMR (151 MHz, $CDCl_3$) δ 168.06, 157.64, 153.97, 138.42, 138.28, 137.95, 137.75, 128.60, 128.55, 128.49, 128.08, 128.06, 127.97, 127.95, 127.89, 127.86, 127.75, 95.48, 84.87, 81.34, 77.26, 75.79, 75.46, 75.17, 73.54, 68.16, 63.22, 48.92, 39.77, 25.41, 22.97.

HRMS (ESI) m/z calcd for $C_{48}H_{50}N_4O_8Na$ $[M+Na]^+$ 833.3521; **found**: 833.3520.

Compound GA91

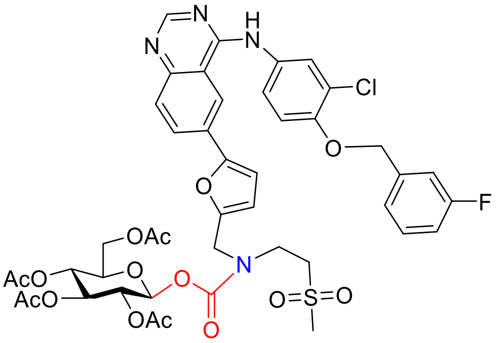

Compound **GA91** was synthesized according to the *General procedure* using amine **A69** (145 mg, 0.25 mmol, 1.0 eq) and glycosyl halide **G2** (206 mg, 0.5 mmol, 2.0 eq) in presence of Cs_2CO_3 (122 mg, 0.375 mmol, 1.5 eq). After the reaction, the mixture was purified by silica gel flash chromatography (petroleum ether/EtOAc = 1:1 to 1:8) to give **GA91** as a white solid (230 mg, 97%).

$[\alpha]_D^{25}$ = -15.5 (*c* 0.1, CH_2Cl_2).

^{1}H NMR (500 MHz, $CDCl_3$) (mixture of rotamers) δ 8.64 (s, 2H), 8.39 (s, 1H), 7.92–7.86 (m, 2H), 7.85–7.79 (m, 1H), 7.71–7.63 (m, 1H), 7.37–7.29 (m, 1H),

7.24–7.14 (m, 2H), 7.02–6.96 (m, 1H), 6.96–6.91 (m, 1H), 6.73–6.68 (m, 1H), 6.47–6.41 (m, 1H), 5.66 (d, *J* = 7.6 Hz, 0.44H), 5.63 (d, *J* = 8.2 Hz, 0.55H), 5.33–5.24 (m, 1.46H), 5.20–5.07 (m, 3.56H), 4.74 (d, *J* = 16.0 Hz, 0.43H), 4.65 (d, *J* = 15.7 Hz, 0.55H), 4.58 (d, *J* = 15.8 Hz, 0.55H), 4.38 (d, *J* = 16.0 Hz, 0.43H), 4.34–4.25 (m, 1H), 4.17–4.02 (m, 2.81H), 4.02–3.88 (m, 1H), 3.87–3.81 (m, 1H), 3.54–3.44 (m, 0.45H), 3.44–3.27 (m, 1.55H), 3.00 (s, 1.66H), 2.92 (s, 1.33H), 2.10–1.96 (m, 12H).

^{13}C NMR (126 MHz, $CDCl_3$) (mixture of rotamers) δ 170.62, 170.08, 169.94, 169.83, 169.52, 163.03 (d, *J* = 246.3 Hz), 158.01, 153.46, 153.32, 153.12, 152.88, 150.88, 150.20, 150.10, 139.26, 139.20, 132.66, 130.24 (d, *J* = 8.1 Hz), 129.08, 128.20, 125.00, 124.94, 123.12, 123.08, 122.51, 122.28, 122.20, 115.60, 114.93 (d, *J* = 21.2 Hz), 114.17, 114.02 (d, *J* = 21.8 Hz), 112.27, 112.04, 107.63, 93.70, 93.61, 72.61, 72.54, 72.25, 70.36, 70.29, 70.11, 67.80, 61.41, 61.28, 52.61, 52.18, 44.59, 44.50, 41.46, 41.17, 20.81, 20.79, 20.62, 20.59.

^{19}F NMR (376 MHz, $CDCl_3$) δ -112.60, -112.62.

HRMS (ESI) m/z calcd for $C_{44}H_{45}ClFN_4O_{15}S$ $[M+H]^+$ 955.2286; **found**: 955.2269.

Compound GA92

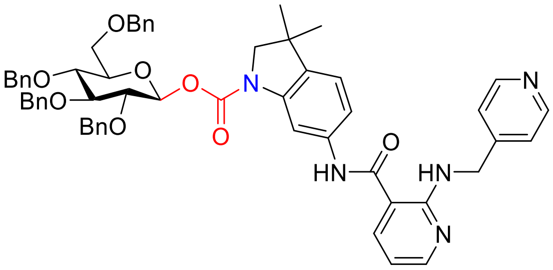

Compound **GA92** was synthesized according to the *General procedure* using amine **A70** (75 mg, 0.2 mmol, 1.0 eq) and glycosyl halide **G2** (335 mg, 0.6 mmol, 3.0 eq) in presence of Cs_2CO_3 (98 mg, 0.3 mmol, 1.5 eq). After the reaction, the mixture was purified by silica gel flash chromatography (petroleum ether/EtOAc = 1:1 to 1:4) to give **GA92** as a yellow solid (141 mg, 75%).

$[\alpha]_D^{25}$ = 17.2 (*c* 0.5, $CHCl_3$).

^{1}H NMR (500 MHz, $CDCl_3$) (mixture of rotamers) δ 8.63 (t, *J* = 6.0 Hz, 1.12H), 8.59–8.40 (m, 2.44H), 8.35–8.29 (m, 1.19H), 8.19 (d, *J* = 4.8 Hz, 1.15H), 7.98 (d, *J* =

2.0 Hz, 1H), 7.81 (d, J = 7.6 Hz, 1H), 7.78–7.69 (m, 0.34H), 7.46 (dd, J = 8.0, 1.9 Hz, 1.11H), 7.37–7.19 (m, 23.22H), 7.19–7.11 (m, 3.39H), 7.08 (d, J = 8.1 Hz, 1.23H), 6.59 (dd, J = 7.6, 4.8 Hz, 1H), 6.55–6.49 (m, 0.14H), 5.87 (d, J = 7.7 Hz, 0.15H), 5.71 (d, J = 8.1 Hz, 1H), 4.91 (d, J = 11.0 Hz, 1.20H), 4.88–4.78 (m, 4.87H), 4.73 (t, J = 4.8 Hz, 2.15H), 4.67–4.50 (m, 3H), 4.46 (d, J = 11.9 Hz, 1.31H), 3.90–3.71 (m, 6.55H), 3.64 (d, J = 8.5 Hz, 2.30H), 3.56 (d, J = 10.6 Hz, 1H), 1.27 (s, 4.20H), 1.25 (s, 3.19H).

^{13}C NMR (126 MHz, $CDCl_3$) (mixture of rotamers) δ 166.78, 157.87, 152.10, 151.12, 150.12, 149.12, 141.45, 138.47, 138.09, 138.07, 137.73, 137.26, 136.99, 135.84, 128.53, 128.50, 128.44, 128.33, 128.17, 127.96, 127.92, 127.89, 127.86, 127.79, 127.74, 122.51, 122.36, 116.49, 111.69, 110.45, 108.27, 95.46, 84.98, 81.13, 77.27, 75.75, 75.36, 75.16, 75.05, 73.62, 68.10, 62.30, 43.74, 39.69, 28.83, 28.65.

HRMS (ESI) m/z calcd for $C_{57}H_{58}N_5O_8$ $[M+H]^+$ 940.4280; **found**: 940.4280.

Compound GA93

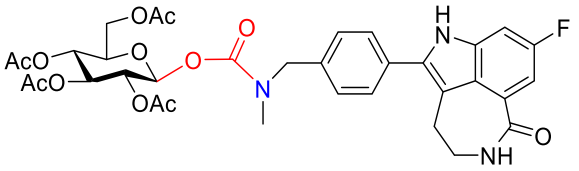

Compound **GA93** was synthesized according to the *General procedure* using amine **A71** (42 mg, 0.1 mmol, 1.0 eq) and glycosyl halide **G2** (83 mg, 0.2 mmol, 2.0 eq) in presence of K_2CO_3 (21 mg, 0.15 mmol, 1.5 eq). After the reaction, the mixture was purified by silica gel flash chromatography (petroleum ether/EtOAc = 1:1 to 1:5) to give **GA93** as an orange solid (60 mg, 86%).

$[\alpha]_D^{25}$ = -37.1 (c 1.0, $CHCl_3$).

^{1}H NMR (600 MHz, $CDCl_3$) (mixture of rotamers) δ 9.70 (s, 0.45H), 9.66 (s, 0.43H), 7.66–7.59 (m, 1H), 7.57–7.42 (m, 3H), 7.33–7.23 (m, 2H), 7.22–7.18 (m, 1H), 5.71–5.65 (m, 1H), 5.28–5.20 (m, 1H), 5.19–5.06 (m, 2H), 4.56–4.22 (m, 3H), 4.15–4.05 (m, 1H), 3.84–3.78 (m, 1H), 3.54 (s, 2H), 3.08 (s, 2H), 2.89 (s, 1.48H), 2.82 (s, 1.58H), 2.06–1.96 (m, 11.23H), 1.87 (s, 1.46H).

^{13}C NMR (151 MHz, $CDCl_3$) (mixture of rotamers) δ 170.93, 170.81, 170.74, 170.15,

170.12, 169.55, 169.48, 169.42, 160.16, 158.58, 154.43, 153.86, 136.95, 136.87, 136.53, 136.45, 135.36, 135.34, 131.27, 131.17, 128.32, 128.22, 127.83, 124.30, 123.79, 112.28, 112.23, 111.61, 111.43, 102.04, 101.87, 93.53, 93.40, 72.78, 72.65, 72.52, 72.43, 70.30, 70.15, 68.01, 67.93, 67.89, 61.61, 61.50, 52.59, 52.12, 43.15, 34.80, 34.03, 28.75, 20.75, 20.64, 20.62, 20.55.

HRMS (ESI) m/z calcd for $C_{34}H_{37}N_3O_{12}F$ $[M+H]^+$ 698.2356; **found**: 698.2358.

Compound GA94

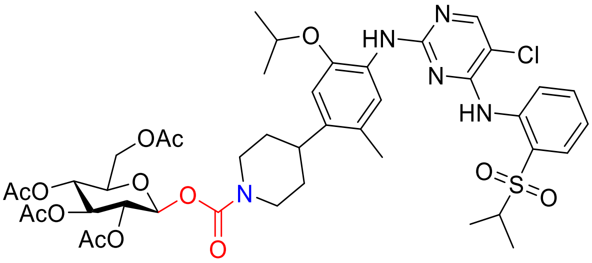

Compound **GA94** was synthesized according to the *General procedure* using amine **A72** (100 mg, 0.18 mmol, 1.0 eq) and glycosyl halide **G2** (140 mg, 0.34 mmol, 2.00 eq) in presence of Cs_2CO_3 (83 mg, 0.255 mmol, 1.5 eq). After the reaction, the mixture was purified by silica gel flash chromatography (petroleum ether/EtOAc = 4:1 to 1:1) to give **GA94** (136 mg, 81%) as a yellow solid.

$[\alpha]_D^{25}$ = 3.7 (*c* 1.0, $CHCl_3$).

^{1}H NMR (600 MHz, $CDCl_3$) (mixture of rotamers) δ 9.53–9.50 (m, 1H), 8.57 (d, *J* = 8.4 Hz, 1H), 8.15 (s, 1H), 8.02 (s, 1H), 7.95–7.90 (m, 1H), 7.64–7.59 (m, 1H), 7.56 (s, 1H), 7.29–7.23 (m, 1H), 6.71 (s, 0.51H), 6.65 (s, 0.49H), 5.69–5.61 (m, 1H), 5.32–5.26 (m, 1H), 5.20 (t, *J* = 9.0 Hz, 1H), 5.15 (t, *J* = 9.7 Hz, 1H), 4.60–4.46 (m, 1H), 4.39–4.30 (m, 2H), 4.25–4.10 (m, 2H), 3.90–3.85 (m, 1H), 3.29–3.22 (m, 1H), 3.00–2.73 (m, 3H), 2.16 (s, 3H), 2.10 (s, 3H), 2.06–2.00 (m, 9H), 1.82–1.75 (m, 2H), 1.69–1.61 (m, 2H), 1.39–1.34 (m, 6H), 1.32 (d, *J* = 6.9 Hz, 6H).

^{13}C NMR (151 MHz, $CDCl_3$) (mixture of rotamers) δ 170.79, 170.74, 170.22, 169.62, 169.54, 169.34, 157.51, 155.49, 155.38, 152.85, 152.73, 144.91, 144.84, 138.58, 136.77, 136.68, 134.76, 131.43, 128.05, 127.94, 127.19, 126.90, 125.08, 123.81, 123.30, 120.97, 120.87, 110.87, 106.00, 93.56, 93.48, 72.78, 72.74, 72.67, 72.62,

71.84, 71.76, 70.39, 70.29, 68.07, 61.63, 61.60, 55.60, 45.32, 45.16, 45.07, 44.87, 38.12, 38.05, 32.98, 32.46, 32.26, 32.19, 22.38, 20.90, 20.79, 20.75, 20.73, 19.07, 15.50.

HRMS (ESI) m/z calcd for $C_{43}H_{55}ClN_5O_{14}S$ $[M+H]^+$ 932.3156; **found**: 932.3149.

Compound GA96

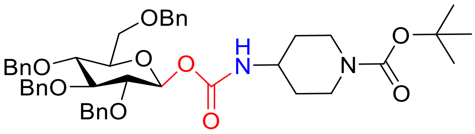

Compound **GA96** was synthesized according to the *General procedure* using amine **A33** (100 mg, 0.5 mmol, 1.0 eq) and glycosyl halide **G4** (839 mg, 1.5 mmol, 3.0 eq) in presence of Cs_2CO_3 (245 mg, 0.75 mmol, 1.5 eq). After the reaction, the mixture was purified by silica gel flash chromatography (petroleum ether/EtOAc = 4:1) to give **GA96** (261 mg, 68%) as a white liquid.

$[\alpha]_D^{25}$ = -0.04 (*c* 0.1, CH_2Cl_2).

^{1}H NMR (600 MHz, $CDCl_3$) (mixture of rotamers) δ 7.33–7.25 (m, 18H), 7.15–7.11 (m, 2H), 5.54 (d, *J* = 8.2 Hz, 1H), 4.89 (d, *J* = 10.9 Hz, 1H), 4.84–4.79 (m, 2H), 4.78–4.71 (m, 2H), 4.63 (d, *J* = 12.1 Hz, 1H), 4.57–4.51 (m, 2H), 4.47 (d, *J* = 12.1 Hz, 1H), 4.09–3.95 (m, 2H), 3.77–3.70 (m, 4H), 3.68–3.61 (m, 1H), 3.60–3.51 (m, 2H), 2.89–2.79 (m, 2H), 1.91 (tt, *J* = 8.7, 3.5 Hz, 2H), 1.46 (s, 9H).

^{13}C NMR (151 MHz, $CDCl_3$) (mixture of rotamers) δ 154.81, 153.53, 138.52, 138.40, 138.18, 138.05, 128.56, 128.53, 128.49, 128.18, 128.10, 128.06, 128.00, 127.90, 127.87, 127.86, 127.82, 95.14, 84.97, 81.30, 79.88, 75.90, 75.33, 75.14, 75.12, 73.62, 68.21, 48.61, 32.30, 28.56.

HRMS (ESI) m/z calcd for $C_{45}H_{58}N_3O_9$ $[M+NH_4]^+$ 784.4172; **found**: 784.4168.

Compound GA97

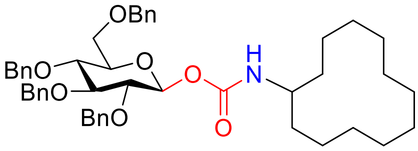

Compound **GA97** was synthesized according to the *General procedure* using amine

A34 (183 mg, 1.0 mmol, 1.0 eq) and glycosyl halide **G4** (1680 mg, 3.0 mmol, 3.0 eq) in presence of K_2CO_3 (207 mg, 1.5 mmol, 1.5 eq). After the reaction, the mixture was purified by silica gel flash chromatography (petroleum ether/EtOAc = 8:1). The crude product was further purified by silica gel flash chromatography (toluene/EtOAc = 25:1) to give **GA97** as a white solid (292 mg, 39%).

$[\alpha]_D^{25}$ = -7.2 (*c* 1.0, $CHCl_3$).

^{1}H NMR (500 MHz, $CDCl_3$) δ 7.43–7.20 (m, 18H), 7.19–7.11 (m, 2H), 5.60 (d, *J* = 8.2 Hz, 1H), 4.93 (d, *J* = 10.8 Hz, 1H), 4.88–4.80 (m, 2H), 4.77 (s, 2H), 4.67 (d, *J* = 12.2 Hz, 1H), 4.58–4.46 (m, 3H), 3.86–3.70 (m, 5H), 3.64 – 3.52 (m, 2H), 1.67–1.57 (m, 2H), 1.49–1.24 (m, 20H).

^{13}C NMR (126 MHz, $CDCl_3$) δ 153.87, 138.55, 138.34, 138.17, 138.07, 128.51, 128.49, 128.43, 128.19, 128.06, 128.03, 128.00, 127.85, 127.82, 127.79, 127.74, 95.10, 84.94, 81.31, 75.85, 75.29, 75.10, 75.07, 73.56, 68.13, 48.01, 30.63, 30.54, 23.88, 23.84, 23.71, 23.62, 23.57, 23.55, 23.41, 21.51.

HRMS (ESI) m/z calcd for $C_{47}H_{59}NO_7Na$ [M+Na]+ 722.4184; **found**: 722.4185.

Compound GA98

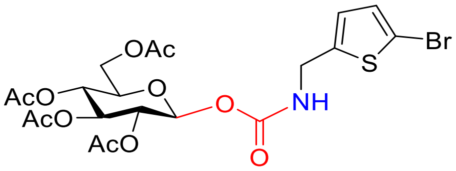

Compound **GA98** was synthesized according to the *General procedure* using amine **A36** (48 mg, 0.25 mmol, 1.0 eq) and glycosyl halide **G1** (183 mg, 0.5 mmol, 2.0 eq) in presence of Cs_2CO_3 (123 mg, 0.375 mmol, 1.5 eq). After the reaction, the mixture was purified by silica gel flash chromatography (petroleum ether/EtOAc = 1:1 to 1:3) to give **GA98** as a white liquid (41 mg, 29%).

$[\alpha]_D^{25}$ = -4.4 (*c* 1.0, $CHCl_3$).

^{1}H NMR (500 MHz, $CDCl_3$) (mixture of rotamers) δ 6.87 (d, *J* = 3.8 Hz, 1H), 6.71 (d, *J* = 3.7 Hz, 1H), 5.67 (d, *J* = 8.3 Hz, 1H), 5.49 (td, *J* = 6.1, 2.0 Hz, 1H), 5.24 (t, *J* = 9.5 Hz, 1H), 5.15–5.02 (m, 2H), 4.51–4.35 (m, 2H), 4.29 (dd, *J* = 12.5, 4.5 Hz, 1H), 4.13–4.05 (m, 1H), 3.83 (ddd, *J* = 10.2, 4.4, 2.2 Hz, 1H), 2.06 (s, 3H), 2.01 (s, 3H),

2.00 (s, 3H), 1.99 (s, 3H).

^{13}C NMR (126 MHz, $CDCl_3$) (mixture of rotamers) δ 170.75, 170.15, 169.60, 169.55, 153.67, 142.03, 129.71, 126.67, 112.11, 93.09, 72.80, 72.58, 70.28, 67.89, 61.55, 40.15, 20.82, 20.70, 20.66.

HRMS (ESI) m/z calcd for $C_{20}H_{24}NO_{11}SBrNa$ [M+Na]+ 588.0146; **found**: 590.0134.

Compound GA99

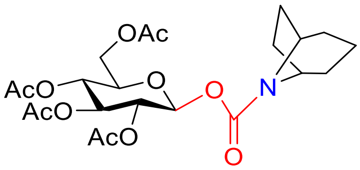

Compound **GA99** was synthesized according to the *General procedure* using amine **A35** (148 mg, 1.0 mmol, 1.0 eq) and glycosyl halide **G2** (822 mg, 2.0 mmol, 2.0 eq) in presence of Cs_2CO_3 (490 mg, 1.5 mmol, 1.5 eq). After the reaction, the mixture was purified by silica gel flash chromatography (petroleum ether/EtOAc = 4:1 to 3:1) to give **GA99** as a yellow liquid (273 mg, 56%).

$[\alpha]_D^{25}$ = -8.2 (*c* 1.0, $CHCl_3$).

^{1}H NMR (500 MHz, $CDCl_3$) (mixture of rotamers) δ 5.63–5.58 (m, 1H), 5.26–5.18 (m, 1H), 5.15–5.05 (m, 2H), 4.33–4.24 (m, 1H), 4.24–4.11 (m, 2H), 4.11–4.03 (m, 1H), 3.85–3.78 (m, 1H), 2.05–2.01 (m, 3H), 2.00–1.94 (m, 9H), 1.94–1.79 (m, 2H), 1.75–1.58 (m, 4H), 1.54–1.35 (m, 4H).

^{13}C NMR (126 MHz, $CDCl_3$) (mixture of rotamers) δ 170.64, 170.08, 169.48, 169.47, 169.37, 169.19, 150.68, 150.24, 92.93, 92.84, 72.75, 72.41, 72.36, 70.29, 70.16, 68.12, 67.98, 61.58, 61.51, 54.47, 54.42, 54.19, 31.39, 31.32, 30.59, 30.50, 28.24, 28.02, 27.49, 27.44, 20.74, 20.61, 20.59, 16.61, 16.56.

HRMS (ESI) m/z calcd for $C_{22}H_{31}NO_{11}Na$ $[M+Na]^+$ 508.1789; **found**: 508.1796.

Compound GA100

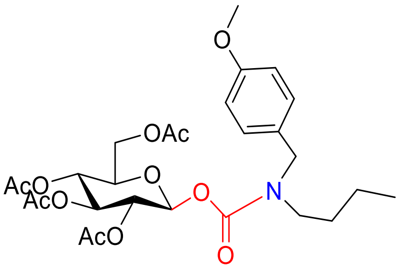

Compound **GA100** was synthesized according to the *General procedure* using amine **A37** (1000 mg, 5.17 mmol, 1.0 eq) and glycosyl halide **G2** (4252 mg, 10.34 mmol, 2.0 eq) in presence of Cs_2CO_3 (2527 mg, 7.755 mmol, 1.5 eq). After the reaction, the mixture was purified by silica gel flash chromatography (petroleum ether/EtOAc = 4:1 to 2:1) to give **GA100** as a white liquid (2797 mg, 95%).

$[\alpha]_D^{25}$ = 1.4 (*c* 0.1, CH_2Cl_2).

^{1}H NMR (600 MHz, $CDCl_3$) (mixture of rotamers) δ 7.20–7.15 (m, 1H), 7.10–7.05 (m, 1H), 6.87–6.80 (m, 2H), 5.74–5.69 (m, 1H), 5.28–5.22 (m, 1H), 5.21–5.11 (m, 2H), 4.50–4.44 (m, 1H), 4.36–4.29 (m, 1.50H), 4.30–4.21 (m, 0.62H), 4.16–4.08 (m, 1H), 3.87–3.82 (m, 1H), 3.80–3.77 (m, 3H), 3.33–3.20 (m, 1H), 3.15–3.08 (m, 0.42H), 3.03–2.96 (m, 0.48H), 2.10–2.06 (m, 3H), 2.06–1.96 (m, 9H), 1.49 (p, *J* = 7.5 Hz, 1H), 1.45–1.30 (m, 1H), 1.29–1.18 (m, 2H), 0.91–0.84 (m, 3H).

^{13}C NMR (151 MHz, $CDCl_3$) (mixture of rotamers) δ 170.79, 170.25, 169.59, 169.35, 159.24, 159.12, 154.38, 153.75, 129.48, 129.33, 129.29, 128.74, 114.10, 93.34, 93.32, 73.09, 73.07, 72.55, 72.52, 70.35, 70.24, 68.10, 61.65, 61.62, 55.41, 50.25, 49.55, 46.84, 46.12, 30.20, 29.55, 20.87, 20.72, 20.60, 20.10, 19.97, 13.90, 13.87.

HRMS (ESI) m/z calcd for $C_{27}H_{37}NO_{12}Na$ $[M+Na]^+$ 590.2206; **found**: 590.2208.

Compound GA101

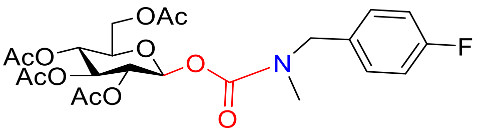

Compound **GA101** was synthesized according to the *General procedure* using amine **A41** (106 mg, 0.76 mmol, 1.0 eq) and glycosyl halide **G2** (625 mg, 1.52 mmol, 2.0 eq) in presence of Cs_2CO_3 (37 mg, 1.14 mmol, 1.5 eq). After the reaction, the mixture was purified by silica gel flash chromatography (petroleum ether/EtOAc = 4:1 to 2:1) to

give **GA101** (289 mg, 74%) as a white liquid.

$[\alpha]_D^{25}$ = -13.7 (*c* 0.1, CH_2Cl_2).

^{1}H NMR (600 MHz, $CDCl_3$) (mixture of rotamers) δ 7.24–7.18 (m, 1H), 7.17–7.11 (m, 1H), 7.04–6.98 (m, 2H), 5.71–5.67 (m, 1H), 5.31–5.24 (m, 1H), 5.22–5.11 (m, 2H), 4.51 (d, *J* = 15.6 Hz, 0.46H), 4.47–4.38 (m, 1H), 4.37–4.26 (m, 1.51H), 4.17–4.10 (m, 1H), 3.89–3.93 (m, 1H), 2.89 (s, 1.42H), 2.81 (s, 1.42H), 2.09 (s, 3H), 2.04–1.99 (m, 7.56H), 1.92 (s, 1.40H).

^{13}C NMR (151 MHz, $CDCl_3$) (mixture of rotamers) δ 170.77, 170.22, 169.60, 169.58, 169.45, 169.38, 162.35 (d, *J* = 246.0 Hz), 162.25 (d, *J* = 246.0 Hz), 154.35, 153.80, 132.54, 132.52, 129.83, 129.77, 129.22, 129.17, 115.58 (d, *J* = 21.5 Hz), 115.56 (d, *J* = 21.5 Hz), 93.56, 93.49, 77.37, 77.16, 76.95, 72.95, 72.80, 72.68, 72.58, 70.39, 70.18, 68.07, 68.05, 61.62, 61.57, 52.30, 51.84, 34.63, 33.81, 20.88, 20.73, 20.63.

HRMS (ESI) m/z calcd for $C_{23}H_{28}FNO_{11}Na$ $[M+Na]^+$ 536.1545; **found**: 536.1539.

Compound GA102

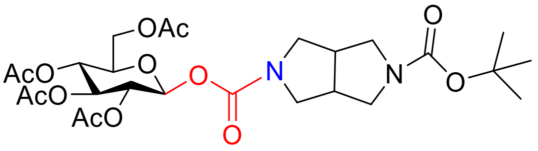

Compound **GA102** was synthesized according to the *General procedure* using amine **A38** (200 mg, 0.94 mmol, 1.0 eq) and glycosyl halide **G2** (773 mg, 1.88 mmol, 2.0 eq in presence of Cs_2CO_3 (460 mg, 1.41 mmol, 1.5 eq)). After the reaction, the mixture was purified by silica gel flash chromatography (petroleum ether/EtOAc = 4:1 to 2:1 to 1:1) to give **GA102** (419 mg, 76%) as a white solid.

$[\alpha]_D^{25}$ = -3.9 (*c* 0.1, CH_2Cl_2).

^{1}H NMR (600 MHz, $CDCl_3$) δ 5.65–5.59 (m, 1H), 5.28–5.22 (m, 1H), 5.19–5.05 (m, 2H), 4.34–4.27 (m, 1H), 4.14–4.08 (m, 1H), 3.86–3.81 (m, 1H), 3.70–3.44 (m, 4H), 3.38–3.11 (m, 4H), 2.89–2.80 (m, 2H), 2.09–2.06 (m, 3H), 2.04–2.00 (m, 9H), 1.46–1.42 (m, 9H).

^{13}C NMR (151 MHz, $CDCl_3$) δ 170.79, 170.76, 170.18, 169.59, 169.49, 154.52, 154.49, 152.14, 93.14, 79.83, 72.82, 72.79, 72.57, 70.36, 70.24, 68.03, 61.62, 50.31,

50.24, 50.01, 49.87, 49.72, 49.40, 42.42, 41.54, 40.64, 28.59, 20.89, 20.88, 20.82, 20.80, 20.73, 20.71.

HRMS (ESI) m/z calcd for $C_{26}H_{38}N_2O_{13}Na$ $[M+Na]^+$ 609.2260; **found**: 609.2266.

Compound GA103

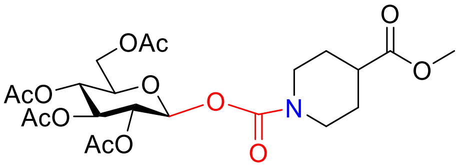

Compound **GA103** was synthesized according to the *General procedure* using amine **A39** (0.27 mL, 2.0 mmol, 1.0 eq) and glycosyl halide **G2** (1644 mg, 4.0 mmol, 2.0 eq) in presence of Cs_2CO_3 (980 mg, 3.0 mmol, 1.5 eq). After the reaction, the mixture was purified by silica gel flash chromatography (petroleum ether/EtOAc = 2:1) to give **GA103** as a brown solid (622 mg, 60%).

$[\alpha]_D^{25}$ = 15.9 (*c* 1.0, $CHCl_3$).

^{1}H NMR (500 MHz, $CDCl_3$) (mixture of rotamers) δ 5.63–5.57 (m, 1H), 5.31–5.20 (m, 1H), 5.18–5.05 (m, 2H), 4.34–4.24 (m, 1H), 4.15–4.01 (m, 2H), 3.98–3.89 (m, 1H), 3.86–3.79 (m, 1H), 3.67 (s, 3H), 3.02–2.80 (m, 2H), 2.47 (dp, *J* = 10.3, 6.6, 6.1 Hz, 1H), 2.09–2.05 (m, 4H), 2.04–1.98 (m, 8H), 1.95–1.79 (m, 2H), 1.76–1.55 (m, 2H).

^{13}C NMR (126 MHz, $CDCl_3$) (mixture of rotamers) δ 174.72, 174.65, 170.78, 170.19, 169.59, 169.48, 152.70, 152.57, 93.37, 72.73, 72.63, 72.51, 70.19, 70.14, 67.99, 61.54, 52.00, 43.56, 43.47, 43.34, 40.64, 27.99, 27.82, 27.72, 27.61, 20.85, 20.70, 20.68.

HRMS (ESI) m/z calcd for $C_{22}H_{31}NO_{13}Na$ $[M+Na]^+$ 540.1688; **found**: 540.1683.

Compound GA104

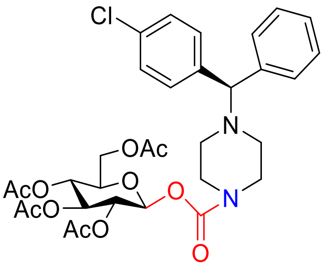

Compound **GA104** was synthesized according to the *General procedure* using amine

A40 (200 mg, 0.7 mmol, 1.0 eq) and glycosyl halide **G2** (576 mg, 1.4 mmol, 2.0 eq) in presence of Cs_2CO_3 (342 mg, 1.05 mmol, 1.5 eq). After the reaction, the mixture was purified by silica gel flash chromatography (petroleum ether/EtOAc = 4:1 to 3:1 to 1:1) to give **GA104** as a white solid (362 mg, 78%).

$[\alpha]_D^{25}$ = -12.5 (*c* 0.1, CH_2Cl_2).

^{1}H NMR (600 MHz, $CDCl_3$) δ 7.36–7.31 (m, 4H), 7.30–7.23 (m, 4H), 7.22–7.18 (m, 1H), 5.59 (d, *J* = 8.3 Hz, 1H), 5.26 (t, *J* = 9.5 Hz, 1H), 5.12 (ddd, *J* = 12.6, 9.9, 8.8 Hz, 2H), 4.31 (dd, *J* = 12.5, 4.2 Hz, 1H), 4.21 (s, 1H), 4.10 (dd, *J* = 12.5, 2.2 Hz, 1H), 3.84 (ddd, *J* = 10.1, 4.2, 2.2 Hz, 1H), 3.59–3.32 (m, 4H), 2.47–2.30 (m, 3H), 2.23 (ddd, *J* = 11.2, 6.9, 3.1 Hz, 1H), 2.08 (s, 3H), 2.02 (s, 3H), 2.00 (s, 6H).

^{13}C NMR (151 MHz, $CDCl_3$) δ 170.77, 170.18, 169.58, 169.38, 152.68, 141.58, 140.78, 133.00, 129.23, 128.95, 128.89, 127.88, 127.58, 93.41, 75.27, 72.67, 72.60, 70.20, 68.04, 61.58, 51.47, 51.33, 44.32, 44.15, 20.88, 20.72, 20.70.

HRMS (ESI) m/z calcd for $C_{32}H_{37}ClN_2O_{11}Na$ $[M+Na]^+$ 683.1985; **found**: 683.1978.

Compound GA105

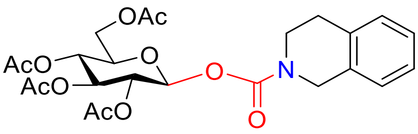

Compound **GA105** was synthesized according to the *General procedure* using amine **A42** (266 mg, 2.0 mmol, 1.0 eq) and glycosyl halide **G2** (904 mg, 2.2 mmol, 1.1 eq) in presence of Cs_2CO_3 (980 mg, 3.0 mmol, 1.5 eq). After the reaction, the mixture was purified by silica gel flash chromatography (petroleum ether/EtOAc = 3:1) to give **GA105** as a white solid (626 mg, 62%).

$[\alpha]_D^{25}$ = 13.6 (*c* 1.0, $CHCl_3$).

^{1}H NMR (600 MHz, $CDCl_3$) (mixture of rotamers) δ 7.22–7.16 (m, 2H), 7.15–7.09 (m, 2H), 5.70–5.67 (m, 1H), 5.31–5.25 (m, 1H), 5.24–5.19 (m, 1H), 5.17–5.10 (m, 1H), 4.70–4.46 (m, 2H), 4.34–4.29 (m, 1H), 4.13–4.09 (m, 1H), 3.88–3.85 (m, 1H), 3.77–3.57 (m, 2H), 2.94–2.71 (m, 2H), 2.08 (s, 3H), 2.03–2.02 (m, 3H), 2.02–2.01 (m, 4.45H), 1.93 (s, 1.37H).

^{13}C NMR (151 MHz, $CDCl_3$) (mixture of rotamers) δ 170.78, 170.20, 170.19, 169.60, 169.58, 169.52, 169.50, 153.08, 152.93, 134.35, 134.32, 132.91, 132.69, 128.83, 128.74, 126.89, 126.64, 126.62, 126.47, 126.46, 93.39, 72.82, 72.78, 72.60, 72.57, 70.27, 70.26, 68.08, 68.03, 61.62, 61.57, 45.99, 45.82, 41.98, 41.93, 28.93, 28.54, 20.87, 20.76, 20.74, 20.71, 20.65.

HRMS (ESI) m/z calcd for $C_{24}H_{29}NO_{11}Na$ $[M+Na]^+$ 530.1633; **found**: 530.1637.

Compound GA106

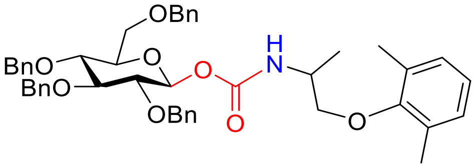

Compound **GA106** was synthesized according to the *General procedure* using amine **A73** (100 mg, 0.56 mmol, 1.0 eq) and glycosyl halide **G4** (934 mg, 1.67 mmol, 3.0 eq) in presence of Cs_2CO_3 (365 mg, 1.12 mmol, 1.5 eq). After the reaction, the mixture was purified by silica gel flash chromatography (petroleum ether/EtOAc = 10:1 to 8:1) to give **GA106** as a white solid (278 mg, 66%).

$[\alpha]_D^{25}$ = -2.0 (*c* 0.1, CH_2Cl_2).

^{1}H NMR (600 MHz, $CDCl_3$) (mixture of rotamers) δ 7.35–7.21 (m, 18H), 7.16–7.11 (m, 2H), 7.01–6.88 (m, 3H), 5.61–5.55 (m, 1H), 5.18 (d, J = 8.4 Hz, 0.44H), 5.09 (d, J = 8.2 Hz, 0.56H), 4.94–4.70 (m, 5H), 4.67–4.60 (m, 1H), 4.56–4.45 (m, 2H), 4.15–4.03 (m, 1H), 3.81–3.69 (m, 6H), 3.60–3.53 (m, 2H), 2.26 (s, 3H), 2.21 (s, 3H), 1.45–1.38 (m, 3H).

^{13}C NMR (151 MHz, $CDCl_3$) (mixture of rotamers) δ 155.02, 153.94, 153.83, 138.60, 138.57, 138.39, 138.23, 138.11, 130.97, 130.85, 129.11, 128.55, 128.53, 128.49, 128.45, 128.28, 128.09, 128.04, 128.02, 127.89, 127.82, 127.80, 124.25, 124.23, 95.46, 95.24, 85.05, 84.89, 81.19, 75.88, 75.38, 75.36, 75.13, 74.98, 74.03, 73.83, 73.68, 73.62, 68.24, 68.20, 47.52, 47.50, 18.05, 18.01, 16.44, 16.29.

HRMS (ESI) m/z calcd for $C_{46}H_{51}NO_8Na$ $[M+Na]^+$ 768.3509; **found**: 768.3507.

Compound GA107

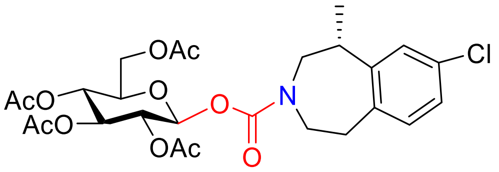

Compound **GA107** was synthesized according to the *General procedure* using amine **A74** (215 mg, 1.10 mmol, 1.0 eq) and glycosyl halide **G2** (905 mg, 2.20 mmol, 2,0 eq) in presence of Cs_2CO_3 (538 mg, 1.65 mmol, 1.5 eq). After the reaction, the mixture was purified by silica gel flash chromatography (petroleum ether/EtOAc = 4:1 to 2:1 to 1:1) to give **GA107** as a white solid (611 mg, 97%).

$[\alpha]_D^{25}$ = -13.8 (*c* 0.1, CH_2Cl_2).

^{1}H NMR (600 MHz, $CDCl_3$) (mixture of rotamers) δ 7.15–7.06 (m, 2H), 7.03–6.99 (m, 1H), 5.69–5.66 (m, 1H), 5.32–5.24 (m, 1H), 5.24–5.18 (m, 1H), 5.16–5.11 (m, 1H), 4.36–4.25 (m, 1H), 4.13–4.09 (m, 1H), 3.87–3.75 (m, 2H), 3.72–3.53 (m, 1.50H), 3.46–3.24 (m, 1.36H), 3.16–2.97 (m, 1.52H), 2.95–2.88 (m, 0.44H), 2.87–2.75 (m, 1H), 2.09–2.06 (m, 3H), 2.05–1.99 (m, 9H), 1.27 (d, *J* = 7.2 Hz, 3H).

^{13}C NMR (151 MHz, $CDCl_3$) (mixture of rotamers) δ 170.77, 170.73, 170.20, 170.18, 169.61, 169.56, 169.44, 153.54, 146.16, 146.07, 137.64, 137.09, 132.57, 132.55, 132.01, 131.87, 128.52, 126.55, 126.48, 93.55, 93.37, 72.89, 72.74, 72.65, 72.48, 70.37, 70.26, 68.16, 68.09, 61.62, 61.58, 51.76, 51.08, 47.11, 46.83, 41.36, 40.95, 36.17, 35.26, 20.88, 20.85, 20.76, 20.73, 18.15, 17.41.

HRMS (ESI) m/z calcd for $C_{26}H_{32}ClNO_{11}Na$ $[M+Na]^+$ 592.1560; **found**: 592.1556.

Compound GA108

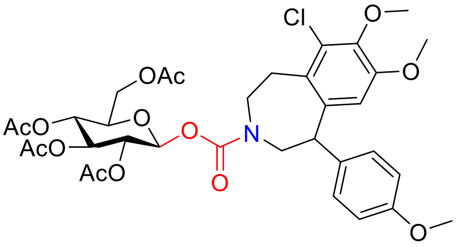

Compound **GA108** was synthesized according to the *General procedure* using amine **A75** (70 mg, 0.2 mmol, 1.0 eq) and glycosyl halide **G2** (165 mg, 0.4 mmol, 2,0 eq) in presence of Cs_2CO_3 (98 mg, 0.3 mmol, 1.5 eq). After the reaction, the mixture was

purified by silica gel flash chromatography (petroleum ether/EtOAc = 8:1 to 4:1 to 2:1 to 1:1) to give **GA108** (99 mg, 69%) as a white solid.

$[\alpha]_D^{25}$ = -3.1 (*c* 0.1, CH_2Cl_2).

^{1}H NMR (600 MHz, $CDCl_3$) (mixture of diastereomers and rotamers) δ 7.09–6.97 (m, 2H), 6.93–6.80 (m, 2H), 6.51–6.39 (m, 1H), 5.69–5.64 (m, 0.21H), 5.61–5.49 (m, 0.86H), 5.31–5.18 (m, 1.48H), 5.17–5.06 (m, 1.62H), 4.52–4.44 (m, 0.61H), 4.41–4.22 (m, 1.65H), 4.21–4.05 (m, 1.89H), 3.99 (dd, *J* = 14.7, 6.5 Hz, 0.24H), 3.86–3.76 (m, 7.22H), 3.72–3.67 (m, 2.90H), 3.59–3.48 (m, 0.92H), 3.48–3.39 (m, 1.13H), 3.35 (dt, *J* = 15.6, 4.3 Hz, 0.21H), 3.28 (dt, *J* = 15.4, 4.1 Hz, 0.52H), 3.16 (td, *J* = 11.2, 5.6 Hz, 0.22H), 3.11–3.03 (m, 0.63H), 3.00–2.92 (m, 0.23H), 2.12–2.06 (m, 2.98H), 2.06–1.98 (m, 7.65H), 1.93 (s, 0.63H), 1.84 (s, 0.45H), 1.82 (s, 1.05H).

^{13}C NMR (151 MHz, $CDCl_3$) (mixture of diastereomers and rotamers) δ 170.87, 170.80, 170.69, 170.28, 170.23, 170.21, 170.15, 169.76, 169.68, 169.65, 169.57, 169.48, 169.36, 169.31, 158.60, 158.58, 158.50, 153.88, 153.86, 153.82, 153.46, 152.05, 152.02, 151.97, 151.84, 144.23, 144.20, 144.14, 144.02, 138.31, 137.79, 137.64, 137.60, 134.34, 134.20, 134.14, 133.22, 129.22, 129.19, 129.04, 129.01, 128.88, 128.81, 128.76, 128.69, 128.09, 128.07, 128.02, 114.32, 114.10, 113.99, 113.73, 113.61, 113.50, 93.52, 93.37, 93.32, 72.87, 72.85, 72.69, 72.63, 72.58, 72.57, 72.54, 71.15, 70.44, 70.36, 70.09, 70.00, 69.94, 68.57, 68.54, 68.15, 68.10, 68.06, 67.98, 67.48, 62.06, 61.70, 61.67, 61.62, 61.52, 60.62, 60.56, 56.08, 56.05, 55.96, 55.41, 55.38, 50.96, 50.77, 50.47, 50.27, 49.68, 49.46, 49.40, 49.05, 46.17, 45.87, 45.79, 29.19, 28.67, 28.22, 27.95, 20.90, 20.87, 20.83, 20.81, 20.77, 20.73, 20.71, 20.47, 20.27.

HRMS (ESI) m/z calcd for $C_{34}H_{44}ClN_2O_{14}$ $[M+H]^+$ 739.2480; **found**: 739.2476.

Compound GA109

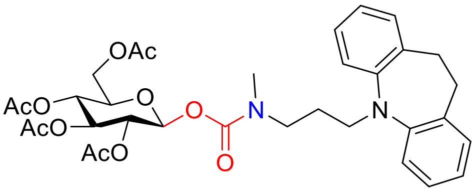

Compound **GA109** was synthesized according to the *General procedure* using amine

A76 (152 mg, 0.5 mmol, 1.0 eq) and glycosyl halide **G2** (226 mg, 0.55 mmol, 1.1 eq) in presence of Cs_2CO_3 (245 mg, 0.75 mmol, 1.5 eq). After the reaction, the mixture was purified by silica gel flash chromatography (petroleum ether/EtOAc = 3:1) to give **GA109** as a brown liquid (224 mg, 70%).

$[\alpha]_D^{25}$ = -31.9 (*c* 1.0, $CHCl_3$).

^{1}H NMR (600 MHz, $CDCl_3$) (mixture of rotamers) δ 7.17–7.01 (m, 6H), 6.96–6.88 (m, 2H), 5.63–5.57 (m, 1H), 5.28–5.22 (m, 1H), 5.18–5.04 (m, 2H), 4.33–4.25 (m, 1H), 4.11–4.06 (m, 1H), 3.89–3.65 (m, 3H), 3.32–3.27 (m, 1H), 3.27–3.22 (m, 1H), 3.18–3.12 (m, 4H), 2.77 (s, 1.47H), 2.72 (s, 1.53H), 2.08–2.05 (m, 3H), 2.04–1.96 (m, 9H), 1.84–1.76 (m, 1.52H), 1.74–1.67 (m, 0.65H).

^{13}C NMR (151 MHz, $CDCl_3$) (mixture of rotamers) δ 170.77, 170.20, 170.19, 169.60, 169.57, 169.46, 169.39, 153.76, 153.75, 148.11, 148.09, 134.38, 134.35, 130.02, 126.60, 126.56, 122.84, 122.81, 119.96, 119.93, 93.25, 72.93, 72.82, 72.51, 72.44, 70.36, 70.30, 68.15, 68.04, 68.00, 61.78, 61.60, 47.93, 47.69, 47.49, 47.06, 35.09, 34.27, 32.28, 32.25, 26.48, 26.00, 20.88, 20.86, 20.73, 20.71.

HRMS (ESI) m/z calcd for $C_{33}H_{40}N_2O_{12}Na$ $[M+Na]^+$ 663.2524; **found**: 663.2527.

Compound GA110

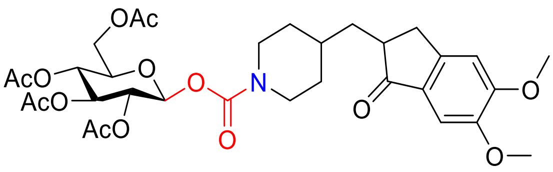

Compound **GA110** was synthesized according to the *General procedure* using amine **A77** (289 mg, 1.0 mmol, 1.0 eq) and glycosyl halide **G2** (822 mg, 2.0 mmol, 2.0 eq) in presence of Cs_2CO_3 (490 mg, 1.5 mmol, 1.5 eq). After the reaction, the mixture was purified by silica gel flash chromatography (petroleum ether/EtOAc = 2:1) to give **GA110** as a white solid (451mg, 68%).

$[\alpha]_D^{25}$ = -22.5 (*c* 1.0, $CHCl_3$).

^{1}H NMR (600 MHz, $CDCl_3$) (mixture of diastereomers) δ 7.14 (s, 1H), 6.84 (s, 2H), 5.64–5.57 (m, 1H), 5.28–5.21 (m, 1H), 5.18–5.07 (m, 2H), 4.33–4.25 (m, 1H), 4.20–3.99 (m, 3H), 3.94 (s, 3H), 3.88 (s, 3H), 3.86–3.81 (m, 1H), 3.26–3.19 (m, 1H),

2.88–2.72 (m, 2H), 2.70–2.63 (m, 2H), 2.10–1.96 (m, 12H), 1.92–1.64 (m, 5H), 1.40–1.27 (m, 2H).

^{13}C NMR (151 MHz, $CDCl_3$) (mixture of diastereomers) δ 207.46, 170.76, 170.17, 169.58, 169.47, 169.44, 155.70, 155.67, 152.80, 152.56, 149.61, 148.70, 130.98, 129.24, 128.88, 107.43, 104.48, 93.35, 93.32, 72.78, 72.67, 72.50, 70.22, 70.20, 68.05, 61.59, 61.56, 56.32, 56.19, 45.09, 45.07, 44.52, 44.49, 44.41, 44.20, 38.62, 38.57, 34.30, 33.51, 33.49, 33.26, 32.95, 32.56, 32.40, 32.18, 31.99, 31.73, 31.60, 31.39, 31.33, 20.84, 20.83, 20.71, 20.68, 20.66.

HRMS (ESI) m/z calcd for $C_{32}H_{41}NO_{14}Na$ $[M+Na]^+$ 686.2419; **found**: 686.2420.

Compound GA111

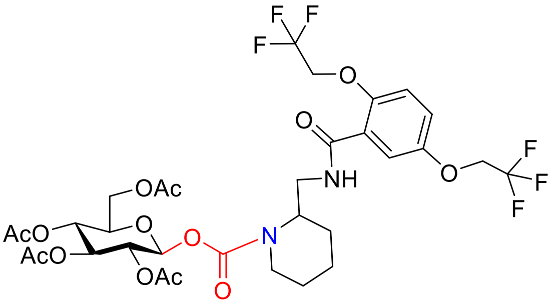

Compound **GA111** was synthesized according to the *General procedure* using amine **A78** (200 mg, 0.48 mmol, 1.0 eq) and glycosyl halide **G2** (395 mg, 0.96 mmol, 2.0 eq) in presence of Cs_2CO_3 (235 mg, 0.72 mmol, 1.5 eq). After the reaction, the mixture was purified by silica gel flash chromatography (petroleum ether/EtOAc = 4:1 to 2:1 to 1:1). The crude product was purified by silica gel flash chromatography (dichloromethane/MeOH = 200:1 to 100:1) to give **GA111** (273 mg, 72%) as a white solid.

$[\alpha]_D^{25}$= -6.2 (*c* 0.1, CH_2Cl_2).

^{1}H NMR (600 MHz, $CDCl_3$) (mixture of diastereomers and rotamers) δ 7.79–7.53 (m, 2H), 7.12–7.02 (m, 1H), 6.98–6.87 (m, 1H), 5.69–5.39 (m, 1H), 5.32–4.94 (m, 3H), 4.65–4.04 (m, 8H), 4.02 – 3.57 (m, 4H), 3.13–2.78 (m, 1H), 2.14–1.98 (m, 11.75H), 1.94–1.82 (m, 1.67H), 1.79–1.60 (m, 6H).

^{13}C NMR (151 MHz, $CDCl_3$) (mixture of diastereomers and rotamers) δ 170.73, 170.22, 169.59, 169.36, 164.22, 153.79, 153.55, 153.12, 153.05, 150.53, 123.2 (m),

120.73, 117.51, 117.18, 115.74, 115.54, 115.37, 93.46, 72.64, 72.51, 70.24, 70.03, 69.65, 68.40, 68.04, 67.73, 67.52, 67.29, 66.4 (m), 62.01, 61.58, 61.39, 51.25, 50.91, 40.71, 40.24, 39.86, 39.55, 39.21, 39.04, 29.84, 29.41, 26.44, 25.54, 25.17, 24.85, 20.85, 20.70, 20.40, 19.29, 19.17, 18.68.

HRMS (ESI) m/z calcd for $C_{32}H_{38}F_6N_2O_{14}Na$ $[M+Na]^+$ 811.2115; **found**: 811.2119.

Compound GA112

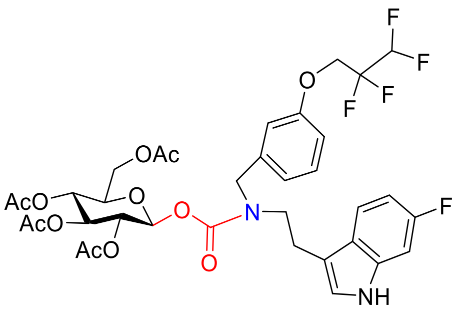

Compound **A112** was synthesized according to the *General procedure* using amine **A79** (43 mg, 0.1 mmol, 1.0 eq) and glycosyl halide **G2** (82 mg, 0.2 mmol, 2.0 eq) in presence of Cs_2CO_3 (49 mg, 0.15 mmol, 1.5 eq). After the reaction, the mixture was purified by silica gel flash chromatography (petroleum ether/EtOAc = 4:1 to 2:1) to give **GA112** (44.6 mg, 58%) as a yellow solid.

$[\alpha]_D^{25}$= 15.6 (*c* 0.1, CH_2Cl_2).

^{1}H NMR (600 MHz, $CDCl_3$) (mixture of rotamers) δ 8.07 (s, 1H), 7.47–7.33 (m, 2H), 7.26–7.21 (m, 2H), 7.05–7.01 (m, 1H), 7.00 (d, *J* = 2.2 Hz, 0.54H), 6.96 (d, *J* = 2.2 Hz, 0.48H), 6.92–6.83 (m, 1.54H), 6.82–6.76 (m, 1.48H), 6.74 (t, *J* = 2.1 Hz, 0.54H), 6.65 (t, *J* = 2.1 Hz, 0.49H), 6.17–5.93 (m, 1H), 5.76 (d, *J* = 8.3 Hz, 0.52H), 5.73 (d, *J* = 8.4 Hz, 0.48H), 5.34–5.06 (m, 3.54H), 4.49 (d, *J* = 16.0 Hz, 0.50H), 4.40–4.09 (m, 6.26H), 3.92–3.84 (m, 1H), 3.72–3.65 (m, 0.47H), 3.51–3.33 (m, 1.49H), 3.10–2.92 (m, 1H), 2.92–2.80 (m, 1H), 2.13–2.07 (m, 4.54H), 2.07–2.01 (m, 7.36H).

^{13}C NMR (151 MHz, $CDCl_3$) (mixture of rotamers) δ 170.85, 170.24, 169.64, 169.60, 169.57, 169.34, 160.19 (d, *J* = 237.9 Hz), 157.74, 154.44, 153.75, 139.44, 139.31, 136.30 (d, *J* = 12.5 Hz), 136.27 (d, *J* = 12.5 Hz), 130.10, 130.06, 124.08, 123.99, 122.72, 122.70, 122.37, 122.35, 122.08, 121.34, 119.46 (d, *J* = 30.6 Hz), 119.40 (d, *J* = 30.6 Hz), 114.62, 113.98, 113.66, 113.39, 112.93, 112.71, 109.17 (tt, J = 249.6, 34.0

Hz), 108.42 (d, J = 24.4 Hz), 108.39 (d, J = 24.3 Hz), 97.68 (d, J = 25.8 Hz), 97.63 (d, J = 26.2 Hz), 93.56, 93.49, 77.37, 77.16, 76.95, 72.97, 72.92, 72.71, 72.69, 71.15, 70.46, 70.14, 69.94, 68.57, 68.54, 68.12, 68.06, 67.49, 65.29 (t, J = 29.5 Hz), 61.57, 61.53, 51.40, 50.82, 48.37, 47.58, 29.85, 24.29, 23.48, 20.89, 20.87, 20.84, 20.77, 20.74, 20.71, 20.43.

HRMS (ESI) m/z calcd for $C_{35}H_{37}F_5N_2O_{12}Na$ $[M+Na]^+$ 795.2161; **found**: 795.2159.

9. Competition experiments

9.1 Competition experiments in Fig. 3A-a

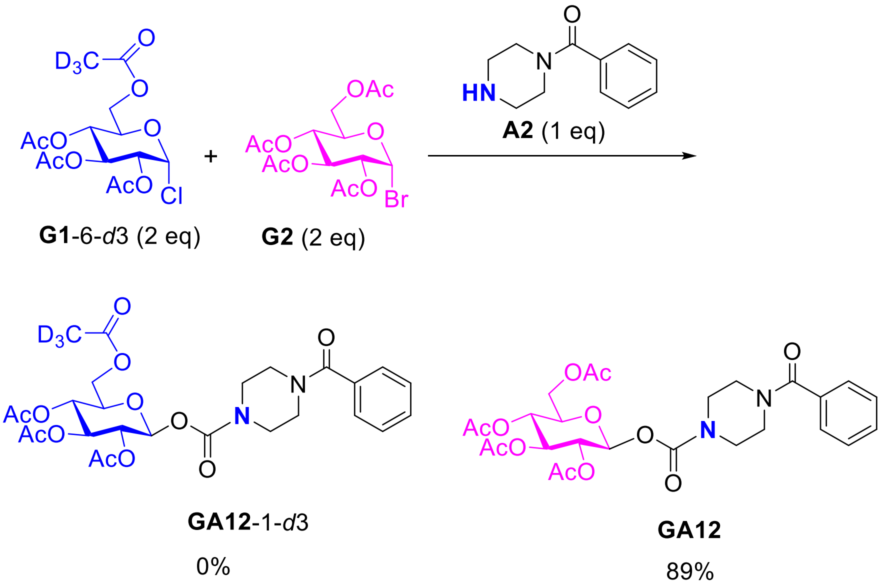

Fig. S34

An oven-dried Schlenk tube (25 ml) were charged with **A2** (48 mg, 0.25 mmol, 1.0 eq), Cs_2CO_3 (123 mg, 0.375 mmol, 1.5 eq) and a Teflon-coated magnetic stirring bar. After the mixture was evacuated and backfilled with CO_2 gas three times, DMSO (2.0 mL) was added via a syringe. The reaction mixture was allowed to stir for 2 h under a CO_2 atmosphere at RT, and then a solution of **G1**-6-*d*3 (185 mg, 0.50 mmol, 2.0 eq) and **G2** (206 mg, 0.50 mmol, 2.0 eq) in DMSO (2 mL) was added, and the reaction was further stirred at RT for 12 h. H_2O (10 mL) was added, and the mixture was extracted with CH_2Cl_2 (20 mL×3), washed with saturated NH_4Cl (50 mL), then dried over anhydrous Na_2SO_4 and filtered. The solvent was removed in vacuo and the residue was purified by silica gel column chromatography (petroleum ether/EtOAc = 1:1) to afford products **GA12** (126 mg, 89%). ^{1}H NMR and HRMS analysis indicated that no **GA12**-6-*d*3 was formed.

9.2 Competition experiments in Fig. 3A-b

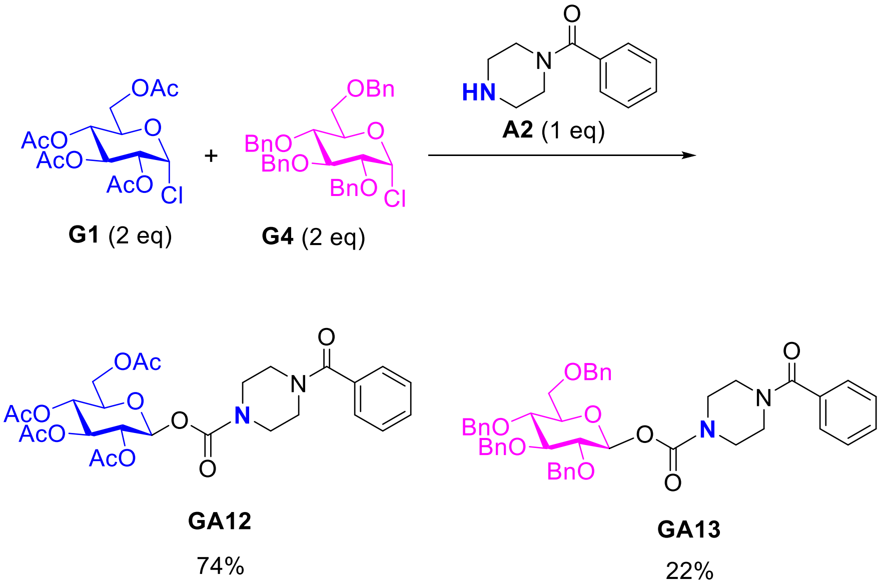

Fig. S35

An oven-dried Schlenk tube (25 ml) were charged with **A2** (38 mg, 0.20 mmol, 1.0 eq), Cs_2CO_3 (98 mg, 0.30 mmol, 1.5 eq) and a Teflon-coated magnetic stirring bar. After the mixture was evacuated and backfilled with CO_2 gas three times, DMSO (2.0 mL) was added via a syringe. The reaction mixture was allowed to stir for 2 h under a CO_2 atmosphere at RT, and then a solution of **G1** (147 mg, 0.40 mmol, 2.0 eq) and **G4** (224 mg, 0.40 mmol, 2.0 eq) in DMSO (2 mL) was added, and the reaction was further stirred at RT for 12 h. H_2O (10 mL) was added, and the mixture was extracted with CH_2Cl_2 (20 mL×3), washed with saturated NH_4Cl (50 mL), then dried over anhydrous Na_2SO_4 and filtered. The solvent was removed in vacuo and the residue was purified by silica gel column chromatography (petroleum ether/EtOAc = 2:1 to 1:1) to afford products **GA12** (84 mg, 74%) and **GA13** (33 mg, 22%).

9.3 Competition experiment in Fig. 3A-c

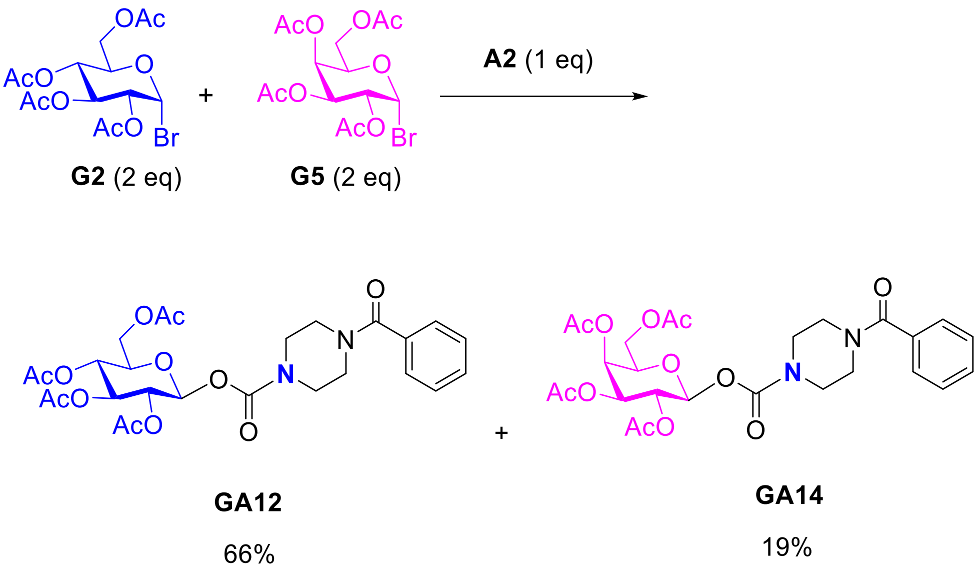

Fig. S36

An oven-dried Schlenk tube (25 mL) were charged with **A2** (95 mg, 0.50 mmol, 1.0 eq), Cs_2CO_3 (245 mg, 0.75 mmol, 1.5 eq) and a Teflon-coated magnetic stirring bar. After the mixture was evacuated and backfilled with CO_2 gas three times, DMSO (2.0 mL) was added via a syringe. The reaction mixture was allowed to stir for 2 h under a CO_2 atmosphere at RT, and then a solution of **G2** (411 mg, 1.0 mmol, 2.0 eq) and **G5** (411 mg, 1.0 mmol, 2.0 eq) in DMSO (2 mL) was added, and the reaction was further stirred at RT for 12 h. H_2O (10 mL) was added, and the mixture was extracted with CH_2Cl_2 (20 mL×3), washed with saturated NH_4Cl (50 mL), then dried over anhydrous Na_2SO_4 and filtered. The solvent was removed in vacuo and the residue was purified by silica gel column chromatography (petroleum ether/EtOAc = 1:1 to 2:3) to afford a mixture of product **GA12** and **GA13** (238 mg, 84%), ^{1}H NMR analysis revealed that the ratio of **GA12** and **GA13** was 3.55 : 1, thus the yield of **GA12** was calculated to be 66%, and the yield of **GA13** was calculated to be 19%.

9.4 Competition experiment in Fig. 3A-d

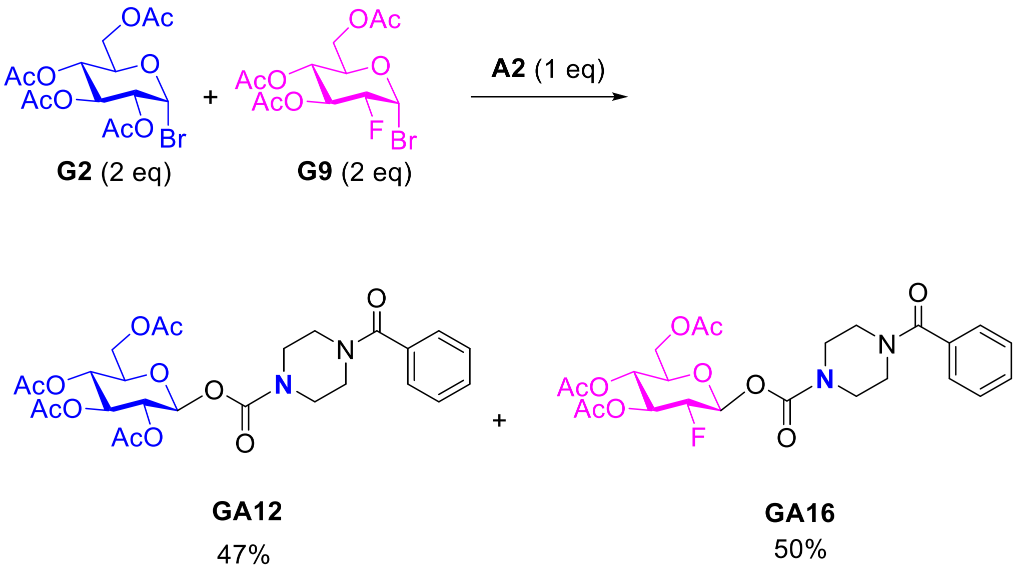

Fig. S37

An oven-dried Schlenk tube (25 ml) were charged with **A2** (48 mg, 0.25 mmol, 1.0 eq), Cs_2CO_3 (122 mg, 0.375 mmol, 1.5 eq) and a Teflon-coated magnetic stirring bar. After the mixture was evacuated and backfilled with CO_2 gas three times, DMSO (2.0 mL) was added via a syringe. The reaction mixture was allowed to stir for 2 h under a CO_2 atmosphere at RT, and then a solution of **G2** (206 mg, 0.50 mmol, 2.0 eq) and **G9** (185 mg, 0.50 mmol, 2.0 eq) in DMSO (2 mL) was added, and the reaction was further stirred at RT for 12 h. H_2O (10 mL) was added, and the mixture was extracted with CH_2Cl_2 (20 mL×3), washed with saturated NH_4Cl (50 mL), then dried over anhydrous Na_2SO_4 and filtered. The solvent was removed in vacuo and the residue was purified by silica gel column chromatography (petroleum ether/EtOAc = 1:1 to 1:3) to afford products **GA12** (67 mg, 47%) and **GA16** (66 mg, 50%).

9.5 Competition experiment in Fig. 3A-e

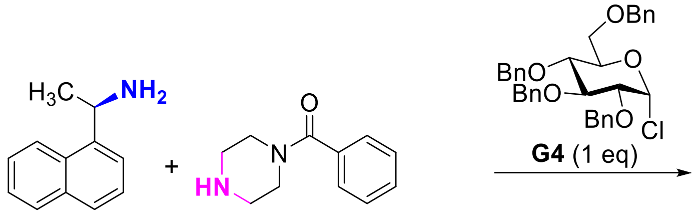

A1 (1.5 eq) **A2** (1.5 eq)

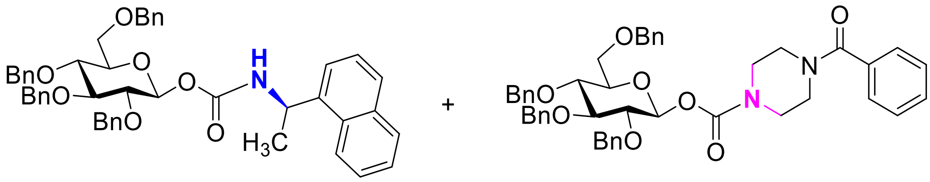

GA2
0%

GA13
46%

Fig. S38

An oven-dried Schlenk tube (25 ml) were charged with **A1** (103 mg, 0.6 mmol, 1.5 eq) and **A2** (95 mg, 0.6 mmol, 1.5 eq),Cs_2CO_3 (391 mg, 1.2 mmol, 3.0 eq) and a Teflon-coated magnetic stirring bar. After the mixture was evacuated and backfilled with CO_2 gas three times, DMSO (2.0 mL) was added via a syringe. The reaction mixture was allowed to stir for 2 h under a CO_2 atmosphere at RT, and then a solution of **G4** (224 mg, 0.40 mmol, 2.0 eq) in DMSO (2 mL) was added, and the reaction was further stirred at RT for 12 h. H_2O (10 mL) was added, and the mixture was extracted with CH_2Cl_2 (20 mL×3), washed with saturated NH_4Cl (50 mL), then dried over anhydrous Na_2SO_4 and filtered. The solvent was removed in vacuo and the residue was purified by silica gel column chromatography (petroleum ether/EtOAc = 3:1 to 1:1) to afford product **GA13** (135 mg, 45%).

10. Reactivity test of equatorial anomer of glycosyl chloride

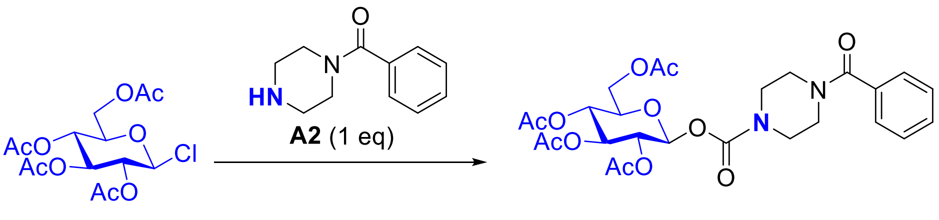

Fig. S39

An oven-dried Schlenk tube (25 ml) were charged with **A2** (57 mg, 0.30 mmol, 1.0 eq), Cs_2CO_3 (147 mg, 0.45 mmol, 1.5 eq) and a Teflon-coated magnetic stirring bar. After the mixture was evacuated and backfilled with CO_2 gas three times, DMSO (2.0 mL) was added via a syringe. The reaction mixture was allowed to stir for 2 h under a CO_2 atmosphere at RT, and then a solution of **G31** (220 mg, 0.60 mmol, 2.0 eq) in DMSO (2 mL) was added, and the reaction was further stirred at RT for 12 h. H_2O (10 mL) was added, and the mixture was extracted with CH_2Cl_2 (20 mL×3), washed with saturated NH_4Cl (50 mL), then dried over anhydrous Na_2SO_4 and filtered. The solvent was removed in vacuo and the residue was purified by silica gel column chromatography (petroleum ether/EtOAc = 1:1 to 1:3) to afford product **GA12** (27 mg, 16%) as a white solid, ^1H NMR analysis revealed that the product is a mixture of the α and β anomer (α/β = 1/0.6).

11. NMR experiments

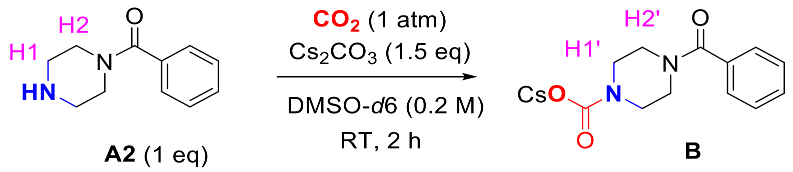

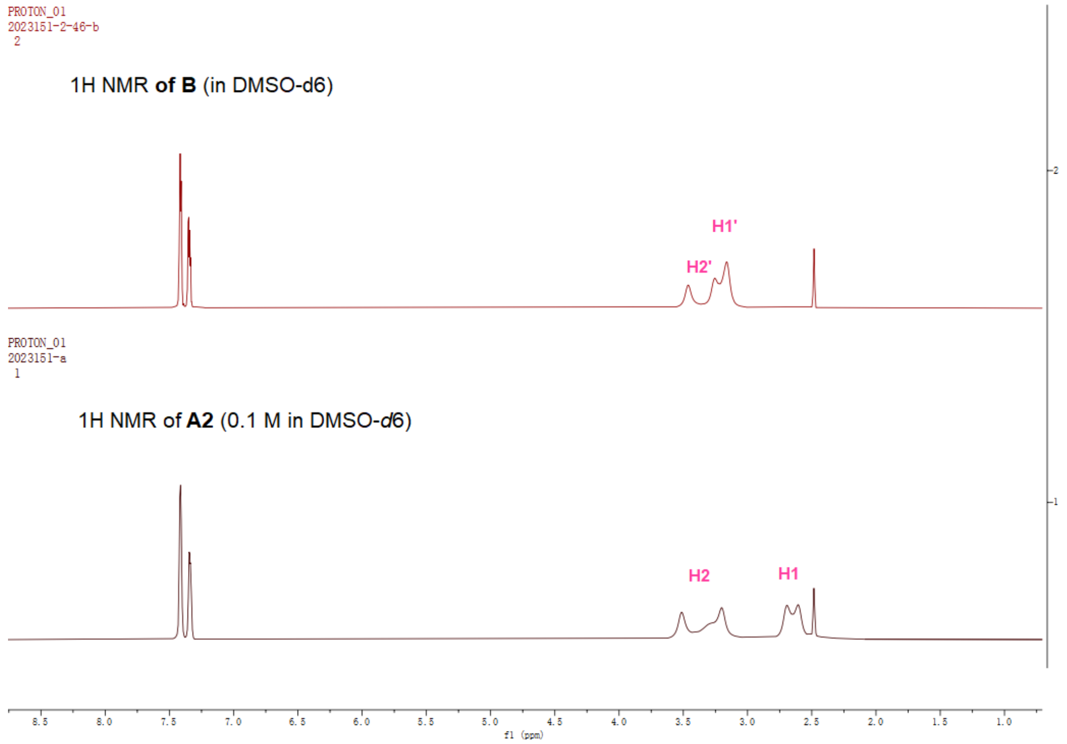

Fig. S40

12. Secondary α-deuterium kinetic isotope effects determination

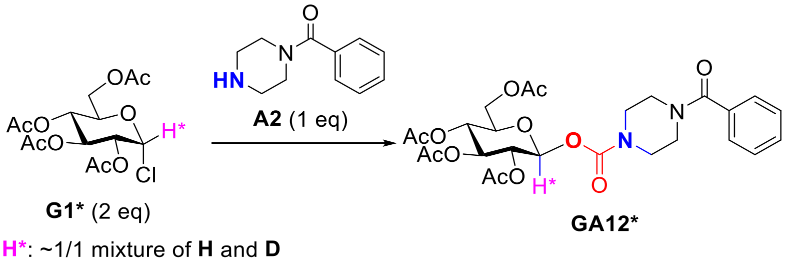

Fig. S41

An oven-dried Schlenk tube (25 ml) were charged with **A2** (95.1 mg, 0.50 mmol, 1.0 eq), Cs_2CO_3 (244.4 mg, 0.75 mmol, 1.5 eq) and a Teflon-coated magnetic stirring bar. After the mixture was evacuated and backfilled with CO_2 gas three times, DMSO (2.0 mL) was added via a syringe. The reaction mixture was allowed to stir for 2 h under a CO_2 atmosphere at RT, and then a solution of **G1*** (367.3 mg, 1.0 mmol, 2.0 eq) in DMSO (2 mL) was added, and the reaction was further stirred at RT for 18 h. H_2O (10 mL) was added, and the mixture was extracted with CH_2Cl_2 (20 mL×3), washed with saturated NH_4Cl (50 mL), then dried over anhydrous Na_2SO_4 and filtered. The solvent was removed in vacuo, an internal standard 2-bromo-5-methoxy-1,3-dimethyl-benzene (8 μL, 0.05 mmol) was added, and the yield of product **GA12*** (*F*) was calculated via ^{1}H NMR analysis. The residue was then purified by silica gel column chromatography (petroleum ether/EtOAc = 2:1 to 1:1) to afford pure products **GA12***.

The ^{1}H NMR spectra of the substrate **G1*** and the product (**GA12***) were recorded in $CDCl_3$ at 600 MHz, and the integration of the anomeric position (H-1) of **G1*** and **GA12*** (marked **A** and **C**, respectively), and the integration of the H-2 position of the sugar ring of **G1*** and **GA12*** (marked **B** and **D**, respectively) were obtained. The α-DKIE value was determined from these vales using the following equation:

$$\mathrm{KIE} = \ln(1 - F)/\ln[1 - (F\mathrm{R2}/\mathrm{R1})]$$

wherein *F* is the fractional conversion of the **G1*** (yield of **GA12***) and R2 and R1 the ratios of the H-2 position of the sugar ring to anomeric (H-1) resonances in the product **GA12*** and the glycosyl chloride **G1***, respectively.

The results were summarized in Table S5.

	Run 1	Run 2
F	46.5%	39.0%
R1	2.04	2.04
R2	2.24	2.28
KIE	0.88	0.86
KIE (mean)	0.87	

Table S5

13. Kinetic studies

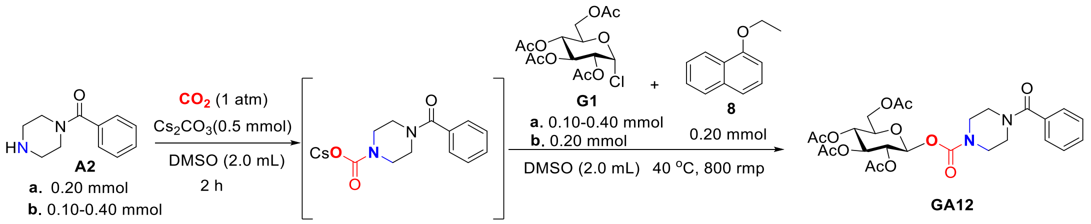

Fig. S42

System a: order in **G1**

An oven-dried Schlenk tube (25 ml) were charged with **A2** (76.1 mg, 0.40 mmol), Cs_2CO_3 (325.8 mg, 1.0 mmol) and a Teflon-coated magnetic stirring bar. After the mixture was evacuated and backfilled with CO_2 gas three times, DMSO (2.0 mL) was added via a syringe. The reaction mixture was allowed to stir for 2 h under a CO_2 atmosphere at 40 °C (oil bath), and then a solution of **G1** (73.3 mg ~ 366.8 mg, 0.2 mmol, 0.4 mmol, 0.6 mmol, 0.8 mmol) and **8** (34.5 mg, 0.2 mmol) in DMSO (2 mL) was added, and the reaction was further stirred at 40 °C. At regular intervals, 100 μL of the reaction solution from the reaction system was quench with water (0.5 mL), extracted with CH_2Cl_2 (0.5 mL) and the organic layer was analyzed by HPLC. The reaction conversion was calculated by HPLC internal standard method.

System a: order in **A2**

An oven-dried Schlenk tube (25 ml) were charged with **A2** (38 mg ~ 190.3 mg, 0.2 mmol, 0.4 mmol, 0.6 mmol, 0.8 mmol), Cs_2CO_3 (325.8 mg, 1.0 mmol) and a Teflon-coated magnetic stirring bar. After the mixture was evacuated and backfilled with CO_2 gas three times, DMSO (2.0 mL) was added via a syringe. The reaction mixture was allowed to stir for 2 h under a CO_2 atmosphere at 40 °C (oil bath), and then a solution of **G1** (146.7 mg, 0.4 mmol) and **8** (34.5 mg, 0.2 mmol) in DMSO (2 mL) was added, and the reaction was further stirred at 40 °C. At regular intervals, 100 μL of the reaction solution from the reaction system was quench with water (0.5 mL), extracted with CH_2Cl_2 (0.5 mL) and the organic layer was analyzed by HPLC. The reaction conversion was calculated by HPLC internal standard method.

System **a**, 0.2 mmol of **G1**

Time (min)	Concentration of **GA12** (mol/L)	Yield of **GA12** (%)
15	4.19E-03	4.2
30	1.02E-02	10.2
45	1.45E-02	14.5
60	1.95E-02	19.5

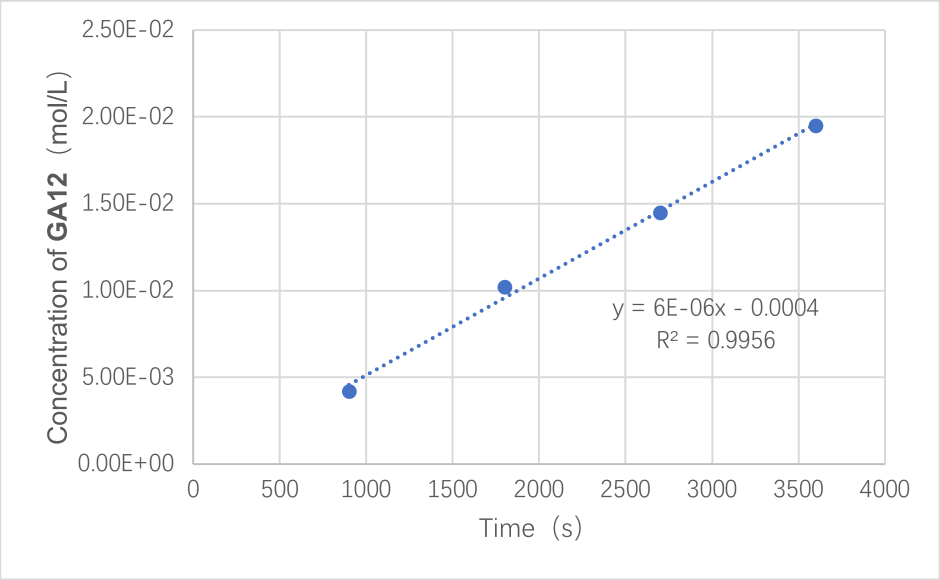

Fig. S43

System **a**, 0.4 mmol of **G1**

Time (min)	Concentration of **GA12** (mol/L)	Yield of **GA12** (%)
2	7.40E-03	7.4
4	1.48E-02	14.8
6	2.09E-02	20.9
8	2.62E-02	26.2
10	3.25E-02	32.5
12	4.11E-02	41.1

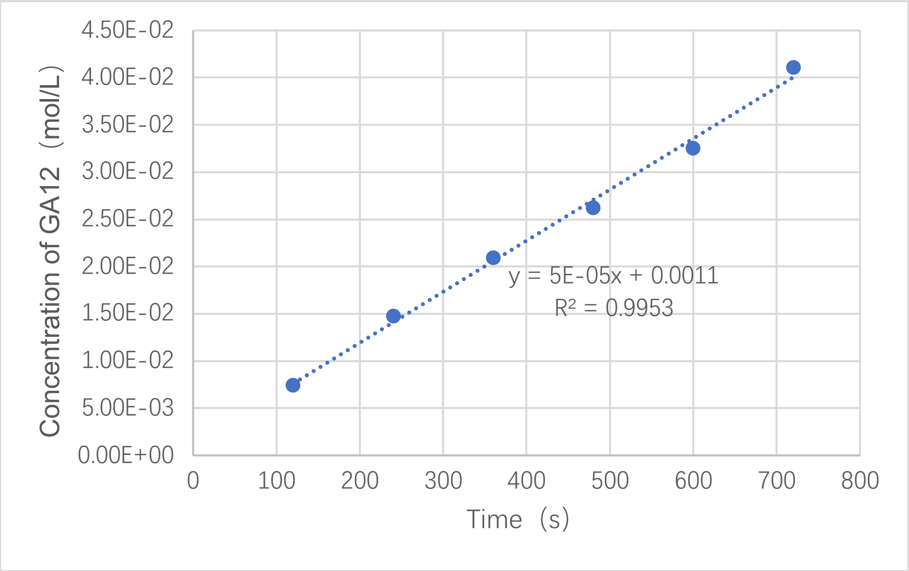

Fig. S44

System **a**, 0.6 mmol of **G1**

Time (min)	Concentration of **GA12** (mol/L)	Yield of **GA12** (%)
1	9.86E-03	9.9
3	2.13E-02	21.3
5	3.24E-02	32.4
7	4.20E-02	42.0
9	6.06E-02	60.6
11	6.64E-02	66.4
13	7.69E-02	76.9

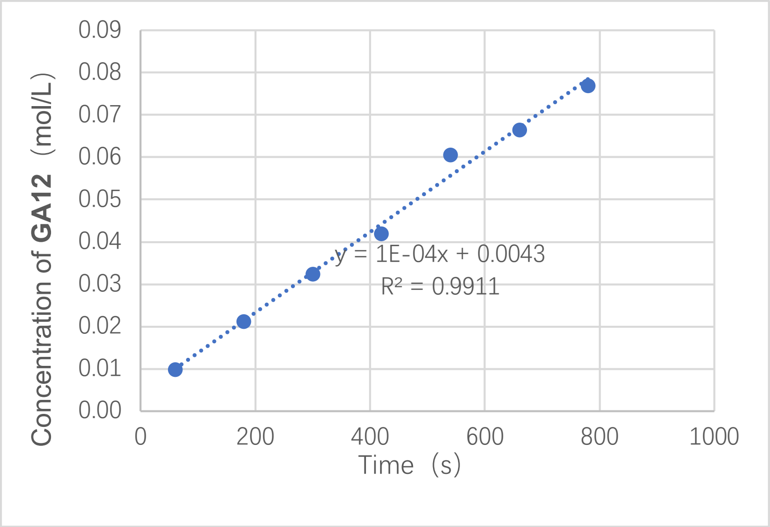

Fig. S45

System **a**, 0.8 mmol of **G1**

Time (min)	Concentration of **GA12** (mol/L)	Yield of **GA12** (%)
1	1.42E-02	14.2
3	3.64E-02	36.5
5	5.14E-02	51.5
7	8.31E-02	83.1

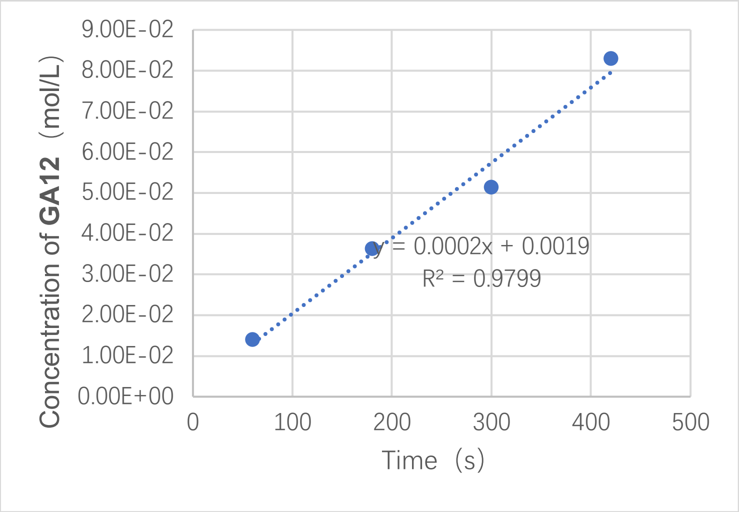

Fig. S46

System b, 0.2 mmol of A2

Time (min)	Concentration of **GA12** (mol/L)	Yield of **GA12** (%)
10	3.01E-02	30.06
15	3.70E-02	36.97
20	4.34E-02	43.42
25	5.04E-02	50.41
30	5.85E-02	58.47

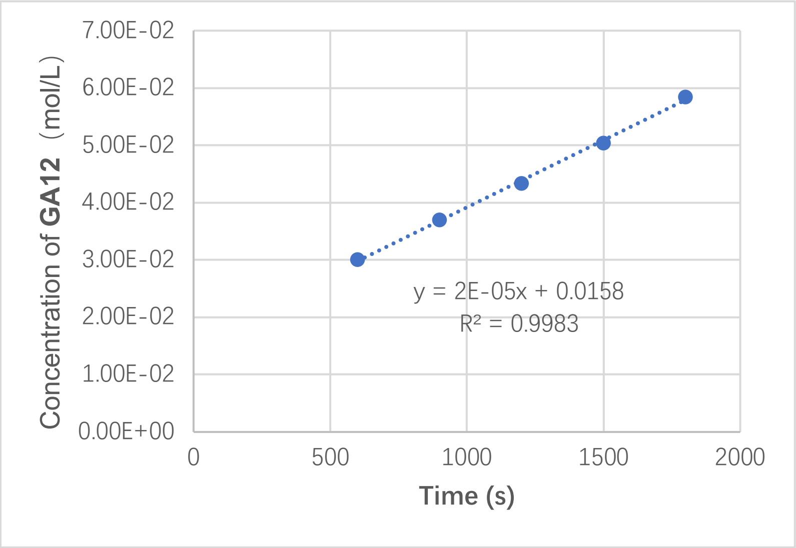

Fig. S47

System b, 0.4 mmol of A2

Time (min)	Concentration of **GA12** (mol/L)	Yield of **GA12** (%)
2	7.40E-03	7.4
4	1.48E-02	14.8
6	2.09E-02	20.9
8	2.62E-02	26.2
10	3.25E-02	32.5
12	4.11E-02	41.1

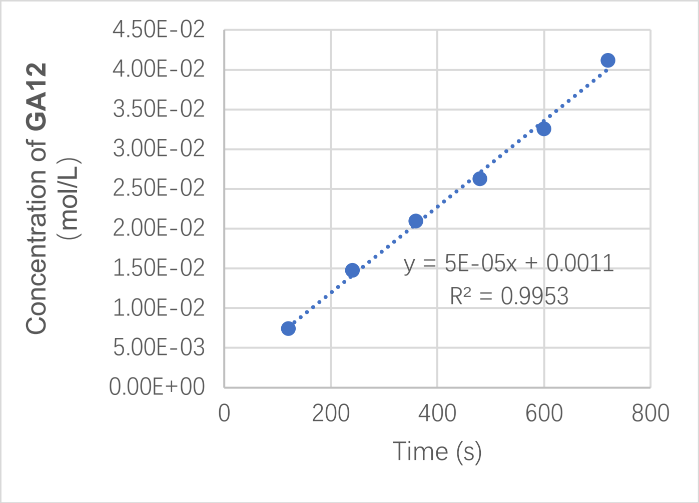

Fig. S48

System b, 0.6 mmol of A2

Time (min)	Concentration of **GA12** (mol/L)	Yield of **GA12** (%)
2	5.41E-03	5.4
4	1.31E-02	13.1
6	1.85E-02	18.5
8	2.50E-02	25.0
10	3.16E-02	31.6
12	3.80E-02	38.0

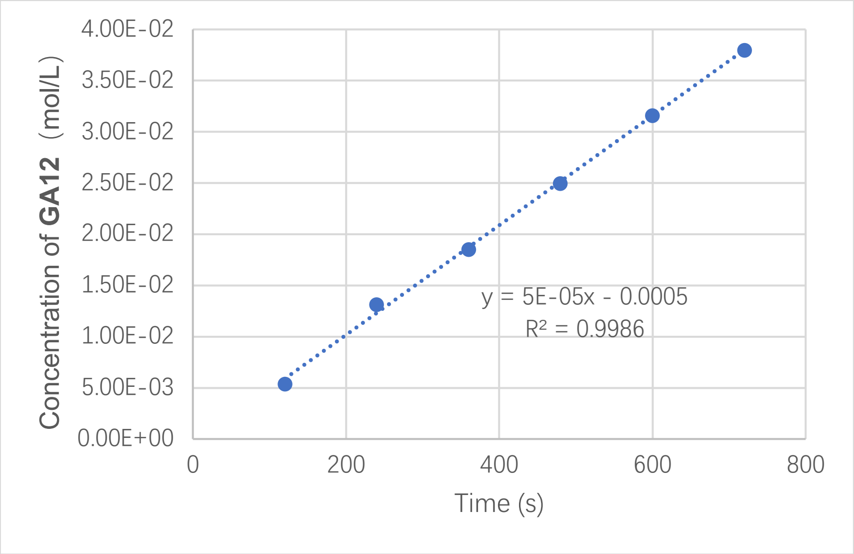

Fig. S49

System b, 0.8 mmol of A2

Time (min)	Concentration of **GA12** (mol/L)	Yield of **GA12** (%)
2	8.78E-03	8.8
4	1.76E-02	17.6
6	2.65E-02	26.5
8	3.54E-02	35.4
10	4.42E-02	44.2
12	5.29E-02	52.9
14	6.06E-02	60.6

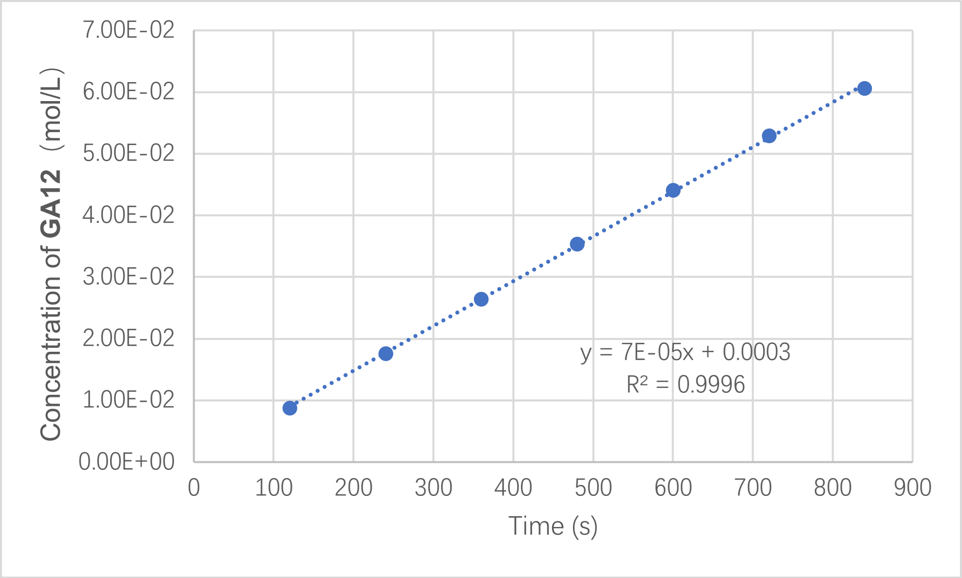

Fig. S50

Variable G1 Equivalents Summary Table

Equiv. of **G1**	Concentration of **G1** (mol/L)	Initial rate (mol*L^{-1}*s^{-1})
0.2	0.05	6.00E-06
0.4	0.1	5.00E-05
0.6	0.15	1.00E-04
0.8	0.2	2.00E-04

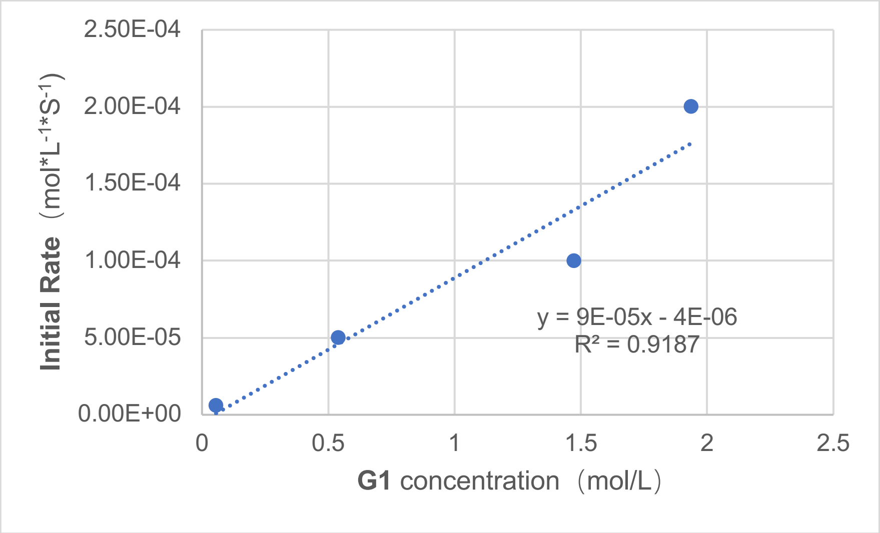

Fig. S51

Variable A2 Equivalents Summary Table

Equiv. of **A2**	Concentration of **A2** (mol/L)	Initial rate (mol*L^{-1}*s^{-1})
0.2	0.05	2.00E-05
0.4	0.1	5.00E-05
0.6	0.15	5.00E-05
0.8	0.2	7.00E-05

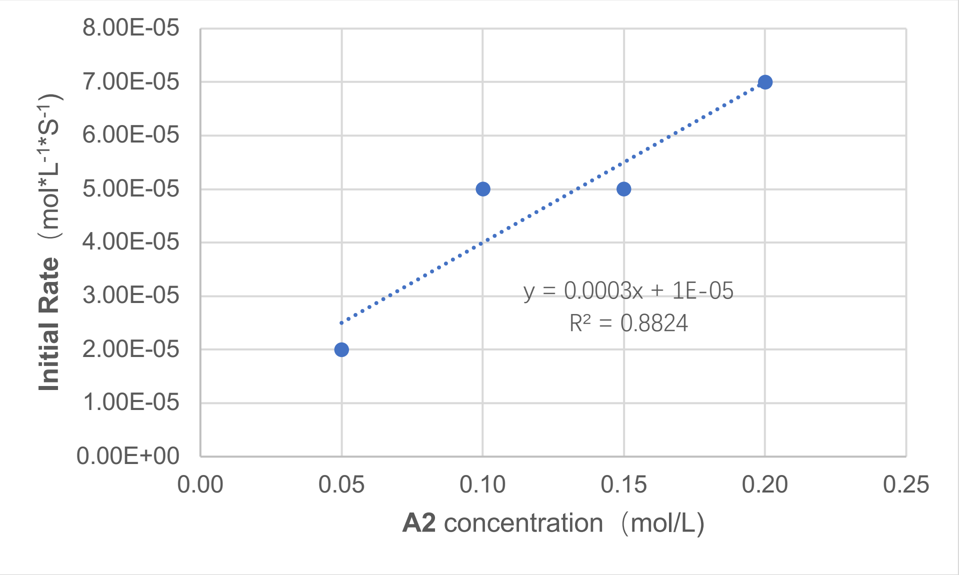

Fig. S52

14 The Scale-up Reaction

14.1 10 mmol scale synthesis of GA12

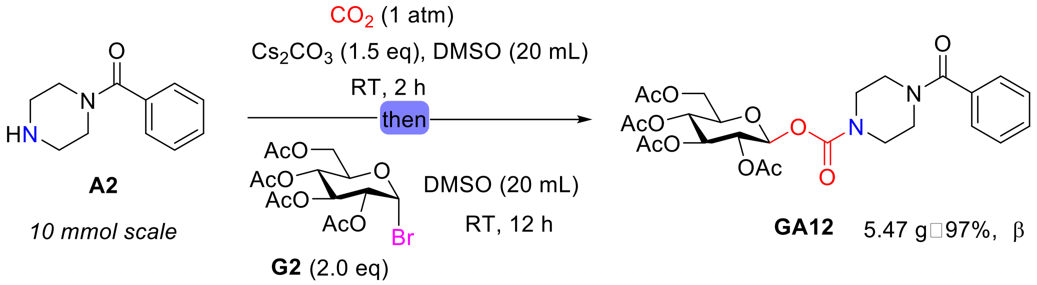

Fig. S53

An oven-dried Schlenk flask (250 ml) were charged with **A2** (1.90 g, 10 mmol), Cs_2CO_3 (4.9 g, 15 mmol) and a Teflon-coated magnetic stirring bar. After the mixture was evacuated and backfilled with CO_2 gas three times, DMSO (20 mL) was added via a syringe. The reaction mixture was allowed to stir for 2 h under a CO_2 atmosphere at RT, and then a solution of **G2** (8.22 g, 20 mmol) in DMSO (20 mL) was added, and the reaction was further stirred at RT for 12 h. H_2O (100 mL) was added, and the mixture was extracted with CH_2Cl_2 (200 mL × 3), washed with saturated NH_4Cl (500 mL), then dried over anhydrous Na_2SO_4 and filtered. The solvent was removed in vacuo and the residue was purified by silica gel flash chromatography (petroleum ether/EtOAc = 2:1 to 3:2 to 1:3) to give **GA12** as a white solid (5.47 g, 97%).

14.2 10 mmol scale synthesis of GA40

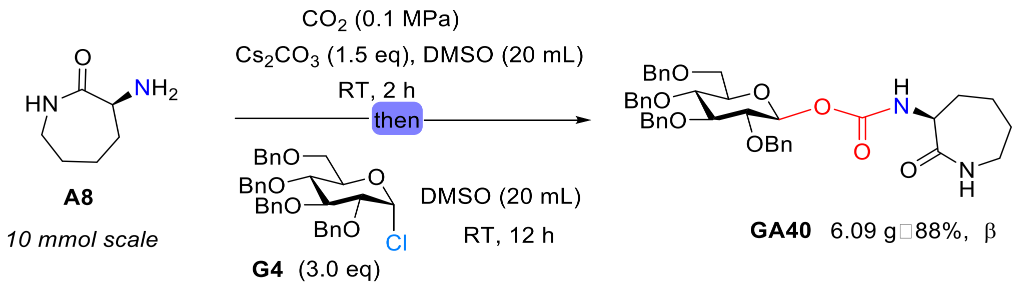

Fig. S54

An oven-dried Schlenk tube (250 ml) were charged with **A8** (1.28 g, 10 mmol), Cs_2CO_3 (4.9 g, 15 mmol) and a Teflon-coated magnetic stirring bar. After the mixture was evacuated and backfilled with CO_2 gas three times, DMSO (20 mL) was added

via a syringe. The reaction mixture was allowed to stir for 2 h under a CO_2 atmosphere at RT, and then a solution of **G4** (16.8 g, 30 mmol) in DMSO (20 mL) was added, and the reaction was further stirred at RT for 12 h. H_2O (100 mL) was added, and the mixture was extracted with CH_2Cl_2 (200 mL × 3), washed with saturated NH_4Cl (500 mL), then dried over anhydrous Na_2SO_4 and filtered. The solvent was removed in vacuo and the residue was purified by silica gel flash chromatography (petroleum ether/EtOAc = 2:1 to 1:1) to give **GA40** as a white solid (6.09 g, 88%).

15 Deprotection

15.1 Deprotection of the acetyl (Ac) groups.

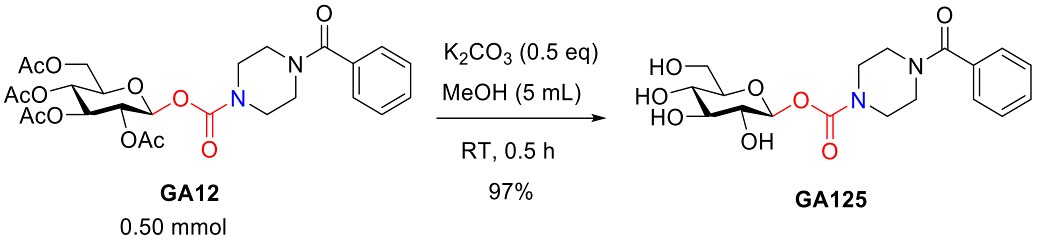

Fig. S55

To a solution of compound **GA12** (282 mg, 0.50 mmol) in MeOH (5.0 mL) was added K_2CO_3 (35 mg, 0.25 mmol). The reaction mixture was stirred at room temperature for 30 mins. Dowex H^+ resin was added to the reaction mixture until neutral pH. The suspension was filtered off, and washed with MeOH. The filtrate was concentrated to give compound **GA125** (193 mg, 97%) as a yellow syrup.

$[\alpha]_D^{25}$ = 5.0 (*c* 1.0, MeOH)

^{1}H NMR (500 MHz, CD_3OD) δ 7.59–7.24 (m, 5H), 5.40 (d, *J* = 8.0 Hz, 1H), 4.01–3.17 (m, 15H).

^{13}C NMR (126 MHz, CD_3OD) δ 172.46, 155.20, 135.91, 131.17, 129.59, 127.83, 97.14, 78.25, 77.38, 73.51, 70.71, 62.10.

HRMS (ESI) m/z calcd for $C_{18}H_{24}N_2O_8Na$ $[M+Na]^+$ 419.1425; **found**: 419.1425.

15.2 Deprotection of the benzyl (Bn) groups.

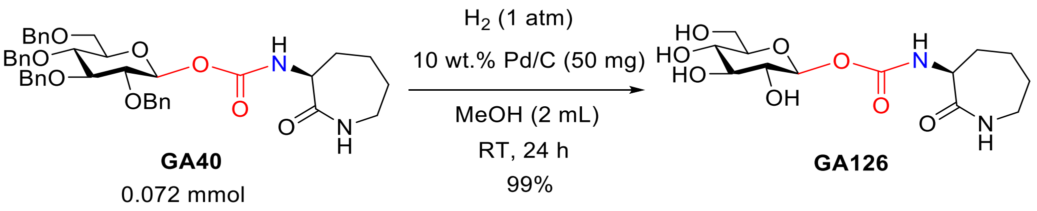

Fig. S56

A mixture of compound **GA40** (50 mg, 0.072 mmol) and 10% Pd on carbon (50 mg) in MeOH (2 mL) was stirred at room temperature under hydrogen atmosphere (1 atm). After 24 h, the catalyst was separated by filtration, and the solvent was removed under reduced pressure to give compound **GA126** (24 mg, 99%) as a white solid.

$[\alpha]_D^{25}$ = -27.9 (*c* 0.25, MeOH)

^{1}H NMR (500 MHz, CD_3OD) δ 5.35 (d, *J* = 8.1 Hz, 1H), 4.39 (dd, *J* = 11.5, 1.8 Hz, 1H), 3.87 (dd, *J* = 12.0, 2.1 Hz, 1H), 3.69 (dd, *J* = 12.0, 5.2 Hz, 1H), 3.48–3.21 (m, 9H), 2.10–1.94 (m, 2H), 1.90–1.73 (m, 2H), 1.45–1.31 (m, 2H).

^{13}C NMR (126 MHz, CD_3OD) δ 177.20, 156.28, 96.94, 78.57, 77.75, 73.96, 71.08, 62.38, 54.84, 42.45, 32.40, 29.84, 29.12.

HRMS (ESI) m/z calcd for $C_{13}H_{22}N_2O_8Na$ $[M+Na]^+$ 357.1268; **found**: 357.1274.

15.3 Deprotection of the *t*-butyloxy carbonyl (Boc) group

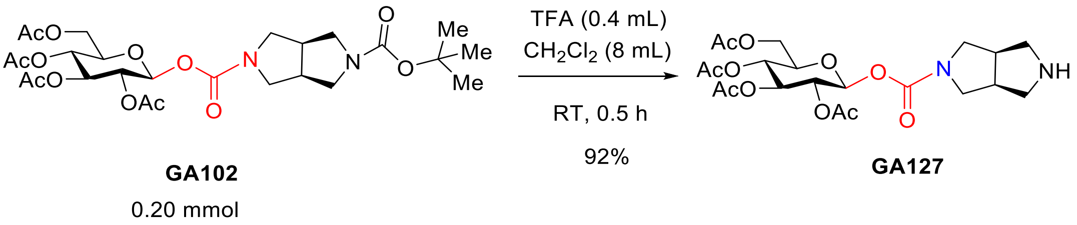

Fig. S57

To a solution of compound **GA102** (117 mg, 0.20 mmol) in CH_2Cl_2 (8.0 mL) at 0 ºC was added TFA (0.40 mL). The reaction mixture was stirred at room temperature for 30 mins. The reaction mixture was filtered off, then the filtrate was concentrated, MeOH (4.0 mL) and K_2CO_3 (27.6 mg, 0.20 mmol) were added. After being stirred at room temperature for 30 mins, the mixture was filtered off, and washed with MeOH. The filtrate was concentrated to give compound **GA127** (89 mg, 92%) as a yellow syrup.

$[\alpha]_D^{25}$ = -9.3 (*c* 1.0, $CHCl_3$)

^{1}H NMR (600 MHz, CD_3OD) δ 5.68 (dd, *J* = 8.5, 4.1 Hz, 1H), 5.34 (t, *J* = 9.5 Hz, 1H), 5.03 (td, *J* = 9.3, 3.0 Hz, 2H), 4.24 (dd, *J* = 12.5, 4.4 Hz, 1H), 4.07 (dd, *J* = 12.5, 2.3 Hz, 1H), 3.99 (ddd, *J* = 10.2, 4.5, 2.3 Hz, 1H), 3.72–3.59 (m, 2H), 3.59–3.49 (m, 3H), 3.46–3.36 (m, 2H), 3.19–3.03 (m, 4H), 2.01 (s, 3H), 1.98 (s, 3H), 1.95 (s, 3H).

^{13}C NMR (151 MHz, CD_3OD) δ 172.26, 171.46, 171.23, 153.63, 153.56, 94.36, 73.74, 73.40, 71.78, 71.66, 71.62, 69.39, 62.82, 50.77, 43.18, 42.27, 20.61, 20.56, 20.51.

HRMS (ESI) m/z calcd for $C_{21}H_{30}N_2O_{11}Na$ $[M+Na]^+$ 509.1742; **found**: 509.1749.

16. Preparation of crizotinib glucoside GA95

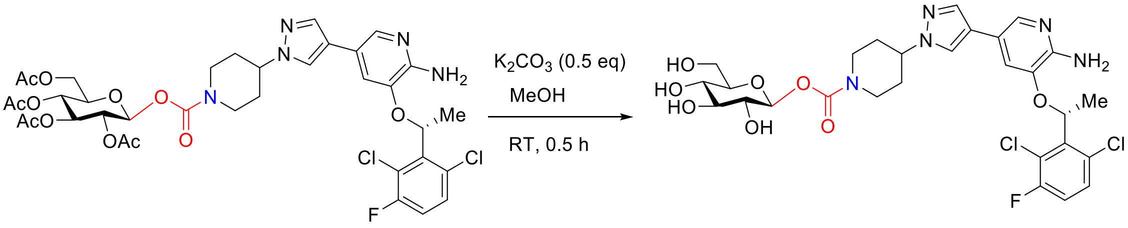

GA89 GA95

Fig. S58

To a solution of compound **GA89** (20.0 mg, 0.024 mmol) in MeOH (1.0 mL) was added K_2CO_3 (1.7 mg, 0.012 mmol). The reaction mixture was stirred at room temperature for 30 mins. Dowex H^+ resin was added to the reaction mixture until neutral pH. The suspension was filtered off, and washed with MeOH. The filtrate was concentrated to give compound **GA95** (12.9 mg, 81%) as a yellow solid.

$[\alpha]_D^{25}$ = -13.8 (*c* 0.1, MeOH)

^{1}H NMR (600 MHz, DMSO-d_6) δ 7.98 (s, 1H), 7.75 (d, J = 1.8 Hz, 1H), 7.57 (dd, J = 9.0, 4.9 Hz, 1H), 7.54 (d, J = 0.8 Hz, 1H), 7.44 (t, J = 8.7 Hz, 1H), 6.91 (d, J = 1.9 Hz, 1H), 6.08 (q, J = 6.7 Hz, 1H), 5.65 (s, 2H), 5.29–5.21 (m, 2H), 5.08 (d, J = 4.9 Hz, 1H), 4.98 (d, J = 5.4 Hz, 1H), 4.57 (t, J = 5.9 Hz, 1H), 4.40–4.32 (m, 1H), 4.16–4.04 (m, 2H), 3.65 (s, 1H), 3.51–3.41 (m, 1H), 3.33 (s, 5H), 3.22 (td, J = 8.9, 4.3 Hz, 1H), 3.20–3.09 (m, 4H), 3.08–2.93 (m, 2H), 2.08–1.97 (m, 2H), 1.88–1.81 (m, 2H), 1.80 (d, J = 6.6 Hz, 3H).

^{13}C NMR (151 MHz, DMSO) δ 157.65, 156.01, 153.12, 149.50, 138.79, 136.84, 135.54, 134.70, 130.57, 128.76, 128.74, 123.69, 121.11, 120.98, 119.25, 117.54, 117.38, 117.31, 114.47, 95.78, 77.62, 76.45, 72.47, 71.99, 69.50, 60.53, 57.86, 48.60, 42.37, 42.33, 40.43, 32.06, 31.62, 18.60.

HRMS (ESI) m/z calcd for $C_{28}H_{33}N_5O_8Cl_2FNa$ $[M+Na]^+$ 678.1504; **found**: 678.1510.

17. Bioactivity study on crizotinib glucoside

17.1 Cell viability assay

Cancer cell lines were procured from the National Collection of Authenticated Cell Cultures, China. All cells were kindly provided by the Cell Bank of the Chinese Academy of Sciences and have been certified through STR analysis. All cells were tested using HiScript III All-in-one RT SuperMix Perfect for qPCR (Vazyme, Cat: R333-01), and the test results were negative for mycoplasma contamination. These cells were cultured in Dulbecco's Modified Eagle's Medium (Gibco), supplemented with 100 U/mL penicillin and 100 U/mL streptomycin (Gibco). The cells were hemi-depleted each week with fresh medium and maintained at 3 × 105 cells/mL at 37 oC and 5% CO2. Cell viability was analyzed by Cell Counting Kit-8 (CCK8, Beyotime, Shanghai, China) according to the manufacturer's protocols. Cells were seeded and cultured at a density of 3 × 103 /well in 100 µL of medium into 96-well microplates (Corning, USA). Then, the cells were treated with various concentrations of compounds (0, 5 nM, 30 µM, 50 nM, 300 nM, 500 nM, 3 µM, 5 µM, 30 µM, 50 µM or 0, 0.13 nM, 0.53 nM, 1.64 nM, 4.92 nM, 14.80 nM, 44.40 nM, 133 nM, 400 nM, 1200 nM). After treatment for 72 hours, 10 µL of CCK-8 reagent was added to each well and then cultured for 4 hours. All experiments were performed in pentaplicate. The absorbance was measured at 450 nm by a microplate reader (PerkinElmer, USA) using wells without cells as blanks. The proliferation of cells was expressed by the absorbance.

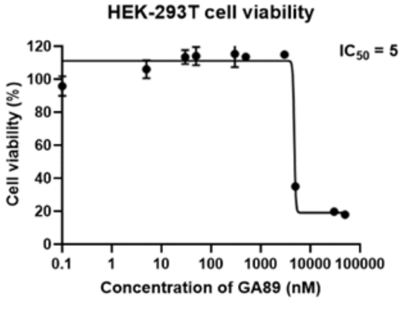

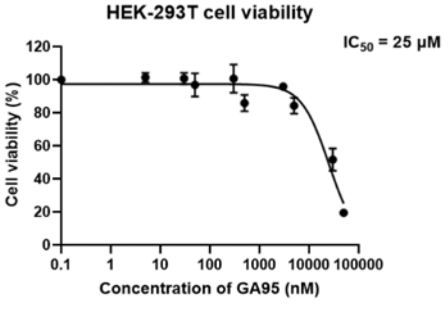

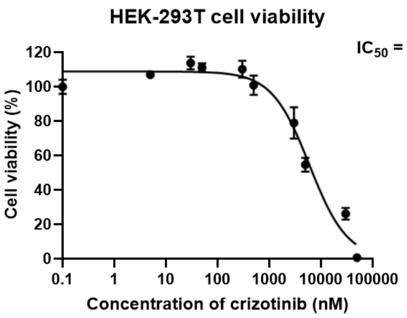

Fig. S59

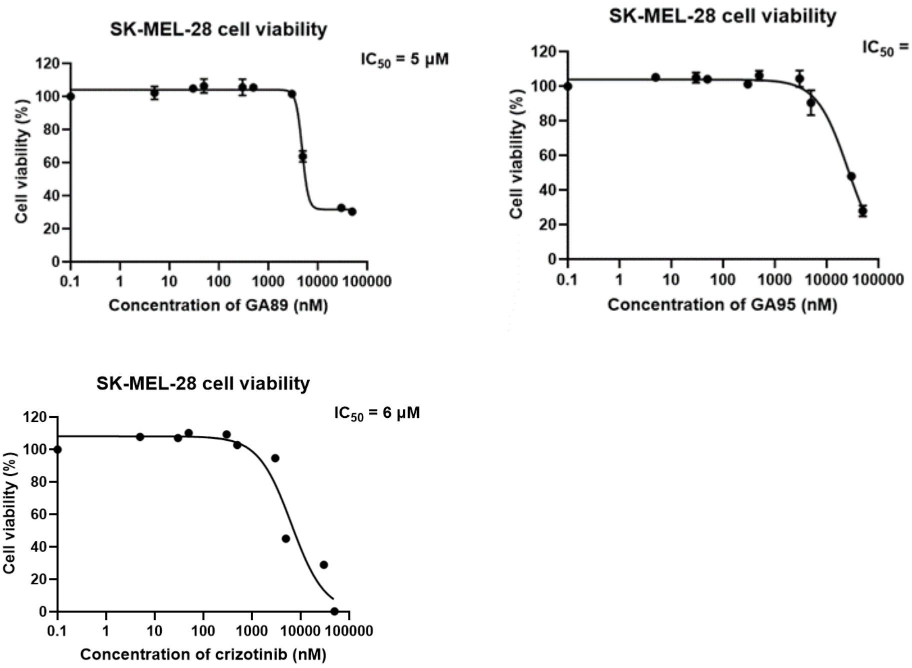

Fig. S60

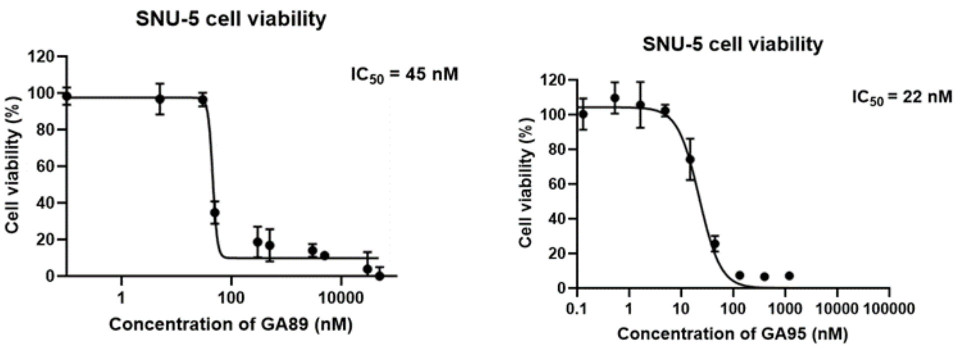

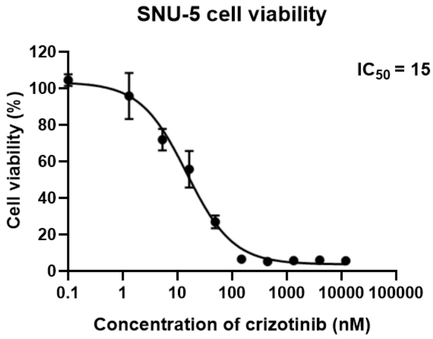

Fig. S61

17.2 Enzymatic inhibition assay

17.2.1 Protein purification[29]

The plasmid pCDNA3.1_8×His-tev-ALK (kinase domain, 1068–1410) was constructed and extracted from E. coli DH5α (DE3) strain by alkaline solution method. The plasmid was transiently transfected into 3 L Expi293 cells at a density of 3×106 cells/mL using transfection reagent PEI (25,000 Da). The cells were collected by centrifugation on the 5th day after transfection, and lysed at 600 bar at 4 °C. The centrifugation supernatant of cell lysate was loaded to a pre-balanced Ni-NTA column and washed with 100 mL solution containing 25 mM Tris pH 8.0, 150 mM NaCl, and 25 mM imidazole. The ALK protein was then eluted with 40 mL solution containing 25 mM Tris pH 8.0, 150 mM NaCl, and 250 mM imidazole. The eluent was concentrated to 500 μL and further purified by a Superdex 200 column with the running buffer containing 25 mM HEPES pH 7.4 and 250 mM NaCl. The peak fractions were used for enzymatic assays.

17.2.2 ATP hydrolysis Assays

The ATP hydrolysis assays were based on Kinase-Glo luminescent Kit (Promega). The experiment was carried out in Corning 384-well white flat bottom microplates. The buffer in the reaction contains 25 mM HEPES pH 7.4, 140 mM NaCl, 40 mM MgCl2, 30 mM KCl, 1 mM DTT, 0.1 mg·mL-1 BSA, and 0.004% Tween-20. The compounds were diluted to different concentrations with a 5-fold gradient.

2.5 μL 100 nM ALK in HEPES buffer was mixed with crizotinib, GA89, or GA95, and incubated for 10 min. Then 5 μL substrate (2 μM ATP) was added to start the

reaction at 37 °C and incubated for 6 h. DMSO was added into negative control wells. When the reaction reaches the planned time, 10 μL diluted Kinase-Glo reagent (1/50 diluted with a buffer containing 50 mM Tris pH 7.5 and 5% glycerol) was added and incubated for 15 min. Luminescence was measured on the Magellan plate reader (Tecan) and nonlinear regression was performed with Prism (GraphPad).

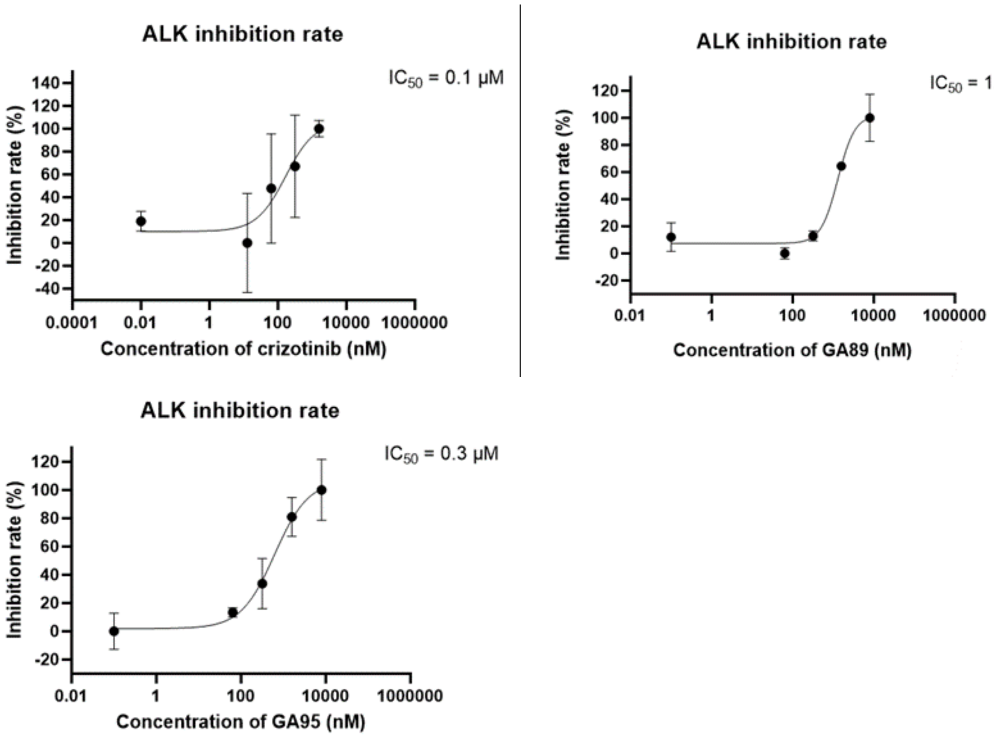

Fig. S62

18. Pharmacokinetic analysis of crizotinib glucosides.

All procedures in the animal studies were performed in accordance with the Guide for the Care and Use of Laboratory Animals of Shanghai Institute of Materia Medica, Chinese Academy of Sciences.

The animals were maintained in cages at 22 ± 3 °C and 55% relative humidity under a 12 h dark/light cycle. Rats were allowed free access to water but were fasted for 12 h before drug administration. The following protocol is typical for to evaluating the pharmacokinetic characteristics of the test molecules in male C57 mices. The animals were deprived from food over a time period of 12 h prior to administration and 4 h after administration of the test molecules. Water was supplied without limitation. On the study day, the animals received test molecule (G89, GA95 or crizotinib) by oral gavage, formulated in mixtures of 0.5%CMC-Na. Then blood was drawn from the retro-orbital venous following time points: 0.25, 0.5, 1, 2, 4, 6, 8 and 24 hours after dosing. Plasma was obtained by centrifugation at 11,000g for 10 minutes and stored at -80°C until analysis.

Circulating concentrations of test compounds were determined using LC/MS/MS methods. The analytical range in mouse plasma was linear over a concentration range of 1.00-8000 ng/mL for crizotinib, 1.00-8000 ng/mL for GA89, 1.00-8000 ng/mL for GA95. Pharmacokinetic parameters were calculated from concentration versus time data using noncompartmental pharmacokinetic methods using Phoenix pharmacokinetic software.

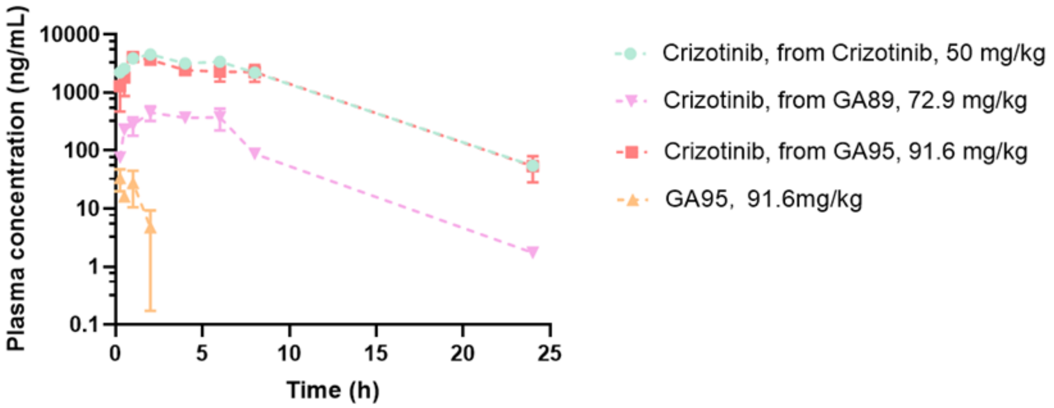

Fig. S63 Pharmacokinetic profiles after oral application of crizotinib (50 mg/kg),

GA84 (72.9 mg/kg) and GA90 (91.6 mg/kg) to healthy male C57 mice, results are the mean±SD of 3 subjects in each group.

Treatment	Analyte	T_{max} (h)	C_{max} (ng/mL)	AUC_{0-t} (μg*h/mL)	$AUC_{0-\infty}$ (μg*h/mL)	MRT (h)
**Crizotinib** (50mg/kg)	Crizotinib	2	4510	44.5	44.8	5.78
**GA95** (91.6mg/kg)	Crizotinib	1	4090	39.7	39.9	6.03
	Glycolcrizotinib	0.25	33.6	0.0376	0.0426	1.08

Table S6. Pharmacokinetic parameters after oral application of crizotinib (50 mg/kg) and GA95 (91.6 mg/kg) to healthy male C57 mice, results are the mean±SD of 3 subjects in each group.

NMR Spectra

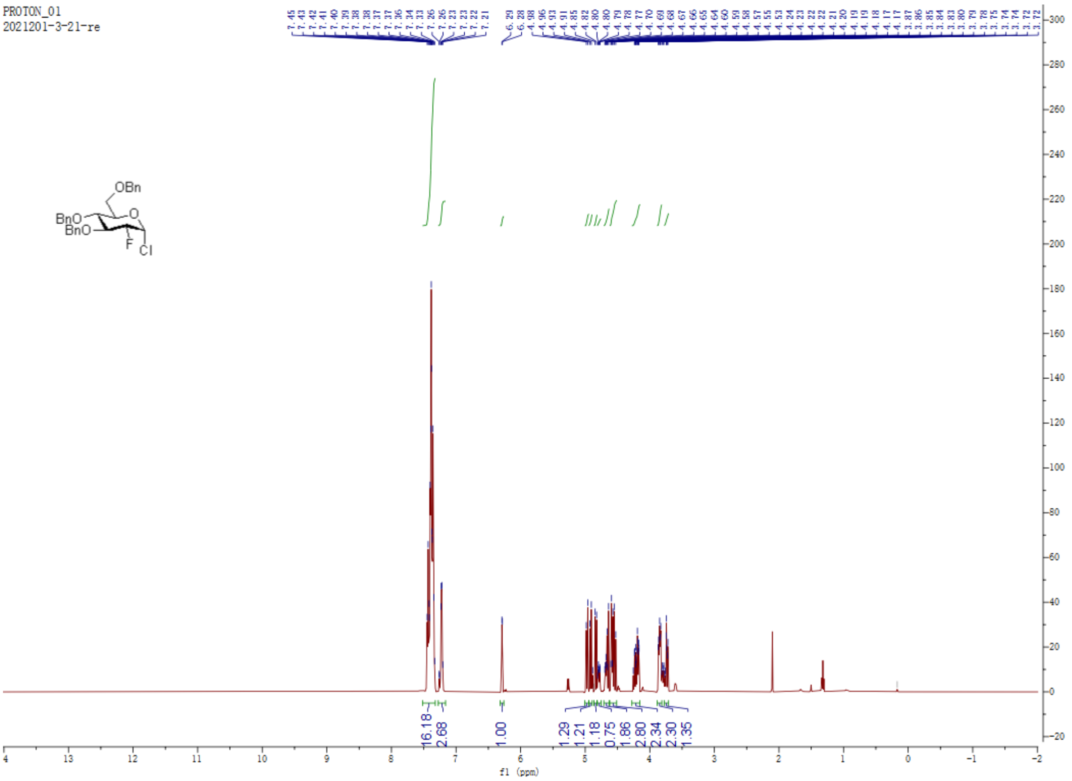

^{1}H NMR spectrum of compound **G10** (500 MHz, $CDCl_3$)

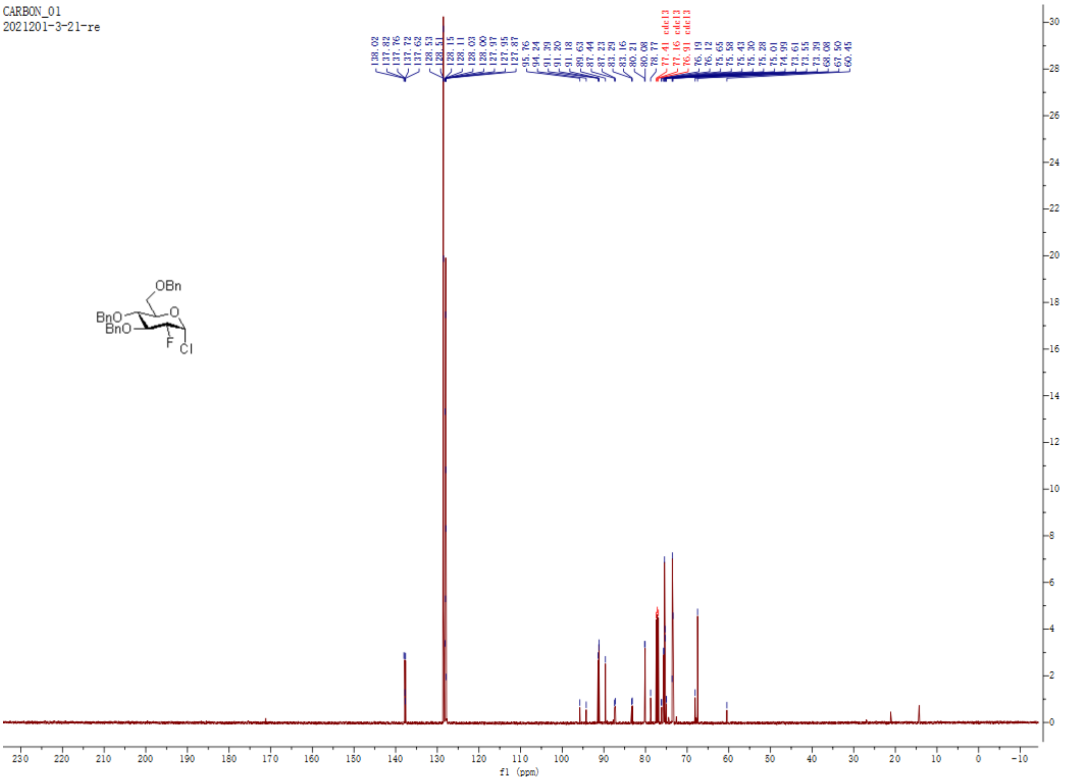

^{13}C NMR spectrum of compound **G10** (126 MHz, $CDCl_3$)

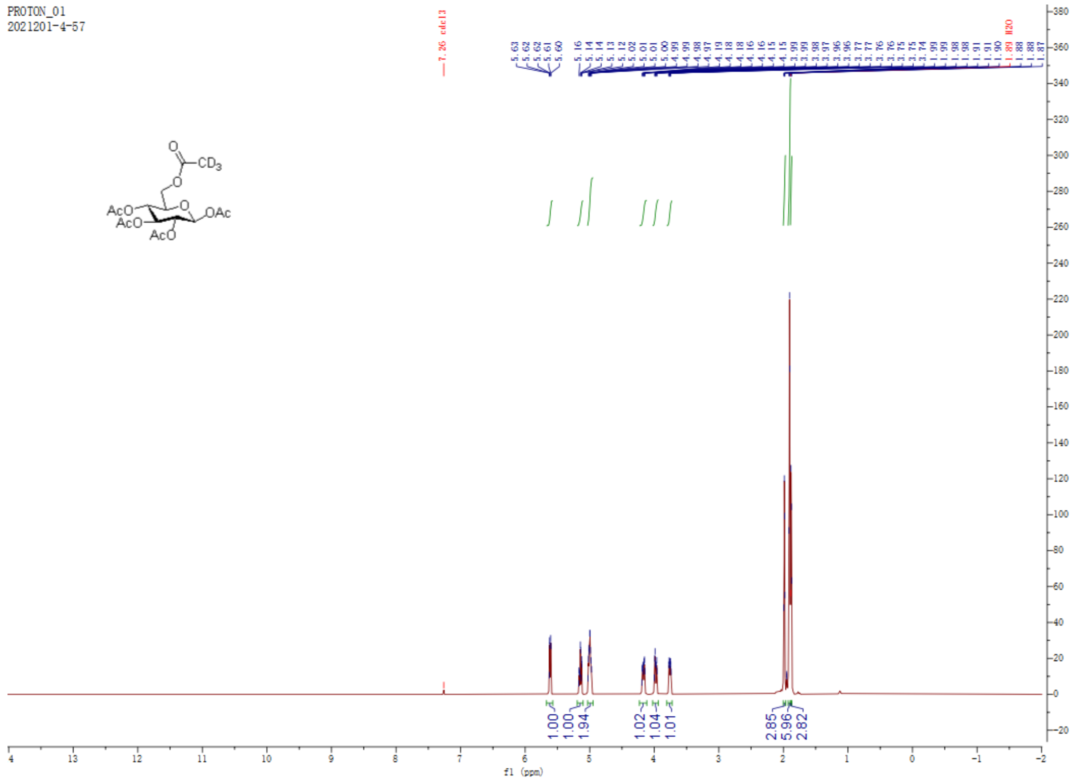

^{1}H NMR spectrum of compound **S3** (500 MHz, $CDCl_3$)

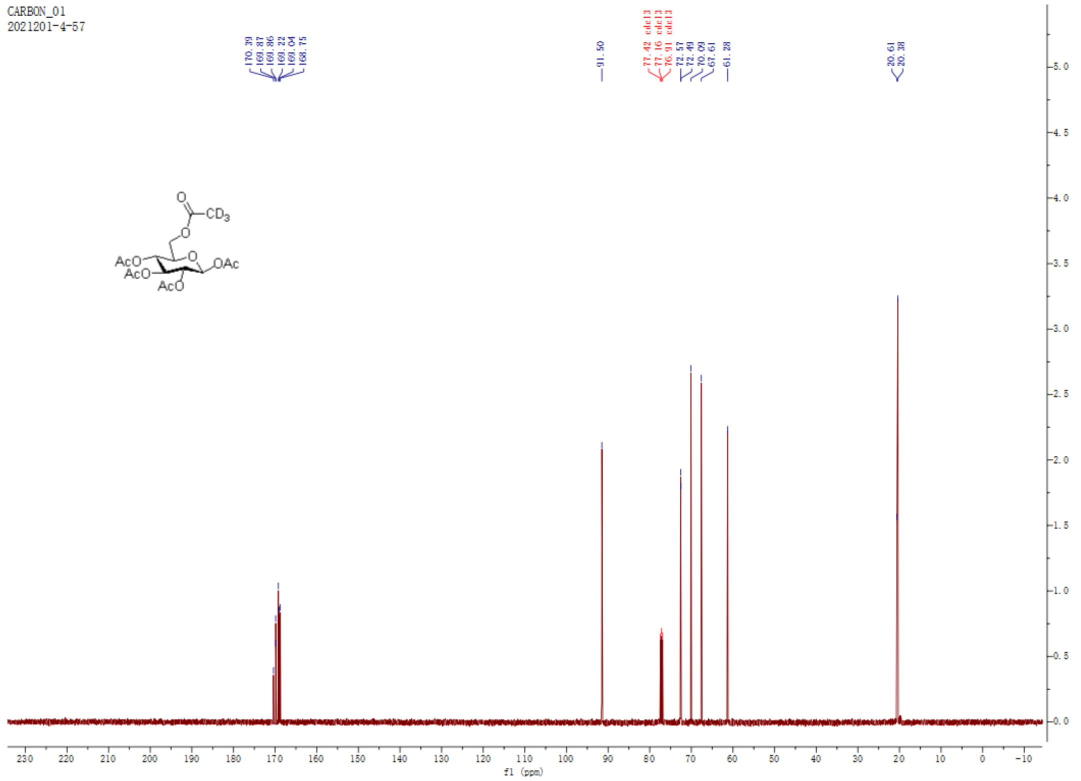

^{13}C NMR spectrum of compound **S3** (126 MHz, $CDCl_3$)

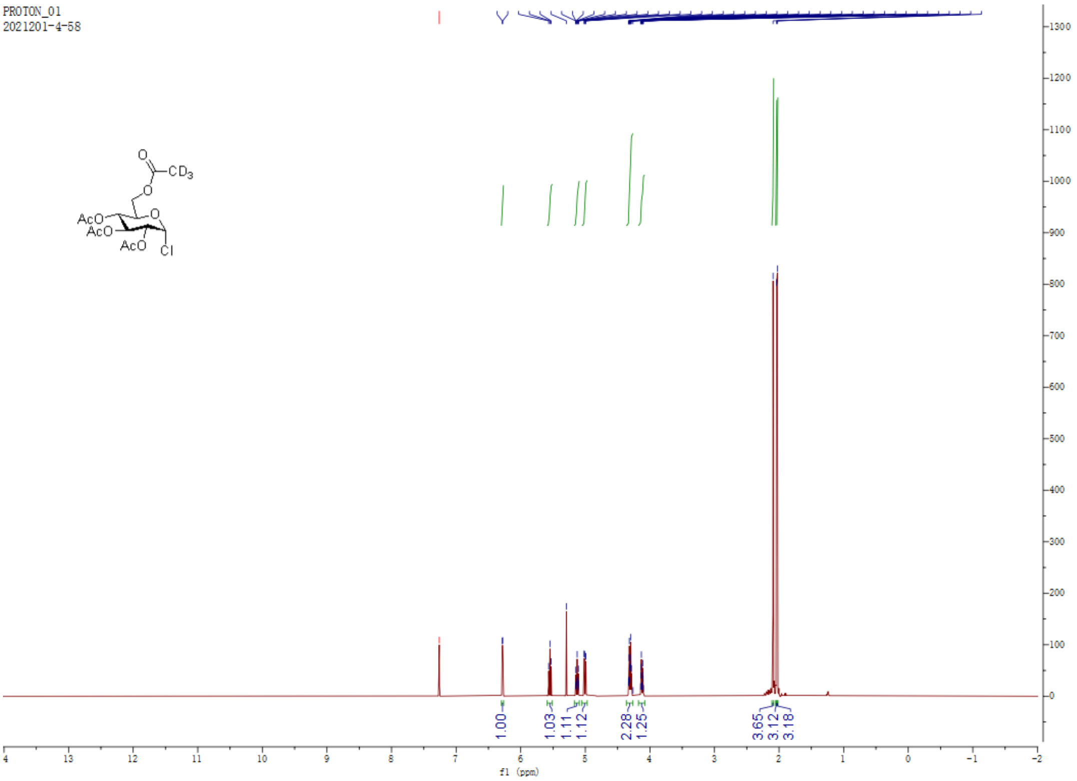

^{1}H NMR spectrum of compound **G1**-6-*d*3 (500 MHz, $CDCl_3$)

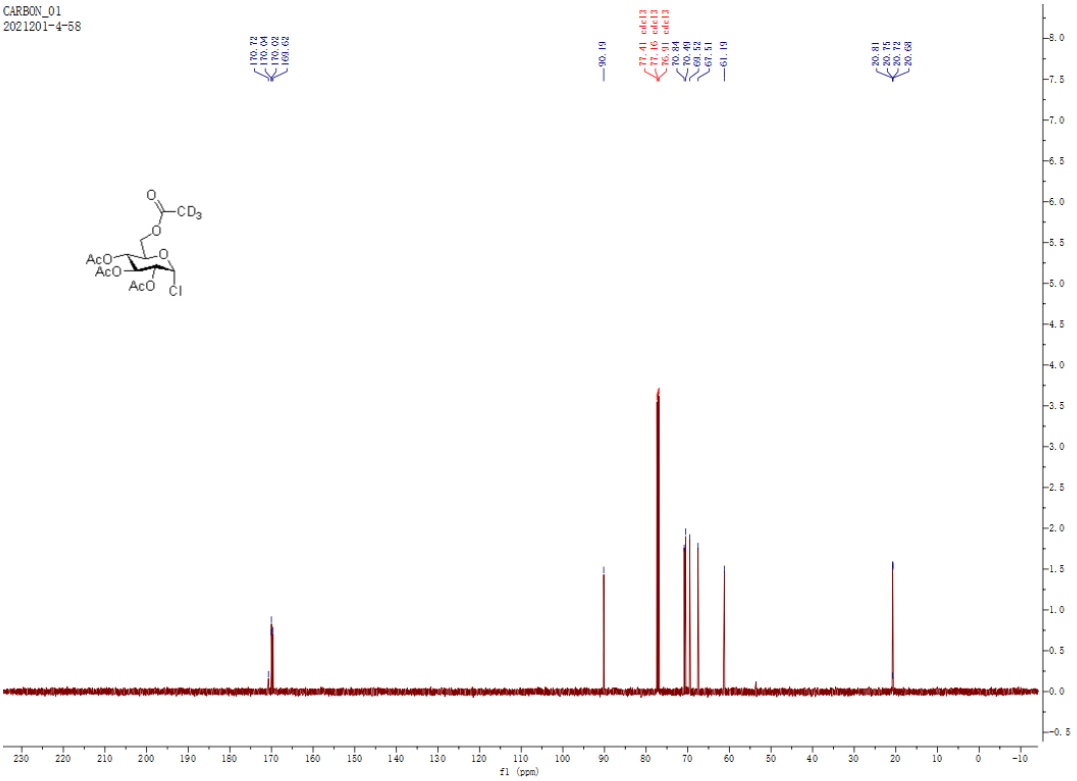

^{13}C NMR spectrum of compound **G1**-6-*d*3 (126 MHz, $CDCl_3$)

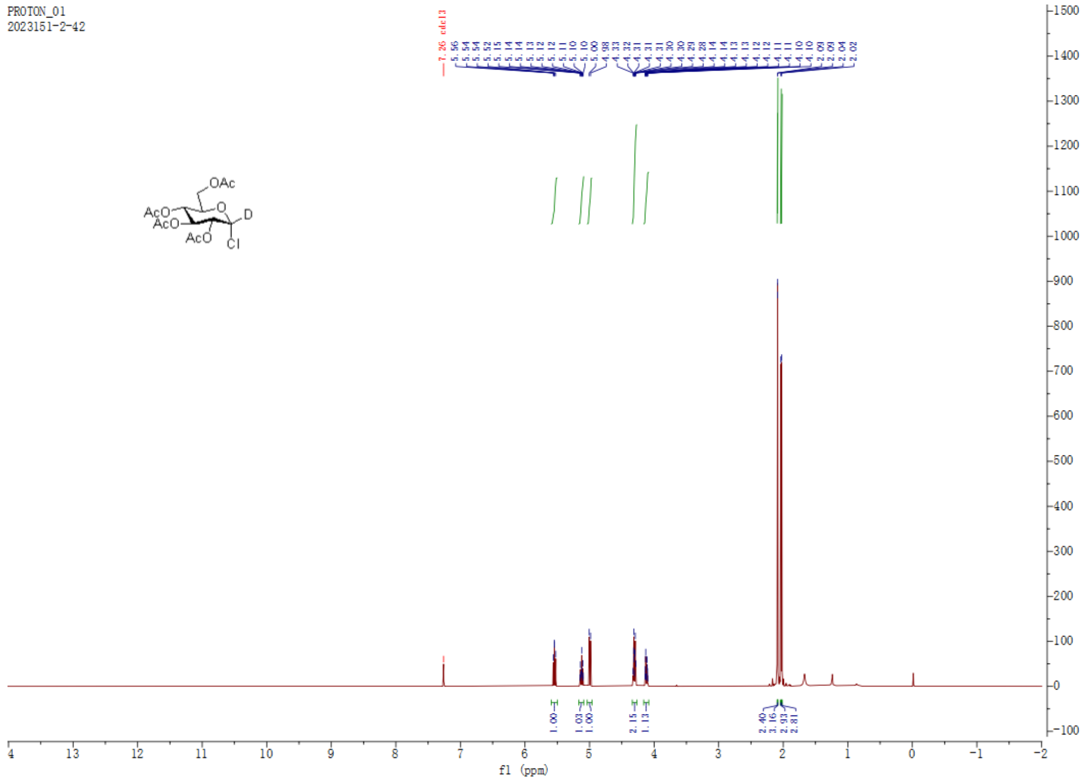

^{1}H NMR spectrum of compound **G1**-1-*d* (500 MHz, $CDCl_3$)

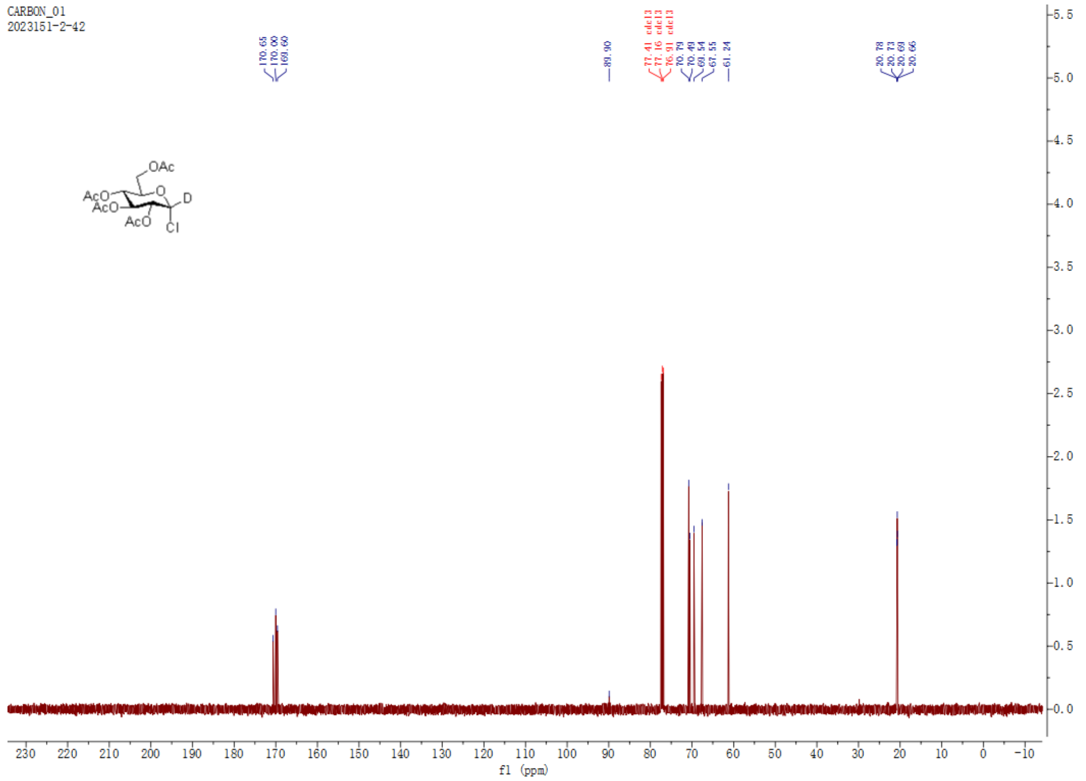

^{13}C NMR spectrum of compound **G1**-1-*d* (126 MHz, $CDCl_3$)

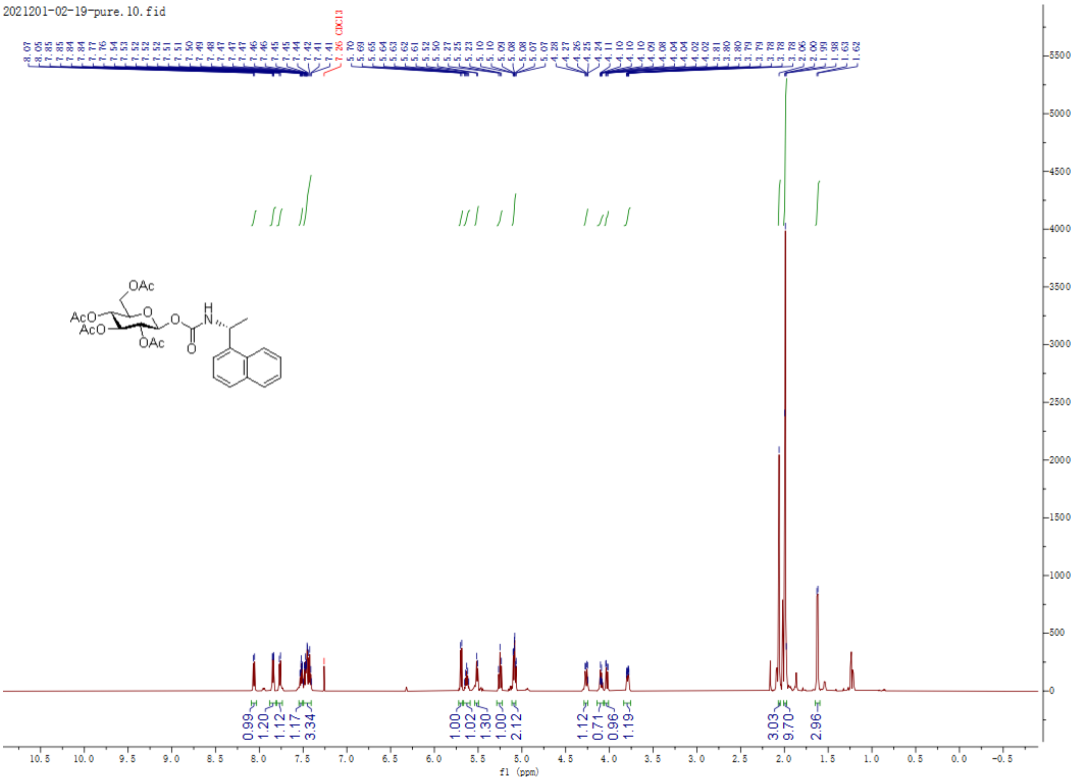

^{1}H NMR spectrum of compound **GA1** (500 MHz, $CDCl_3$)

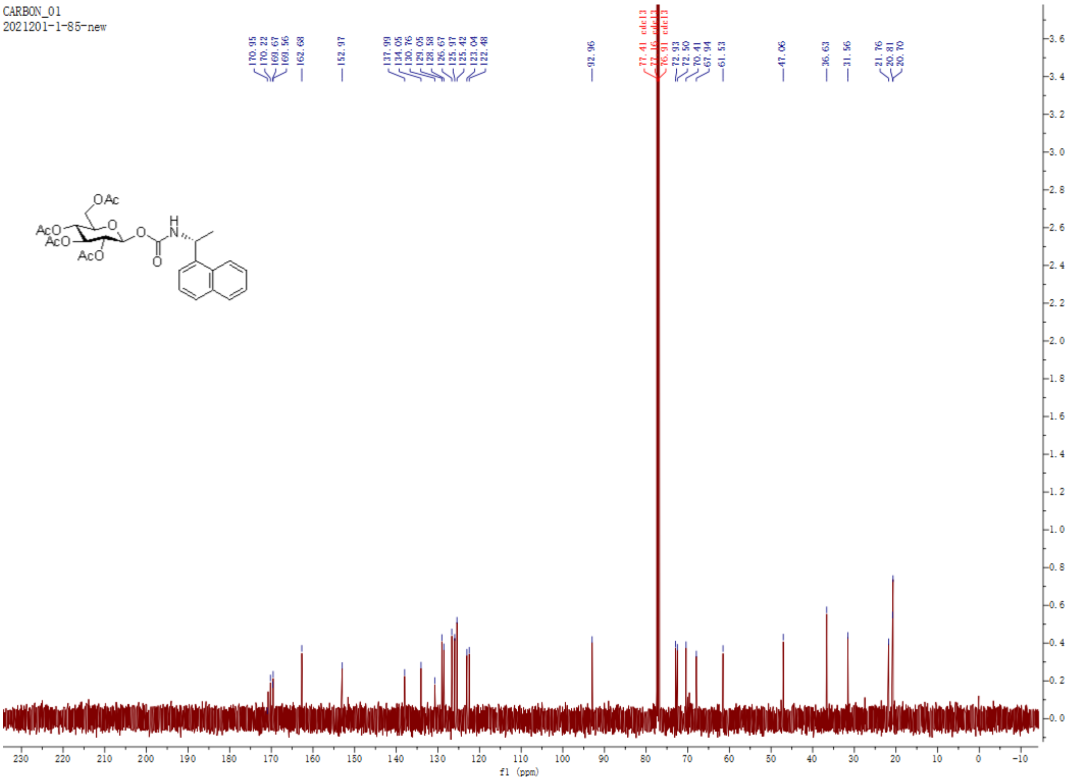

^{13}C NMR spectrum of compound **GA1** (126 MHz, $CDCl_3$)

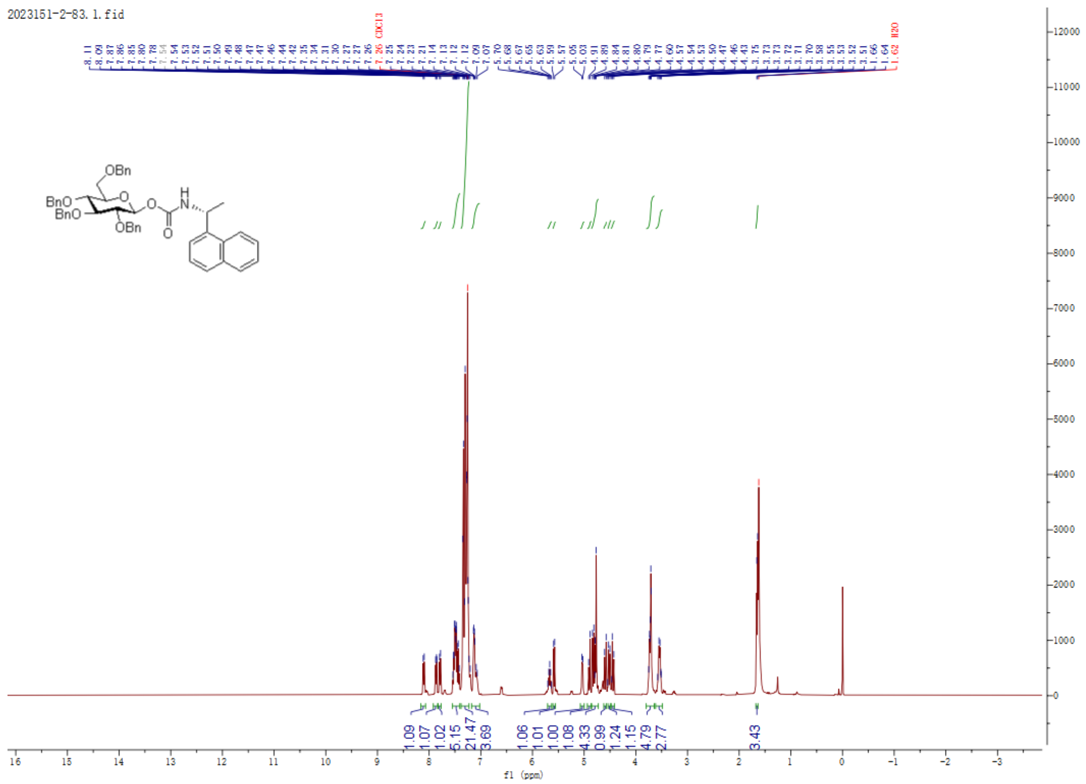

^{1}H NMR spectrum of compound **GA2** (500 MHz, $CDCl_3$)

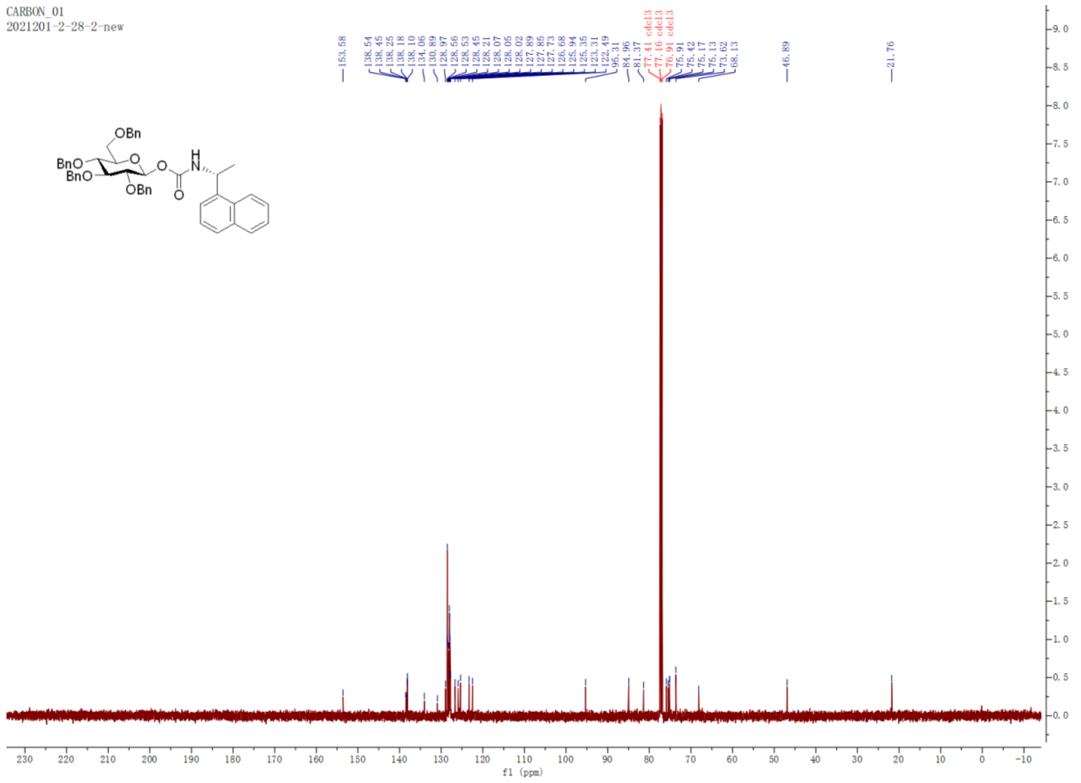

^{13}C NMR spectrum of compound **GA2** (126 MHz, $CDCl_3$)

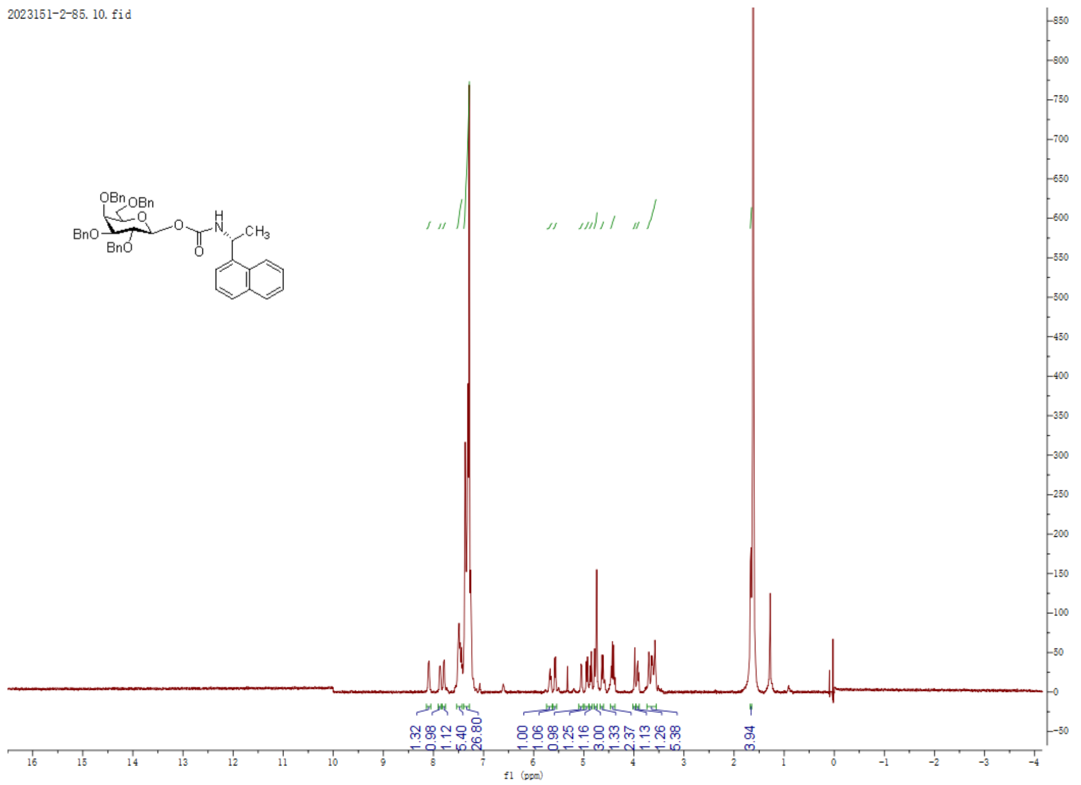

^{1}H NMR spectrum of compound **GA4** (600 MHz, $CDCl_3$)

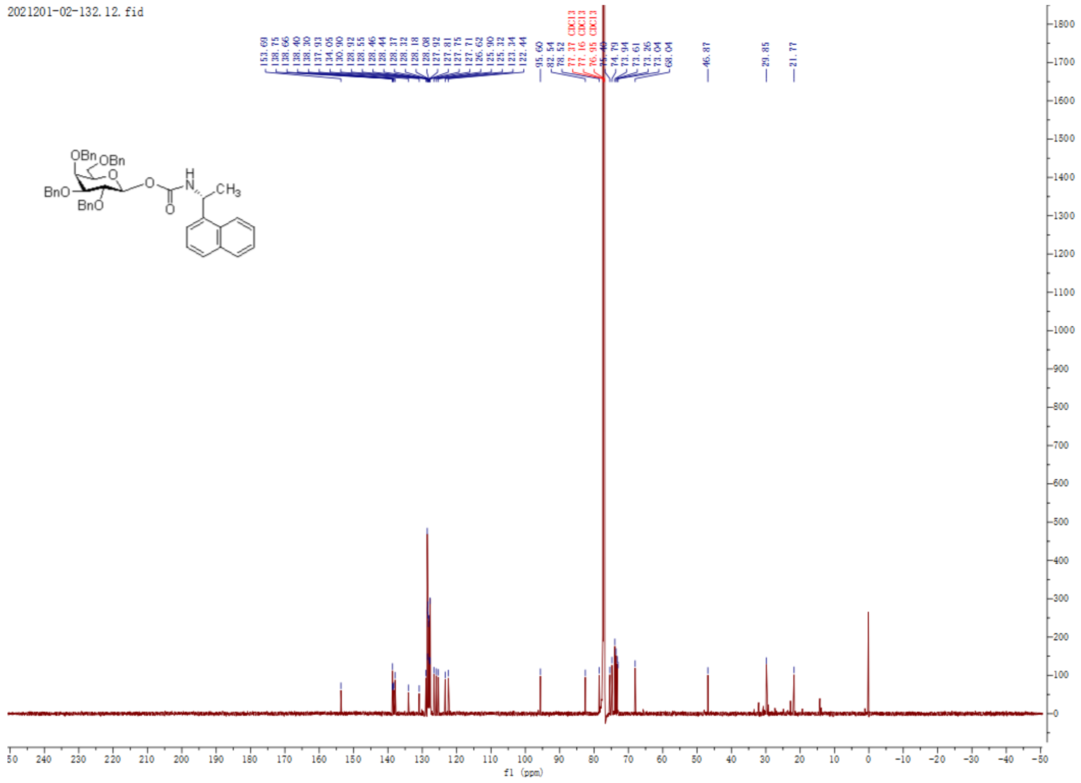

^{13}C NMR spectrum of compound **GA4** (151 MHz, $CDCl_3$)

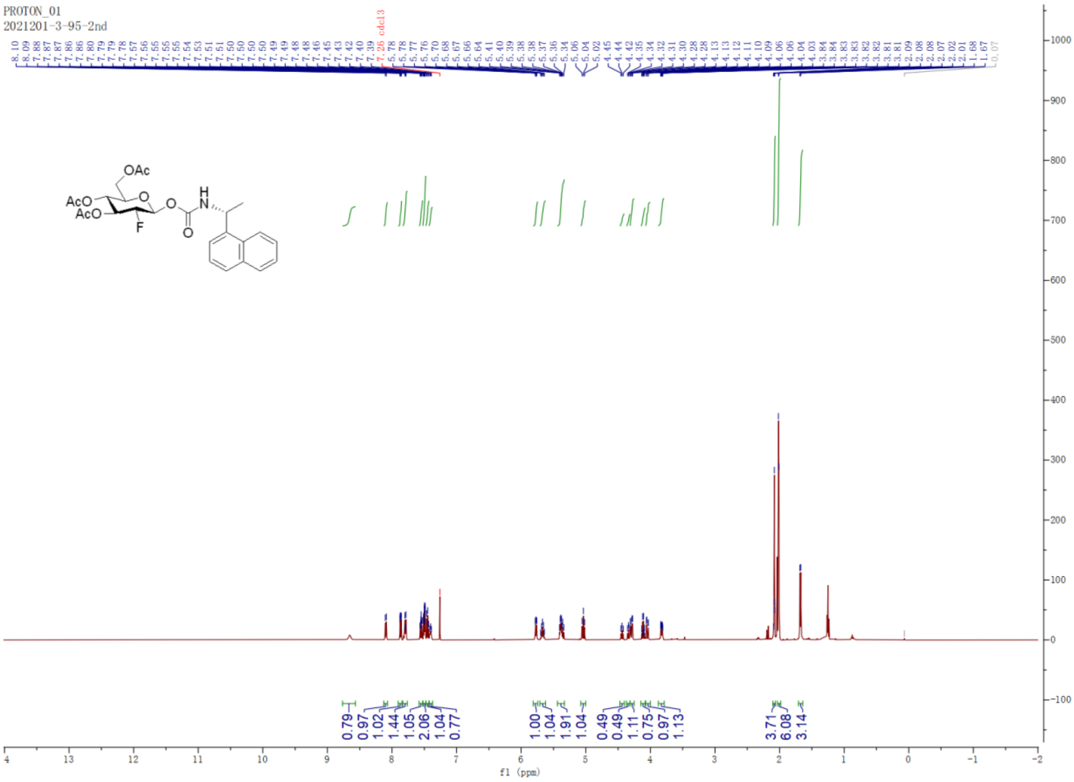

^{1}H NMR spectrum of compound **GA5** (500 MHz, $CDCl_3$)

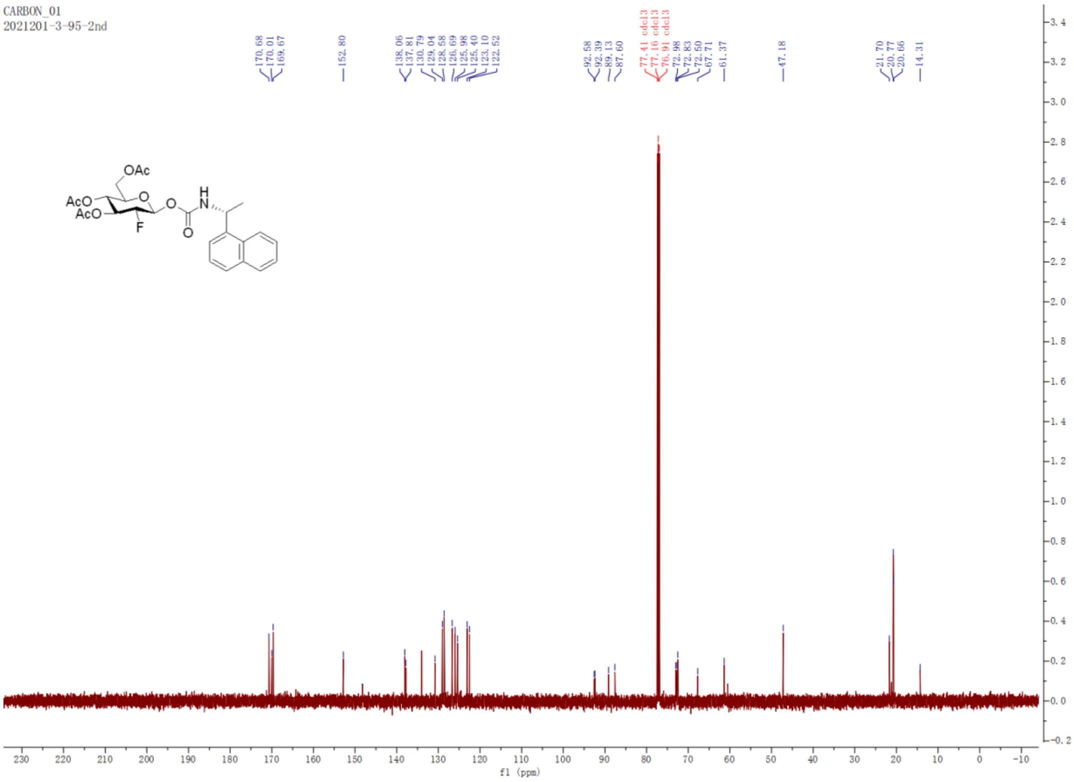

^{13}C NMR spectrum of compound **GA5** (126 MHz, $CDCl_3$)

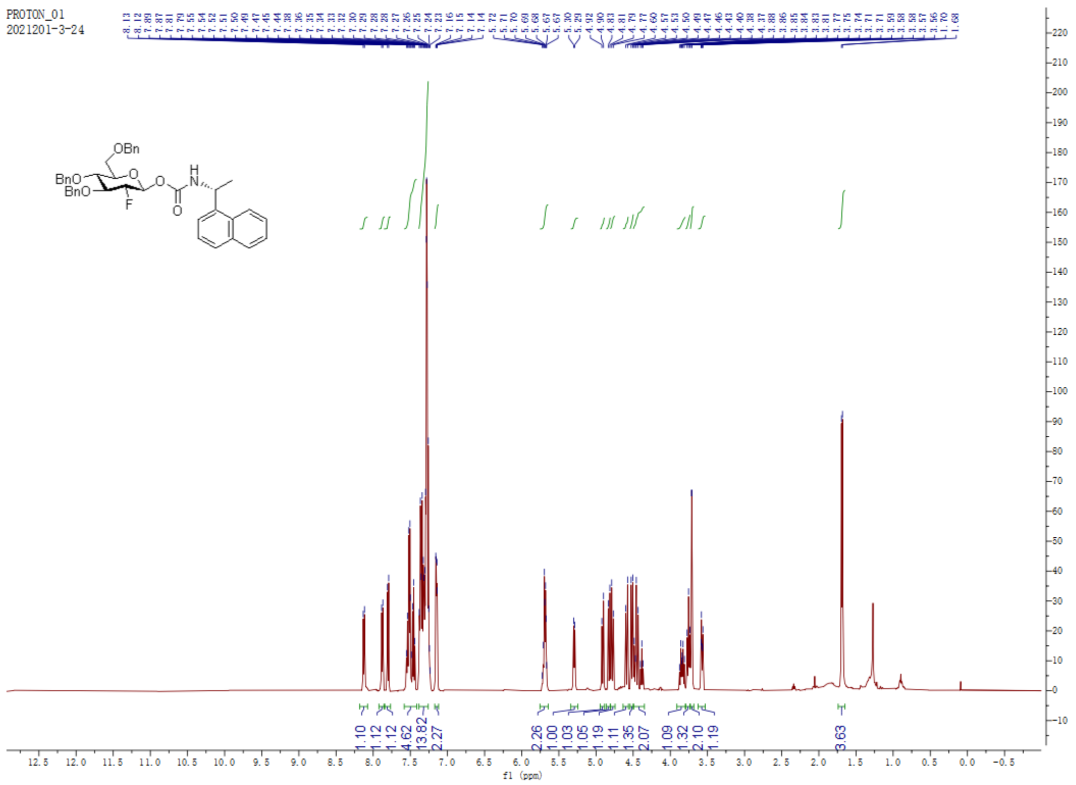

^{1}H NMR spectrum of compound **GA6** (500 MHz, $CDCl_3$)

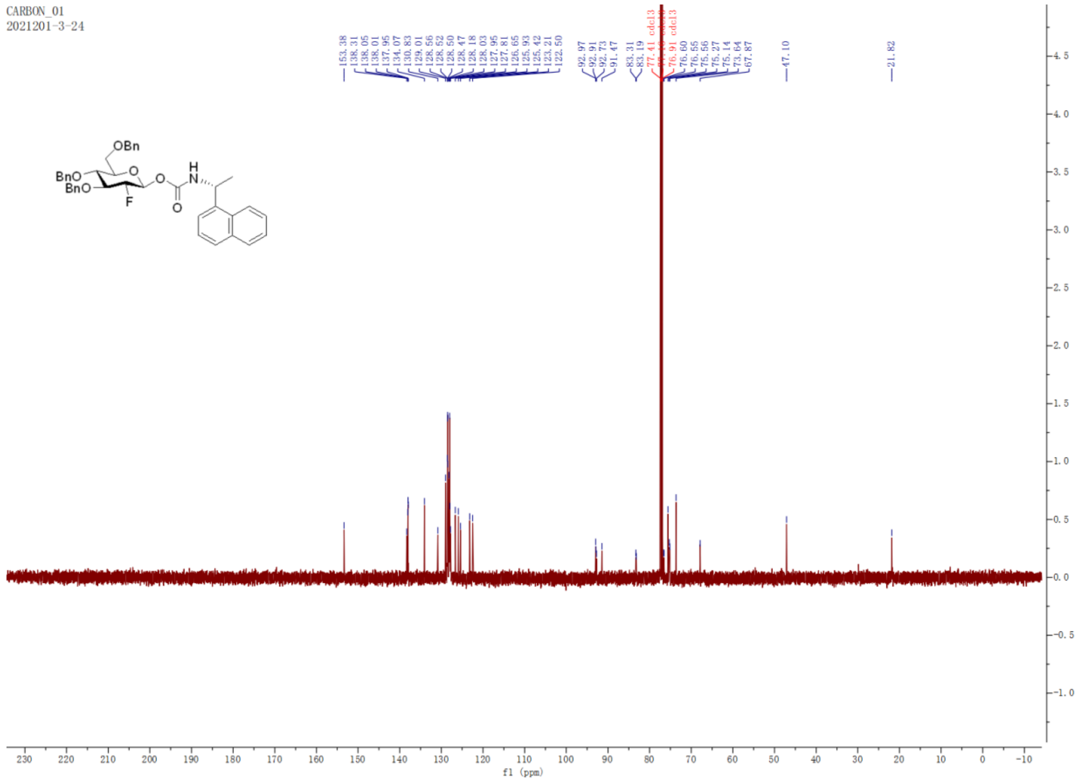

^{13}C NMR spectrum of compound **GA6** (126 MHz, $CDCl_3$)

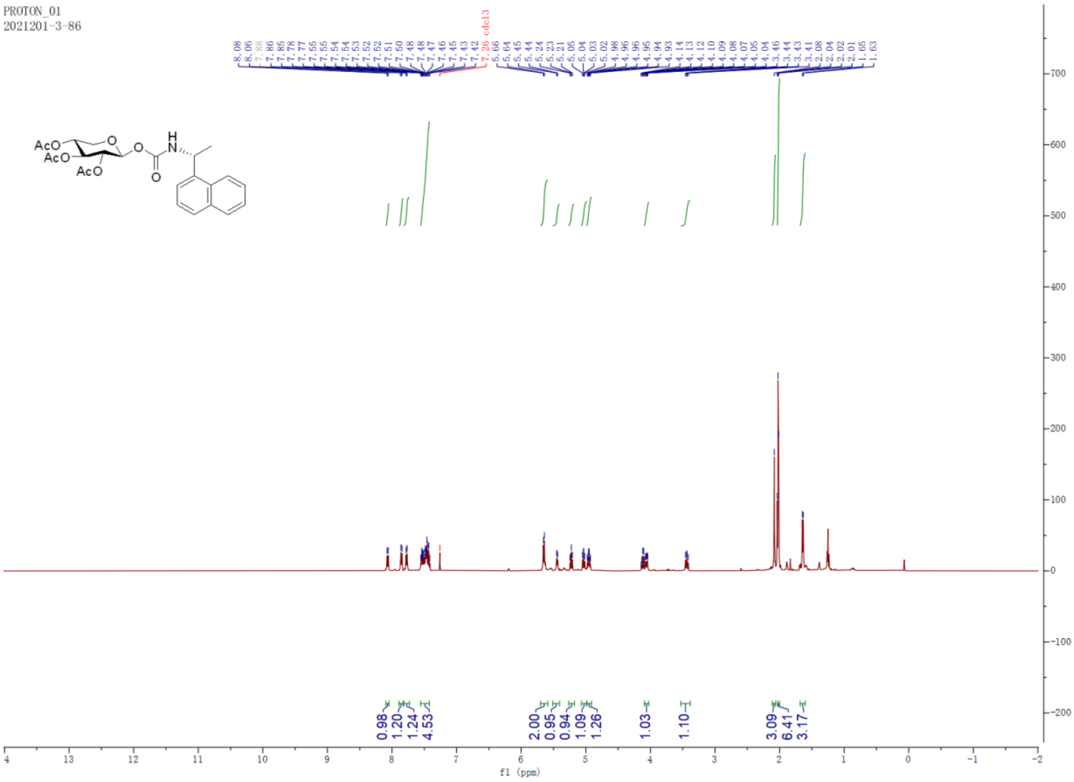

^{1}H NMR spectrum of compound **GA8** (500 MHz, $CDCl_3$)

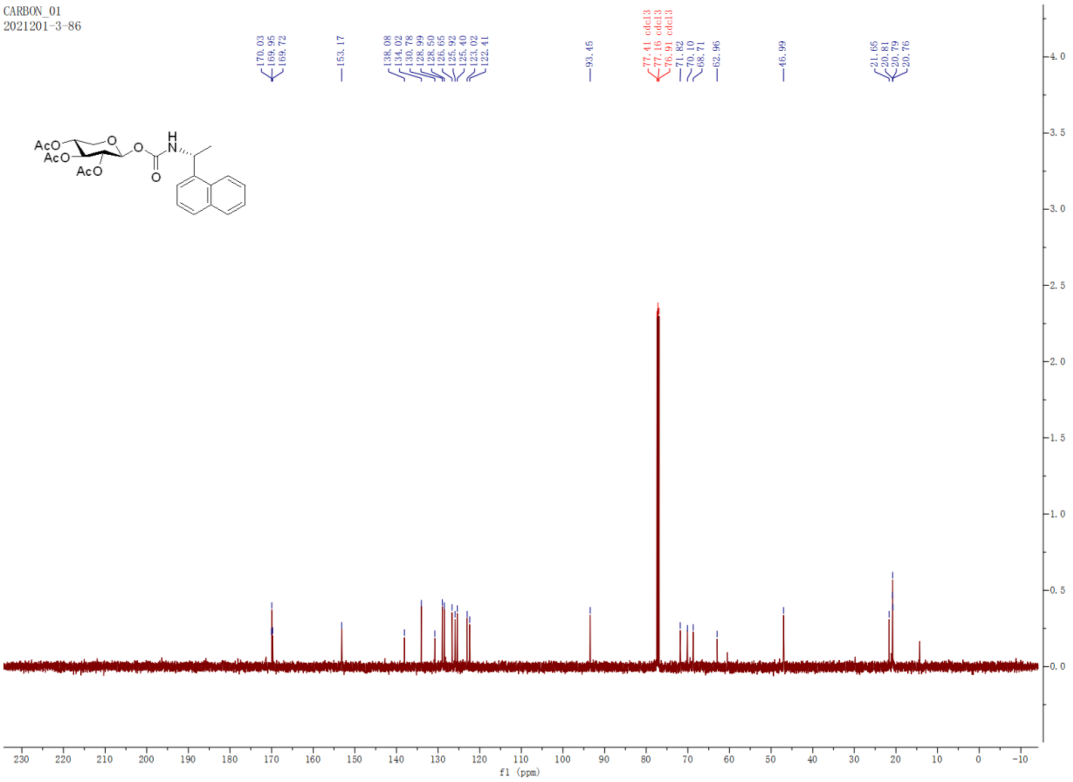

^{13}C NMR spectrum of compound **GA8** (126 MHz, $CDCl_3$)

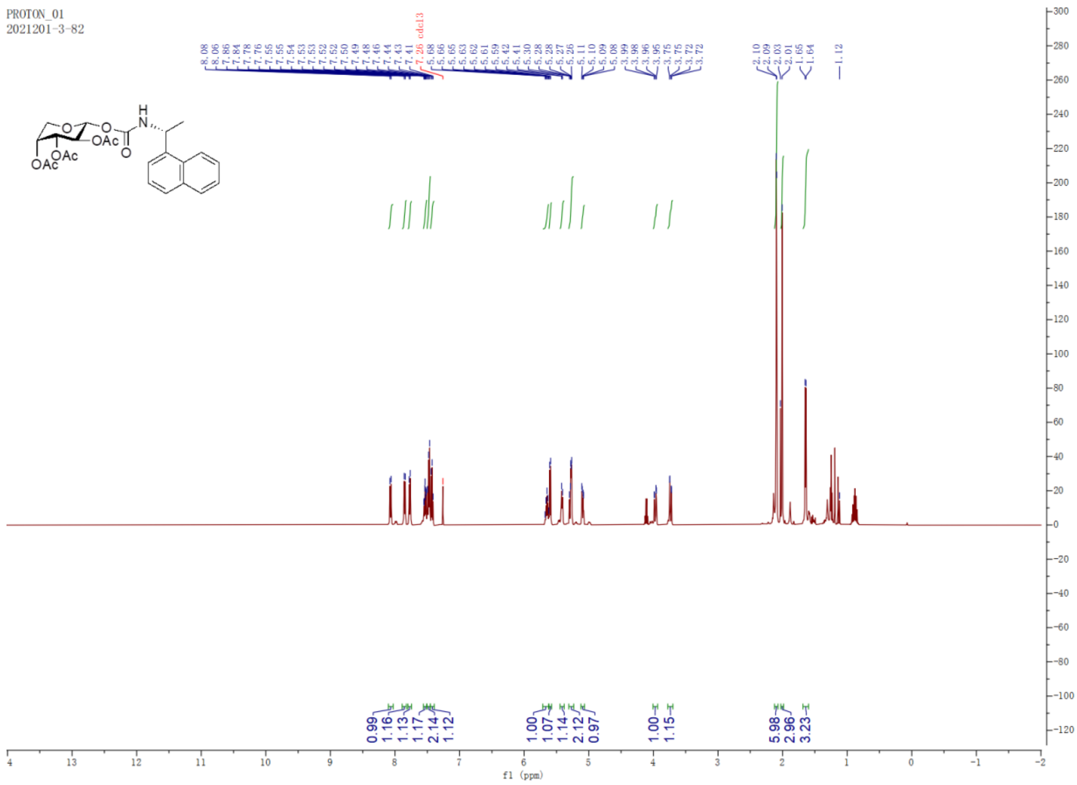

^{1}H NMR spectrum of compound **GA10** (500 MHz, $CDCl_3$)

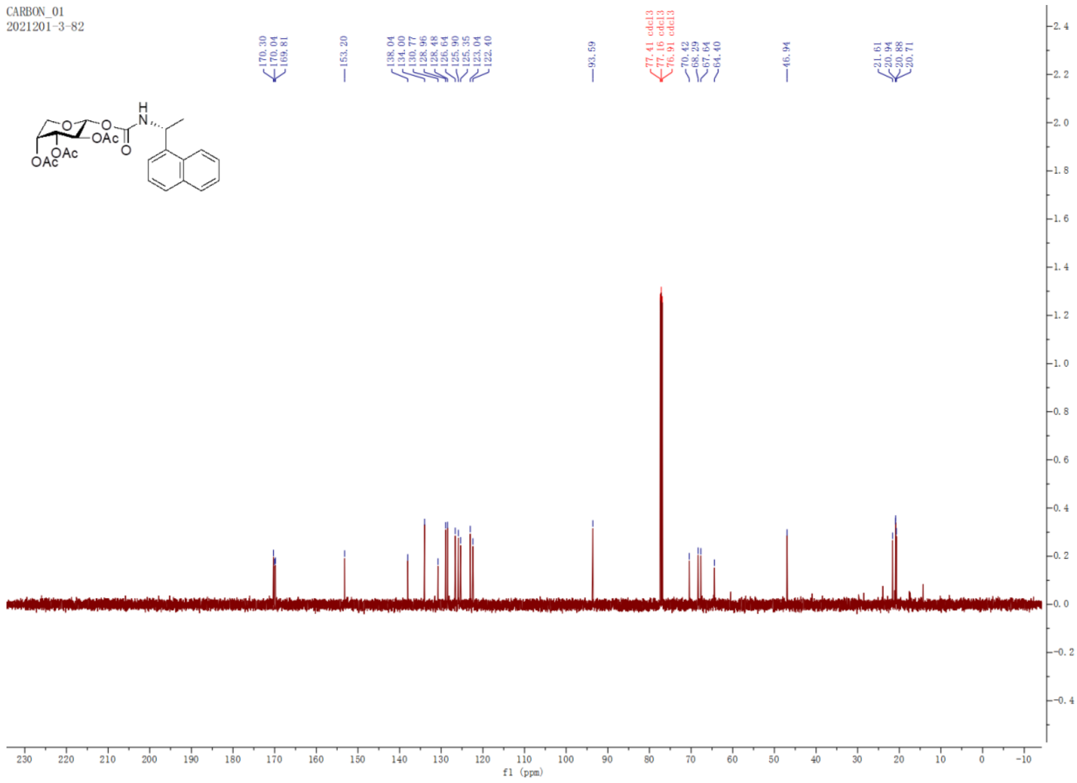

^{13}C NMR spectrum of compound **GA10** (126 MHz, $CDCl_3$)

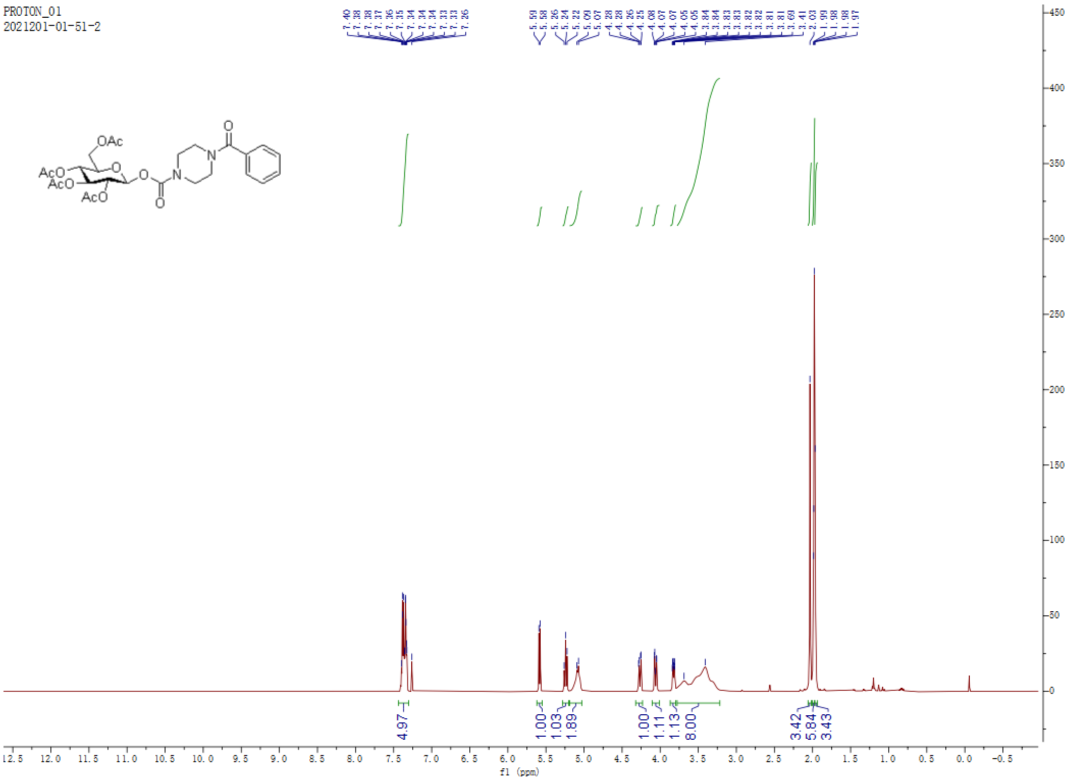

^{1}H NMR spectrum of compound **GA12** (500 MHz, $CDCl_3$)

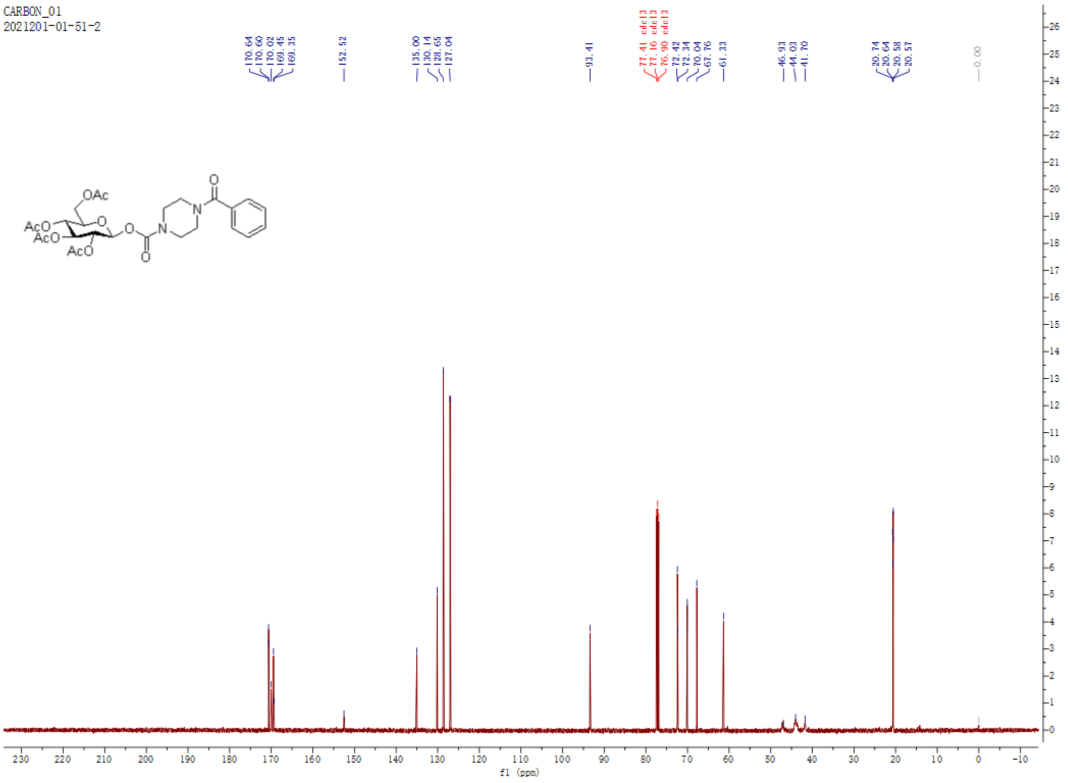

^{13}C NMR spectrum of compound **GA12** (126 MHz, $CDCl_3$)

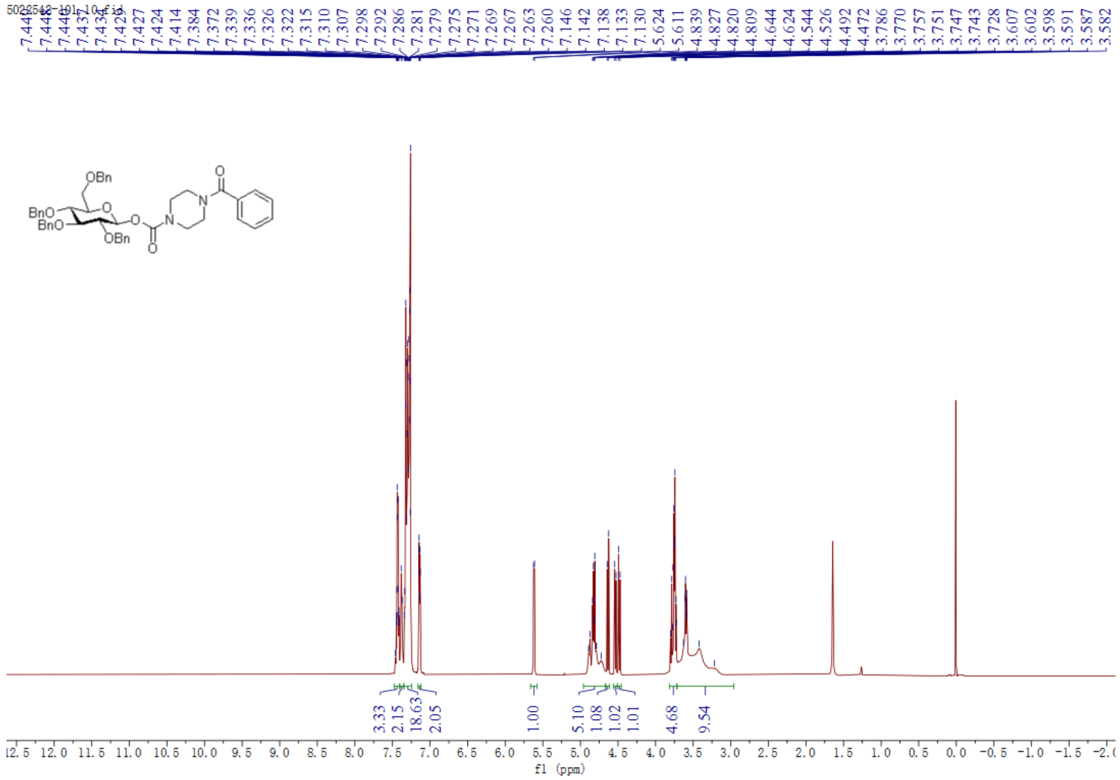

^{1}H NMR spectrum of compound **GA13** (600 MHz, $CDCl_3$)

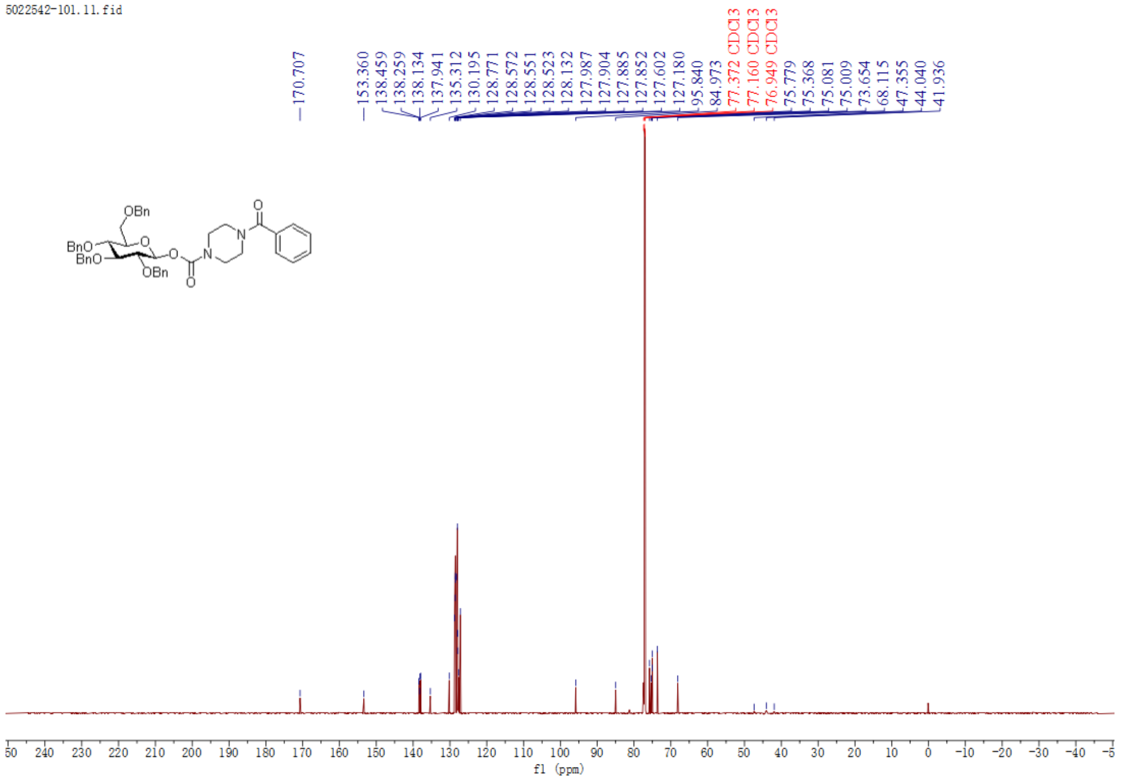

^{13}C NMR spectrum of compound **GA13** (151 MHz, $CDCl_3$)

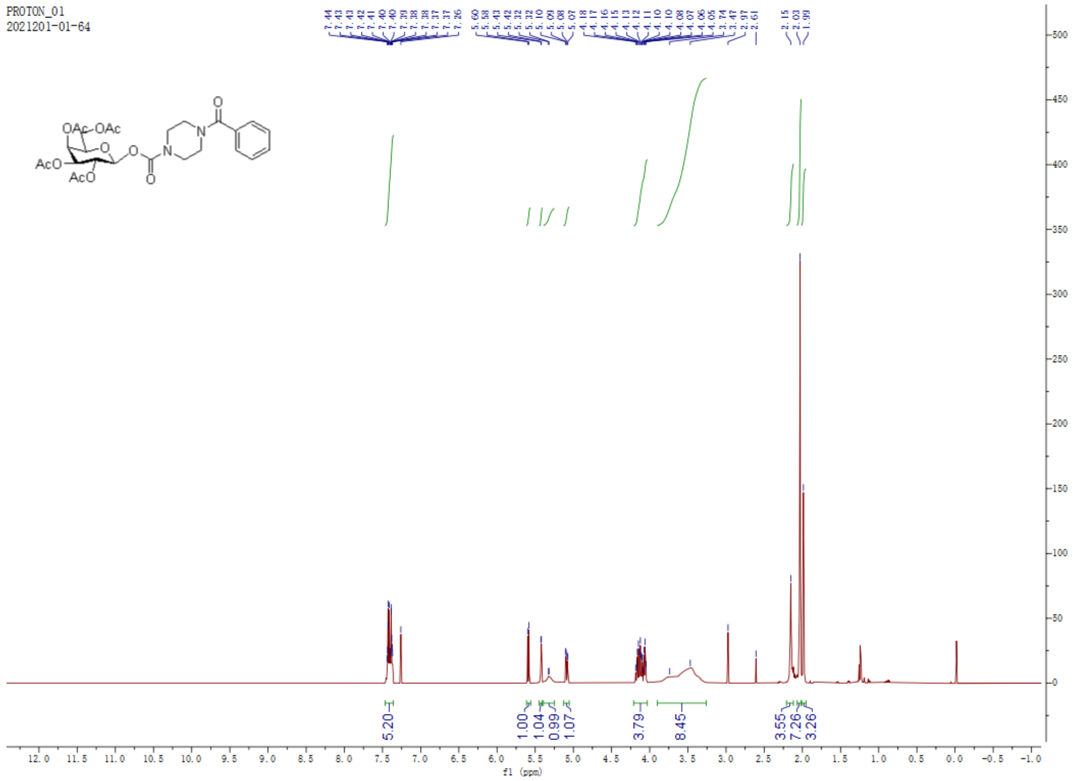

^{1}H NMR spectrum of compound **GA14** (500 MHz, $CDCl_3$)

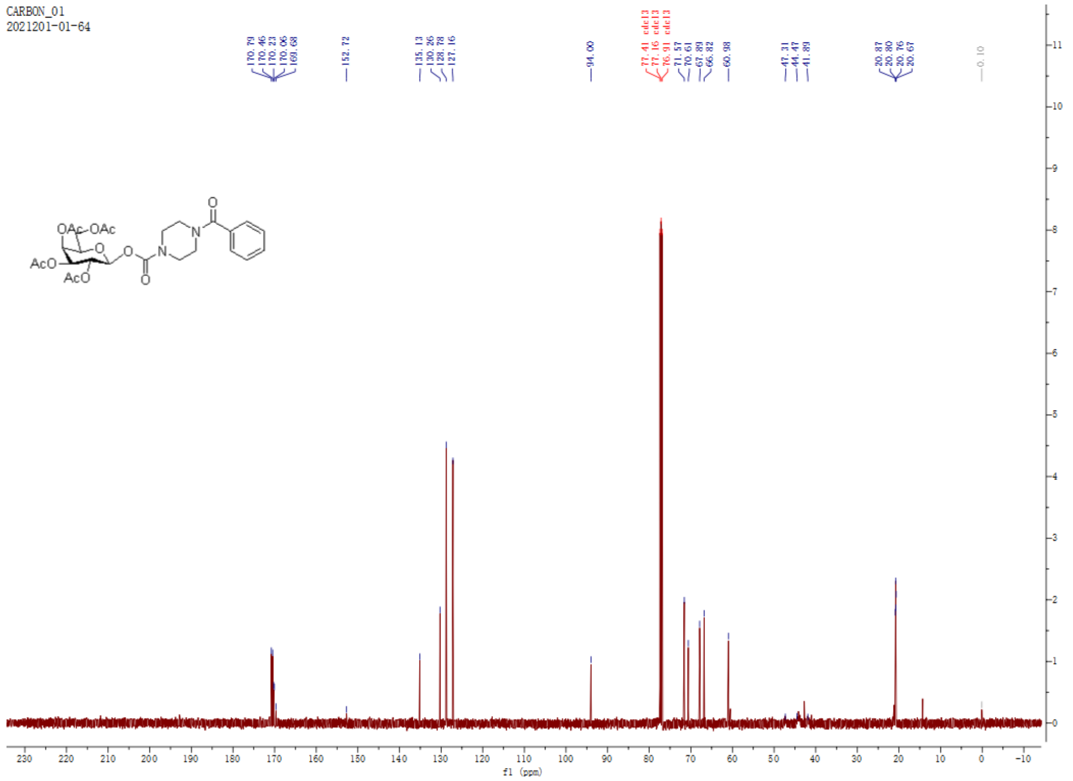

^{13}C NMR spectrum of compound **GA14** (126 MHz, $CDCl_3$)

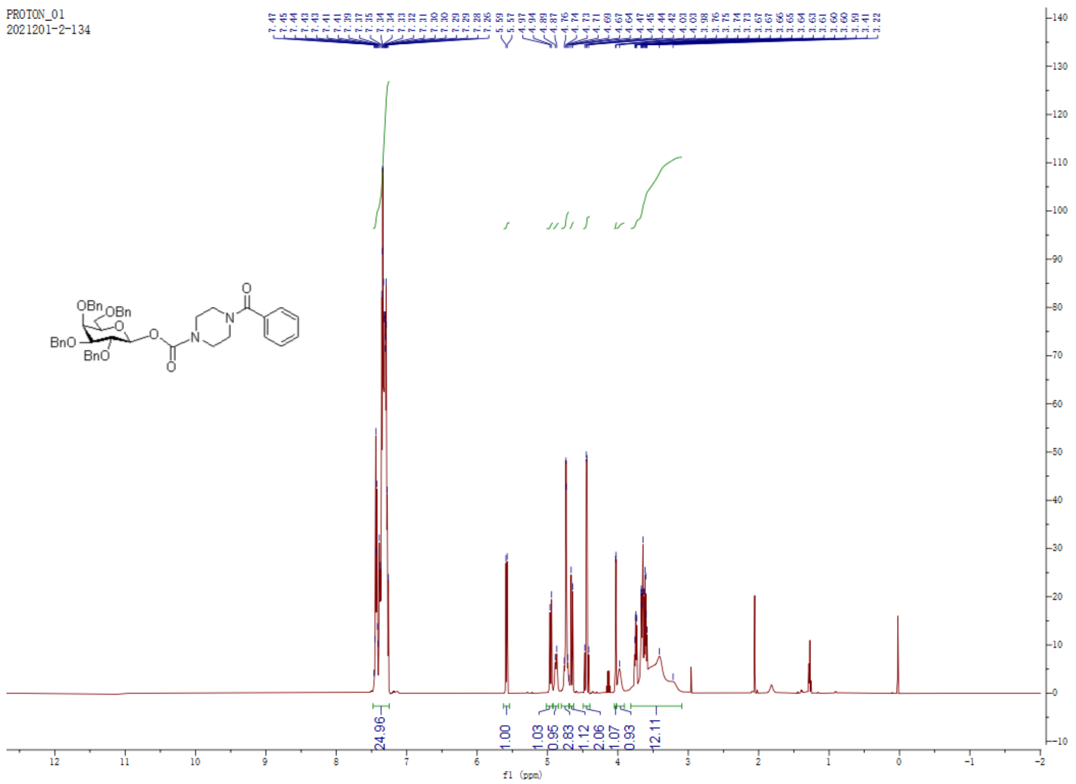

^{1}H NMR spectrum of compound **GA15** (500 MHz, $CDCl_3$)

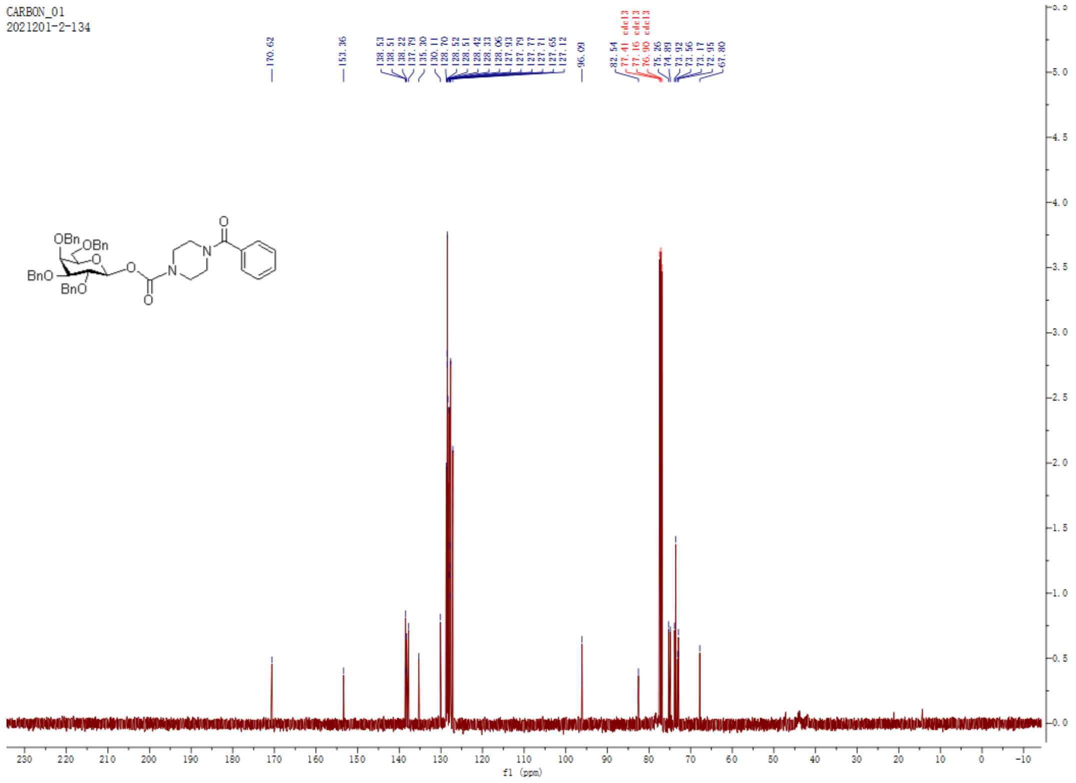

^{13}C NMR spectrum of compound **GA15** (126 MHz, $CDCl_3$)

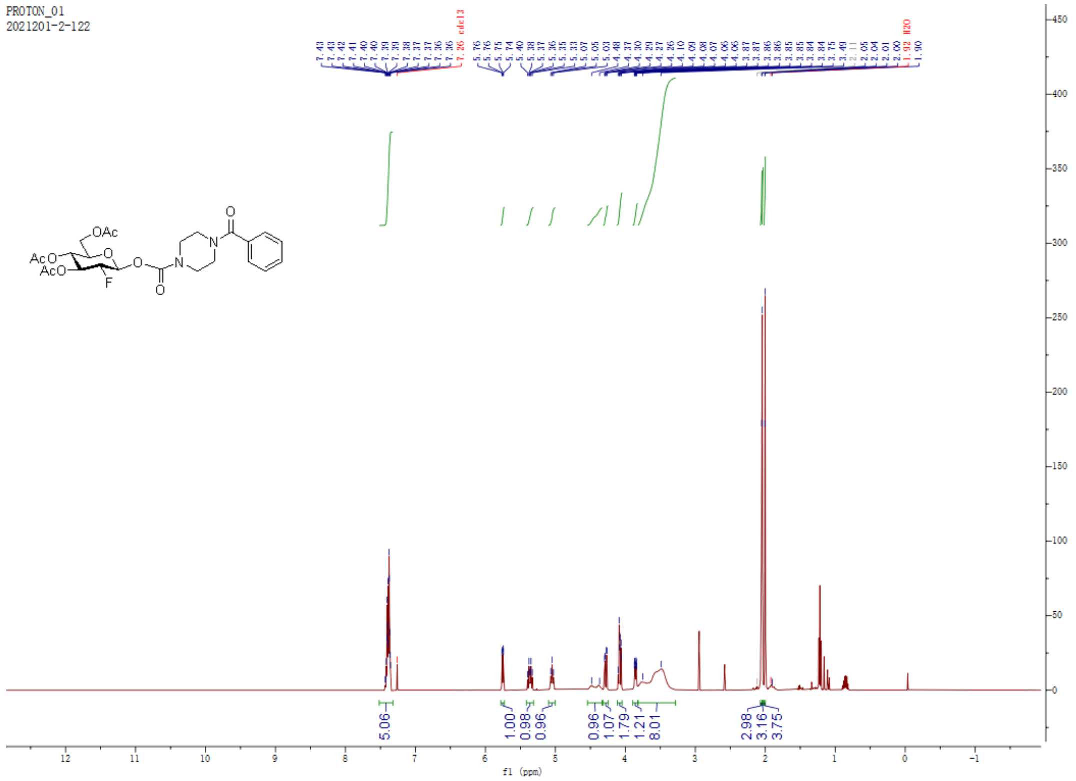

^{1}H NMR spectrum of compound **GA16** (500 MHz, $CDCl_3$)

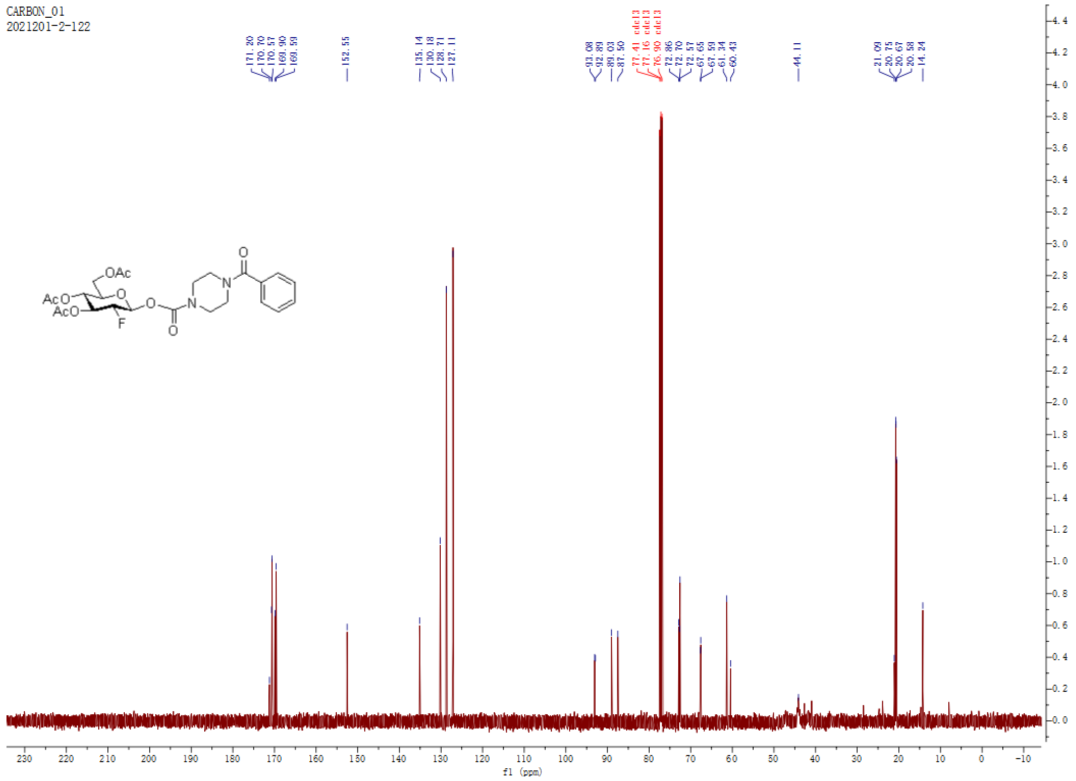

^{13}C NMR spectrum of compound **GA16** (126 MHz, $CDCl_3$)

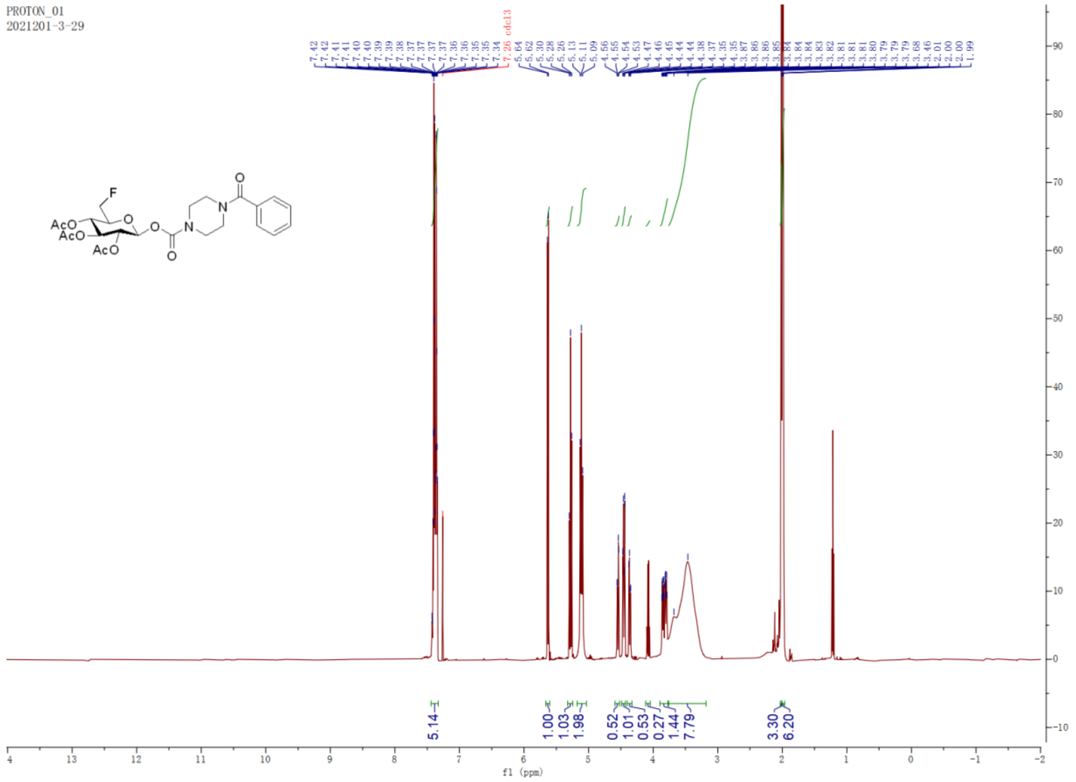

^{1}H NMR spectrum of compound **GA18** (500 MHz, $CDCl_3$)

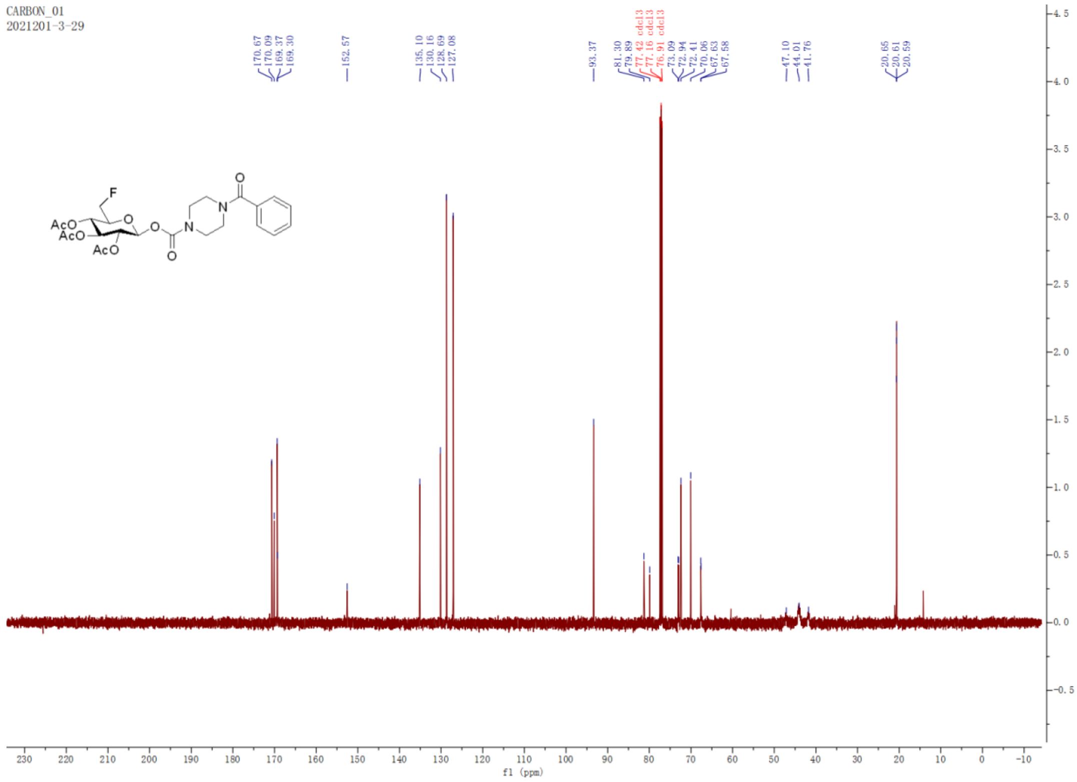

^{13}C NMR spectrum of compound **GA18** (126 MHz, $CDCl_3$)

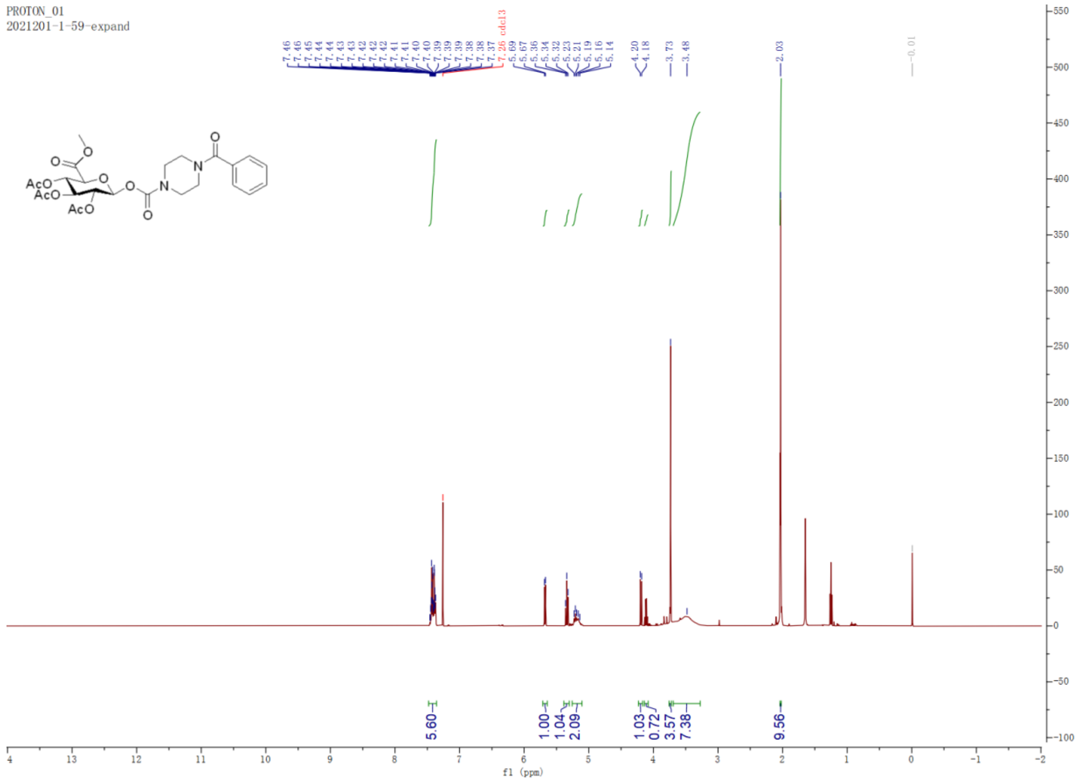

^{1}H NMR spectrum of compound **GA19** (500 MHz, $CDCl_3$)

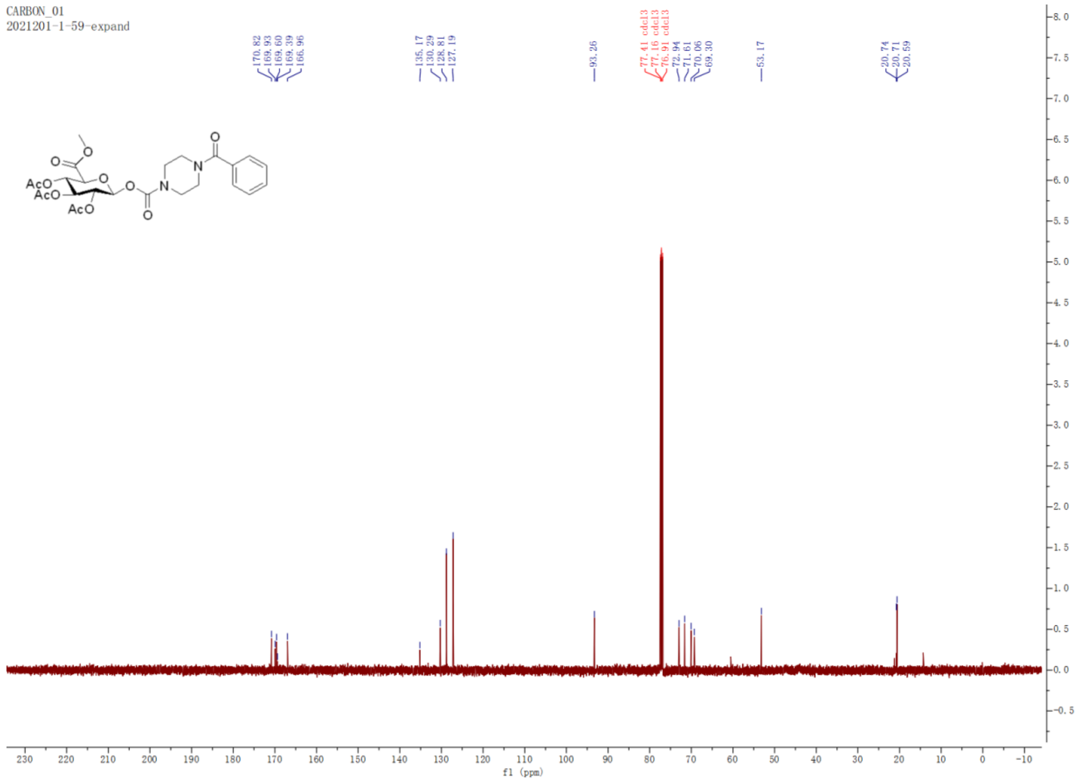

^{13}C NMR spectrum of compound **GA19** (126 MHz, $CDCl_3$)

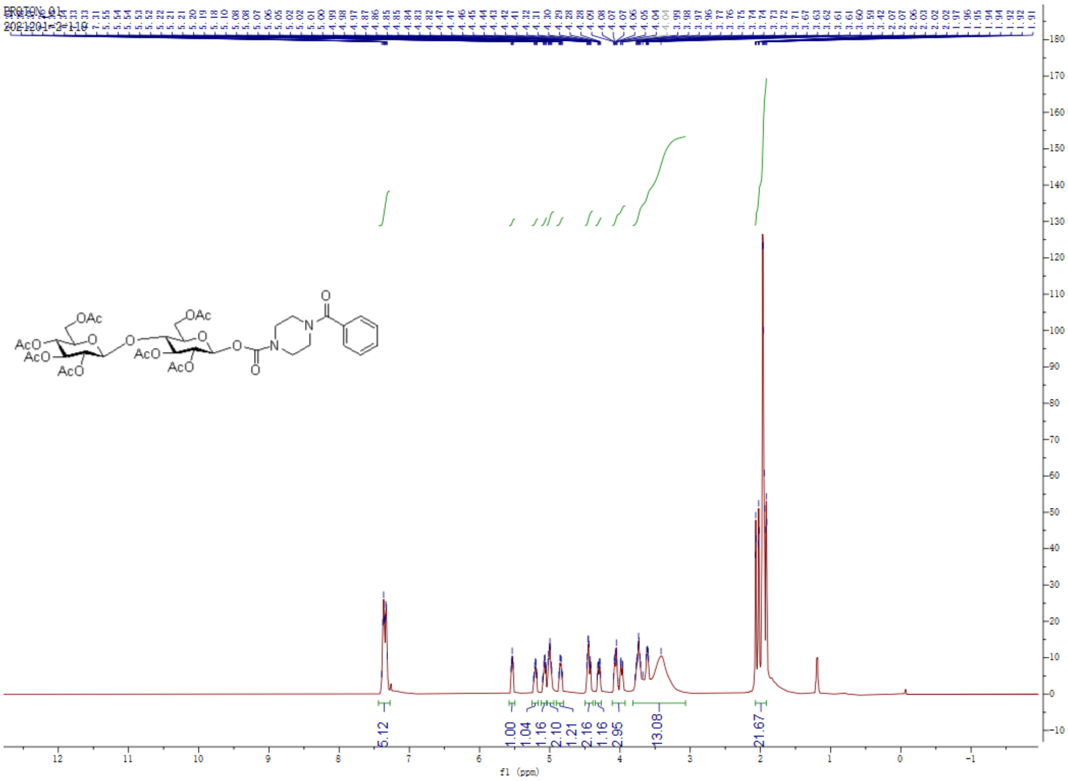

^{1}H NMR spectrum of compound **GA20** (500 MHz, $CDCl_3$)

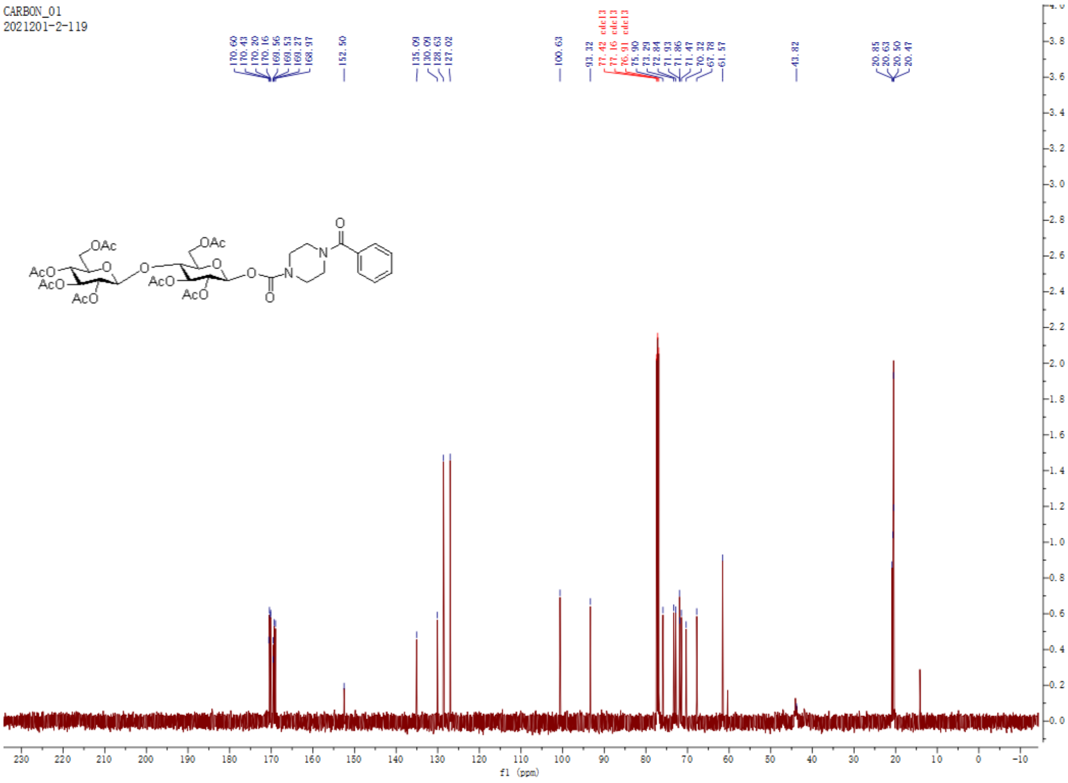

^{13}C NMR spectrum of compound **GA20** (126 MHz, $CDCl_3$)

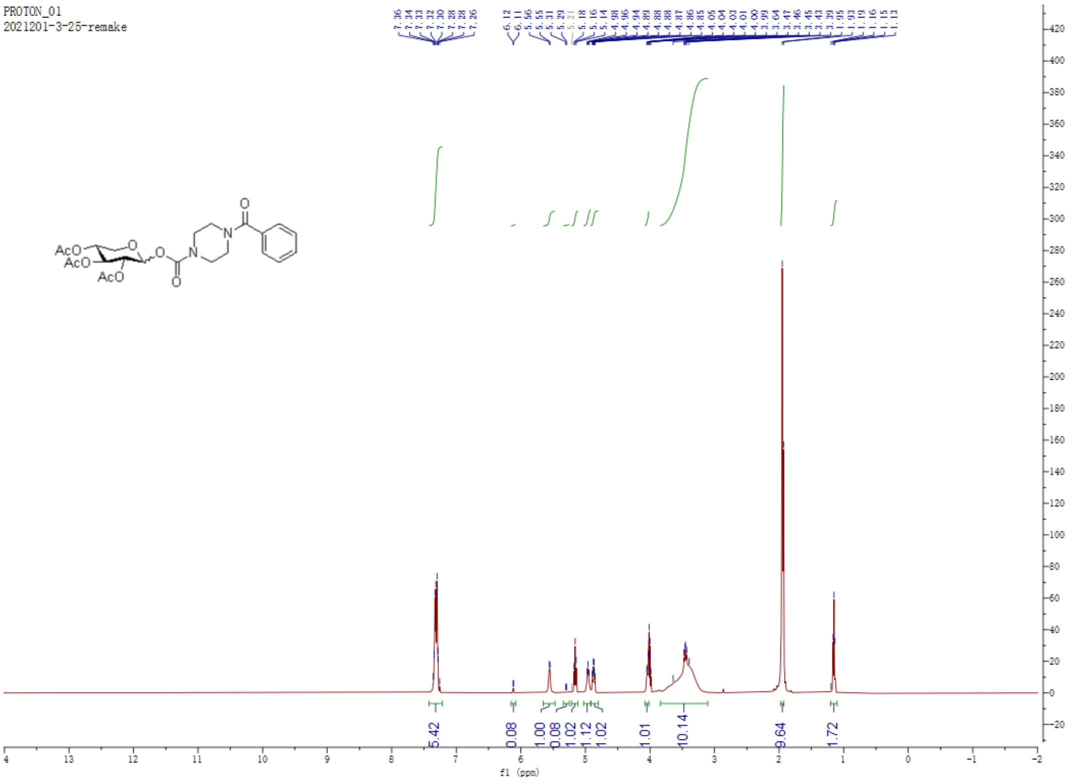

^{1}H NMR spectrum of compound **GA22** (500 MHz, $CDCl_3$)

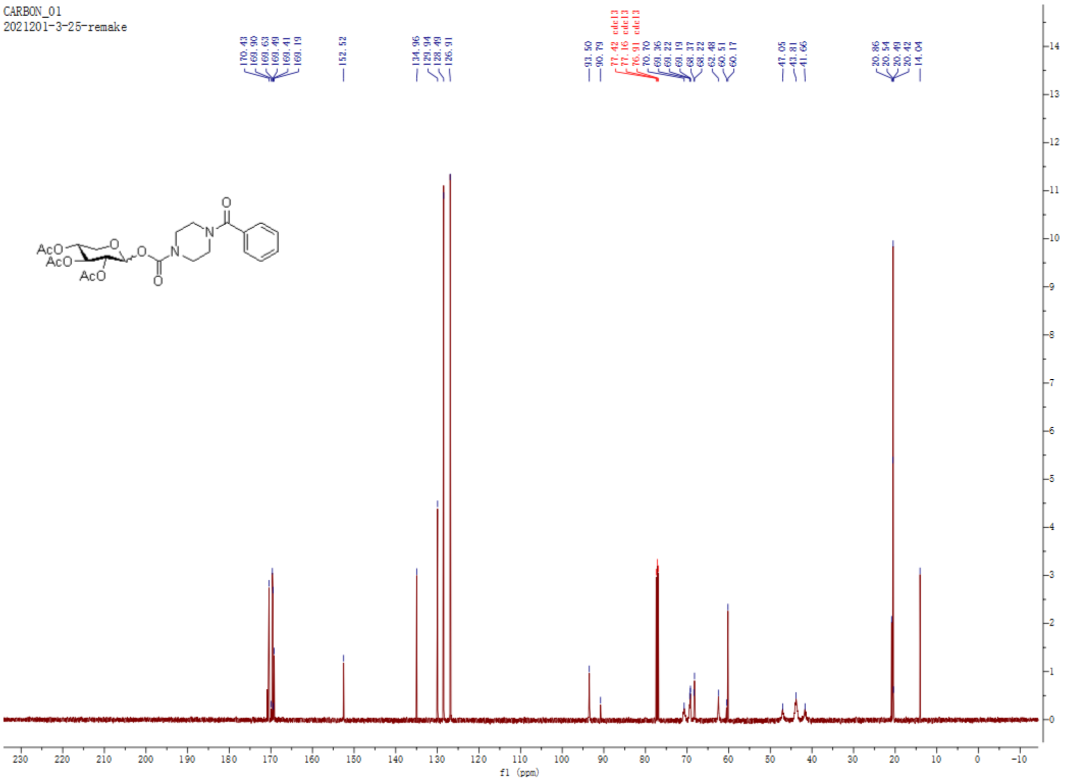

^{13}C NMR spectrum of compound **GA22** (126 MHz, $CDCl_3$)

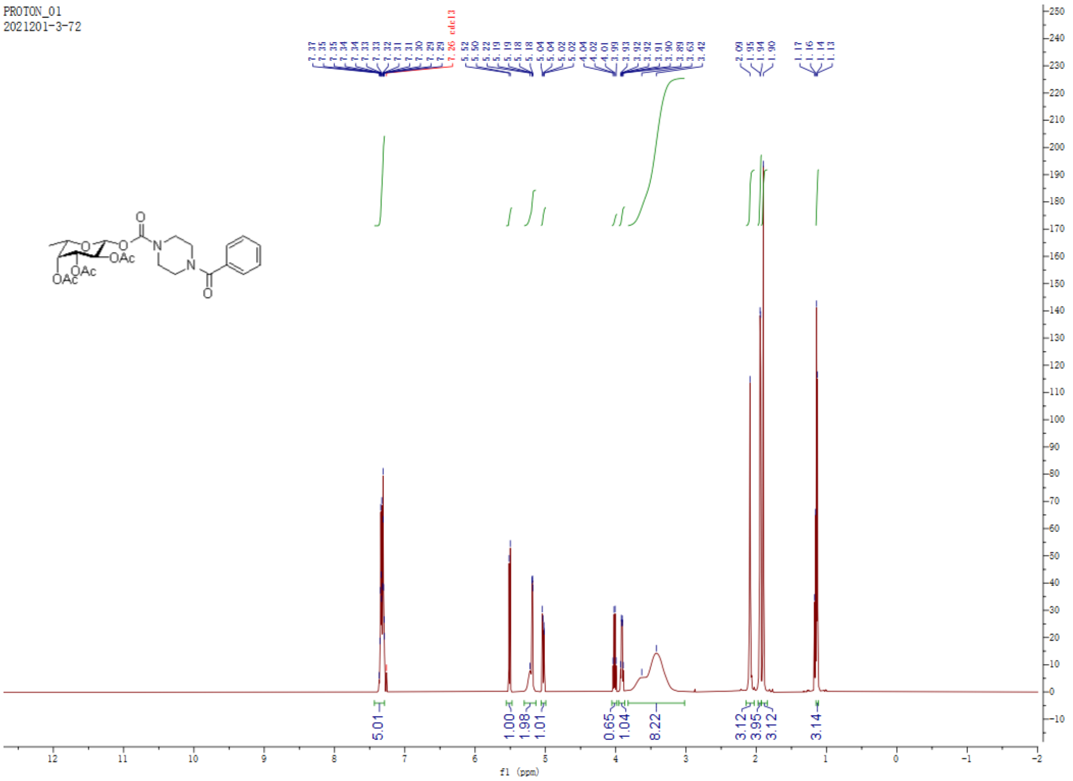

^{1}H NMR spectrum of compound **GA23** (500 MHz, $CDCl_3$)

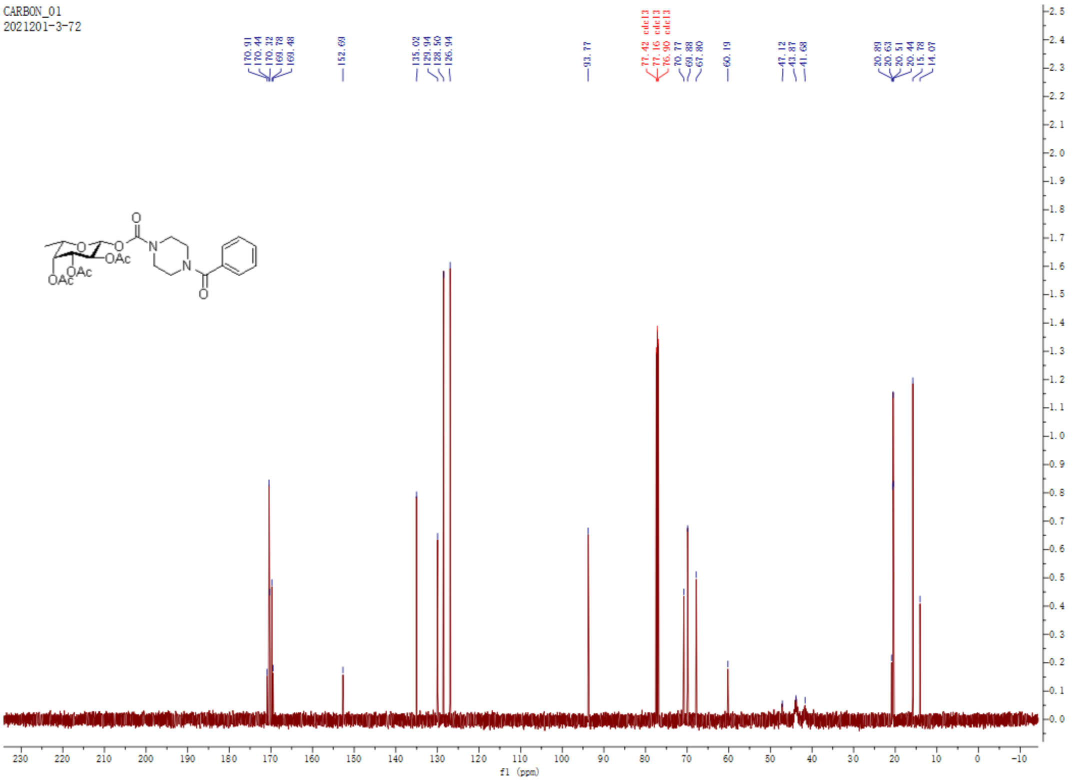

^{13}C NMR spectrum of compound **GA23** (126 MHz, $CDCl_3$)

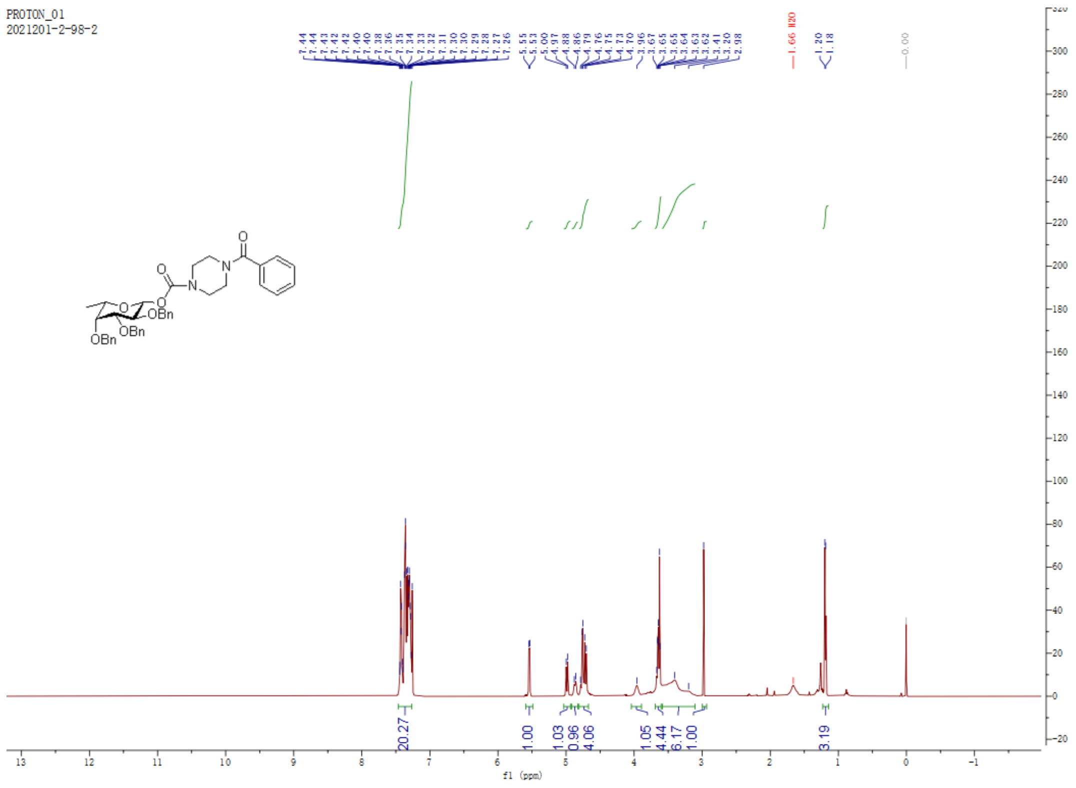

^{1}H NMR spectrum of compound **GA24** (500 MHz, $CDCl_3$)

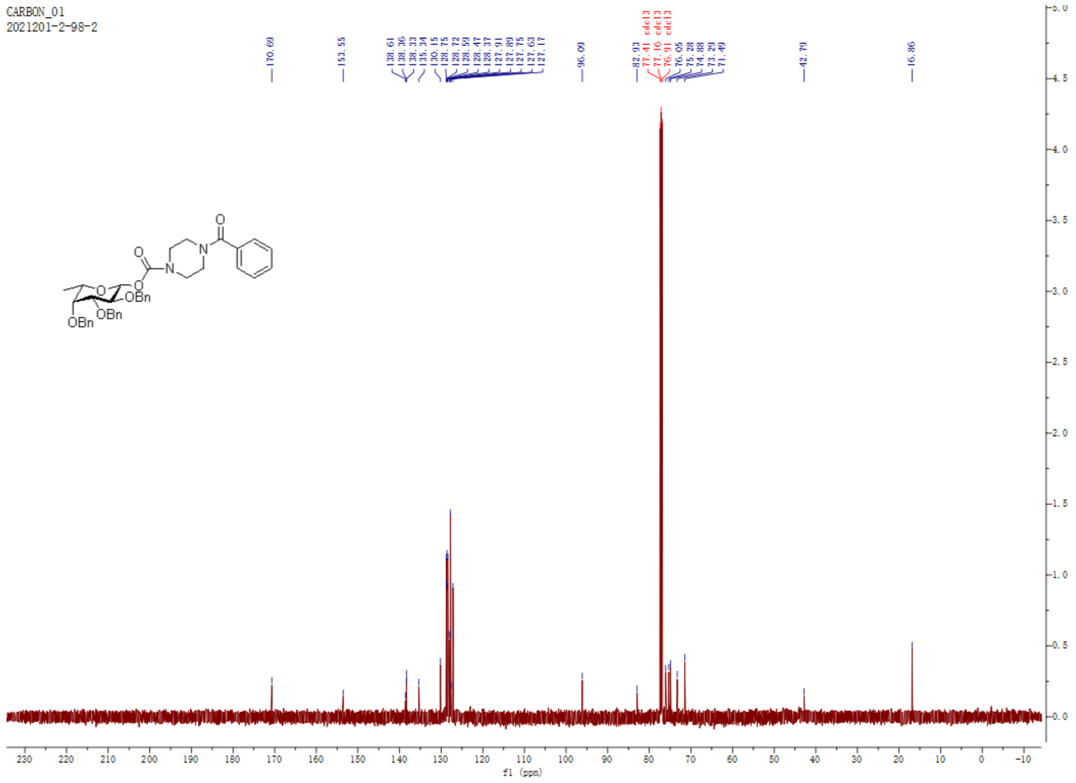

^{13}C NMR spectrum of compound **GA24** (126 MHz, $CDCl_3$)

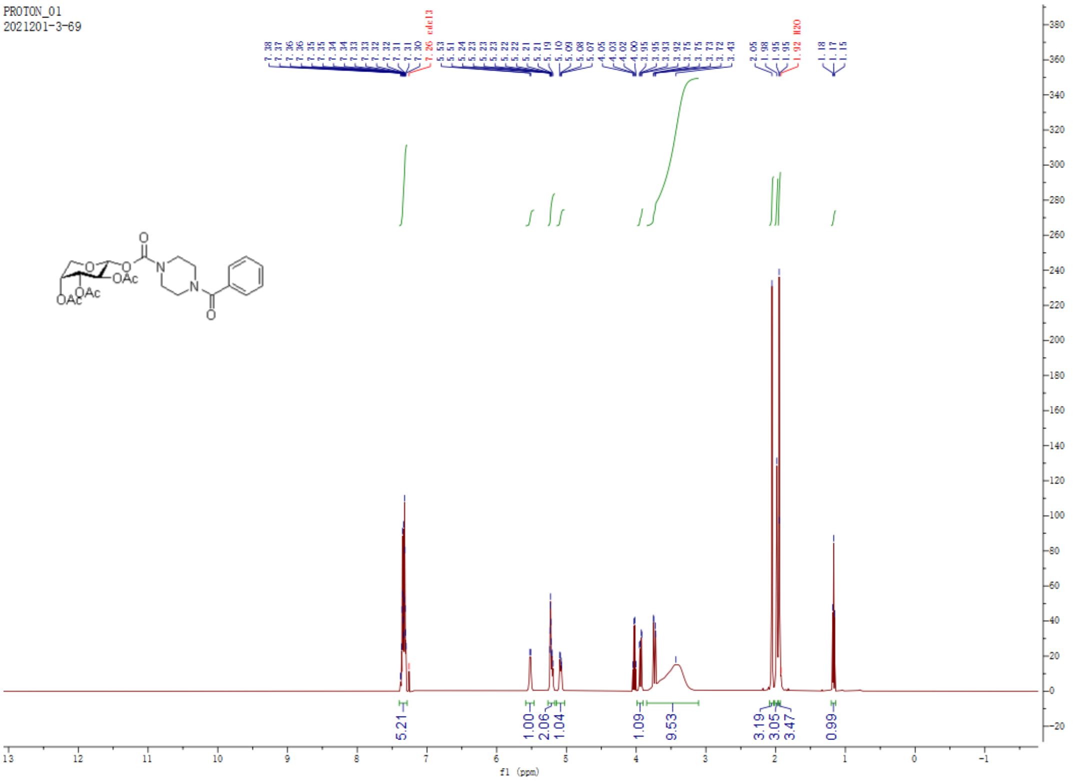

^{1}H NMR spectrum of compound **GA25** (500 MHz, $CDCl_3$)

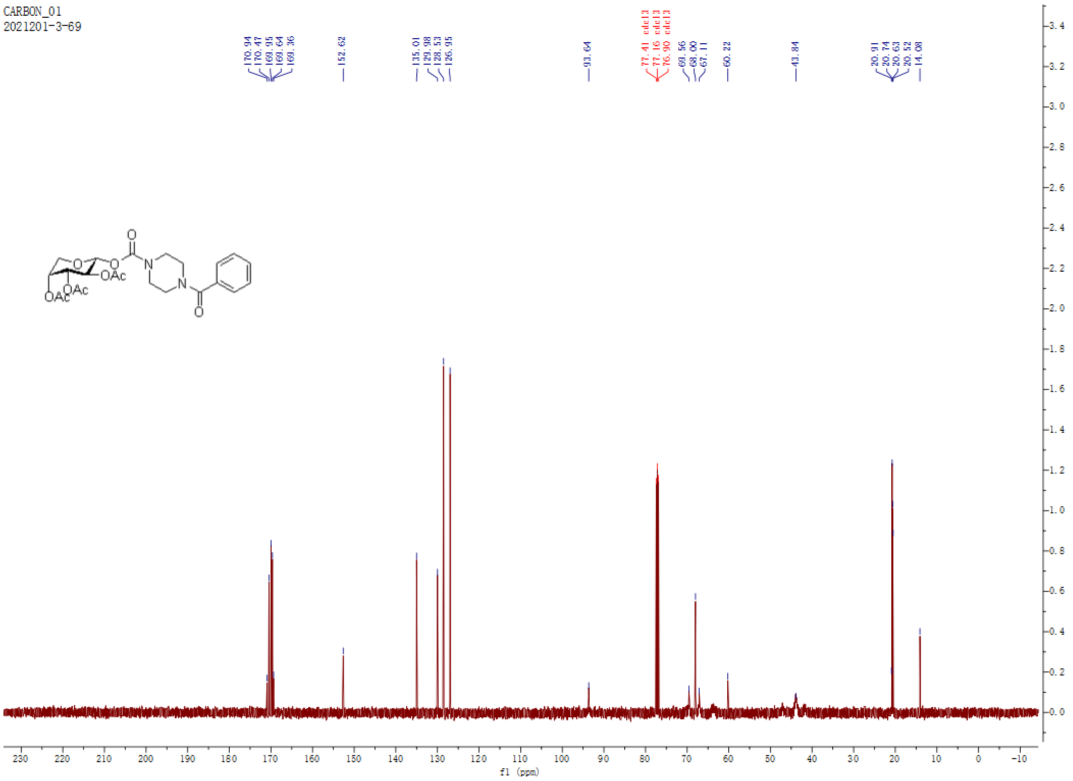

^{13}C NMR spectrum of compound **GA25** (126 MHz, $CDCl_3$)

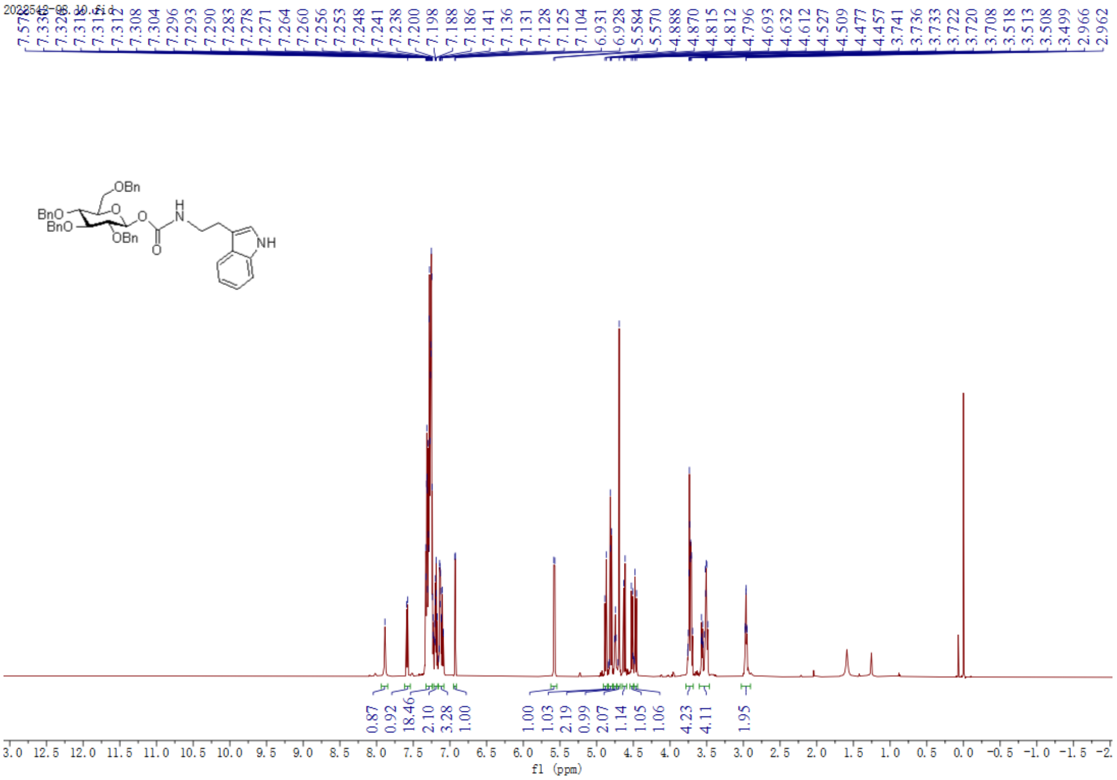

^{1}H NMR spectrum of compound **GA35** (600 MHz, $CDCl_3$)

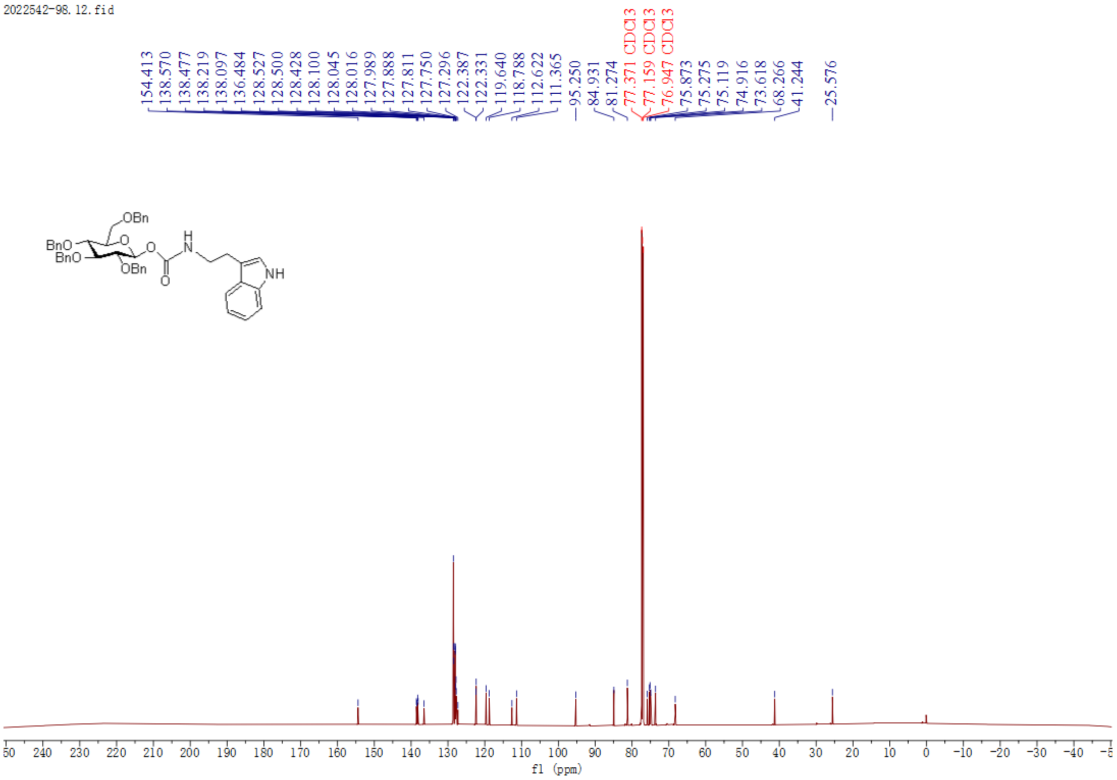

^{13}C NMR spectrum of compound **GA35** (151 MHz, $CDCl_3$)

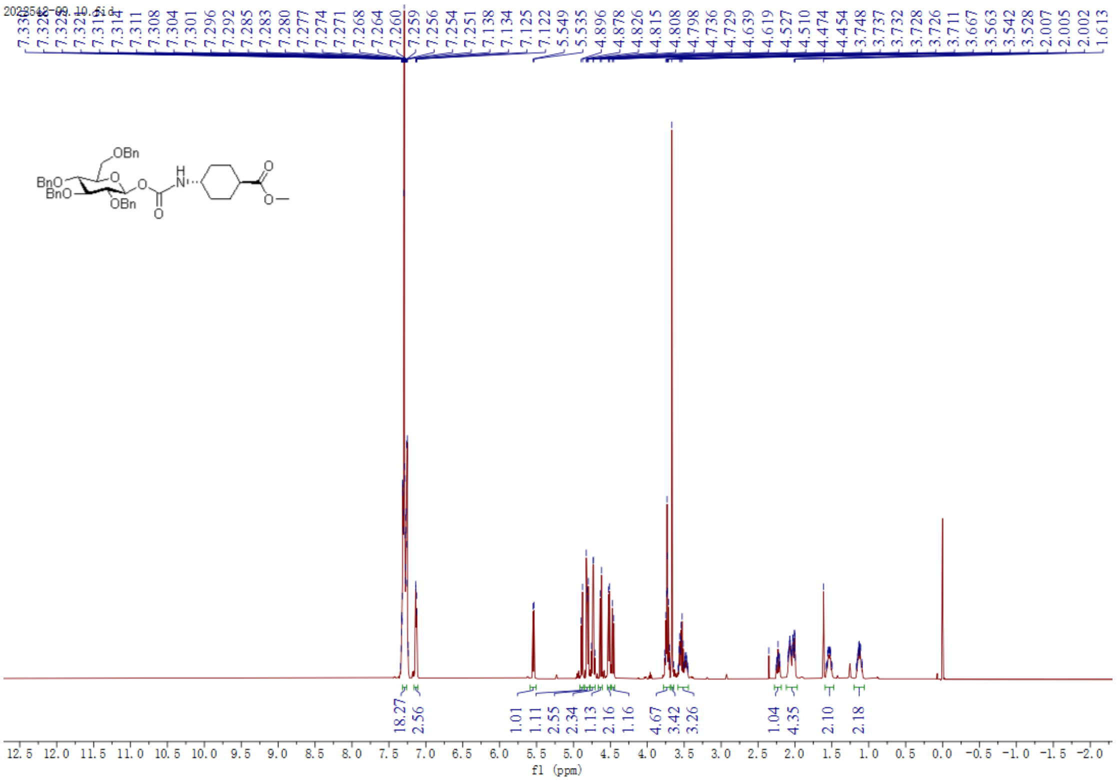

^{1}H NMR spectrum of compound **GA36** (600 MHz, $CDCl_3$)

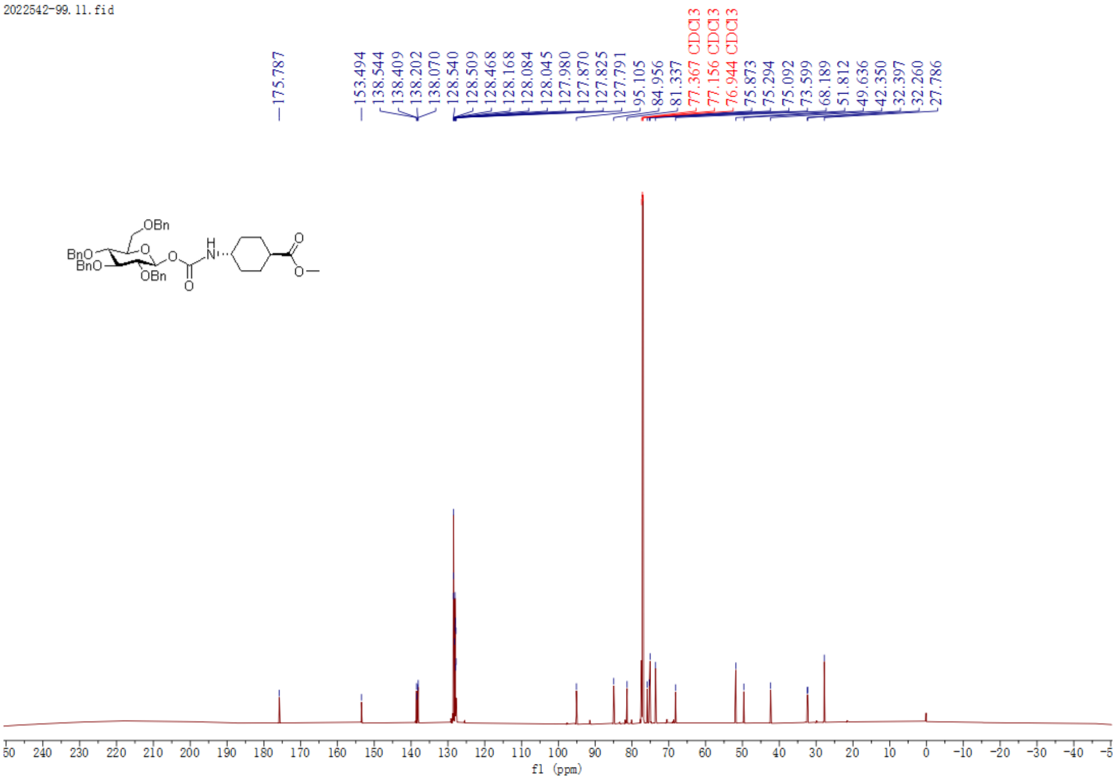

^{13}C NMR spectrum of compound **GA36** (151 MHz, $CDCl_3$)

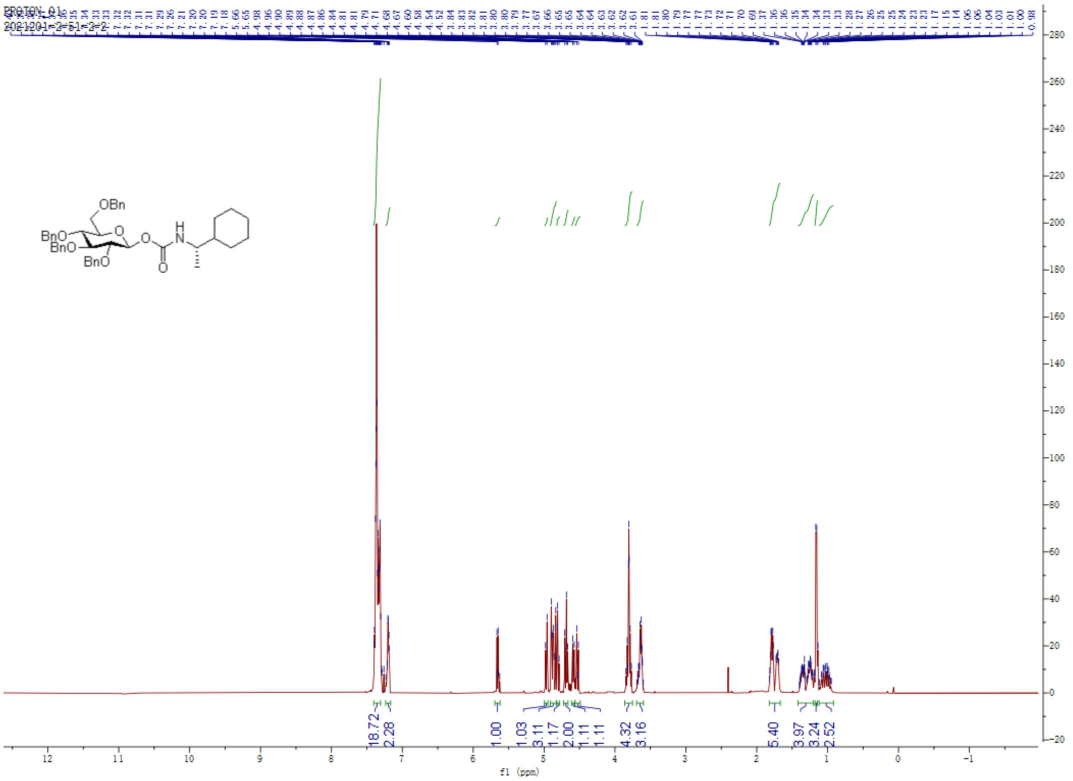

^{1}H NMR spectrum of compound **GA37** (500 MHz, $CDCl_3$)

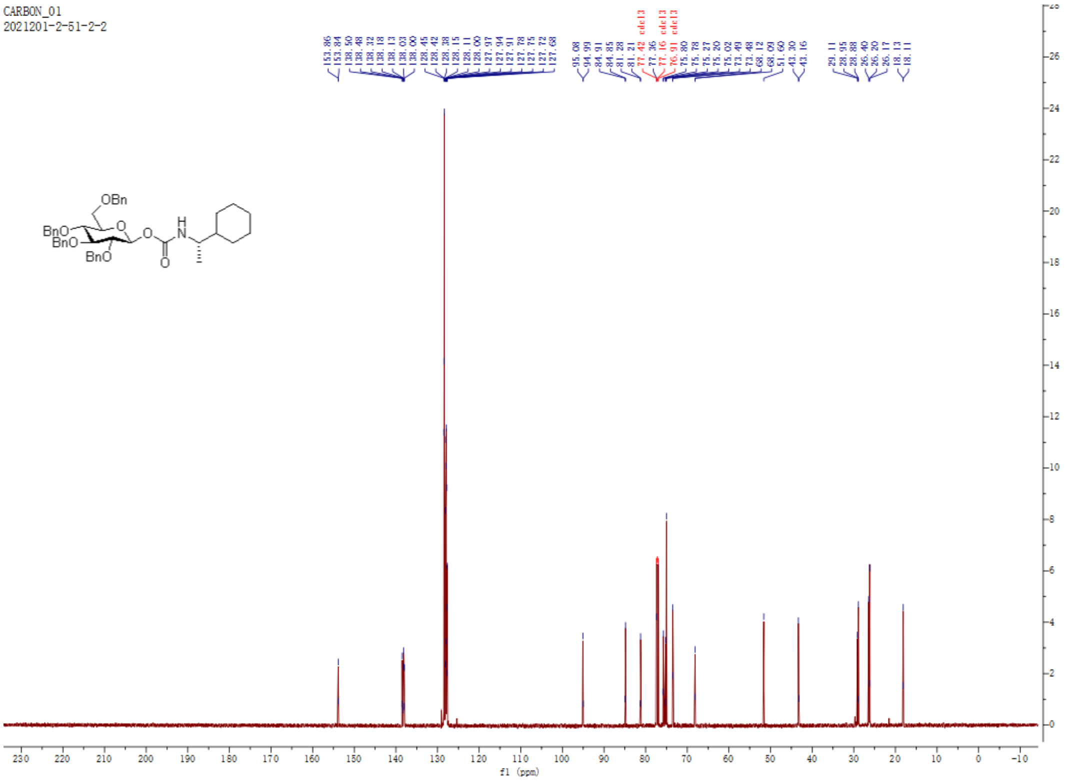

^{13}C NMR spectrum of compound **GA37** (126 MHz, $CDCl_3$)

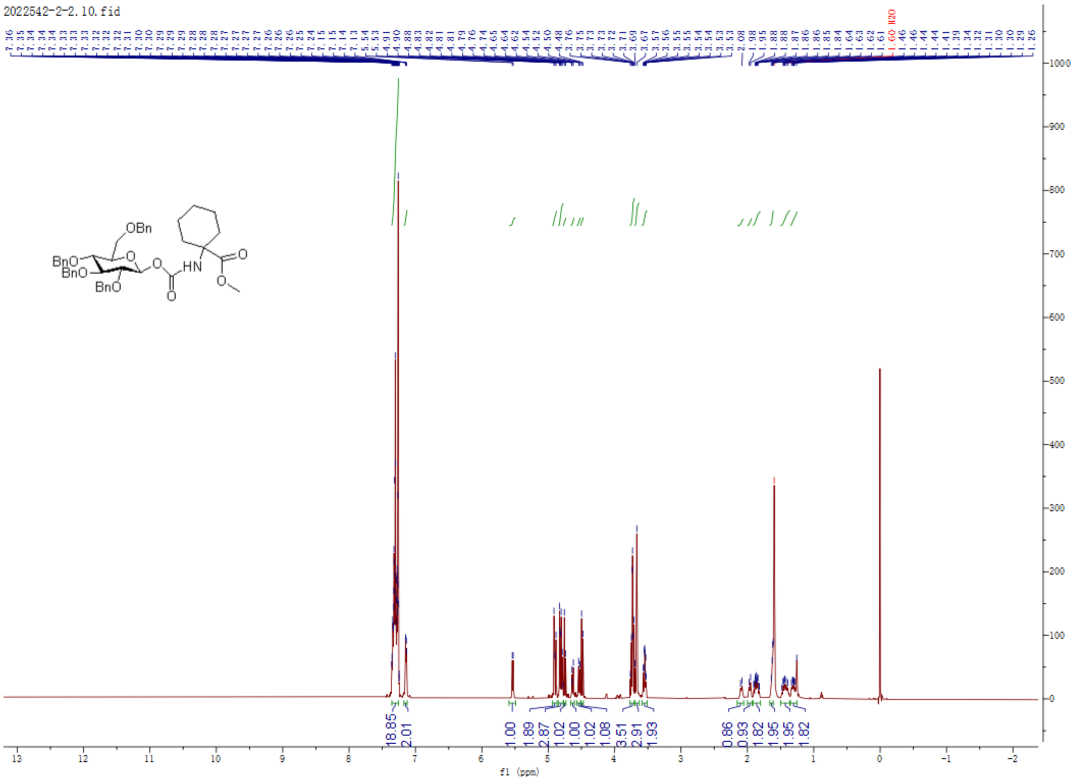

^{1}H NMR spectrum of compound **GA38** (600 MHz, $CDCl_3$)

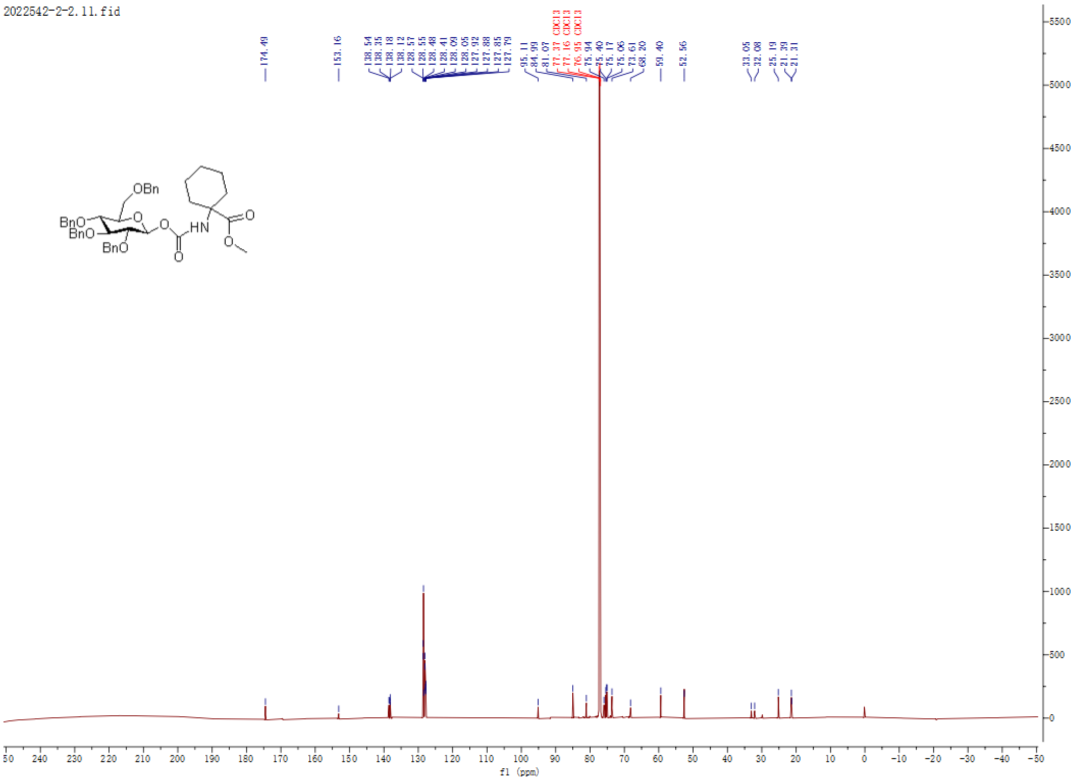

^{13}C NMR spectrum of compound **GA38** (151 MHz, $CDCl_3$)

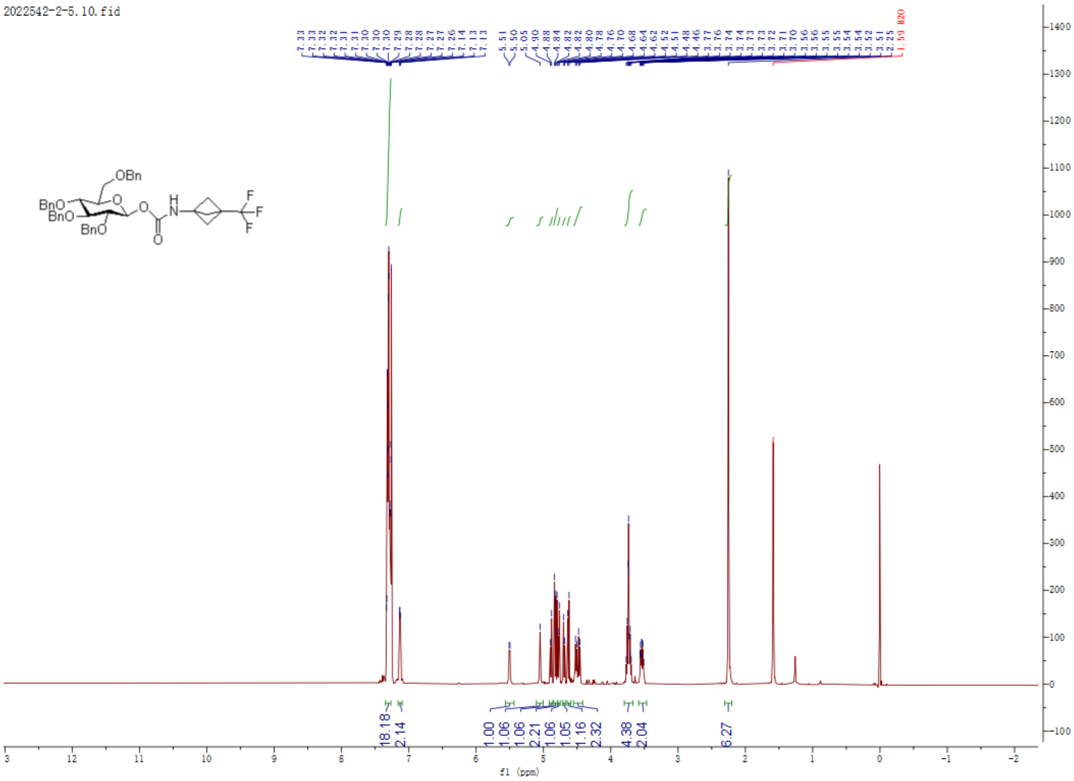

^{1}H NMR spectrum of compound **GA39** (600 MHz, $CDCl_3$)

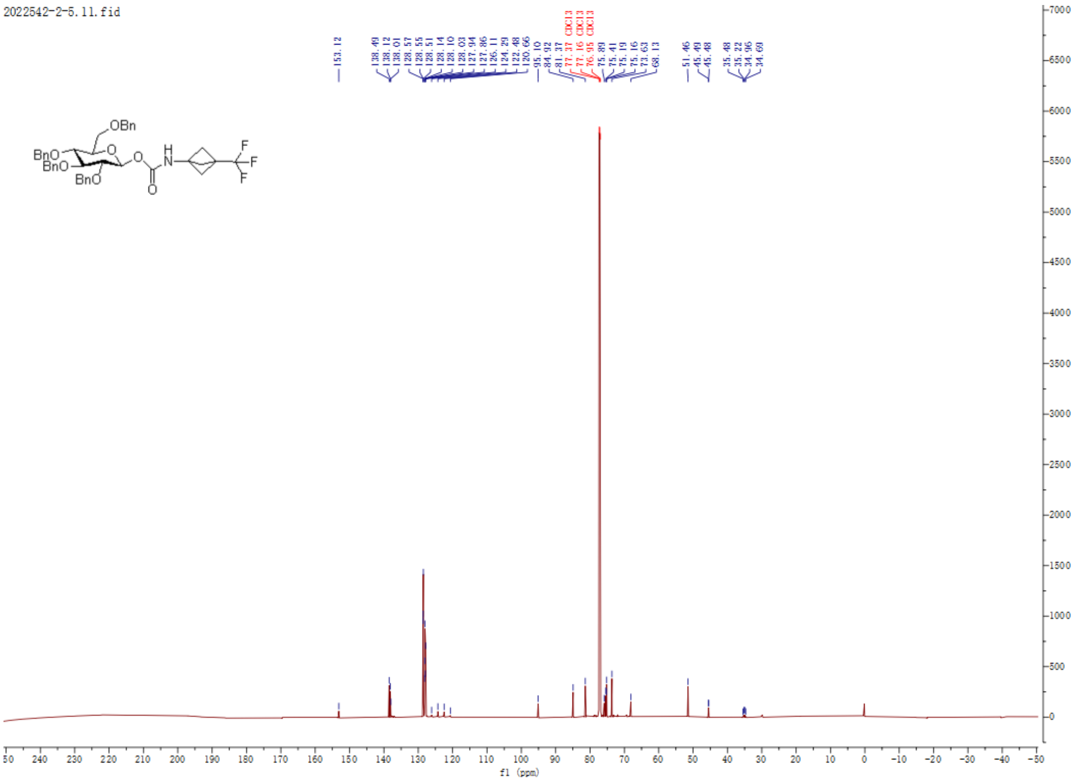

^{13}C NMR spectrum of compound **GA39** (151 MHz, $CDCl_3$)

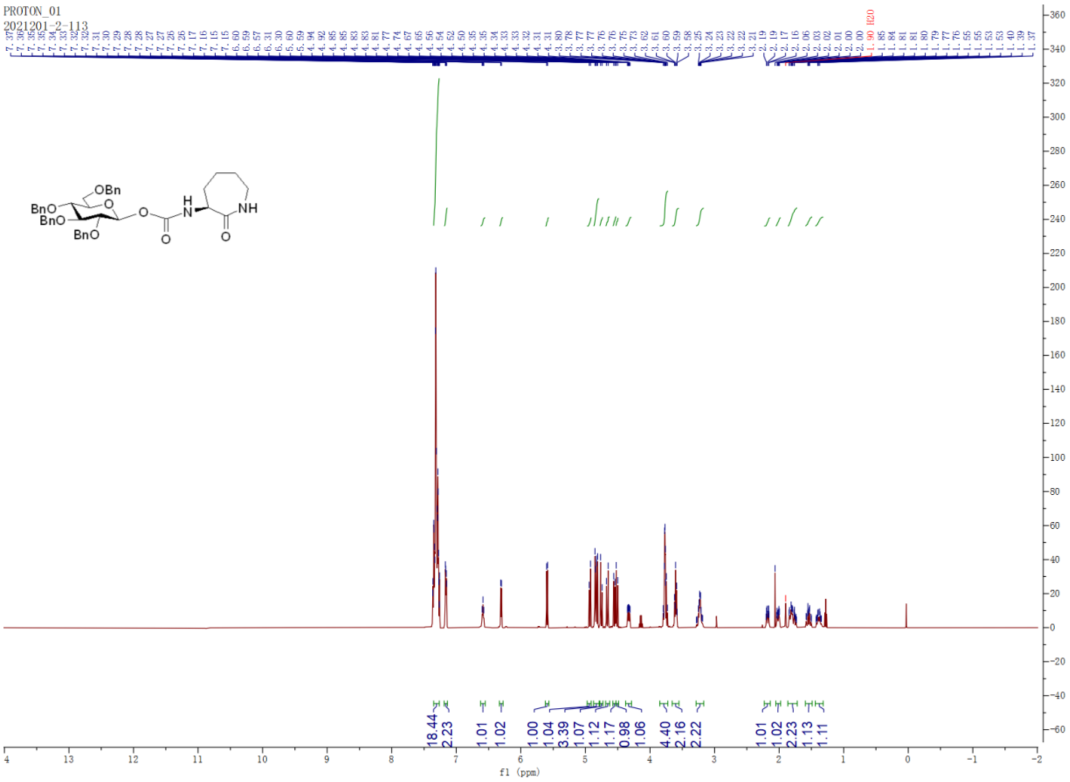

^{1}H NMR spectrum of compound **GA40** (500 MHz, $CDCl_3$)

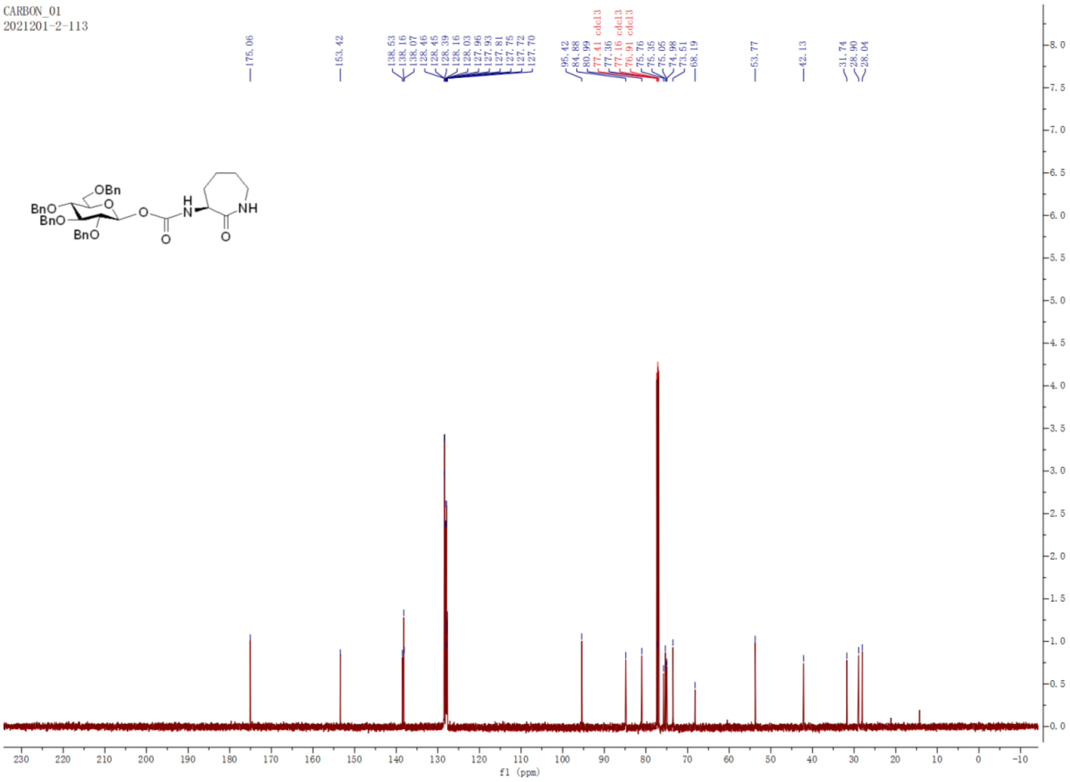

^{13}C NMR spectrum of compound **GA40** (126 MHz, $CDCl_3$)

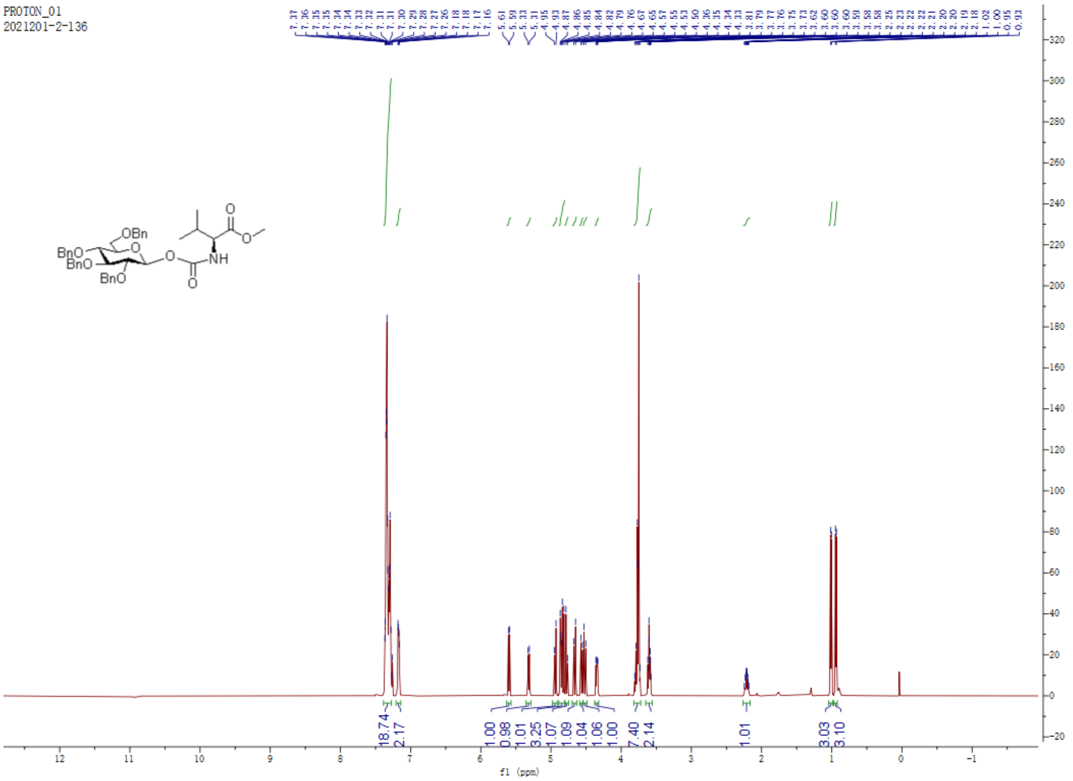

^{1}H NMR spectrum of compound **GA41** (500 MHz, $CDCl_3$)

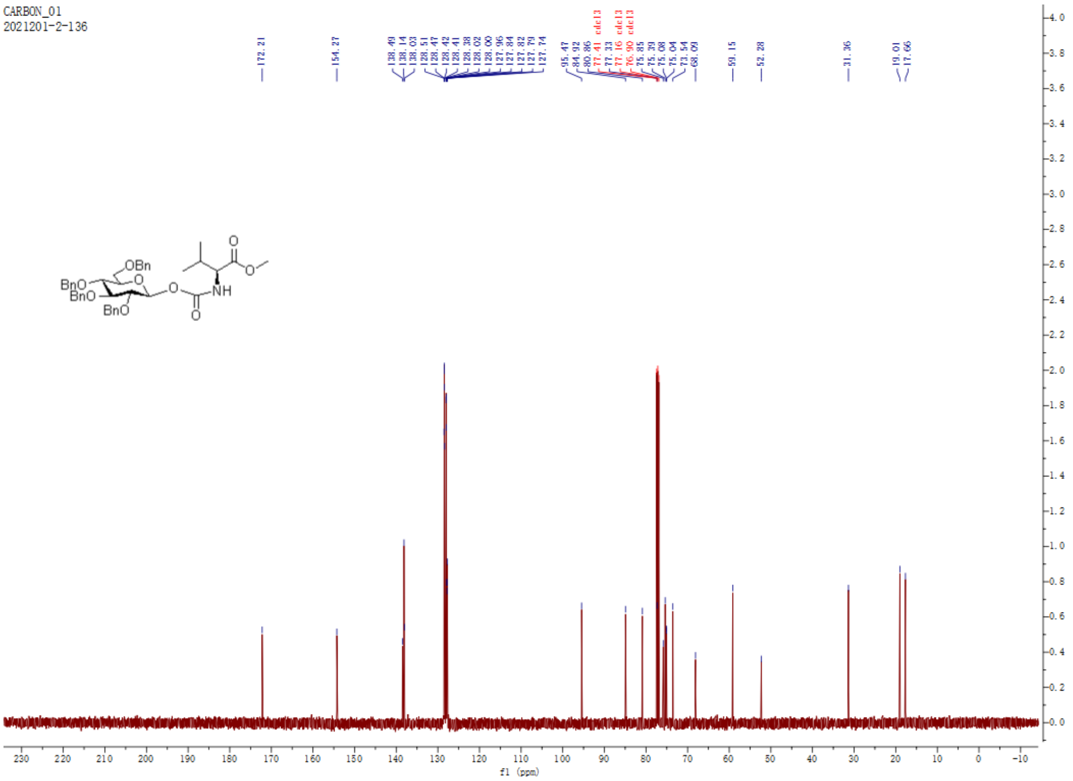

^{13}C NMR spectrum of compound **GA41** (126 MHz, $CDCl_3$)

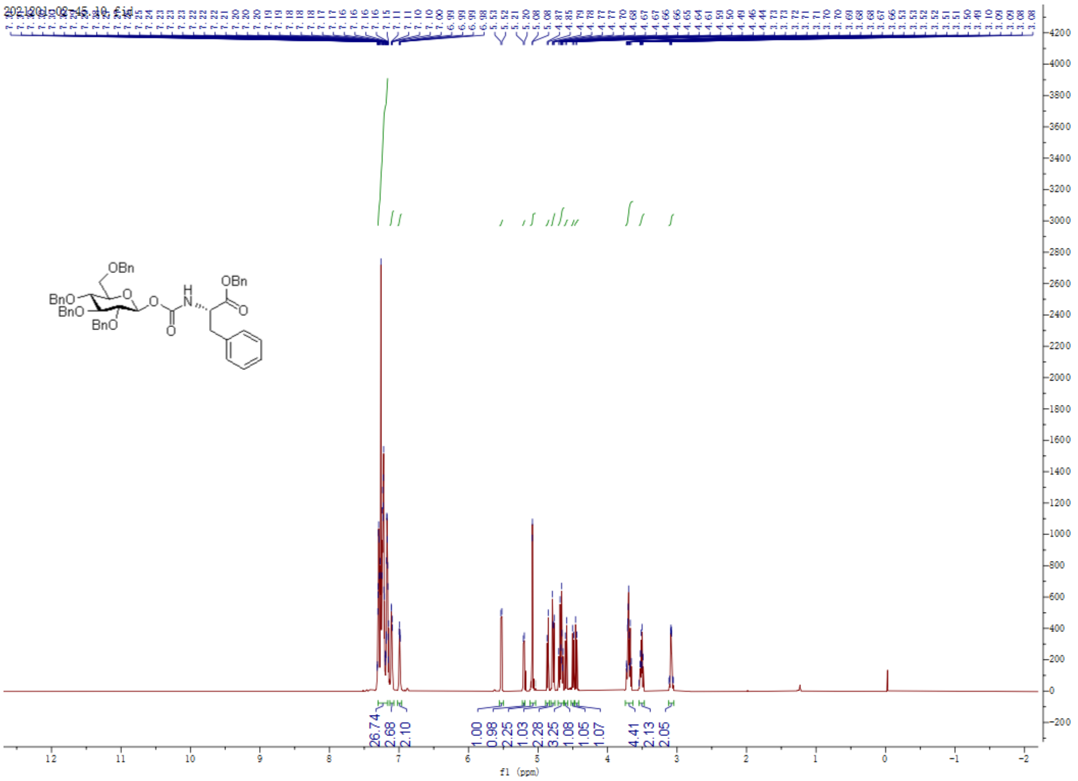

^{1}H NMR spectrum of compound **GA42** (600 MHz, $CDCl_3$)

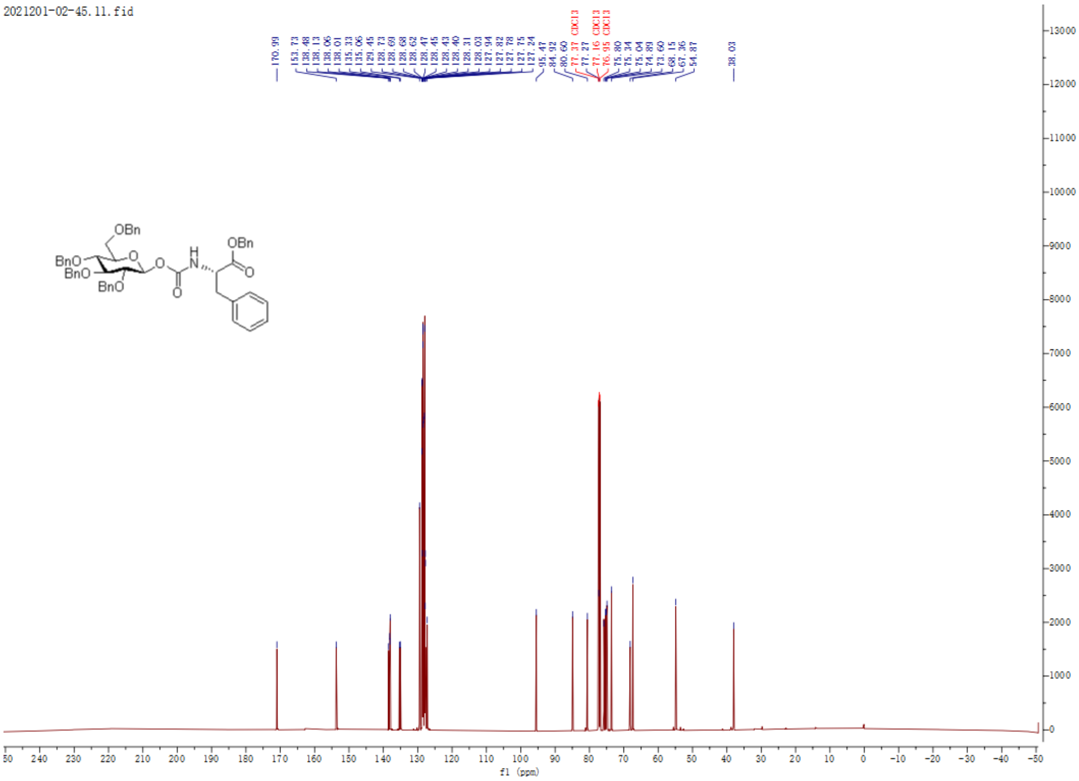

^{13}C NMR spectrum of compound **GA42** (151 MHz, $CDCl_3$)

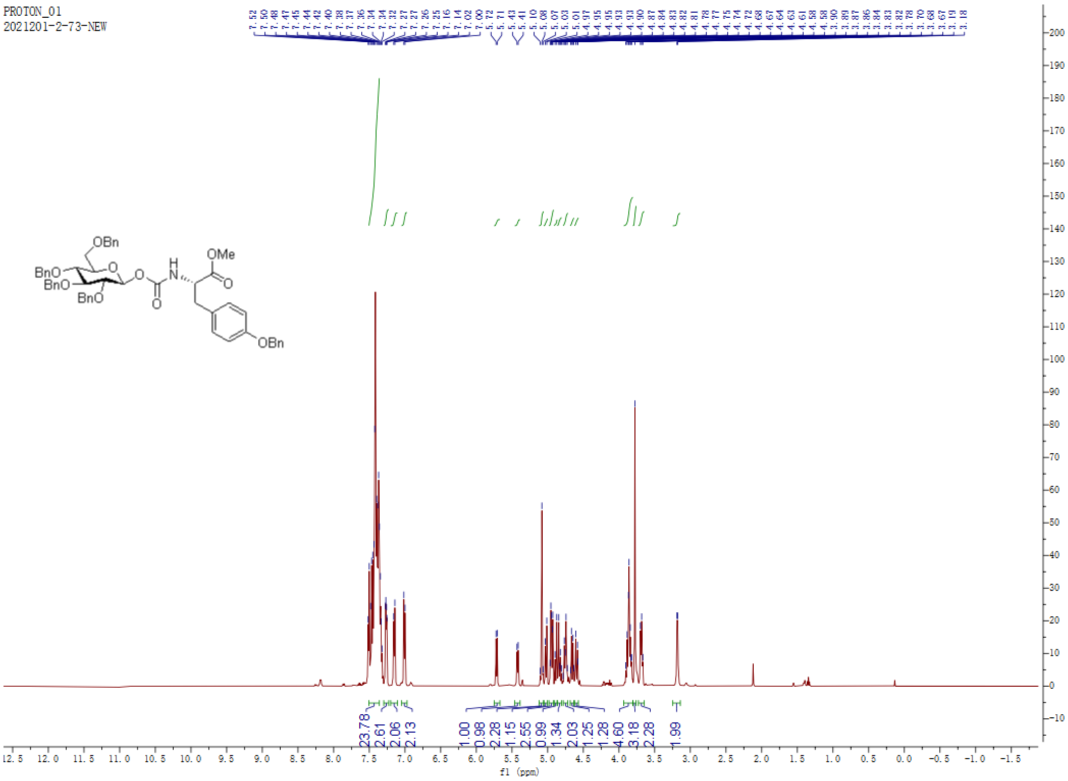

^{1}H NMR spectrum of compound **GA43** (500 MHz, $CDCl_3$)

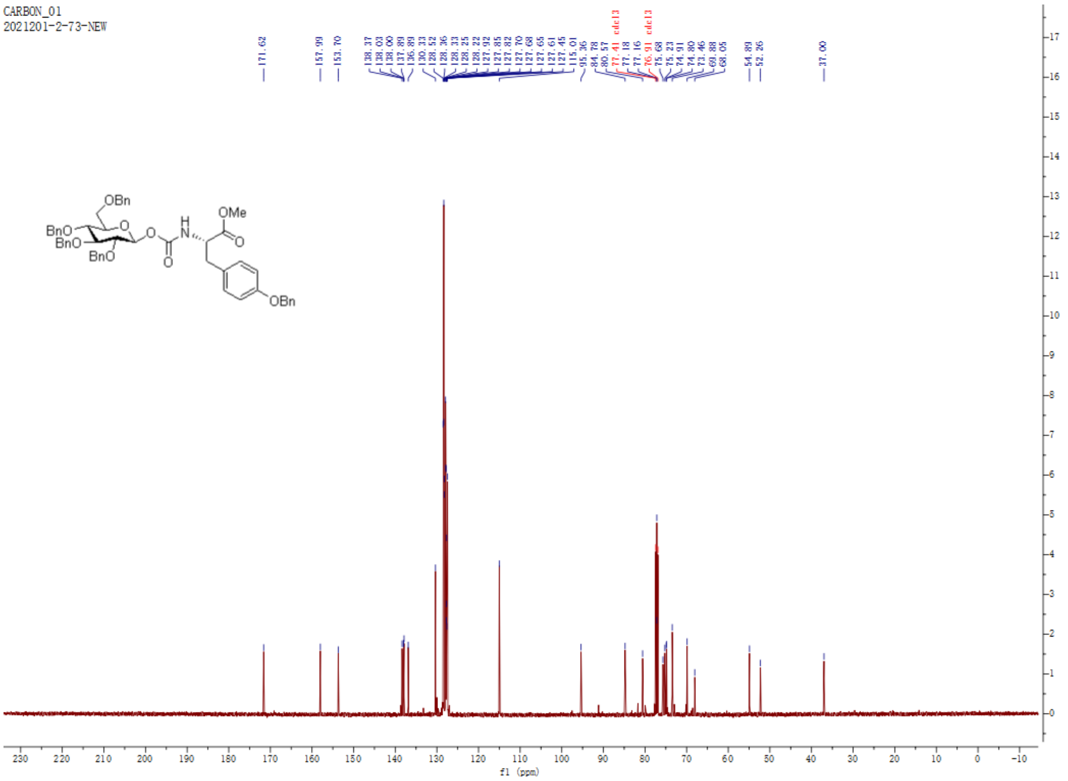

^{13}C NMR spectrum of compound **GA43** (126 MHz, $CDCl_3$)

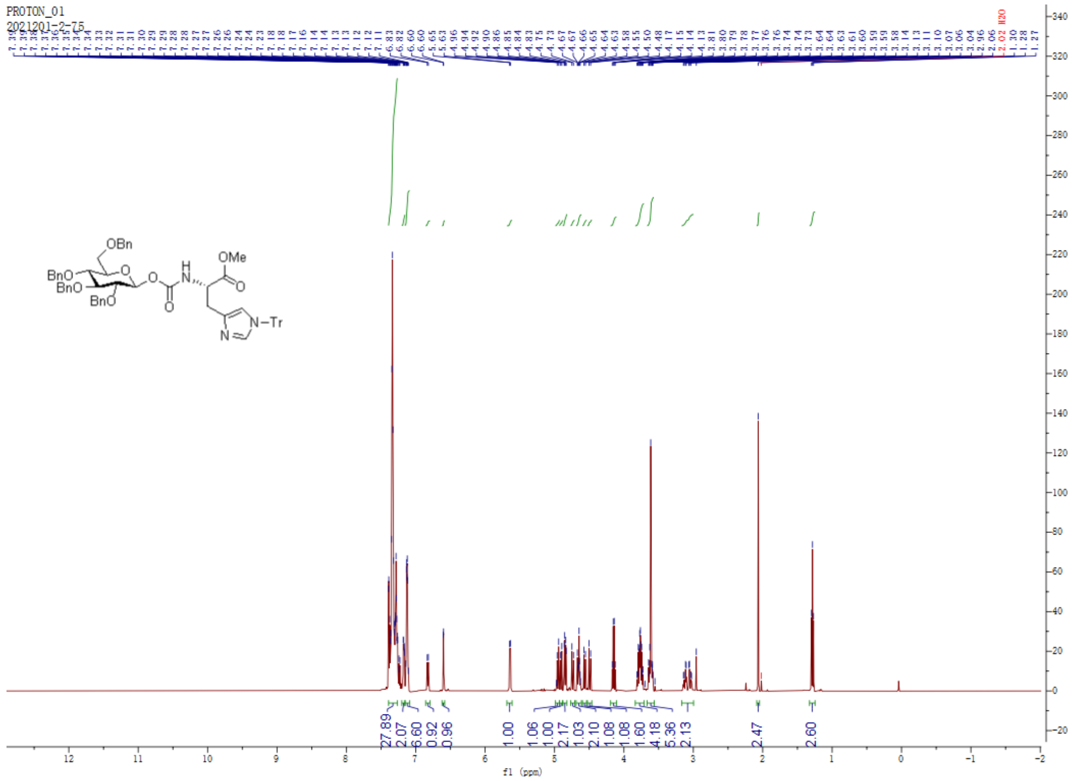

^{1}H NMR spectrum of compound **GA44** (500 MHz, $CDCl_3$)

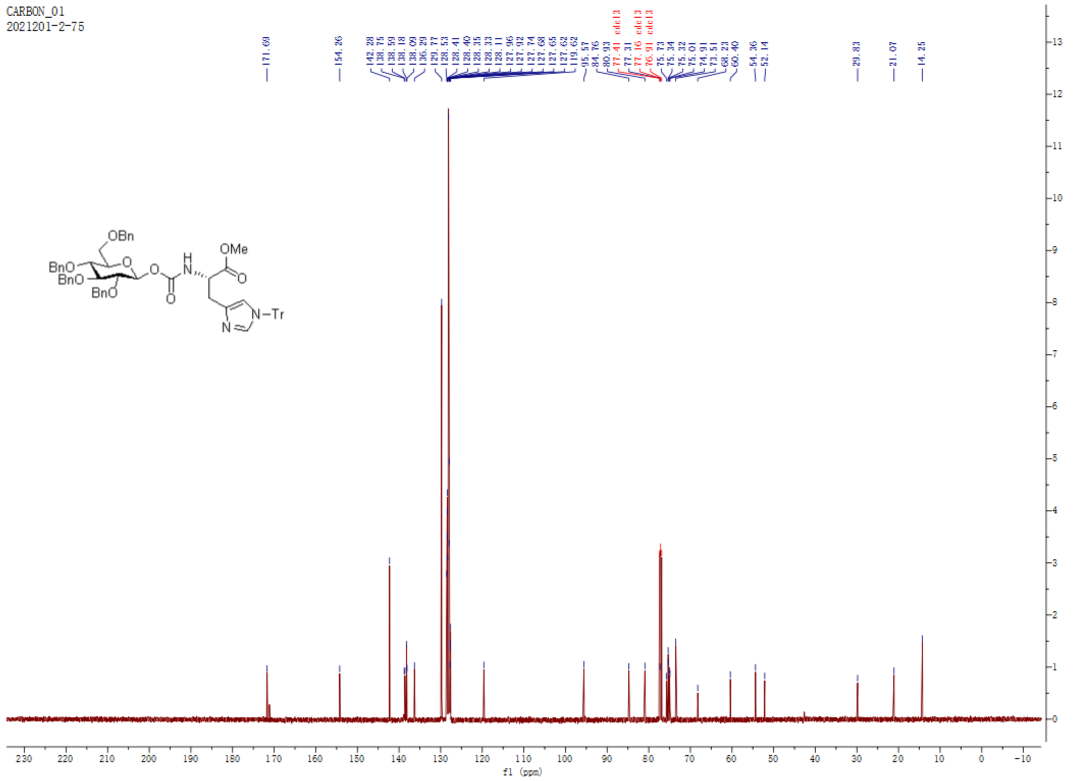

^{13}C NMR spectrum of compound **GA44** (126 MHz, $CDCl_3$)

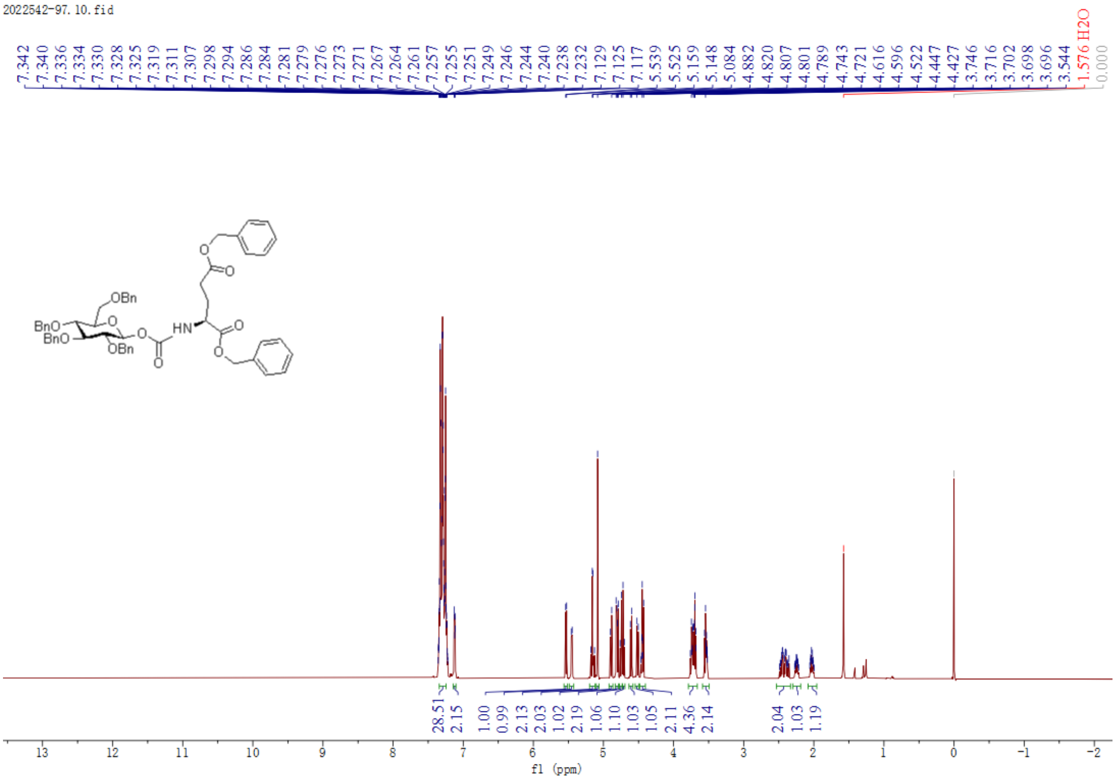

^{1}H NMR spectrum of compound **GA45** (600 MHz, $CDCl_3$)

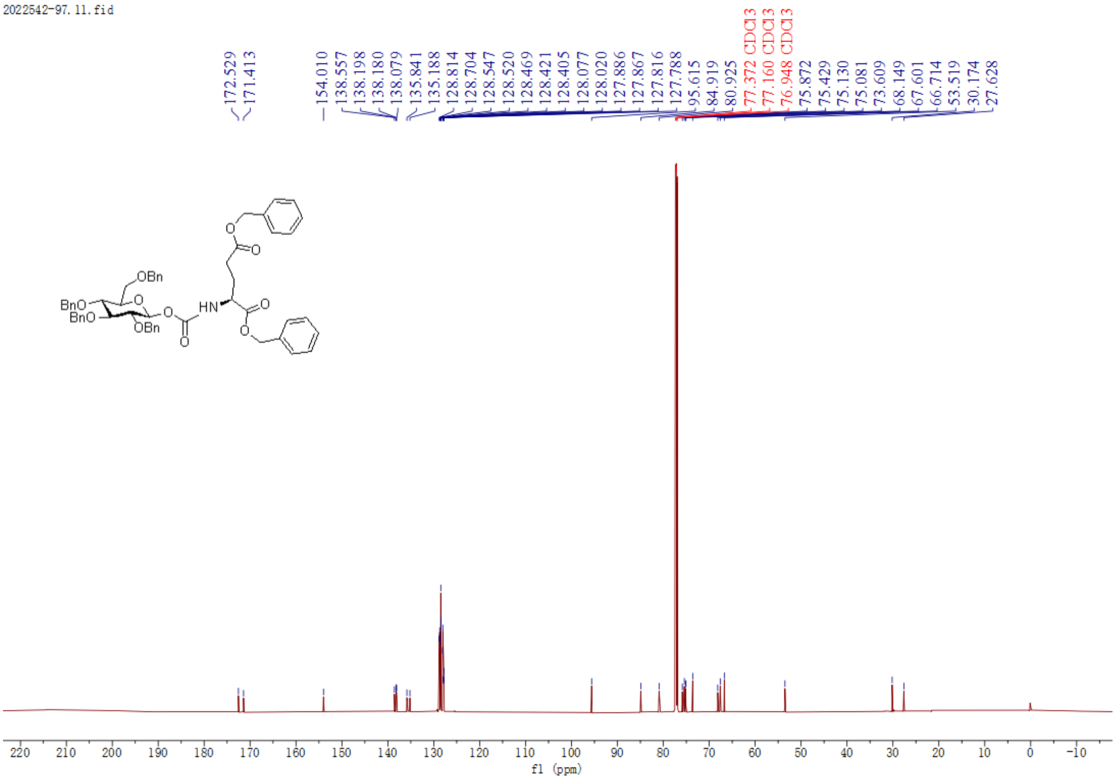

^{13}C NMR spectrum of compound **GA45** (151 MHz, $CDCl_3$)

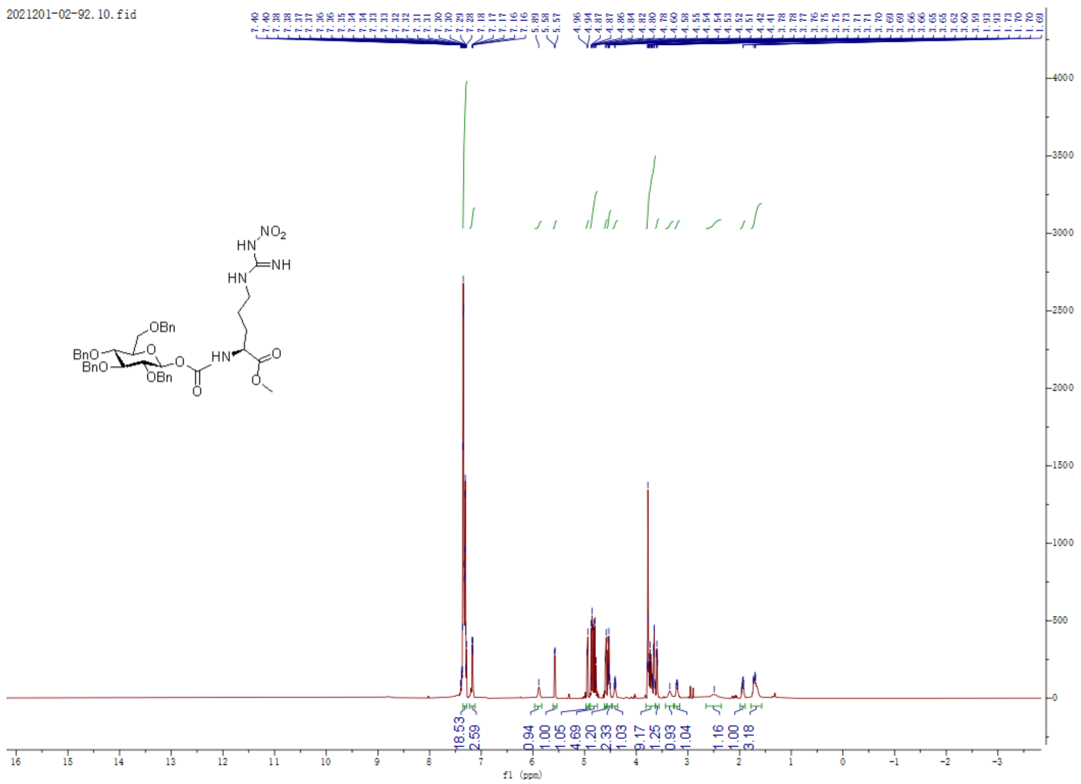

^{1}H NMR spectrum of compound **GA46** (600 MHz, $CDCl_3$)

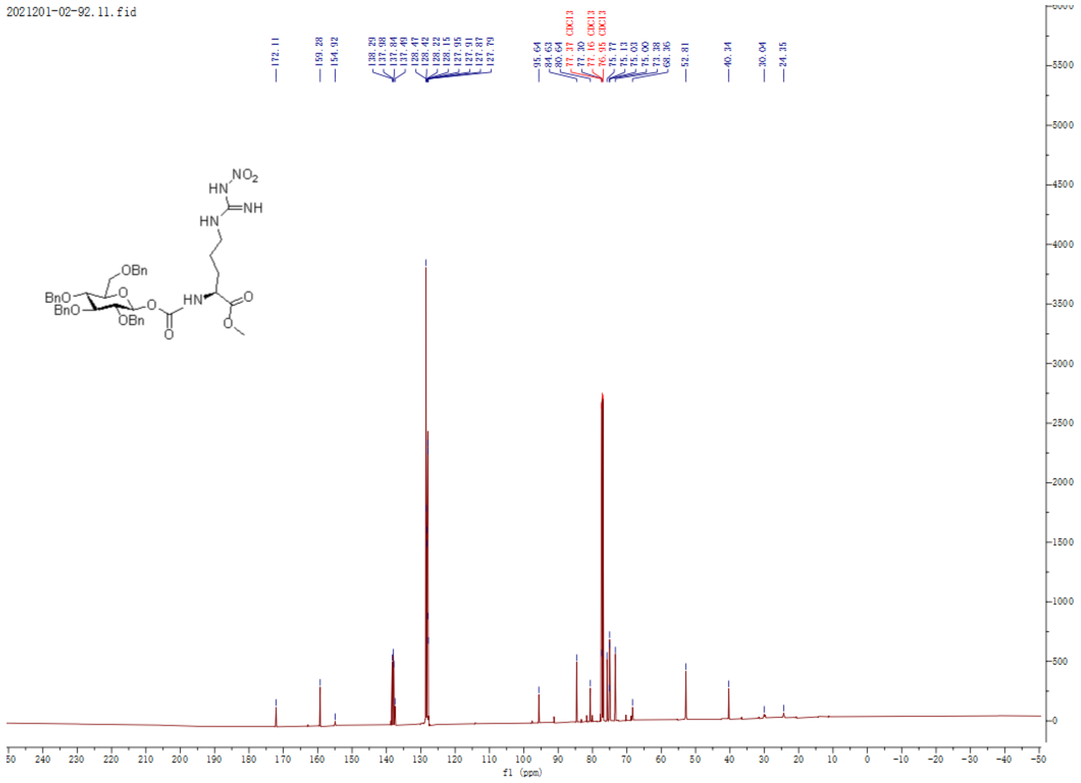

^{13}C NMR spectrum of compound **GA46** (151 MHz, $CDCl_3$)

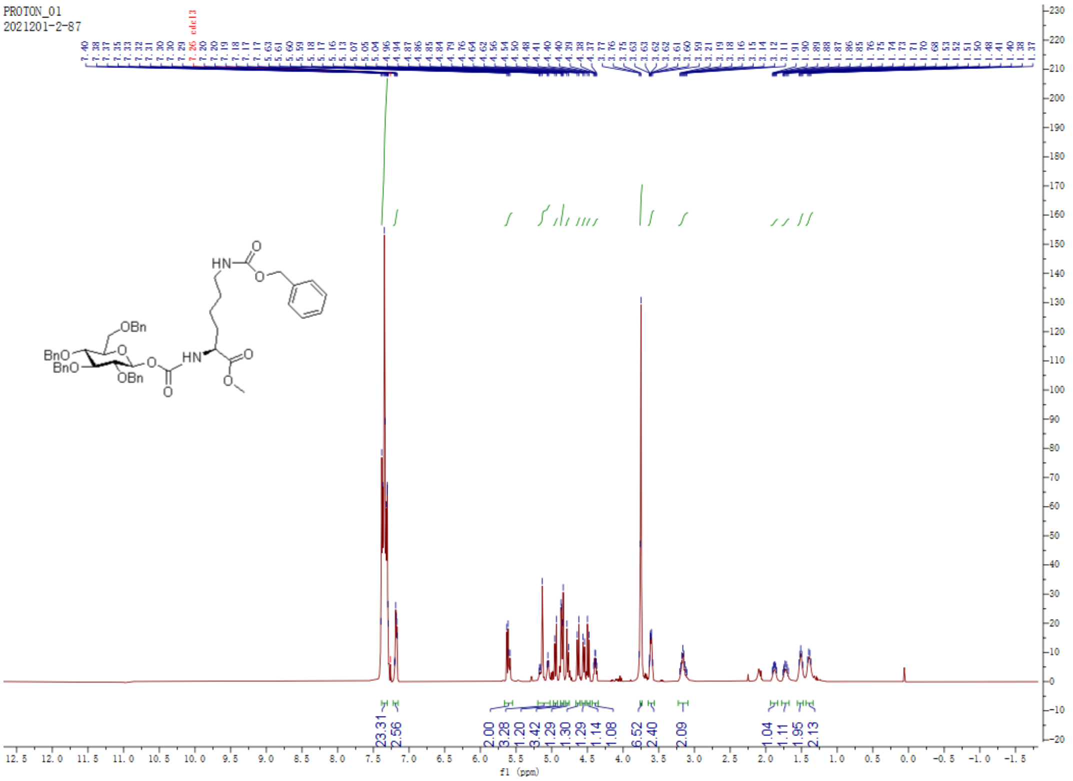

^{1}H NMR spectrum of compound **GA47** (500 MHz, $CDCl_3$)

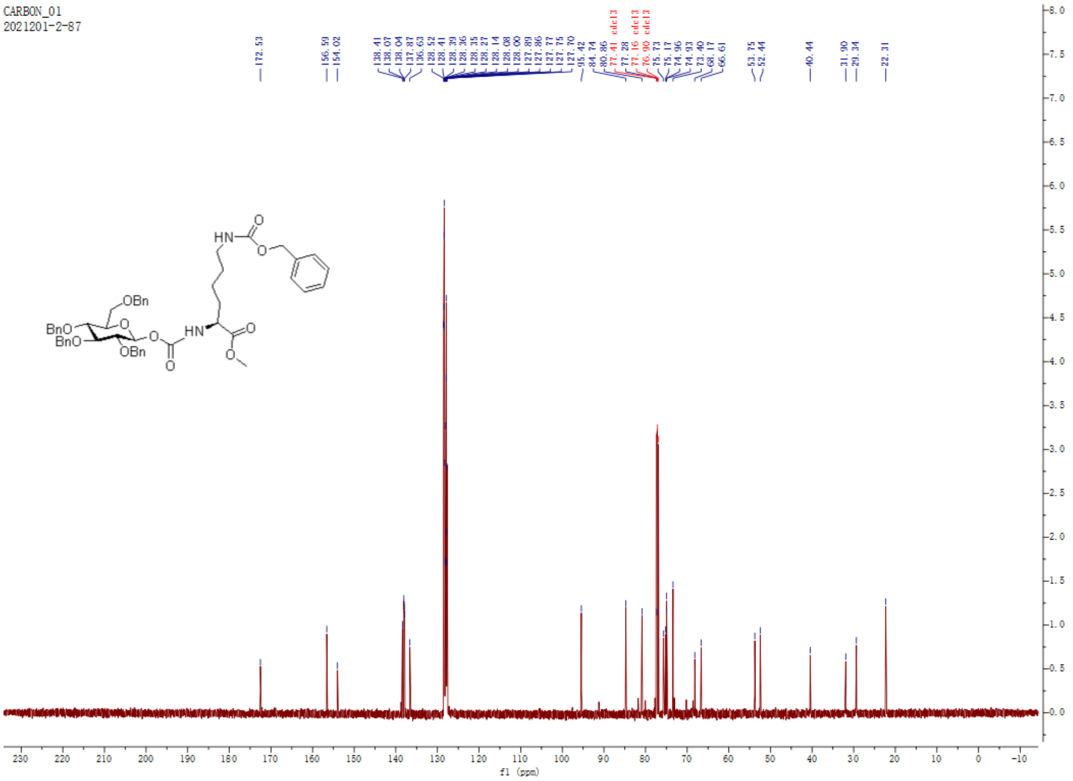

^{13}C NMR spectrum of compound **GA47** (126 MHz, $CDCl_3$)

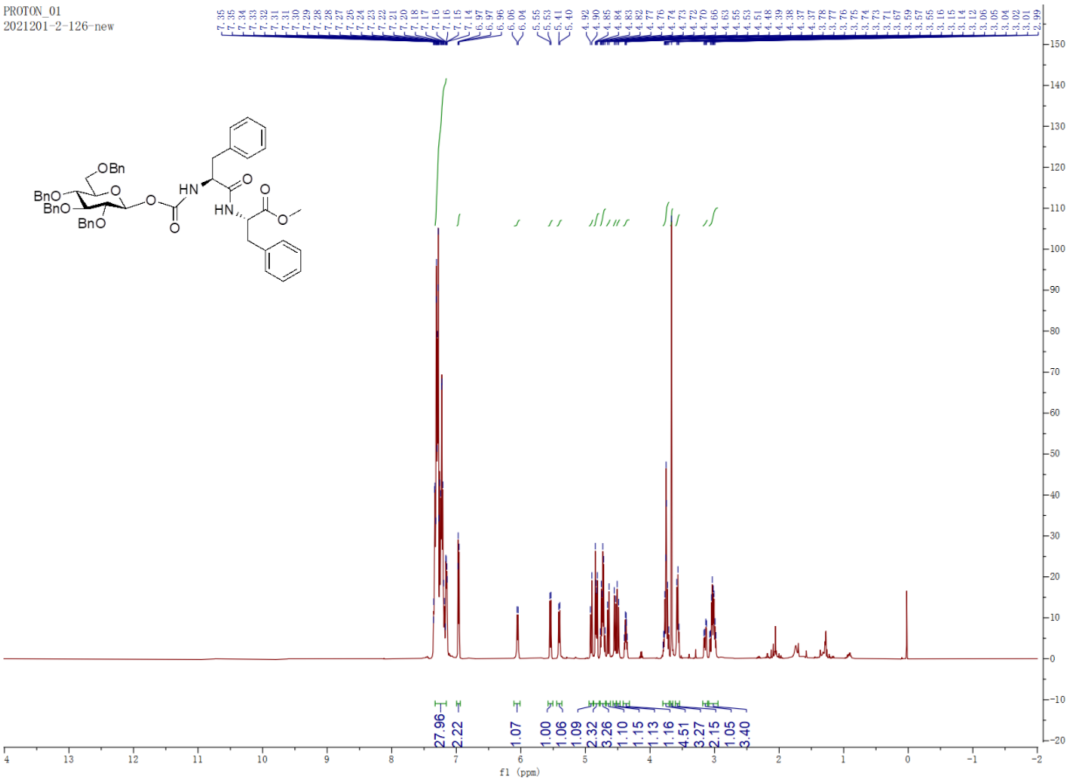

^{1}H NMR spectrum of compound **GA48** (500 MHz, $CDCl_3$)

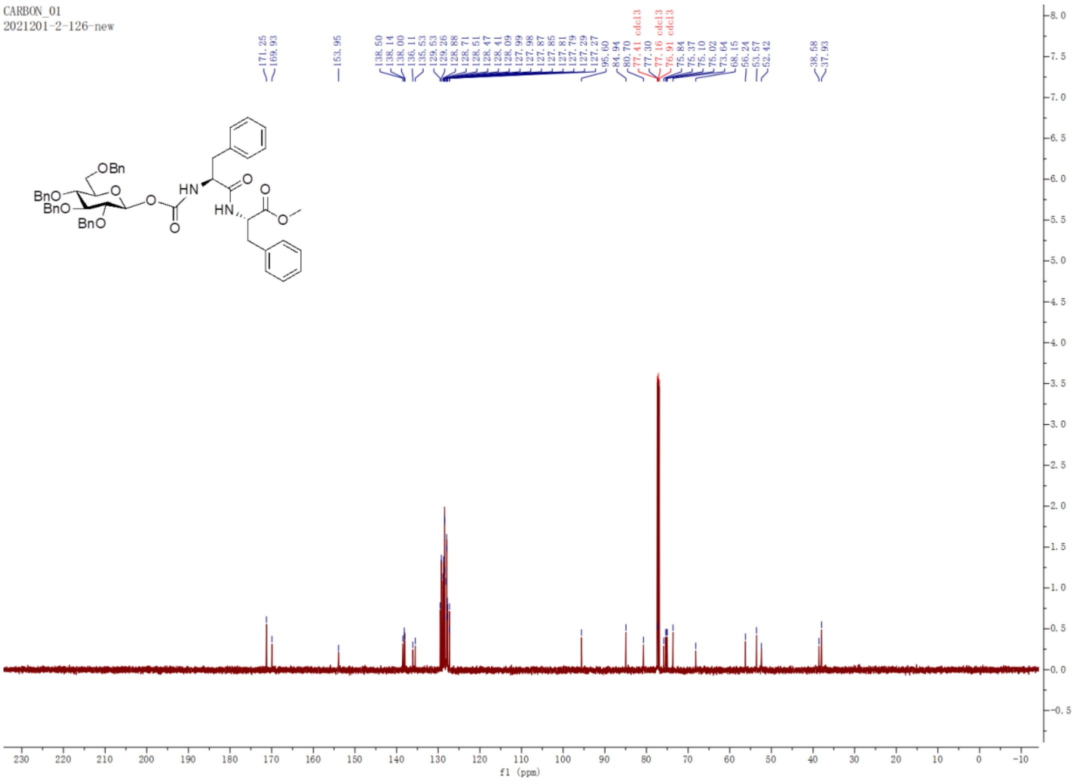

^{13}C NMR spectrum of compound **GA48** (126 MHz, $CDCl_3$)

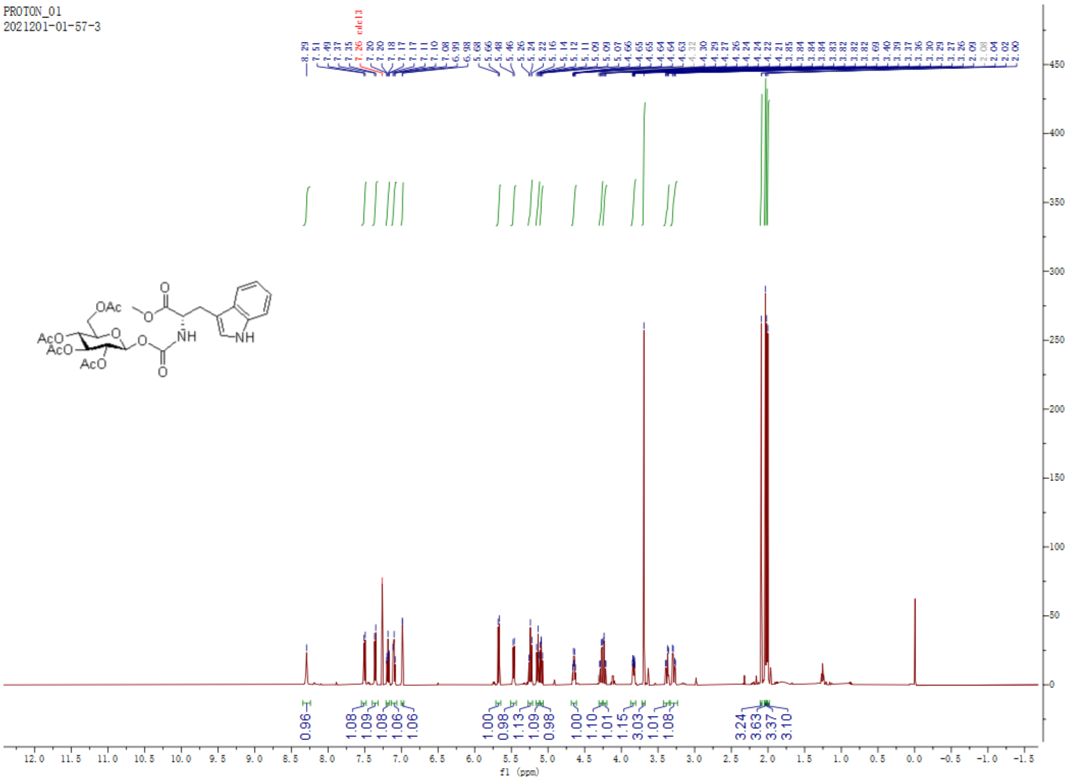

^{1}H NMR spectrum of compound **GA49** (500 MHz, $CDCl_3$)

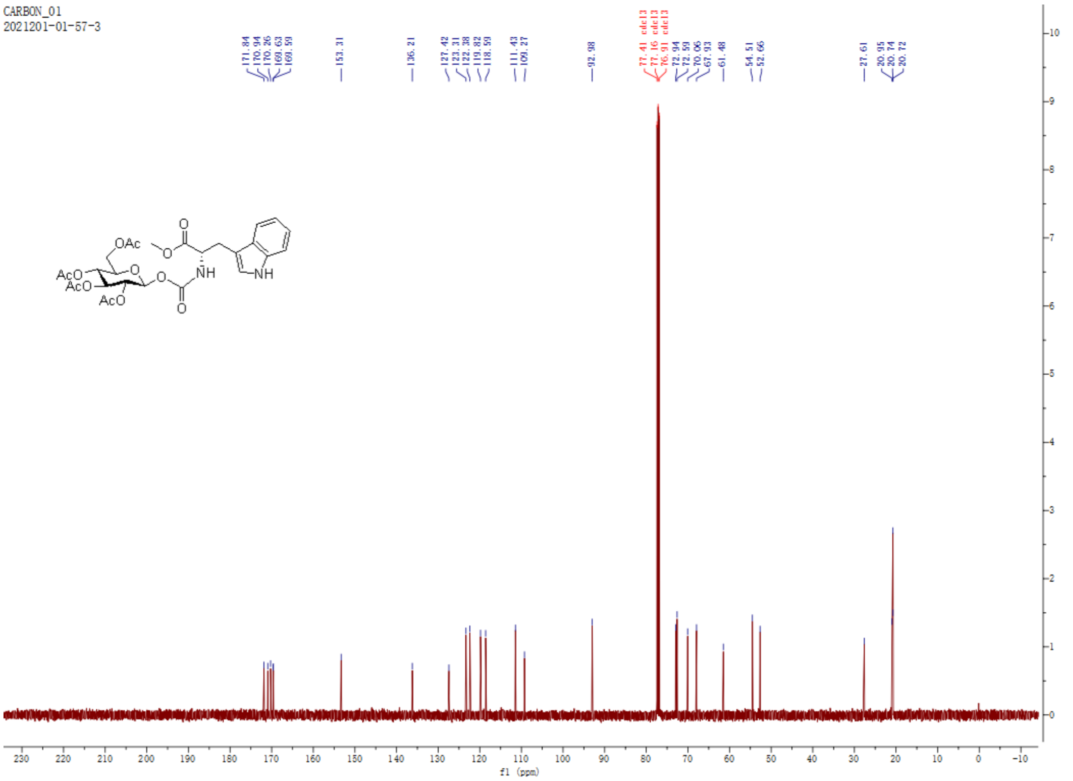

^{13}C NMR spectrum of compound **GA49** (126 MHz, $CDCl_3$)

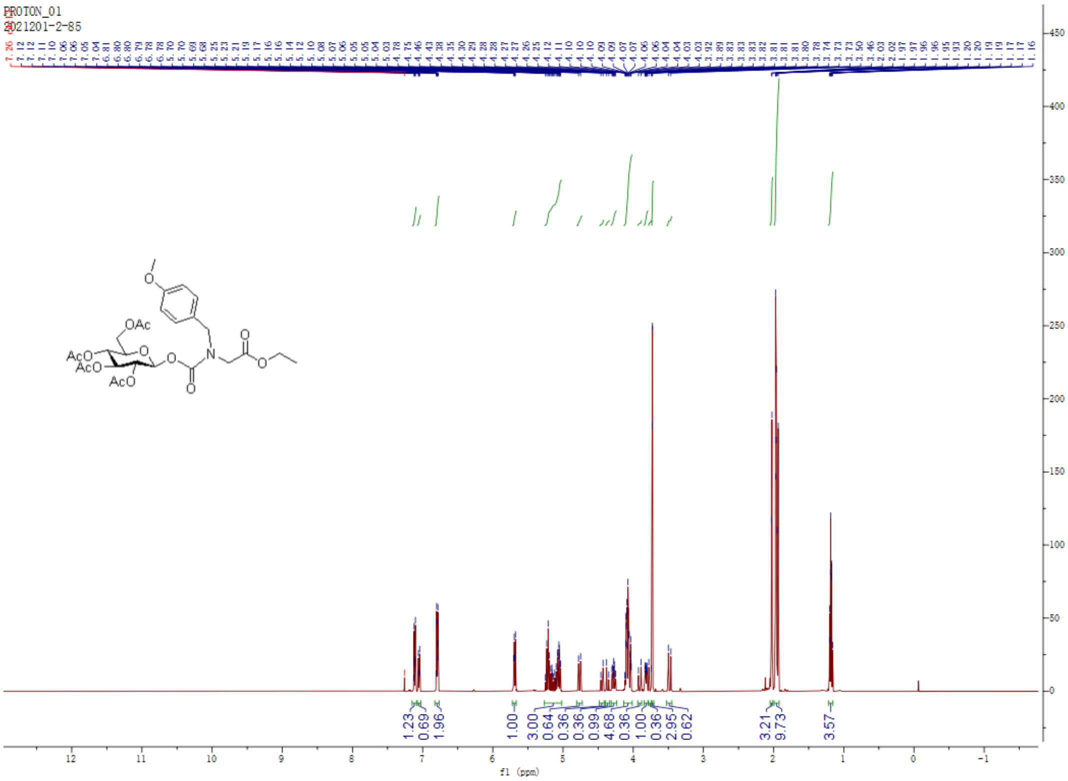

^{1}H NMR spectrum of compound **GA50** (500 MHz, $CDCl_3$)

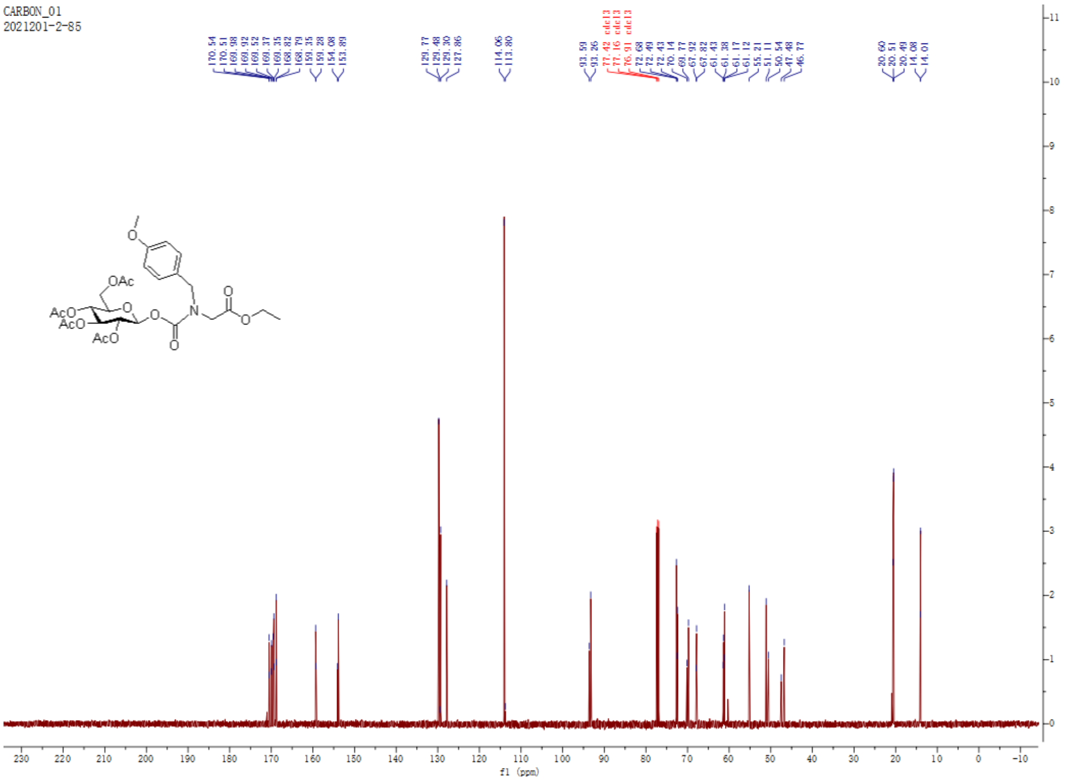

^{13}C NMR spectrum of compound **GA50** (126 MHz, $CDCl_3$)

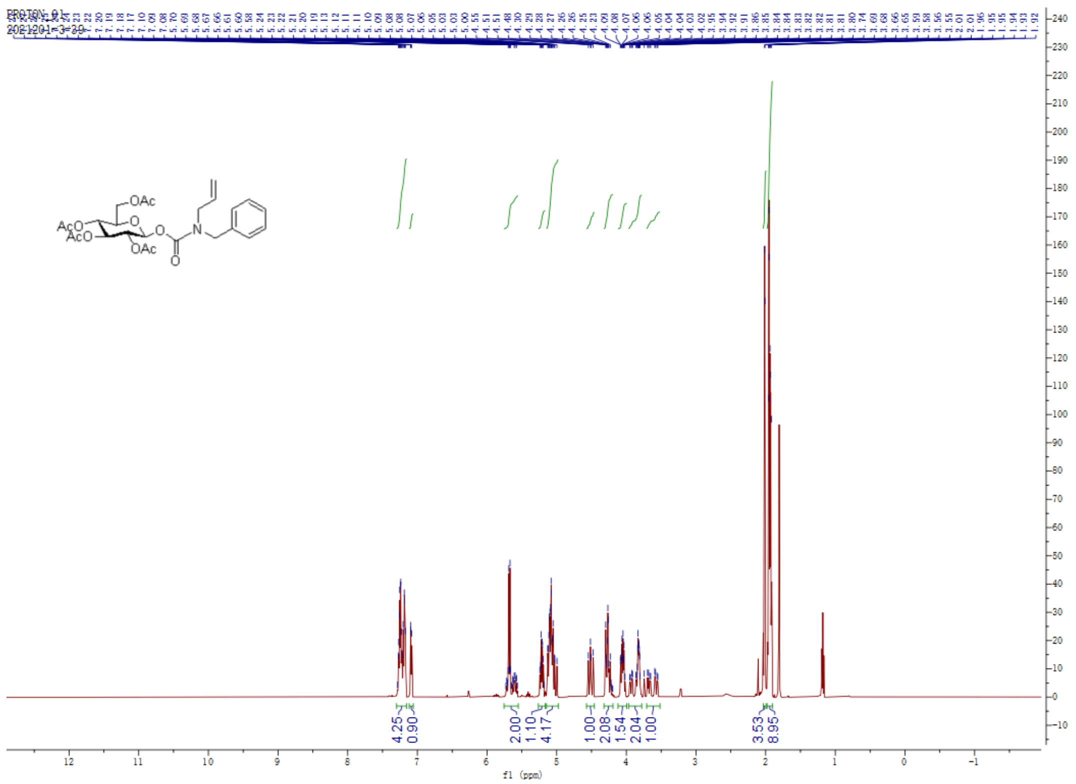

^{1}H NMR spectrum of compound **GA51** (500 MHz, $CDCl_3$)

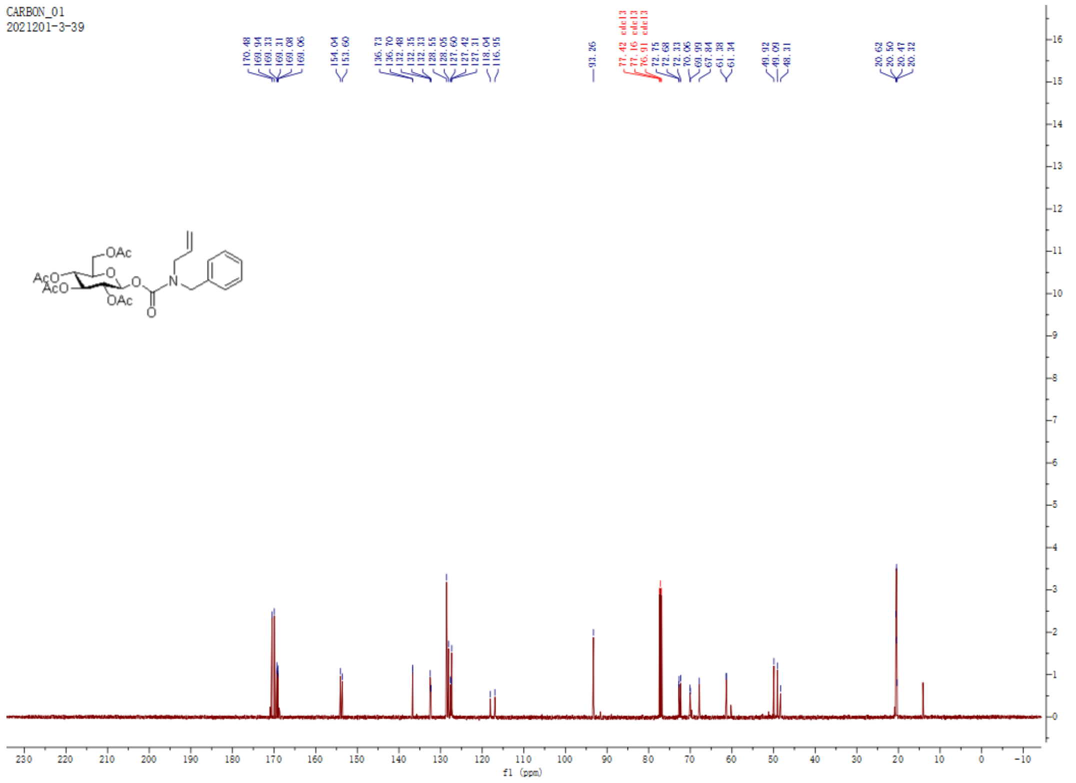

^{13}C NMR spectrum of compound **GA51** (126 MHz, $CDCl_3$)

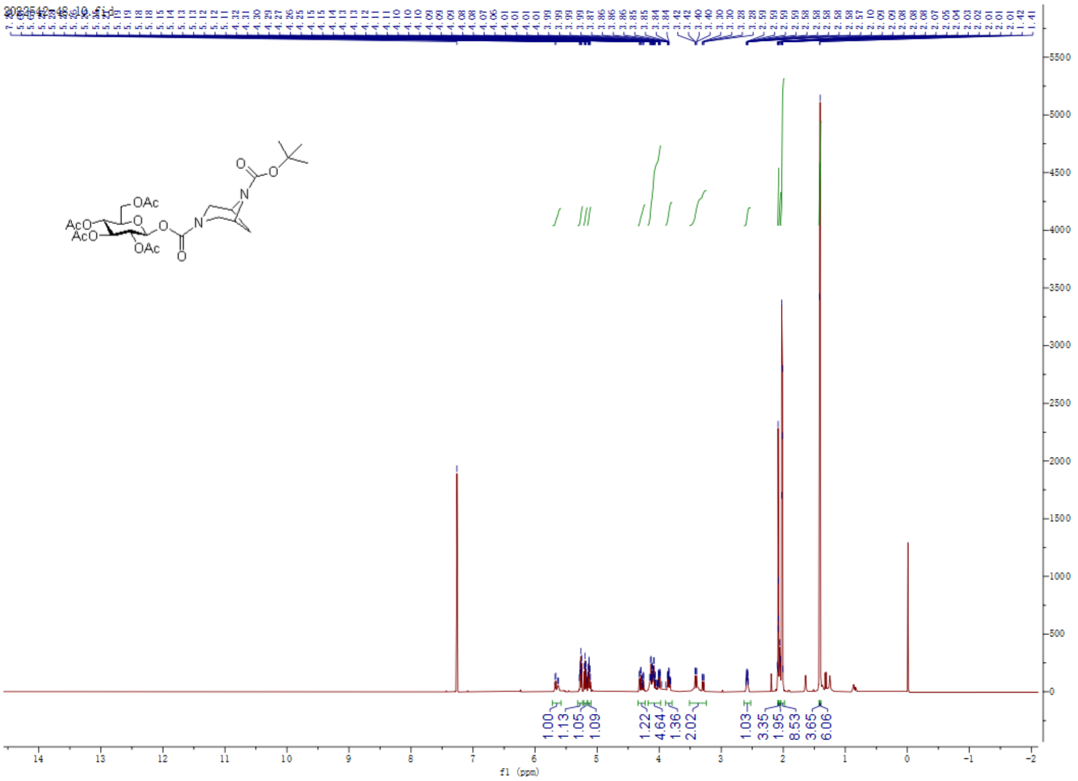

^{1}H NMR spectrum of compound **GA52** (600 MHz, $CDCl_3$)

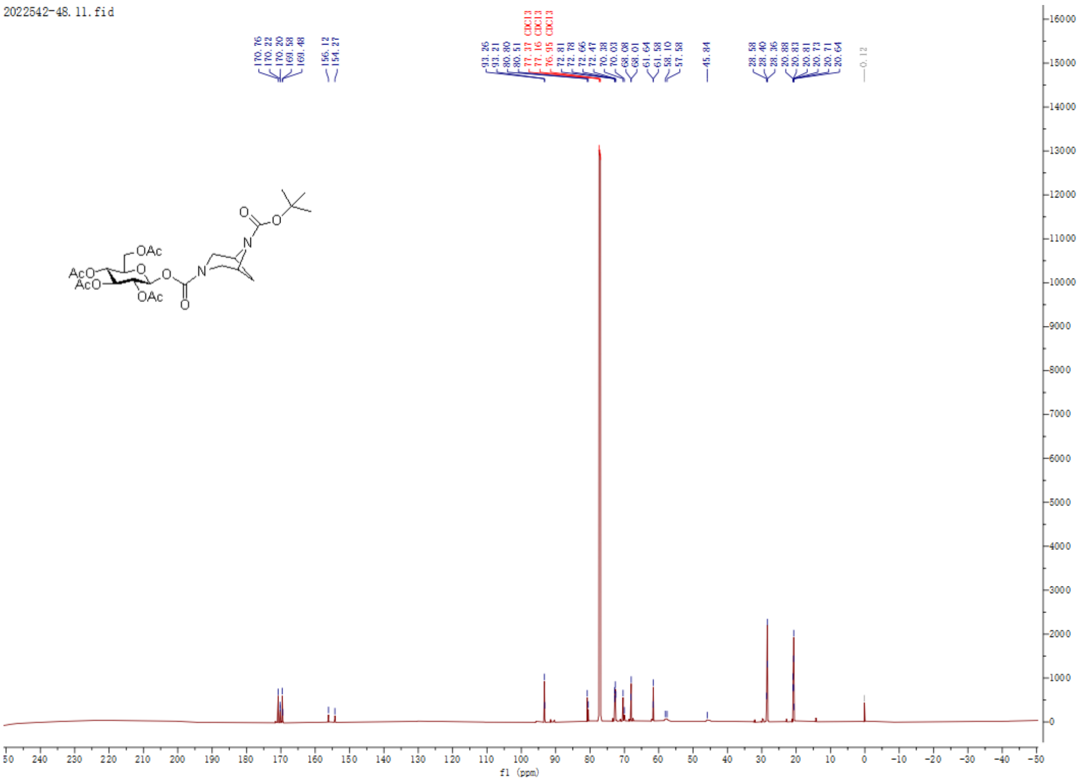

^{13}C NMR spectrum of compound **GA52** (151 MHz, $CDCl_3$)

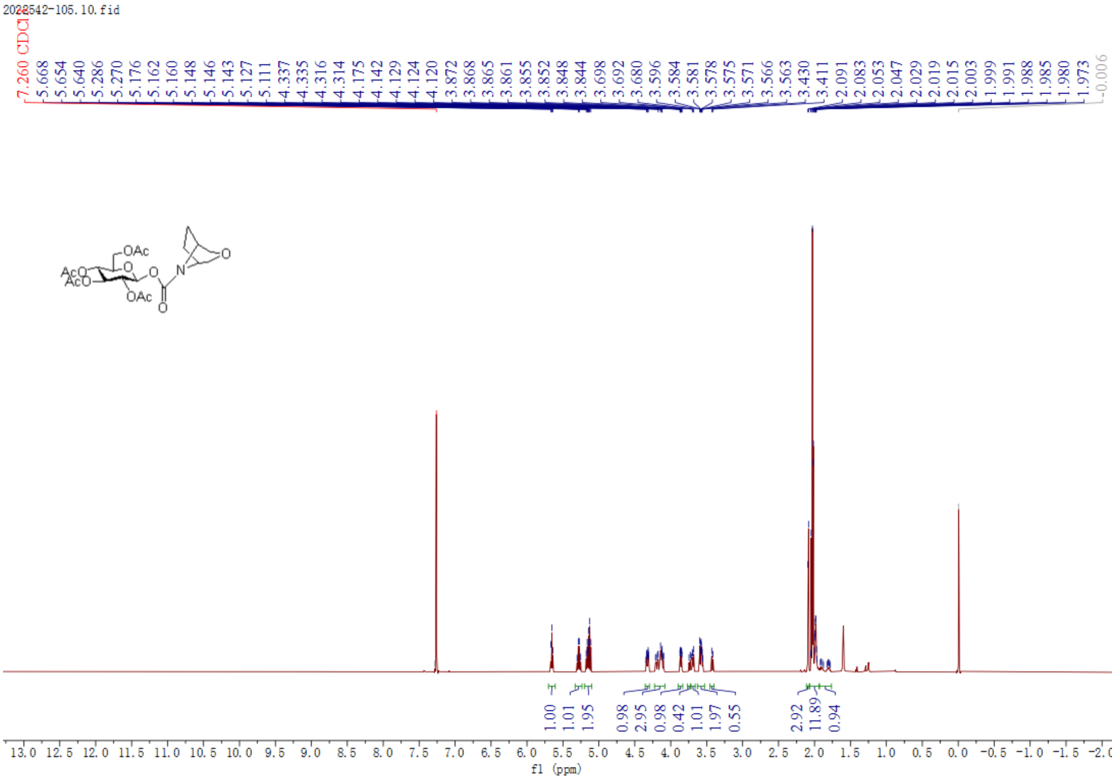

^{1}H NMR spectrum of compound **GA53** (600 MHz, $CDCl_3$)

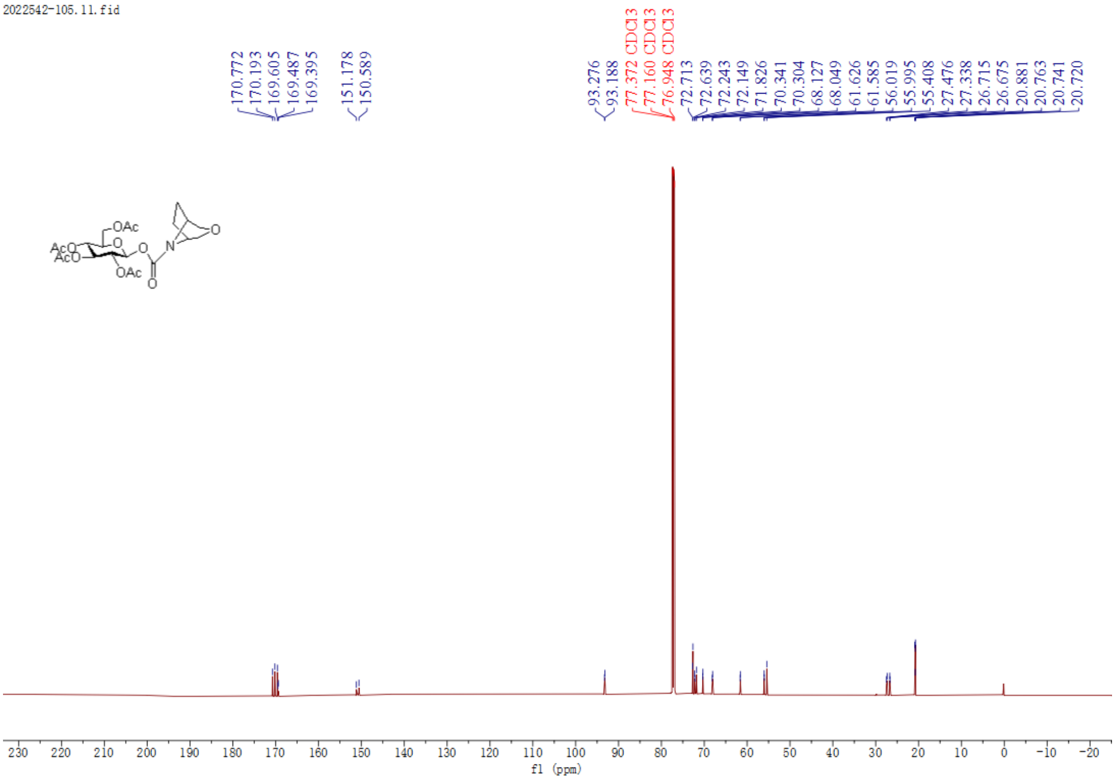

^{13}C NMR spectrum of compound **GA53** (151 MHz, $CDCl_3$)

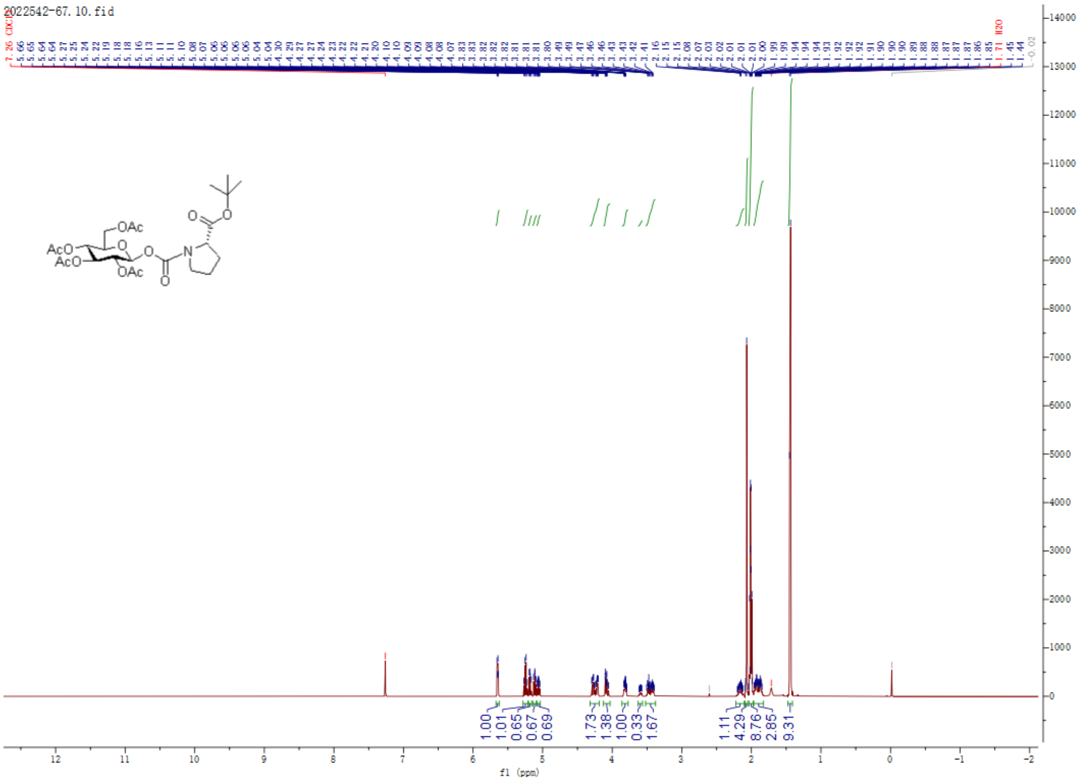

^{1}H NMR spectrum of compound **GA54** (600 MHz, $CDCl_3$)

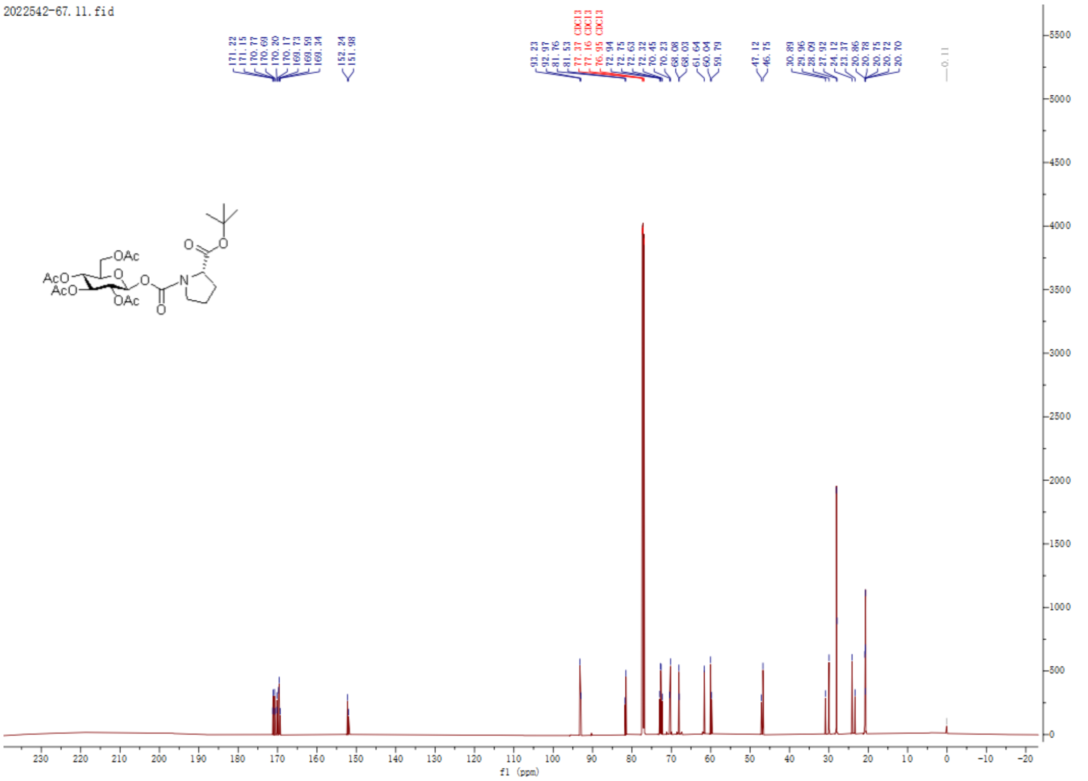

^{13}C NMR spectrum of compound **GA54** (151 MHz, $CDCl_3$)

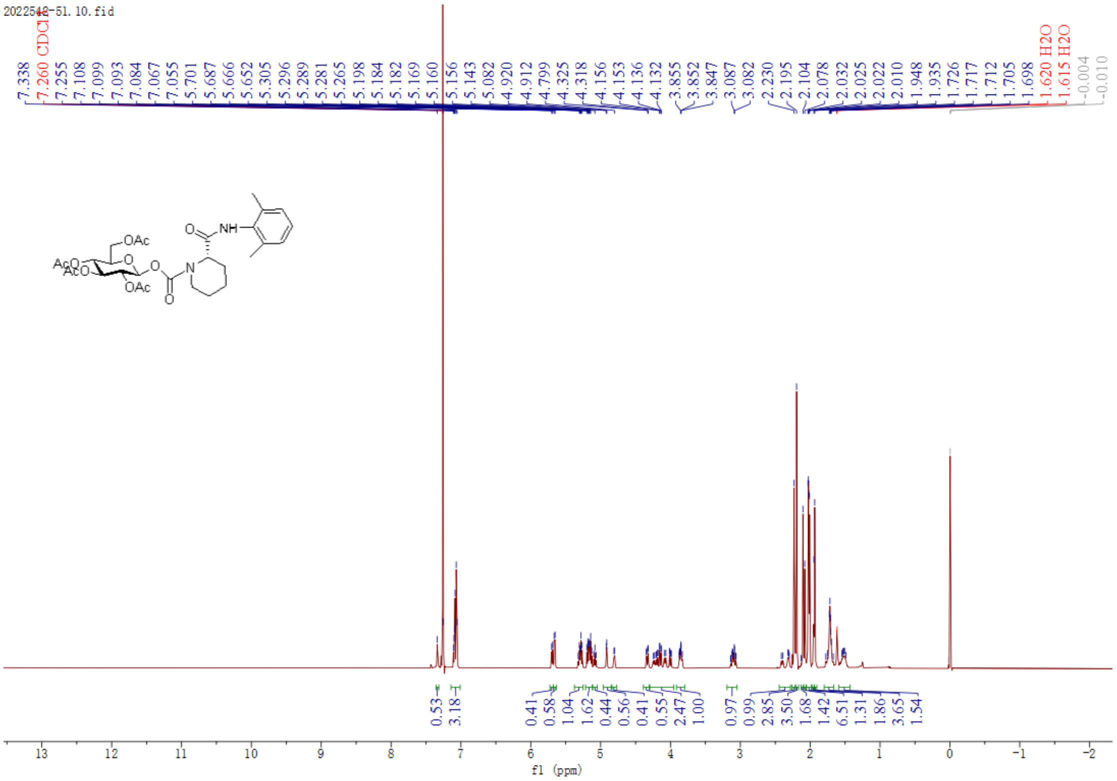

^{1}H NMR spectrum of compound **GA55** (600 MHz, $CDCl_3$)

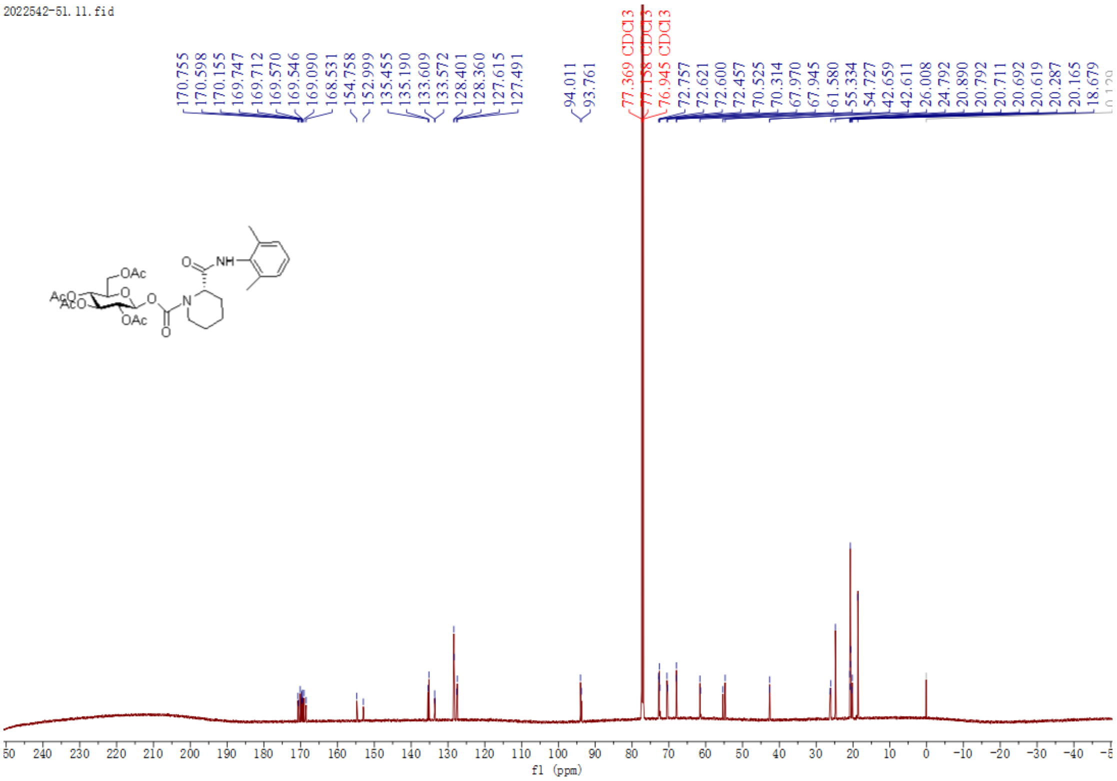

^{13}C NMR spectrum of compound **GA55** (151 MHz, $CDCl_3$)

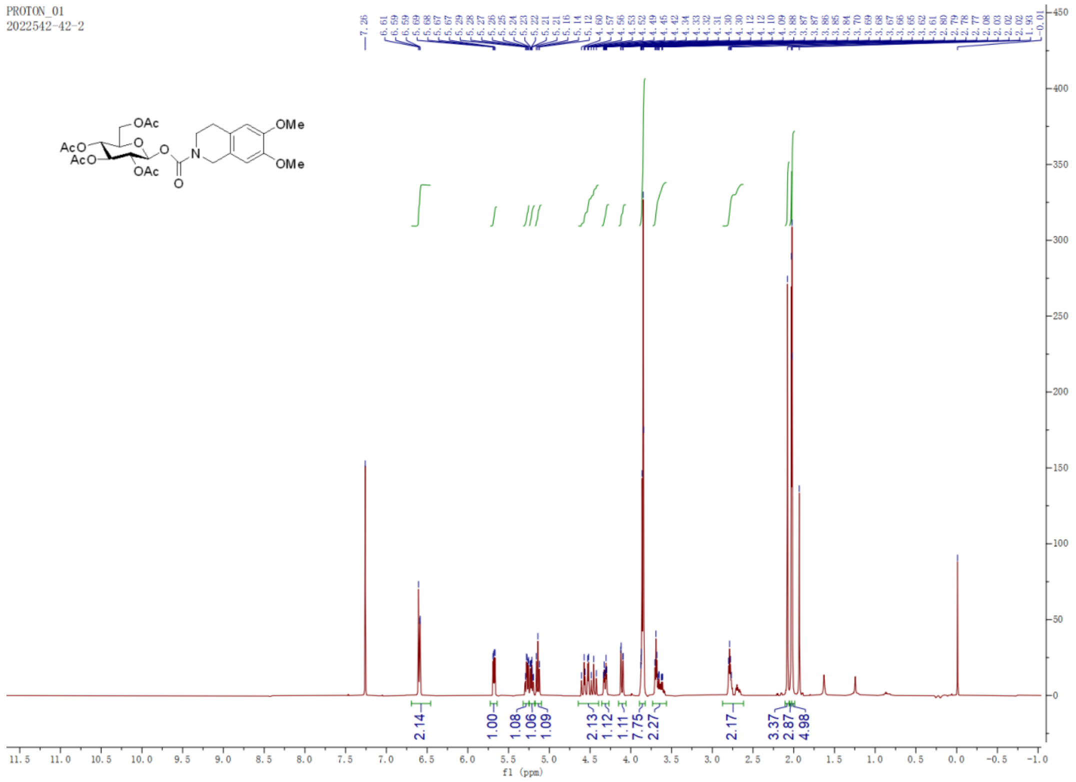

^{1}H NMR spectrum of compound **GA56** (500 MHz, $CDCl_3$)

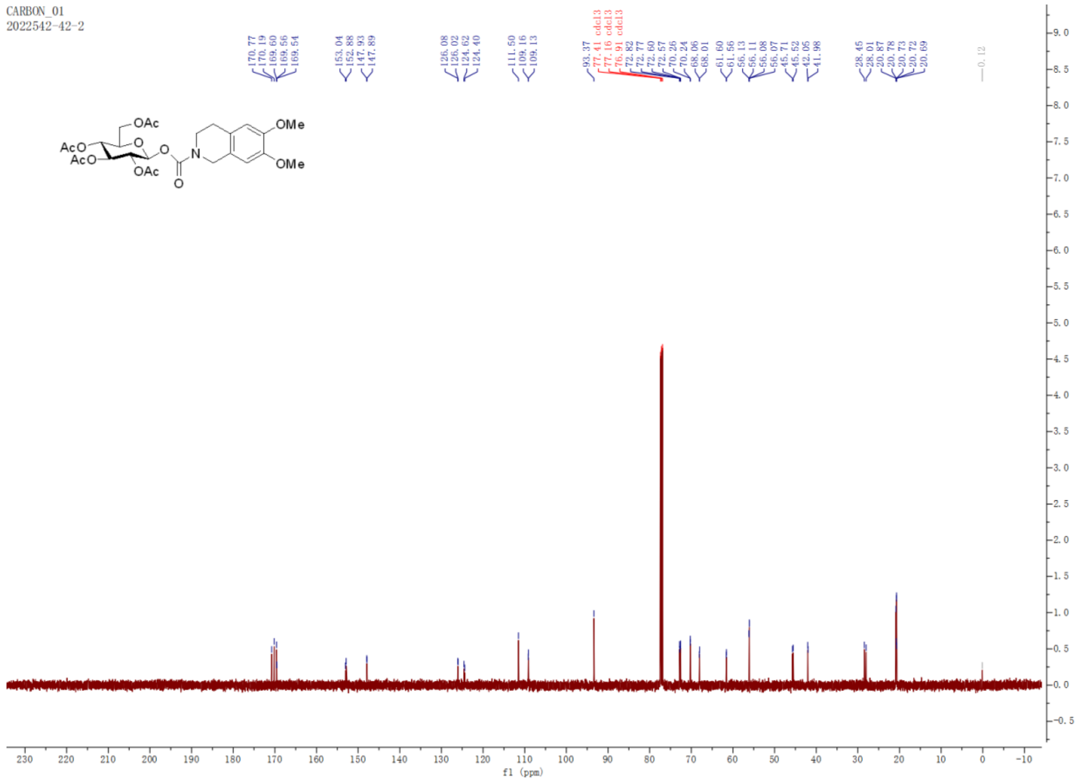

^{13}C NMR spectrum of compound **GA56** (126 MHz, $CDCl_3$)

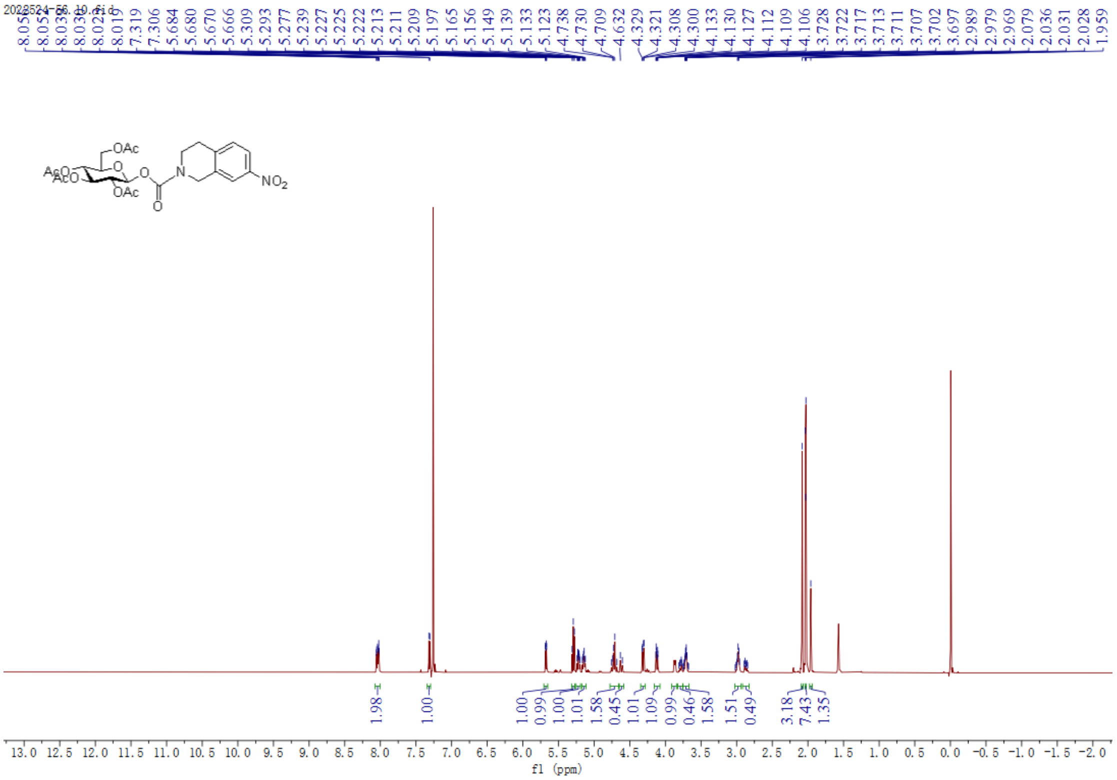

^{1}H NMR spectrum of compound **GA57** (600 MHz, $CDCl_3$)

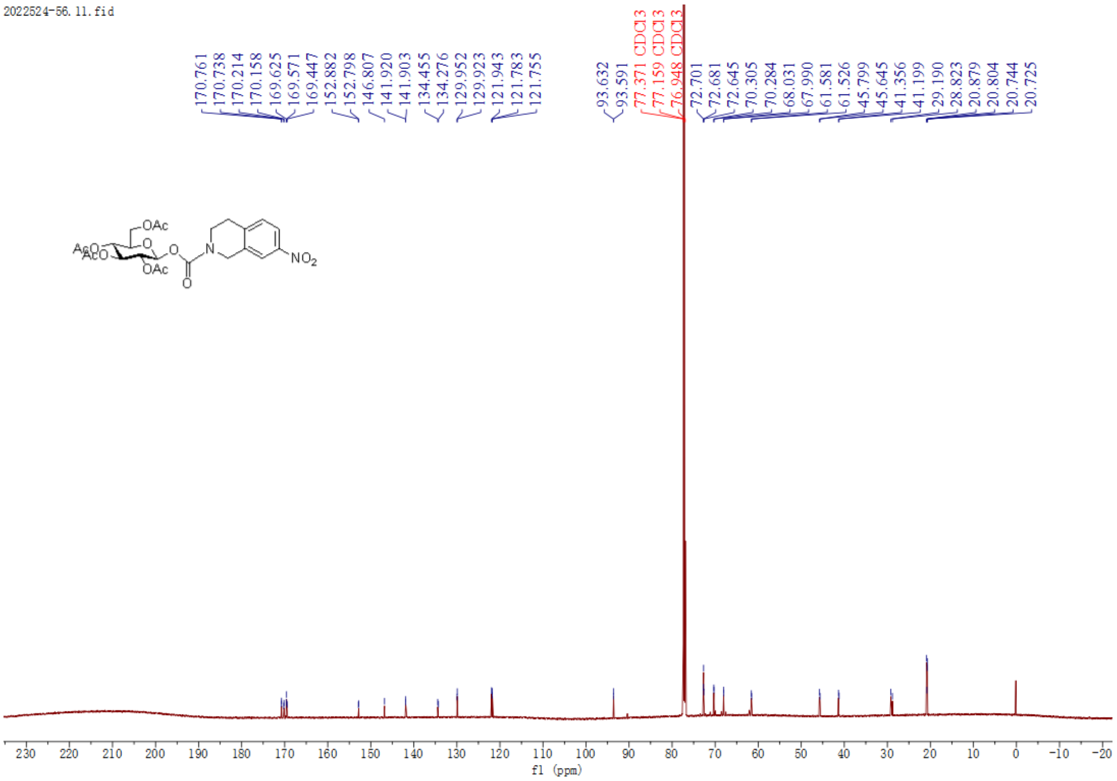

^{13}C NMR spectrum of compound **GA57** (151 MHz, $CDCl_3$)

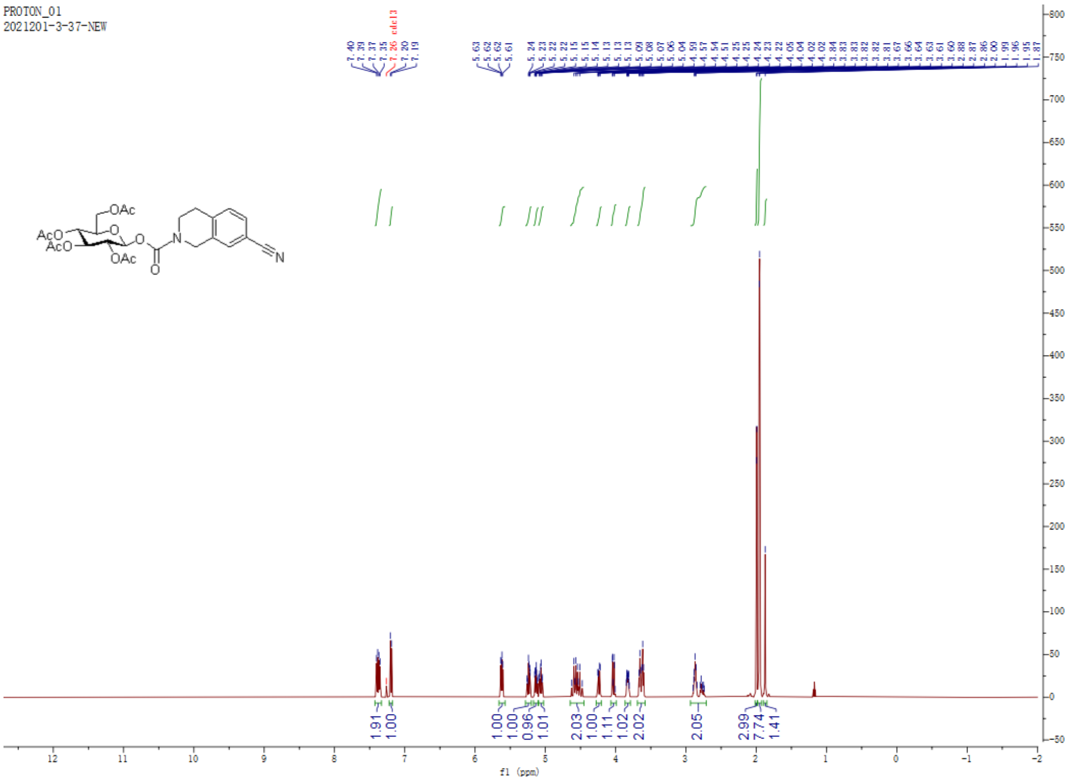

^{1}H NMR spectrum of compound **GA58** (500 MHz, $CDCl_3$)

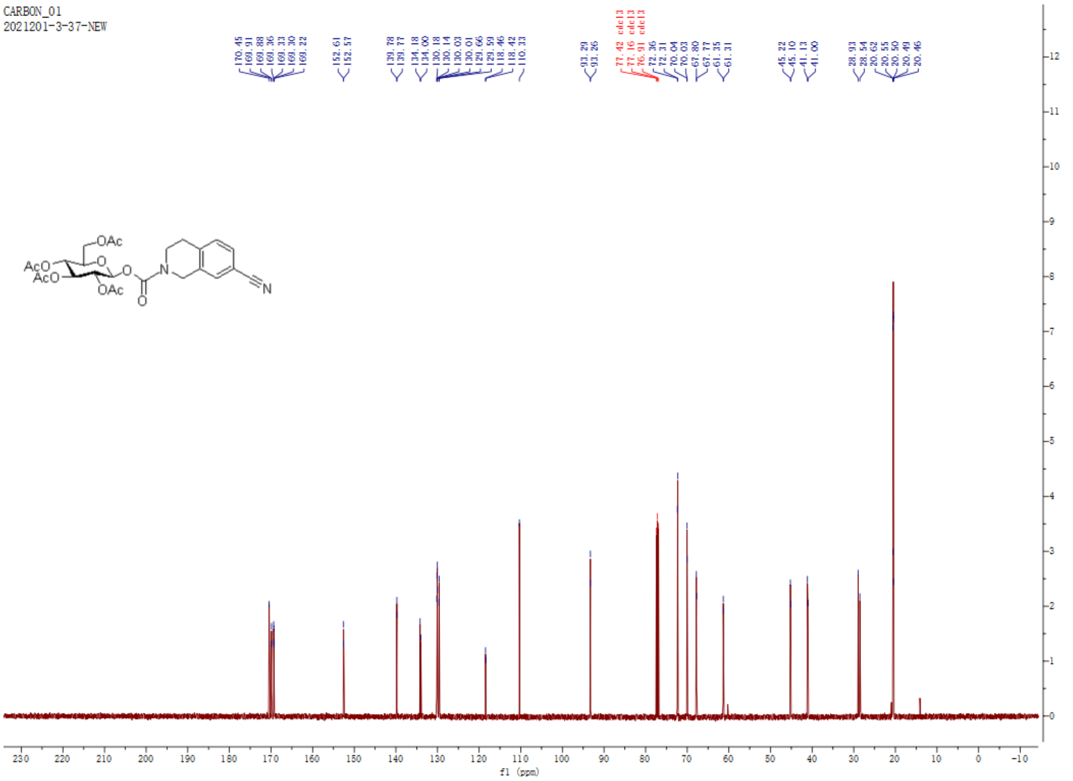

^{13}C NMR spectrum of compound **GA58** (126 MHz, $CDCl_3$)

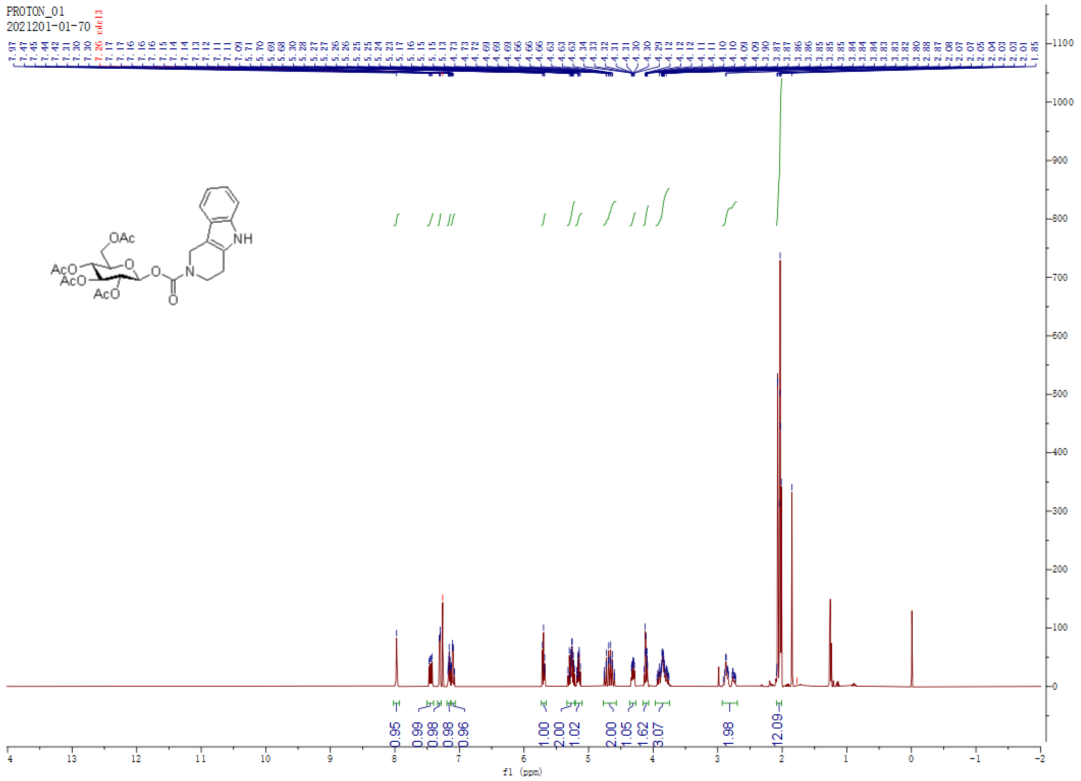

^{1}H NMR spectrum of compound **GA59** (500 MHz, $CDCl_3$)

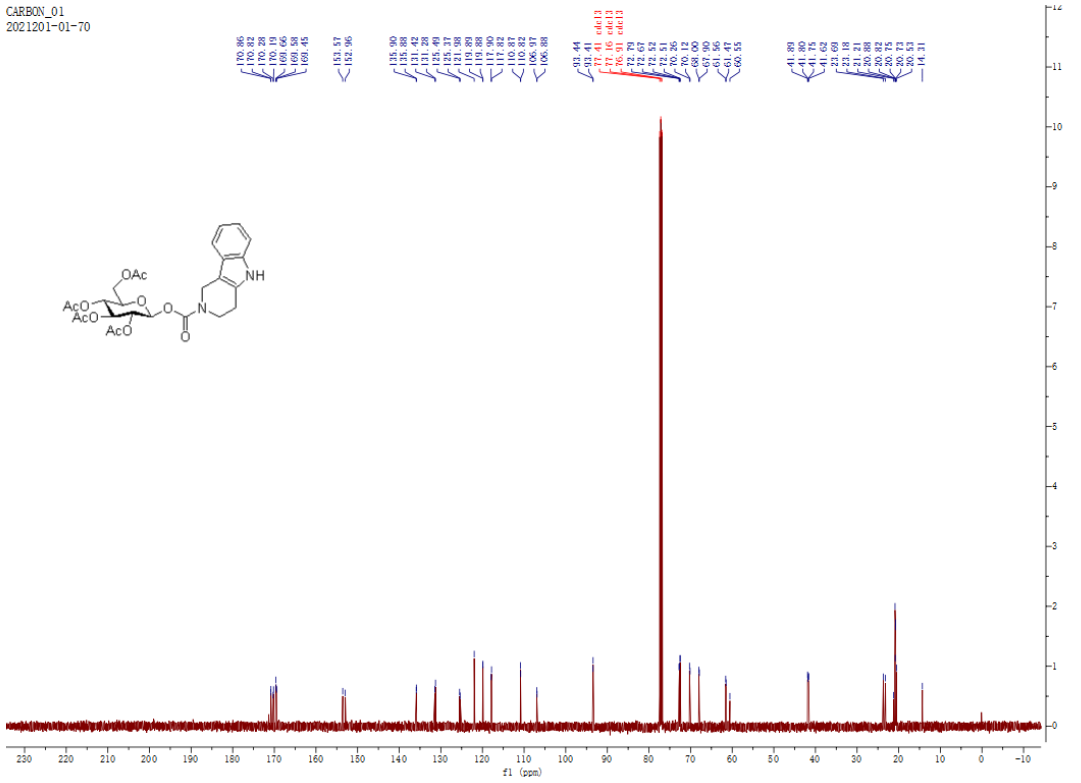

^{13}C NMR spectrum of compound **GA59** (126 MHz, $CDCl_3$)

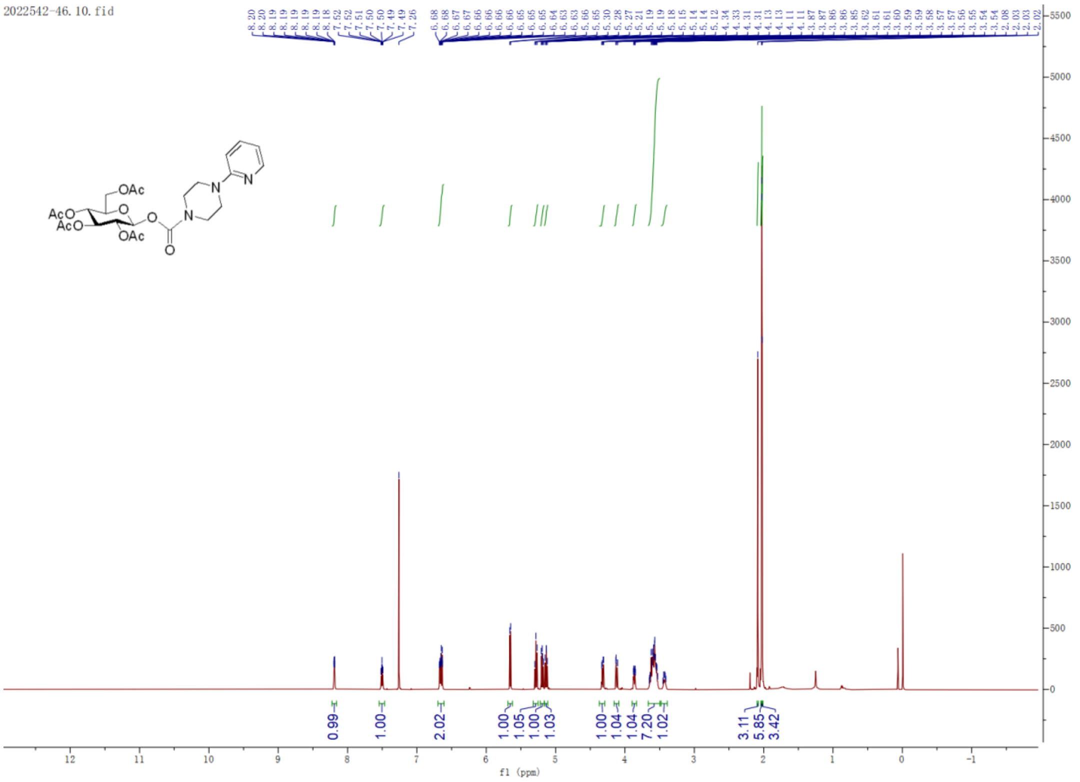

^{1}H NMR spectrum of compound **GA60** (600 MHz, $CDCl_3$)

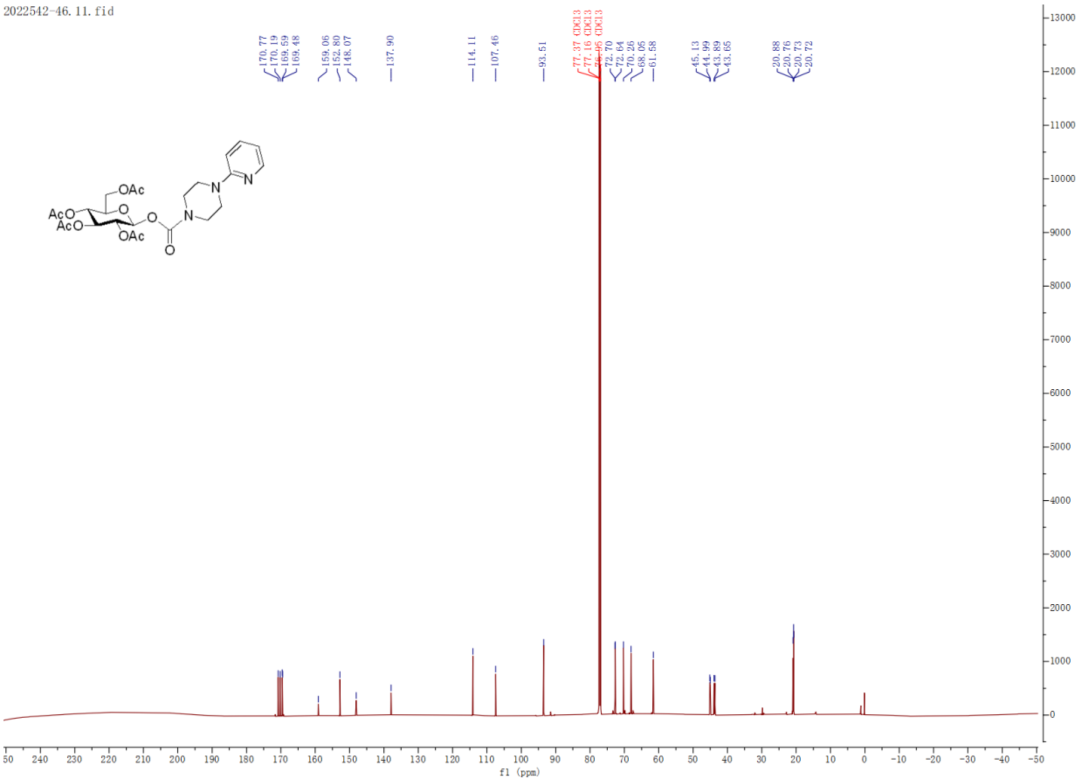

^{13}C NMR spectrum of compound **GA60** (151 MHz, $CDCl_3$)

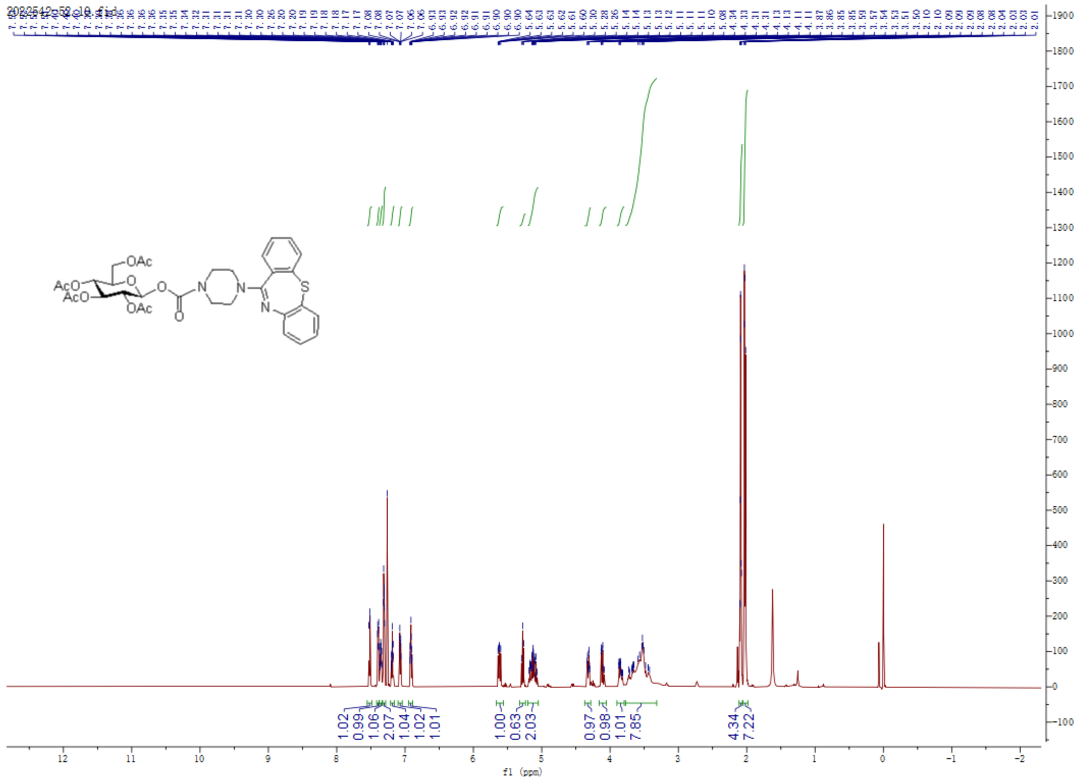

^{1}H NMR spectrum of compound **GA61** (600 MHz, $CDCl_3$)

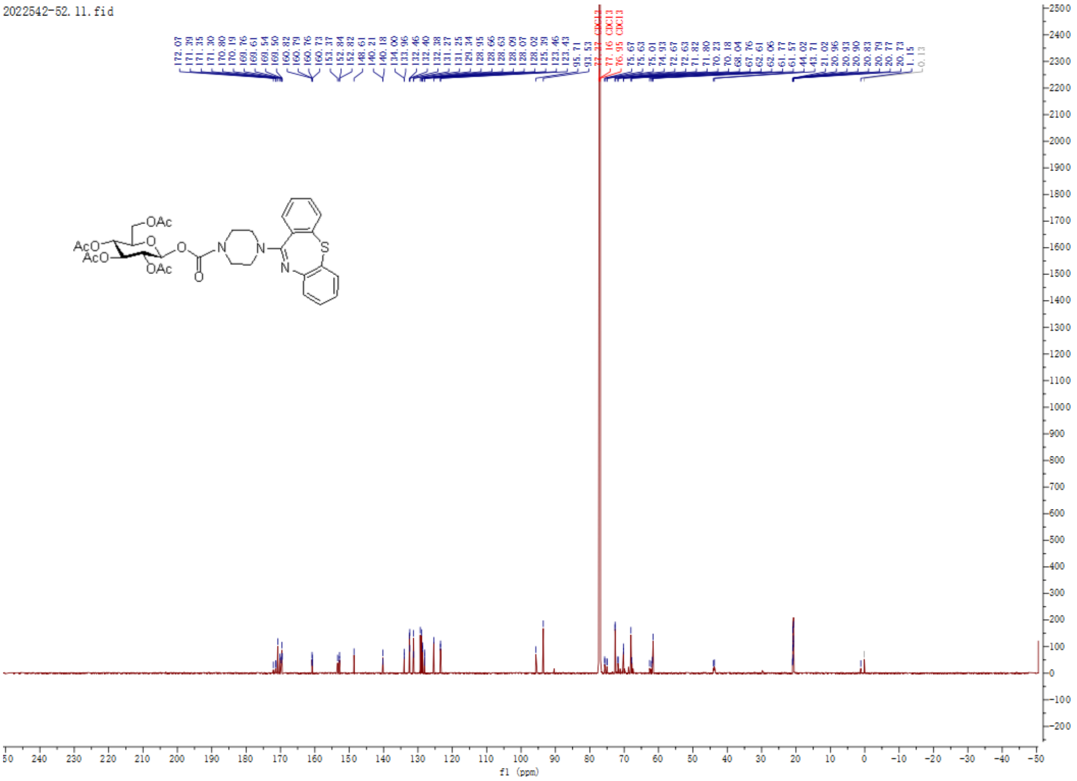

^{13}C NMR spectrum of compound **GA61** (151 MHz, $CDCl_3$)

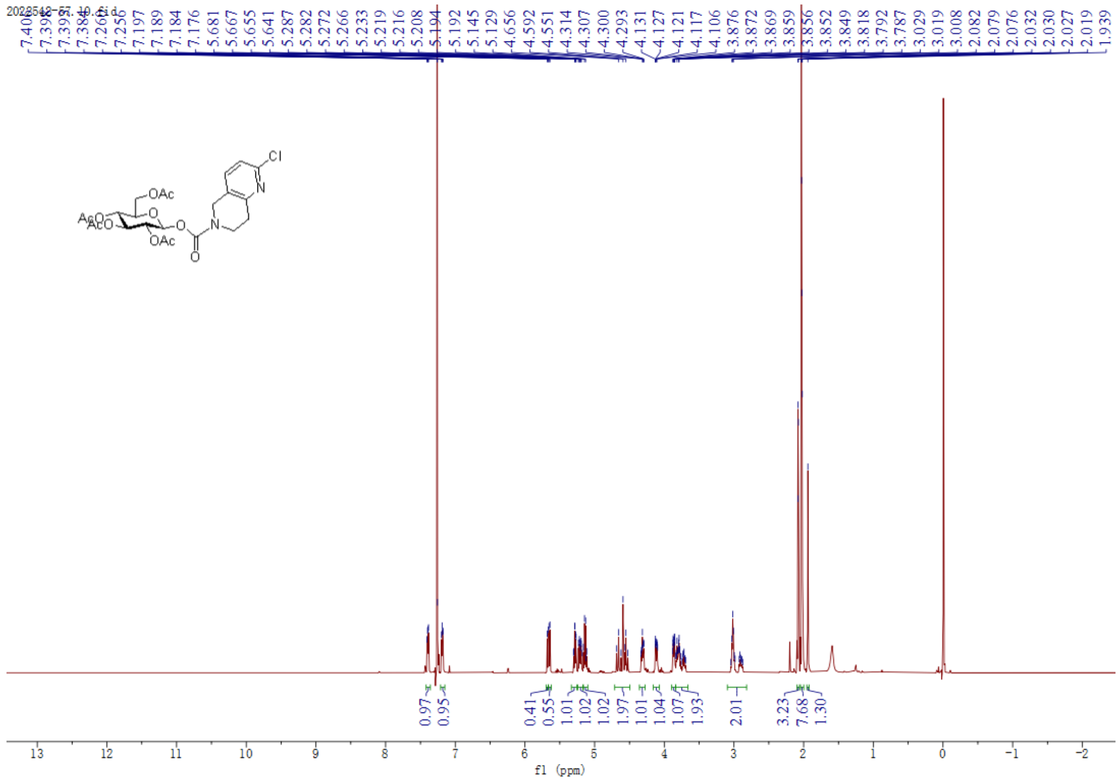

^{1}H NMR spectrum of compound **GA62** (600 MHz, $CDCl_3$)

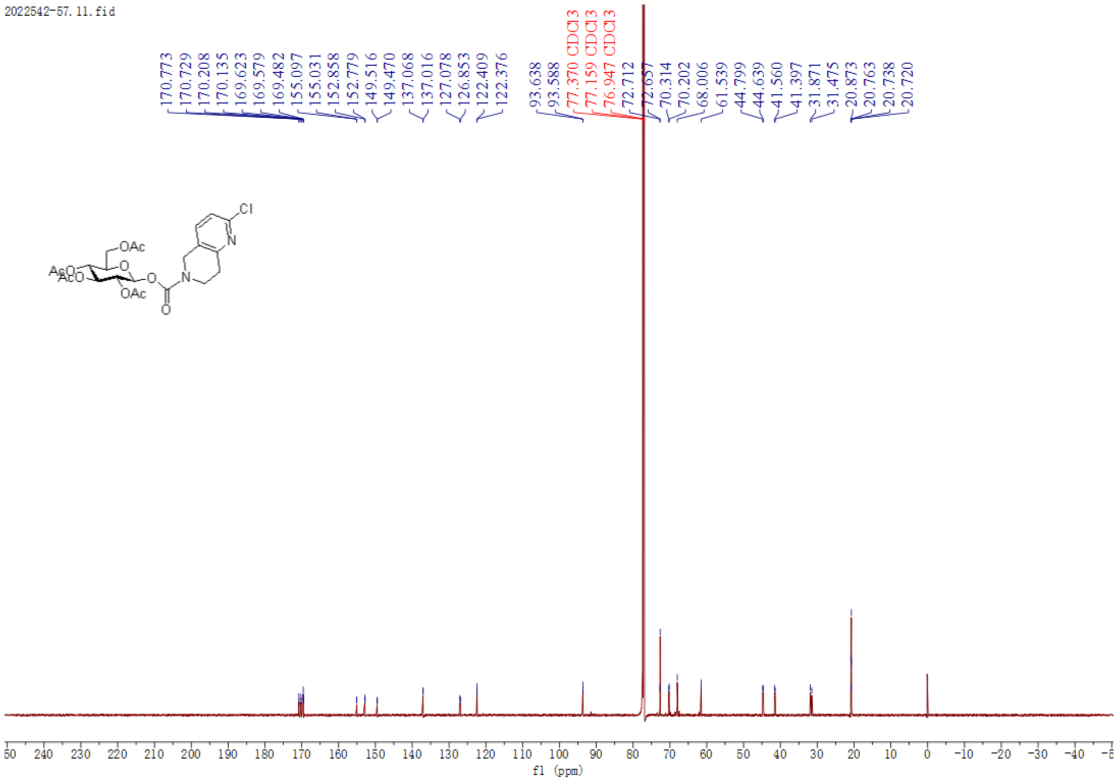

^{13}C NMR spectrum of compound **GA62** (151 MHz, $CDCl_3$)

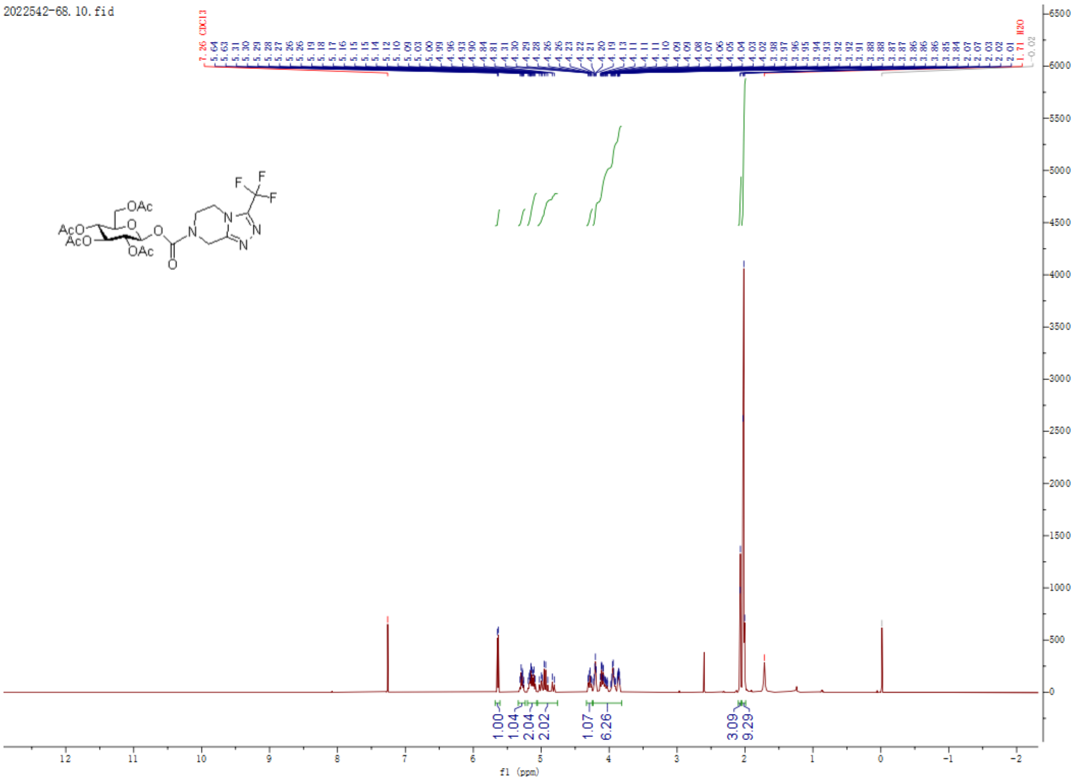

^{1}H NMR spectrum of compound **GA63** (600 MHz, $CDCl_3$)

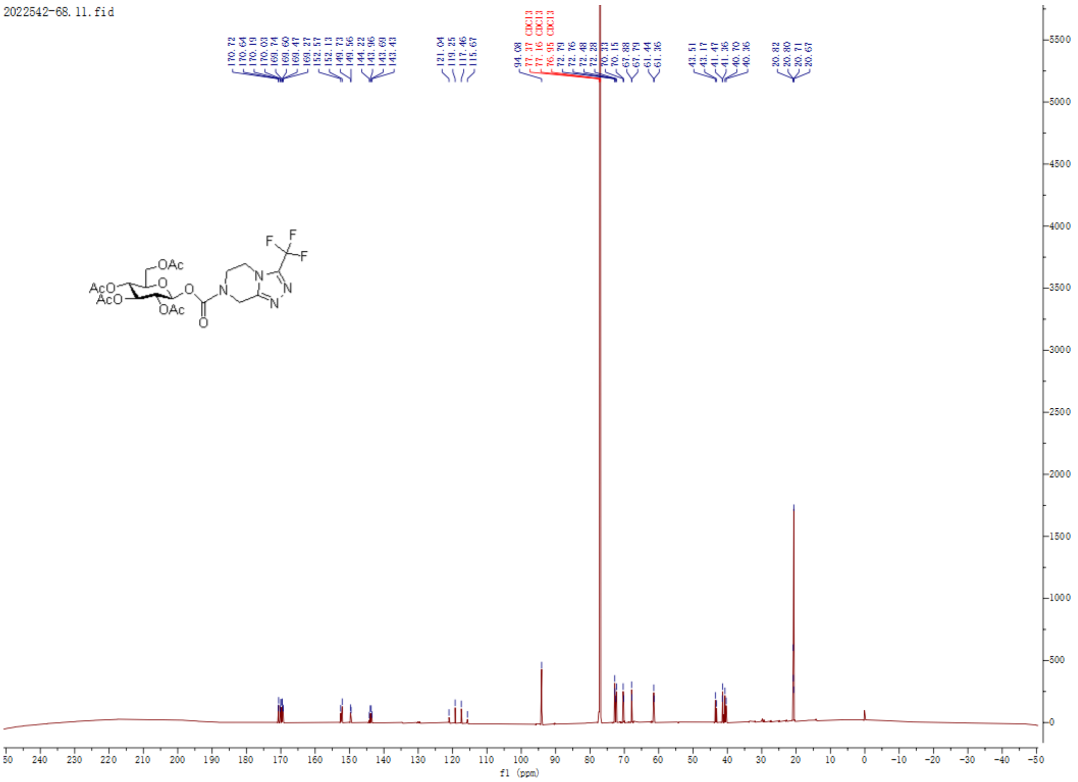

^{13}C NMR spectrum of compound **GA63** (151 MHz, $CDCl_3$)

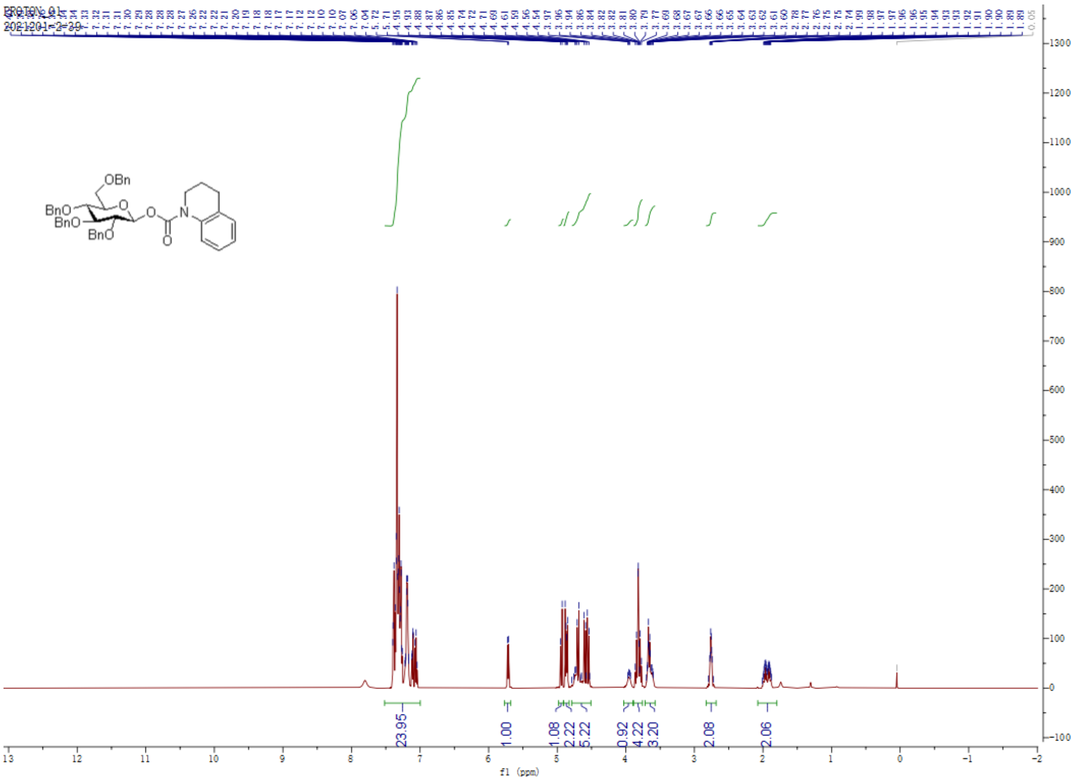

^{1}H NMR spectrum of compound **GA64** (500 MHz, $CDCl_3$)

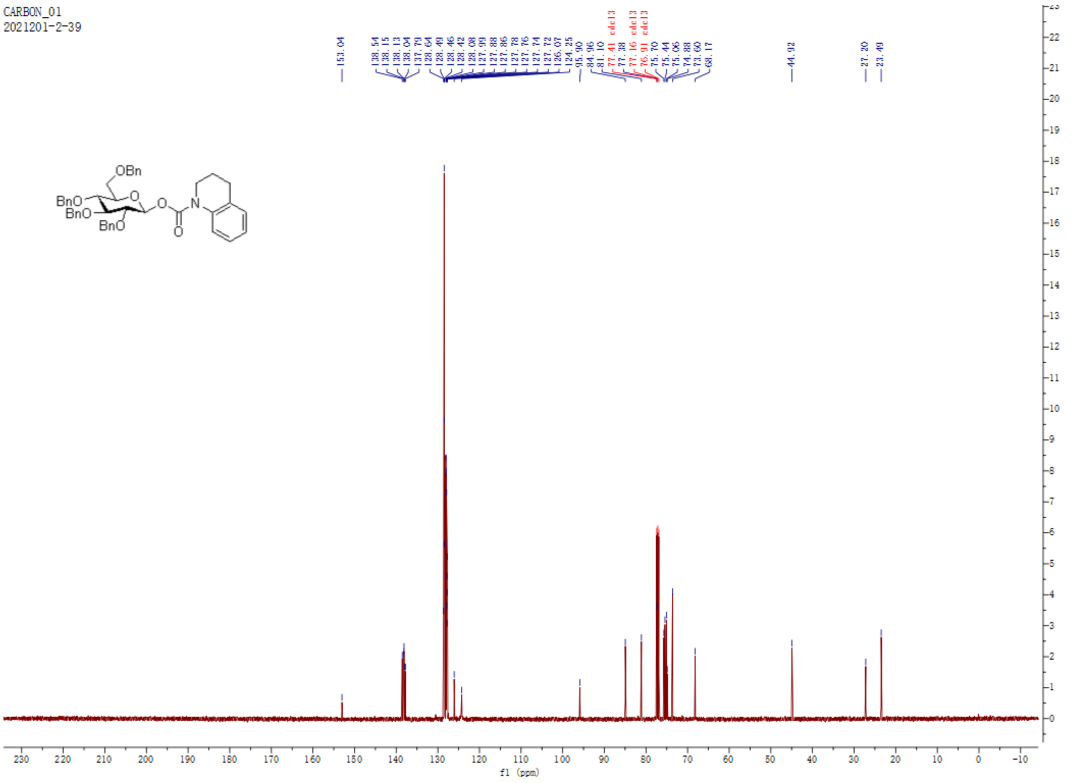

^{13}C NMR spectrum of compound **GA64** (126 MHz, $CDCl_3$)

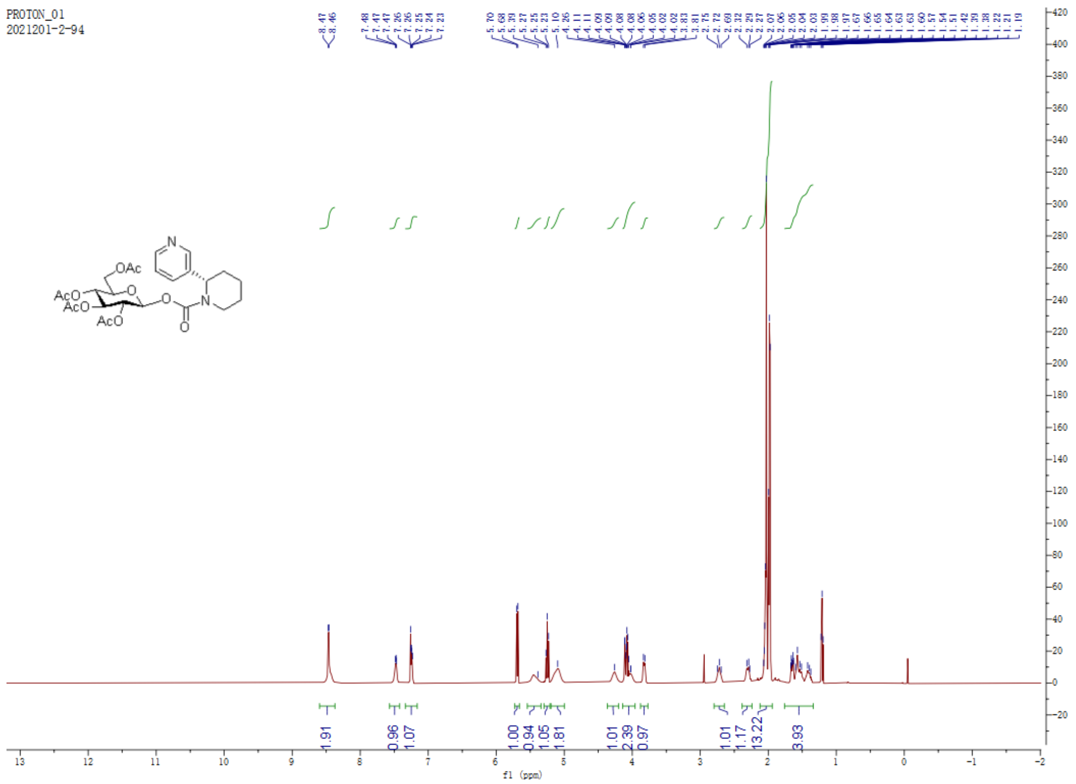

^{1}H NMR spectrum of compound **GA65** (500 MHz, $CDCl_3$)

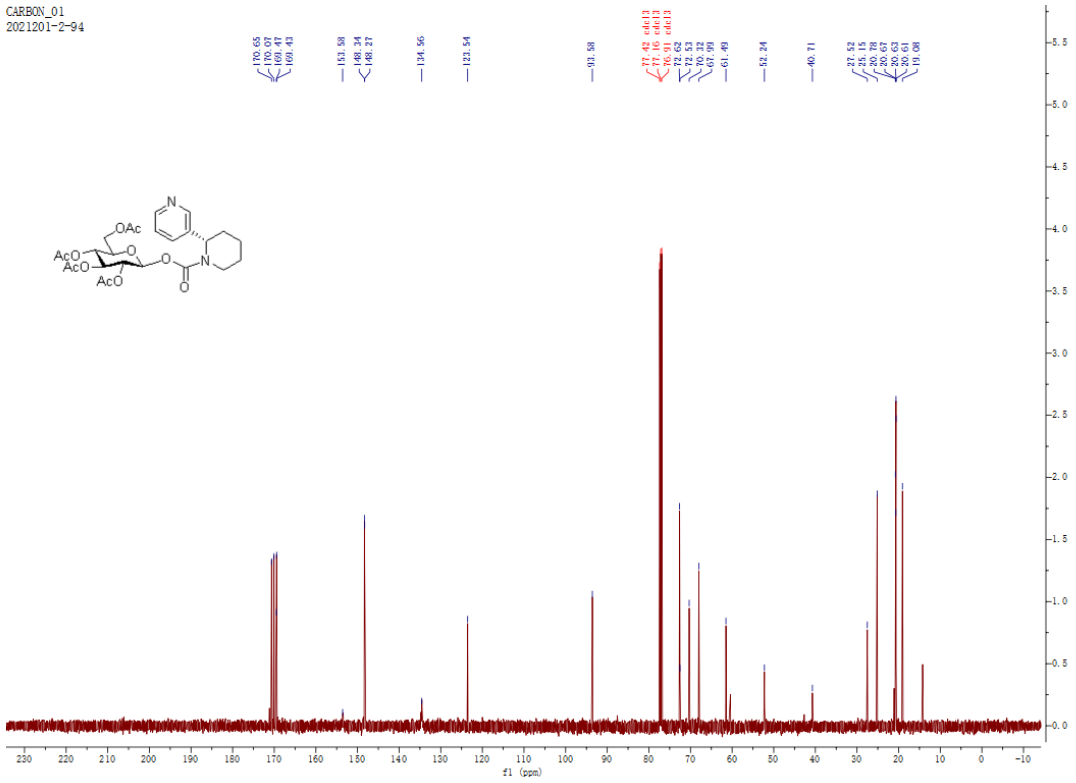

^{13}C NMR spectrum of compound **GA65** (126 MHz, $CDCl_3$)

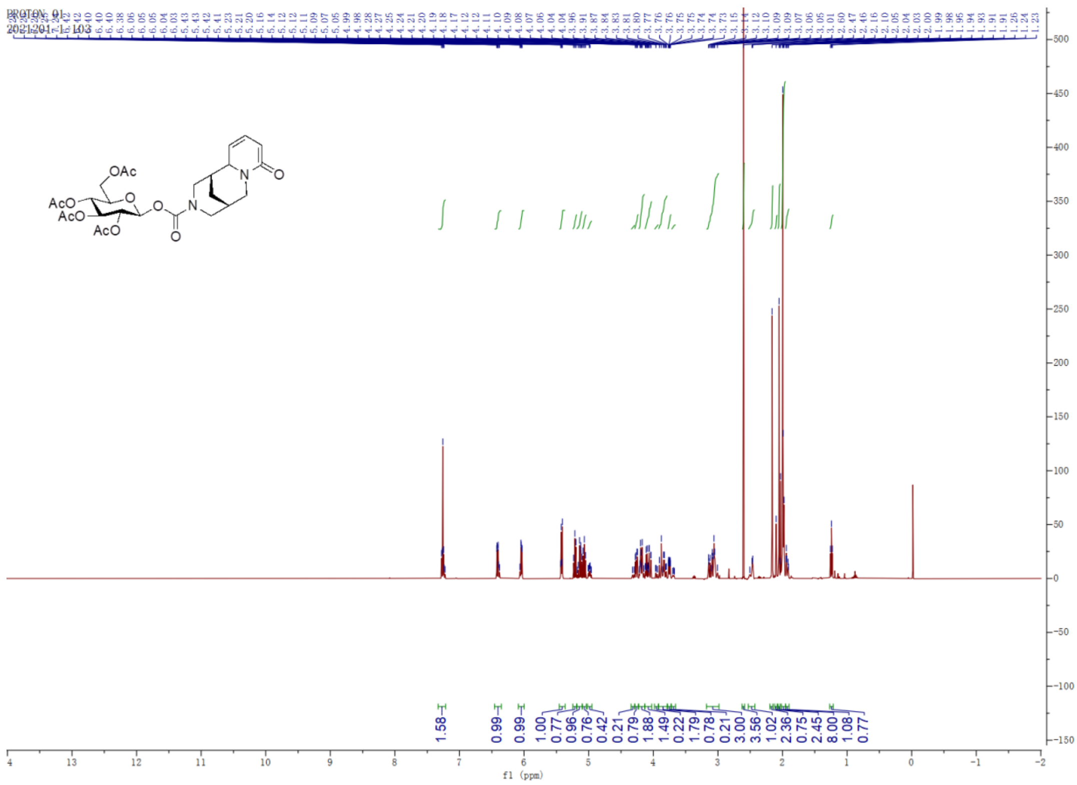

^{1}H NMR spectrum of compound **GA66** (500 MHz, $CDCl_3$)

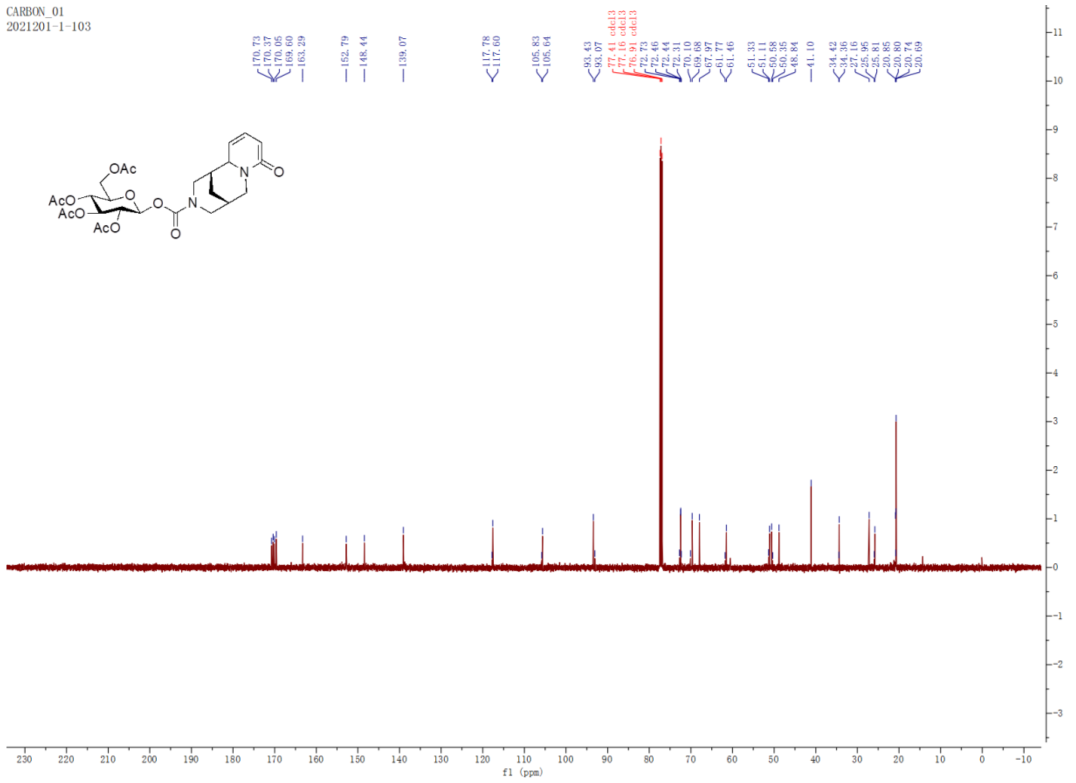

^{13}C NMR spectrum of compound **GA66** (126 MHz, $CDCl_3$)

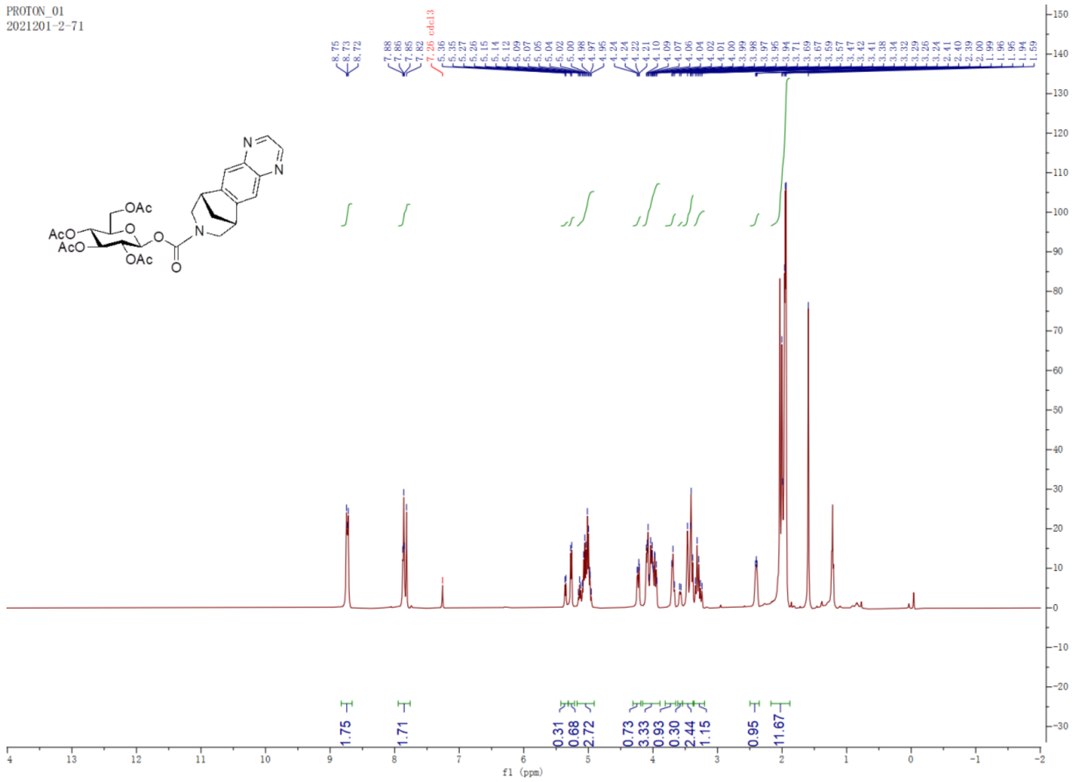

^{1}H NMR spectrum of compound **GA67** (500 MHz, $CDCl_3$)

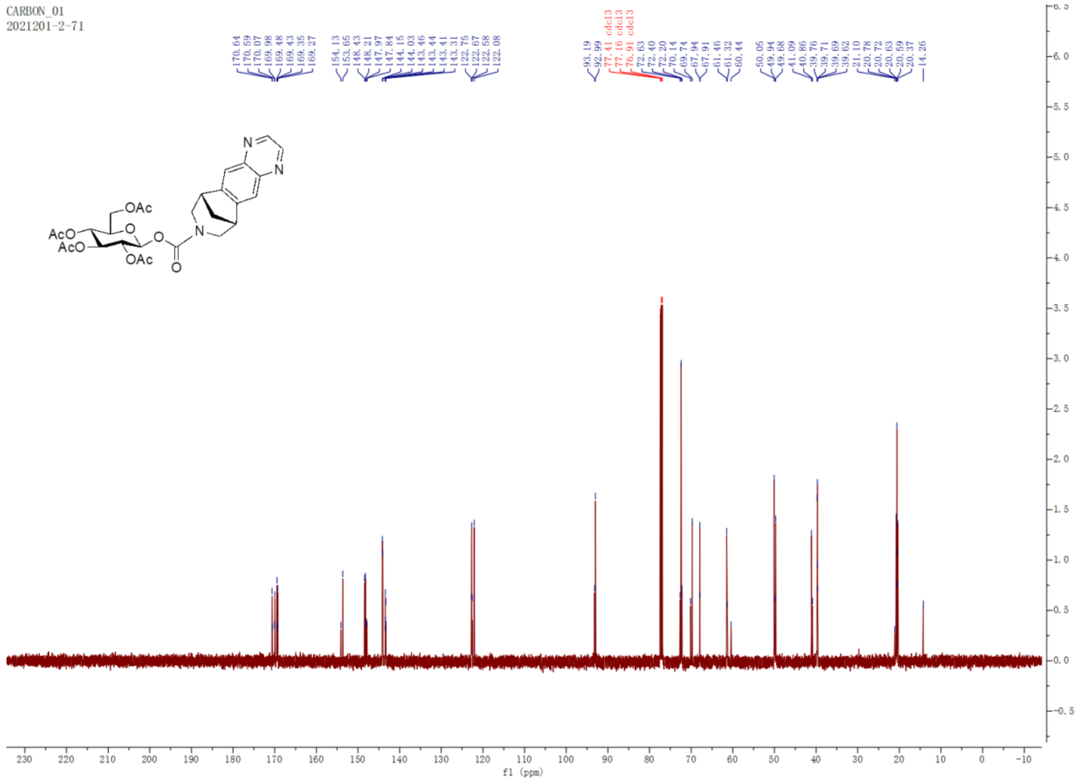

^{13}C NMR spectrum of compound **GA67** (126 MHz, $CDCl_3$)

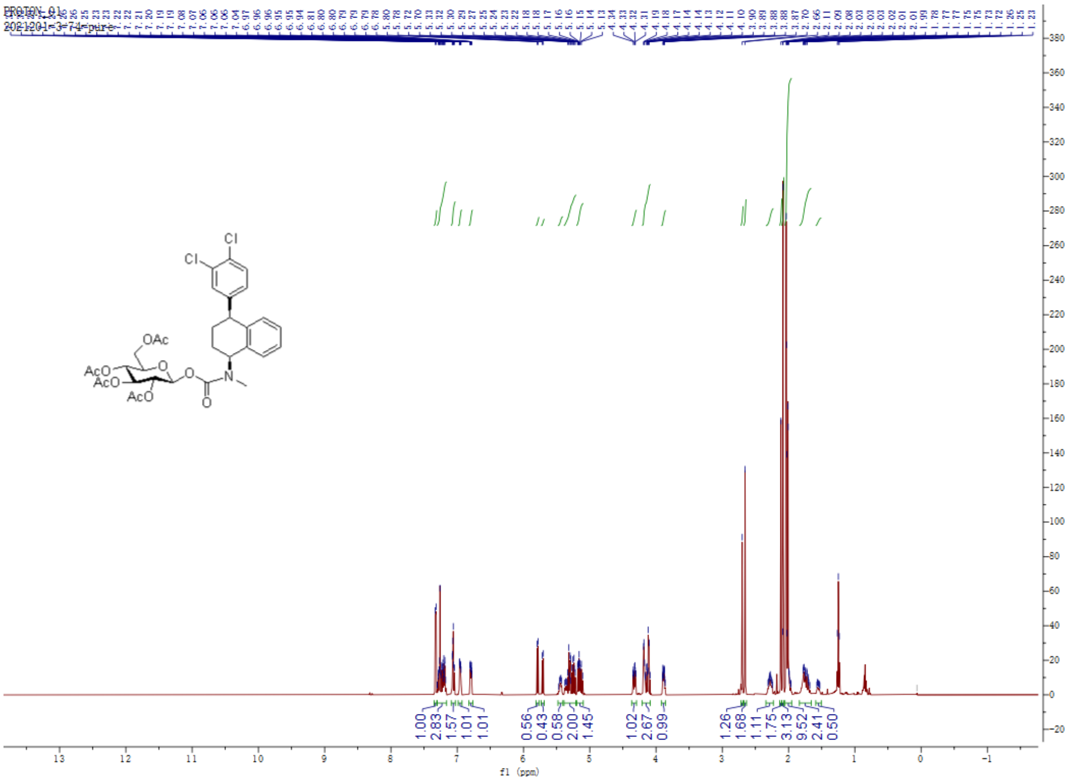

^{1}H NMR spectrum of compound **GA68** (500 MHz, $CDCl_3$)

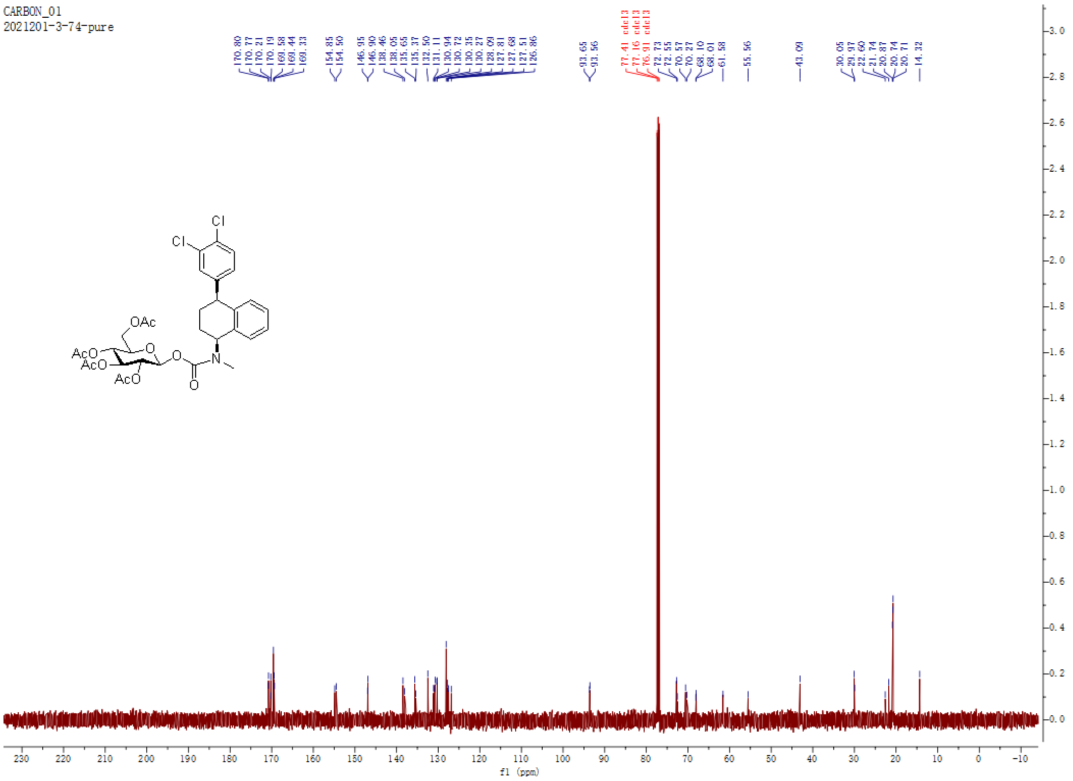

^{13}C NMR spectrum of compound **GA68** (126 MHz, $CDCl_3$)

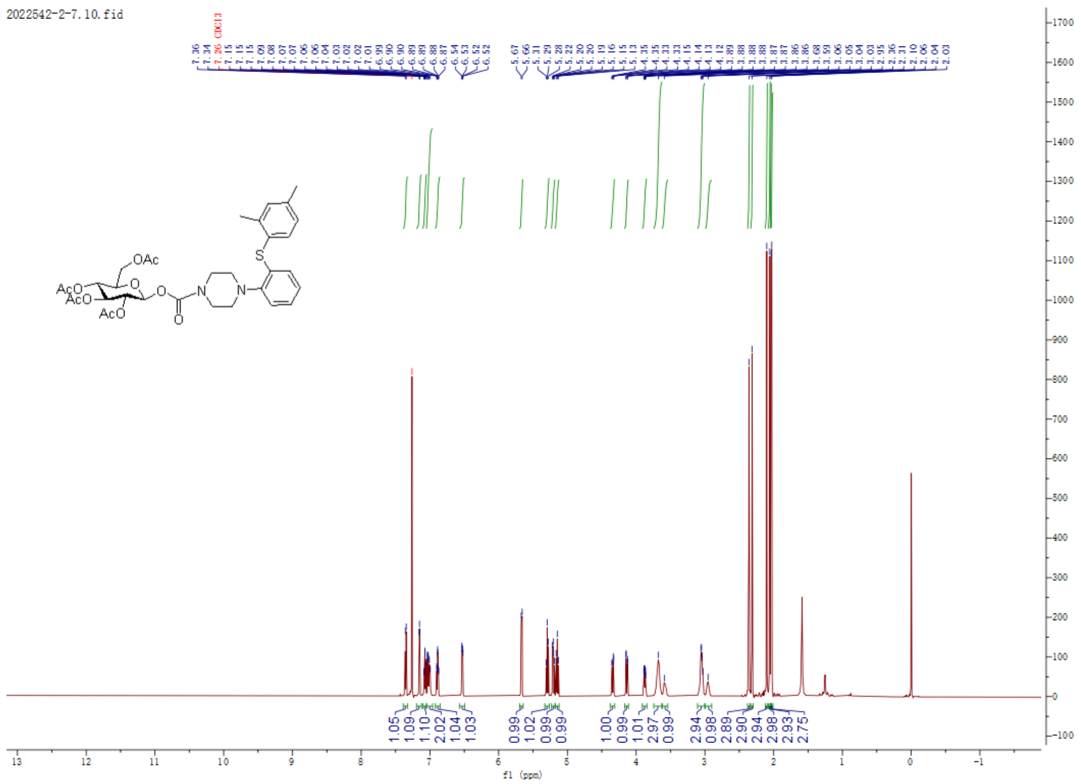

^{1}H NMR spectrum of compound **GA69** (600 MHz, $CDCl_3$)

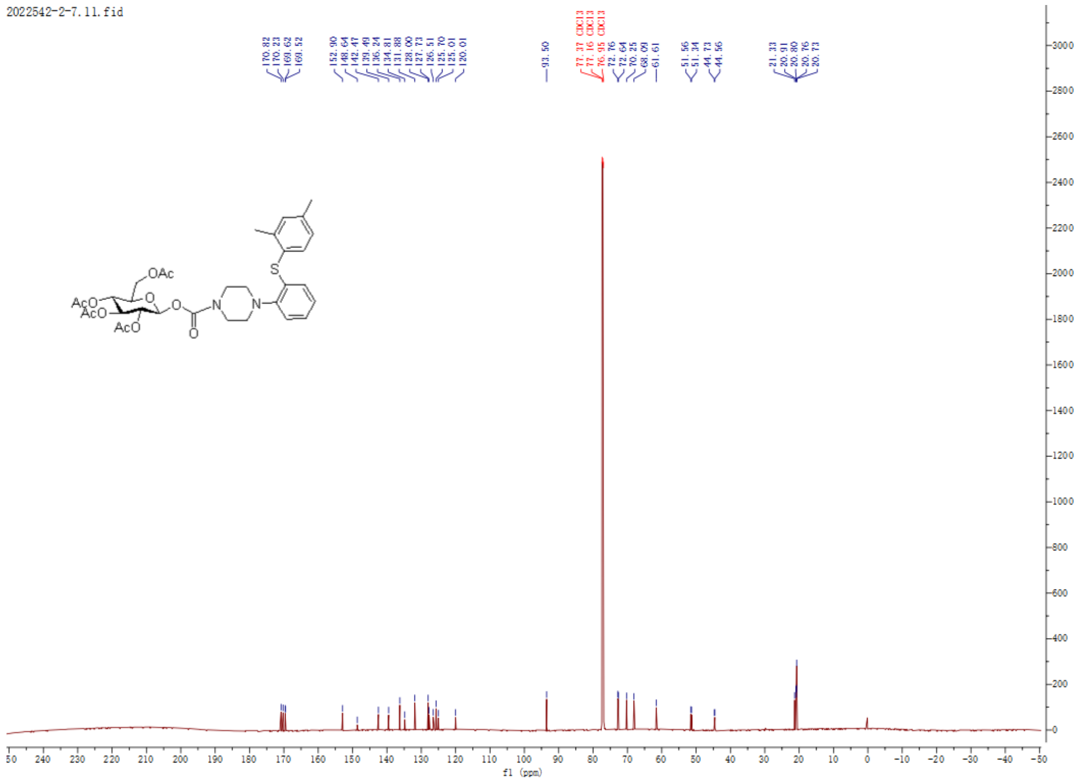

^{13}C NMR spectrum of compound **GA69** (151 MHz, $CDCl_3$)

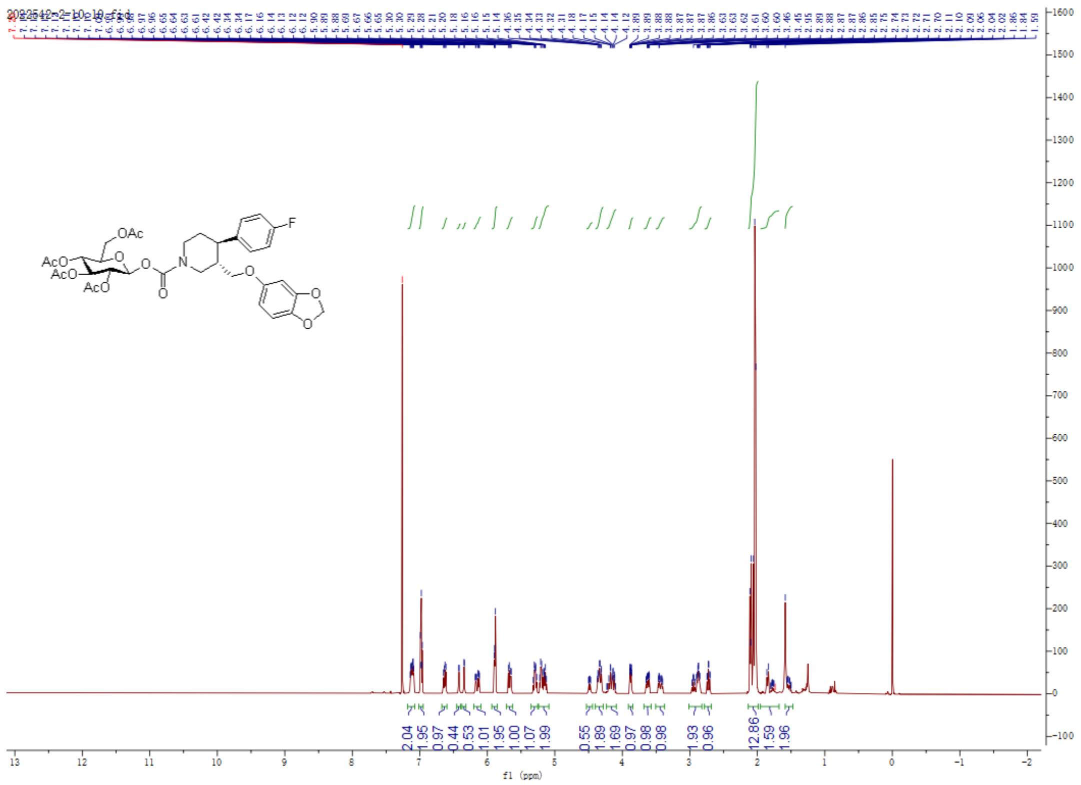

^{1}H NMR spectrum of compound **GA70** (600 MHz, $CDCl_3$)

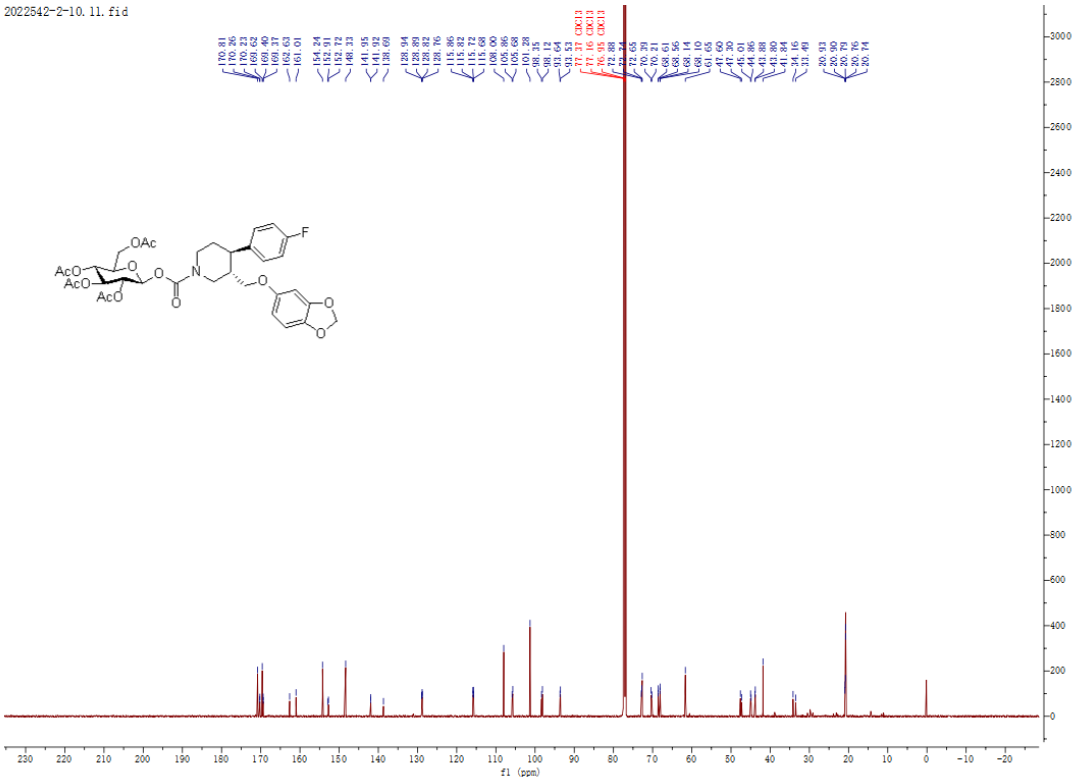

^{13}C NMR spectrum of compound **GA70** (151 MHz, $CDCl_3$)

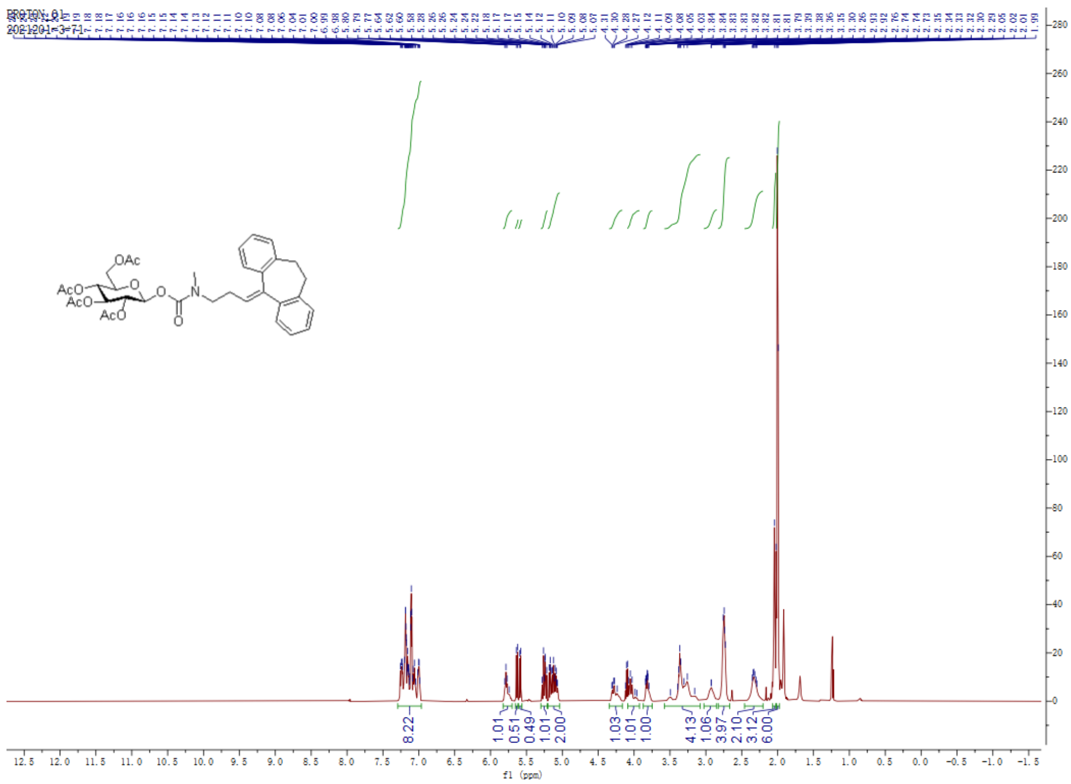

^{1}H NMR spectrum of compound **GA71** (600 MHz, $CDCl_3$)

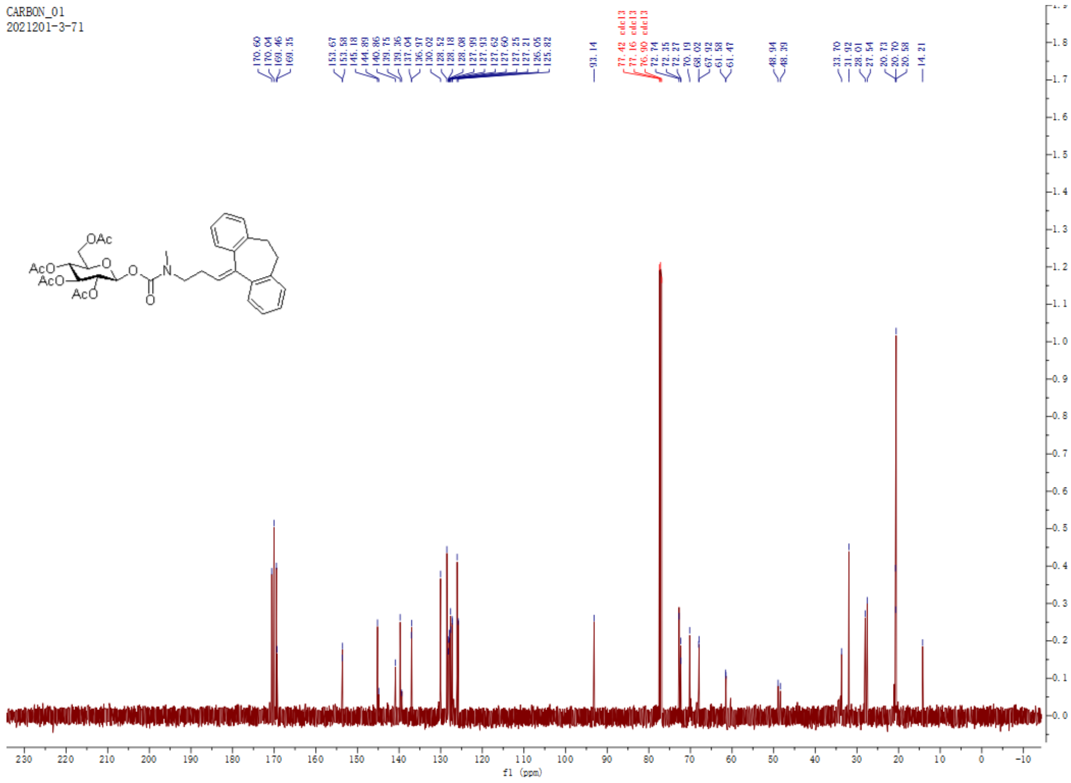

^{13}C NMR spectrum of compound **GA71** (151 MHz, $CDCl_3$)

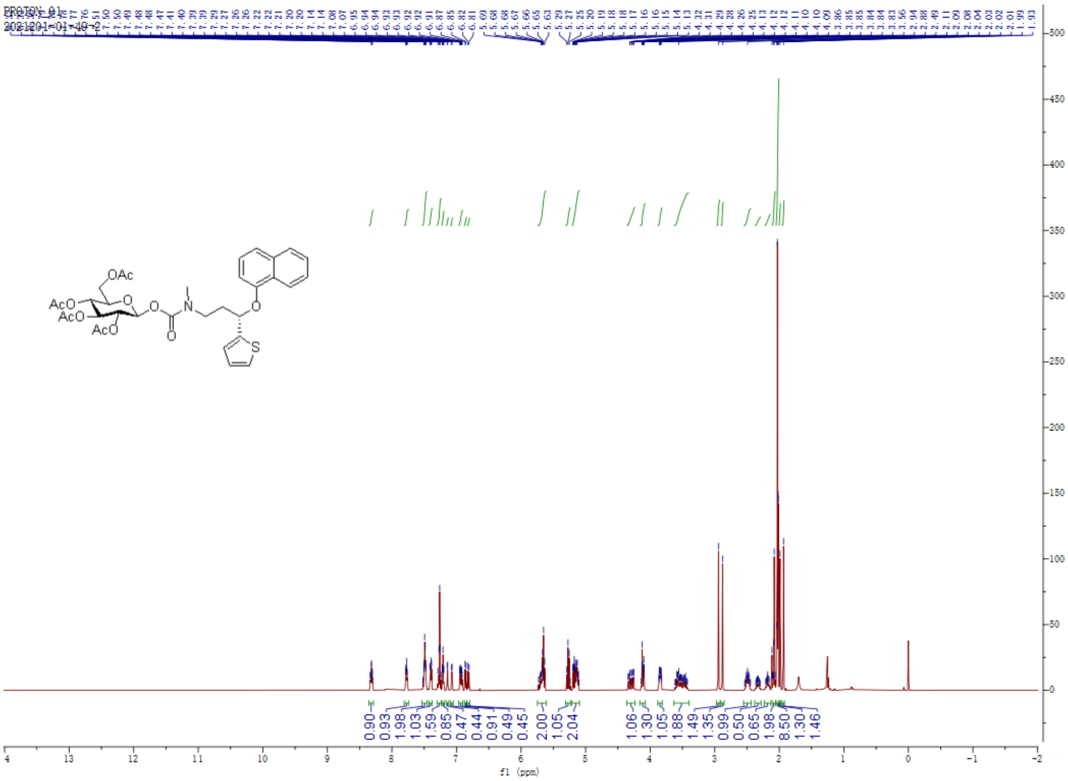

^{1}H NMR spectrum of compound **GA72** (500 MHz, $CDCl_3$)

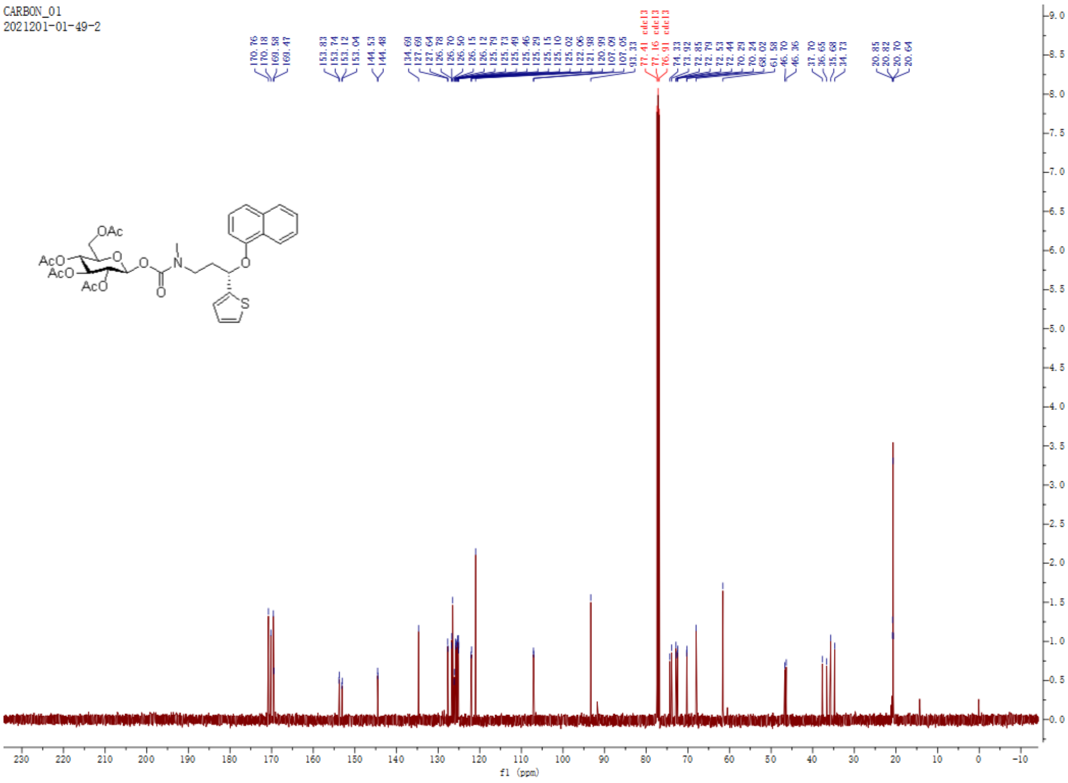

^{13}C NMR spectrum of compound **GA72** (126 MHz, $CDCl_3$)

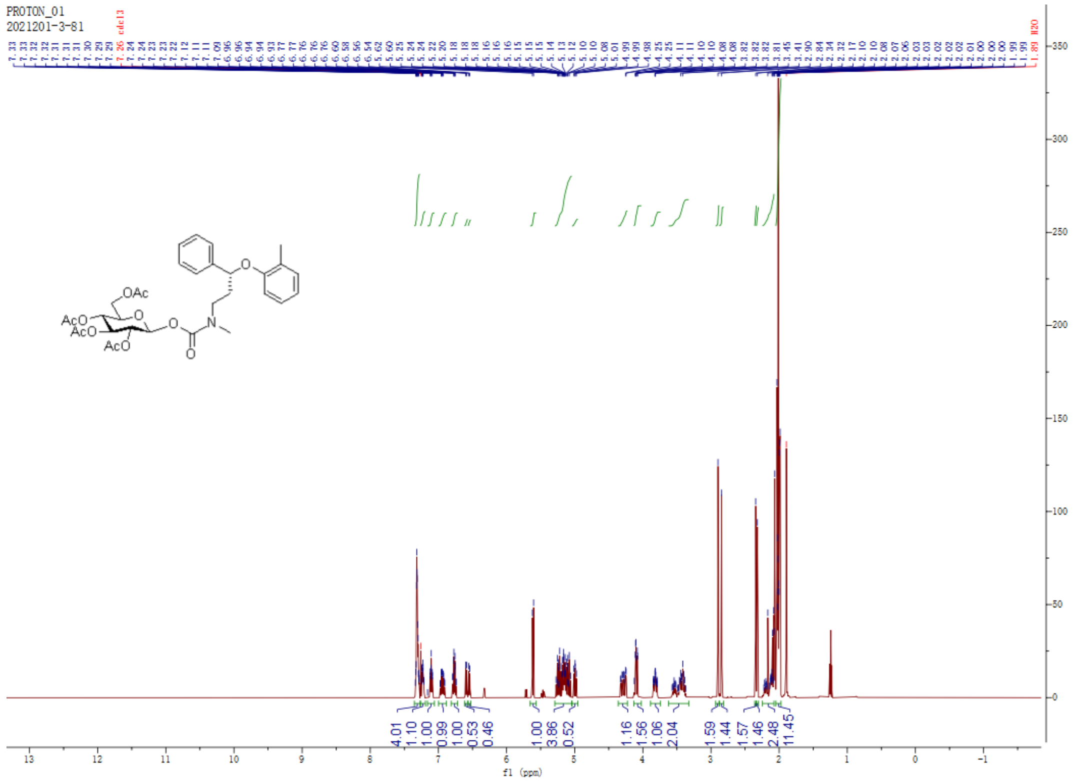

^{1}H NMR spectrum of compound **GA73** (500 MHz, $CDCl_3$)

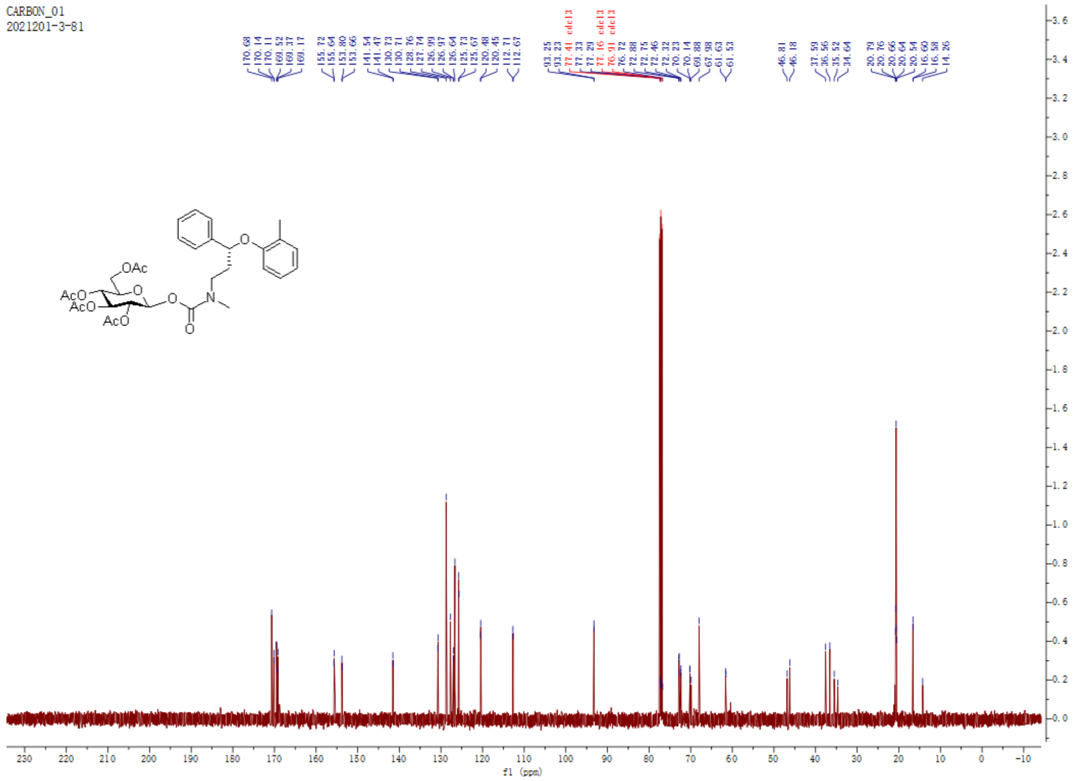

^{13}C NMR spectrum of compound **GA73** (126 MHz, $CDCl_3$)

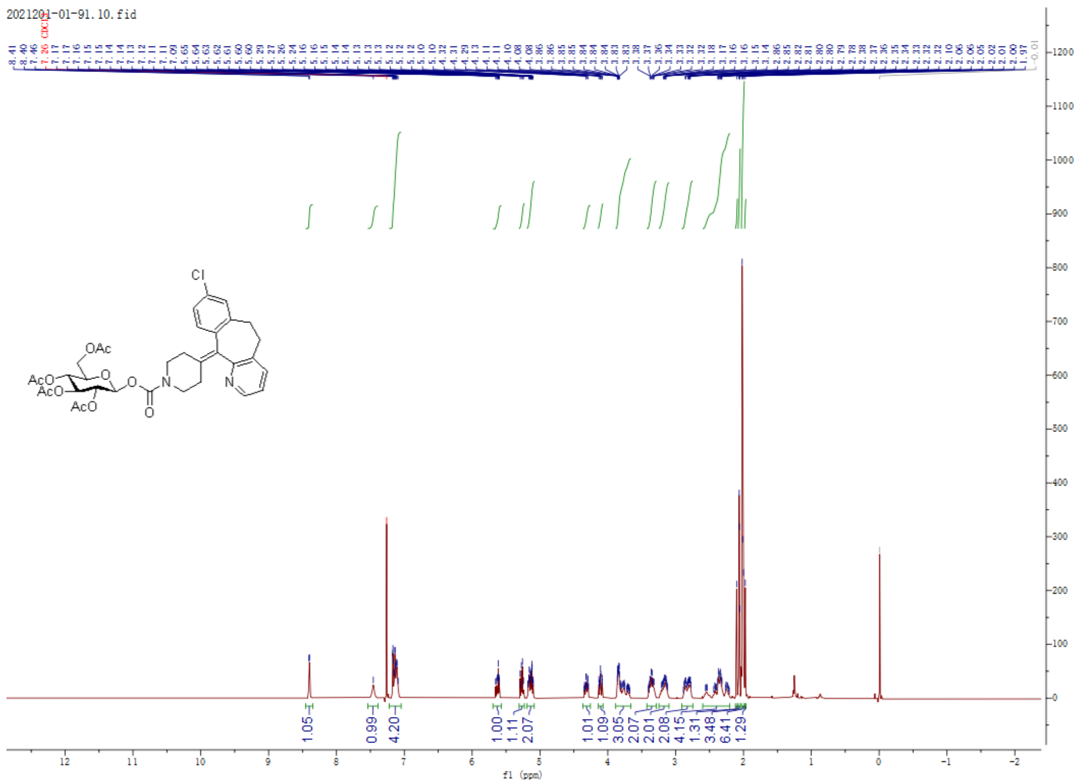

^{1}H NMR spectrum of compound **GA74** (600 MHz, $CDCl_3$)

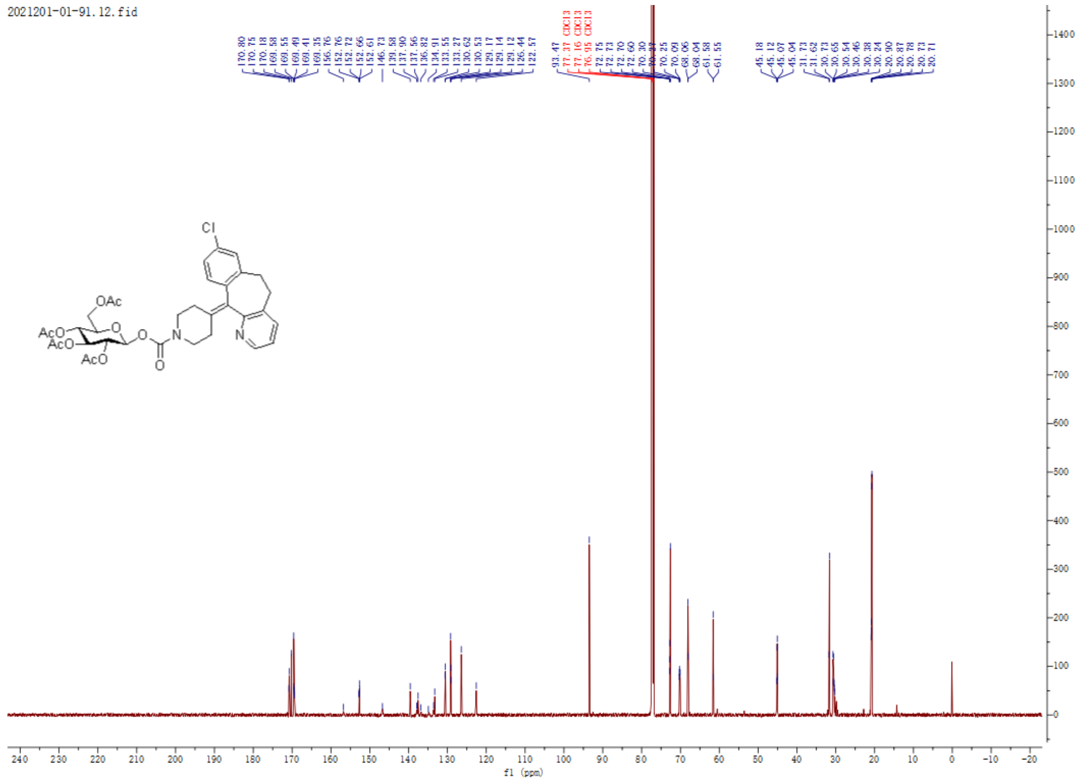

^{13}C NMR spectrum of compound **GA74** (151 MHz, $CDCl_3$)

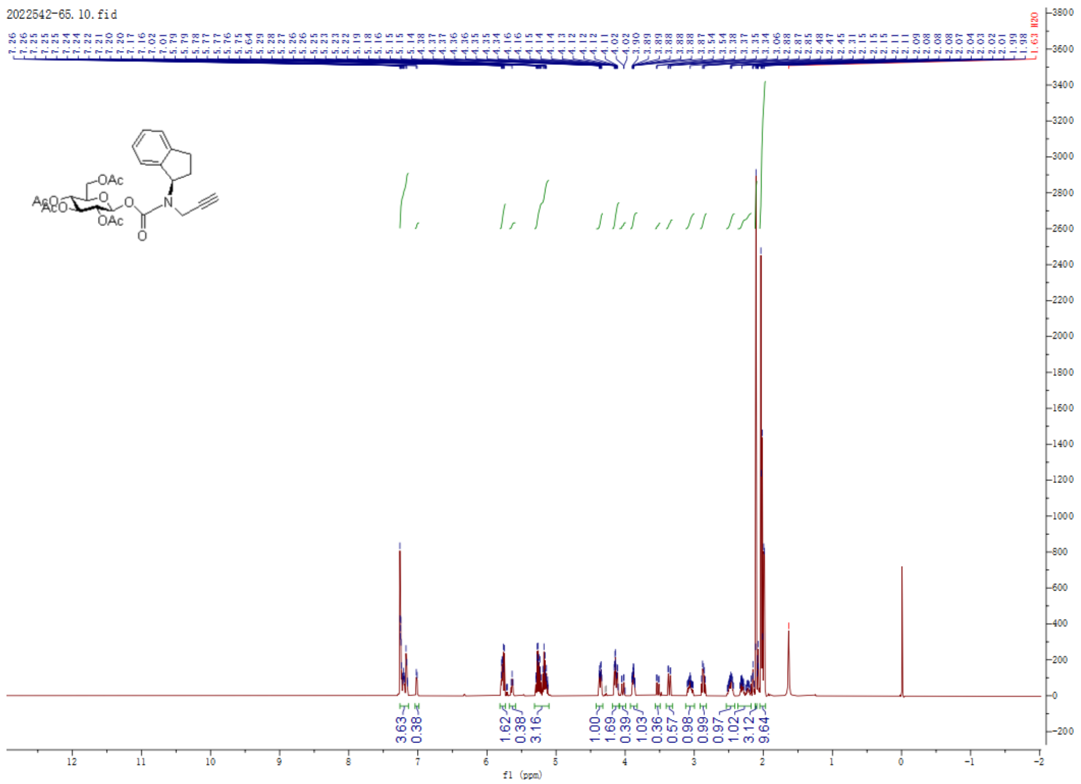

^{1}H NMR spectrum of compound **GA75** (600 MHz, $CDCl_3$)

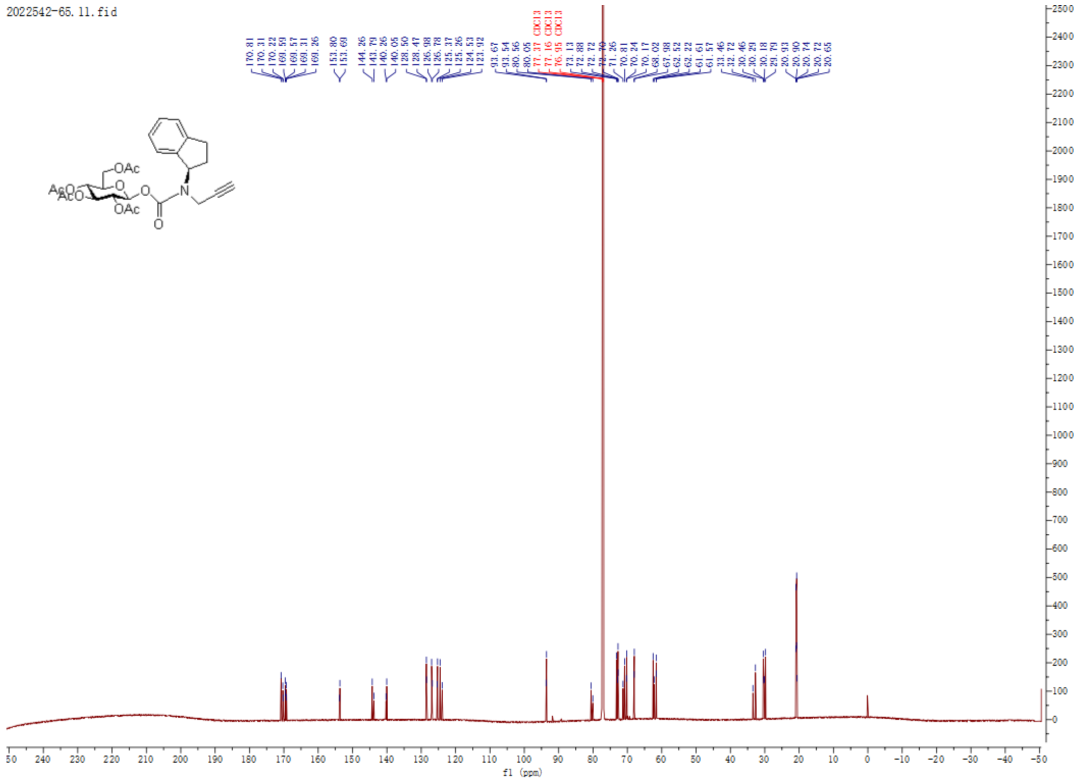

^{13}C NMR spectrum of compound **GA75** (151 MHz, $CDCl_3$)

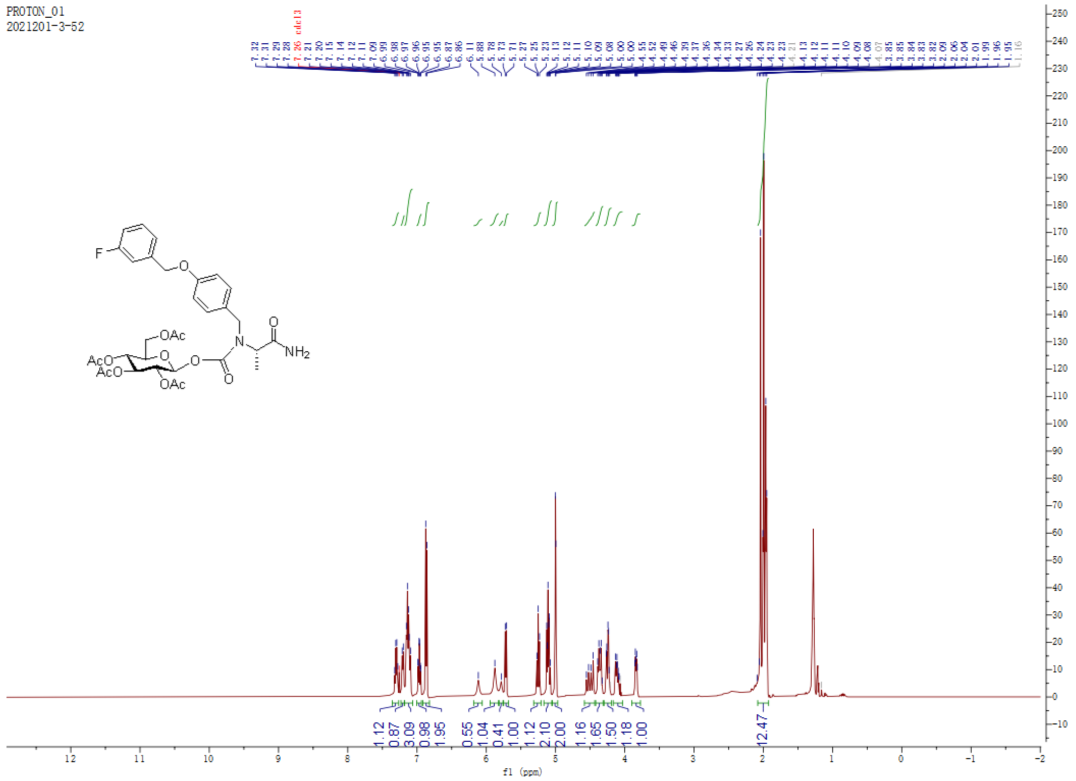

^{1}H NMR spectrum of compound **GA76** (500 MHz, $CDCl_3$)

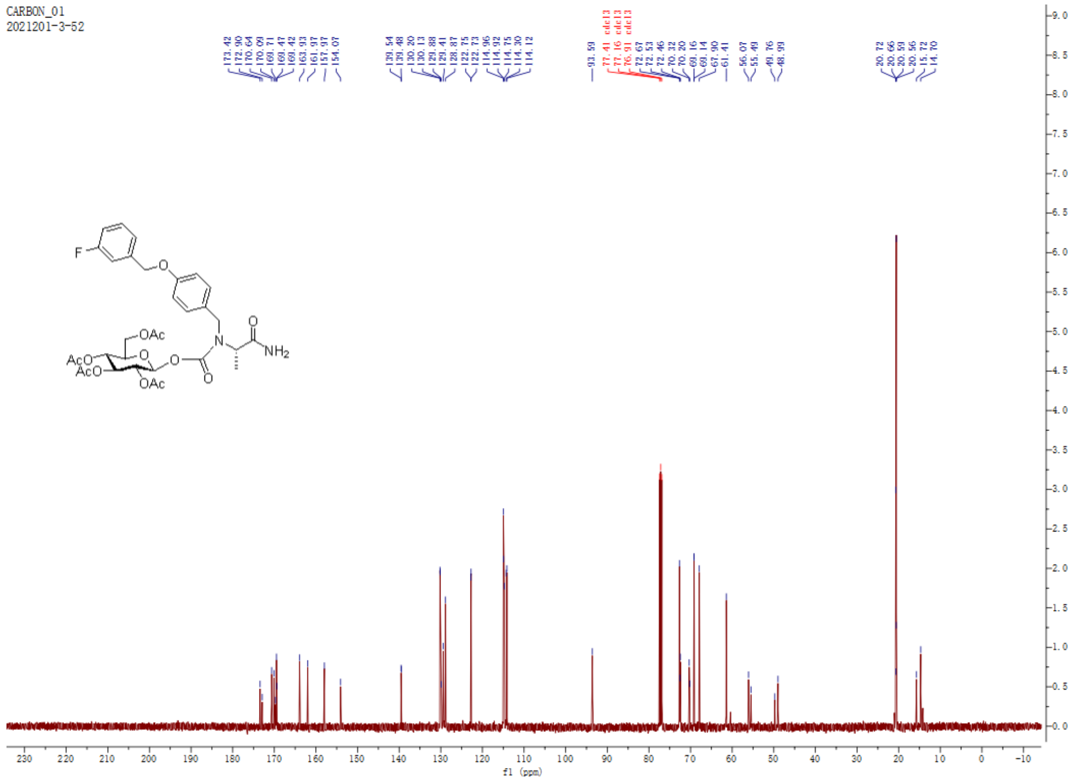

^{13}C NMR spectrum of compound **GA76** (126 MHz, $CDCl_3$)

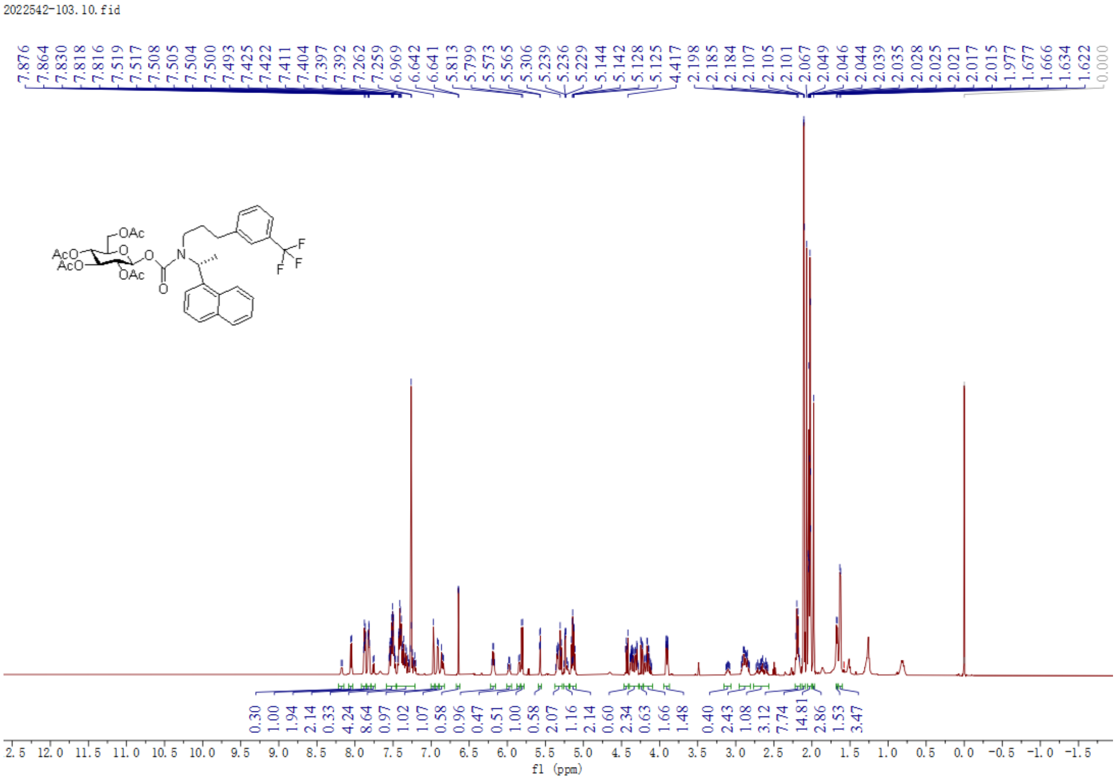

^{1}H NMR spectrum of compound **GA77** (600 MHz, $CDCl_3$)

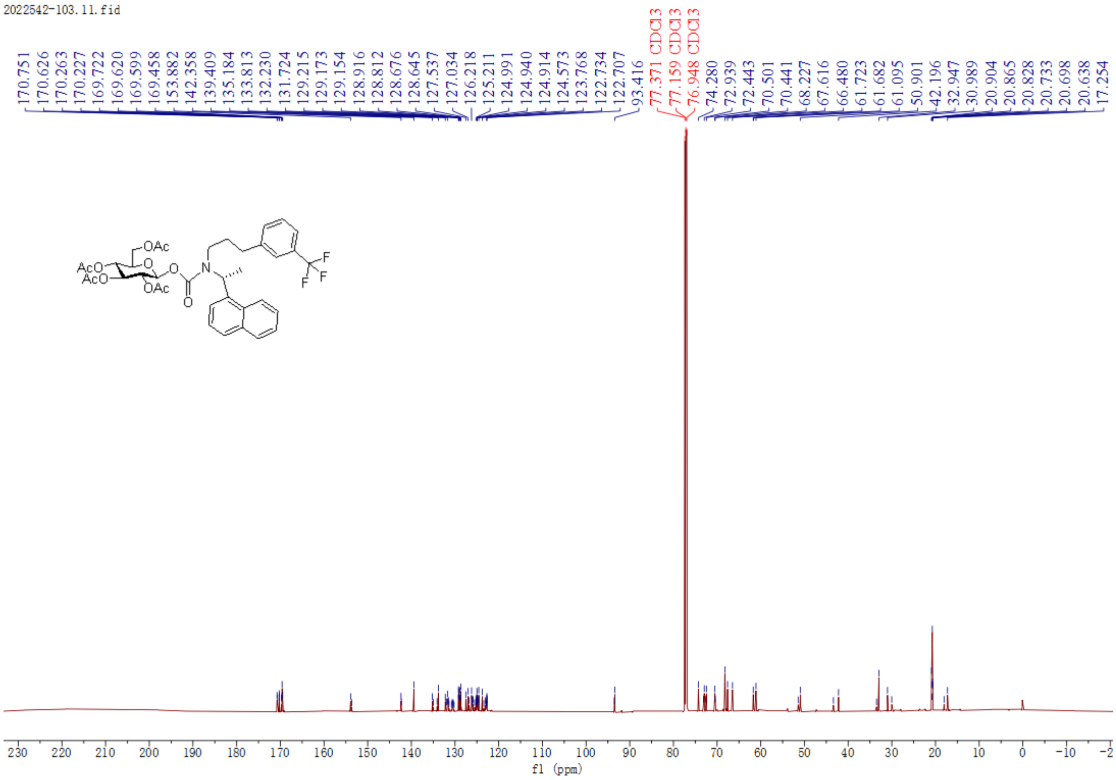

^{13}C NMR spectrum of compound **GA77** (151 MHz, $CDCl_3$)

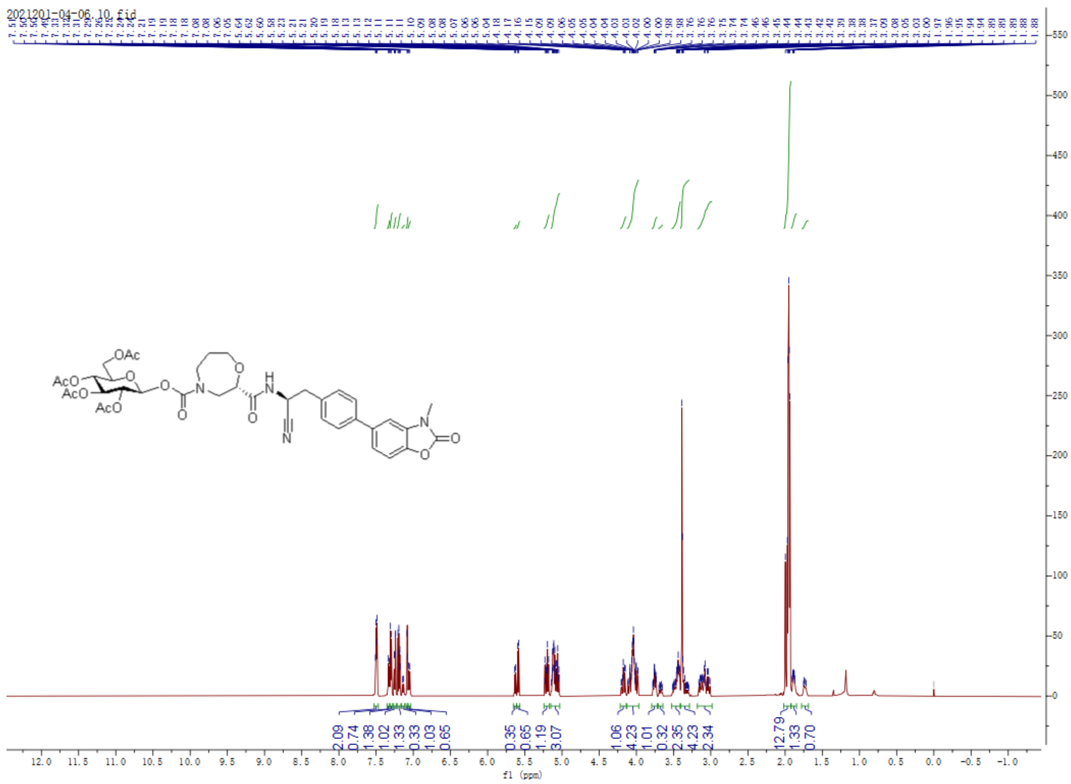

^{1}H NMR spectrum of compound **GA78** (600 MHz, $CDCl_3$)

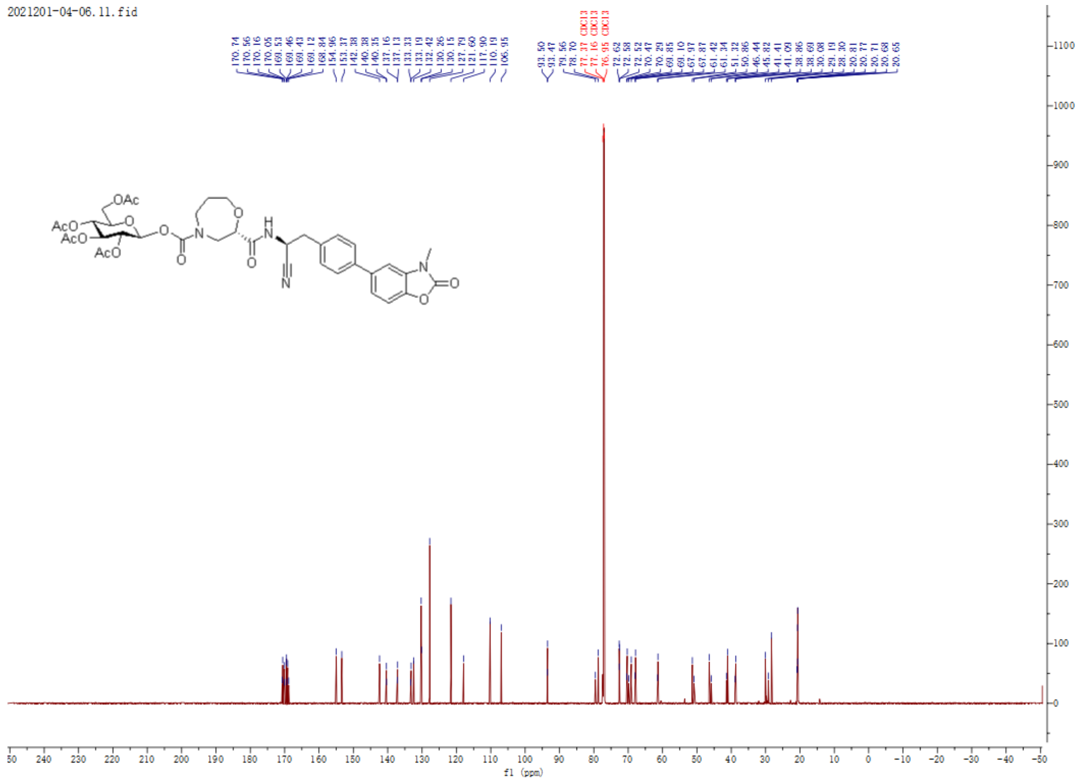

^{13}C NMR spectrum of compound **GA78** (151 MHz, $CDCl_3$)

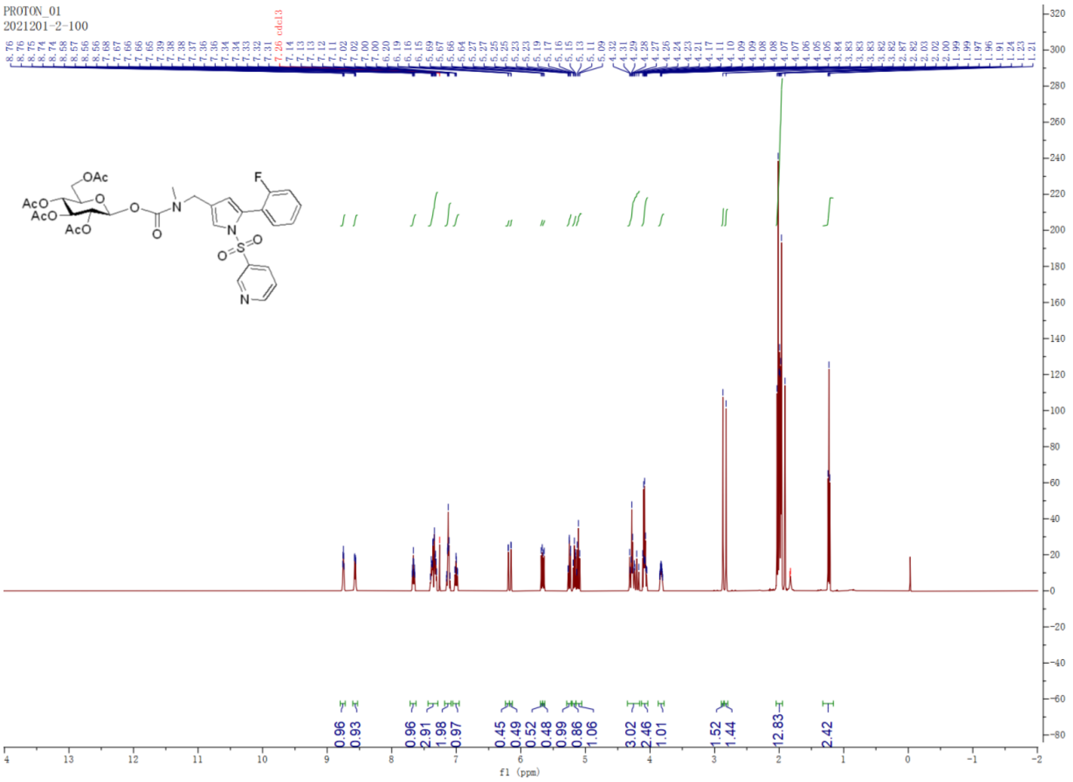

^{1}H NMR spectrum of compound **GA79** (500 MHz, $CDCl_3$)

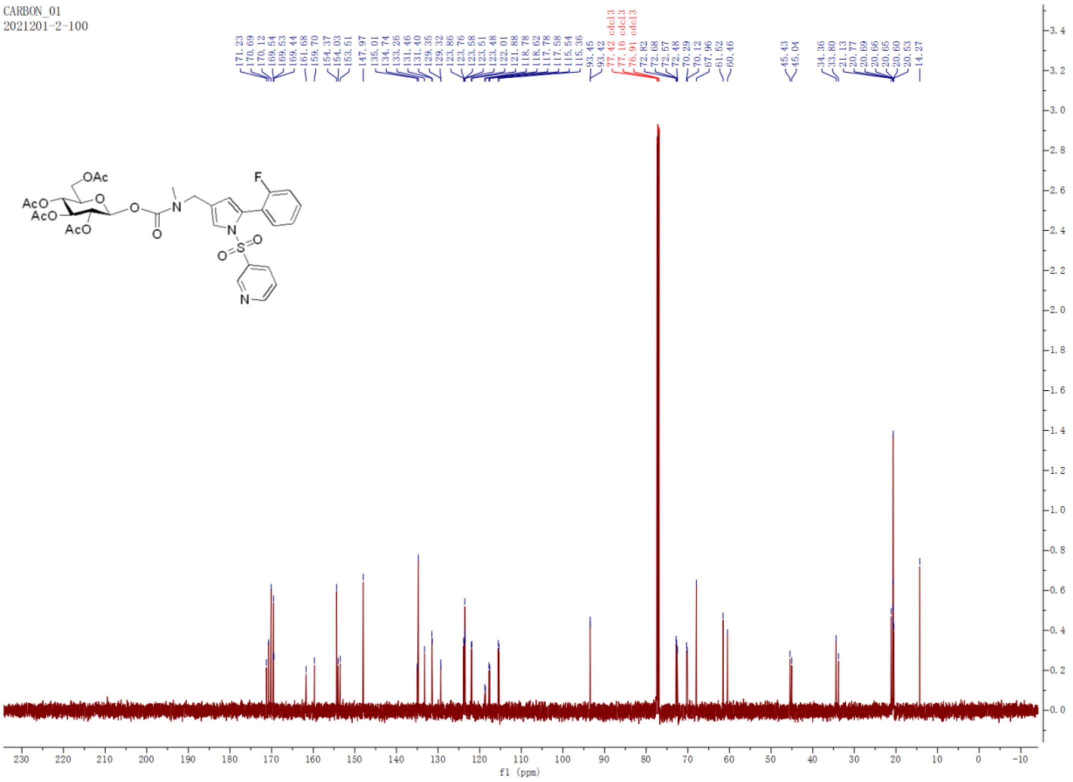

^{13}C NMR spectrum of compound **GA79** (126 MHz, $CDCl_3$)

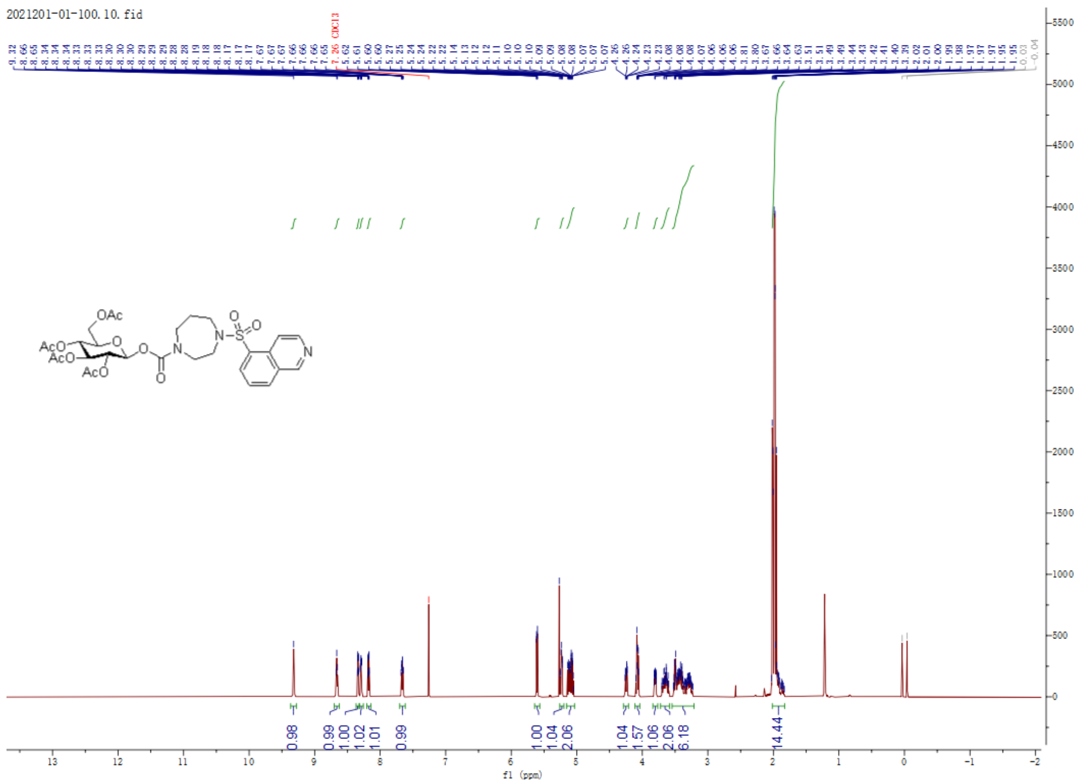

^{1}H NMR spectrum of compound **GA80** (600 MHz, $CDCl_3$)

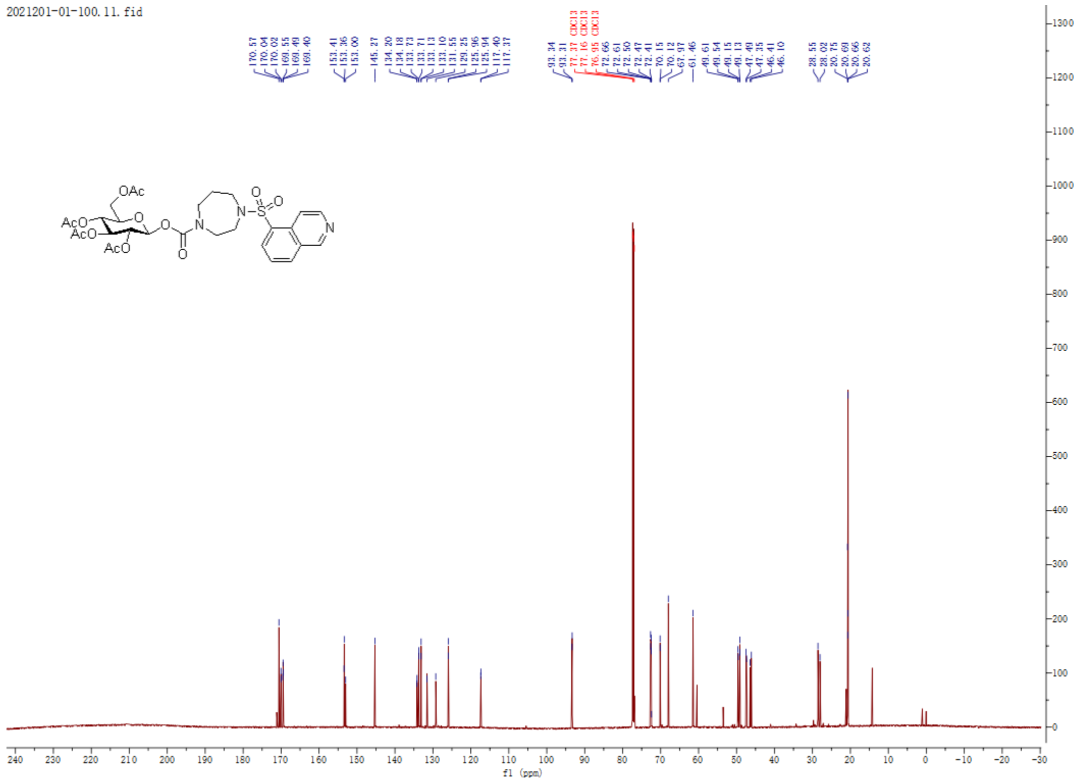

^{13}C NMR spectrum of compound **GA80** (151 MHz, $CDCl_3$)

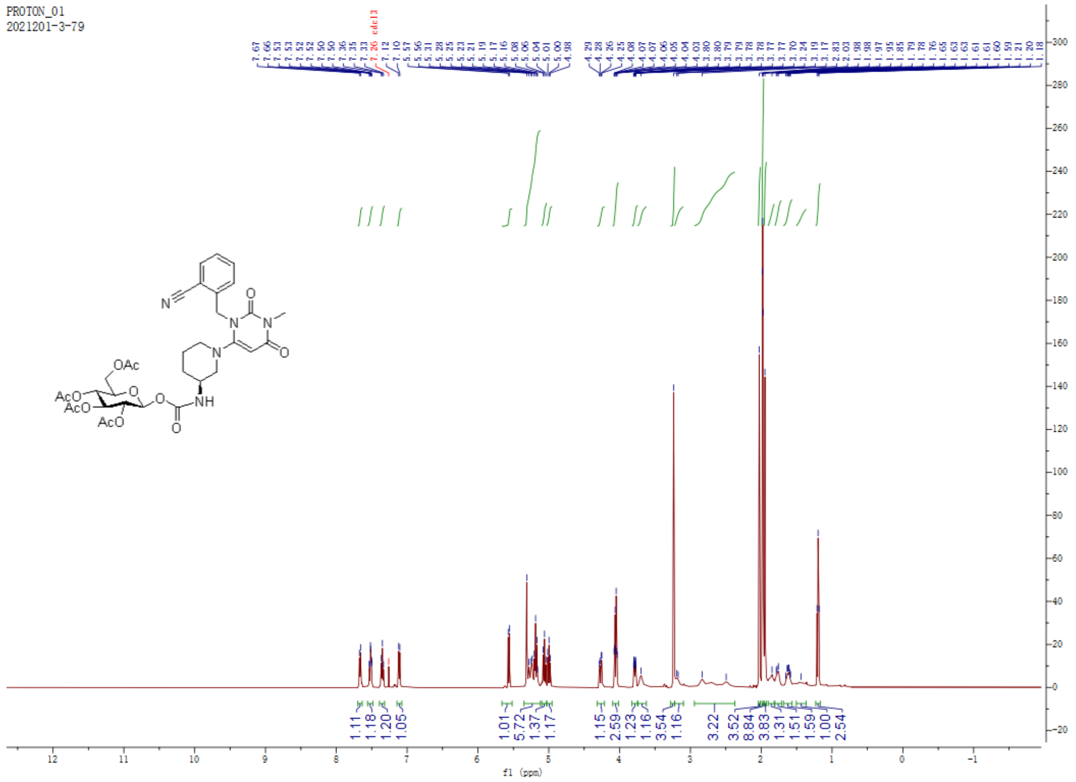

^{1}H NMR spectrum of compound **GA81** (600 MHz, $CDCl_3$)

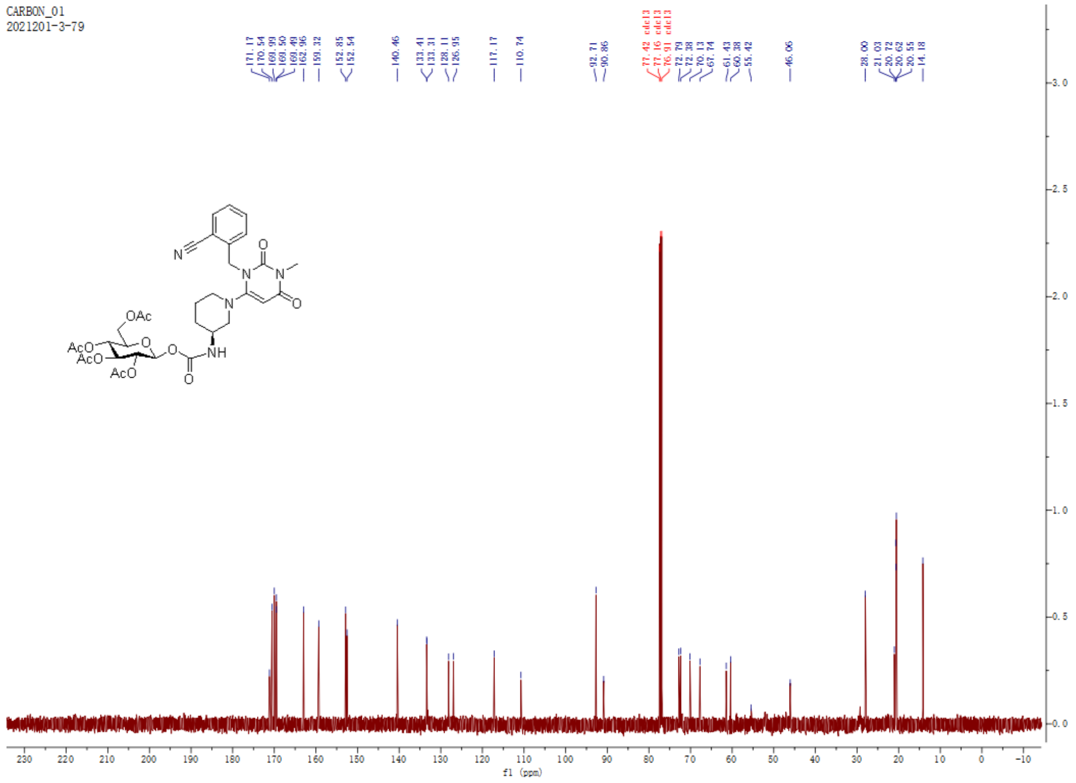

^{13}C NMR spectrum of compound **GA81** (151 MHz, $CDCl_3$)

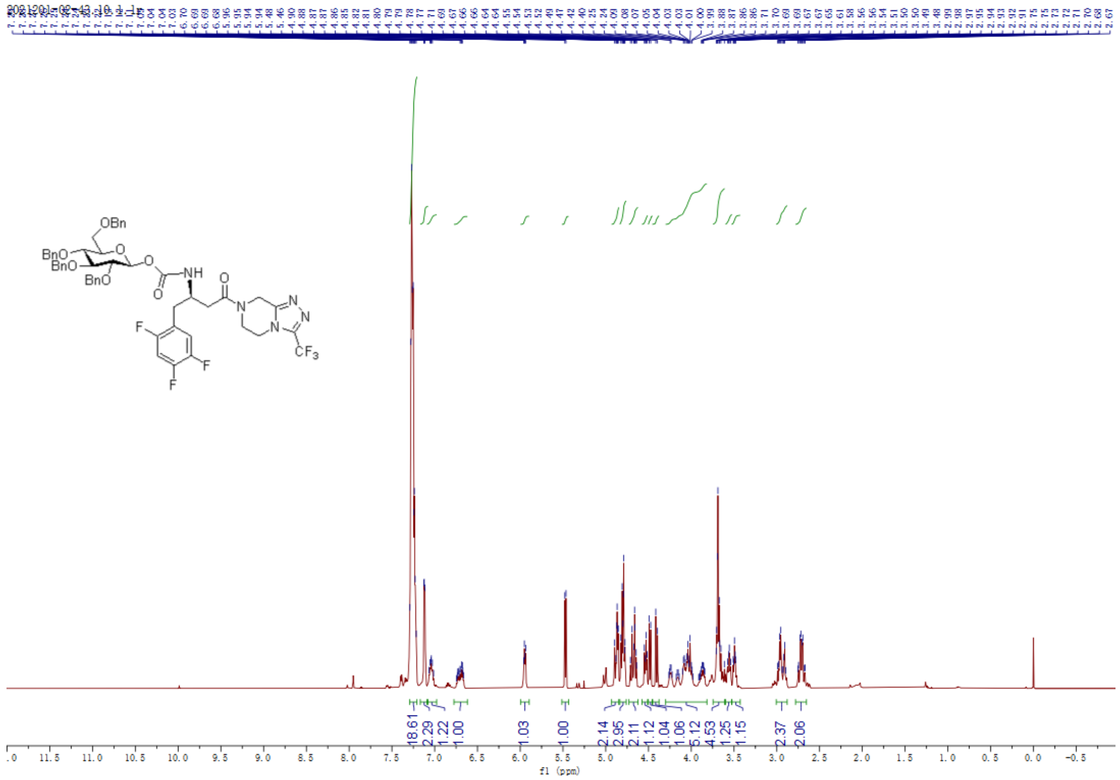

^{1}H NMR spectrum of compound **GA82** (600 MHz, $CDCl_3$)

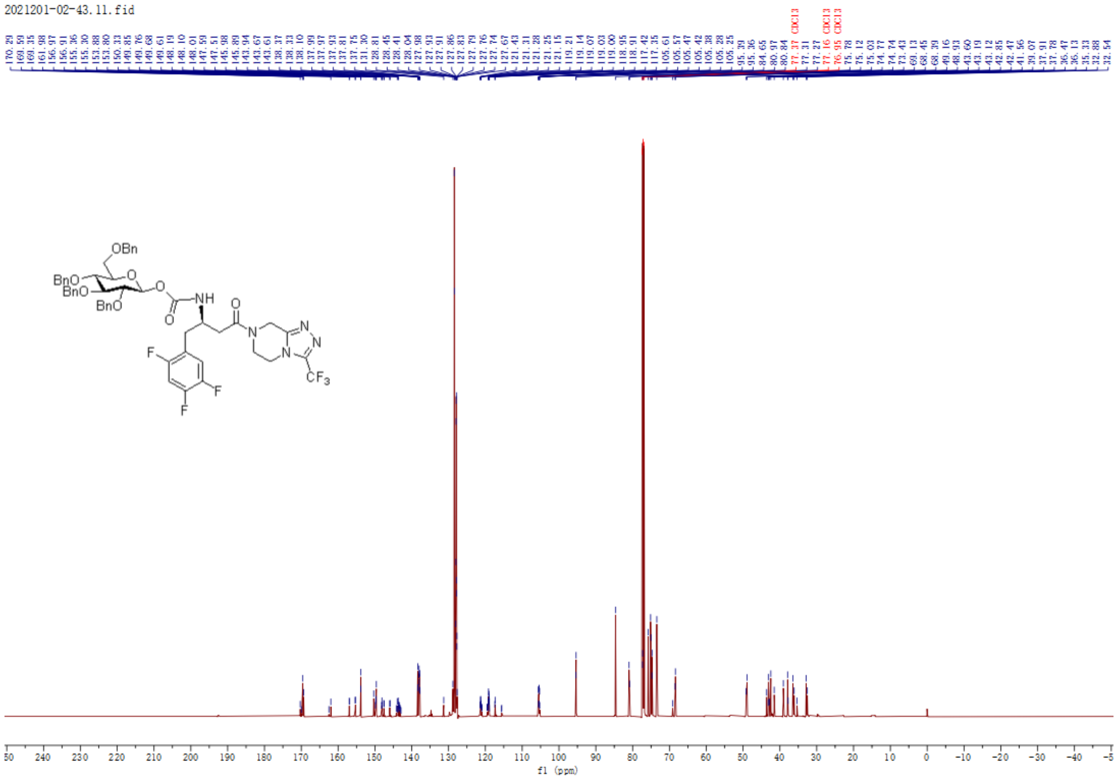

^{13}C NMR spectrum of compound **GA82** (151 MHz, $CDCl_3$)

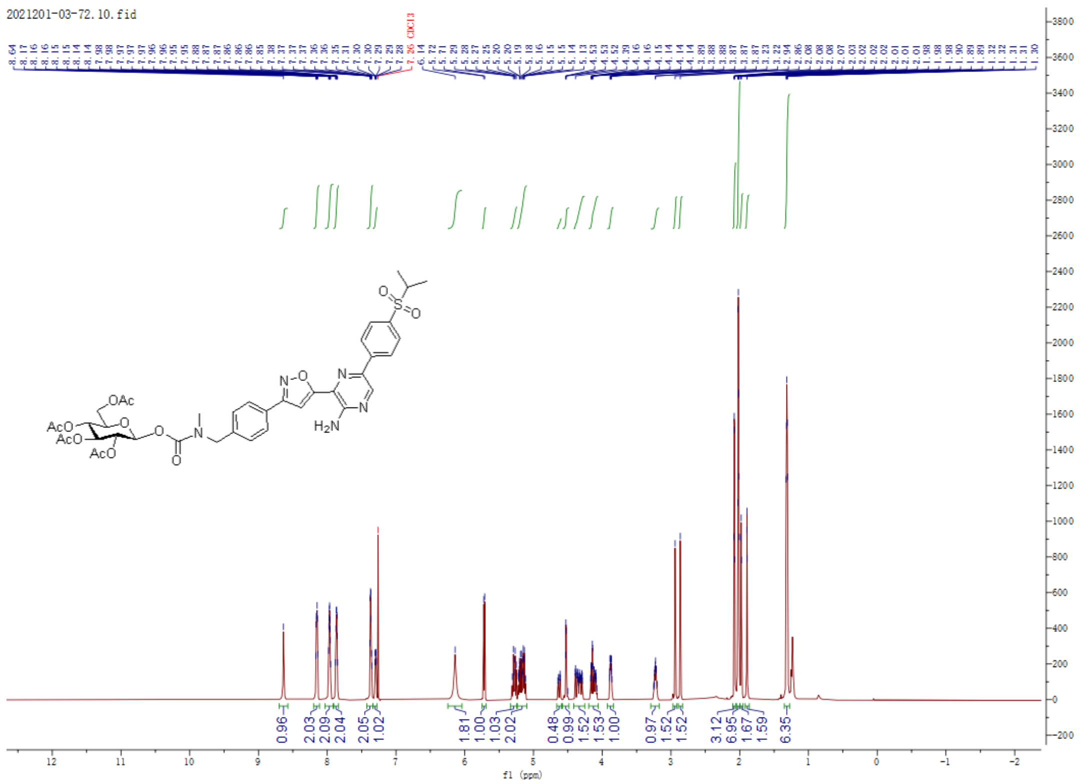

^{1}H NMR spectrum of compound **GA83** (600 MHz, $CDCl_3$)

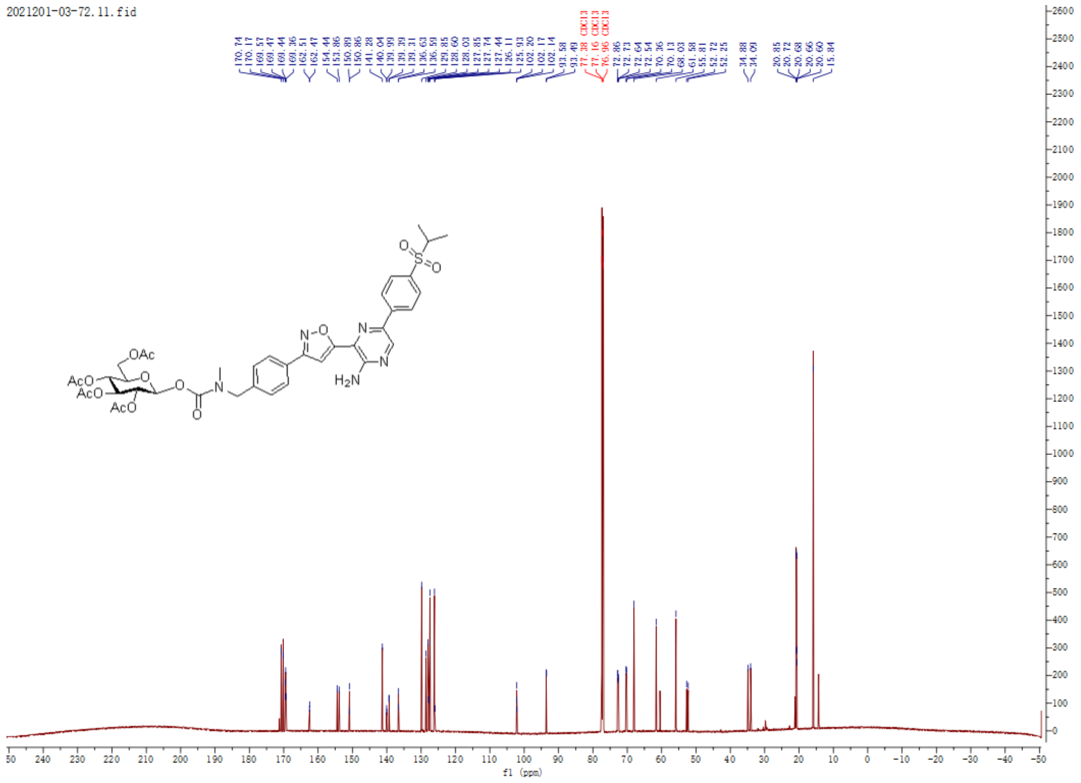

^{13}C NMR spectrum of compound **GA83** (151 MHz, $CDCl_3$)

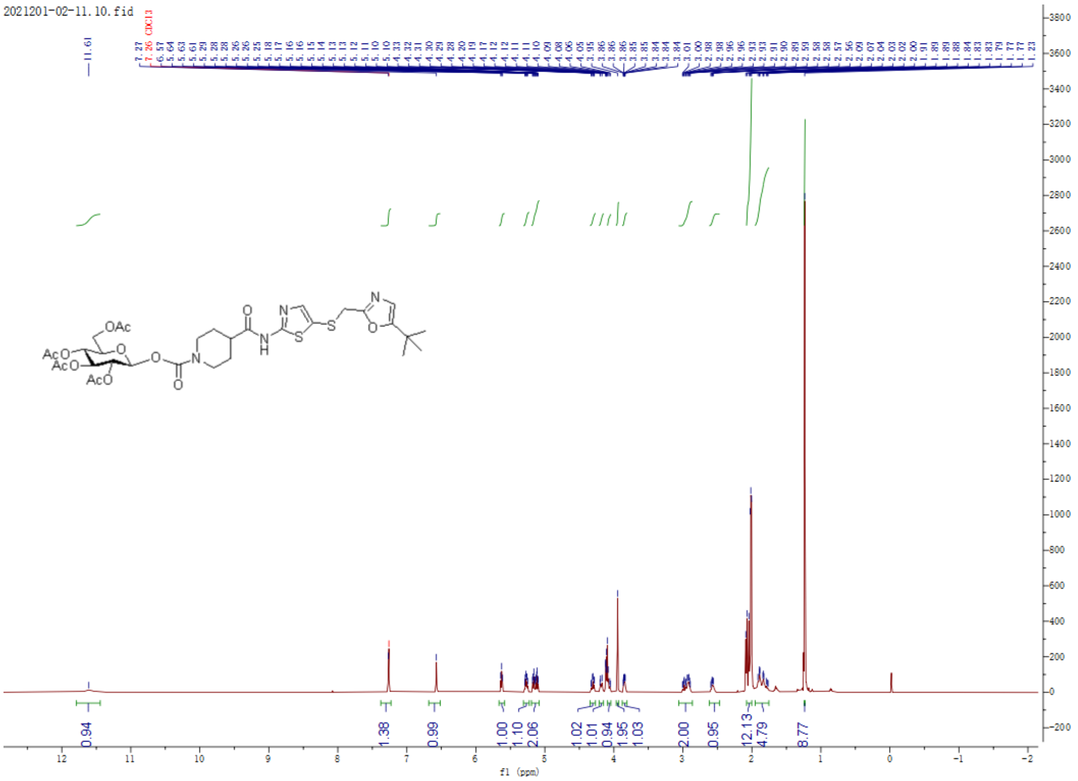

^{1}H NMR spectrum of compound **GA84** (600 MHz, $CDCl_3$)

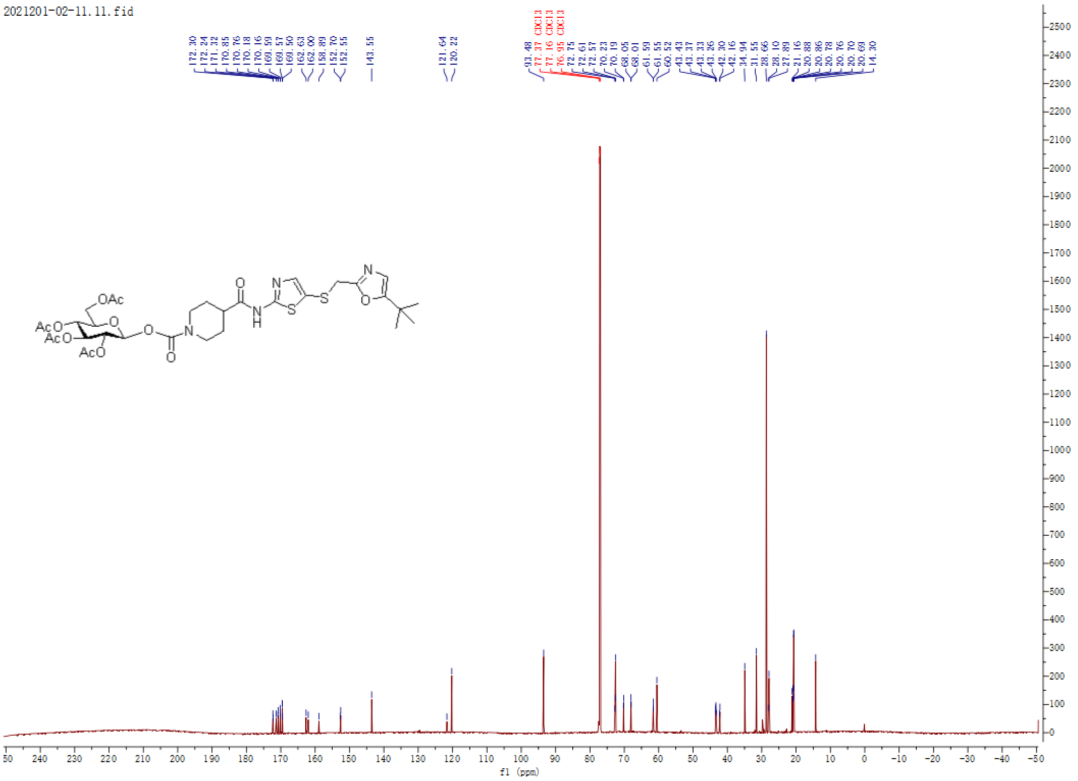

^{13}C NMR spectrum of compound **GA84** (151 MHz, $CDCl_3$)

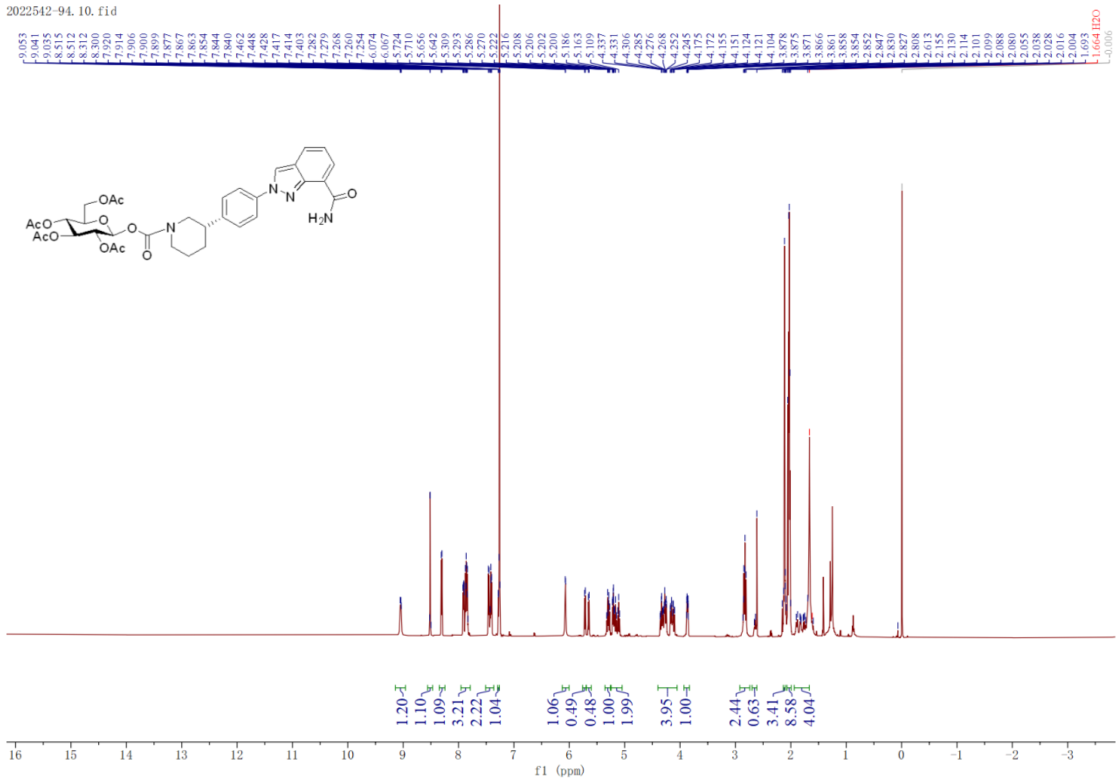

^{1}H NMR spectrum of compound **GA85** (600 MHz, $CDCl_3$)

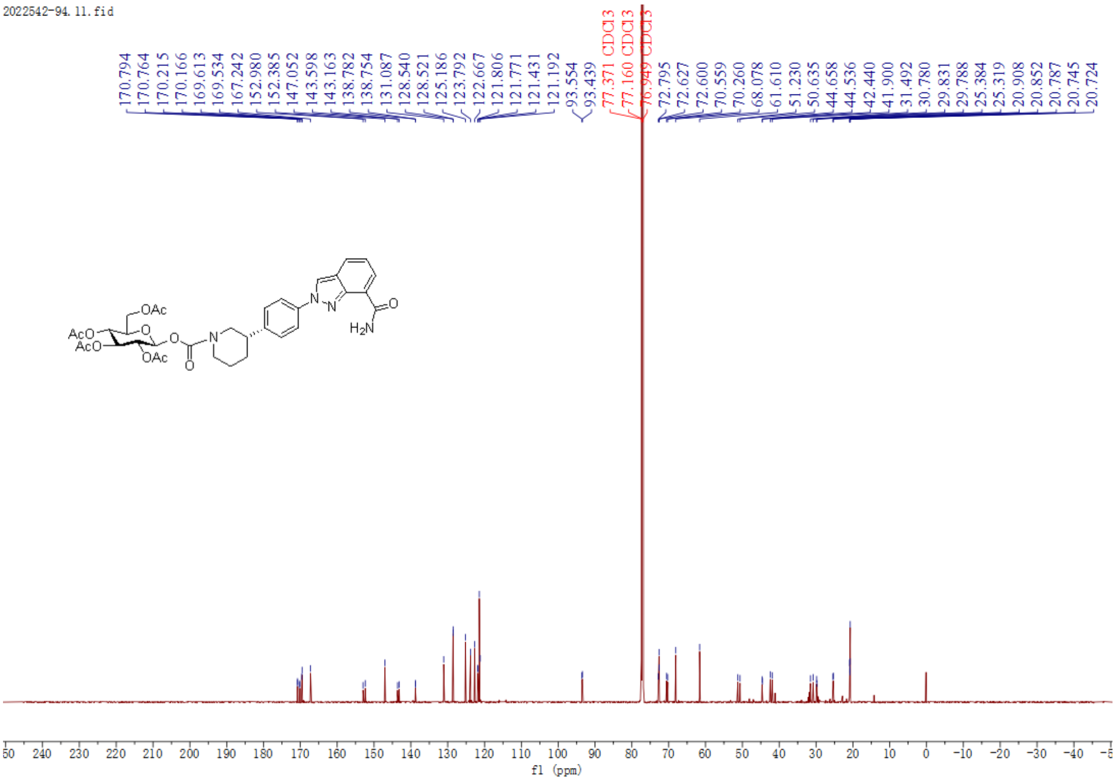

^{13}C NMR spectrum of compound **GA85** (151 MHz, $CDCl_3$)

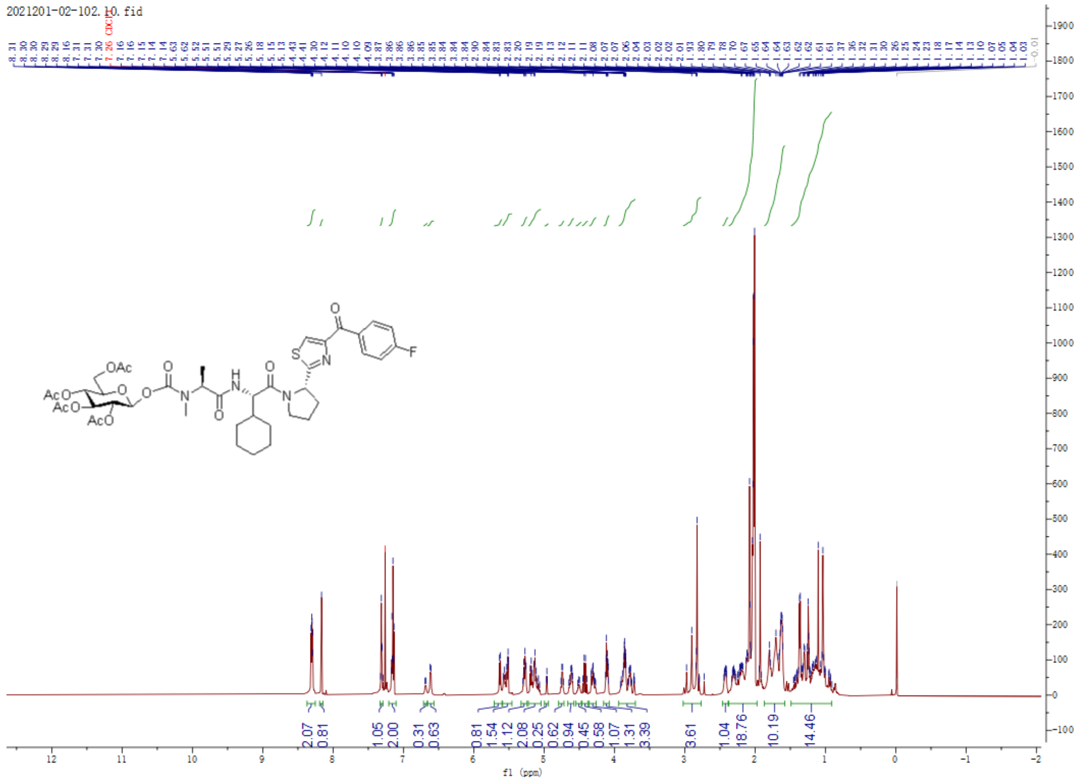

^{1}H NMR spectrum of compound **GA86** (600 MHz, $CDCl_3$)

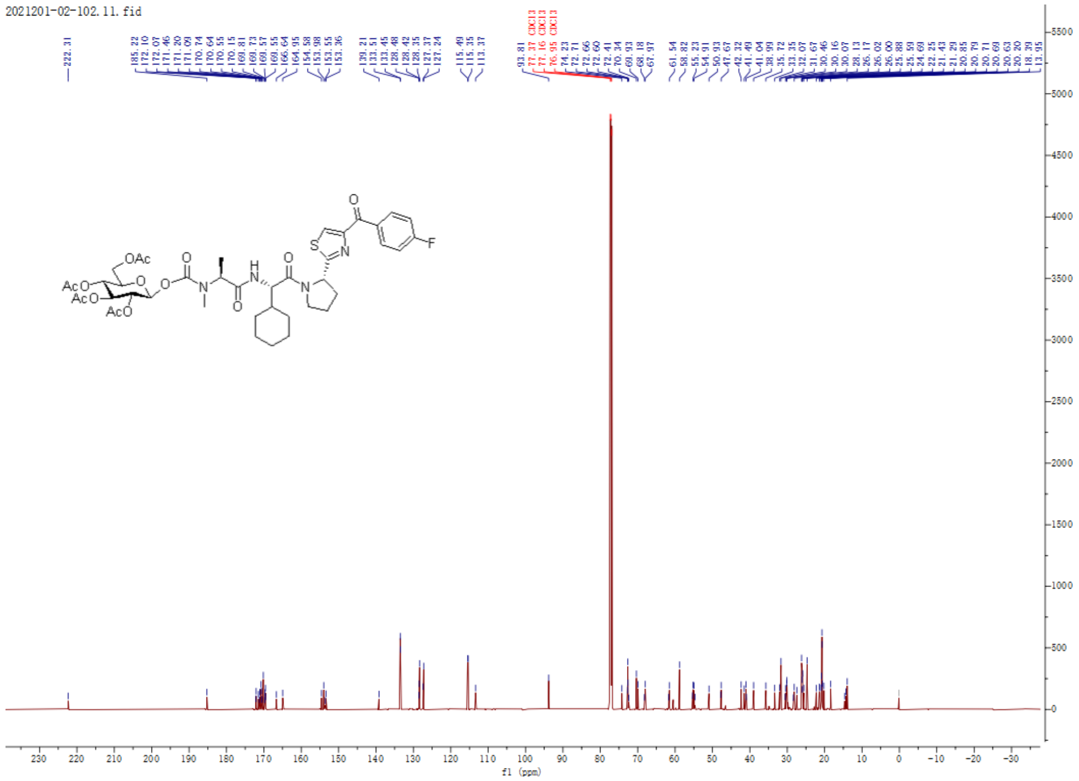

^{13}C NMR spectrum of compound **GA86** (151 MHz, $CDCl_3$)

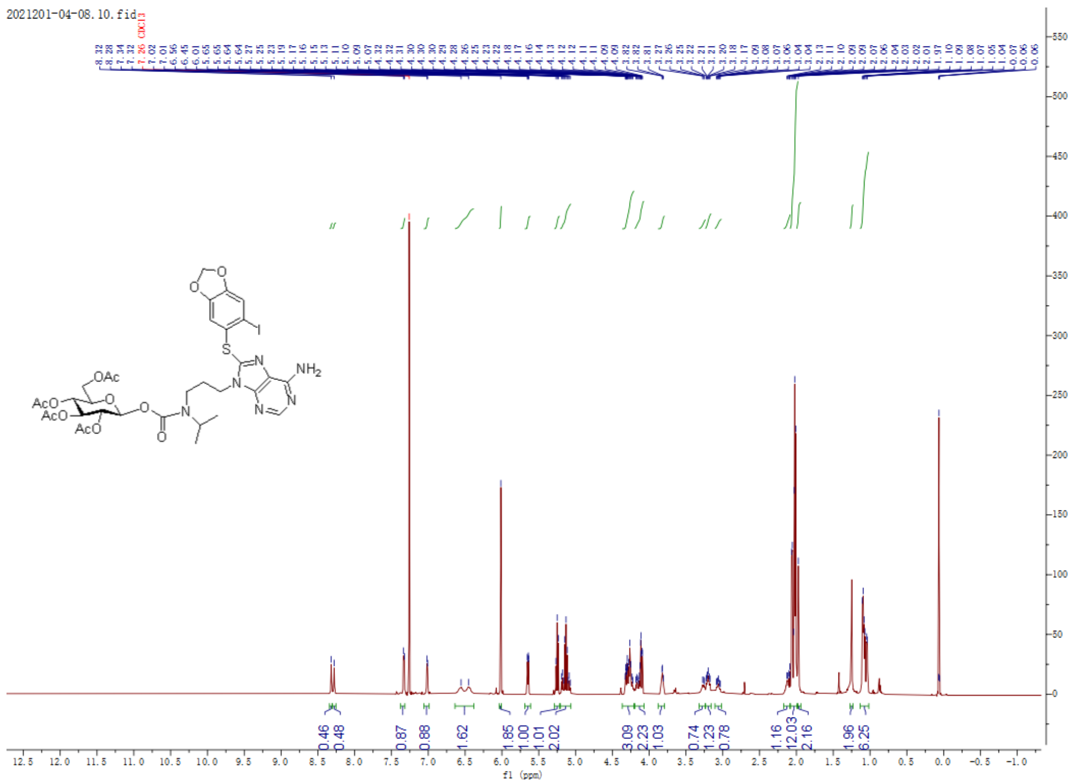

^{1}H NMR spectrum of compound **GA87** (600 MHz, $CDCl_3$)

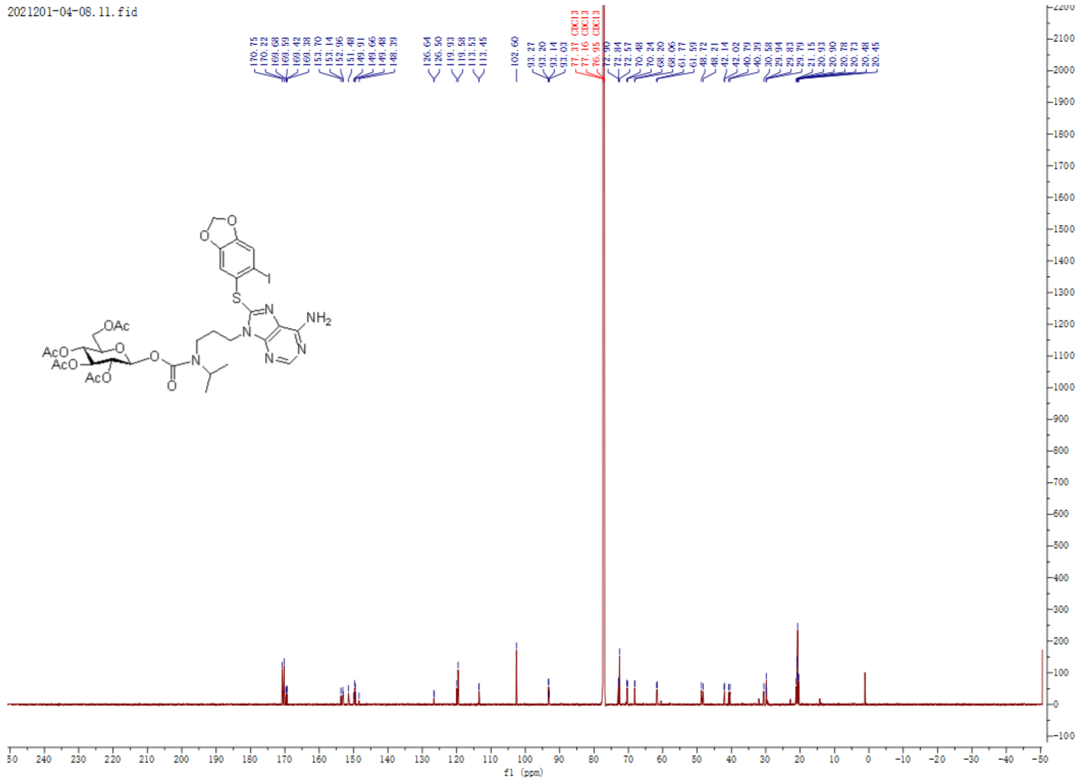

^{13}C NMR spectrum of compound **GA87** (151 MHz, $CDCl_3$)

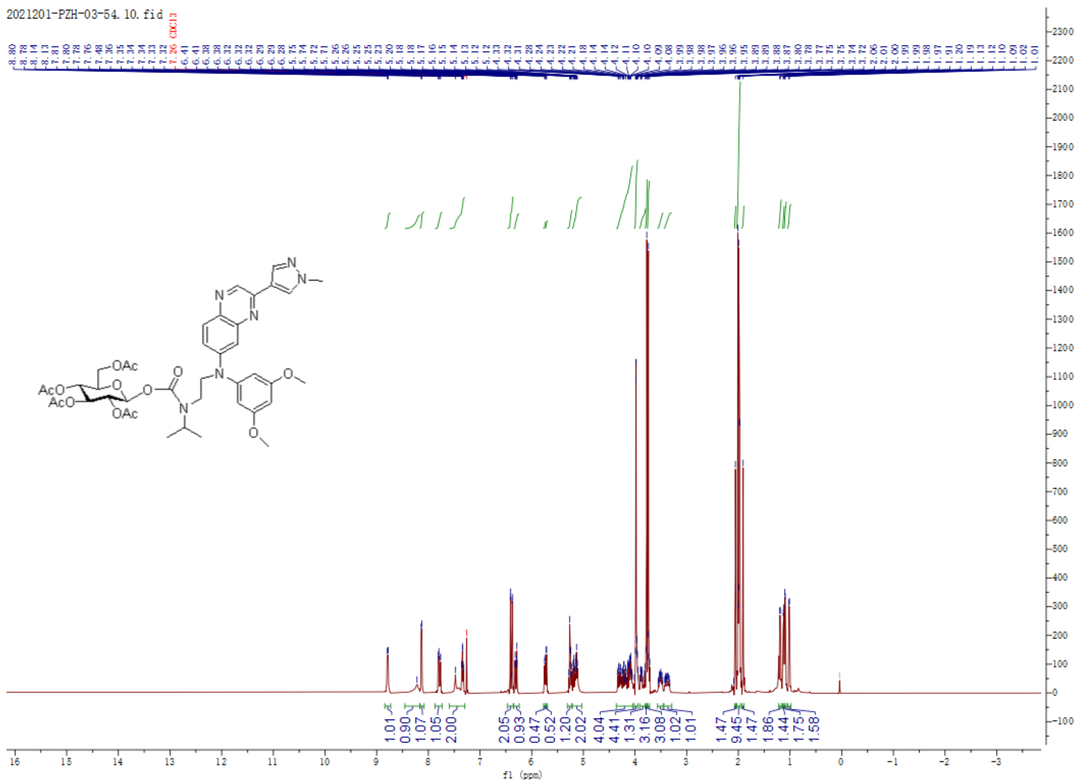

^{1}H NMR spectrum of compound **GA88** (600 MHz, $CDCl_3$)

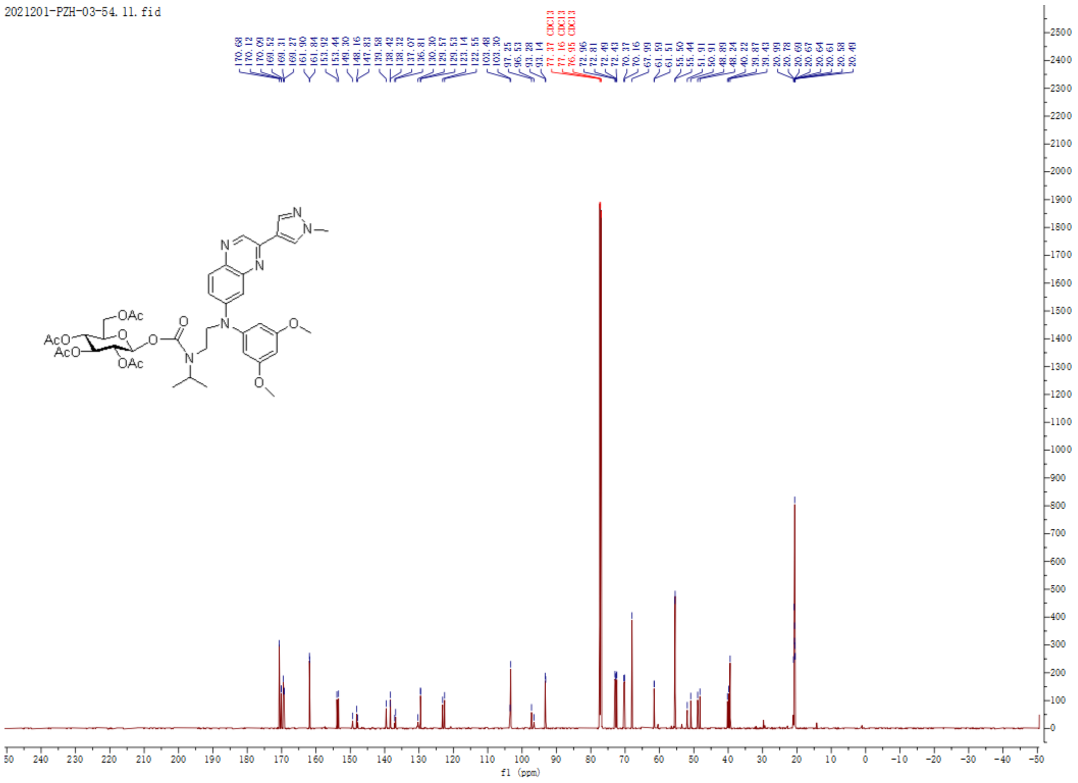

^{13}C NMR spectrum of compound **GA88** (151 MHz, $CDCl_3$)

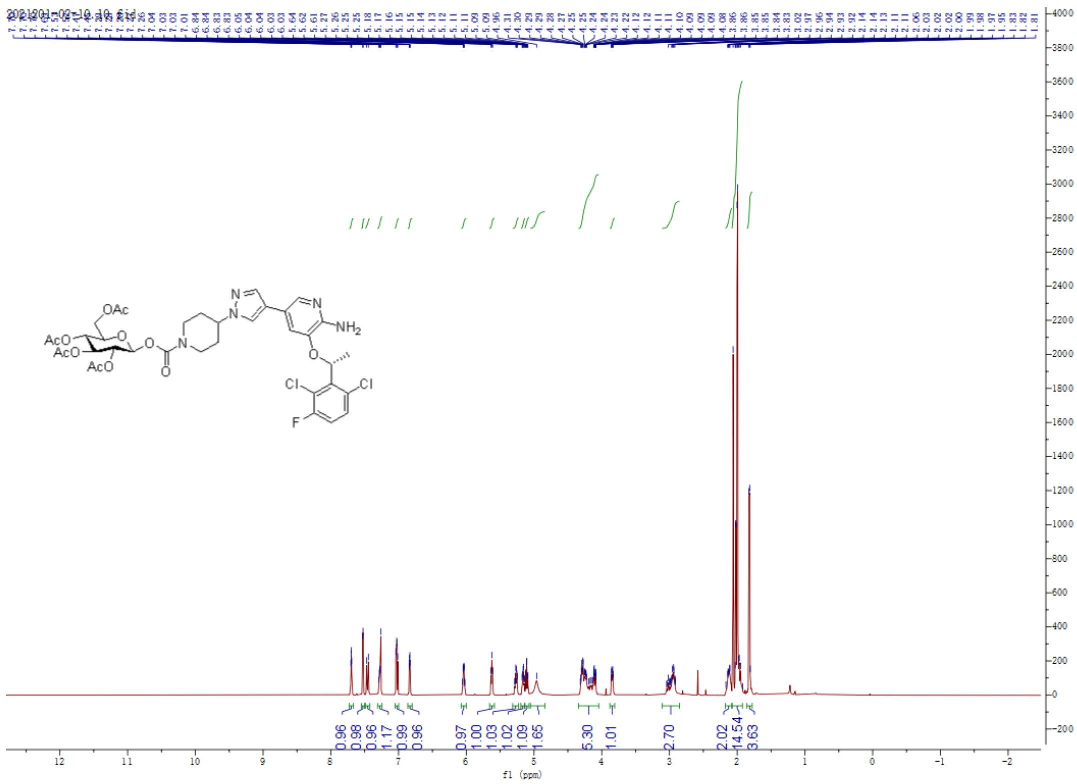

^{1}H NMR spectrum of compound **GA89** (600 MHz, $CDCl_3$)

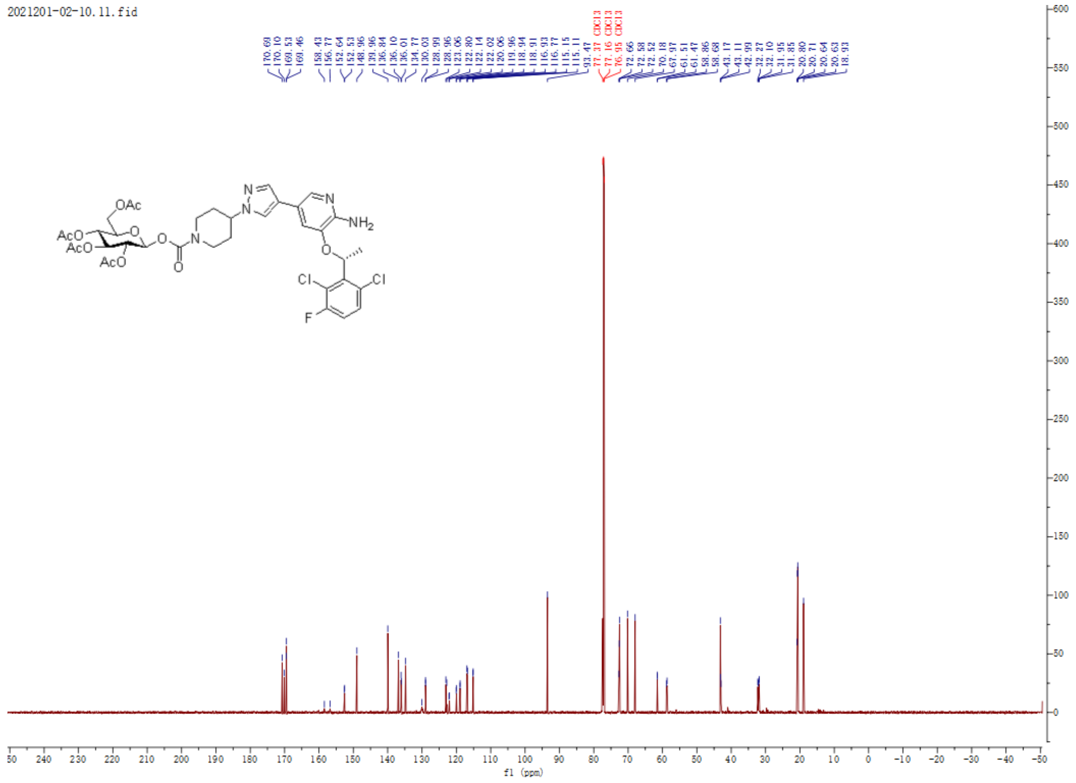

^{13}C NMR spectrum of compound **GA89** (151 MHz, $CDCl_3$)

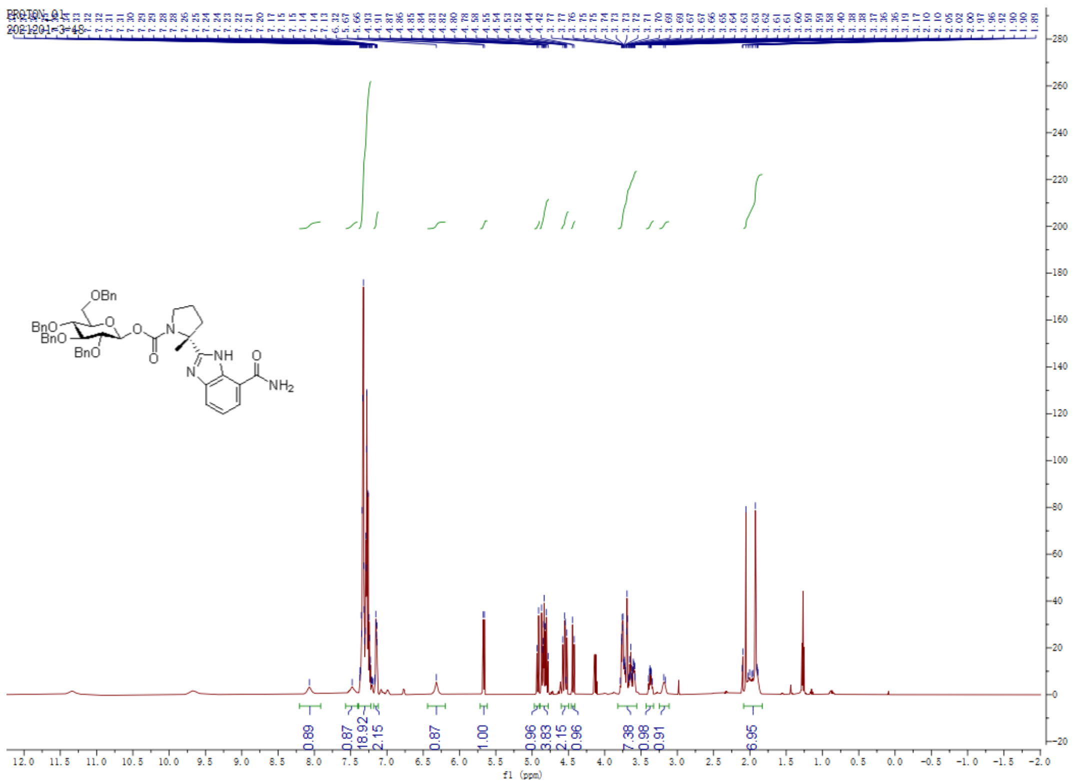

^{1}H NMR spectrum of compound **GA90** (600 MHz, $CDCl_3$)

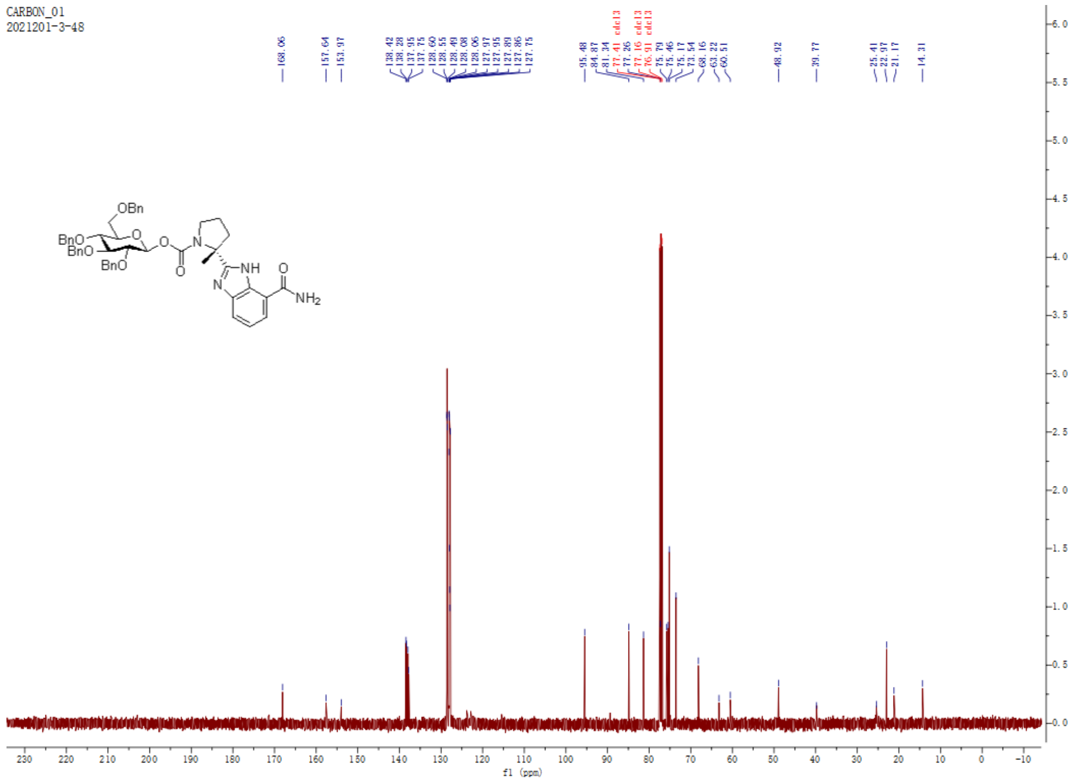

^{13}C NMR spectrum of compound **GA90** (151 MHz, $CDCl_3$)

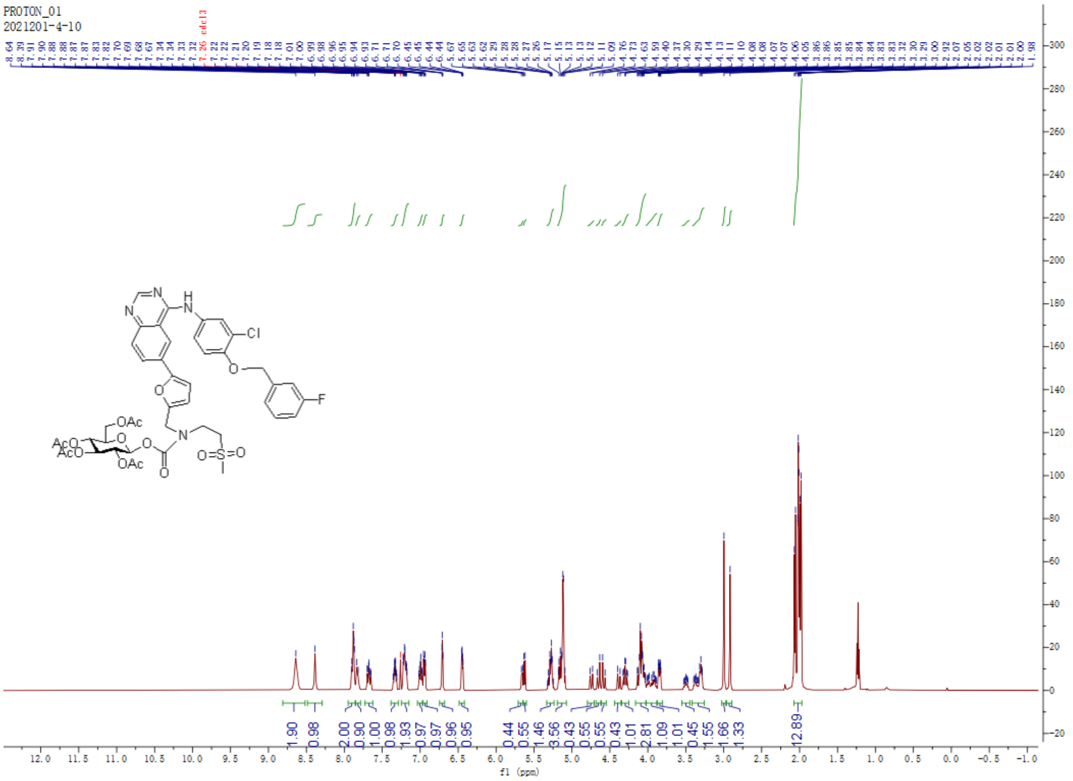

^{1}H NMR spectrum of compound **GA91** (500 MHz, $CDCl_3$)

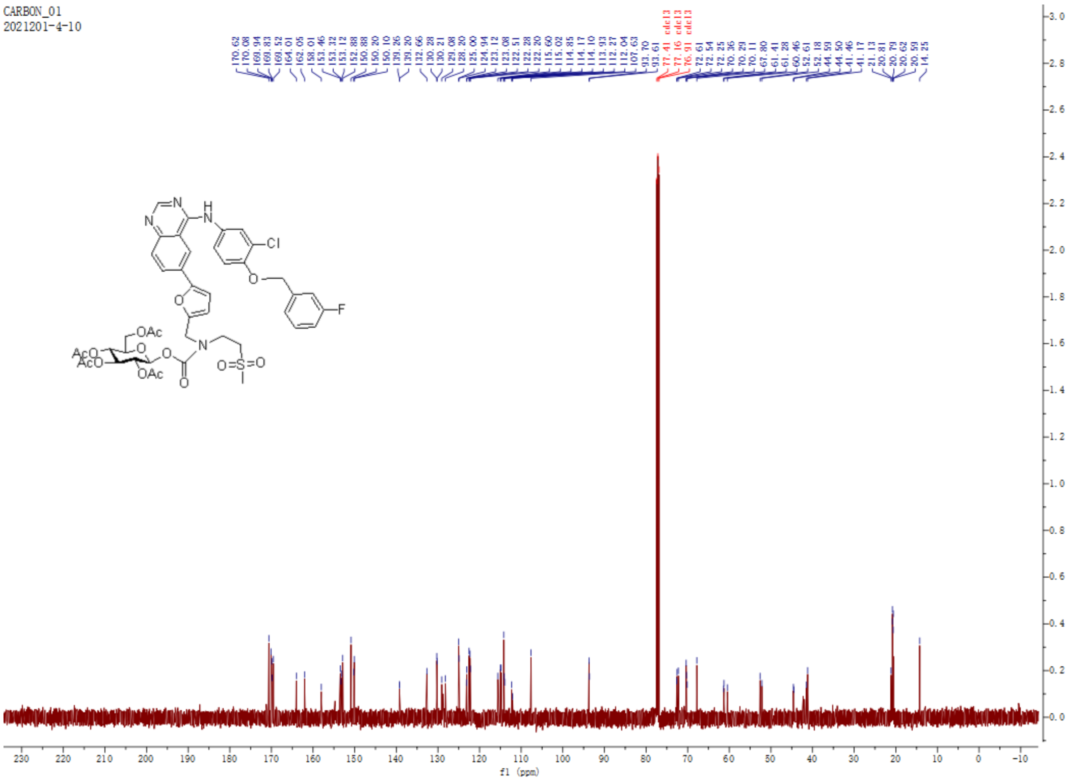

^{13}C NMR spectrum of compound **GA91** (126 MHz, $CDCl_3$)

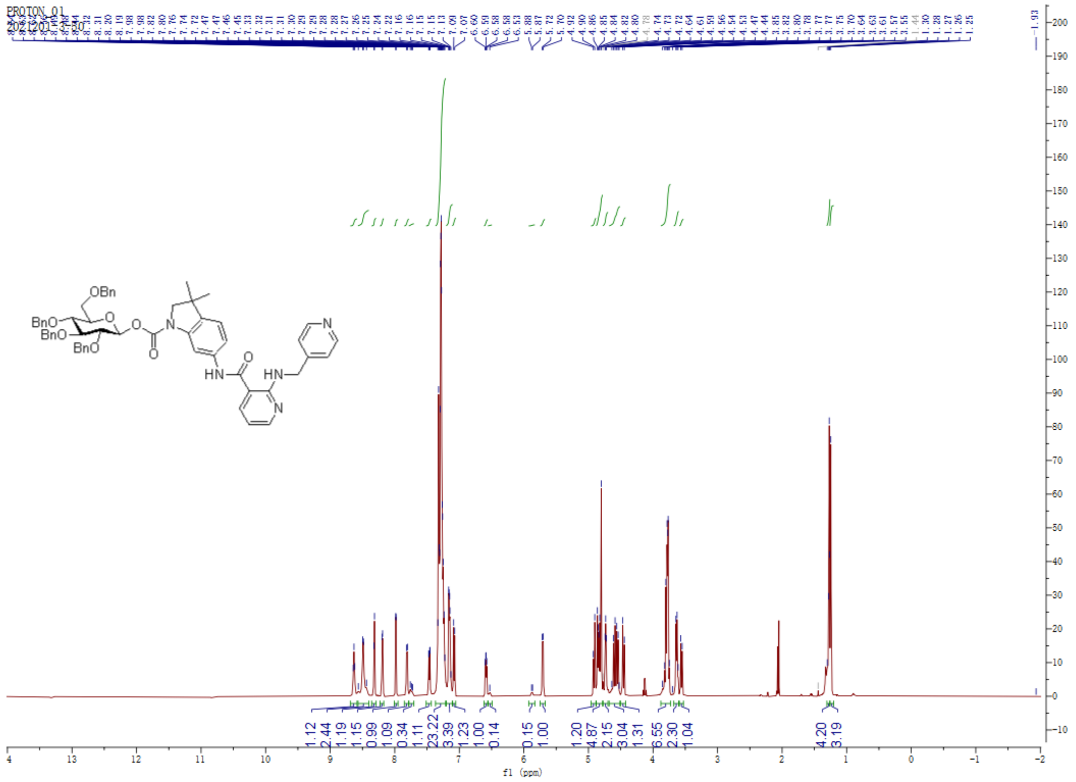

^{1}H NMR spectrum of compound **GA92** (500 MHz, $CDCl_3$)

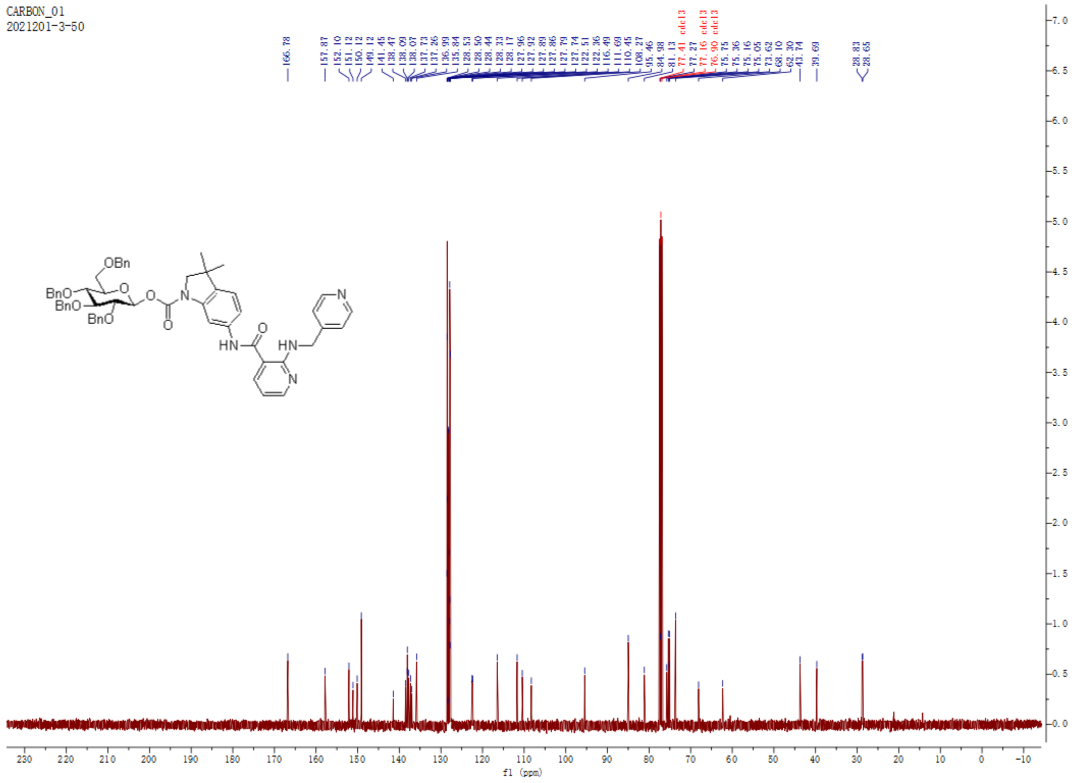

^{13}C NMR spectrum of compound **GA92** (126 MHz, $CDCl_3$)

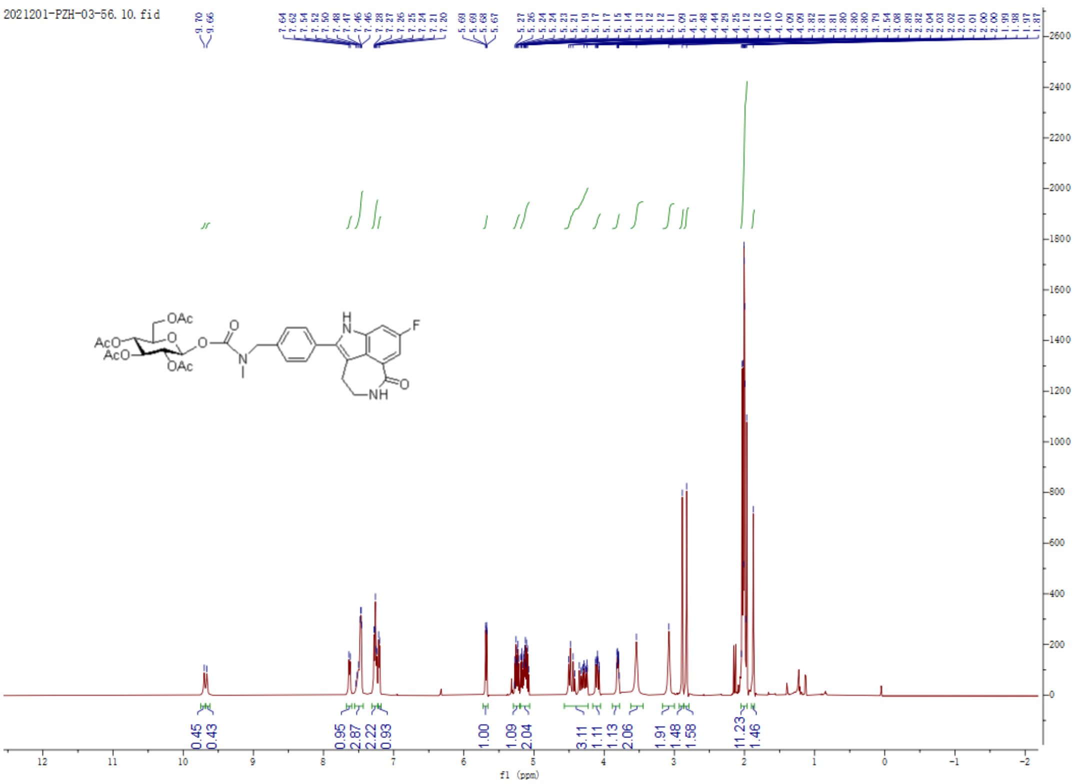

^{1}H NMR spectrum of compound **GA93** (600 MHz, $CDCl_3$)

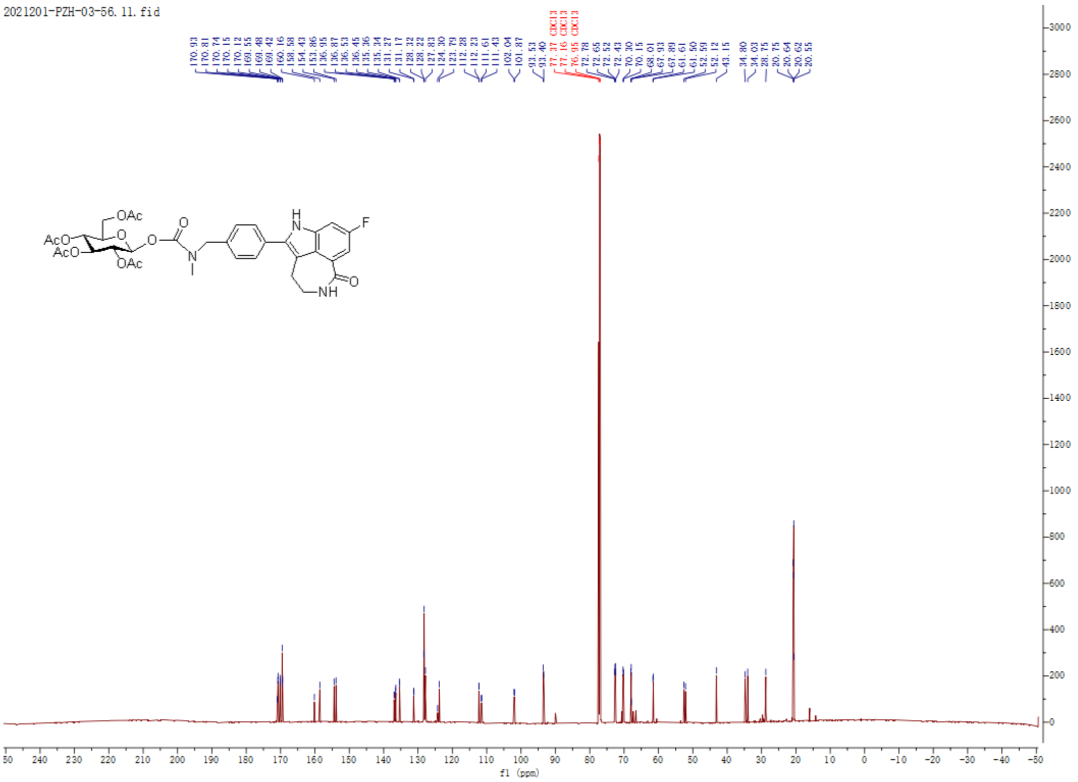

^{13}C NMR spectrum of compound **GA93** (151 MHz, $CDCl_3$)

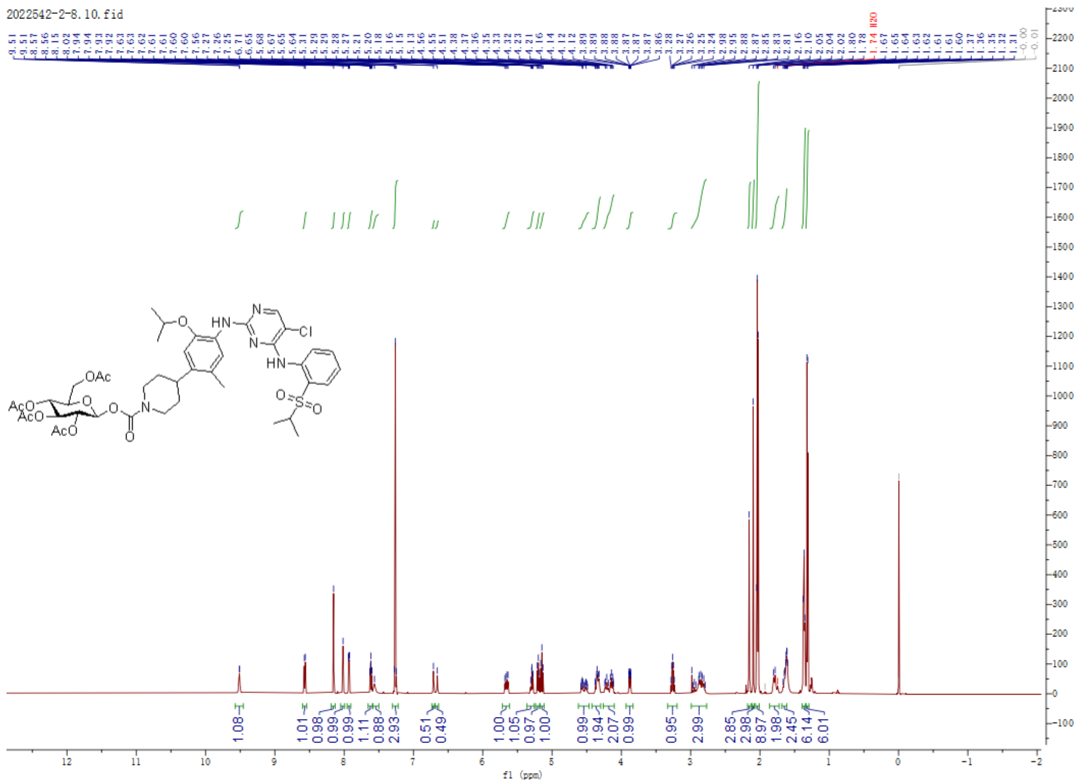

^{1}H NMR spectrum of compound **GA94** (500 MHz, $CDCl_3$)

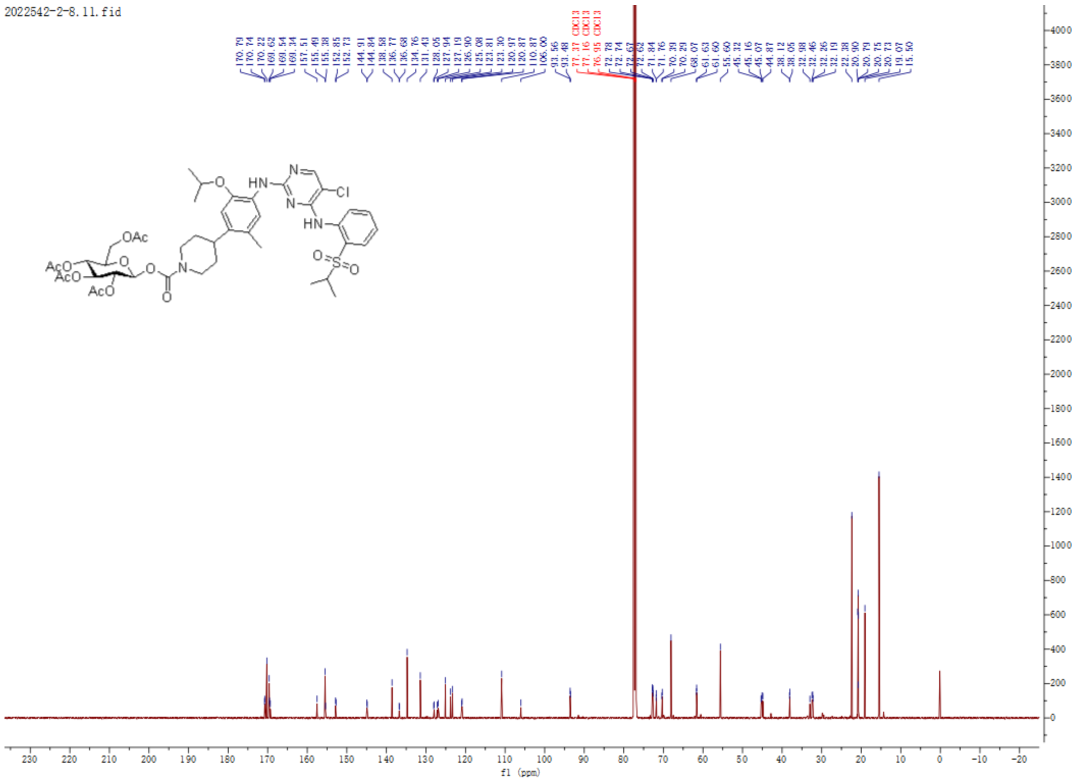

^{13}C NMR spectrum of compound **GA94** (126 MHz, $CDCl_3$)

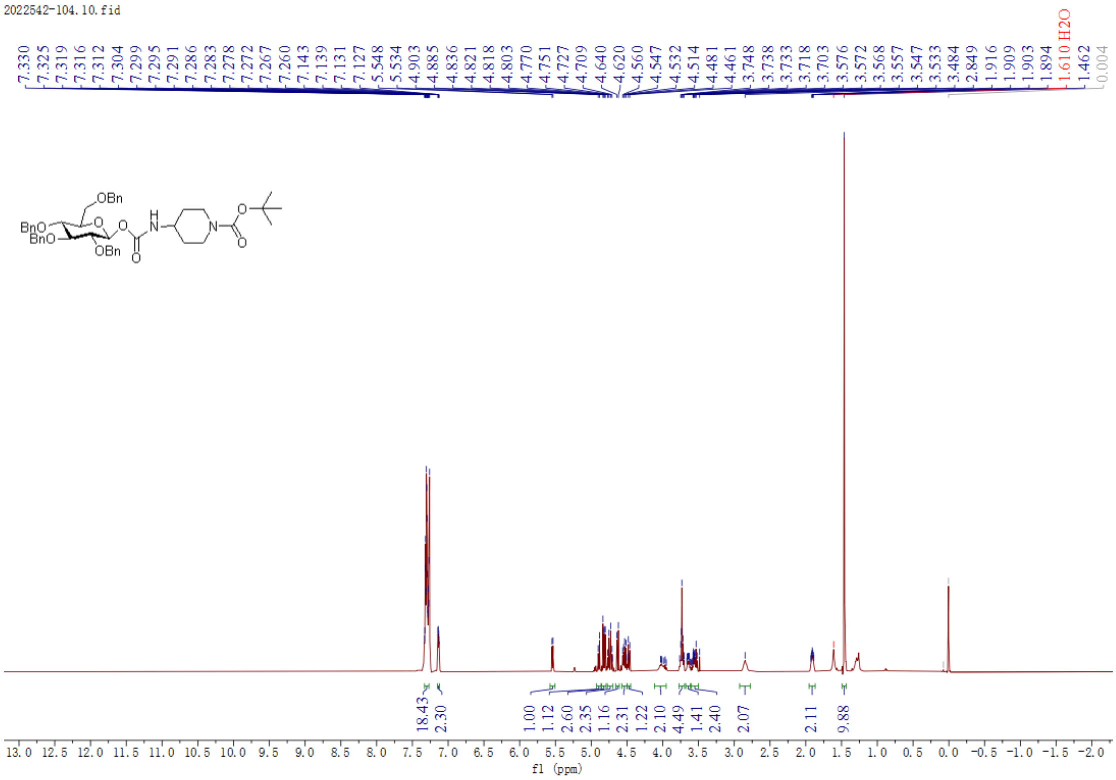

^{1}H NMR spectrum of compound **GA96** (600 MHz, $CDCl_3$)

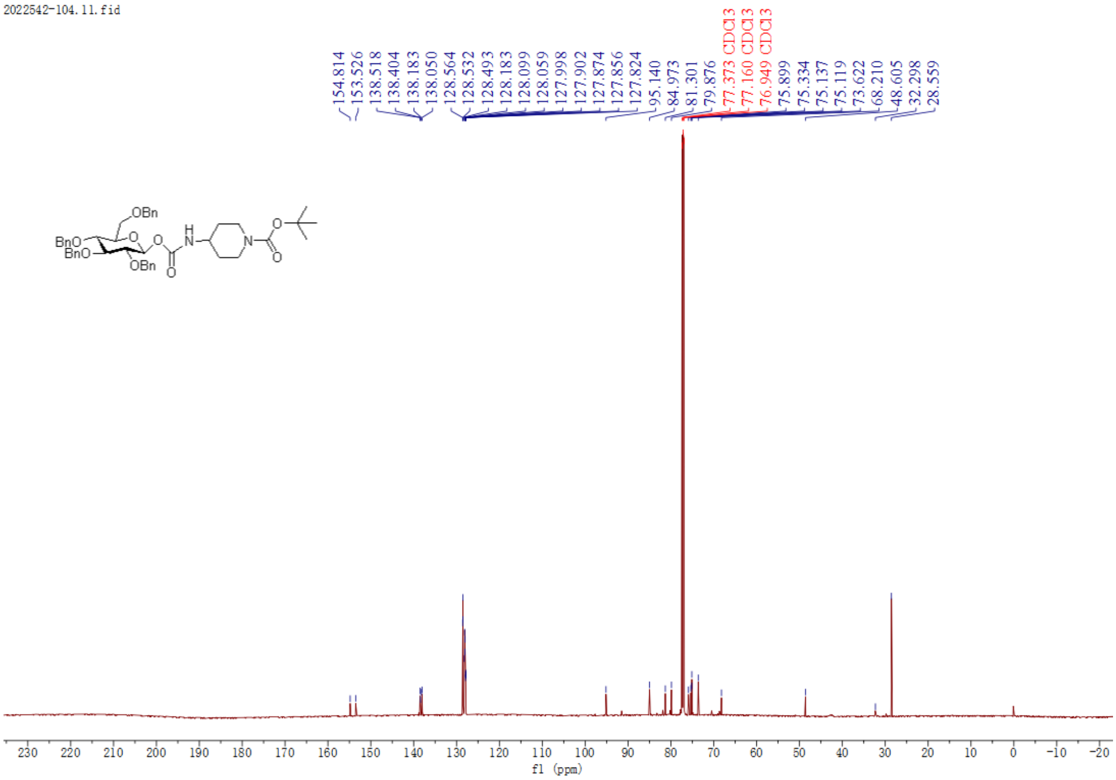

^{13}C NMR spectrum of compound **GA96** (151 MHz, $CDCl_3$)

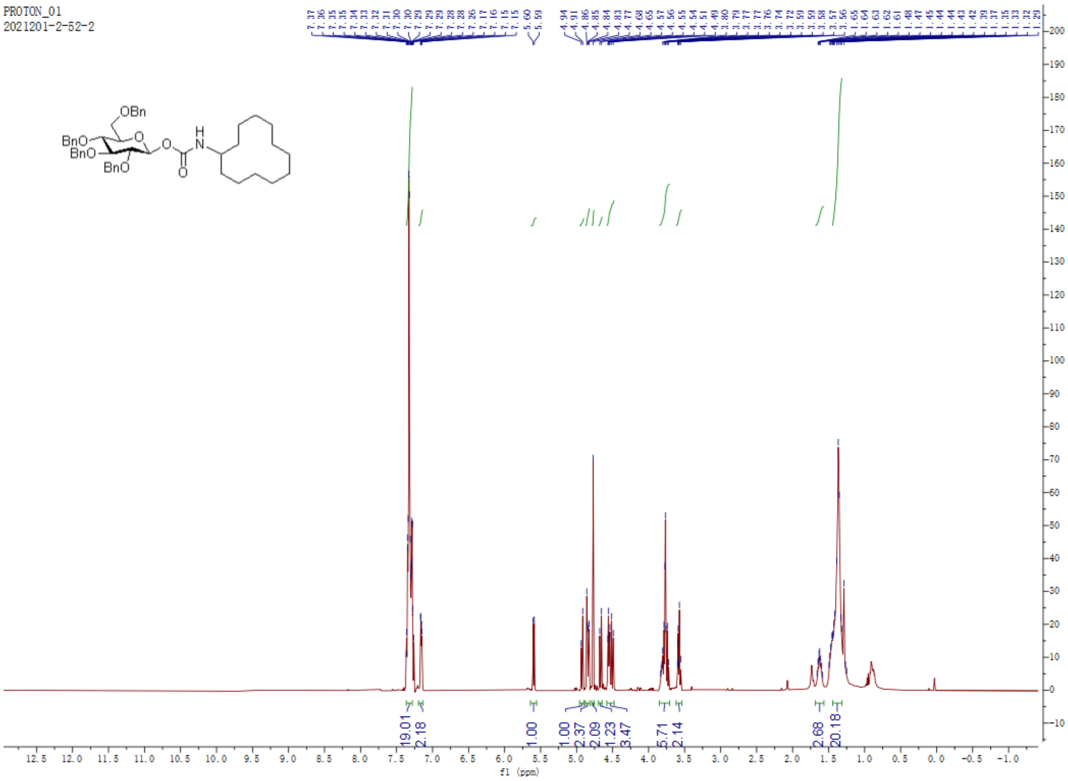

^{1}H NMR spectrum of compound **GA97** (500 MHz, $CDCl_3$)

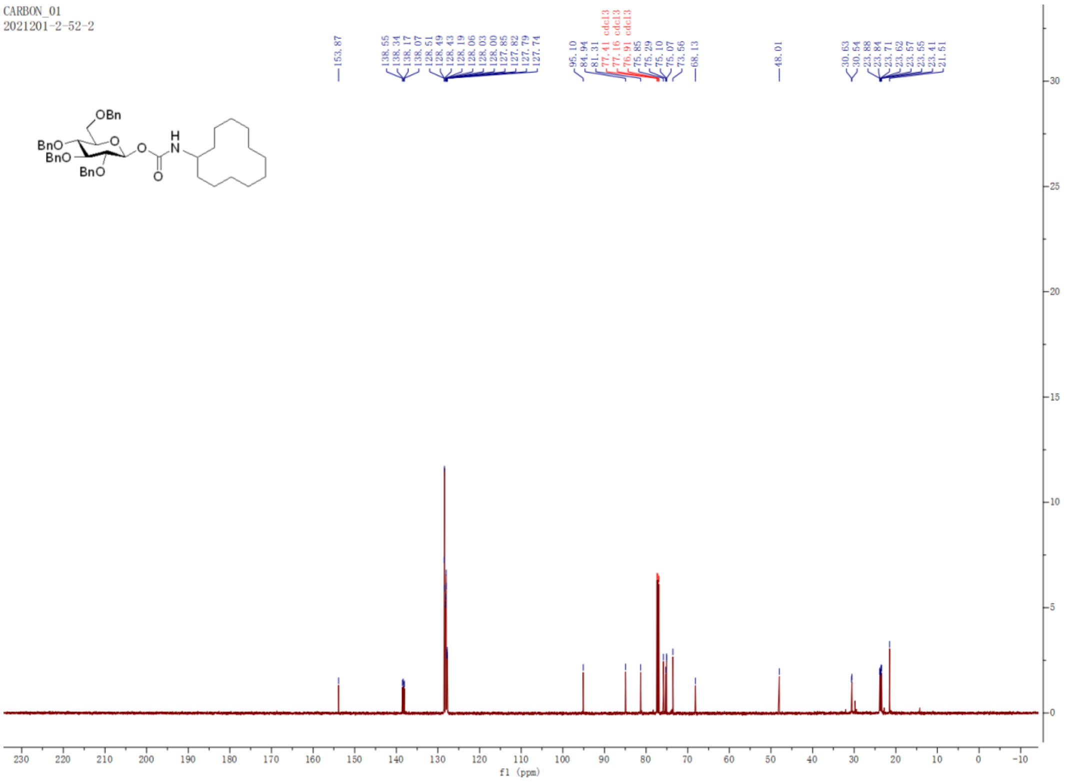

^{13}C NMR spectrum of compound **GA97** (126 MHz, $CDCl_3$)

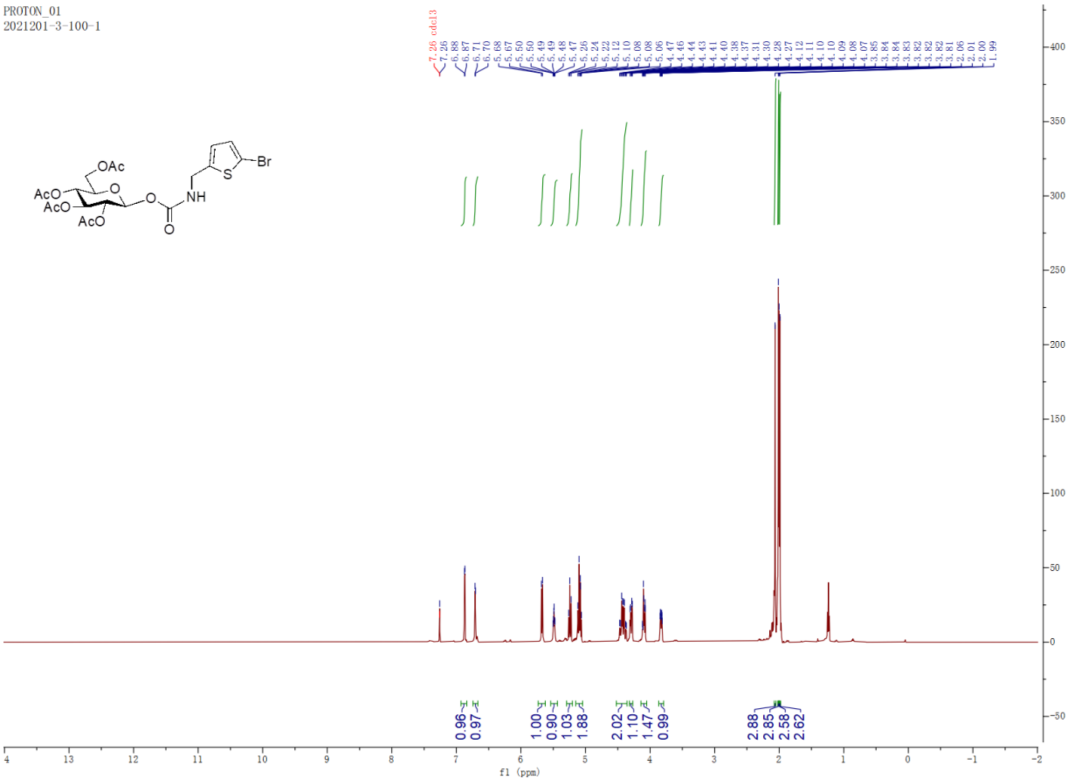

^{1}H NMR spectrum of compound **GA98** (500 MHz, $CDCl_3$)

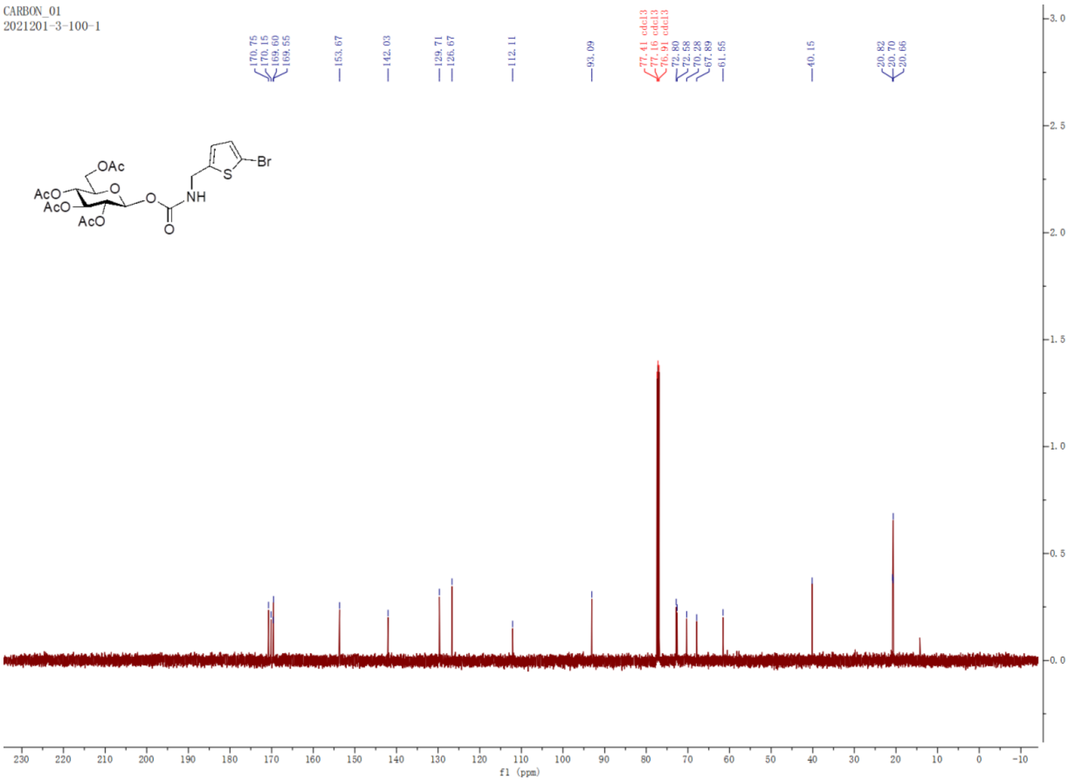

^{13}C NMR spectrum of compound **GA98** (126 MHz, $CDCl_3$)

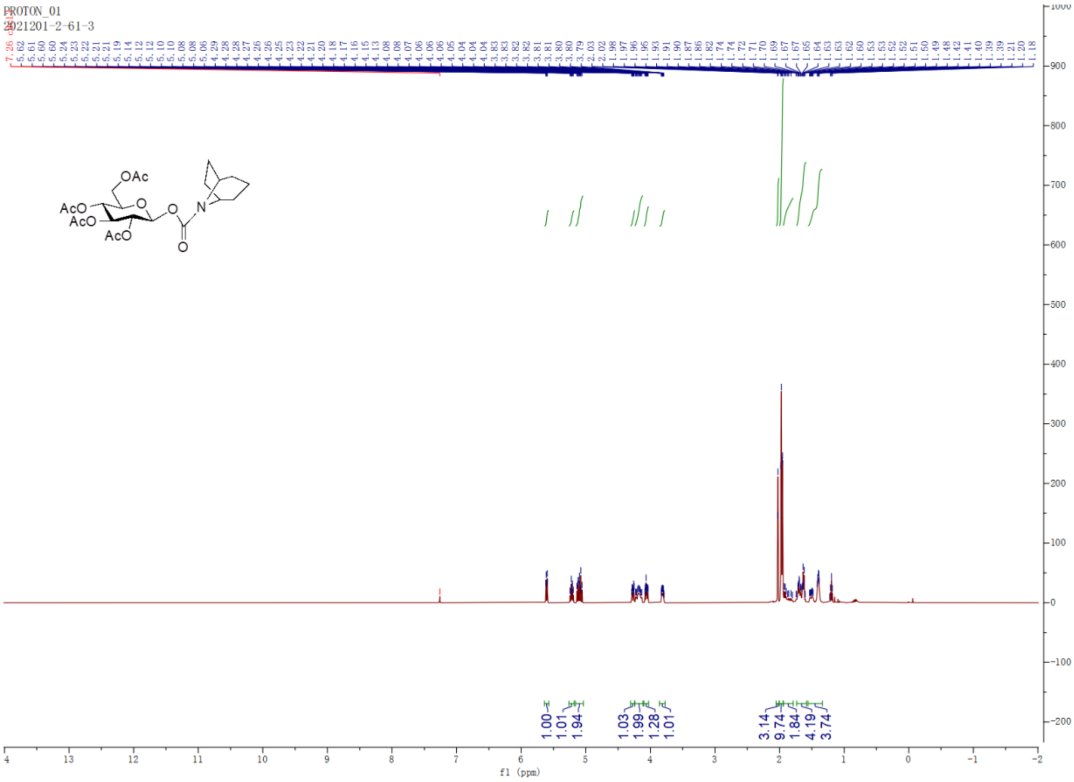

^{1}H NMR spectrum of compound **GA99** (500 MHz, $CDCl_3$)

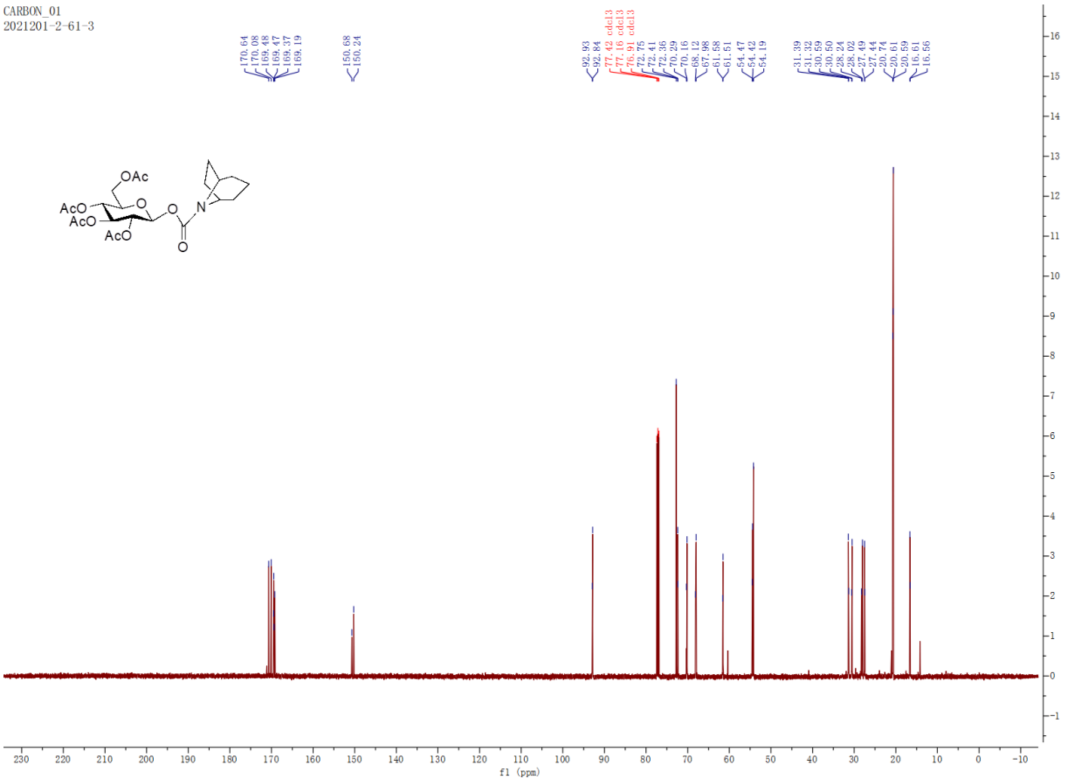

^{13}C NMR spectrum of compound **GA99** (126 MHz, $CDCl_3$)

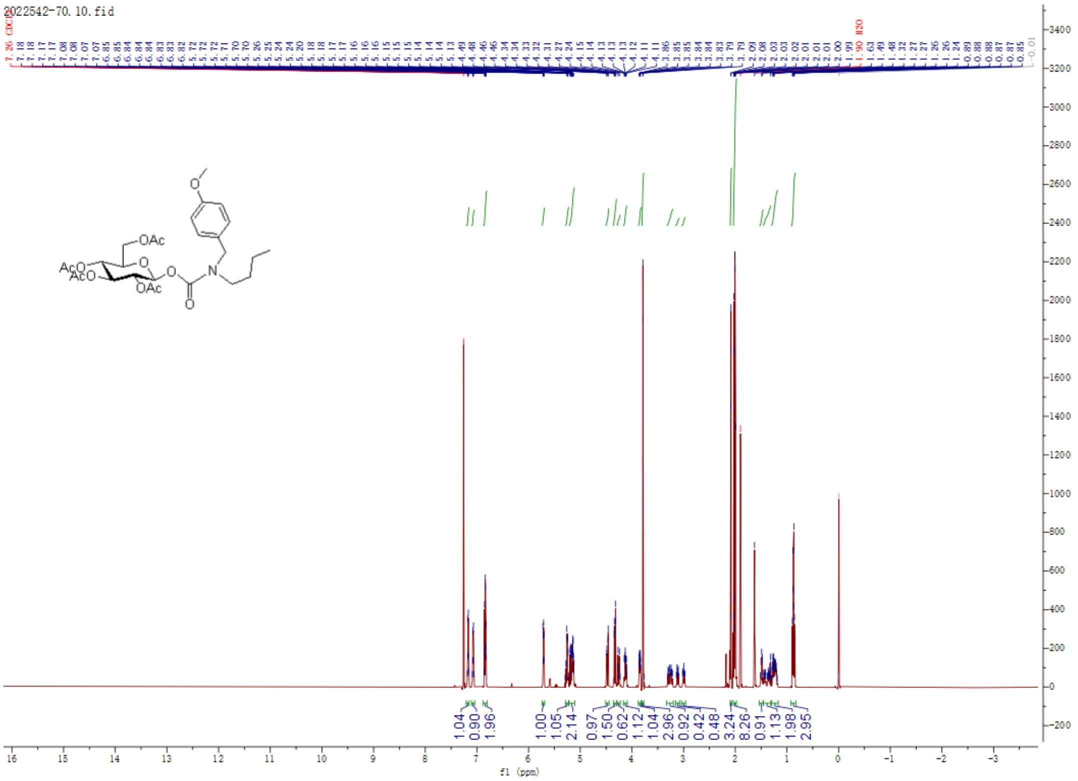

^{1}H NMR spectrum of compound **GA100** (600 MHz, $CDCl_3$)

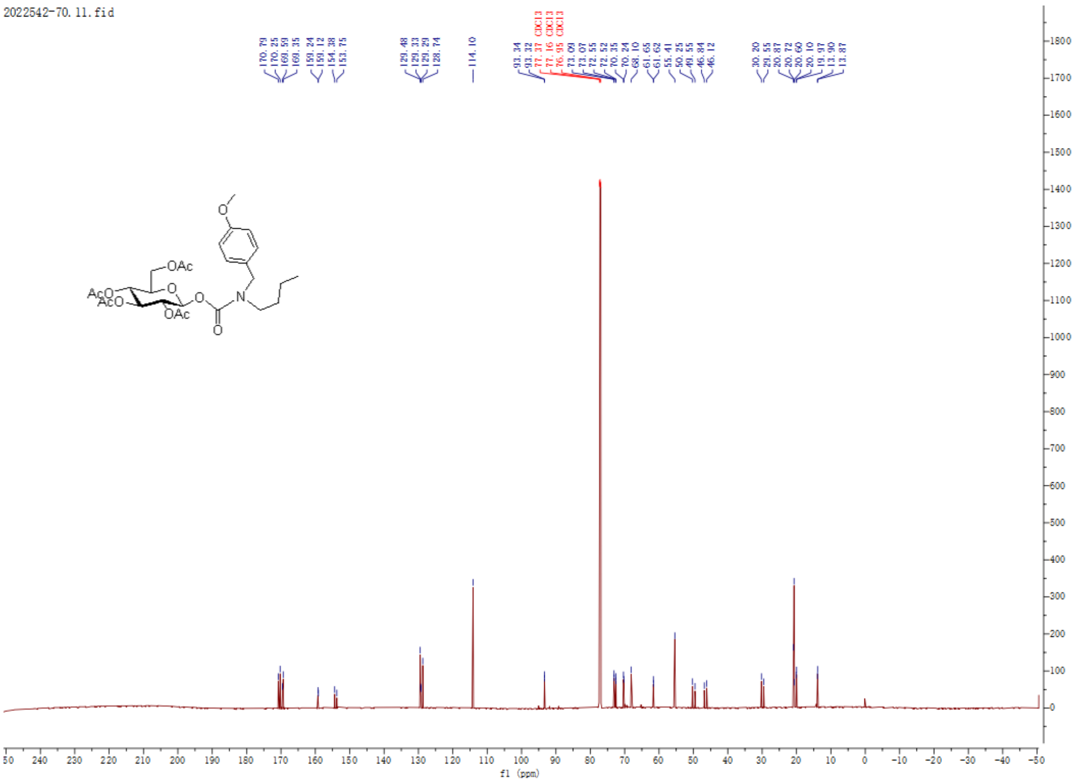

^{13}C NMR spectrum of compound **GA100** (151 MHz, $CDCl_3$)

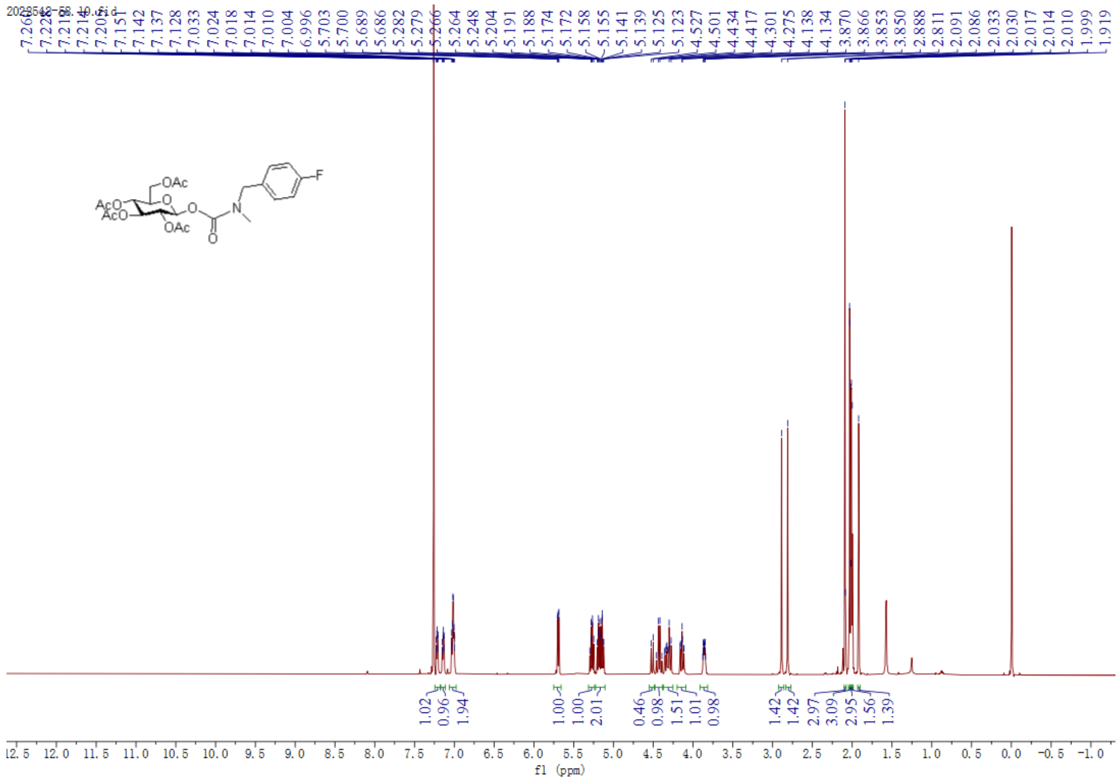

^{1}H NMR spectrum of compound **GA101** (600 MHz, $CDCl_3$)

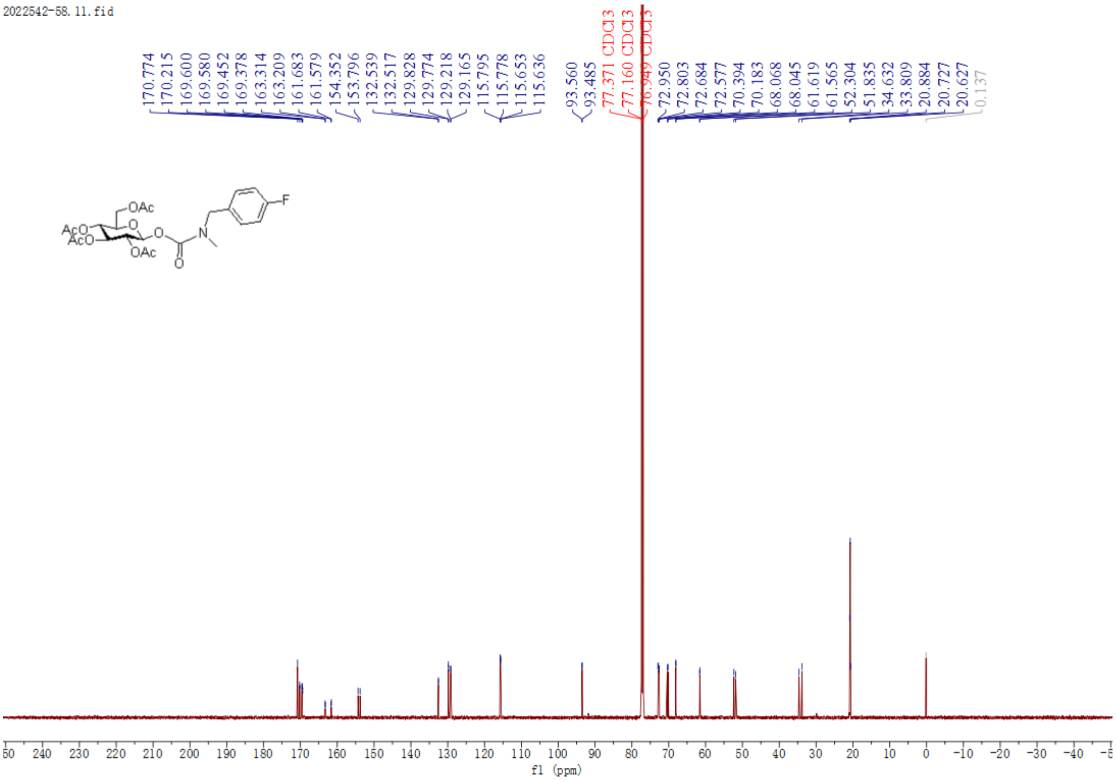

^{13}C NMR spectrum of compound **GA101** (151 MHz, $CDCl_3$)

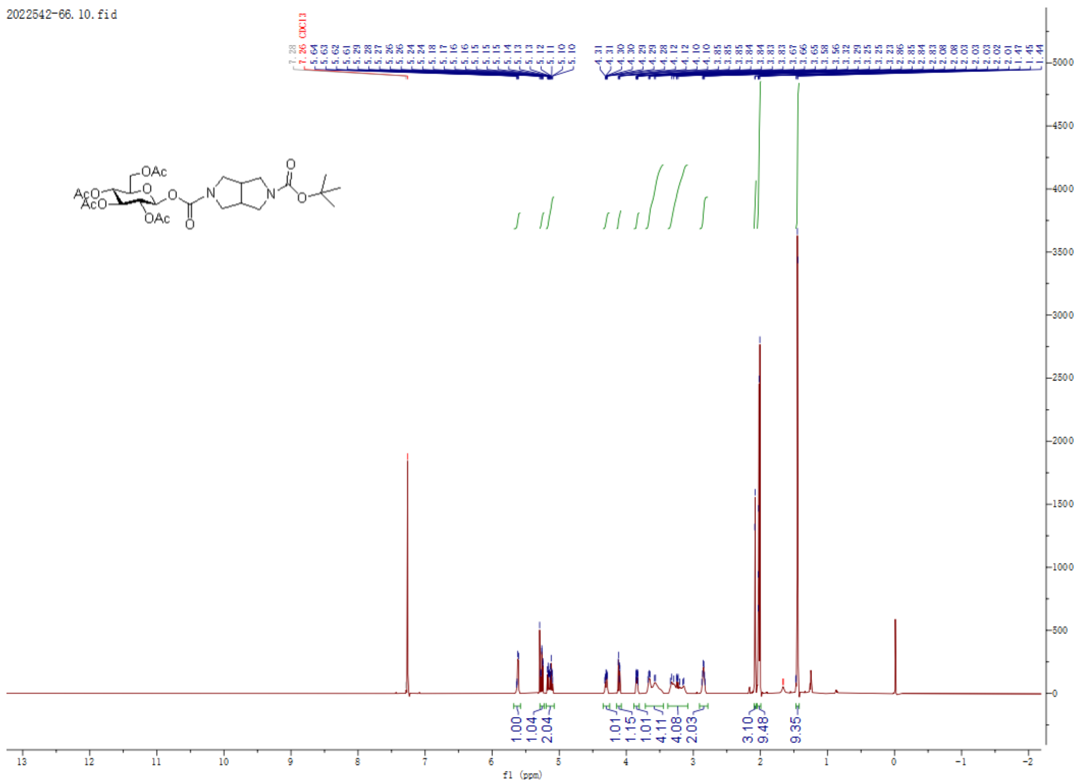

^{1}H NMR spectrum of compound **GA102** (600 MHz, $CDCl_3$)

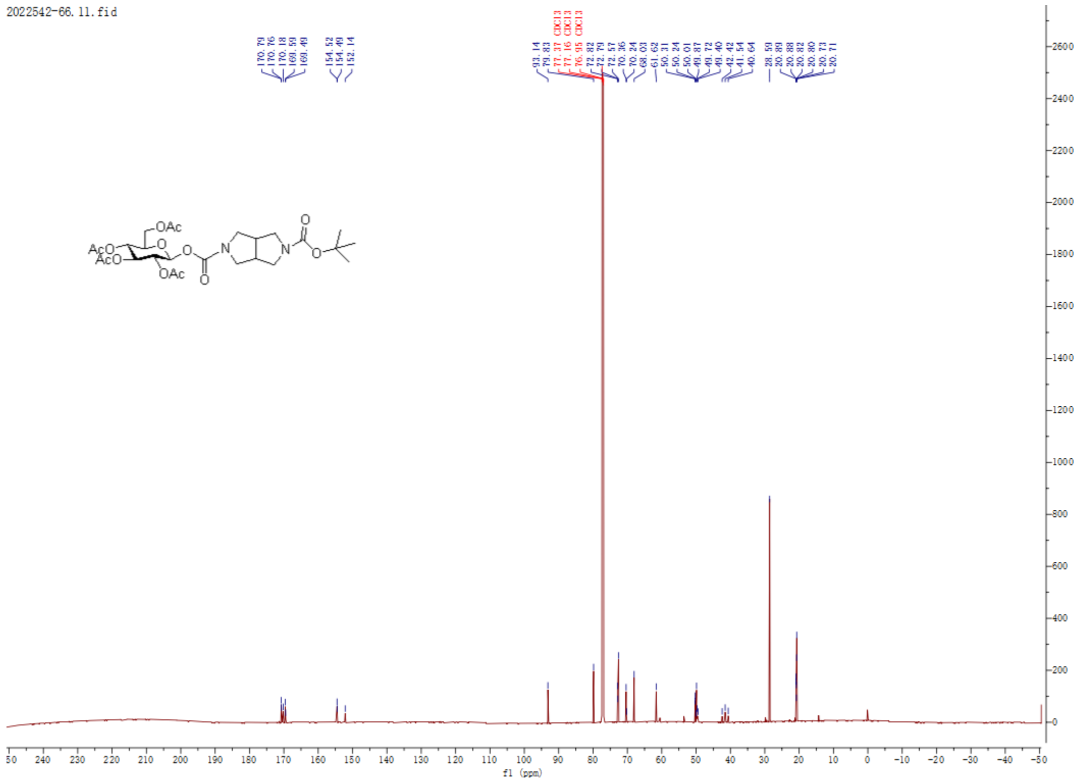

^{13}C NMR spectrum of compound **GA102** (151 MHz, $CDCl_3$)

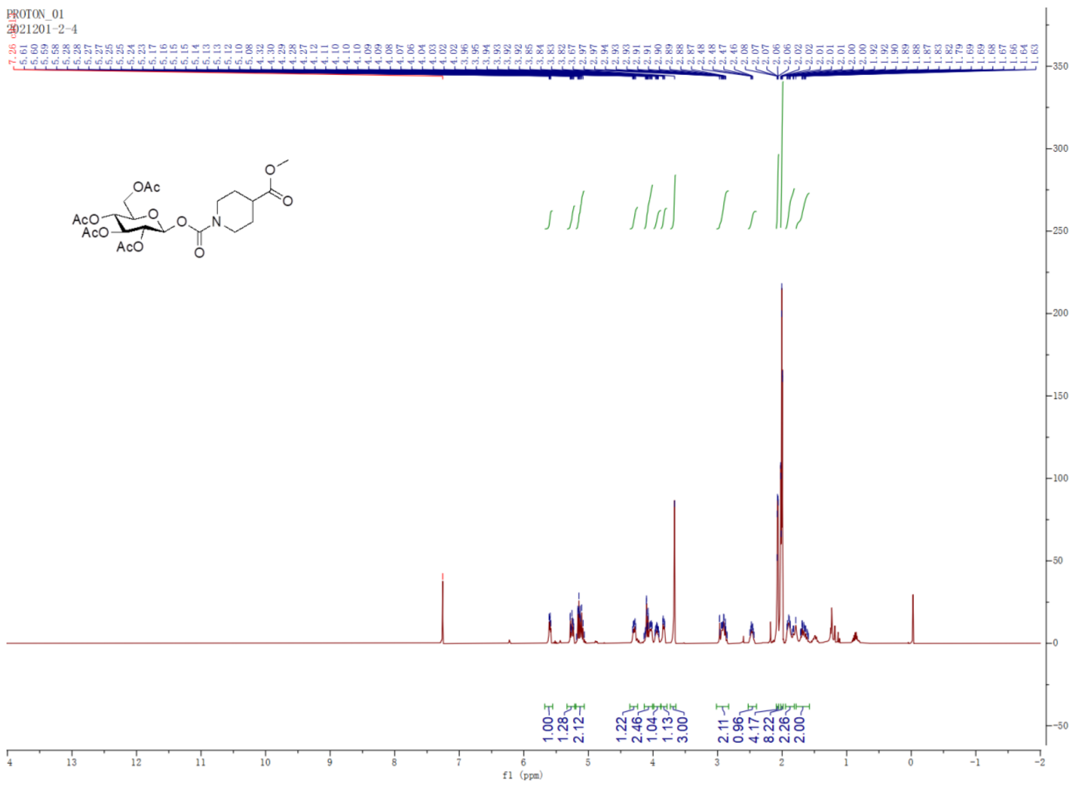

^{1}H NMR spectrum of compound **GA103** (500 MHz, $CDCl_3$)

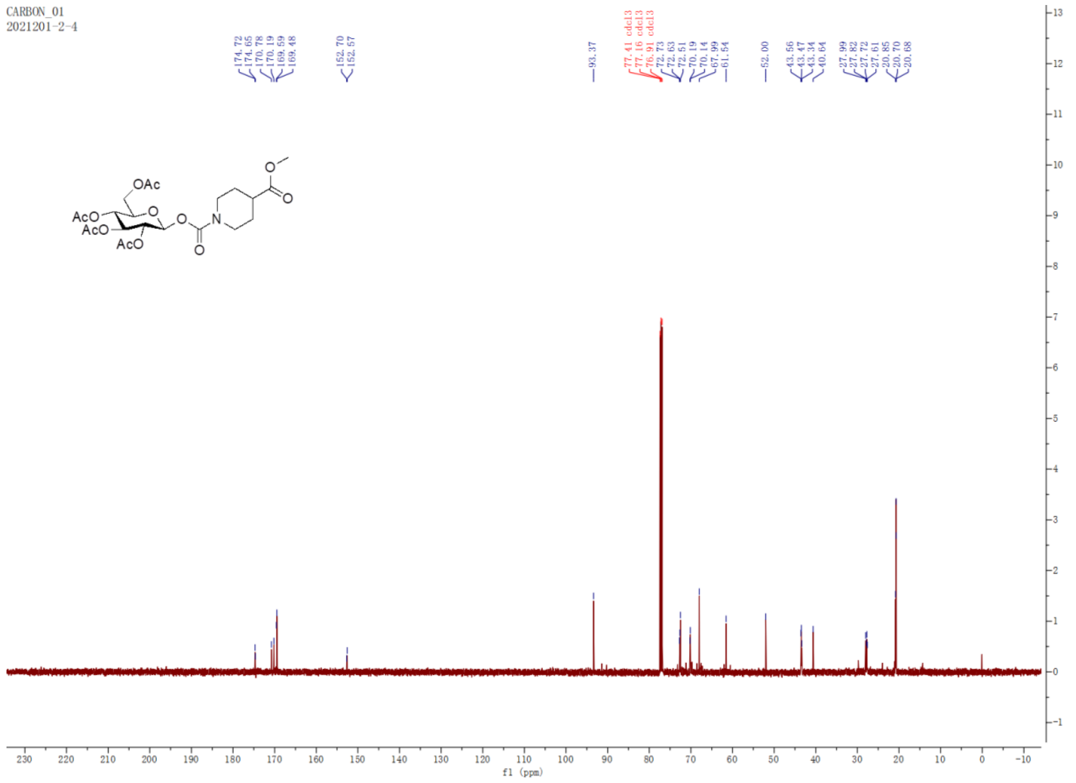

^{13}C NMR spectrum of compound **GA103** (126 MHz, $CDCl_3$)

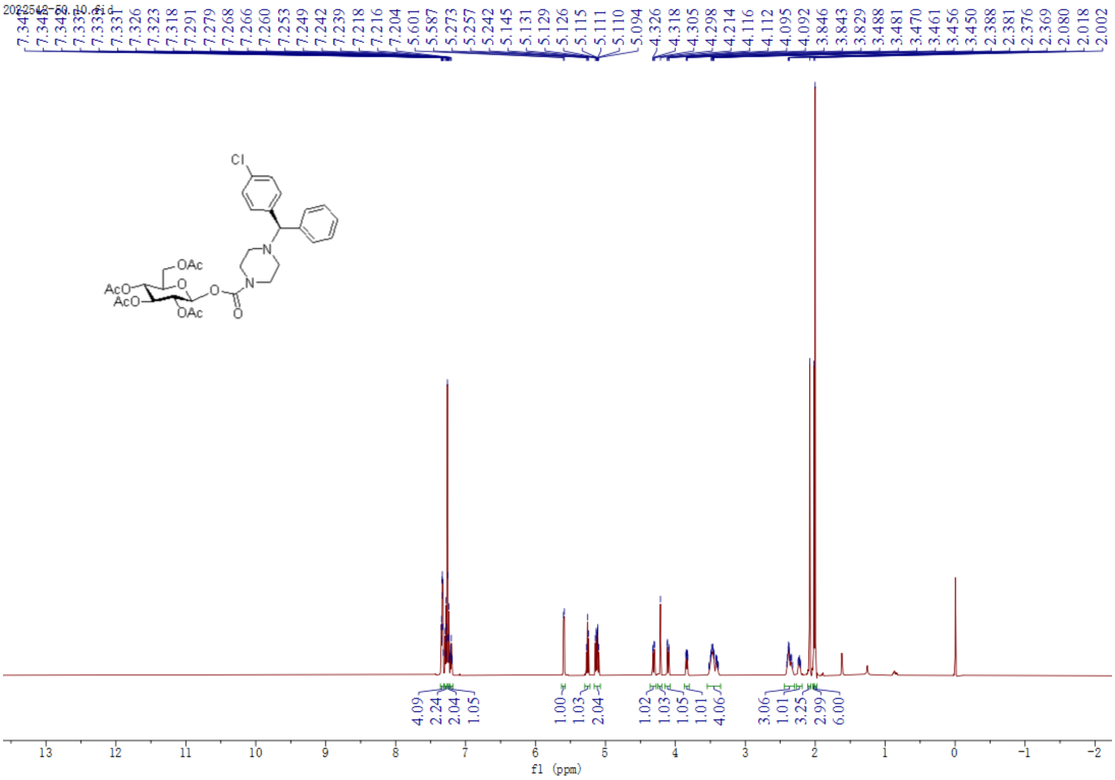

^{1}H NMR spectrum of compound **GA104** (600 MHz, $CDCl_3$)

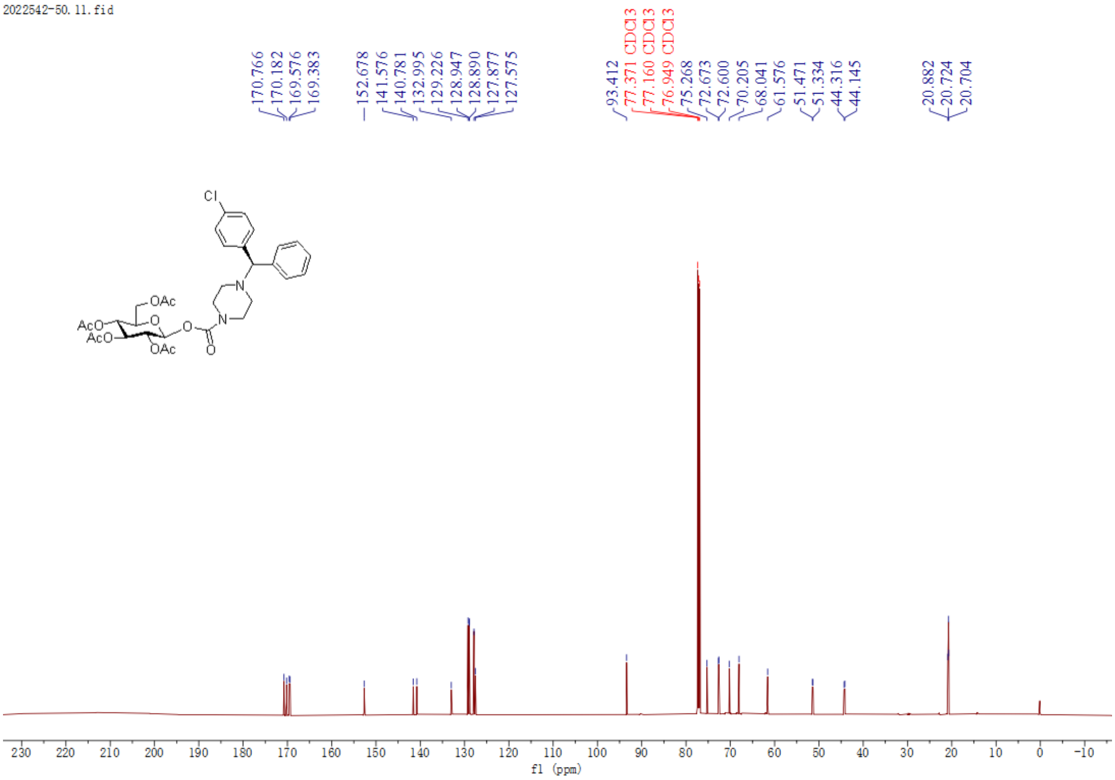

^{13}C NMR spectrum of compound **GA104** (151 MHz, $CDCl_3$)

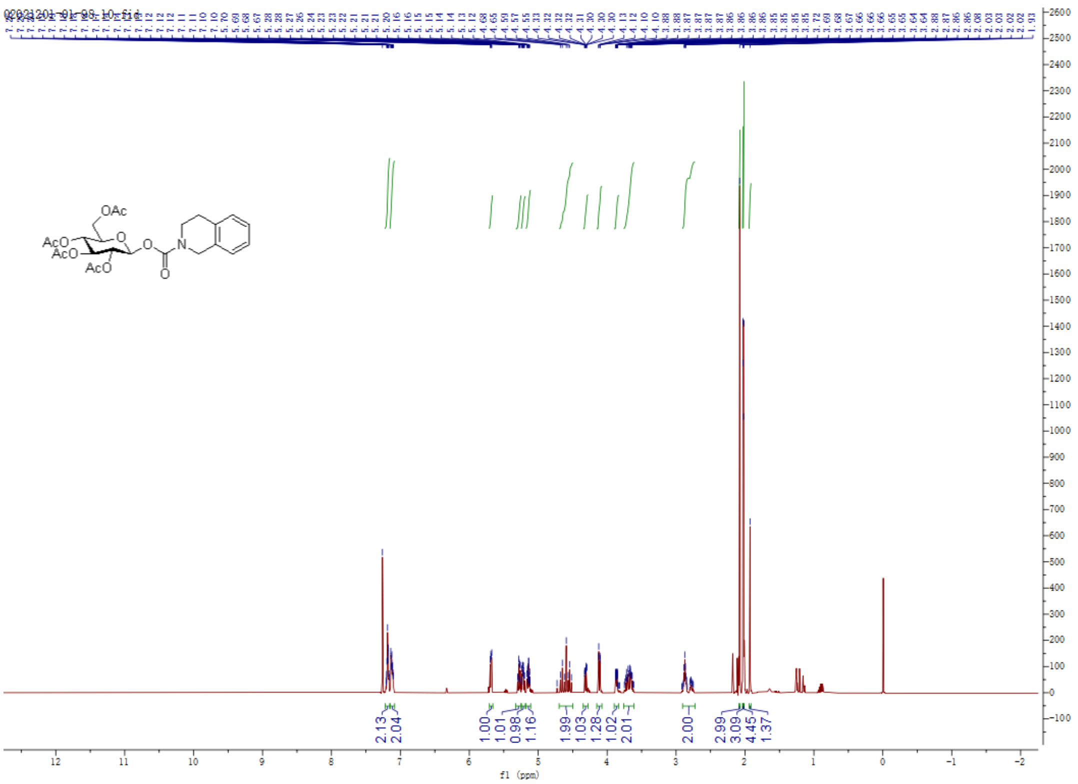

^{1}H NMR spectrum of compound **GA105** (600 MHz, $CDCl_3$)

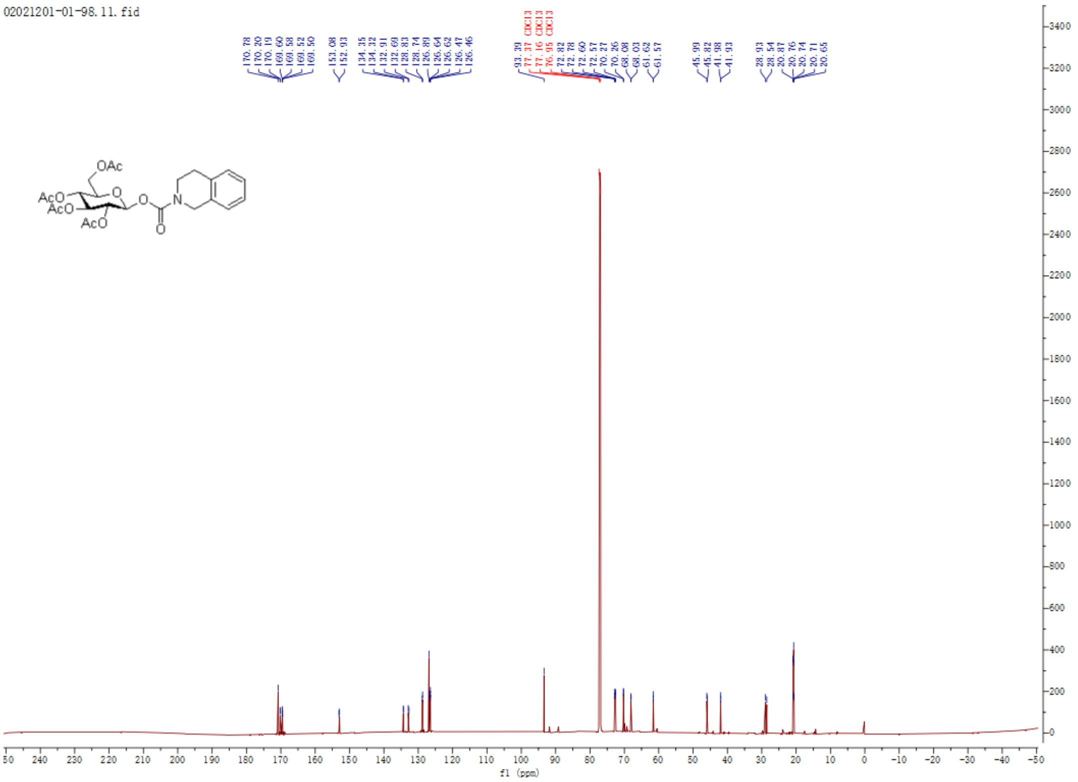

^{13}C NMR spectrum of compound **GA105** (151 MHz, $CDCl_3$)

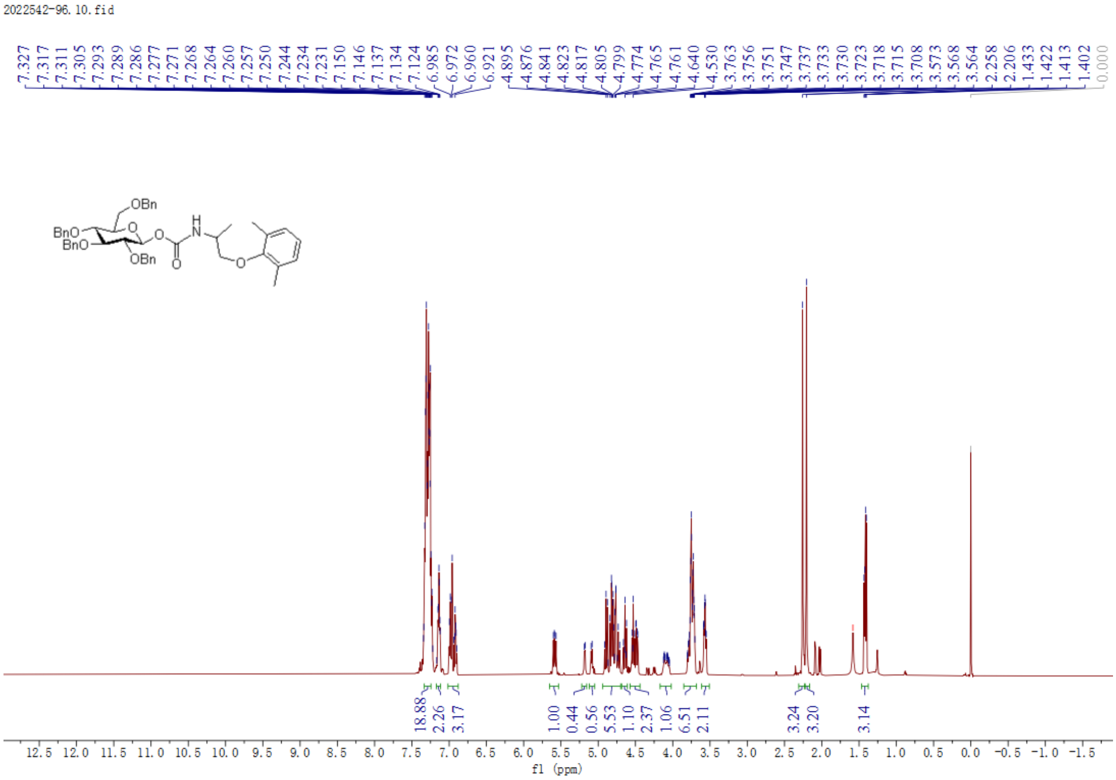

^{1}H NMR spectrum of compound **GA106** (600 MHz, $CDCl_3$)

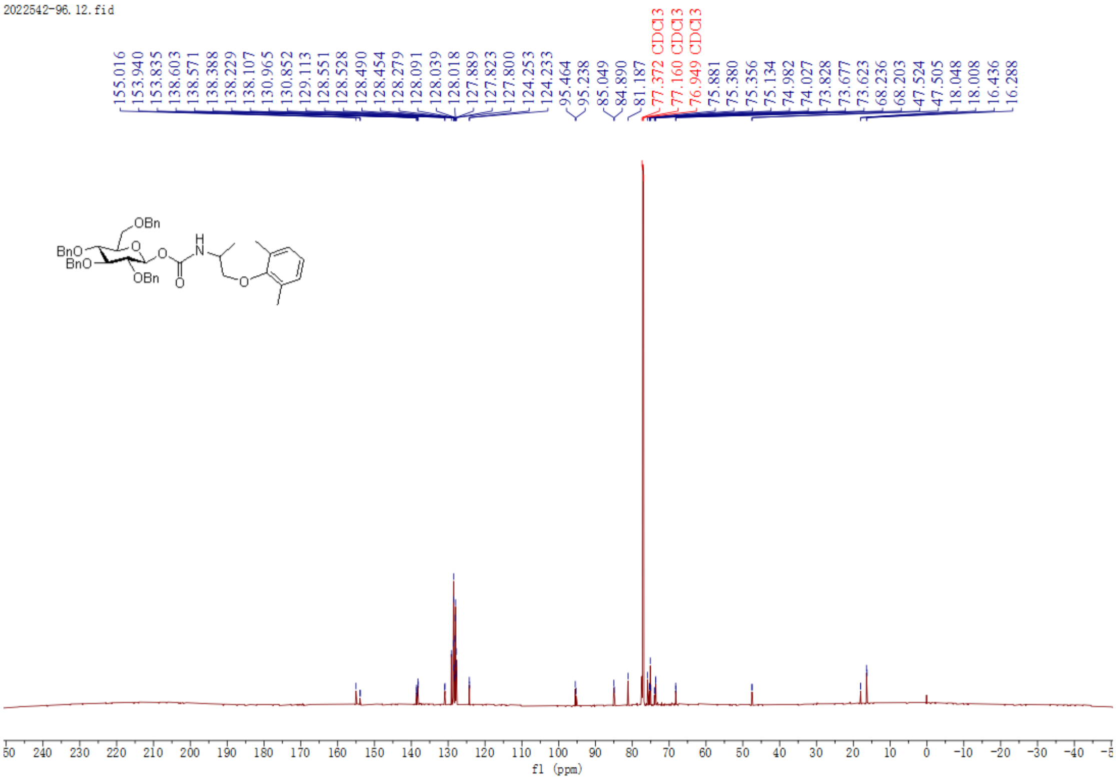

^{13}C NMR spectrum of compound **GA106** (151 MHz, $CDCl_3$)

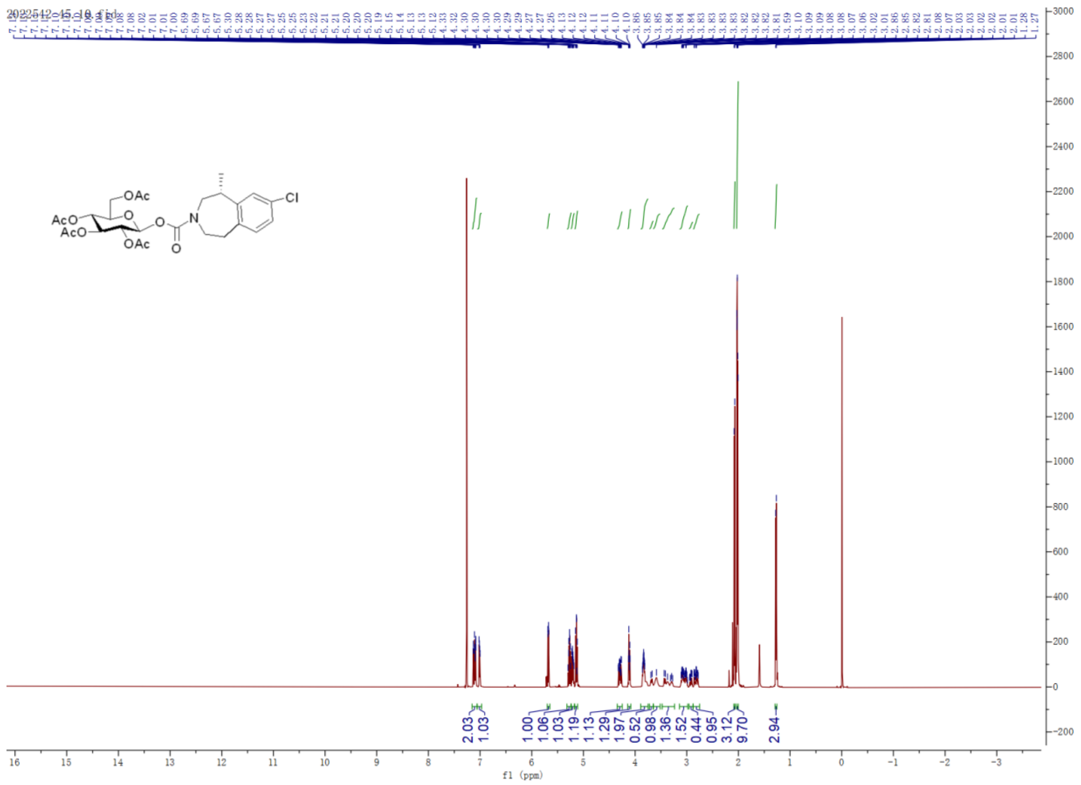

^{1}H NMR spectrum of compound **GA107** (600 MHz, $CDCl_3$)

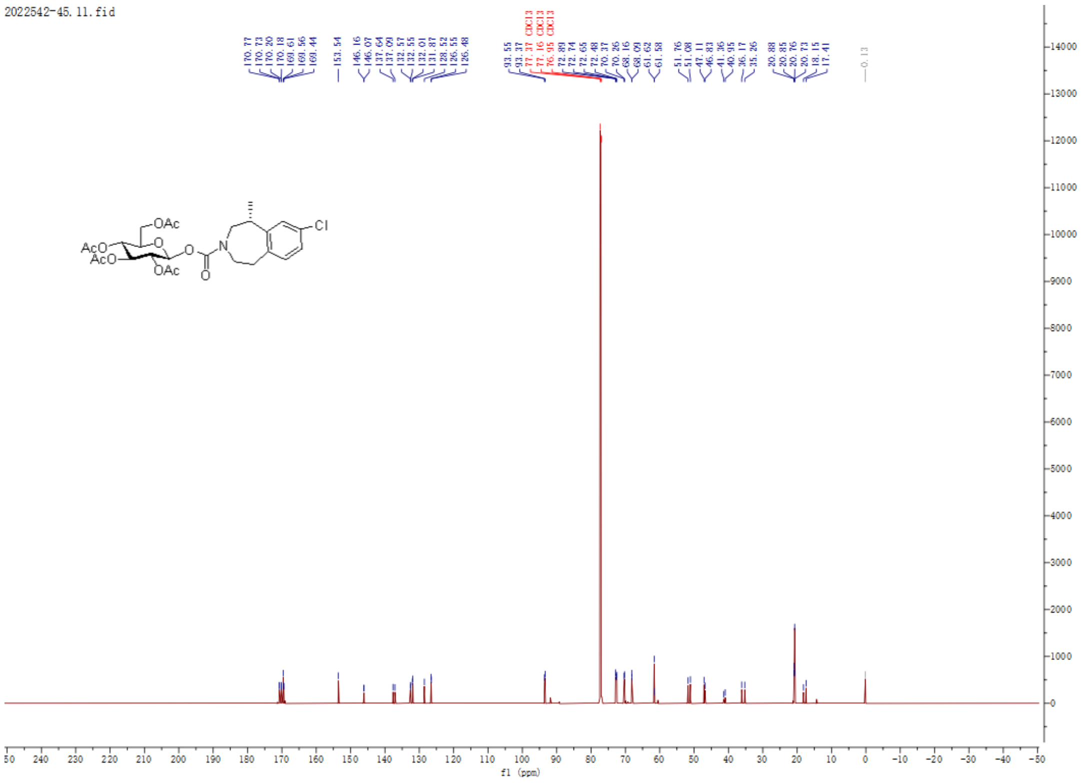

^{13}C NMR spectrum of compound **GA107** (151 MHz, $CDCl_3$)

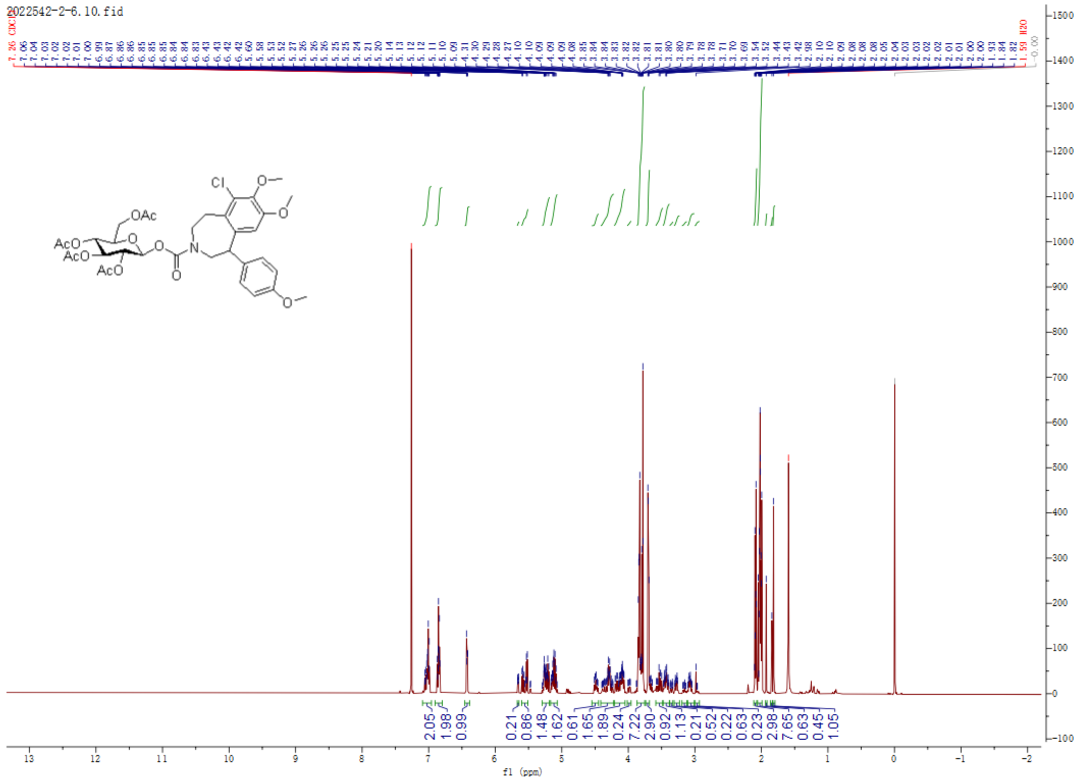

^{1}H NMR spectrum of compound **GA108** (600 MHz, $CDCl_3$)

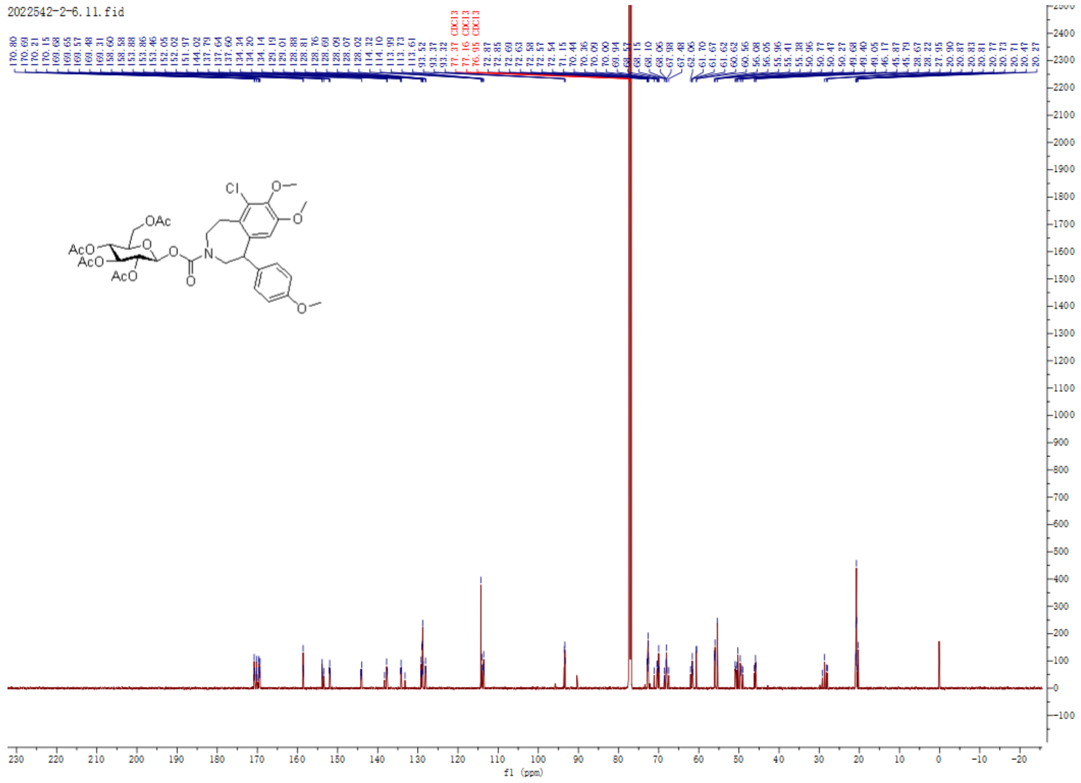

^{13}C NMR spectrum of compound **GA108** (151 MHz, $CDCl_3$)

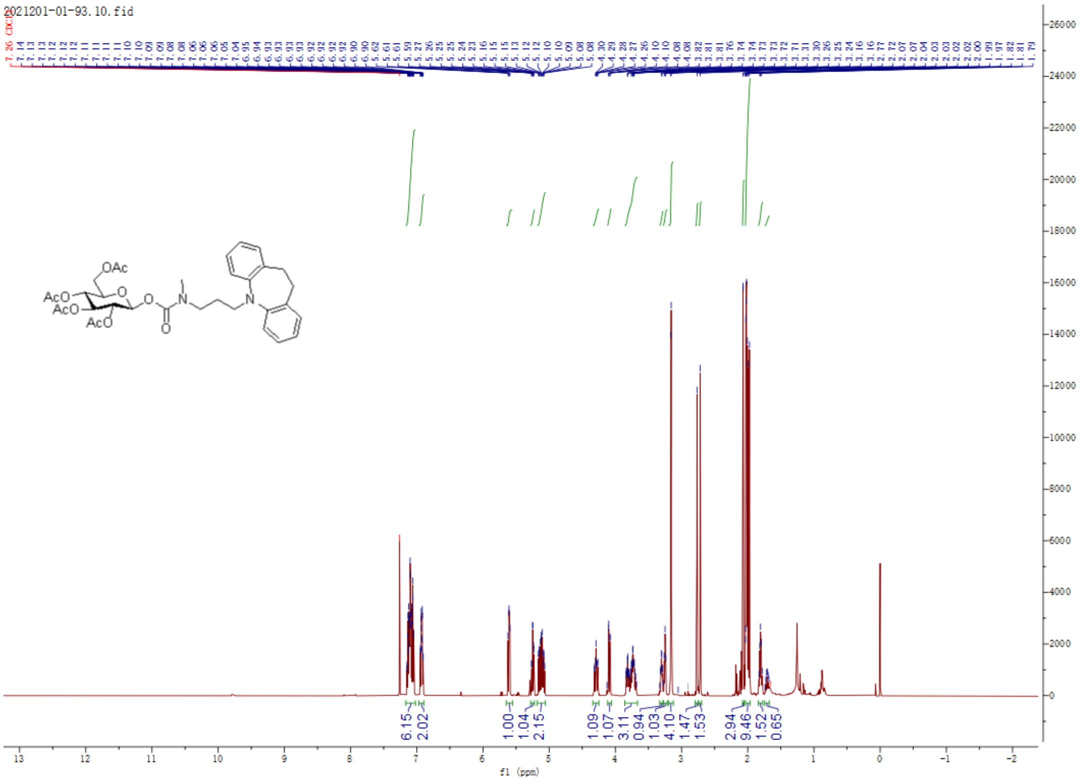

^{1}H NMR spectrum of compound **GA109** (600 MHz, $CDCl_3$)

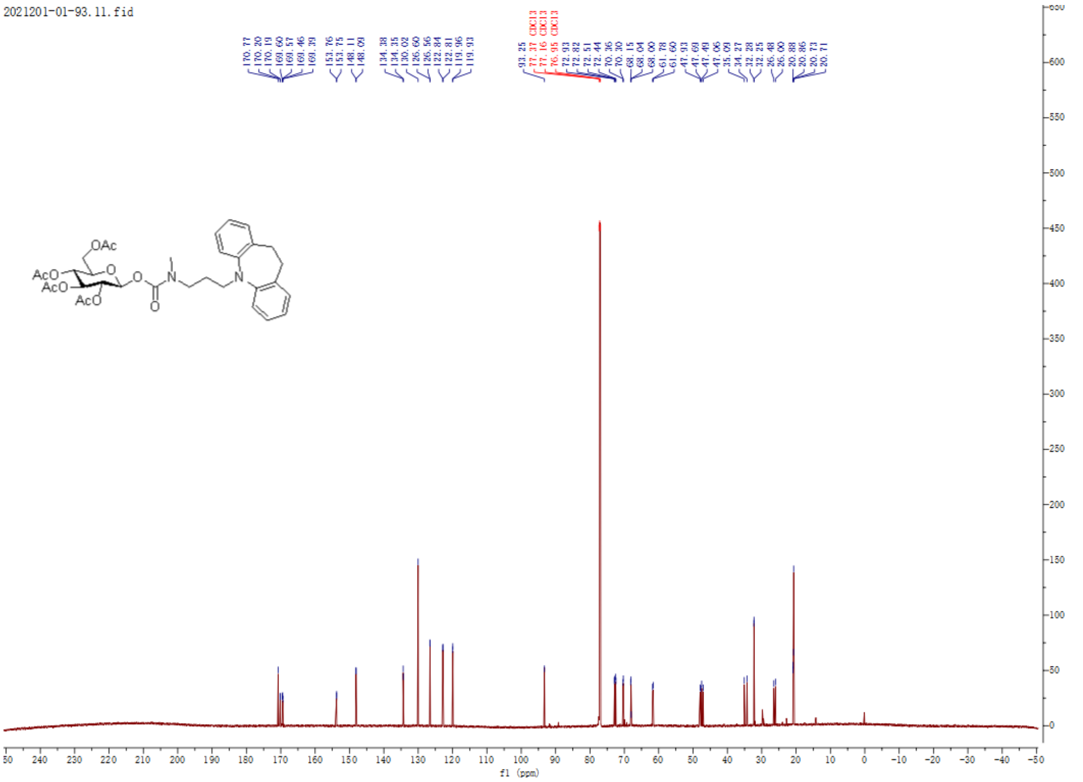

^{13}C NMR spectrum of compound **GA109** (151 MHz, $CDCl_3$)

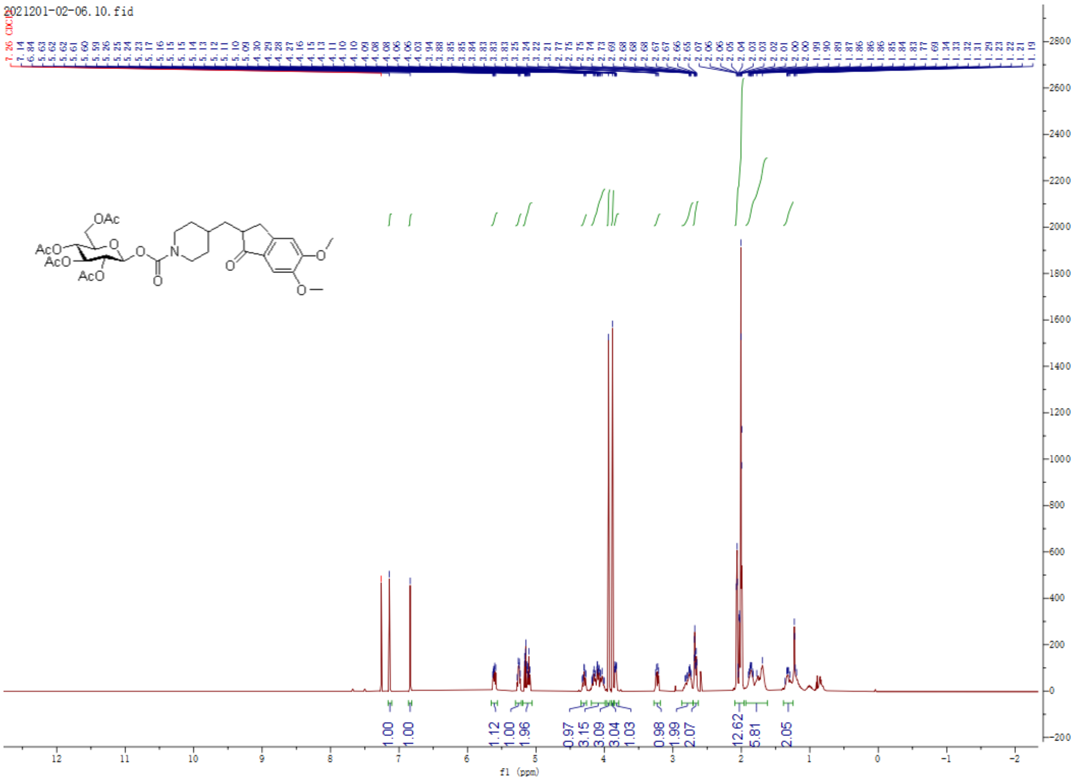

^{1}H NMR spectrum of compound **GA110** (600 MHz, $CDCl_3$)

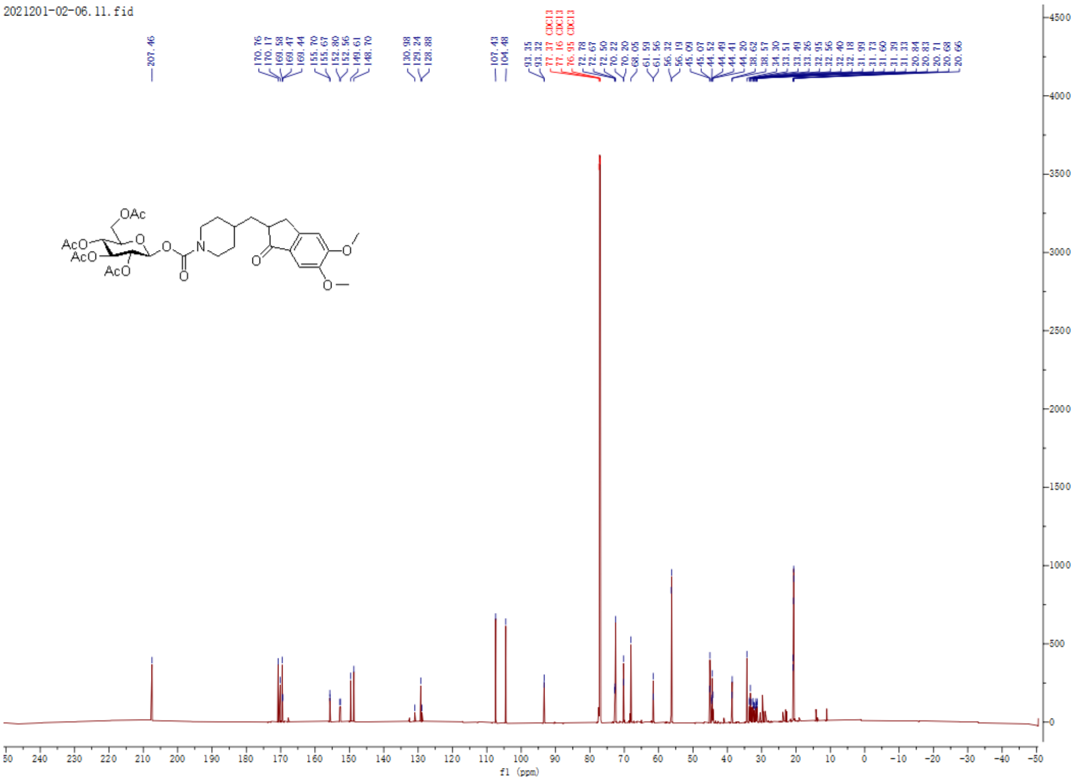

^{13}C NMR spectrum of compound **GA110** (151 MHz, $CDCl_3$)

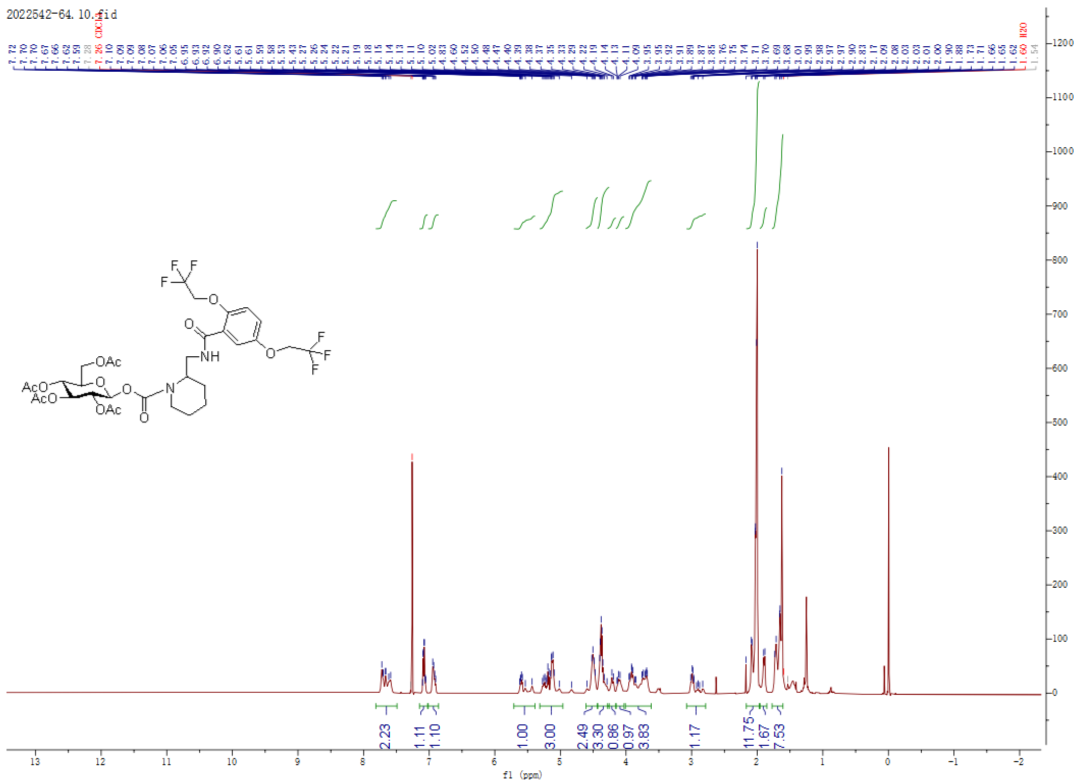

^{1}H NMR spectrum of compound **GA111** (600 MHz, $CDCl_3$)

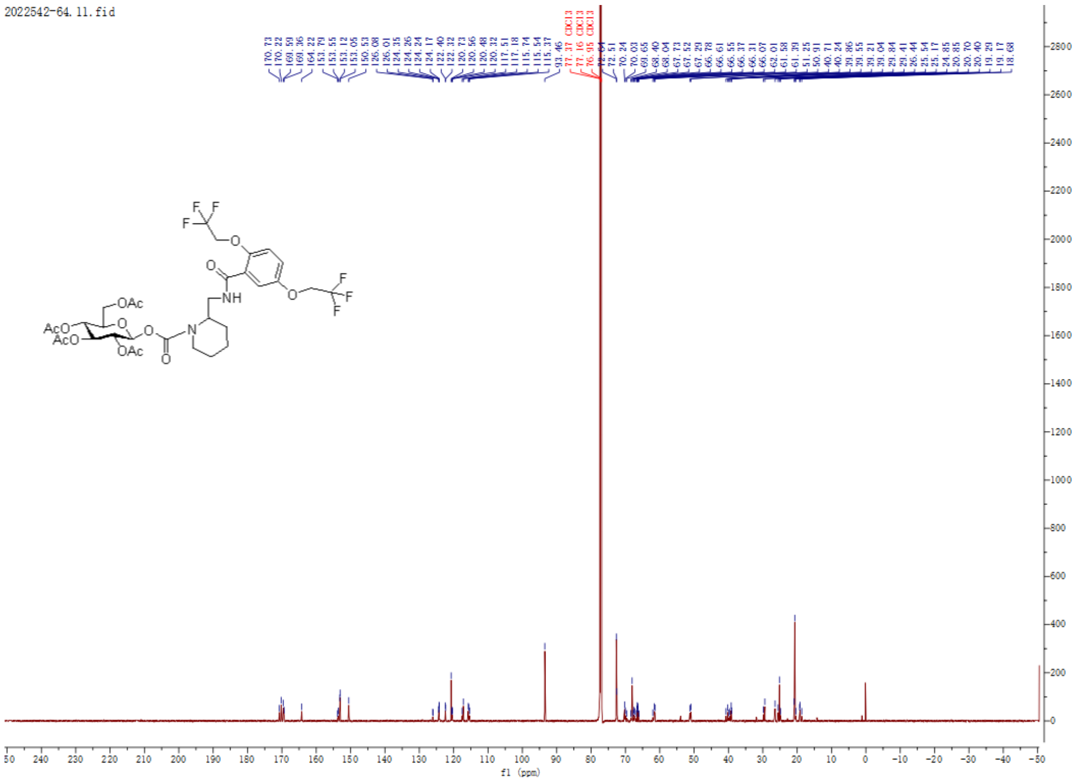

^{13}C NMR spectrum of compound **GA111** (151 MHz, $CDCl_3$)

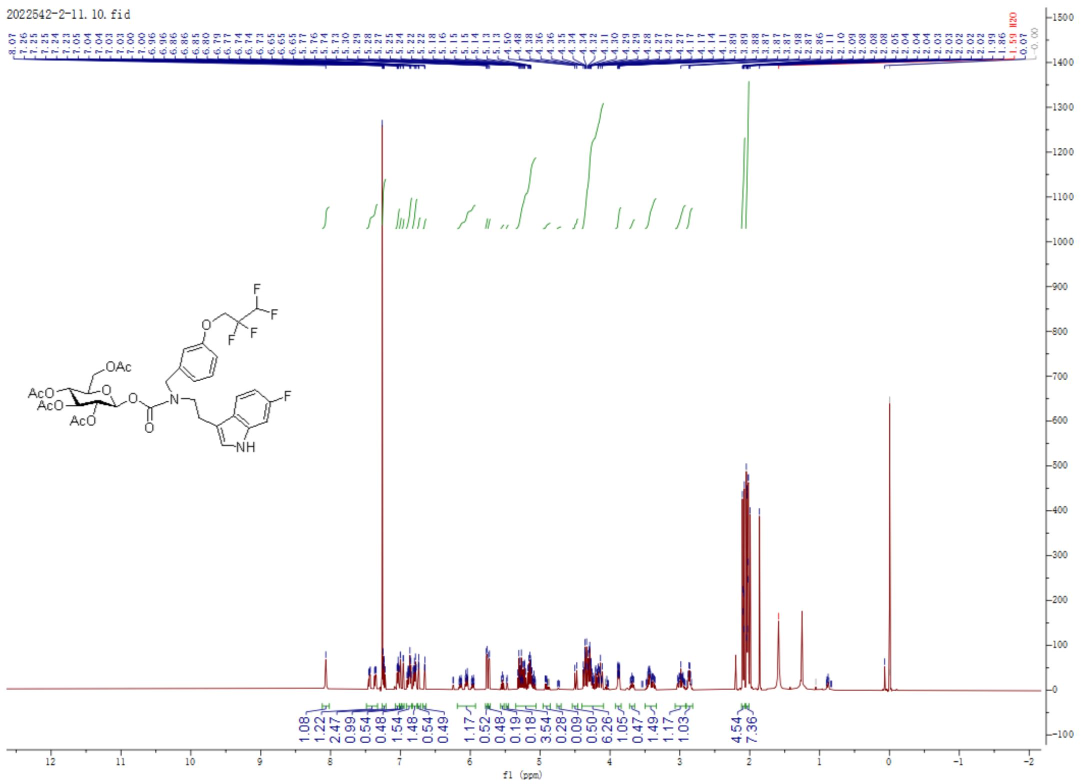

^{1}H NMR spectrum of compound **GA112** (600 MHz, $CDCl_3$)

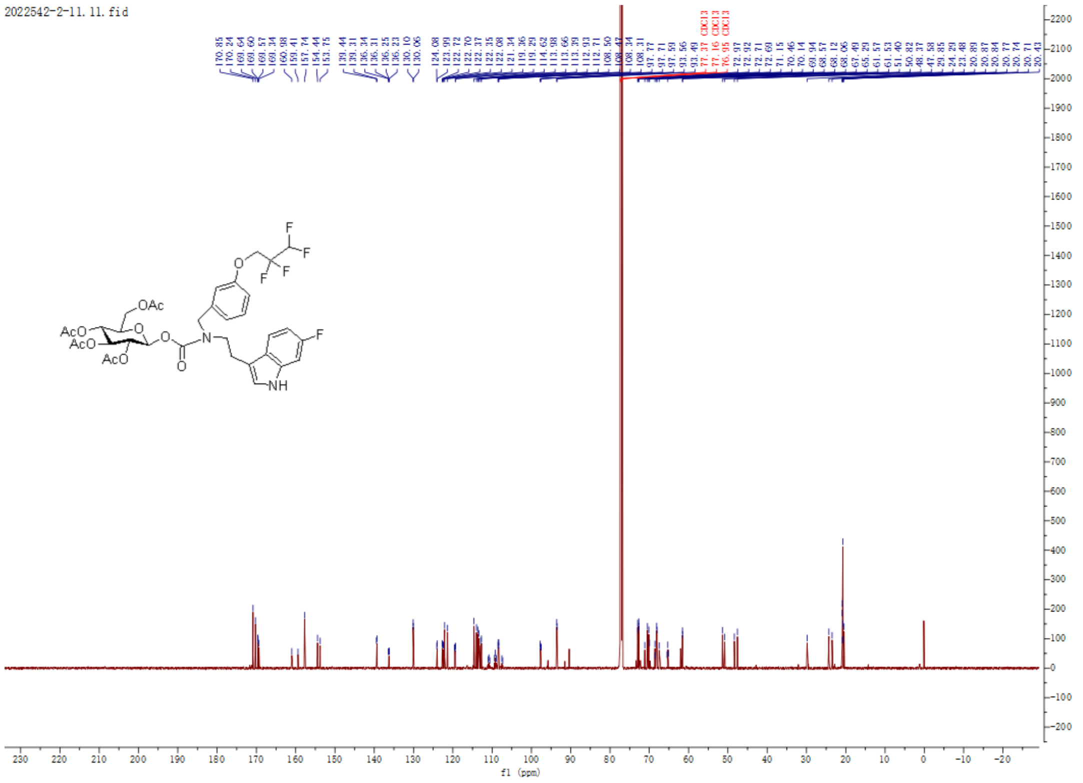

^{13}C NMR spectrum of compound **GA112** (151 MHz, $CDCl_3$)

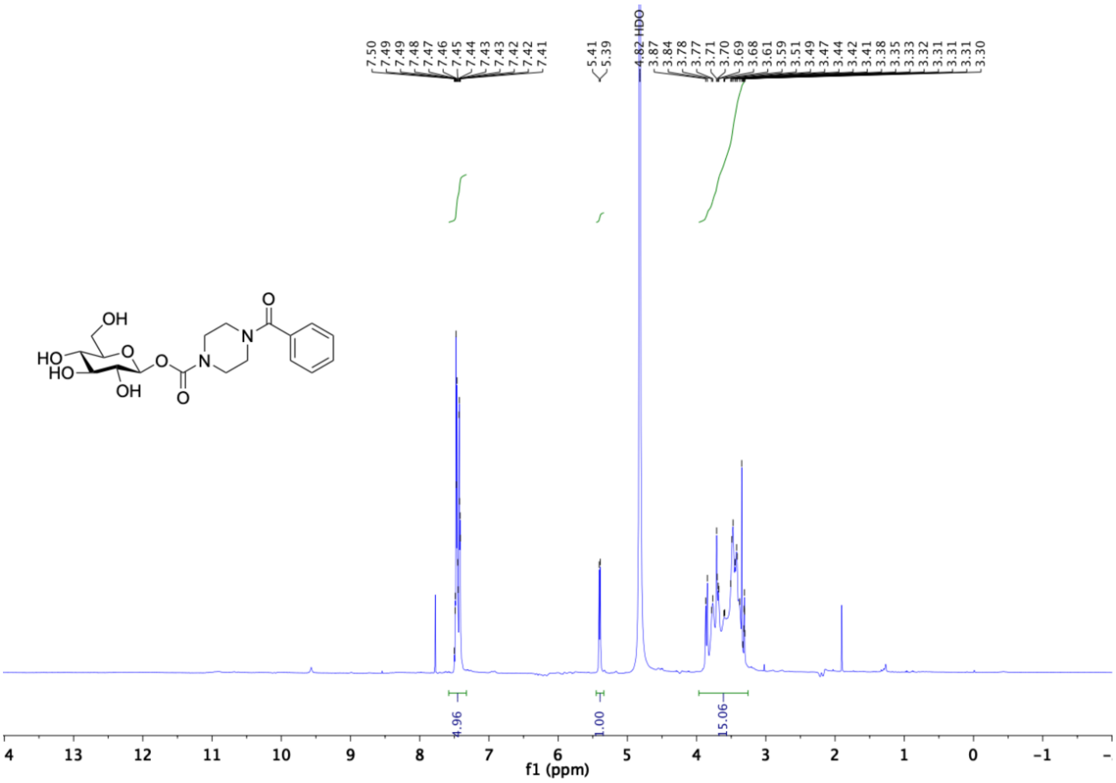

^{1}H NMR spectrum of compound **GA125** (500 MHz, CD_3OD)

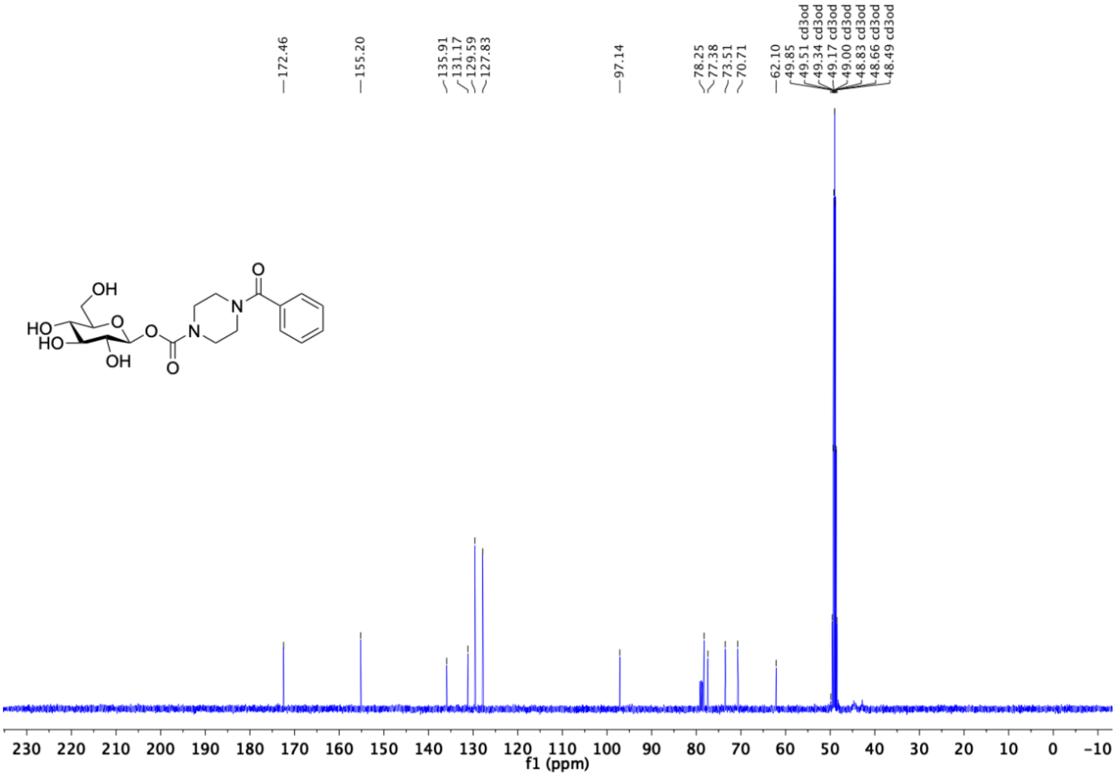

^{13}C NMR spectrum of compound **GA125** (126 MHz, CD_3OD)

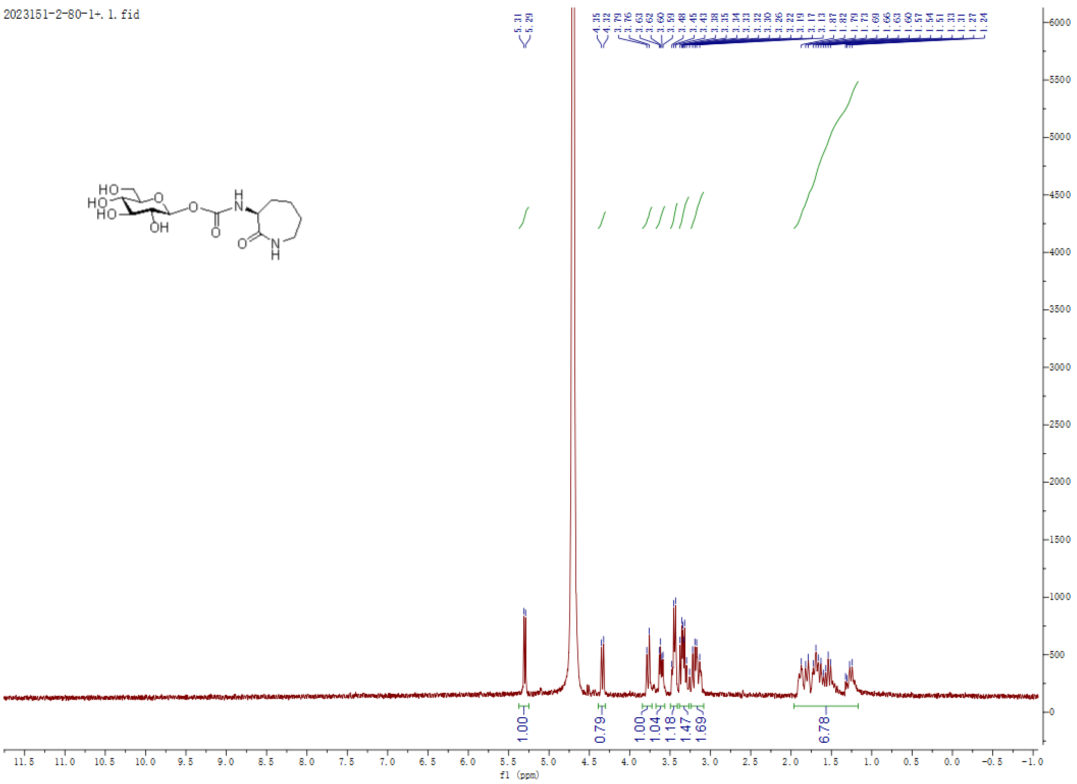

^{1}H NMR spectrum of compound **GA126** (500 MHz, CD_3OD)

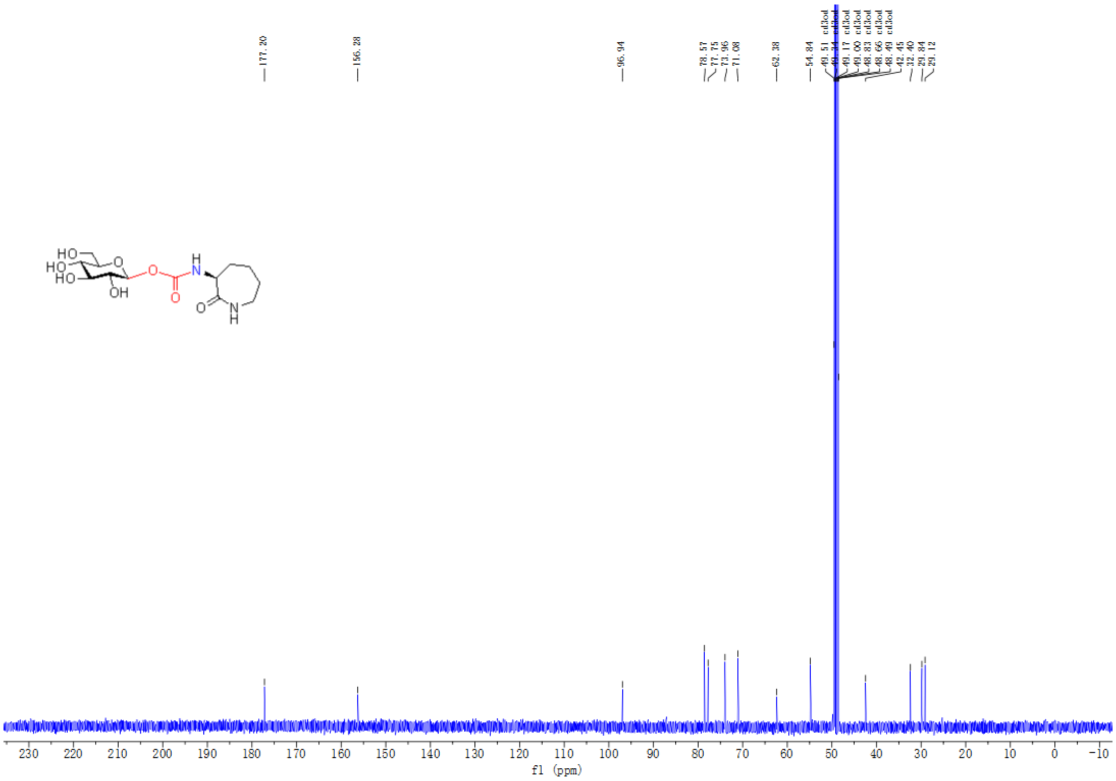

^{13}C NMR spectrum of compound **GA126** (126 MHz, CD_3OD)

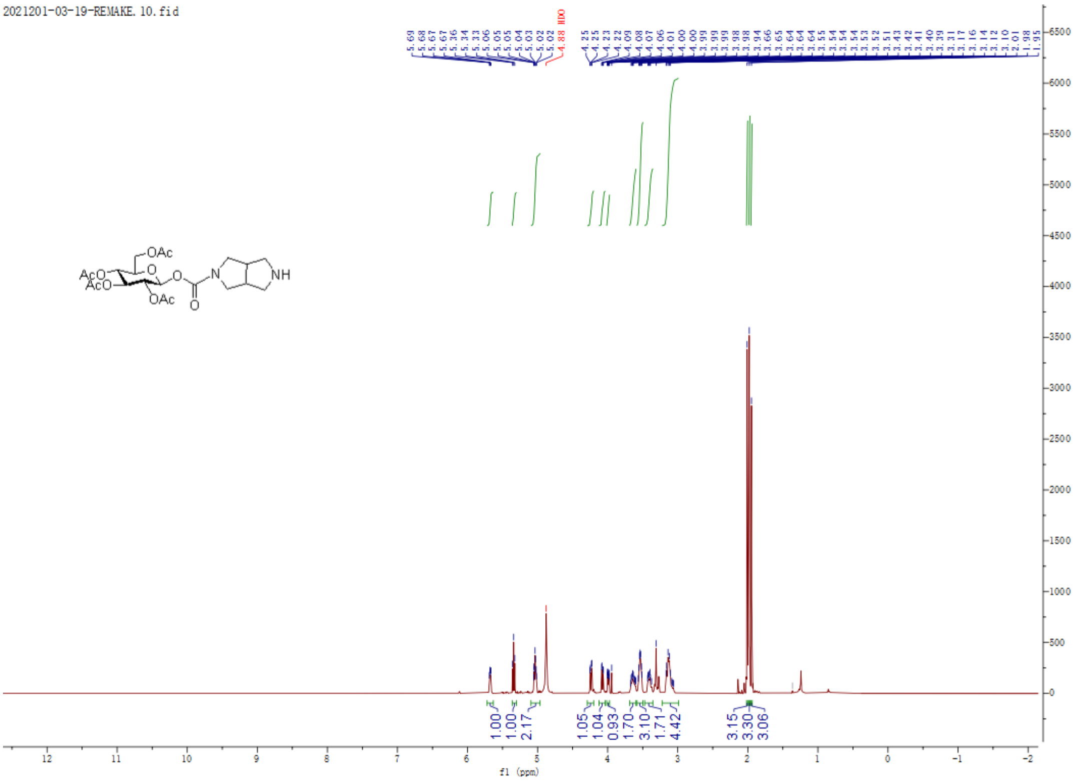

^{1}H NMR spectrum of compound **GA127** (600 MHz, CD_3OD)

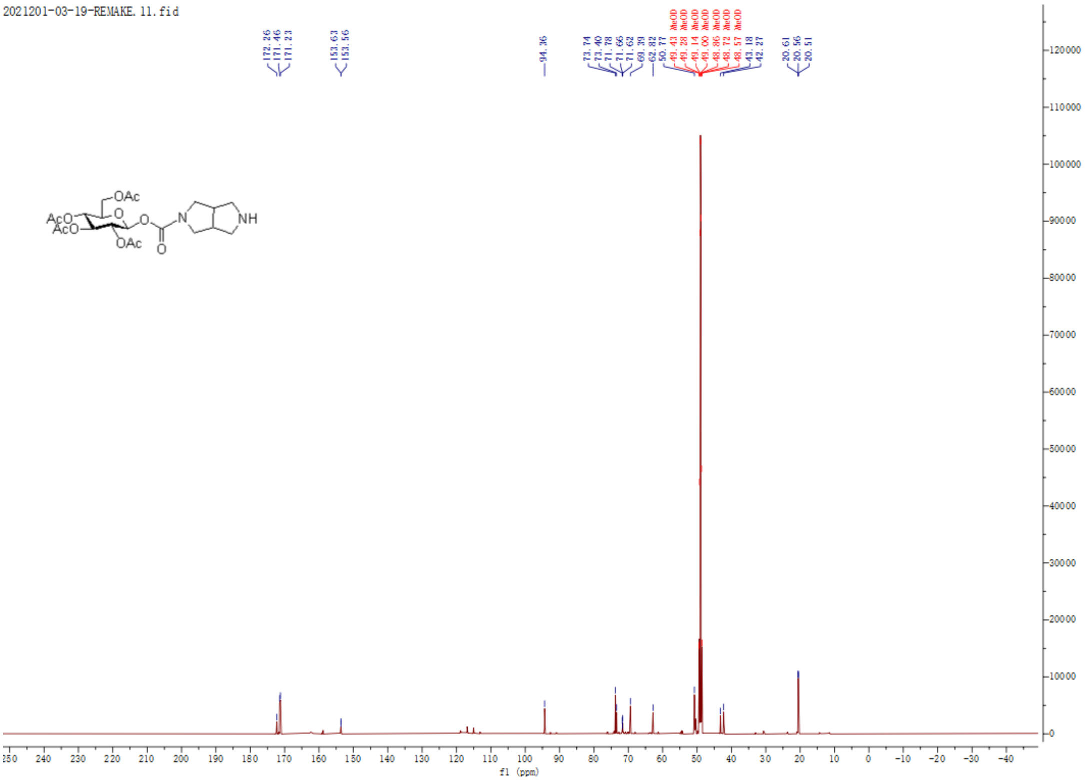

^{13}C NMR spectrum of compound **GA127** (151 MHz, CD_3OD)

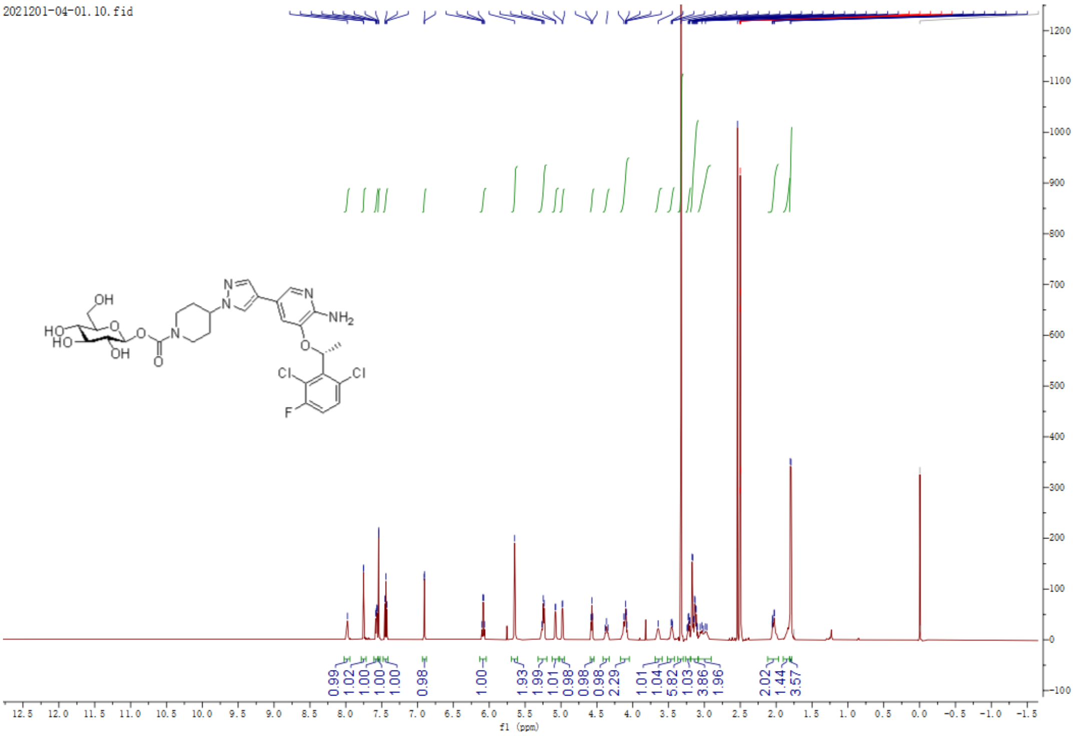

^{1}H NMR spectrum of compound **GA95** (600 MHz, DMSO-d_6)

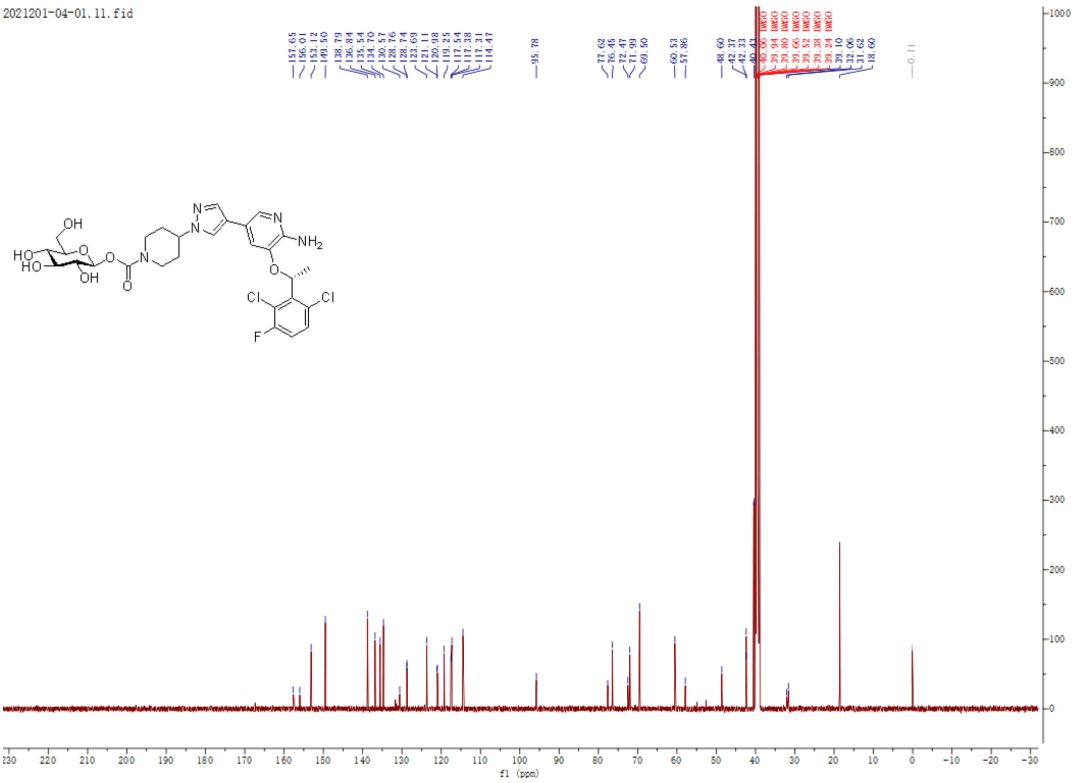

^{13}C NMR spectrum of compound **GA95** (151 MHz, DMSO-d_6)

References

1. Wang, Q. B., Fu, J. & Zhang, J. B. A facile preparation of peracylated alpha-aldopyranosyl chlorides with thionyl chloride and tin tetrachloride. *Carbohydr. Res*. **343**, 2989–2991 (2008).
2. Xu, L., Luo, C. H. & Chen, C. S. Halogenation and anomerization of glycopyranoside by TESH/bromine and BHQ/bromine. *J. Chin. Chem. Soc.* **68**, 315–321 (2021).
3. Geringer, S. A. & Demchenko, A. V. Iron(III) chloride-catalyzed activation of glycosyl chlorides. *Org. Biomol. Chem*. **16**, 9133–9137 (2018).
4. Kovac, P., Yeh, H. J. C. & Glaudemans, C. P. J. Synthesis and n.m.r. spectra of methyl 2-deoxy-2-fluoro- and 3-deoxy-3-fluoro-α- and β-D-glucopyranosides. *Carbohydr. Res*. **169**, 23–34 (1987).
5. Salvadó, M. Amgarten, B., Castillón, S., Bernardes, G. J. L. & Boutureira, O. Synthesis of fluorosugar reagents for the construction of well-defined fluoroglycoproteins. *Org. Lett*. **17**, 2836–2839 (2015).
6. McCarter, J. D., Yeung, W., Chow, J., Dolphin, D. & Withers, S. G. Design and synthesis of 2′-deoxy-2′-fluorodisaccharides as mechanism-based glycosidase inhibitors that exploit aglycon specificity. *J. Am. Chem. Soc*. **119**, 5792–5797 (1997).
7. Nieschalk, J. & O'Hagan, D. Synthesis of 6-fluoro-D-olivose (2,6-dideoxy-6-fluoro-D-arabinohexopyranose). *J. Fluor. Chem*. **91**, 159–163. (1998).
8. Schmidt, R. R. & Rücker, E. Stereoselective glycosidations of uronic acids. *Tetrahedron Lett*. **21**, 1421–1424 (1980).
9. Zhao, G. Y., Yao, W., Mauro, J. N. & Ngai, M. Y. Excited-state palladium-catalyzed 1,2-spin-center shift enables selective C-2 reduction, deuteration, and iodination of carbohydrates. *J. Am. Chem. Soc*. **143**, 1728–1734 (2021).
10. Matwiejuk, M. & Thiem, J. New method for regioselective glycosylation

employing saccharide oxyanions. *Eur. J. Org. Chem.* **29**, 5860–5878 (2011).

11. Gouliaras, C., Lee, D., Chan, L. & Taylor, M. S. Regioselective activation of glycosyl acceptors by a diarylborinic acid-derived catalyst. *J. Am. Chem. Soc.* **133**, 13926–13929 (2011).
12. Gillard, J. W. & Israel, M. Trimethylsilyl bromide as a mild, stereoselective anomeric brominating agent. *Tetrahedron Lett.* **22**, 513–516 (1981).
13. Tadashi, H., Katsuya, I., Kaori, S. & Takafumi, H. Efficient total syntheses of natural neopterin glycosides: Neopterin glucuronide and solfapterin. *Heterocycles* **95**, 390–409 (2017).
14. van Well, R.M.; Ravindranathan Kartha, K.P.; Field, R.A. Iodine Promoted Glycosylation with Glycosyl Iodides: α-Glycoside Synthesis. *J. Carbohydr. Chem.* **24**, 463–474 (2005).
15. Tang, Y., Yu, B. & Zhu, Q. Y. $GeCl_2$·dioxane-$AgBF_4$ catalyzed activation of glycosyl fuorides for glycosylation. *Org. Lett.* **24**, 3626–3630 (2022).
16. Cheschev, P., Marra, A. & Dondoni, A. Direct epoxidation of D-glucal and D-galactal derivatives with in situ generated DMDO. *Carbohydr. Res.* **341**, 2714-2716 (2006).
17. Lebedel, L., Ardá, A., Martin, A., Désiré, J., Mingot, A., Aufiero, M., Aiguabella Font, N., Gilmour, R., Jiménez-Barbero, J., Blériot, Y. & Thibaudeau, S. Structural and computational analysis of 2-halogeno-glycosyl cations in the presence of a superacid: an expansive platform. *Angew. Chem. Int. Ed.* **58**, 13758–13762 (2019).
18. Bucher, C. & Gilmour, R. Fluorine-Directed Glycosylation. *Angew. Chem. Int. Ed.* **49**, 8724–8728 (2010).
19. Reux, B., Weber, V., Galmier, M.-J., Borel, M., Madesclaire, M., Madelmont, J.-C., Debiton, E. & Coudert, P. Synthesis and cytotoxic properties of new fluorodeoxyglucose-coupled chlorambucil derivatives, *Bioorg. Med. Chem.* **16(9)**, 5004–5020 (2008).
20. Wang, J., Hu, J., Liao, P., Xue, S., He, S., Chen, R., Zhao, X., Liu, W. The

synthesis of biphasic metabolites of carfentanil. *Molecules* **28**, 7625 (2023).

21. Kim, S. & Nagorny, P. Electrochemical synthesis of glycosyl fluorides using sulfur(VI) hexafluoride as the fluorinating agent. *Org. Lett.* **24(12)**, 2294–2298 (2022).
22. Wen, P., Simmons, C. J., Ma, Z-x., Blaszczyk, S. A., Balzer, P. G., Ye, W., Duan, X., Wang, H-Y., Yin, D., Stevens, C. M. & Tang, W. Synthesis of glycosyl chlorides and bromides by chelation assisted activation of picolinic esters under mild neutral conditions. *Org. Lett.* **22(4)**, 1495–1498 (2020).
23. Herde, Z.D., John, P.D., Alvarez-Fonseca, D., Satyavolu, J. & Burns, C.T. Stereoselective acetylation of hemicellulosic C5-sugars. *Carbohydr. Res*. **443**, 1–14 (**2017**).
24. Chen, M., Huang, C., He, C., Zhu, W., Xu, Y. & Lu, Y. A glucose-responsive controlled release system using glucose oxidase-gated mesoporous silica nanocontainers. *Chem. Commun*., **48**, 9522-9524 (2012).
25. Tojino, M., Hirose, Y. & Mizuno, M. Convenient synthesis of glycosyl bromide from 1-O-acetyl sugars by photo-irradiative phase-vanishing reaction of molecular bromine. *Tetrahedron Lett*. **54**, 7124-7126 (2013).
26. Han, Z., Zheng, Z., Cai, L., Zhou, D., Li, C., Sui, Q., Liu, S. & Gao, Q. Synthesis of flavonoid 2-deoxyglucosides via the Mitsunobu reaction. *Tetrahedron. Lett*. **59(42)**, 3773-3776 (2018).
27. Indurugalla, D. & Bennet, A. J. A Kinetic isotope effect study on the hydrolysis reactions of methyl xylopyranosides and methyl 5-thioxylopyranosides: oxygen versus sulfur stabilization of carbenium ions. *J. Am. Chem. Soc*. **123(44)**, 10889–10898 (2001).
28. Chen, Y., Yi, X., Cheng, Y., Huang, A., Yang, Z., Zhao, X., Ling, F. & Zhong, W. Rh-Catalyzed highly enantioselective hydrogenation of functionalized olefins with chiral ferrocenylphosphine-spiro phosphonamidite ligands. *J. Org. Chem.* **87**, 7864–7874 (2022).
29. Lee, C. C.; Jia, Y.; Li, N.; Sun, X.; Ng, K.; Ambing, E.; Gao, M.-Y.; Hua, S.;

Chen, C.; Kim, S.; Michellys, P.-Y.; Lesley, S. A.; Harris, J. L.; Spraggon, G. Crystal structure of the ALK (anaplastic lymphoma kinase) catalytic domain. *Biochem J* **430**, 425–437 (2010).
