# Supplementary material for: Stereoselective chemical N-glycoconjugation of amines via CO2 incorporation
Source: Nat Commun. 2024 Nov 29;15:10373. doi: 10.1038/s41467-024-54523-4 (PMC11606974; doi:10.1038/s41467-024-54523-4)
Supplement: Supplementary file 2 — Reporting Summary [file 41467_2024_54523_MOESM2_ESM.pdf]

nature portfolio

Corresponding author(s): Yu Tang ; Biao Yu

Last updated by author(s): Nov 4, 2024

Reporting Summary

Nature Portfolio wishes to improve the reproducibility of the work that we publish. This form provides structure for consistency and transparency in reporting. For further information on Nature Portfolio policies, see our Editorial Policies and the Editorial Policy Checklist.

Statistics

For all statistical analyses, confirm that the following items are present in the figure legend, table legend, main text, or Methods section.

n/a	Confirmed	
☐	☒	The exact sample size (*n*) for each experimental group/condition, given as a discrete number and unit of measurement
☐	☒	A statement on whether measurements were taken from distinct samples or whether the same sample was measured repeatedly
☒	☐	The statistical test(s) used AND whether they are one- or two-sided<br>*Only common tests should be described solely by name; describe more complex techniques in the Methods section.*
☒	☐	A description of all covariates tested
☒	☐	A description of any assumptions or corrections, such as tests of normality and adjustment for multiple comparisons
☐	☒	A full description of the statistical parameters including central tendency (e.g. means) or other basic estimates (e.g. regression coefficient) AND variation (e.g. standard deviation) or associated estimates of uncertainty (e.g. confidence intervals)
☒	☐	For null hypothesis testing, the test statistic (e.g. *F*, *t*, *r*) with confidence intervals, effect sizes, degrees of freedom and *P* value noted<br>*Give P values as exact values whenever suitable.*
☒	☐	For Bayesian analysis, information on the choice of priors and Markov chain Monte Carlo settings
☒	☐	For hierarchical and complex designs, identification of the appropriate level for tests and full reporting of outcomes
☒	☐	Estimates of effect sizes (e.g. Cohen's *d*, Pearson's *r*), indicating how they were calculated

Our web collection on statistics for biologists contains articles on many of the points above.

Software and code

Policy information about availability of computer code

Data collection	no software was used.
Data analysis	no software was used.

For manuscripts utilizing custom algorithms or software that are central to the research but not yet described in published literature, software must be made available to editors and reviewers. We strongly encourage code deposition in a community repository (e.g. GitHub). See the Nature Portfolio guidelines for submitting code & software for further information.

Data

Policy information about availability of data

All manuscripts must include a data availability statement. This statement should provide the following information, where applicable:

- Accession codes, unique identifiers, or web links for publicly available datasets
- A description of any restrictions on data availability
- For clinical datasets or third party data, please ensure that the statement adheres to our policy

The data reported in this paper are available within the article and its Supplementary Information files. All data are available from the corresponding author upon request.

Research involving human participants, their data, or biological material

Policy information about studies with human participants or human data. See also policy information about sex, gender (identity/presentation), and sexual orientation and race, ethnicity and racism.

Reporting on sex and gender	Not applicable.
Reporting on race, ethnicity, or other socially relevant groupings	Not applicable.
Population characteristics	Not applicable.
Recruitment	Not applicable.
Ethics oversight	Not applicable.

Note that full information on the approval of the study protocol must also be provided in the manuscript.

Field-specific reporting

Please select the one below that is the best fit for your research. If you are not sure, read the appropriate sections before making your selection.

☒ Life sciences ☐ Behavioural & social sciences ☐ Ecological, evolutionary & environmental sciences

For a reference copy of the document with all sections, see nature.com/documents/nr-reporting-summary-flat.pdf

Life sciences study design

All studies must disclose on these points even when the disclosure is negative.

Sample size	The sample size for each experiment was chosen based on related published work (1,2) 1) J. Jean Cui et al. Structure Based Drug Design of Crizotinib (PF-02341066), a Potent and Selective Dual Inhibitor of MesenchymalEpithelial Transition Factor (c-MET) Kinase and Anaplastic Lymphoma Kinase (ALK) J. Med. Chem. 2011, 54, 6342–6363; 2) R. W. Sparidans. et al. Liquid chromatography–tandem mass spectrometric assay for the ALK inhibitor crizotinib in mouse plasma. J. Chromatogr. B 905 (2012) 150–154
Data exclusions	No data were exculded.
Replication	All in vitro and cellular experiments were performed in at least 2 indenpendent experiments (N = 2) with biological replicates (n = 2). All attempts at replication were successful.
Randomization	The animals were randomly distributed during pharmacokinetic experiments. Randomization was not applicable for other experiments. All experiments were carried out with appropriate internal and/or positive controls as indicated.
Blinding	The investigators were not blinded because collection or analysis of the presented data was not prone to bias.

Reporting for specific materials, systems and methods

We require information from authors about some types of materials, experimental systems and methods used in many studies. Here, indicate whether each material, system or method listed is relevant to your study. If you are not sure if a list item applies to your research, read the appropriate section before selecting a response.

Materials & experimental systems

n/a	Involved in the study	
☒	☐	Antibodies
☐	☒	Eukaryotic cell lines
☒	☐	Palaeontology and archaeology
☐	☒	Animals and other organisms
☒	☐	Clinical data
☒	☐	Dual use research of concern
☒	☐	Plants

Methods

n/a	Involved in the study	
☒	☐	ChIP-seq
☒	☐	Flow cytometry
☒	☐	MRI-based neuroimaging

Eukaryotic cell lines

Policy information about cell lines and Sex and Gender in Research

Cell line source(s)	Cancer cell lines, including human gastric SNU-5 cell lines, human embryonic kidney 293t cell lines, human malignant melanoma SK-MEL-28 cell lines were procured from the National Collection of Authenticated Cell Cultures, China.
Authentication	All cells were kindly provided by the Cell Bank of the Chinese Academy of Sciences and have been certified through STR analysis.
Mycoplasma contamination	All cells were tested using HiScript III All-in-one RT SuperMix Perfect for qPCR (Vazyme, Cat: R333-01), and the test results were negative for mycoplasma contamination.
Commonly misidentified lines (See ICLAC register)	No commonly misidentified cell lines were used

Animals and other research organisms

Policy information about studies involving animals; ARRIVE guidelines recommended for reporting animal research, and Sex and Gender in Research

Laboratory animals	male C57 mouse
Wild animals	This study did not involve wild animals.
Reporting on sex	male
Field-collected samples	The animals were maintained in cages at 22 ± 3 °C and 55% relative humidity under a 12 h dark/light cycle. Rats were allowed free access to water but were fasted for 12 h before drug administration.
Ethics oversight	All procedures in the animal studies were performed inaccordance with the Guide for the Care and Use of Laboratory Animals of Shanghai Institute of Materia Medica, Chinese Academy of Sciences.

Note that full information on the approval of the study protocol must also be provided in the manuscript.

Plants

Seed stocks	The study did not involve plants.
Novel plant genotypes	The study did not involve plants.
Authentication	The study did not involve plants.
